# The HDAC inhibitor SAHA regulates CBX2 stability via a SUMO-triggered ubiquitin-mediated pathway in leukemia

**DOI:** 10.1038/s41388-018-0143-1

**Published:** 2018-02-22

**Authors:** Antonella Di Costanzo, Nunzio Del Gaudio, Lidio Conte, Carmela Dell’Aversana, Michiel Vermeulen, Hugues de Thé, Antimo Migliaccio, Angela Nebbioso, Lucia Altucci

**Affiliations:** 1 Department of Biochemistry, Biophysics and General Pathology, University of Campania “Luigi Vanvitelli”, Vico L. De Crecchio 7, 80138 Napoli, Italy; 2 Department of Molecular Biology, Faculty of Science, Radboud Institute for Molecular Life Sciences, Radboud University, 6500 HB Nijmegen, The Netherlands; 3 INSERM Unite ´ Mixte de Recherche 944, Equipe labellisée par la Ligue Nationale contre le Cancer, Institut Universitaire d’Hématologie, Hôpital St. Louis, Paris Cedex 10, France

## Abstract

Polycomb group (PcG) proteins regulate transcription, playing a key role in stemness and differentiation. Deregulation of PcG members is known to be involved in cancer pathogenesis. Emerging evidence suggests that CBX2, a member of the PcG protein family, is overexpressed in several human tumors, correlating with lower overall survival. Unraveling the mechanisms regulating CBX2 expression may thus provide a promising new target for anticancer strategies. Here we show that the HDAC inhibitor SAHA regulates CBX2 stability via a SUMO-triggered ubiquitin-mediated pathway in leukemia. We identify CBX4 and RNF4 as the E3 SUMO and E3 ubiquitin ligase, respectively, and describe the specific molecular mechanism regulating CBX2 protein stability. Finally, we show that CBX2-depleted leukemic cells display impaired proliferation, underscoring its critical role in regulating leukemia cell tumorogenicity. Our results show that SAHA affects CBX2 stability, revealing a potential SAHA-mediated anti-leukemic activity though SUMO2/3 pathway.

## Introduction

SUMOylation is a post-translational modification (PTM) that regulates target protein function, playing a critical role in cellular processes such as DNA damage response, cell cycle progression, apoptosis, and cellular stress response [1–3]. Small ubiquitin-like modifier (SUMO) proteins are involved in several cancers, including leukemia [4], functioning as either oncogenes or oncosuppressors in a cell context-dependent manner [5–7]. Leukemias are characterized by bone marrow failure due to oncogenic mutations of hematopoietic stem cells (HSC) or blood precursor cells. HSC differentiation and self-renewal properties are tightly regulated by Polycomb group (PcG) proteins, a well-characterized family of transcriptional epigenetic regulators [8]. PcG proteins form two canonical complexes: Polycomb repressive complex 1 (PRC1), which mediates ubiquitination of H2A at lysine 119 (H2AK119ub), and Polycomb repressive complex 2 (PRC2), which trimethylates H3 at lysine 27 (H3K27me3) [9]. Non-canonical PRC1 complexes have also been described, and are emerging as regulators of gene transcription [10]. Mechanistically, the hierarchical model of PcG-mediated gene silencing requires H3K27 trimethylation by PRC2 followed by binding of PRC1 via one of the five chromobox proteins (CBX2, 4, 6, 7, 8), which in turns triggers H2AK119ub, eventually leading to transcriptional repression [11, 12]. Unsurprisingly, as regulators of stem cell properties and blood cell differentiation, PcG proteins are involved in leukemia and other solid cancers [13–15].

CBX proteins link the activity of PRC1 with PRC2, serving as critical regulators of PcG-mediating activity. While the functional role of some CBX proteins in cancer has been largely described [15–17], recent reports support the specific role of CBX2 in human tumors. CBX2 is overexpressed in several human cancers. Genotranscriptomic meta-analysis of CBX2 revealed its amplification and upregulation in breast, lung, colorectal, prostate, brain, and hematopoietic tumors compared to normal tissue highlighting its potential oncogenic role [18]. Increased CBX2 expression has also been correlated with lower overall survival, whereas CBX2 depletion negatively affects prostate tumor proliferation and progression [18, 19]. CBX2 may thus represent a promising new target for anticancer strategies, warranting a better understanding of the mechanisms regulating CBX2 stability and biological activity. To date, chromodomain inhibitors have been identified for CBX7 [20, 21], but no molecules inhibiting CBX2 have been described. Nevertheless, different chromatin-modulating drugs such as histone deacetylase inhibitors (HDACi) are reported to regulate CBX2 targets on chromatin, suggesting that HDACi might be used to indirectly modulate aberrant effects of CBX2 in cancer [22]. Furthermore, the well-known pan-HDACi SAHA was recently shown to alter the profile of the whole proteome, modulating several PTM pathways such as ubiquitination and acetylation [23]. However, the precise role of HDACi in regulating CBX2 remains to be elucidated.

Here we describe a novel SAHA-mediated mechanism of CBX2 post-translational regulation. We found that CBX2 undergoes SAHA-induced SUMO2/3 modification and that CBX2 SUMOylation promotes its ubiquitination and proteasome-dependent degradation. We also identified the specific molecular pathway and key players regulating CBX2 stability, demonstrating that CBX4 and RNF4 act as the E3 SUMO and E3 ubiquitin ligase, respectively. Additionally, CBX2-depleted leukemic cells display impaired proliferation, showing that CBX2 is required for leukemia cell clonogenicity. Our study provides the first evidence of a non-canonical SAHA-mediated anti-tumorigenic activity via CBX2 SUMOylation and degradation.

## Results

### SUMO2/3 play a functional role in SAHA-induced CBX2 destabilization in leukemia

**Fig. 1:**
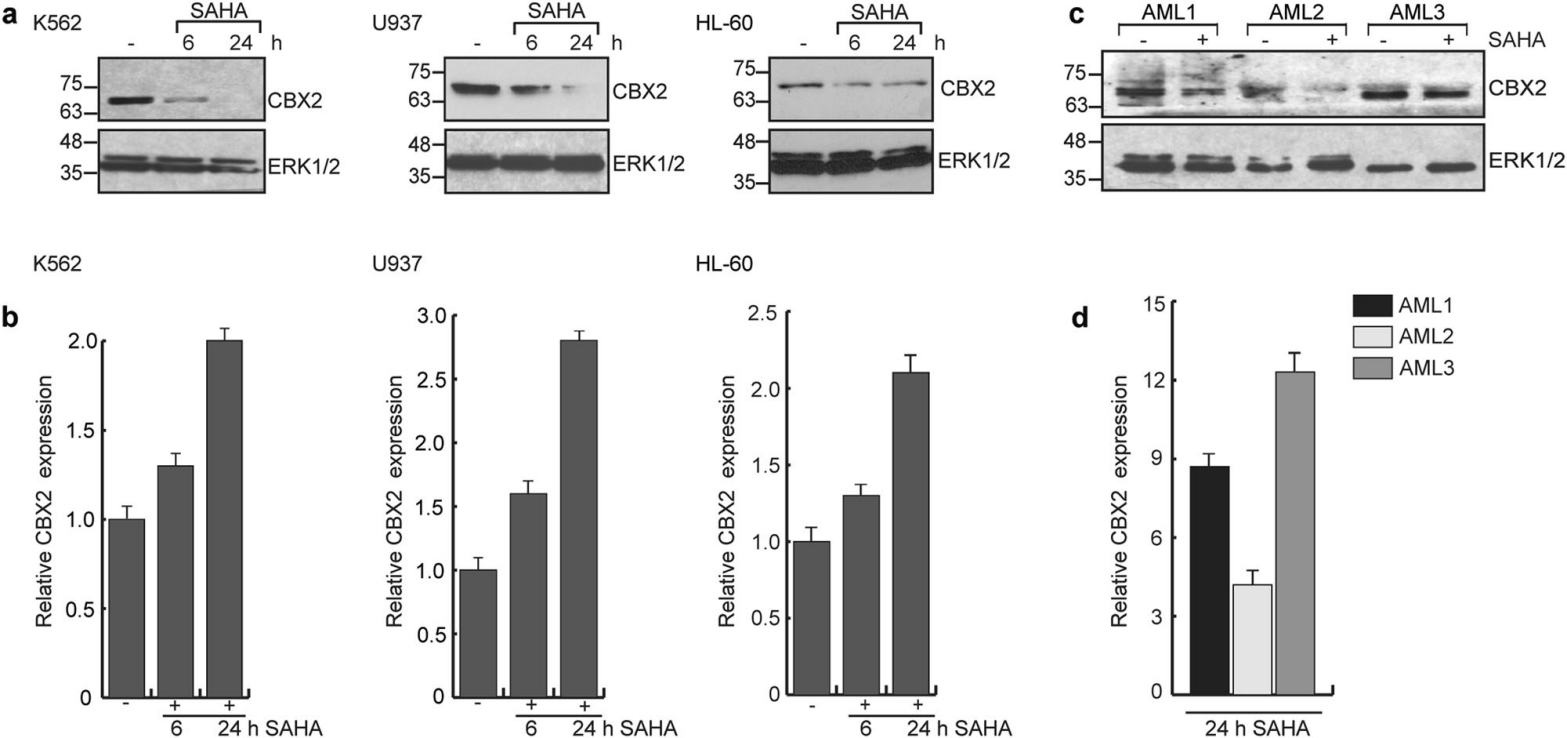
SAHA induces CBX2 degradation. **a** Western blot analysis of CBX2 in K562, U937, and HL-60 cells untreated (−) or treated with 5 μM SAHA at indicated times. ERK1/2 was used as loading control. **b** Real-time qPCR analysis for CBX2 expression in K562, U937 and HL-60 cells untreated (−) or treated with 5 μM SAHA at indicated times. Error bars indicate standard deviation (STD) of three biological replicates. p-value is not significant. **c** Western blot of CBX2 in 3 primary blasts derived from AML patients untreated (−) or treated with 5 μM SAHA at 24 h. **d** Real-time qPCR analysis of CBX2 expression levels (relative to ctr) in three primary blasts derived from AML patients untreated or treated with 5 μM SAHA at 24 h. Results show CBX2 relative fold change in SAHA-treated blasts compared to untreated counterpart. Error bars represent STD of three technical replicates. *p*-value is not significant

**Fig. 2:**
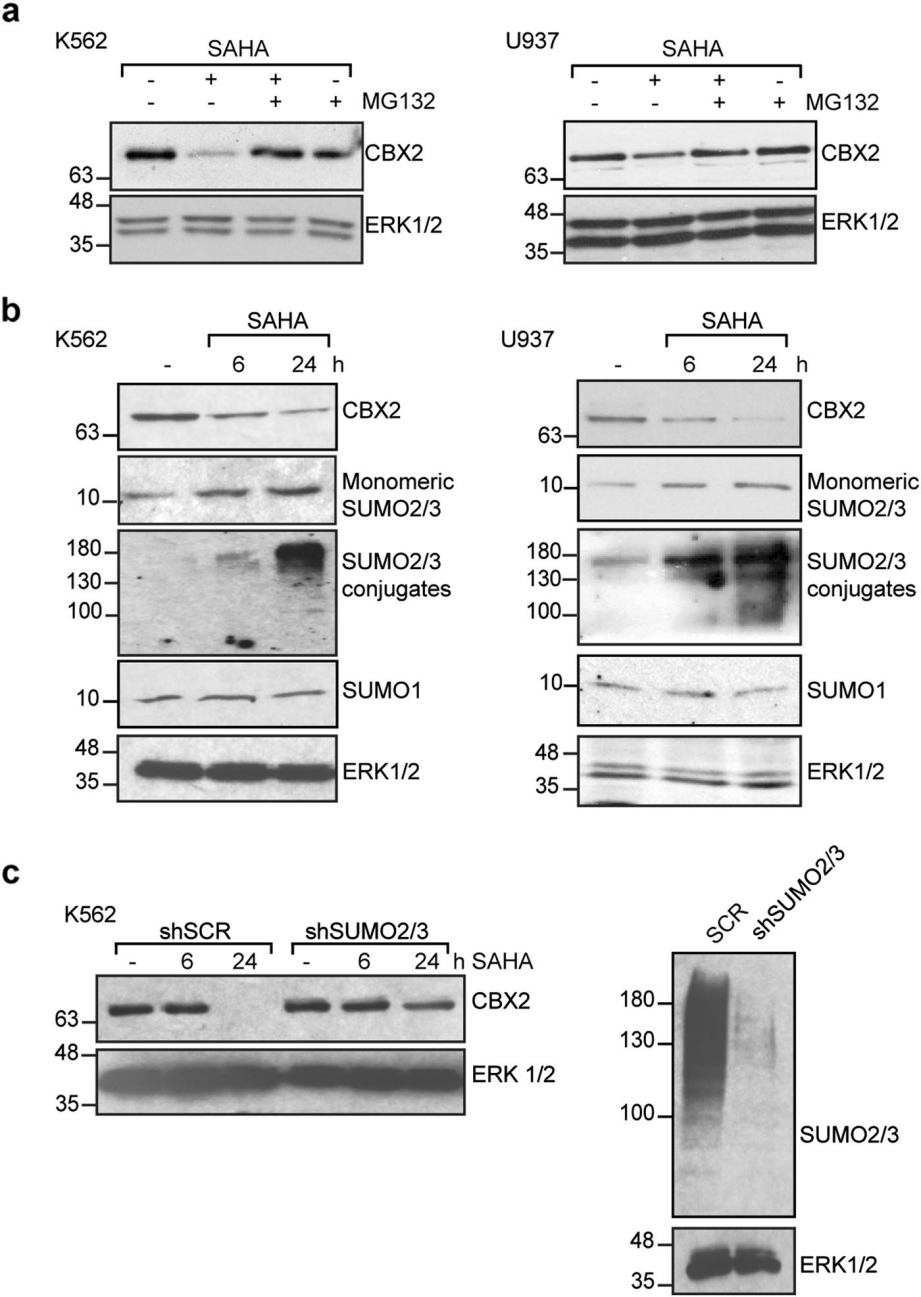
SAHA mediates CBX2 proteasome-dependent degradation and induces SUMO2/3 modification pathway in leukemic cells. **a** Western blot of endogenous CBX2 in K562 and U937 cells untreated or treated with 5 µM SAHA for 6 h alone or in combination with 25 µM MG132. ERK1/2 was used as loading control. **b** Western blot of endogenous CBX2, SUMO2/3 and SUMO1 expression after SAHA treatment at indicated times in K562 and U937 cells. **c** CBX2 levels in K562 cells stably expressing shSUMO2/3 and shSCR control treated with 5 µM SAHA at 6 and 24 h. SUMO2/3 protein levels in shSUMO2/3-transduced K562 cells compared to shSCR control are shown

HDACi regulate CBX2 targets on chromatin [22], suggesting that they might indirectly modulate CBX2 in leukemia. To investigate the effect of SAHA on CBX2 expression, we treated K562, U937 and HL-60 cells with SAHA (5 µM) at different times. Western blot analysis showed CBX2 downregulation in all cell lines tested in a time-dependent manner (Fig. 1a). qRT-PCR experiments showed that SAHA does not exert its effect transcriptionally (Fig. 1b), as previously described for many SAHA target genes [24], suggesting that SAHA acts via post-translational mechanisms. Similarly, CBX2 destabilization was also observed in SAHA-treated ex vivo primary AML blasts at protein (Fig. 1c) but not RNA level (Fig. 1d). To investigate the mechanisms underlying CBX2 destabilization, we performed western blot analysis of K562 and U937 cells treated with the proteasome inhibitor MG132 (Fig. 2a). Our results showed that SAHA promotes CBX2 downregulation via a proteasome-dependent pathway. Interestingly, in addition to CBX2 degradation, SAHA treatment increased endogenous expression of SUMO2/3 (but not SUMO1) and its conjugates in a time-dependent manner (Fig. 2b). We therefore speculated that CBX2 SUMOylation is responsible for SAHA-mediated CBX2 degradation.

To study the involvement of SUMO2/3 pathway in CBX2 degradation after SAHA treatment, we knocked down endogenous SUMO2/3 in K562 cells. SUMO2/3-depleted K562 cells were treated with 5 µM SAHA for 6 and 24 h. Western blot analysis revealed that SUMO2/3 silencing limits SAHA-mediated CBX2 degradation compared to shSCR negative control (Fig. 2c). Moreover, treatment of leukemic cells with other epi-drugs, including also non-HDACi, showed that HDACi-mediated induction of SUMO 2/3 pathway seems to be a required step to promote CBX2 protein degradation (Supplementary Fig. 1a).

Together, these findings suggest that, in leukemic cells, SAHA promotes activation of SUMO2/3 modification pathway, which seems to play a functional role in SAHA-mediated CBX2 destabilization.

### CBX2 undergoes SUMOylation

To investigate whether SUMO-mediated CBX2 modifications are involved in SAHA-induced CBX2 degradation, we first analyzed CBX2 sequence to identify putative SUMOylation sites. CBX2 sequence analysis using SUMOsp 2.0 software revealed that CBX2 is endowed with several potential SUMOylation sites, including K153, which lies in a canonical SUMO consensus sequence [25] (Supplementary Fig. 1b). To determine whether CBX2 is endogenously SUMOylated in leukemic cells, we performed western blot analysis on U937 and K562 cell extracts. We observed three different forms of CBX2: the predicted form at 70 kDa and two slower migrating forms at about 90 and 130 kDa (Supplementary Fig. 2a). CBX2 knockdown led to a reduction in both slower migrating bands, confirming that they are specific forms of CBX2 (Supplementary Fig. 2b). Moreover, SUMO2/3 depletion in K562 cells confirmed that CBX2 undergoes SUMO2/3 covalent modifications (Supplementary Fig. 2c). Noteworthy, SUMO2/3 knockdown causes CBX2 stabilization.

**Fig. 3:**
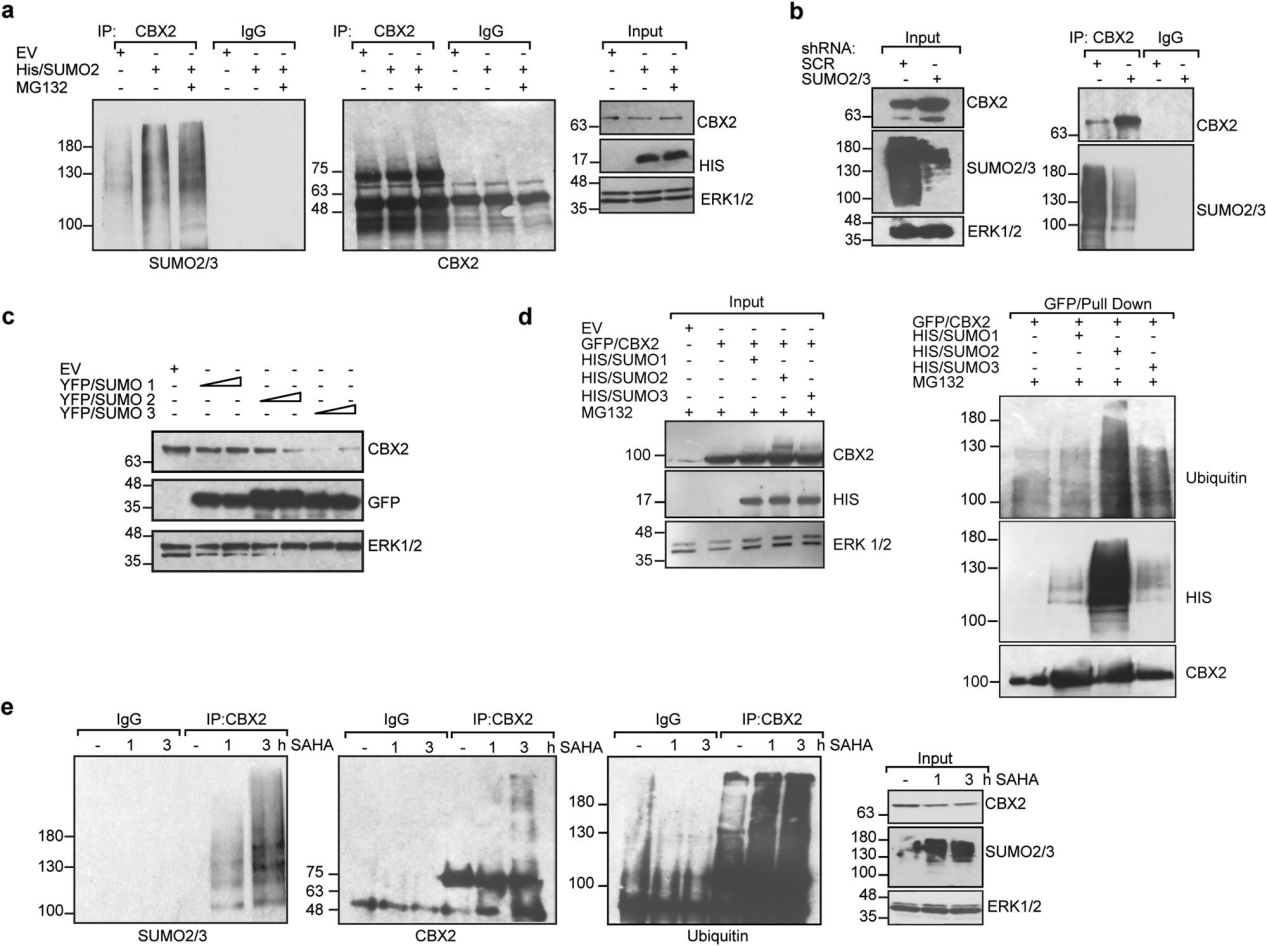
CBX2 is SUMOylated by SUMO2/3 promoting its polyubiquitination and degradation. **a** IP experiment on endogenous CBX2 performed in HEK293-FT cells overexpressing His/SUMO2 (3 µg) with or without 25 µM MG132. Immunoblotting was performed using indicated antibodies. **b** Immunoblot analysis of immunoprecipitated cell lysate from K562 shSUMO2/3 transduced cells. IP was performed against endogenous CBX2 followed by immunoblotting with anti-SUMO2/3 antibody. **c** Western blot of endogenous CBX2 in HEK293-FT cells upon overexpression of increasing amount (1 or 2 µg) of YFP/SUMO1, 2 or 3. ERK1/2 was used as loading control. **d** GFP-Pull-down assaying CBX2 polyubiquitination and SUMOylation in HEK293-FT cells upon transfection of GFP/CBX2 alone or in combination with His/SUMO1-, His/SUMO2-, His/SUMO3- encoding plasmids (3 µg) in presence of 25 µM MG132. IP experiments were performed with anti-CBX2 antibody and immunoblotted with anti-ubiquitin and anti-HIS antibodies in HEK293-FT cells. **e** Endogenous CBX2 SUMOylation and polyubiquitination status in SAHA-treated K562 cells at indicated times. Total extracts were immunoprecipitated with anti-CBX2 antibody. Immunoblotting was performed with indicated antibodies

Taken together, these results indicate that CBX2 is endogenously SUMOylated by SUMO2/3 in leukemic cells. To corroborate that CBX2 undergoes SUMO2/3 modifications, we performed IP experiments of endogenous CBX2 and pull-down of His/SUMO2/3-associated proteins with Ni^2+^ beads. HEK293-FT cells were transiently transfected with His/SUMO2 expression plasmid with or without MG132 to overcome CBX2 destabilization. IP followed by immunoblotting analysis revealed that endogenous CBX2 undergoes polySUMOylation upon SUMO2 overexpression, and that its SUMOylation status increases with MG132 proteasome inhibitor treatment (Fig. 3a). Similarly, IP experiments upon SUMO2/3 depletion showed a reduction in CBX2-SUMO2/3 conjugates (Fig. 3b). Nickel pull-down assay also confirmed that CBX2 is SUMOylated by SUMO2 (Supplementary Fig. 3a).

Together, these findings demonstrate that CBX2 is SUMOylated by SUMO2/3 in vivo.

### SUMO2/3 modification of CBX2 triggers its polyubiquitination and proteasomal degradation

To investigate the molecular function of SUMO-mediated CBX2 modification, we transfected HEK293-FT cells with increasing amounts of YFP-SUMO1, 2 or 3. In line with our results, overexpression of either SUMO2 or SUMO3 but not SUMO1 reduced CBX2 expression in a dose-dependent manner (Fig. 3c). Since SUMOylation regulates gene expression and chromatin dynamics, we performed real-time qPCR experiments to investigate whether SUMO2 transcriptionally modifies CBX2 expression. Our results showed that SUMO2 alters CBX2 expression at post-translational level only, since no effect was observed on its mRNA (Supplementary Fig. 3b). SUMO2/3 conjugation and the ubiquitin-proteasome system cooperate to regulate the stability of a subset of SUMO2/3-conjugated proteins [26]. Thus, to determine whether the ubiquitin-proteasome system is involved in SUMO2/3-mediated CBX2 degradation, we transfected HEK293-FT cells with increasing amounts of His/SUMO2 plasmid with or without MG132. Western blot analysis revealed that MG132 blocks SUMO-dependent CBX2 degradation, providing evidence that this phenomenon occurs through a ubiquitin-proteasome pathway (Supplementary Fig. 4a). To examine the possible interplay between SUMOylation and ubiquitination in regulating CBX2 turnover, we performed GFP/Pull-down on exogenous GFP/CBX2 to assay its ubiquitination rates following SUMO1, SUMO2 and SUMO3 overexpression. Western blotting using anti-ubiquitin antibody showed that CBX2 was polyubiquitinated exclusively by SUMO2 and SUMO 3. Although SUMO1 seems to slightly modify CBX2, its modification is not responsible for CBX2 polyubiquitination (Fig. 3d). Moreover, SUMO2-mediated ubiquitination was also observed upon IP experiments of endogenous CBX2 (Supplementary Fig. 4b).

Likewise, SUMO2/3 knockdown decreased CBX2 polyubiquitination compared to non-targeting shRNA-expressing cells (Supplementary Fig. 4c).

These findings indicate that CBX2 SUMOylation promotes its ubiquitination and proteasome-mediated degradation. As SAHA treatment promotes both CBX2 degradation and SUMO2/3 induction, we assayed CBX2 SUMOylation and ubiquitination status after SAHA treatment. K562 cells were treated with SAHA (5 µM) at 1 and 3 h instead of 6 and 24 h to avoid degradation of CBX2 and its SUMOylated forms. IP of endogenous CBX2 after SAHA treatment followed by immunoblotting with anti-SUMO2/3 and anti-ubiquitin antibodies, respectively, showed that SAHA promotes CBX2 polySUMOylation and polyubiquitination (Fig. 3e).

These results provide evidence that SAHA affects CBX2 expression by inducing SUMO2/3 pathway. CBX2 SUMOylation by SUMO2 and SUMO3 causes its polyubiquitination and proteasome-dependent degradation, revealing a novel and non-canonical mechanism by which SAHA regulates CBX2 expression.

### Lysines K60, K153 and K410 are SUMOylated by SUMO2/3 and govern CBX2 stability

**Fig. 4:**
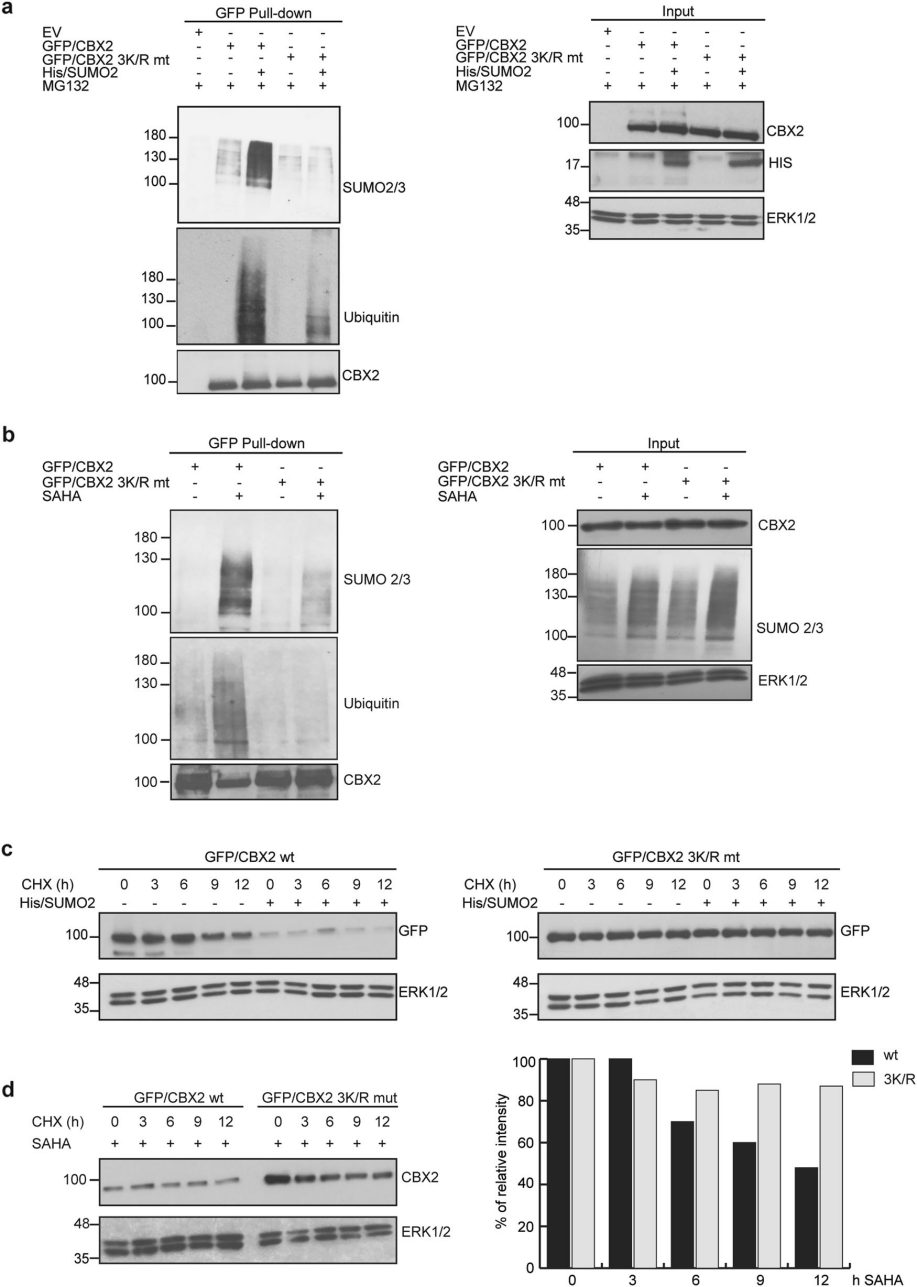
Lysine residues in CBX2 responsible for SUMO2/3 conjugation. **a** GFP-Pull-down of wt CBX2 and 3 K/R lysine mutant overexpressing His-tagged SUMO 2 (3 µg) in HEK293-FT cells, in presence of MG132. Immunoblotting was performed with indicated antibodies to assay SUMOylation and ubiquitination rates. **b** GFP-Pull-down of wt CBX2 and 3 K/R mutant in K562 cells untreated or treated with 5 µM SAHA for 3 h. Immunoblotting was performed with indicated antibodies to assay SUMOylation and ubiquitination rates. **c** Western blot analysis of wt CBX2 (0,5 µg) and 3 K/R (0,5 µg) mutant protein half-life following His/SUMO2 overexpression (1 µg). CHX was added at indicated times. **d** Western blot analysis of wt CBX2 (0.5 µg) and 3 K/R (0,5 µg) mutant protein half-life upon SAHA treatment (5 µM) for 24 h in HEK293-FT cells. CHX was added at indicated times. Quantitative densitometry analysis is shown

SUMOylation commonly occurs on specific lysine residues falling in the so-called SUMO-acceptor site ΨKxE (where Ψ is an aliphatic branched amino acid and x is any amino acid), although many proteins are SUMOylated through non-canonical lysine residues [27]. CBX2 sequence analysis using SUMOsp 2.0 software identified several canonical and non-canonical lysine (K) residues as potential SUMOylation sites, with K153 lying in a canonical SUMO consensus site. To identify CBX2-SUMO2/3 acceptor site(s), we replaced, by point mutagenesis, several K residues with an arginine (R), inserting CBX2 K/R mt in GFP-tagged vectors. wt and mt GFP/CBX2 were transfected with His/SUMO2 in HEK293-FT cells. GFP pull-down followed by western blot using anti-SUMO2/3 antibody identified three K residues, K60, K153, and K410, which partially impair CBX2 SUMOylation when replaced by R (Supplementary Fig. 5a). We therefore constructed a triple CBX2-3K/R mt in which all three lysines were replaced. MG132-treated HEK293-FT cells were co-transfected with SUMO2/3 and either wt CBX2- or CBX2-3K/R-expressing plasmids. IP assays followed by western blot with anti-SUMO2/3 and anti-ubiquitin antibodies showed that CBX2 SUMOylation was completely abolished in CBX2-3K/R (Fig. 4a). As expected, GFP pull-down and western blot of CBX2-3K/R showed impairment of its polyubiquitination (Fig. 4a). Accordingly, 3 K/R mutant also showed lower SUMOylation and ubiquitination rate upon SAHA treatment (Fig. 4b).

To evaluate the effect of SUMO2/3 on CBX2-3K/R stability, we measured the turnover rate of wt and triple mt CBX2 upon SUMO2/3 overexpression using CHX. HEK293-FT cells were transfected with GFP-tagged wt CBX2 and CBX2-3K/R with or without a fixed amount of SUMO2/3. After transfection (24 h), cells were treated with CHX at different times. While the protein half-life of wt CBX2 was around 9 h, CBX2-3K/R was much more stable. Furthermore, SUMO2/3 overexpression almost completely eliminated wt CBX2 expression after around 3 h, while CBX2-3K/R retained its stability (Fig. 4c). Next, we tested the effect of SAHA treatment on CBX2-3K/R. K562 cells were nucleofected with GFP-tagged wt and triple mt CBX2. After nucleofection (24 h), cells were treated with SAHA at the indicated times. Western blot analysis followed by immunoblotting with anti-GFP antibody showed that SAHA robustly promotes degradation of wt CBX2, but has a much reduced effect on CBX2-3K/R (Supplementary Fig. 5b). Furthermore, wt and CBX2-3K/R half-life was also measured upon SAHA treatment using CHX. HEK293-FT cells were transfected with CBX2 wt or 3 K/R mutant and then treated with 5 µM SAHA. 24 h upon SAHA treatment, CBX2 wt and 3 K/R mutant protein decay were assayed adding CHX at different time points. Results showed that SAHA strongly reduces CBX2 wt protein expression. Conversely, 3 K/R mutant is much more stable showing a differential rate of decay compared to CBX2 wt protein (Fig. 4d).

These findings indicate that K60, K153 and K410 are key residues for SAHA-mediated CBX2 degradation.

Taken together, our data demonstrate that these three lysines are responsible for CBX2 SUMOylation by SUMO2/3, governing its stability.

### RNF4 is the E3 ubiquitin ligase of SUMOylated CBX2

**Fig. 5:**
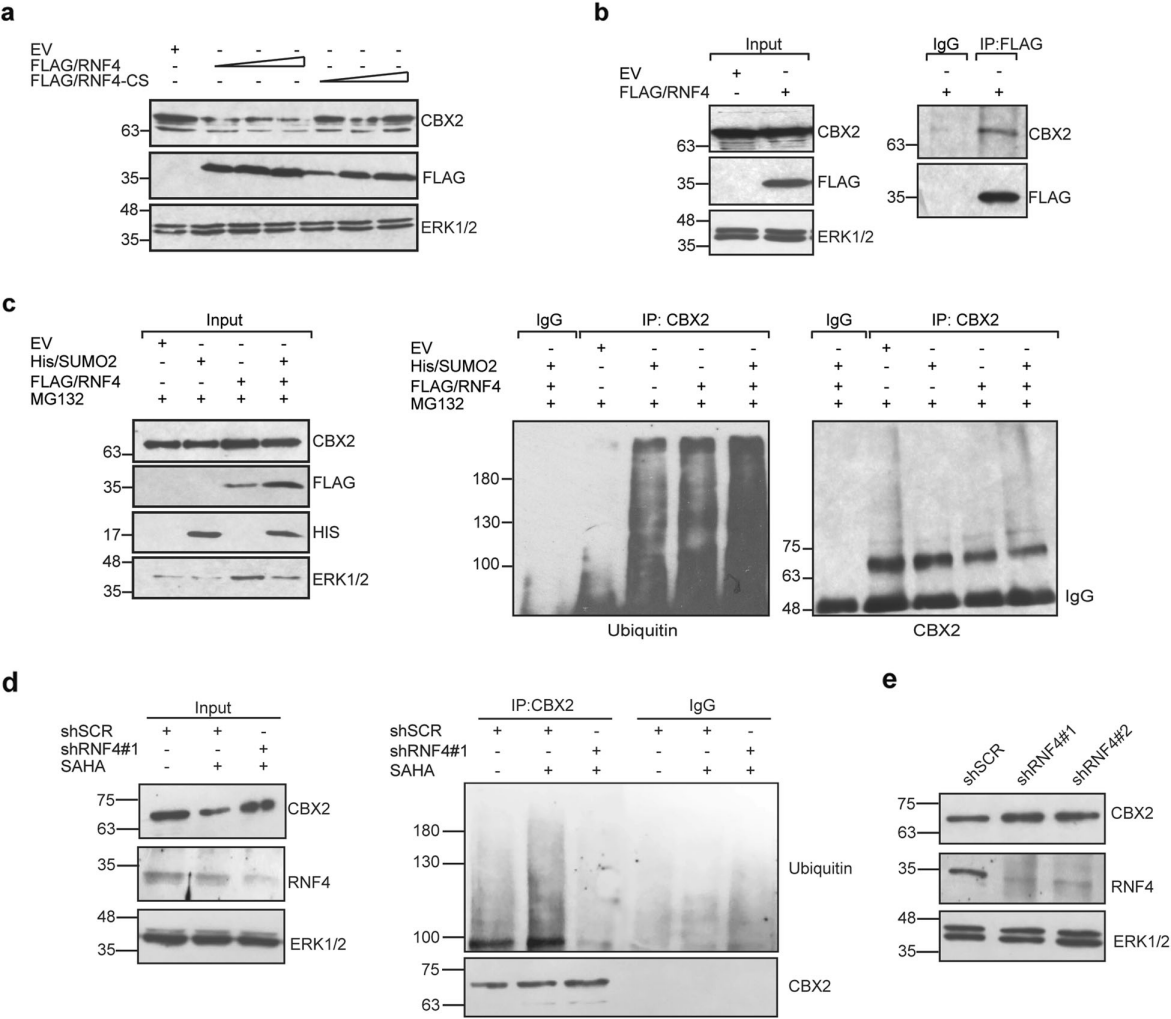
RNF4 acts as E3 ubiquitin ligase of SUMOylated CBX2. **a** Western blot of endogenous CBX2 following transfection of increasing amounts of wt FLAG/RNF4 or FLAG/RNF4-CS (0,5, 1 or 2 µg) in HEK293-FT cells. **b** IP of endogenous CBX2 upon FLAG/RNF4 overexpression in HEK293-FT cells. **c** IP of endogenous CBX2 following transfection of either His/SUMO2 (3 µg) or FLAG/RNF4 alone or in combination. Immunoblotting was performed with indicated antibodies. **d** Endogenous CBX2 polyubiquitination status K562 stably expressing shRNF4 construct #1 and shSCR control treated with 5 µM SAHA at 3 cells Total extracts were immunoprecipitated with anti-CBX2 antibody. CBX2 polyubiquitination rate in SAHA-treated shRNF4#1-transduced K562 cells compared to shSCR control are shown. **e** Western blot analysis of endogenous CBX2 in K562 cells stably expressing two different shRNA construct targeting shRNF4 (#1 and #2) or shSCR control. ERK1/2 was used as loading control

Human RNF4 protein is one of the most extensively studied SUMO-targeted ubiquitin ligases. To determine whether RNF4 is involved in CBX2 degradation, we transfected HEK293-FT cells with increasing amounts of FLAG-tagged RNF4 and its mutant form, RNF4-CS, lacking E3 ubiquitin ligase activity. Western blot analysis revealed that RNF4 mediates CBX2 degradation, while RNF4-CS does not affect CBX2 stability, suggesting that RNF4 E3 ubiquitin ligase activity is required to promote CBX2 degradation (Fig. 5a).

To verify whether RNF4 and CBX2 interact, we immunoprecipitated CBX2 following FLAG-tagged RNF4 overexpression. Our results show that CBX2 physically interacts with RNF4 (Fig. 5b).

We then investigated the involvement of RNF4 in SUMO-triggered CBX2 polyubiquitination. HEK293-FT cells were transfected with His/SUMO2 and FLAG-tagged RNF4 plasmids, and treated with MG132.

Following CBX2 IP, immunoblotting with anti-ubiquitin antibody showed that RNF4 promotes CBX2 polyubiquitination. Moreover, RNF4 and SUMO2 co-expression synergistically caused a strong increase in CBX2 polyubiquitinated forms compared to either SUMO2 or RNF4 alone (Fig. 5c). To investigate the role of RNF4 in SAHA-mediated CBX2 polyubiquitination HEK293-FT cells were cotransfected with GFP/CBX2, FLAG-tagged RNF4 or RNF4-CS mutant plasmids and treated with SAHA and MG132. GFP/Pull-down assay of CBX2 followed by immunoblotting with anti-Ubiquitin showed that RNF4, but not its catalytic mutant, promotes CBX2 ubiquitination upon SAHA treatment (Supplementary Fig. 6a). In parallel, SAHA-treated K562 cells knocked down for RNF4 showed an impairment of CBX2 polyubiquitination compared to SCR control cells (Fig. 5d). Accordingly, silencing of RNF4 promote CBX2 protein stabilization (Fig. 5e).

Taken together, these results indicate that RNF4 acts as the ubiquitin ligase involved in SAHA-mediated CBX2 ubiquitination and degradation of SUMOylated CBX2.

### CBX4 SUMO E3 ligase mediates CBX2 SUMOylation

**Fig. 6:**
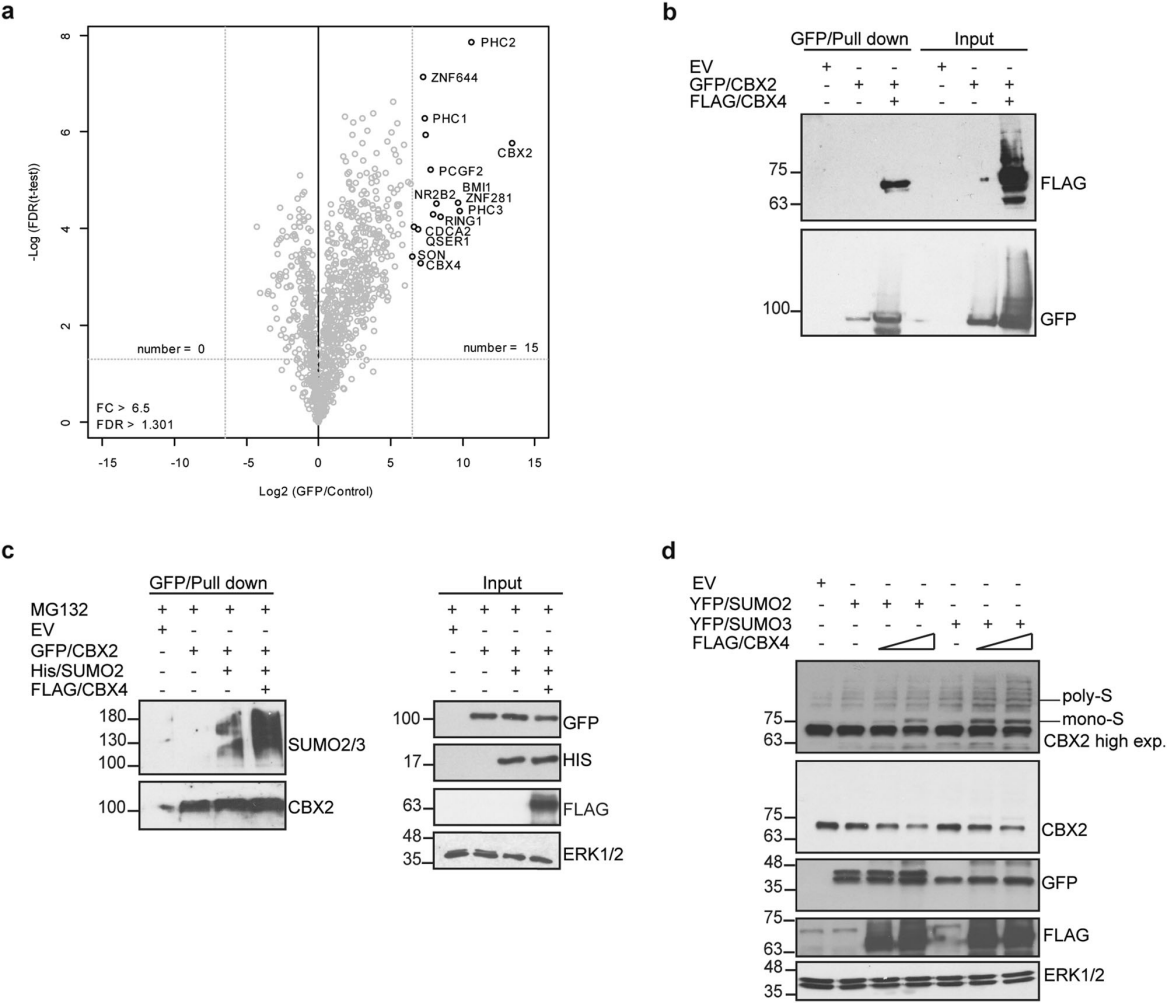
CBX4 E3 SUMO ligase mediates SUMO modification of CBX2. **a** Volcano plot from label-free GFP pull-down of GFP/CBX2 HeLa cell nuclear extracts. Bait and its interactors are shown in the upper right corner. Statistically, enriched proteins in GFP/CBX2 pull-down were identified by a permutation-based FDR-corrected *t*-test. Label-free quantification intensity of GFP pull-down relative to control (fold change (FC), *x* axis) is plotted against the log2-transformed p-value of *t*-test (*y* axis). **b** IP and immunoblot of GFP-tagged CBX2 and FLAG/CBX4 in HEK293-FT cells. **c** GFP pull-down and immunoblot of CBX2-SUMO2/3 conjugates upon His/SUMO2 and FLAG/CBX4 overexpression. **d** Western blot of endogenous CBX2 in HEK293-FT cells upon overexpression of a fixed amount (0.5 µg) of YFP/SUMO2 or YFP/SUMO3 alone or together to increasing amounts (1 and 2 µg) of FLAG/CBX4. Upper panel: CBX2 high exposure. Poly-S is referred to polySUMOylated forms and mono-S to monoSUMOylated form of CBX2, respectively

To identify novel CBX2 interactors, we performed MS-based quantitative interaction proteomics. We generated a transgenic inducible Tet-On HeLa stable cell line overexpressing N-terminal GFP-tagged CBX2. After doxycycline induction (24 h), nuclear extract was obtained from transgenic (Tet-On inducible GFP/CBX2) and wt HeLa cells. A single step GFP-affinity enrichment (GFP-Trap) of lysates was performed in triplicate. Affinity purification of GFP/CBX2 followed by label-free LC-MS/MS analysis revealed the interaction of CBX2 with several proteins including CBX4 (Fig. 6a and Supplementary Table 1). Human CBX4 is the only member of the canonical PRC1 complex that possesses SUMO E3 ligase activity [28]. A limited number of substrates for CBX4 are reported [29–31]. To validate LC-MS/MS results, we performed IP experiments in HEK293-FT cells after transfection of GFP-tagged CBX2 alone or with FLAG-tagged CBX4-encoding vector. IP experiments confirmed that CBX2 interacts with CBX4 (Fig. 6b). To investigate whether CBX4 has a functional role in CBX2 SUMO2/3-mediated modifications, IP experiments were conducted. HEK293-FT cells were transfected with GFP-tagged CBX2 alone, with His-tagged SUMO2, or with both SUMO2 and FLAG-tagged CBX4 plasmids. Our results show that CBX4 overexpression increases CBX2 SUMOylation, highlighting that CBX4 is required for SUMO moiety catalyzation (Fig. 6c). To test the effect of CBX4 overexpression on CBX2 stability, we transfected HEK293-FT cells with a fixed quantity of SUMO2 and SUMO3 either alone or together to increasing amounts of CBX4. Western blot analysis showed that, in presence of either SUMO2 or SUMO3, CBX4 promotes CBX2 polySUMOylation and, subsequently, its degradation in a dose-dependent manner (Fig. 6d). Our results identify CBX4 as the E3 SUMO ligase of CBX2, highlighting its involvement in SUMO2/3-dependent CBX2 degradation.

### CBX2 depletion impairs tumorigenicity and proliferation of leukemic cells

**Fig. 7:**
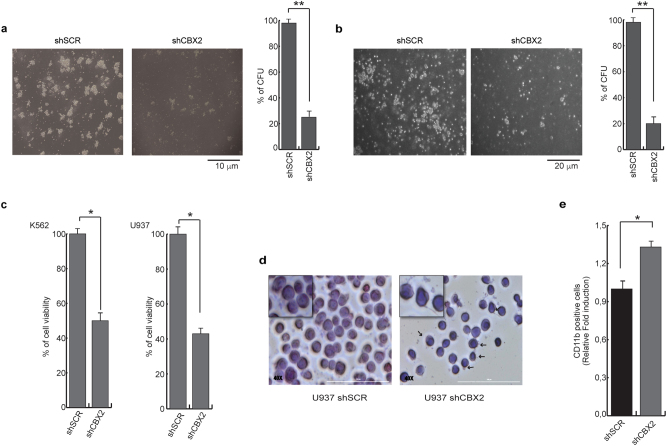
CBX2 depletion impairs tumorigenicity and proliferation of leukemic cells. **a** CFU analysis of transduced shSCR and shCBX2 U937 and (**b**) K562 cells. Error bars represent STD three independent experiments conducted in triplicate (**P < 0.01). **c** MTT assay performed in CBX2-silenced K562 and U937 cells. Error bars represent STD of three independent experiments conducted in triplicate (*P < 0.05). **d** Giemsa assay performed in U937 cells upon CBX2 depletion. Giemsa Digital images acquired in color brightfield microscopy by Cytation 5 Cell Imaging Multi-Mode Reader. Arrows indicate polarized cytoplasm, cytoplasmatic protrusions and filaments. Magnifications ×40. **e** FACS analysis of CD11b expression U937 CBX2-depleted cells. Error bars represent STD three independent experiments conducted in triplicate (*P < 0.05)

The involvement of CBX2 in maintaining hematopoietic stem and progenitor self-renewal was recently described [32]. To investigate the functional role of CBX2 in hematopoietic malignancies, we analyzed the effect of CBX2 depletion in leukemic cells. To mimic SAHA-associated CBX2 downregulation, U937 and K562 cells were transduced with lentivirus expressing non-targeting shRNA (shSCR) and shCBX2. Following CBX2 knockdown, we examined colony-formation unit (CFU) efficiency to evaluate tumorigenic properties and self-renewal (clonogenic) capability of leukemic cells. CBX2 depletion resulted in a marked reduction in CFU efficiency (Fig. 7a, b). Silencing of CBX2 also affected cell viability and proliferation of U937 and K562 cells (Fig. 7c). We also assayed the effect on leukemic cell viability and proliferation of 3 K/R mutant following SAHA treatment by MTT assay. CBX2 and 3K/R mutant were overexpressed in K562 cells and then treated (or left untreated) with SAHA for 24 h. SAHA-mediated impairment of K562 cell viability was significantly reduced by 3K/R mutant, but not by CBX2wt, highlighting the possibility that reduced sensitivity of 3K/R mutant to SAHA-mediated degradation counteracts the impairment of cell proliferation by SAHA (Supplementary Fig. 7).

In addition, we performed Giemsa staining and Immunophenotypic assays to verify whether silencing of CBX2 affects cell differentiation. shCBX2-transduced U937 cells display morphological changes in cell size, more distinct nuclei and nucleoli, and polarized cytoplasm compared to SCR (Fig. 7d). Moreover, cellular margins show cytoplasmic protrusion and filaments, a phenotype reminiscent of monocyte/macrophage differentiated cells. In addition, FACS analysis of CBX2 knocked down cells showed an increase of CD11b differentiation marker (Fig. 7e). These data indicate that CBX2 silencing causes a reduction of leukemic cell tumorigenicity as revealed by cell proliferation impairment and higher leukemic cell differentiation potential.

To investigate the potential interplay of CBX2 downregulation and SAHA-mediated anti proliferative effects, we analyzed microarray gene expression profile data of U937 and K562 SAHA-treated cells [33] to identify common targets in SAHA-treated cells (Supplementary Fig. 8a). Gene Ontology analysis of common deregulated genes between K562 and U937 cells was performed (Supplementary Table 2). Since CBX2 silencing affects leukemic cell proliferation, we focused on genes related to SAHA-altered cell proliferation biological processes. In particular, we selected genes differentially regulated by SAHA (up- and down-regulated in both cell lines; Supplementary Fig. 8b and c) including CDKN1A, a well-known in cis CBX2 target [32] and analyzed by qRT-PCR their expression upon CBX2 depletion. The selected SAHA-deregulated genes were also similarly regulated upon CBX2 silencing (Supplementary Fig. 9).

Collectively, these findings reveal a potential role for CBX2 in sustaining tumorigenic properties of leukemic cells, highlighting its possible oncogenic role in hematological malignancies. Moreover, data suggest that SAHA can also exert its anti-proliferative effects reducing CBX2 protein levels and consequently its transcriptional activity.

## Discussion

Several PcG proteins are deregulated in cancer [15] and their dysfunction is responsible for proliferation, inhibition of apoptosis, and increase in cancer stem cell population [34–36]. The critical interplay of PcG members in determining cellular behavior gives rise to a dynamic regulation of their biological function. However, the molecular mechanisms underlying the finely tuned control of PcG activity are poorly understood. PTMs are an effective way to regulate and possibly modify biological activities and properties. The increasing number of studies demonstrating that several PcG proteins are targets for ubiquitination, SUMOylation and phosphorylation [30, 37, 38] are providing a better understanding of PcG functional regulation by post-translational mechanisms. For example, CBX2 is phosphorylated at Ser42 residue and its phosphorylation changes the binding specificity of CBX2 for methylated H3 [39]. However, no other PTMs regulating CBX2 biological activity have as yet been reported. Identifying novel PcG PTMs could be an important step forward that may help to better understand the regulatory mechanisms of PcG protein activity. Here we provide the first evidence that the pan-HDACi SAHA induces CBX2 SUMOylation and degradation, the latter resulting in cell proliferation arrest and loss of self-renewal in leukemia cell lines.

Our identification of a novel mechanism by which SAHA directly regulates CBX2 stability via a SUMO-triggered ubiquitin-mediated pathway may have important implications. Besides their well-established effects on HDACs, HDACi seem to have a much broader and more complex range of action. The destabilization of CBX2 may contribute to chromatin reset at specific loci. Since these loci are known, the use of targeted strategies might apply to leukemias where chromatin alterations are reported [22]. In agreement, CBX2 is potentially a tumorigenic target in hematological malignancies, since it regulates stemness and self-renewal of leukemic cells.

Given that SAHA targets CBX2 destabilization, its anti-tumorigenic activity might be attributed not only to regulation of the histone and non-histone acetylome, but also to PTM pathway modulation. Interestingly, our findings indicate that SAHA acts specifically on SUMO2/3 and not SUMO1 modification pathway. As SUMO conjugations can be induced by cell stress responses [40], and SAHA is reported to induce cell stress [41], it is tempting to speculate that SAHA regulation of PTM pathways might be linked to cell stress response. However, this hypothesis requires further investigation.

Similarly, as other SUMO2/3 targets such as PML-RARα [5] and NR4A1 [42] have been described, the effects exerted by SAHA on CBX2 might extend to a plethora of targets, likely explaining the broad effects reported on proteome modulation.

Although SUMOylation of other PcG proteins has been described [30, 37, 38], here we clearly demonstrate that CBX2 SUMOylation is responsible for its degradation. Consequently, several intriguing hypotheses might be advanced: i) SUMO-mediated CBX2 degradation may lead to a rearrangement of the canonical PRC1 complex, affecting selective chromatin bindings; ii) PcG proteins may be able to inter-regulate their biological activity (and cell fate) via CBX4 E3 ligase action; iii) since CBX2 is the only human CBX family member able to induce chromatin compaction [43], it may exert a crucial function in PcG-mediated transcriptional repression. Therefore, SAHA-mediated CBX2 degradation suggests that HDACi induce an open chromatin conformation status not only by modulating global acetylation levels, but also by regulating key proteins involved in chromatin compaction.

Targeting ubiquitin ligases such as RNF4 and/or SUMO ligases such as the newly identified CBX4 may represent a novel way through which PcG members can be regulated. This could potentially represent an attractive alternative therapeutic strategy to affect and ‘drug’ chromatin complexes.

## Materials and methods

### Cell culture

U937, K562 and HL-60 cells (DSMZ) were cultured in RPMI 1640 medium (Euroclone, Italy) supplemented with 10% heat-inactivated FBS (Sigma-Aldrich, Italy), 1% glutamine (Euroclone) and 1% penicillin/streptomycin (Euroclone) at 37 °C and 5% CO2. HEK293-FT (Sigma-Aldrich) and HeLa cells (provided by M. Vermeulen) were grown in DMEM (Euroclone) supplemented with 10% FBS, 100 U/mL penicillin/streptomycin (Euroclone) and 6 mM (HEK293-FT) or 2 mM (HeLa) glutamine (Euroclone). They have been tested for mycoplasma contamination.

### AML ex vivo samples

AML blasts cells were recovered from bone marrow, purified and cultured as previously reported [33]. AML blasts were treated with SAHA at 5 μM concentration for 24 h. All experiments were approved by the University of Campania “Luigi Vanvitelli” ethical committee.

### Transfection

HEK293-FT cells were transfected using Lipofectamine 2000 reagent (Thermo Fisher Scientific, Italy) according to manufacturer’ s instructions. After transfection (24 or 48 h), cells were harvested and lysed with the appropriate lysis buffer.

### Antibodies, plasmids and chemicals

Primary antibodies used: anti-CBX2 (Bethyl Laboratories, USA, A302-524A), anti-CBX4 (Abcam, UK, Ab-139815), anti-SUMO1 (add), anti-SUMO-2/3 [8A2] (Abcam, Ab81371), anti-His (Ge Healthcare, Italy, GE27-4710-01), anti-FLAG-M2 (Sigma-Aldrich, F3040), anti-ubiquitin (Abcam, Ab7780), anti-ERK1/2 (Santa Cruz Biotechnology, USA, SC-94), anti-GFP (Abcam, Ab290), anti-RNF4 (11-25) (Sigma-Aldrich, SAB1100321), FITC-Mouse Anti-Human CD11b clone ICRF44 (BD Biosciences, Italy). Antibodies were used according to manufacturer’s instructions.

pcDNA 6xHis/SUMO1-3 were kindly provided by R.T. Hay. YFP/SUMO1-3 were provided by H. de Thé. pcDNA 3xFLAG/RNF4 and pcDNA 3xFLAG/RNF4CS were gifts from J. Palvimo. pcDNA/FLAG CBX4 was kindly provided by Zhou Jing. Human CBX2 coding sequence was cloned into pcDNA FRT-TAP plasmid. pcDNA FRT/CBX2 point single K/R mutants (mt) and 3K/R mt were generated by BIO-FAB Research (Italy).

pLK0.1 shRNA lentiviral vectors were purchased from MISSION shRNA library (Sigma-Aldrich). shRNA vectors included scrambled control (SHC002), shSUMO2/3-655 (TRCN0000007655), shCBX2-183 (TRCN0000364183), shRNF4-821 #1(TRCN0000284821), and shRNF4-894 #2 (TRCN0000281894).

SAHA (Merck, USA), Entinostat (MS275, Alexis Biochemicals, Roma, Italy), UVI5008 (Sigma-Aldrich), GSK-J4 (Sigma-Aldrich), MC1568 (Sigma-Aldrich) and Etoposide (Sigma-Aldrich) were dissolved in DMSO (Sigma-Aldrich) and used at a final concentration of 5 µM. MG132 (Santa Cruz Biotechnology) was dissolved in DMSO and used at final concentration of 25 µg/ml. NEM (Santa Cruz Biotechnology, sc-202719) was used at a final concentration of 10 mM.

CHX (Sigma-Aldrich, C4859) was dissolved in DMSO, used at a final concentration of 50 µg/mL and added 48 h after transfection.

### Lentivirus production and K562 cell transduction

Lentivirus production was performed as previously described [33]. For cell transduction, 50 µL RPMI containing lentiviral vectors was added to 1 × 10^6^ cells. K562 cells were selected in puromycin at a final concentration of 1 μg/mL for 1 week.

### Western blot

Western blot analysis was performed as previously described [44].

Quantification of band intensities was performed by ImageJ software and normalized to loading control.

### SUMOylation and ubiquitination assays

To detect CBX2 SUMOylated forms, cells were lysed in SUMO lysis buffer (62.5 mM Tris-HCL pH 6.8, 2% SDS). Samples were boiled at 95 °C for 10 min and subjected to western blot analysis and immunodetection with the relevant antibodies.

CBX2 SUMOylation and ubiquitination was assayed as previously reported [45].

### Immunoprecipitation and GFP pull-down assay

Immunoprecipitation (IP) assay was performed as previously described [44].

GFP pull-down was performed using GFP-Trap beads (ChromoTek, Germany, gta-20) according to manufacturer’s instructions.

### Generation of inducible HeLa cell line overexpressing GFP-tagged CBX2

HeLa-FRT cells were transiently transfected by lipofectamine, according to supplier’s instructions, with pcDNA5_FRT/CBX2 and pOG44 plasmids. HeLa cells were then selected with 100 µg/mL hygromycin B (Thermo Fisher Scientific) and 3 µg/mL blasticidin S (Sigma-Aldrich). GFP-CBX2 expression was induced by treating cells overnight with 1 µg/mL doxycycline (Sigma-Aldrich).

### Nuclear extraction and GFP pull-down for mass spectrometry

Nuclear extraction was performed as previously reported [46].

GFP pull-down of GFP/CBX2 HeLa and wt HeLa nuclear extracts was performed in triplicate using GFP Nano-Trap beads (Chromotek). For each pull-down, 1 mg of nuclear extract was incubated with 15 µL beads in incubation buffer (300 mM NaCl, 0.15% NP-40, 0.5 mM DDT, 20 mM HEPES-KOH pH 7.9) containing ethidium bromide at a final concentration of 50 mg/ml to prevent indirect DNA-mediated interactions. Beads were then washed twice with incubation buffer containing 0.5% NP-40, twice with PBS containing 0.5% NP-40, and finally twice with PBS alone.

### Sample preparation, mass spectrometry and data analysis

Sample preparation and MS analysis were performed as previously described [47]. For MS data analysis, raw data were analyzed by MaxQuant software (version 1.5.1.0) using standard settings with the additional options match between runs, LFQ and iBAQ. Volcano plots were produced as previously described [47] using Perseus version 1.4.0.8 and in-house R scripts. The mass spectrometry proteomics data have been deposited to the ProteomeXchange Consortium via the PRIDE [1] partner repository with the data set identifier PXD008287”.

### Real-Time qPCR

RNA extraction, cDNA synthesis and gene expression evaluation was performed as previously described [33].

### His-Tag pull-down assay

His-Tag pull-down assay was performed as previously described [45]. His-tagged proteins were eluted in elution buffer and analyzed by Western blot.

### MethoCult assay

Colony formation capability of cells was tested by MethoCult H4535 Enriched without EPO (STEMCELL Technologies, Canada) according to manufacturer’s protocol. Cells (1 × 10^4^) were plated in duplicate and number of colonies was scored after 2 weeks.

### Giemsa staining and MTT assay

Giemsa stain and MTT cell growth assay were purchased by Sigma-Aldrich and used according to manufacture’s instruction.

### Gene expression microarray data analysis

Gene expression data analysis (GSE55154) was performed as previously described [33].

### Statistical analysis

Statistical analysis was performed by two-tailed unpaired *t*-test. *p*-value < 0.05 was considered significant. All experiments were performed in three biological and/or technical replicates.

## Electronic supplementary material

SUPPLEMENTARY FIGURES AND TABLES

Supplementary Table 1 proteinGroups CBX2.pdf

Supplementary Table 2 GO 6h saha k562 u937
