# Supplementary material for: The HDAC inhibitor SAHA regulates CBX2 stability via a SUMO-triggered ubiquitin-mediated pathway in leukemia
Source: Oncogene. 2018 Feb 22;37(19):2559–72. doi: 10.1038/s41388-018-0143-1 (PMC5945585; doi:10.1038/s41388-018-0143-1)
Supplement: Supplementary file 1 — SUPPLEMENTARY FIGURES AND TABLES [file 41388_2018_143_MOESM1_ESM.docx]

**SUPPLEMENTARY FIGURES AND TABLES**

**Supplementary Figure 1**

**a**

**b**

**
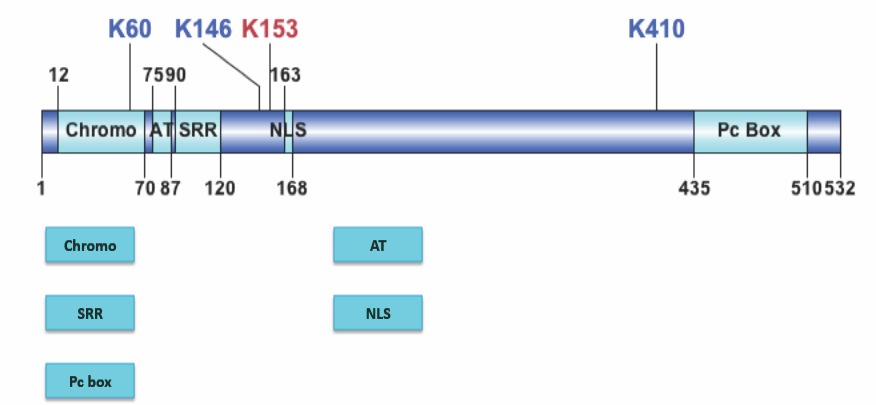
**

**Supplementary Figure 1.** (**a**) Western blot analysis of K562 cell treated with 5 µM of indicated compounds for 24h. Immunoblot was performed with the indicated antibodies. (**b**) Identification of putative SUMO modification sites in the human CBX2 protein sequence using SUMOsp 2.0 software.

**Supplementary Figure 2**

**Supplementary Figure 2**. (**a**) Western blot of endogenous CBX2 in leukemic cells. Slower migrating CBX2 forms are indicated by a bracket (**b**) Western blot of K562 cells transduced with shRNA control and shCBX2 mRNA. Total cell lysate was analyzed for modified CBX2 forms. Modified forms are indicated by a bracket. (**c**) Western blot analysis of unmodified and modified CBX2 forms following SUMO2/3 knockdown in K562 cells.

**Supplementary Figure 3**

**Supplementary Figure 3.** (**a**) Pull-down assay with Ni-NTA-beads of endogenous CBX2 following His/SUMO2 overexpression in HEK293-FT. Western blot of whole protein extracts or His-tagged purified proteins analyzed using anti-CBX2 and anti-His antibodies. (**b**) Real-time qPCR analysis of *CBX2* following overexpression of a fixed amount (2 μg) of His/SUMO2 or SUMO3 plasmids in HEK293-FT.

**Supplementary Figure 4**

**Supplementary Figure 4.** (**a**) Western blot of CBX2 expression following His/SUMO2 overexpression in presence or absence of MG132 in HEK293-FT cells. S-CBX2 is referred to CBX2 SUMOylated forms. (**b**) CBX2 polyubiquitination analysis upon SUMO2 overexpression in presence of 25 µM MG132. IP experiments were performed with anti-CBX2 antibody and immunoblotted with anti-ubiquitin antibody in HEK293-FT cells.

(**c**) Immunoblot analysis of immunoprecipitated cell lysate from K562 shSUMO2/3 knockdown. IP was performed against endogenous CBX2 followed by immunoblotting with anti-ubiquitin antibody.

**Supplementary Figure 5**

**b**

**Supplementary Figure 5.** (**a**) GFP pull-down analysis of wt CBX2 and single K mutant SUMO 2/3-conjugates upon His/SUMO2 overexpression (3μg) in HEK293-FT cells, in presence of 25μM MG132. Western blot of pulled down proteins and input was performed with indicated antibodies. (**b**) Western blot of wt GFP/CBX2 and 3K/R mutant expression in K562 cells treated with SAHA at indicated times. Quantitative densitometry analysis is shown.

**Supplementary Figure 6**

**Supplementary Figure 6.** GFP pull-down analysis of wt CBX2 (3µg) upon FLAG-tagged RNF4 or RNF4-CS overexpression (3µg), treated or not with 5 µM SAHA for 24h in presence of 25µM MG132. Western blot of pulled down proteins and input was performed with indicated antibodies.

**Supplementary Figure 7**

**Supplementary Figure 7.** MTT assay performed in K562 cells overexpressing GFP/CBX2wt or GFP/CBX2 3K/R mutant treated or not with 5 µM SAHA for 24h. Error bars represent STD of three independent experiments conducted in triplicate (**P<0.01; *P<0.05).

**Supplementary Figure 8**

**Supplementary Figure 8.** Comparison analysis of microarray gene expression profile of U937 and K562 cells following 6 h SAHA treatment (5 µM). (**a**) Venn diagram of common altered genes. (**b**) Venn diagram of common positively regulated genes. (**c**) Venn diagram of common negatively regulated genes. FDR ≤ 0.05.

**Supplementary Figure 9**

**Supplementary Figure 9.** qRT-PCR analysis of the indicated genes in U937 CBX2-depleted cells compared to SCR control. Error bars represent STD of three independent experiments conducted in triplicate (**P<0.01).

**Supplementary Table 1. Primer sequences**

| **Primers** | **Forward** | **Reverse** |
| --- | --- | --- |
| ***HPRT1*** | TGAGGATTTGGAAAGGGTGT | CCTCCCATCTCCTTCATCAC |
| ***CBX2*** | CCCACCGACACAAGCAAAAG | TAGCACTGCCCTTGACACAG |
| ***BTG1*** | CAAGTTTCTCCGCACCAAGG | TGCATGGCTTTTCTGGGAAC |
| ***CDC25A*** | ACAACCGATGCAAGCTGTTT | CCAGACATGCTCTTCCTCCT |
| ***GADD45A*** | GGCTGGTAACGAATCCAT | TCCATGTAGCGATTCCCG |
| ***CDKN1A*** | ACTCTCAGGGTCGAAAACGG | ATGTAGAGCGGGCCTTTGAG |
