# Supplementary material for: The HDAC inhibitor SAHA regulates CBX2 stability via a SUMO-triggered ubiquitin-mediated pathway in leukemia
Source: Oncogene. 2018 Feb 22;37(19):2559–72. doi: 10.1038/s41388-018-0143-1 (PMC5945585; doi:10.1038/s41388-018-0143-1)
Supplement: Supplementary file 2 — Supplementary Table 1 proteinGroups CBX2.pdf [file 41388_2018_143_MOESM2_ESM.pdf]

Protein IDs	Majority pro	Peptide coun	Peptide coun	Peptide coun	Protein name	Gene names
F5H6N1;Q96	F5H6N1;Q96	5;5;5;4;2;2;1	5;5;5;4;2;2;1	5;5;5;4;2;2;1	Protein mago	MAGOHB;MA
Q9Y5S9;A0A	Q9Y5S9;A0A	4;4;2;1	4;4;2;1	4;4;2;1	RNA-binding	RBM8A
P98179;A0A	P98179;A0A	2;2	2;2	2;2	Putative RNA	RBM3
B3KNA1;Q9N	B3KNA1;Q9N	10;10;10;10;	10;10;10;10;	10;10;10;10;	Histone deac	DKFZp566E0
A0A087X1I3;	A0A087X1I3;	5;5;5;5;4;4;3	5;5;5;5;4;4;3	5;5;5;5;4;4;3	Succinate de	SDHA
A0A087WVT	A0A087WVT	3;3;3	3;3;3	3;3;3	Zinc finger pr	ZNF48
B3KTZ7;A0A	B3KTZ7;A0A	1;1;1;1	1;1;1;1	1;1;1;1	Probable fibr	FBRS
Q0QEW2;H0	Q0QEW2;H0	6;6;6;6;6;5;5	6;6;6;6;6;5;5	6;6;6;6;6;5;5	60S ribosoma	RPL18
P08621;A8K	P08621;A8K	7;7;7;4;2;1	7;7;7;4;2;1	7;7;7;4;2;1	U1 small nuc	SNRNP70
M0QXN5;P3	M0QXN5;P3	2;2;2	2;2;2	2;2;2	Nuclear pore	NUP62
Q8IV63;A0A	Q8IV63;A0A	4;4;3;3;2;1;1	4;4;3;3;2;1;1	4;4;3;3;2;1;1	Inactive serin	VRK3
A0A024QZJ8	A0A024QZJ8	3;3;3;3;2;2;2	2;2;2;2;1;1;1	2;2;2;2;1;1;1	Cyclin-depen	CDK1;DKFZp
Q53F48;P31	Q53F48;P31	5;5;5;3;2	5;5;5;3;2	5;5;5;3;2	Heterogeneo	HNRNPH3
B4DSN3;G5E	B4DSN3;G5E	2;2;2;2	2;2;2;2	2;2;2;2	Protein trans	SEC24C
O95229;A0A	O95229;A0A	3;3;2;2;2	3;3;2;2;2	3;3;2;2;2	ZW10 intera	ZWINT
B4DKM5;A0A	B4DKM5;A0A	1;1;1;1;1	1;1;1;1;1	1;1;1;1;1	Voltage-depe	VDAC2
Q9BRX2;B3K	Q9BRX2;B3K	11;11;11	11;11;11	11;11;11	Protein pelot	PELO
H7C2B1;Q9U	H7C2B1;Q9U	1;1;1	1;1;1	1;1;1	Nucleolar pro	NOL7
P49790;A0A	P49790;A0A	25;25	25;25	25;25	Nuclear pore	NUP153
Q13523;A0A	Q13523;A0A	7;7;6;4;2	7;7;6;4;2	7;7;6;4;2	Serine/threo	PRPF4B
Q99880;Q99	Q99880;Q99	6;6;6;6;6;6;6	6;6;6;6;6;6;6	6;6;6;6;6;6;6	Histone H2B	HIST1H2BL;H
Q93077;Q7L	Q93077;Q7L	4;4;4;4;4	1;1;1;1;1	1;1;1;1;1	Histone H2A	HIST1H2AC;H
E9PF10;B4DL	E9PF10;B4DL	18;18;18;18;	18;18;18;18;	18;18;18;18;	Nuclear pore	NUP155
A0A024R0A9	A0A024R0A9	2;2;2;1;1	2;2;2;1;1	2;2;2;1;1	45 kDa calciu	SDF4
B3KQ42;A0A	B3KQ42;A0A	9;9;9;3;3;1;1	9;9;9;3;3;1;1	9;9;9;3;3;1;1	Zinc finger pr	ZNF131
C9JUG7;F8W	C9JUG7;F8W	1;1;1;1;1;1;1	1;1;1;1;1;1;1	1;1;1;1;1;1;1	F-actin-cappi	CAPZA2;CAP
Q8N7H5;A0A	Q8N7H5;A0A	8;8;3	8;8;3	8;8;3	RNA polymer	PAF1
B4DJP0;Q9B	B4DJP0;Q9B	1;1;1	1;1;1	1;1;1	Methylosom	WDR77
A8K3W4;A0A	A8K3W4;A0A	6;6;6;6;6;6;5	6;6;6;6;6;6;5	6;6;6;6;6;6;5	Heterogeneo	HNRNPUL1
B7Z6N1;P50	B7Z6N1;P50	8;8;8	8;8;8	8;8;8	ETS domain-	ERF
Q3ZCQ8;Q0V	Q3ZCQ8;Q0V	9;9;9;6;5;4;3	9;9;9;6;5;4;3	9;9;9;6;5;4;3	Mitochondria	TIMM50
Q14588;A0A	Q14588;A0A	2;2	1;1	1;1	Zinc finger pr	ZNF234
Q9NZL3;A8K	Q9NZL3;A8K	2;2;2;1;1;1;1	1;1;1;0;0;0;0	1;1;1;0;0;0;0	Zinc finger pr	ZNF224;ZNF
Q8IV08;A0A	Q8IV08;A0A	2;2;1;1;1	2;2;1;1;1	2;2;1;1;1	Phospholipas	PLD3
B3KY11;Q9B	B3KY11;Q9B	3;3;3;3	3;3;3;3	3;3;3;3	Probable ATP	DDX23
F8VZ70;F8VL	F8VZ70;F8VL	1;1;1;1;1;1;1	1;1;1;1;1;1;1	1;1;1;1;1;1;1	SWI/SNF-rela	SMARCD1
Q12800;A0A	Q12800;A0A	5;5;4;4;1;1;1	5;5;4;4;1;1;1	4;4;4;3;0;1;1	Alpha-globin	TFCP2
Q8N3V2;P22	Q8N3V2;P22	2;2;2;2;2;1;1	2;2;2;2;2;1;1	2;2;2;2;2;1;1	Nuclear rece	DKFZp451G1
H0YID1;F8VL	H0YID1;F8VL	1;1;1;1;1;1;1	1;1;1;1;1;1;1	1;1;1;1;1;1;1	KAT8 regulat	KANSL2
Q9H0H5;B2R	Q9H0H5;B2R	5;5;5;3;2;2;1	5;5;5;3;2;2;1	5;5;5;3;2;2;1	Rac GTPase-a	RACGAP1
Q9Y2D5;A0A	Q9Y2D5;A0A	1;1;1;1	1;1;1;1	1;1;1;1	A-kinase anc	AKAP2
O95347;B7Z	O95347;B7Z	16;16;16;16;	16;16;16;16;	16;16;16;16;	Structural ma	SMC2
B2R923;A0A	B2R923;A0A	2;2;2	2;2;2	2;2;2	Hydroxyster	HSDL2
O95758;A0A	O95758;A0A	7;7;4	5;5;3	5;5;3	Polypyrimidi	PTBP3

Q9NXF1;A0A	Q9NXF1;A0A	7;7;4;3;1	7;7;4;3;1	7;7;4;3;1	Testis-expres	TEX10
Q09161;A0A(	Q09161;A0A(	2;2;1	2;2;1	2;2;1	Nuclear cap-l	NCBP1
Q13356;A8K(	Q13356;A8K(	3;3;3;2	3;3;3;2	3;3;3;2	Peptidyl-prol	PPIL2
A0A087WW\	A0A087WW\	2;2;2;1;1;1;1;	2;2;2;1;1;1;1;	2;2;2;1;1;1;1;	Calcineurin-b	CABIN1
Q9HD72;A8K	Q9HD72;A8K	9;9;9;9;9;9;9;	8;8;8;8;8;8;8;	8;8;8;8;8;8;8;	POZ-, AT hoo	PATZ1
A0A024R1J3;	A0A024R1J3;	2;2	2;2	2;2	Cdc42 effect(	CDC42EP1
Q04917;A0A(	Q04917;A0A(	3;3;2;2;2;2;2;	1;1;0;0;0;0;0;	1;1;0;0;0;0;0;	14-3-3 protei	YWHAH;YWH
Q15459;A0A(	Q15459;A0A(	3;3;1	3;3;1	3;3;1	Splicing facto	SF3A1
B4DE32;B1AI	B4DE32;B1AI	6;6;6;6;6;3;2	6;6;6;6;6;3;2	6;6;6;6;6;3;2	X-ray repair (	XRCC6
Q9UH99;A0A	Q9UH99;A0A	4;4;4;3;3;3;2;	4;4;4;3;3;3;2;	4;4;4;3;3;3;2;	SUN domain-	SUN2;UNC84
Q9BTQ7;P62	Q9BTQ7;P62	7;7;7;5;4;4;3;	7;7;7;5;4;4;3;	7;7;7;5;4;4;3;	60S ribosoma	RPL23
Q99741;B2R!	Q99741;B2R!	2;2;2	2;2;2	2;2;2	Cell division (	CDC6
J3QKS7;B4D(	J3QKS7;B4D(	7;7;7;7;4;3;3;	7;7;7;7;4;3;3;	7;7;7;7;4;3;3;	SWI/SNF-rela	SMARCE1
F8WFE7;Q86	F8WFE7;Q86	1;1;1	1;1;1	1;1;1	Active regula	RPS19BP1
CON__Q927(	CON__Q927(	15;15;15;15;:	10;10;10;10;(	3;3;3;3;0	Keratin, type	KRT35
A0A024R1Y2	A0A024R1Y2	3;3;3;3;2;1	3;3;3;3;2;1	3;3;3;3;2;1	ATP-citrate s	ACLY;ACLY va
P46060;A0A(	P46060;A0A(	9;9;6;6;4;2	9;9;6;6;4;2	9;9;6;6;4;2	Ran GTPase-a	RANGAP1
Q7RTV0;A0A	Q7RTV0;A0A	4;4	4;4	4;4	PHD finger-lil	PHF5A
E9PC22;E7EN	E9PC22;E7EN	1;1;1;1;1;1;1;	1;1;1;1;1;1;1;	1;1;1;1;1;1;1;	Breast cance	BRCA1
E4W6B6;P61	E4W6B6;P61	8;8;8;8;7;5;2;	8;8;8;8;7;5;2;	8;8;8;8;7;5;2;	60S ribosoma	RPL27
P35227;A0A(	P35227;A0A(	16;16;10;8	16;16;10;8	13;13;8;6	Polycomb gr(	PCGF2
P14923;A0A(	P14923;A0A(	9;9	5;5	4;4	Junction plak	JUP
B3KVB0;K7ES	B3KVB0;K7ES	1;1;1;1	1;1;1;1	1;1;1;1	GH3 domain-	GHDC
J7FRP4;A0A0	J7FRP4;A0A0	1;1;1;1;1;1;1;	1;1;1;1;1;1;1;	1;1;1;1;1;1;1;	Proteasome a	PSME3
K7EIY6;A0A0	K7EIY6;A0A0	4;4;4;3;3;3;1;	4;4;4;3;3;3;1;	4;4;4;3;3;3;1;	E3 ubiquitin-|	RNF126
P61978;A0A(	P61978;A0A(	24;24;24;23;:	24;24;24;23;:	24;24;24;23;:	Heterogeneo	HNRNPK;HNI
Q16649;D9IV	Q16649;D9IV	2;2;2	2;2;2	2;2;2	Nuclear facto	NFIL3
Q76FK4;A0A(	Q76FK4;A0A(	10;10;9;9;9;8	10;10;9;9;9;8	10;10;9;9;9;8	Nucleolar pro	NOL8;DKFZp(
A0A087WXN	A0A087WXN	5;5;5;5;5;5;5;	5;5;5;5;5;5;5;	5;5;5;5;5;5;5;	60S ribosoma	RPL17
K7EL15;K7EL	K7EL15;K7EL	1;1;1;1;1;1;1;	1;1;1;1;1;1;1;	1;1;1;1;1;1;1;	Mothers agai	SMAD4
O15269;A0A(	O15269;A0A(	2;2;1	2;2;1	2;2;1	Serine palmit	SPTLC1
A0A024R2D7	A0A024R2D7	2;2;2;1	2;2;2;1	2;2;2;1	Transcription	TADA3
Q9BXW9;A0/	Q9BXW9;A0/	9;9;5;1	9;9;5;1	9;9;5;1	Fanconi aner	FANCD2
D3YTB1;P62S	D3YTB1;P62S	6;6;6;6;1	6;6;6;6;1	6;6;6;6;1	60S ribosoma	RPL32
Q53H10;A0A	Q53H10;A0A	5;5;4;2	5;5;4;2	5;5;4;2	E3 ubiquitin-|	RAD18
C9JAW5;Q9Y	C9JAW5;Q9Y	1;1;1;1;1	1;1;1;1;1	1;1;1;1;1	HIG1 domain	HIGD1A
C9J9K3;P088	C9J9K3;P088	6;6;6;6;5;4;3;	6;6;6;6;5;4;3;	6;6;6;6;5;4;3;	40S ribosoma	RPSA;LAMR1
Q15024;B2RI	Q15024;B2RI	5;5;5;3	5;5;5;3	5;5;5;3	Exosome con	EXOSC7
B4DSW9;B4D	B4DSW9;B4D	3;3;3;3;3;3;1	2;2;2;2;2;2;0	2;2;2;2;2;2;0	Catenin beta	CTNNB1
P61313;A0A(	P61313;A0A(	8;8;6;6;5;4;3;	8;8;6;6;5;4;3;	8;8;6;6;5;4;3;	60S ribosoma	RPL15
A0A0D9SEH2	A0A0D9SEH2	2;2;2;2	2;2;2;2	2;2;2;2	ATR-interacti	ATRIP
P52756;A0A(	P52756;A0A(	5;5;4;1;1;1;1;	5;5;4;1;1;1;1;	5;5;4;1;1;1;1;	RNA-binding	RBM5
A0A024R2Z6	A0A024R2Z6	6;5	6;5	6;5	Guanine nucl	GNL3
F8W6N3;Q92	F8W6N3;Q92	2;2;2;1	2;2;2;1	2;2;2;1	Ubiquitin car	BAP1
A0A024R326	A0A024R326	2;2;2	2;2;2	2;2;2	60S ribosoma	RPL29

Q9BS79;F5G)	Q9BS79;F5G)	2;2;2;2;2;2;1,	2;2;2;2;2;2;1;	2;2;2;2;2;2;1,	Double-stran	MRE11A
Q9BTE7;A0A(	Q9BTE7;A0A(	4;4;3;2;2;2;1,	4;4;3;2;2;2;1;	4;4;3;2;2;2;1,	DCN1-like pr(	DCUN1D5
Q9BRL6;A0A(	Q9BRL6;A0A(	4;4	2;2	2;2	Serine/argini	SRSF8
A0A024R3B4	A0A024R3B4	2;2;2	2;2;2	2;2;2	Spliceosome-	CWC15
Q13315;A0A(	Q13315;A0A(	8;8;5;4;2;2;2,	8;8;5;4;2;2;2;	8;8;5;4;2;2;2,	Serine-protei	ATM;DKFZp7
Q9NVF7;A0A	Q9NVF7;A0A	1;1	1;1	1;1	F-box only pr	FBXO28
P09874;A0A(	P09874;A0A(	21;21;20;20;(	21;21;20;20;(	21;21;20;20;(	Poly [ADP-rib	PARP1
Q8WWC4;A0	Q8WWC4;A0	1;1	1;1	1;1	Uncharacteri	C2orf47
A0A0A0MSG	A0A0A0MSG	4;4;4;4	4;4;4;4	4;4;4;4	SPATS2-like p	SPATS2L
Q9H2H8;A0A	Q9H2H8;A0A	5;5;5;4;3;2	5;5;5;4;3;2	5;5;5;4;3;2	Peptidyl-prol	PPIL3
P24534;A0A(	P24534;A0A(	2;2;1;1;1;1	2;2;1;1;1;1	2;2;1;1;1;1	Elongation fa	EEF1B2;LOC3
P10809;A0A(	P10809;A0A(	13;13;12;12;:	13;13;12;12;:	13;13;12;12;:	60 kDa heat s	HSPD1
B8ZZJ0;B9A0	B8ZZJ0;B9A0	2;2;2;2;2;1	2;2;2;2;2;1	2;2;2;2;2;1	Small ubiquit	SUMO1
E9PHS0;Q6FI	E9PHS0;Q6FI	5;5;5;5;5;5;2,	5;5;5;5;5;5;2;	5;5;5;5;5;5;2,	LanC-like pro	LANCL1
Q13416;A0A(	Q13416;A0A(	4;4;3;1;1	4;4;3;1;1	4;4;3;1;1	Origin recogr	ORC2
Q5R210;Q5R	Q5R210;Q5R	28;28;28;28;:	28;28;28;28;:	28;28;28;28;:	Carbamoyl-p	CPS1
B3KN05;O95	B3KN05;O95	2;2;2;2;1;1	2;2;2;2;1;1	2;2;2;2;1;1	Long-chain-fa	ACSL3
P19338;A0A(	P19338;A0A(	20;20;19;18;:	20;20;19;18;:	20;20;19;18;:	Nucleolin	NCL
A0A0A0N0L3	A0A0A0N0L3	7;7;7;6;6;6;6,	7;7;7;6;6;6;6;	7;7;7;6;6;6;6,	TAR DNA-bin	TARDBP
K7EM90;P06	K7EM90;P06	2;2;2;1;1;1;1,	2;2;2;1;1;1;1;	2;2;2;1;1;1;1,	Enolase;Alph	ENO1;ENO2;I
Q0QF37;Q75	Q0QF37;Q75	5;5;5;5;5;4;3	5;5;5;5;5;4;3	5;5;5;5;5;4;3	Malate dehy(	MDH2
P46781;A0A(	P46781;A0A(	6;6;4;3;3;3;1,	6;6;4;3;3;3;1;	6;6;4;3;3;3;1,	40S ribosoma	RPS9
M0R0F0;Q53	M0R0F0;Q53	8;8;8;8;7;4;2	8;8;8;8;7;4;2	8;8;8;8;7;4;2	40S ribosoma	RPS5
P61081;A0A(	P61081;A0A(	2;2;1	2;2;1	2;2;1	NEDD8-conju	UBE2M
A0A024R4V8	A0A024R4V8	4;4;3	4;4;3	4;4;3	G2 and S pha	GTSE1
Q9UKX7;A0A	Q9UKX7;A0A	2;2	2;2	2;2	Nuclear pore	NUP50
A0A0A6YYC7	A0A0A6YYC7	2;2;2;2;1;1	2;2;2;2;1;1	2;2;2;2;1;1	E3 ubiquitin-|	ZFP91;ZFP91-
Q08945;A0A(	Q08945;A0A(	12;12;1;1;1	12;12;1;1;1	12;12;1;1;1	FACT comple	SSRP1
O94776;A0A(	O94776;A0A(	16;16	16;16	13;13	Metastasis-a:	MTA2
A0A024R566	A0A024R566	15;14;14;14;:	15;14;14;14;:	12;12;12;12;:	Splicing facto	SF1
Q9UE24;E7EI	Q9UE24;E7EI	3;3;3;3;3;3;1	3;3;3;3;3;3;1	3;3;3;3;3;3;1	Menin	MEN1
Q92785;A0A(	Q92785;A0A(	3;3;3;1	3;3;3;1	3;3;3;1	Zinc finger pr	DPF2
Q9Y6J9;A8K(	Q9Y6J9;A8K(	2;2;2;1;1	2;2;2;1;1	2;2;2;1;1	TAF6-like RN.	TAF6L
Q9H880;Q7L	Q9H880;Q7L	1;1;1;1;1;1;1,	1;1;1;1;1;1;1;	1;1;1;1;1;1;1,	Serine/threo	SAPS3;PPP6R
P50416;B2R/	P50416;B2R/	2;2;2	2;2;2	2;2;2	Carnitine O-p	CPT1A
X5DNI9;Q9UI	X5DNI9;Q9UI	2;2;2;2;1;1;1,	2;2;2;2;1;1;1;	2;2;2;2;1;1;1,	7-dehydroch(	DHCR7
C9JB90;C9JU	C9JB90;C9JU	1;1;1;1;1;1;1,	1;1;1;1;1;1;1;	1;1;1;1;1;1;1,	Ras-related p	RAB6B;RAB6/
E9PQ34;E9PJ	E9PQ34;E9PJ	1;1;1;1;1;1;1,	1;1;1;1;1;1;1;	1;1;1;1;1;1;1,	Serpin H1	SERPINH1;ra-
A0A024R5M!	A0A024R5M!	134;130;104;	134;130;104;	2;2;2;2;2;2;2		
A0A024R5R8	A0A024R5R8	2;2	2;2	2;2	Dimethylade	TFB2M
B4DGI9;B4DZ	B4DGI9;B4DZ	4;4;4;4;4;3;3,	4;4;4;4;4;3;3;	4;4;4;4;4;3;3,	Transcription	TCF12
H3BMQ2;Q9I	H3BMQ2;Q9I	1;1;1;1	1;1;1;1	1;1;1;1	Probable ribo	RSL24D1
H0YL80;D6R!	H0YL80;D6R!	1;1;1;1;1;1;1,	1;1;1;1;1;1;1;	1;1;1;1;1;1;1,	Tropomyosin	TPM1;TPM3;
V9HW65;P07	V9HW65;P07	19;19;19;18;:	19;19;19;18;:	19;19;19;18;:	Annexin A2;A	ANXA2;ANXA
A0A024R609	A0A024R609	2;2;2;2;2;2;2,	2;2;2;2;2;2;2;	2;2;2;2;2;2;2,	Pyruvate kina	PKM;PKM2;P

Q7Z612;Q6I(	Q7Z612;Q6I(	1;1;1;1;1	1;1;1;1;1	1;1;1;1;1	60S acidic rib	RPLP1
Q6PJG2;A0A(	Q6PJG2;A0A(	14;14;13;13;	14;14;13;13;	14;14;13;13;	ELM2 and SA	ELMSAN1;C1
P84090;A0A(	P84090;A0A(	6;6;5	6;6;5	6;6;5	Enhancer of	ERH
Q13243;A0A(	Q13243;A0A(	4;4;3;2	3;3;2;2	3;3;2;2	Serine/argini	SRSF5;SFRS5
B3KMI0;B3KI	B3KMI0;B3KI	2;2;2;2;1	2;2;2;2;1	2;2;2;2;1	Cleavage and	CPSF2
B4DRJ7;Q5EE	B4DRJ7;Q5EE	1;1;1;1;1;1;1	1;1;1;1;1;1;1	1;1;1;1;1;1;1	PAP-associat	PAPD5
A0A024R6Q8	A0A024R6Q8	2;2	2;2	2;2	Bromodomai	BRD7
O60884;A0A(	O60884;A0A(	8;8;4;4;3;1	8;8;4;4;3;1	8;8;4;4;3;1	DnaJ homolo	DNAJA2
Q8WVB5;Q8	Q8WVB5;Q8	1;1;1;1;1;1;1;	1;1;1;1;1;1;1;	1;1;1;1;1;1;1;	Metallothion	MT1X;MT1M
Q5HYH5;O43	Q5HYH5;O43	2;2;2;2	2;2;2;2	2;2;2;2	Cleavage and	DKFZp313O2
Q13951;A0A(	Q13951;A0A(	4;4;3;3;2;1;1	4;4;3;3;2;1;1	4;4;3;3;2;1;1	Core-binding	CBFB
A0A024R734	A0A024R734	3;3;2	3;3;2	3;3;2	Nuclear facto	NFAT5
Q9NW13;A0/	Q9NW13;A0/	4;4;2;1;1;1	4;4;2;1;1;1	4;4;2;1;1;1	RNA-binding	RBM28
Q6IAW5;O43	Q6IAW5;O43	3;3;3;3;3;2;1	3;3;3;3;3;2;1	3;3;3;3;3;2;1	Calumenin	CALU
A0A024R7C4	A0A024R7C4	3;3;3;1;1;1;1	3;3;3;1;1;1;1	3;3;3;1;1;1;1	Protein KRI1	KRI1
K7EKI4;K7ELF	K7EKI4;K7ELF	1;1;1;1;1;1;1	1;1;1;1;1;1;1	1;1;1;1;1;1;1	39S ribosoma	MRPL4
Q12906;A0A(	Q12906;A0A(	18;18;16;16;	18;18;16;16;	2;2;2;2;2;0;2;	Interleukin e	ILF3
Q96RE7;A0A(	Q96RE7;A0A(	2;2;1;1	2;2;1;1	2;2;1;1	Nucleus accu	NACC1;NACC
P26358;A0A(	P26358;A0A(	28;28;25;4;3;	28;28;25;4;3;	4;4;2;4;3;2;1;	DNA (cytosin	DNMT1
Q8IX01;A8K5	Q8IX01;A8K5	26;26;26;26;	26;26;26;26;	26;26;26;26;	SURP and G-	SUGP2
B4DKZ7;Q86	B4DKZ7;Q86	3;3;3;3;2	3;3;3;3;2	3;3;3;3;2	Transcription	GATAD2A
A0A024R7N3	A0A024R7N3	1;1;1;1	1;1;1;1	1;1;1;1	SURP and G-	SUGP1;SF4
Q96RL1;A0A(	Q96RL1;A0A(	6;6;4;3;2;2;1;	6;6;4;3;2;2;1;	6;6;4;3;2;2;1;	BRCA1-A con	UIMC1;DKFZ
P52597;A0A(	P52597;A0A(	13;13;11	11;11;9	11;11;9	Heterogeneo	HNRNPF
P17026;A0A(	P17026;A0A(	2;2	1;1	1;1	Zinc finger pr	ZNF22
B4DLA6;P33	B4DLA6;P33	4;4;4;4;1;1;1	4;4;4;4;1;1;1	4;4;4;4;1;1;1	DNA replicati	MCM4
Q9P2D1;A0A	Q9P2D1;A0A	26;26;10	17;17;8	15;15;8	Chromodoma	CHD7
R4GMY8;E5R	R4GMY8;E5R	2;2;2;2	2;2;2;2	2;2;2;2	Transcription	TCEB1
P18124;A0A(	P18124;A0A(	9;9;8;3;2	9;9;8;3;2	5;5;4;3;2	60S ribosoma	RPL7
Q8NHZ8;A0A	Q8NHZ8;A0A	1;1	1;1	1;1	Anaphase-pr	CDC26
A0A024R856	A0A024R856	3;3;3;1	3;3;3;1	3;3;3;1	Trinucleotide	TNRC18
O00743;A0A(	O00743;A0A(	2;2;1	2;2;1	2;2;1	Serine/threo	PPP6C
F2Z388;P427	F2Z388;P427	2;2;2;1	2;2;2;1	2;2;2;1	60S ribosoma	RPL35;LOC15
H0Y5D5;Q9B	H0Y5D5;Q9B	6;6;6;6;6;6;6;	6;6;6;6;6;6;6;	6;6;6;6;6;6;6;	Cip1-interact	CIZ1
A0A024R889	A0A024R889	3;3;3;3;2;1;1;	3;3;3;3;2;1;1;	3;3;3;3;2;1;1;	Spectrin alph	SPTAN1;DKFZ
A0A0C4DFV9	A0A0C4DFV9	2;2;2;2;2;2;2	2;2;2;2;2;2;2	2;2;2;2;2;2;2	Protein SET	SET
Q5TBH0;Q5T	Q5TBH0;Q5T	1;1;1;1;1;1;1;	1;1;1;1;1;1;1;	1;1;1;1;1;1;1;	Serine/threo	NEK6;NEK7
Q96I24;A0A(	Q96I24;A0A(	11;11	10;10	9;9	Far upstream	FUBP3
Q7Z3C4;A0A(	Q7Z3C4;A0A(	8;8;8;8;3;2;2;	8;8;8;8;3;2;2;	8;8;8;8;3;2;2;	Nuclear pore	DKFZp686J03
Q15361;B4D	Q15361;B4D	3;3;3;1	3;3;3;1	3;3;3;1	Transcription	TTF1
Q9BV86;A0A	Q9BV86;A0A	2;2;1;1	2;2;1;1	2;2;1;1	N-terminal X	NTMT1
P04183;B5BU	P04183;B5BU	2;2;2;2;2;1;1	2;2;2;2;2;1;1	2;2;2;2;2;1;1	Thymidine ki	TK1
J3KT73;J3QL(	J3KT73;J3QL(	4;4;4;4;2	4;4;4;4;2	4;4;4;4;2	60S ribosoma	RPL38
P61956;A0A(	P61956;A0A(	3;3;1;1;1;1	3;3;1;1;1;1	3;3;1;1;1;1	Small ubiquit	SUMO2
B4DNL5;P07	B4DNL5;P07	13;13;13;12;	13;13;13;12;	13;13;13;12;	Protein disul	P4HB

J3KP15;Q53F	J3KP15;Q53F	9;9;9;9;8;7;7;	9;9;9;9;8;7;7;	7;7;7;7;6;5;5;	Serine/argini	SRSF2;SFRS2
Q9P275;A0A(	Q9P275;A0A(	14;14;12;7;7;	14;14;12;7;7;	14;14;12;7;7;	Ubiquitin car	USP36;DUB1
P38919;A0A(	P38919;A0A(	7;7;4;1;1;1;1;	7;7;4;1;1;1;1;	6;6;3;0;0;0;0;	Eukaryotic in	EIF4A3
A0A0C4DG88	A0A0C4DG88	2;2;2;2	2;2;2;2	2;2;2;2	POU domain,	POU2F1
A0A087WYY(	A0A087WYY(	3;3;3;2;1	3;3;3;2;1	3;3;3;2;1	Plakophilin-1	PKP1
Q15291;A0A(	Q15291;A0A(	5;5;5;4	5;5;5;4	5;5;5;4	Retinoblasto	RBBP5
A0A024R9E2	A0A024R9E2	11;11;11;11;1	11;11;11;11;1	11;11;11;11;1	Polyadenylat	PABPC1
P62888;A0A(	P62888;A0A(	10;10;9;4;4;3	10;10;9;4;4;3	10;10;9;4;4;3	60S riboson	RPL30
E5RHX8;Q9N	E5RHX8;Q9N	2;2;2;1;1;1;1	2;2;2;1;1;1;1	2;2;2;1;1;1;1	Enhancer of y	ENY2
Q6PL18;A0A(	Q6PL18;A0A(	6;6;4	6;6;4	6;6;4	ATPase famil	ATAD2
O60216;A0A(	O60216;A0A(	8;8;3;2;2;2;2;	8;8;3;2;2;2;2;	8;8;3;2;2;2;2;	Double-stran	RAD21
Q96QD9;A0A	Q96QD9;A0A	2;2;1	2;2;1	2;2;1	UAP56-intera	FYTTD1
B4E1N7;P011	B4E1N7;P011	5;5;5;4;4;2;2;	5;5;5;4;4;2;2;	5;5;5;4;4;2;2;	Myc proto-or	MYC
H0YLY7;H0YK	H0YLY7;H0YK	1;1;1;1;1;1;1	1;1;1;1;1;1;1	1;1;1;1;1;1;1	Calcineurin B	CHP1
Q9H2Y7;A0A	Q9H2Y7;A0A	17;17;9;8;8;7	17;17;9;8;8;7	17;17;9;8;8;7	Zinc finger pr	ZNF106
D6RAA6;P57(	D6RAA6;P57(	1;1;1;1	1;1;1;1	1;1;1;1	Transmembr	TMEM33;SHI
Q05DU1;Q9N	Q05DU1;Q9N	10;10;10	10;10;10	10;10;10	Guanine nucl	GNL3L
O00483;A0A(	O00483;A0A(	2;2	2;2	2;2	NADH dehyd	NDUFA4
P35869;A0A(	P35869;A0A(	2;2	2;2	2;2	Aryl hydroca	AHR
A8K5D9;Q9N	A8K5D9;Q9N	25;25;25;22;4	25;25;25;22;4	25;25;25;22;4	Actin-binding	ANLN
Q96GN5;A8K	Q96GN5;A8K	10;10;10;2;1;	10;10;10;2;1;	10;10;10;2;1;	Cell division c	CDCA7L
P22626;A0A(	P22626;A0A(	20;20;19;16;1	20;20;19;16;1	20;20;19;16;1	Heterogeneo	HNRNPA2B1
Q86V48;A0A	Q86V48;A0A	2;2	2;2	2;2	Leucine zippe	LUZP1
Q9P258;A0A(	Q9P258;A0A(	27;27;26	27;27;26	27;27;26	Protein RCC2	RCC2
A0A0C4DGS1	A0A0C4DGS1	2;2;2;1	2;2;2;1	2;2;2;1	Dolichyl-diph	DDOST
Q9NVP1;A0A	Q9NVP1;A0A	10;10;9;6;5;4	10;10;9;6;5;4	10;10;9;6;5;4	ATP-depende	DDX18
B7Z8S8;E7EC	B7Z8S8;E7EC	2;2;2;2;2;2;2;	2;2;2;2;2;2;2;	2;2;2;2;2;2;2;	Calpastatin	CAST
Q99878;Q96I	Q99878;Q96I	5;5;5;5;5;5;5;	5;5;5;5;5;5;5;	2;2;2;2;2;2;2;	Histone H2A	HIST1H2AJ;H
Q9H0S4;A0A	Q9H0S4;A0A	11;11;10;10;(	11;11;10;10;(	11;11;10;10;(	Probable ATF	DDX47;E4-DE
F8VZY9;P057	F8VZY9;P057	12;12;12;12;1	11;11;11;11;9	10;10;10;10;8	Keratin, type	KRT18
F8W6I7;A0A(	F8W6I7;A0A(	15;15;15;15;1	15;15;15;15;1	15;15;15;15;1	Heterogeneo	HNRNPA1;HN
E7ESI2;A0A0	E7ESI2;A0A0	6;6;6;6;6;5;2;	6;6;6;6;6;5;2;	5;5;5;5;5;4;1;	Cyclin-depen	CDK2
P62854;A0A(	P62854;A0A(	3;3;2;2;1;1	3;3;2;2;1;1	3;3;2;2;1;1	40S riboson	RPS26;RPS26
Q59G16;Q8T	Q59G16;Q8T	16;16;16;16;1	16;16;16;16;1	8;8;8;8;7;1;1	SWI/SNF con	SMARCC2
Q15185;A0A(	Q15185;A0A(	1;1;1;1	1;1;1;1	1;1;1;1	Prostaglandir	PTGES3
F8W1N5;F8V	F8W1N5;F8V	1;1;1;1;1;1;1;	1;1;1;1;1;1;1;	1;1;1;1;1;1;1;	Nascent poly	NACA
Q9UL25;A0A(	Q9UL25;A0A(	2;2;1	2;2;1	2;2;1	Ras-related p	RAB21
Q16527;A0A(	Q16527;A0A(	12;12;11;11	12;12;11;11	12;12;11;11	Cysteine and	CSRP2
Q9Y2U8;A0A	Q9Y2U8;A0A	4;4;2	4;4;2	4;4;2	Inner nuclear	LEMD3
P42167;A0A(	P42167;A0A(	14;14;13;11;1	5;5;4;2;2;3;1	5;5;4;2;2;3;1	Lamina-assoc	TMPO
Q8NCF7;B2R	Q8NCF7;B2R	9;9;9;8;8;8;8;	9;9;9;8;8;8;8;	9;9;9;8;8;8;8;	Phosphate ca	SLC25A3
H0YHI8;A0A(	H0YHI8;A0A(	1;1;1;1	1;1;1;1	1;1;1;1	Protein phos	PPP1R12A
Q9ULV4;Q53	Q9ULV4;Q53	7;7;7;7;7;6;5;	7;7;7;7;7;6;5;	7;7;7;7;7;6;5;	Coronin-1C;C	CORO1C
B3KMP2;Q2N	B3KMP2;Q2N	1;1;1;1;1;1;1	1;1;1;1;1;1;1	1;1;1;1;1;1;1	Anaphase-pr	ANAPC7
Q8TBK5;Q8N	Q8TBK5;Q8N	12;12;12;12;1	12;12;12;12;1	12;12;12;12;1	60S riboson	RPL6

Q9UPN1;F8V	Q9UPN1;F8V	6;6;6;6;5;5;2	6;6;6;6;5;5;2	2;2;2;2;2;1;2	Serine/threo	PPP1CC
F8VVL1;O435	F8VVL1;O435	3;3;3	3;3;3	3;3;3	Density-regu	DENR
Q8N3J9;A0A(	Q8N3J9;A0A(	3;3;2	2;2;1	2;2;1	Zinc finger pr	ZNF664
Q53HW2;P05	Q53HW2;P05	5;5;5;5;4;4;4	5;5;5;5;4;4;4	5;5;5;5;4;4;4	60S acidic rib	RPLP0;RPLP0
C9J7T7;C9JES	C9J7T7;C9JES	10;10;10;10;1	10;10;10;10;1	10;10;10;10;1	Nuclear rece	NCOR2
Q53HK3;P41(	Q53HK3;P41(	6;6;6;6;5;4;4	6;6;6;6;5;4;4	6;6;6;6;5;4;4	Eukaryotic tr	EIF2S3;EIF2S3
P53701;A0A(	P53701;A0A(	4;4;3	4;4;3	4;4;3	Cytochrome	HCCS;DKFZp7
Q15555;A0A(	Q15555;A0A(	1;1	1;1	1;1	Microtubule-	MAPRE2
P17028;A0A(	P17028;A0A(	3;3	3;3	2;2	Zinc finger pr	ZNF24
Q6P1N4;A0A	Q6P1N4;A0A	4;4;4;4;4;2;2	4;4;4;4;4;2;2	4;4;4;4;4;2;2	Ras GTPase-a	IQGAP1
O43663;A0A(	O43663;A0A(	7;7;3;2;2;2;1	7;7;3;2;2;2;1	7;7;3;2;2;2;1	Protein regul	PRC1
A0A0J9YX88;	A0A0J9YX88;	2;2;2;2;2;2;2	2;2;2;2;2;2;2	2;2;2;2;2;2;2	PHD and RIN	PHRF1
P05387;A0A(	P05387;A0A(	2;2;1	2;2;1	2;2;1	60S acidic rib	RPLP2
Q7L311;A8K5	Q7L311;A8K5	4;4;4;1;1;1;1	4;4;4;1;1;1;1	4;4;4;1;1;1;1	Armadillo re	ARMCX2
O43309;A8K1	O43309;A8K1	2;2;2	2;2;2	1;1;1	Zinc finger ar	ZSCAN12
Q59HG5;Q15	Q59HG5;Q15	8;8;8;1;1	8;8;8;1;1	8;8;8;1;1	Zinc finger pr	ZKSCAN8
C9J423;Q9H5	C9J423;Q9H5	4;4;4;4;4;4;4	4;4;4;4;4;4;4	3;3;3;3;3;3;3	Zinc finger ar	ZSCAN31;ZNI
Q13838;A0A(	Q13838;A0A(	6;6;5;5;5;4;4	6;6;5;5;5;4;4	6;6;5;5;5;4;4	Spliceosome	DDX39B;DDX
B4DMT1;B2R	B4DMT1;B2R	2;2;2;2;2;2;2	2;2;2;2;2;2;2	2;2;2;2;2;2;2	Zinc finger ar	ZSCAN26
A0A0G2JIS2;(	A0A0G2JIS2;(	38;38;38;37;3	37;37;37;36;3	37;37;37;36;3	Histone-lysin	EHMT2
A0A024RCR6	A0A024RCR6	6;6;6;4;3;3;3	6;6;6;4;3;3;3	6;6;6;4;3;3;3	Large proline	BAG6
B4E063;Q9B\	B4E063;Q9B\	11;11;11;8;1;	11;11;11;8;1;	11;11;11;8;1;	Kinesin-like p	KIFC1
P17096;B4D\	P17096;B4D\	1;1;1	1;1;1	1;1;1	High mobility	HMGA1
B4DZZ8;A0A(	B4DZZ8;A0A(	1;1;1;1;1	1;1;1;1;1	1;1;1;1;1	Zinc transpor	SLC39A7
Q9Y3C6;A0A(	Q9Y3C6;A0A(	1;1	1;1	1;1	Peptidyl-prol	PPIL1
C9J7M8;A0A(	C9J7M8;A0A(	1;1;1;1	1;1;1;1	1;1;1;1	Transcription	TAF8
A8K2K2;Q9B.	A8K2K2;Q9B.	2;2;2;1;1;1;1	2;2;2;1;1;1;1	2;2;2;1;1;1;1	GTP-binding	GTPBP2
A0A0A0MTI6	A0A0A0MTI6	1;1;1;1;1	1;1;1;1;1	1;1;1;1;1	Elongation o	ELOVL5
O75376;A0A(	O75376;A0A(	7;7;5;4;3;3;3	7;7;5;4;3;3;3	7;7;5;4;3;3;3	Nuclear rece	NCOR1
B3KXK2;A0A(	B3KXK2;A0A(	2;2;2;2	2;2;2;2	2;2;2;2	Transcription	TRERF1;BCAF
Q6PK50;B4D	Q6PK50;B4D	2;2;2;2;2	1;1;1;1;1	1;1;1;1;1	Heat shock p	HSP90AB1
A0A024RDA6	A0A024RDA6	2;2	2;2	2;2	Insulin-like g	IGFBP7
Q9UNQ0;I7JE	Q9UNQ0;I7JE	5;5;5;2;2;2;2	5;5;5;2;2;2;2	5;5;5;2;2;2;2	ATP-binding	ABCG2
A0A024RDF4	A0A024RDF4	11;11;11;10;9	11;11;11;10;9	10;10;10;9;8;	Heterogeneo	HNRNPD
B4DTA2;A0A(	B4DTA2;A0A(	3;3;3;3	2;2;2;2	2;2;2;2	Heterogeneo	HNRPDL
P49207;A0A(	P49207;A0A(	2;2;1	2;2;1	2;2;1	60S ribosom	RPL34
Q9NY12;A0A	Q9NY12;A0A	2;2	2;2	2;2	H/ACA ribon	GAR1
A0A087WXN	A0A087WXN	2;2;2;1;1;1;1	2;2;2;1;1;1;1	2;2;2;1;1;1;1	Integrator co	INTS12
A1JUI8;B4DP	A1JUI8;B4DP	3;3;3;3;3;3;2	3;3;3;3;3;3;2	3;3;3;3;3;3;2	T-complex pr	CCT6A
A0A024RDP4	A0A024RDP4	1;1;1	1;1;1	1;1;1	Paraspeckle	PSPC1
B4DKE6;P40S	B4DKE6;P40S	12;12;12;11;1	12;12;12;11;1	12;12;12;11;1	Replication f	RFC3
Q5T7C4;B7ZS	Q5T7C4;B7ZS	4;4;4;4;4;3;3	4;4;4;4;4;3;3	3;3;3;3;3;3;2	High mobility	HMGB1;HMG
Q9UBW7;A0.	Q9UBW7;A0.	13;13;12;7;3	13;13;12;7;3	13;13;12;7;3	Zinc finger M	ZMYM2
R4GND2;B3K	R4GND2;B3K	1;1;1;1;1;1;1	1;1;1;1;1;1;1	1;1;1;1;1;1;1	Transcription	SUPT20H;FAI
B4E3J6;Q9N\	B4E3J6;Q9N\	4;4;4	4;4;4	4;4;4	Arginine and	ARGLU1

B3KWG6;B4E	B3KWG6;B4E	4;4;4;4;4;1	4;4;4;4;4;1	4;4;4;4;4;1	Importin-5	IPO5
A0A0F7W082;U3N9E1;Q50I06;Q9UQT7;A0ZXY8;F6IB50;I0J9E2;V6CKU4;X5EM81;X5CHJ2;X4YT						
8RHH1;F8RHG8;F8RHG2;F8RHG1;F8RHG0;F8RHF7;F8RHF6;F8RHF5;F8RHF1;F8RHF0;F8RHE9;F						
B6;A0A0A7C!	A0A0F7W082;U3N9E1;Q50I06;Q9UQT7;A0ZXY8;F6IB50;I0J9E2;V6CKU4;X5EM81					
8RHH1;F8RHG8;F8RHG2;F8RHG1;F8RHG0;F8RHF7;F8RHF6;F8RHF5;F8RHF1;F8RHF0;F8RHE9;F						
B6;A0A0A7C!	2;2;2;2;2;2;2	2;2;2;2;2;2;2	2;2;2;2;2;2;2	HLA class I hi	HLA-A;HLA-C	>tr|A0A0F7V
181;181;181;	1.67	6	3	0	By matching	By MS/MS
Q9Y314;A8K(	Q9Y314;A8K(	7;7;7;6;4;3;3	7;7;7;6;4;3;3	7;7;7;6;4;3;3	Nitric oxide s	NOSIP
A0A075B6Q3	A0A075B6Q3	14;8;5;1	13;8;4;1	2;0;2;0		BCORL1
A0A075B6Q5	A0A075B6Q5	1	1	1		
A0A075B6T1	A0A075B6T1	2;2;2;1;1	2;2;2;1;1	2;2;2;1;1	Activating m	AMBRA1
H0YN73;P08	H0YN73;P08	1;1;1;1	1;1;1;1	1;1;1;1	40S ribosoma	RPS17L;RPS1
A0A075B730	A0A075B730	4;4;3	1;1;1	1;1;1	Epiplakin	EPPK1
B4E312;A0A(	B4E312;A0A(	2;2;2;1	2;2;2;1	2;2;2;1	TATA-binding	TAF15
K7ERP4;A0A(	K7ERP4;A0A(	4;4;4;4;3;3;3	4;4;4;4;3;3;3	4;4;4;4;3;3;3	Glutathione	GPX4
A0A087WT1!	A0A087WT1!	1;1;1;1;1;1;1	1;1;1;1;1;1;1	1;1;1;1;1;1;1	Keratin-asso	KRTAP10-3;K
A0A087WTU	A0A087WTU	1;1;1;1	1;1;1;1	1;1;1;1	Kv channel-ir	KCNIP1
Q96T88;A0A(	Q96T88;A0A(	20;20;20;8;7	20;20;20;8;7	19;19;19;7;6	E3 ubiquitin-	UHRF1
A0A087WTY!	A0A087WTY!	1;1;1	1;1;1	1;1;1	MORF4 famil	MRFAP1
E9PL01;Q15(	E9PL01;Q15(	2;2;2;2;2;1	2;2;2;2;2;1	2;2;2;2;2;1	Signal peptid	SPCS2
Q14BN2;A0A	Q14BN2;A0A	2;2;2;2;2;1;1	2;2;2;2;2;1;1	2;2;2;2;2;1;1	GON-4-like p	GON4L;YY1A
D9ZGF5;P09(	D9ZGF5;P09(	4;4;4	4;4;4	4;4;4	Fibroblast gr	FGF2
A0A087WZG!	A0A087WZG!	1;1;1;1;1;1	1;1;1;1;1;1	1;1;1;1;1;1	Retrotranspo	hCG_164274
A0A087WUL!	A0A087WUL!	4;4	4;4	4;4	Polycomb gro	PCGF1
E7ETK0;A0A(	E7ETK0;A0A(	2;2;2	2;2;2	2;2;2	40S ribosoma	RPS24
A0A087WV0	A0A087WV0	1;1;1;1;1;1;1	1;1;1;1;1;1;1	1;1;1;1;1;1;1	Zinc finger pr	ZFP30;ZFP82
A0A087WV6(	A0A087WV6(	162;162;4;1;	162;162;4;1;	147;147;4;0;	Antigen KI-67	MKI67
P46087;A0A(	P46087;A0A(	10;10;2;1	10;10;2;1	10;10;2;1	Putative ribo	NOP2
A0A087WYH	A0A087WYH	1;1;1	1;1;1	1;1;1	Zinc finger pr	ZNF407
B4DED6;A0A	B4DED6;A0A	8;8;8;7;6;6;2	6;6;6;5;4;4;2	6;6;6;5;4;4;2	ATP-depende	DDX50;mcdrl
B4DI61;A0A(	B4DI61;A0A(	4;4;4;4;4;3	4;4;4;4;4;3	4;4;4;4;4;3	Protein CYR6	CYR61
B2RD24;A0A(	B2RD24;A0A(	3;3;3;3;1;1;1	3;3;3;3;1;1;1	3;3;3;3;1;1;1	Monofunctio	MTHFD1L
Q14517;A0A(	Q14517;A0A(	1;1	1;1	1;1	Protocadheri	FAT1
B3KUD6;P53	B3KUD6;P53	2;2;2;2;2;2;2	2;2;2;2;2;2;2	2;2;2;2;2;2;2	Smoothelin	SMTN
A0A087WYS	A0A087WYS	1;1;1;1;1	1;1;1;1;1	1;1;1;1;1	Protein kinas	ZMYND8
A0A087WVZ!	A0A087WVZ!	1;1;1;1	1;1;1;1	1;1;1;1	DNA-directe	POLR2E
Q86W17;Q5	Q86W17;Q5	1;1;1;1;1;1;1	1;1;1;1;1;1;1	1;1;1;1;1;1;1	Putative tryp	PRSS1;PRSS2
P24928;A0A(	P24928;A0A(	6;6;6;1	6;6;6;1	6;6;6;1	DNA-directe	POLR2A
D3DR86;A0A	D3DR86;A0A	9;9;9;1;1;1	9;9;9;1;1;1	9;9;9;1;1;1	Nuclear facto	NFKB2
A0A087WWF	A0A087WWF	3;3;1	3;3;1	3;3;1	Putative RNA	RBM15
A0A0C4DH7!	A0A0C4DH7!	1;1;1;1	1;1;1;1	1;1;1;1	Round sperm	RSBN1
Q68E00;A0A(	Q68E00;A0A(	1;1;1	1;1;1	1;1;1	Proteasomal	DKFZp686G2
A0A087WX6(	A0A087WX6(	3;3;1	2;2;1	2;2;1	Myopalladin	MYPN
F8WBF5;B7Z	F8WBF5;B7Z	1;1;1;1;1;1;1	1;1;1;1;1;1;1	1;1;1;1;1;1;1	Dual specifici	CLK1;CLK4
A0A087WXF	A0A087WXF	1;1;1	1;1;1	1;1;1	Inhibitor of g	ING1

A0A087WXU	A0A087WXU	3;3;3;3;3;3;2;	3;3;3;3;3;3;2;	3;3;3;3;3;3;2;	Nucleolar pro	ZCCHC17
Q9H4H8;A0A	Q9H4H8;A0A	3;3	3;3	3;3	Protein FAM8	FAM83D
E9PM37;E9P	E9PM37;E9P	1;1;1;1;1;1;1;	1;1;1;1;1;1;1;	1;1;1;1;1;1;1;	Forkhead bo	FOXK2;FOXK
E9KY40;Q2L4	E9KY40;Q2L4	1;1;1;1;1;1	1;1;1;1;1;1	1;1;1;1;1;1	Paired box p	PAX9;Pax9;P
A0A087WXZ	A0A087WXZ	6;6;6;2;2;1;1	6;6;6;2;2;1;1	4;4;4;2;0;1;0	YTH domain	YTHDF2
A0A087WXZ	A0A087WXZ	1;1	1;1	1;1	Protein SREK	SREK1IP1
A0A087WY8	A0A087WY8	1;1;1	1;1;1	1;1;1	Keratin-asso	KRTAP4-1
A0A087WYZ	A0A087WYZ	3;3;2;2;2;2;2;	1;1;1;1;1;1;1;	0;0;0;0;0;0;0;	Putative zinc	ZNF852;ZNF6
K7EKR9;Q8IY	K7EKR9;Q8IY	1;1;1;1	1;1;1;1	1;1;1;1	Ribonucleop	RAVER1
M0R210;A0A	M0R210;A0A	4;4;4;3;3;2;2;	4;4;4;3;3;2;2;	4;4;4;3;3;2;2;	40S ribosom	RPS16
D6R9P3;D6R	D6R9P3;D6R	4;4;4;4;4;4;4	4;4;4;4;4;4;4	4;4;4;4;4;4;4	Heterogeneo	HNRNPAB
A0A087WZZ	A0A087WZZ	16;16;14;14;	16;16;14;14;	16;16;14;14;	Splicing facto	SF3B2;DKFZp
A0A087X1K6	A0A087X1K6	1;1;1	1;1;1	1;1;1	Krueppel-like	KLF13
B2R5U7;A0A	B2R5U7;A0A	6;6;6;2	6;6;6;2	6;6;6;2	CCAAT/enha	CEBPZ
B2RDK5;A0A	B2RDK5;A0A	2;2;2;2;1	2;2;2;2;1	2;2;2;2;1	Stromal mem	SMAP1
H7C2D3;C9J	H7C2D3;C9J	1;1;1;1	1;1;1;1	1;1;1;1	Round sperm	RSBN1L
Q6PKC6;B3K	Q6PKC6;B3K	1;1;1;1;1;1;1;	1;1;1;1;1;1;1;	1;1;1;1;1;1;1;	WD repeat-c	WDR6;DKFZp
B4DP70;F1T	B4DP70;F1T	1;1;1;1;1	1;1;1;1;1	1;1;1;1;1	ATP-depende	DDX1
D6RIC3;Q9Y	D6RIC3;Q9Y	4;4;4;4;3;3;2;	4;4;4;4;3;3;2;	4;4;4;4;3;3;2;	Nucleolar pro	NOP16
Q14525;CON	Q14525;CON	16;16;16;1	4;4;4;0	3;3;3;0	Keratin, type	KRT33B
S4S3R8;Q15	S4S3R8;Q15	4;4;4;3;3;3;2;	4;4;4;3;3;3;2;	4;4;4;3;3;3;2;	Histone-lysin	EZH2
Q8TA86;A0A	Q8TA86;A0A	7;7;6;4;2	7;7;6;4;2	7;7;6;4;2	Retinitis pigm	RP9
F5H0Q6;B3K	F5H0Q6;B3K	1;1;1;1	1;1;1;1	1;1;1;1	CLK4-associa	CLASRP
C9JP00;Q9N	C9JP00;Q9N	3;3;3;2;1;1;1;	3;3;3;2;1;1;1;	3;3;3;2;1;1;1;	Muscleblind-	MBNL1
P98175;Q7Z	P98175;Q7Z	6;6;6;4	6;6;6;4	6;6;6;4	RNA-binding	RBM10;DKFZ
K7ENH8;Q6P	K7ENH8;Q6P	2;2;2;2;2;1;1;	2;2;2;2;2;1;1;	2;2;2;2;2;1;1;	Transcription	TCF3
F8WDT3;F8W	F8WDT3;F8W	1;1;1;1;1;1;1	1;1;1;1;1;1;1	1;1;1;1;1;1;1	Protein-L-iso	PCMT1
Q14978;B2R	Q14978;B2R	15;15;15;10;	15;15;15;10;	13;13;13;10;	Nucleolar an	NOLC1
A0A0A0MRP	A0A0A0MRP	4;4	4;4	4;4	MAP7 domai	MAP7D3
Q86UA8;P28	Q86UA8;P28	14;14;14;14;	3;3;3;3;1	3;3;3;3;1	Probable glo	SMARCA1
Q5TAL4;Q53	Q5TAL4;Q53	1;1;1;1	1;1;1;1	1;1;1;1	U1 small nuc	SNRPC
B6VEX5;B6V	B6VEX5;B6V	1;1;1;1;1;1	1;1;1;1;1;1	1;1;1;1;1;1	Abl interacto	ABI1
Q9P2E9;A0A	Q9P2E9;A0A	4;4;4;3;3;3;3;	4;4;4;3;3;3;3;	4;4;4;3;3;3;3;	Ribosome-bi	RRBP1
Q5VW28;O0	Q5VW28;O0	5;5;5;5;5;5;4;	3;3;3;3;3;3;2;	3;3;3;3;3;3;2;	Nuclear facto	NFIB
Q6PJR5;Q5U	Q6PJR5;Q5U	2;2;2;2;2;2;2;	2;2;2;2;2;2;2;	2;2;2;2;2;2;2;	Zinc finger M	ZMYND11
Q9BV39;Q68	Q9BV39;Q68	1;1;1;1;1;1	1;1;1;1;1;1	1;1;1;1;1;1	Multidrug re	DKFZp781G1
Q6I9R8;Q14	Q6I9R8;Q14	9;9;9;9;9;8;8;	9;9;9;9;9;8;8;	9;9;9;9;9;8;8;	Four and a h	FHL2;AAG11
B4DG61;A0A	B4DG61;A0A	33;33;32;32;	33;33;32;32;	30;30;29;29;	Polyhomeoti	PHC2;HPH2
A7E2S2;B4D	A7E2S2;B4D	1;1;1;1;1;1;1;	1;1;1;1;1;1;1;	1;1;1;1;1;1;1;	Plastin-3	PLS3
A0A0A0MTB	A0A0A0MTB	5;5	5;5	5;5	WD repeat-c	WDR36
V9HWF0;Q1	V9HWF0;Q1	5;5;5;1	5;5;5;1	5;5;5;1	Integrin-link	ILK
Q2M1V9;O1	Q2M1V9;O1	1;1;1	1;1;1	1;1;1	TATA-binding	BTAF1
Q5JRY4;B4E1	Q5JRY4;B4E1	1;1;1;1;1;1;1;	1;1;1;1;1;1;1;	1;1;1;1;1;1;1;	Transcription	TCF7L2
B4E2I1;B4DL	B4E2I1;B4DL	2;2;2;2;2	2;2;2;2;2	2;2;2;2;2	Krueppel-like	KLF5
Q86VU2;A0P	Q86VU2;A0P	1;1;1;1;1;1	1;1;1;1;1;1	1;1;1;1;1;1	Ribosomal pr	RPS6KA4

A0A0A6YYG7	A0A0A6YYG7	4;4;2;1	4;4;2;1	4;4;2;1	Condensin-2	NCAPH2
Q9NQ55;A0A	Q9NQ55;A0A	4;4;4;3;2;1	4;4;4;3;2;1	4;4;4;3;2;1	Suppressor o	PPAN
Q9H6W3;A0	Q9H6W3;A0	2;2	2;2	2;2	Bifunctional l	NO66
A0A0B4J1Z1;	A0A0B4J1Z1;	8;8;8;8;6;3	7;7;7;7;6;3	7;7;7;7;6;3	Serine/argini	SRSF7
E9PLD3;E9PR	E9PLD3;E9PR	1;1;1;1	1;1;1;1	1;1;1;1		C11orf48
F8VV32;A0A	F8VV32;A0A	1;1;1;1	1;1;1;1	1;1;1;1	Lysozyme C	LYZ
E7EUL6;Q03	E7EUL6;Q03	3;3;3;3;3;3;1	3;3;3;3;3;3;1	3;3;3;3;3;3;1	MDS1 and E	MECOM;EVI1
B7Z7W2;H0Y	B7Z7W2;H0Y	1;1;1;1;1;1;1	1;1;1;1;1;1;1	1;1;1;1;1;1;1	Regulating sy	RIMS1
A0A0C4DFX7	A0A0C4DFX7	9;9;1	9;9;1	9;9;1	WD repeat-c	WDR76
A0PJ71;A0A0	A0PJ71;A0A0	1;1;1;1;1	1;1;1;1;1	1;1;1;1;1	Nibrin	NBN
F5GYV5;H7B	F5GYV5;H7B	7;7;7;7;7;7;6	7;7;7;7;7;7;6	7;7;7;7;7;7;6	ADP-ribosyla	ARL6IP4;FLJ0
A8K6X3;A0A	A8K6X3;A0A	20;20;19;16;	20;20;19;16;	20;20;19;16;	Probable ATP	DDX46
Q59GQ7;A0A	Q59GQ7;A0A	11;11;11;10;	11;11;11;10;	11;11;11;10;	Helicase-like	HLTF
B3KY63;F5G	B3KY63;F5G	26;26;26;26;	26;26;26;26;	20;20;20;20;	Chromodoma	CHD4
A0A0C4DGL3	A0A0C4DGL3	3;3;3;3;2;2;2	3;3;3;3;2;2;2	3;3;3;3;2;2;2	Deoxyuridine	DUT
A0A0C4DGP	A0A0C4DGP	4;4;4;2	4;4;4;2	4;4;4;2	Kinesin-like p	KIF18B
O95218;A0A	O95218;A0A	5;5	5;5	5;5	Zinc finger R	ZRANB2
B4DKV5;Q96	B4DKV5;Q96	1;1;1;1;1;1	1;1;1;1;1;1	1;1;1;1;1;1	Constitutive	FAM120B
A0A0D9SFB3	A0A0D9SFB3	32;32;32;32;	31;31;31;31;	31;31;31;31;	ATP-depende	DDX3X;DDX3
A0A0D9SF63	A0A0D9SF63	7;7;6;3;3;3;2	7;7;6;3;3;3;2	7;7;6;3;3;3;2	F-box-like/W	TBL1XR1
Q8IWS0;A0A	Q8IWS0;A0A	9;9;7;5	9;9;7;5	9;9;7;5	PHD finger p	PHF6
A0A0F6QNT	A0A0F6QNT	2;2	2;2	2;2	E3 ubiquitin-	TOPORS
E9PFF5;B4D	E9PFF5;B4D	3;3;3;3;3;3;2	3;3;3;3;3;3;2	3;3;3;3;3;3;2	Fragile X me	FXR1
Q8N8Y5;A0A	Q8N8Y5;A0A	4;4;4	4;4;4	4;4;4	Zinc finger pr	ZFP41;DKFZp
P0DMV9;P0D	P0DMV9;P0D	25;25;25;25;	21;21;21;21;	1;1;1;1;1;0;0;	0;0;0;0;0;0;0	HSPA1B
H0Y6G3;A0A	H0Y6G3;A0A	1;1;1;1;1;1;1	1;1;1;1;1;1;1	1;1;1;1;1;1;1	WD repeat-c	WDR46
Q9BYR2;Q9B	Q9BYR2;Q9B	3;3;3;3;3;3;2	3;3;3;3;3;3;2	3;3;3;3;3;3;2	Keratin-asso	KRTAP4-5;KR
Q14160;A0A	Q14160;A0A	12;12;12;11;	12;12;12;11;	11;11;11;11;	Protein scrib	SCRIB
A0A0G2JRI7;	A0A0G2JRI7;	1;1;1;1	1;1;1;1	1;1;1;1	Lysophospha	AYTL2;LPCAT
B0QYV1;A0A	B0QYV1;A0A	9;9;9;7;7;7;7	9;9;9;7;7;7;7	9;9;9;7;7;7;7	RNA binding	RBM9;RBFO
A0A0G2JRY5	A0A0G2JRY5	2;2;1;1;1;1;1	2;2;1;1;1;1;1	2;2;1;1;1;1;1	Transcription	TAF4;TAF4B;
Q86WI7;C9J	Q86WI7;C9J	1;1;1;1;1;1	1;1;1;1;1;1	1;1;1;1;1;1	SWI/SNF-rela	SMARCB1
A0A0J9YYL3;	A0A0J9YYL3;	11;11;11;11;	11;11;11;11;	11;11;11;11;	Poly(U)-bindi	PUF60
J3QT77;Q15	J3QT77;Q15	1;1;1;1;1;1	1;1;1;1;1;1	1;1;1;1;1;1	Serum parao	PON2
A0A0J9YYJ0;	A0A0J9YYJ0;	2;2	2;2	2;2	Protein cano	CNPY4
A7E2X7;A0A	A7E2X7;A0A	3;3;3	3;3;3	3;3;3	DNA topoiso	TOPBP1
Q99728;Q53	Q99728;Q53	3;3;3;3;2;2	3;3;3;3;2;2	3;3;3;3;2;2	BRCA1-assoc	BARD1
A0JLS5;Q127	A0JLS5;Q127	4;4;3;3	4;4;3;3	4;4;3;3	Transducin b	TBL3
A0JLU2;B4D	A0JLU2;B4D	4;4;4;3	4;4;4;3	4;4;4;3	Mitotic chec	BUB1
Q2TAY7;A0M	Q2TAY7;A0M	2;2	2;2	2;2	WD40 repea	SMU1
Q9BQ67;A0M	Q9BQ67;A0M	8;8;7;7;7;6;4	8;8;7;7;7;6;4	8;8;7;7;7;6;4	Glutamate-ri	GRWD1;DKF
Q96DI7;B2R	Q96DI7;B2R	4;4;4;2	4;4;4;2	4;4;4;2	U5 small nuc	SNRNP40;W
A0PJ52;Q6A	A0PJ52;Q6A	2;2;2	2;2;2	2;2;2	F-box only pr	FBXO38
J3QSV6;A0PJ	J3QSV6;A0PJ	14;14;14;14;	14;14;14;14;	14;14;14;14;	Ribosomal L1	RSL1D1
E7EPB3;A0PJ	E7EPB3;A0PJ	4;4;4;4;4;4	4;4;4;4;4;4	1;1;1;1;1;1	60S ribosom	RPL14

A0PJ70;Q8TF	A0PJ70;Q8TF	12;12;12	12;12;12	12;12;12	Serine/threo	GSG2
Q2TAL8;A1L3	Q2TAL8;A1L3	5;5;3;2	5;5;3;2	5;5;3;2	Glutamine-ri	QRICH1
Q96DV6;P62	Q96DV6;P62	6;6;6;4;3;2	6;6;6;4;3;2	6;6;6;4;3;2	40S ribosoma	RPS6
B4DHJ4;A2RU	B4DHJ4;A2RU	1;1;1;1	1;1;1;1	1;1;1;1	MORC family	MORC3
B3KTM6;P46	B3KTM6;P46	11;11;11;10;	11;11;11;10;	11;11;11;10;	60S ribosoma	RPL5
A2VCR1;B3K	A2VCR1;B3K	1;1;1;1;1	1;1;1;1;1	1;1;1;1;1	Exosome con	EXOSC2
Q6NUN2;O1	Q6NUN2;O1	12;12;12;11;	12;12;12;11;	12;12;12;11;	NF-kappa-B-r	NKRF;NRF
Q4VB24;P10	Q4VB24;P10	10;10;10;10;	2;2;2;2;2;0;0;	2;2;2;2;2;0;0;	Histone H1.4	HIST1H1E;HIS
A4D110	A4D110	1	1	1		LOC401309
Q13185;A4D	Q13185;A4D	6;6;2;2;2;1;1;	6;6;2;2;2;1;1;	5;5;2;2;2;1;1;	Chromobox p	CBX3
Q8N201;A4D	Q8N201;A4D	4;4;4;1	4;4;4;1	4;4;4;1	Integrator co	INTS1;DKFZP
A4D2A4;Q86	A4D2A4;Q86	2;2;2;2;2	1;1;1;1;1	0;0;0;0;0	Zinc finger pr	ZNF3;DKFZp6
O94901;E9PI	O94901;E9PI	3;3;3;2;2;1;1;	3;3;3;2;2;1;1;	3;3;3;2;2;1;1;	SUN domain-	SUN1;UNC84
Q1XBU8;O75	Q1XBU8;O75	2;2;2;1;1;1;1;	2;2;2;1;1;1;1;	2;2;2;1;1;1;1;	E3 SUMO-pro	PIAS1;PIAS2
H0YMJ0;H0Y	H0YMJ0;H0Y	3;3;3;3;3;2;2;	3;3;3;3;3;2;2;	3;3;3;3;3;2;2;	Mortality fac	MORF4L1
A5D8Z7;Q9B	A5D8Z7;Q9B	1;1	1;1	1;1	Sialoadhesin	SIGLEC1
A7YDK7;D2C	A7YDK7;D2C	1;1;1;1;1;1;1;	1;1;1;1;1;1;1;	1;1;1;1;1;1;1;	AH receptor-	AIP
B4DXG8;D6R	B4DXG8;D6R	1;1;1;1	1;1;1;1	1;1;1;1	Exosome con	EXOSC9
A6H8X9;B2R	A6H8X9;B2R	3;3;3;3;1;1;1;	3;3;3;3;1;1;1;	3;3;3;3;1;1;1;	Centrosomal	CEP170
Q9NTG1;A6N	Q9NTG1;A6N	2;2	2;2	2;2	Polycystic kic	PKDREJ
A6NCN2	A6NCN2	13	2	2	Keratin-81-lik	KRT121P
P51610;A6N	P51610;A6N	24;24;11;6	24;24;11;6	24;24;11;6	Host cell fact	HCFC1
A6NEM5;Q9	A6NEM5;Q9	2;2;1	2;2;1	2;2;1	GPI-anchor tr	PIGK
Q14202;A6N	Q14202;A6N	17;17;16;2;2	17;17;16;2;2	17;17;16;2;2	Zinc finger M	ZMYM3
A6NHR9;J3K	A6NHR9;J3K	8;6	8;6	8;6	Structural ma	SMCHD1
B7Z3E7;A6N	B7Z3E7;A6N	8;8;8;5;4;3;1	8;8;8;5;4;3;1	7;7;7;5;4;3;1	Tumor suppr	TP53BP1
Q9NSJ7;A6N	Q9NSJ7;A6N	1;1;1	1;1;1	1;1;1	Ubiquitin car	DKFZp434K1
Q9H8A8;A6P	Q9H8A8;A6P	2;2;2;2;1;1;1;	2;2;2;2;1;1;1;	2;2;2;2;1;1;1;	Selenium-bin	SELENBP1
P51532;A7E2	P51532;A7E2	22;22;22;22;	22;22;22;22;	22;22;22;22;	Transcription	SMARCA4
B2RNX8;A7N	B2RNX8;A7N	1;1;1	1;1;1	1;1;1	Protein cram	CRAMP1L
A7MD96;Q8	A7MD96;Q8	1;1;1	1;1;1	1;1;1	Synaptopodi	SYNPO
Q05DQ6;O14	Q05DQ6;O14	4;4;4;1	4;4;4;1	4;4;4;1	Kinetochore	NDC80;KNTC
G3XAN4;Q6F	G3XAN4;Q6F	1;1;1;1;1	1;1;1;1;1	1;1;1;1;1	Translocating	TRAM1
Q68CQ4;A8K	Q68CQ4;A8K	3;3	3;3	3;3	Digestive org	DIEXF
A8K194;P541	A8K194;P541	6;6;4;2	6;6;4;2	6;6;4;2	Protein HIRA	HIRA
Q5U0N1;Q1	Q5U0N1;Q1	3;3;3;1	3;3;3;1	3;3;3;1	Myeloid leuk	MLF2
Q9H081;A8K	Q9H081;A8K	2;2	2;2	2;2	Protein MIS1	MIS12
H7C128;H7C	H7C128;H7C	3;3;3;3;3;3;2;	3;3;3;3;3;3;2;	3;3;3;3;3;3;2;	Bromodomai	BRD8
J3QQW9;Q1	J3QQW9;Q1	4;4;4	4;4;4	4;4;4	Polycomb pro	SUZ12
A8K201;Q9Y	A8K201;Q9Y	5;5;1	5;5;1	5;5;1	RRP15-like pr	RRP15
O75600;A8K	O75600;A8K	1;1	1;1	1;1	2-amino-3-ke	GCAT
B2RCW4;A8K	B2RCW4;A8K	2;2;2;1	2;2;2;1	2;2;2;1	Zinc finger ar	ZBTB20
B4DRR0;P02	B4DRR0;P02	36;36;36;36;	3;3;3;3;3;2	2;2;2;2;2;1	Keratin, type	KRT6A
B7Z4C6;Q71	B7Z4C6;Q71	5;5;5;5;5;5;4	5;5;5;5;5;5;4	5;5;5;5;5;5;4	Lipoprotein li	LPL
Q6MZZ7;A8K	Q6MZZ7;A8K	1;1	1;1	1;1	Calpain-13	CAPN13

A8K2R3;O60:	A8K2R3;O60:	9;9;5;4	9;9;5;4	9;9;5;4	Lysine-specif	KDM1A
B7Z1C7;B7Z2	B7Z1C7;B7Z2	10;10;10;10;:	3;3;3;3;3;3;3;	3;3;3;3;3;3;3;	Scaffold atta(	SAFB
A8K330;Q8IV	A8K330;Q8IV	3;3;1	3;3;1	3;3;1	WD repeat-c(	WDR75
A8K394	A8K394	49	1	1		
B7Z2F4;B7Z9	B7Z2F4;B7Z9	3;3;3;3;3	3;3;3;3;3	3;3;3;3;3	T-complex pr	CCT4
O95232;A8K:	O95232;A8K:	10;10;9;9;8;6	10;10;9;9;8;6	10;10;9;9;8;6	Luc7-like pro	LUC7L3;CROI
Q96NJ6;A8K:	Q96NJ6;A8K:	2;2	1;1	1;1	Zinc finger pr	ZFP3
B4DQY1;B4D	B4DQY1;B4D	3;3;3;3;3;2;2,	3;3;3;3;3;2;2,	3;3;3;3;3;2;2,	Serine/threo	PPP2R1A;PPI
Q13242;A8K:	Q13242;A8K:	7;7;6;6;4	6;6;5;5;4	6;6;5;5;4	Serine/argini	SRSF9
A8K3Q9	A8K3Q9	4	1	1		
Q6DKJ9;Q4G	Q6DKJ9;Q4G	1;1;1;1;1;1	1;1;1;1;1;1	1;1;1;1;1;1	Ribosome bi(	BOP1
P40937;A8K3	P40937;A8K3	5;5;5;4;4;3;2,	5;5;5;4;4;3;2,	5;5;5;4;4;3;2,	Replication fa	RFC5
Q99661;A8K:	Q99661;A8K:	26;26;23;21;:	26;26;23;21;:	25;25;22;20;:	Kinesin-like p	KIF2C
Q4G1A1;Q9H	Q4G1A1;Q9H	3;3;3;2;2;1	3;3;3;2;2;1	3;3;3;2;2;1	Kinetochore-	DSN1
Q9NX58;A8K	Q9NX58;A8K	3;3;1	3;3;1	3;3;1	Cell growth-r	LYAR
C9JW96;Q6P	C9JW96;Q6P	1;1;1;1;1	1;1;1;1;1	1;1;1;1;1	Prohibitin	PHB
V9HWF5;P62	V9HWF5;P62	9;9;9;8;7;7;7,	9;9;9;8;7;7;7,	9;9;9;8;7;7;7,	Peptidyl-prol	PPIA
Q6AHX7;Q15	Q6AHX7;Q15	1;1;1	1;1;1	1;1;1	Pre-mRNA-sp	DKFZp686F2(
Q9BXS6;A8K4	Q9BXS6;A8K4	3;3	3;3	3;3	Nucleolar an(	NUSAP1
Q6NZ55;P26:	Q6NZ55;P26:	9;9;9;4;3;2;1,	9;9;9;4;3;2;1,	9;9;9;4;3;2;1,	60S ribosoma	RPL13
Q59H57;B4D	Q59H57;B4D	5;5;5;5;5;5;5,	5;5;5;5;5;5;5,	5;5;5;5;5;5;5,	RNA-binding	FUS
Q96G23;A8K	Q96G23;A8K	3;3;2;2;2;2;2,	3;3;2;2;2;2;2,	3;3;2;2;2;2;2,	Ceramide syr	CERS2
Q14146;A8K4	Q14146;A8K4	6;6;4	6;6;4	6;6;4	Unhealthy rik	URB2
B4DSJ1;Q5QI	B4DSJ1;Q5QI	3;3;3;3;3;3;2,	3;3;3;3;3;3;2,	3;3;3;3;3;3;2,	RNA-binding	RALY
F5H0J3;Q9BT	F5H0J3;Q9BT	1;1;1;1	1;1;1;1	1;1;1;1	NADH dehyd	NDUFA9
Q6NXR8;P61	Q6NXR8;P61	9;9;9;8;8;8;7,	9;9;9;8;8;8;7,	9;9;9;8;8;8;7,	40S ribosoma	RPS3A
H3BPC4;H3B	H3BPC4;H3B	2;2;2;2;2;2	2;2;2;2;2;2	2;2;2;2;2;2	SUMO-conju	UBE2I
P62266;A8K5	P62266;A8K5	4;4;3;1;1;1	4;4;3;1;1;1	4;4;3;1;1;1	40S ribosoma	RPS23
B1AHB1;Q53	B1AHB1;Q53	15;15;15;15;:	15;15;15;15;:	15;15;15;15;:	DNA replicati	MCM5
Q15233;A8K5	Q15233;A8K5	11;11;8;6;4	11;11;8;6;4	11;11;8;6;4	Non-POU doi	NONO
Q8IZL8;A8K5	Q8IZL8;A8K5	18;18;17;17;:	18;18;17;17;:	18;18;17;17;:	Proline-, glut	PELP1
B4DW90;Q5:	B4DW90;Q5:	2;2;2;2;2;2	2;2;2;2;2;2	2;2;2;2;2;2	Splicing facto	SF3A3
Q59GY3;V9H	Q59GY3;V9H	7;7;7;7;3;3;2,	7;7;7;7;3;3;2,	4;4;4;4;0;0;0,	Serine/argini	SRSF6
Q9P0V3;A8K!	Q9P0V3;A8K!	1;1	1;1	1;1	SH3 domain-	SH3BP4
Q6IQ21;A8K5	Q6IQ21;A8K5	2;2	2;2	2;2	Zinc finger pr	ZNF770
Q59EF5;Q08:	Q59EF5;Q08:	4;4;4	1;1;1	1;1;1	Serine/argini	SRSF4;SFRS4
Q07021;A8K(	Q07021;A8K(	1;1	1;1	1;1	Complement	C1QBP
Q9UBL3;A8K(	Q9UBL3;A8K(	8;8;7;7;3;2;1,	8;8;7;7;3;2;1,	8;8;7;7;3;2;1,	Set1/Ash2 hi:	ASH2L
Q15393;A8K(	Q15393;A8K(	10;10;8;5;1	10;10;8;5;1	10;10;8;5;1	Splicing facto	SF3B3
B4DMI9;Q15	B4DMI9;Q15	6;6;6	6;6;6	6;6;6	Disks large-a:	DLGAP5
Q5STV6;Q9U	Q5STV6;Q9U	3;3;3;3;2	3;3;3;3;2	3;3;3;3;2	NF-kappa-B i	NFKBIL1
Q7Z726;Q6N	Q7Z726;Q6N	5;5;5;5;3;3	5;5;5;5;3;3	5;5;5;5;3;3	Importin sub	KPNA2
P60842;A8K7	P60842;A8K7	3;3;2;2;2;2;2,	2;2;1;1;1;1;1,	2;2;1;1;1;1;1,	Eukaryotic in	EIF4A1;EIF4A
B3KUU7;A8K	B3KUU7;A8K	10;10;10;10;!	10;10;10;10;!	10;10;10;10;!	Dual specifici	CLK3
H3BVC7;I3L3	H3BVC7;I3L3	1;1;1;1;1	1;1;1;1;1	1;1;1;1;1	40S ribosoma	RPS15A;hCG_

O43929;A8K7	O43929;A8K7	2;2;1;1;1;1;1	2;2;1;1;1;1;1	2;2;1;1;1;1;1	Origin recogr	ORC4;ORC4L
B7Z8N6;Q9B	B7Z8N6;Q9B	1;1;1;1;1	1;1;1;1;1	1;1;1;1;1	Dual specifici	CLK2
Q14592;A8K7	Q14592;A8K7	3;3	3;3	2;2	Zinc finger pr	ZNF460
Q9H7B2;A8K	Q9H7B2;A8K	3;3;2;1	3;3;2;1	3;3;2;1	Ribosome pr	RPF2
A8K818;O15	A8K818;O15	4;3;2;2	4;3;2;2	4;3;2;2	DNA-directec	CD3EAP
O43790;CON	O43790;CON	22;22;22;22;	2;2;2;2;1;1;1	0;0;0;0;0;0;0	Keratin, type	KRT86
Q9BTT6;A8K	Q9BTT6;A8K	4;4;2	3;3;1	3;3;1	Leucine-rich	LRRC1
C9IYN3;A8K8	C9IYN3;A8K8	13;13;13;13;	12;12;12;12;	12;12;12;12;	Heterogeneo	HNRPLL;HNR
C9JL92;C9JID	C9JL92;C9JID	1;1;1;1;1	1;1;1;1;1	1;1;1;1;1	NGFI-A-bindi	NAB1
Q8WTT2;A8K	Q8WTT2;A8K	3;3;1	3;3;1	3;3;1	Nucleolar co	NOC3L
K7EP06;Q05I	K7EP06;Q05I	3;3;3;3;2;1;1	3;3;3;3;2;1;1	3;3;3;3;2;1;1	mRNA cap gu	RNMT
E9PKP7;P174	E9PKP7;P174	14;14;14;12;	14;14;14;12;	14;14;14;12;	Nucleolar tra	UBTF
Q9H307;A8K	Q9H307;A8K	12;12;5;5;2;2	12;12;5;5;2;2	12;12;5;5;2;2	Pinin	PNN
O00567;A8K	O00567;A8K	24;24;22;12;	24;24;22;12;	24;24;22;12;	Nucleolar pro	NOP56
Q7Z2W4;A8K	Q7Z2W4;A8K	2;2;1;1	2;2;1;1	2;2;1;1	Zinc finger C	ZC3HAV1
X5D2J9;Q49	X5D2J9;Q49	10;10;10;10;	10;10;10;10;	10;10;10;10;	General tran	GTF2I
E9PFK5;P783	E9PFK5;P783	2;2;2;1	2;2;2;1	2;2;2;1	Nucleolar pro	NOP14
B4DYU4;P42	B4DYU4;P42	1;1;1;1	1;1;1;1	1;1;1;1	Phosphatidyl	PIK3CA;PIK3C
J3KNR6;P486	J3KNR6;P486	1;1;1	1;1;1	1;1;1	Phosphatidyl	PTDSS1
Q70T21;Q70	Q70T21;Q70	2;2;2;2	2;2;2;2	2;2;2;2	Cyclic AMP-r	CREB3L2
P55199;A8K	P55199;A8K	5;5;4;4;3;1;1	5;5;4;4;3;1;1	5;5;4;4;3;1;1	RNA polymer	ELL
Q6IBM8;Q15	Q6IBM8;Q15	11;11;11;10;	11;11;11;10;	10;10;10;9;9;	116 kDa U5 s	U5-116KD;EF
Q68CX8;A8K	Q68CX8;A8K	2;2;2;1	2;2;2;1	2;2;2;1	Ancient ubiq	DKFZp686P1
J3QLE5;Q66K	J3QLE5;Q66K	3;3;3;3;3;3;3	3;3;3;3;3;3;3	3;3;3;3;3;3;3	Small nuclea	SNRPN;SNRP
Q49AN9;F5H	Q49AN9;F5H	2;2;2;2	2;2;2;2	2;2;2;2	Small nuclea	SNRPG
P43243;A8M	P43243;A8M	43;43;40;39;	43;43;40;39;	43;43;40;39;	Matrin-3	MATR3;DKFZ
F2Z2C0;Q9Y	F2Z2C0;Q9Y	1;1;1	1;1;1	1;1;1	Exosome con	DIS3
B0AZM1;P27	B0AZM1;P27	6;6;5;5;3;3;2	6;6;5;5;3;3;2	6;6;5;5;3;3;2	Aryl hydroca	ARNT
Q9UQE7;Q8	Q9UQE7;Q8	21;21;21	21;21;21	21;21;21	Structural ma	SMC3
B0AZS5;O00	B0AZS5;O00	2;2;1	1;1;1	1;1;1	Kinesin-like p	KIF2A
B0QXZ6;O95	B0QXZ6;O95	4;4	3;3	3;3	Chromobox p	CBX6
B0QYK0;Q01	B0QYK0;Q01	5;5;4;4;3;1;1	5;5;4;4;3;1;1	4;4;4;4;3;0;0	RNA-binding	EWSR1
E9PAP1;X6R	E9PAP1;X6R	1;1;1;1;1;1;1	1;1;1;1;1;1;1	1;1;1;1;1;1;1	Histone-lysin	SETDB1
P39019;B0ZB	P39019;B0ZB	4;4;4;2;2;2;2	4;4;4;2;2;2;2	4;4;4;2;2;2;2	40S ribosom	RPS19
Q6FHM6;P5	Q6FHM6;P5	3;3;3	3;3;3	3;3;3	NHP2-like pr	NHP2L1
B1AK63;Q9H	B1AK63;Q9H	1;1	1;1	1;1	Chromatin m	MEAF6
B1AKC8;B3K	B1AKC8;B3K	2;2;2	2;2;2	2;2;2	Zinc finger ar	ZBTB40
B1AKN7;B1A	B1AKN7;B1A	9;9;9;9;9;7;5	9;9;9;9;9;7;5	6;6;6;6;6;4;2	Nuclear facto	NFIA
B1ALC2;B5M	B1ALC2;B5M	2;2;1	2;2;1	2;2;1	Zinc finger pr	ZNF618
B1AMU7;B1	B1AMU7;B1	2;2;2;1;1;1	2;2;2;1;1;1	2;2;2;1;1;1	Exosome con	EXOSC1
H0Y757;Q49	H0Y757;Q49	1;1;1;1;1;1	1;1;1;1;1;1	1;1;1;1;1;1	6-phosphofru	PFKP;PFK-P
Q96IR1;Q53	Q96IR1;Q53	13;13;13;13;	13;13;13;13;	13;13;13;13;	40S ribosom	RPS4X
P62899;B2R	P62899;B2R	7;7;6;6;6;6;5	7;7;6;6;6;6;5	7;7;6;6;6;6;5	60S ribosom	RPL31
P06702;B2R	P06702;B2R	1;1	1;1	1;1	Protein S100	S100A9
Q0VAS5;P62	Q0VAS5;P62	6;6;6;2	6;6;6;2	6;6;6;2	Histone H4	HIST1H4H;HI

P62857;B2R4	P62857;B2R4	3;3	3;3	3;3	40S ribosoma	RPS28
Q969Q0;P83	Q969Q0;P83	2;2;2;2;1;1;1	2;2;2;2;1;1;1	2;2;2;2;1;1;1	60S ribosoma	RPL36AL;RPL
G3V4W0;B4D	G3V4W0;B4D	11;11;11;11;	11;11;11;11;	11;11;11;11;	Heterogeneo	HNRNPC
Q9UK43;Q95	Q9UK43;Q95	4;4;4;4;3;3;3	4;4;4;4;3;3;3	4;4;4;4;3;3;3	Protein Red	CSA2;IK
P84103;B2R6	P84103;B2R6	10;10;6	10;10;6	9;9;5	Serine/argini	SRSF3;SFRS3
Q59EG3;H0Y	Q59EG3;H0Y	1;1;1;1;1;1	1;1;1;1;1;1	1;1;1;1;1;1	DNA mismatc	MLH1
Q53FC7;P17(	Q53FC7;P17(	9;9;9;9;7;5	1;1;1;1;0;1	1;1;1;1;0;1	Heat shock 7	HSPA6;HSPA
Q14244;B2R	Q14244;B2R	8;8;7;6;6;5	8;8;7;6;6;5	8;8;7;6;6;5	Ensconsin	MAP7
B4DXX1;Q7Z	B4DXX1;Q7Z	12;12;12	12;12;12	12;12;12	TRMT1-like p	TRMT1L
E5RIZ4;Q9P0	E5RIZ4;Q9P0	1;1;1	1;1;1	1;1;1	39S ribosoma	MRPL15
B4DDX2;Q07	B4DDX2;Q07	6;6;6;5	6;6;6;5	6;6;6;5	KH domain-c	KHDRBS1
Q53HJ4;P252	Q53HJ4;P252	24;24;24;20;	24;24;24;20;	24;24;24;20;	DNA replicati	MCM3;HCC5
Q6FGU7;Q53	Q6FGU7;Q53	2;2;2;2;1;1;1	2;2;2;2;1;1;1	2;2;2;2;1;1;1	Ras-related p	RAB7L1;RAB
Q6IAP9;Q5T1	Q6IAP9;Q5T1	4;4;4;4;4	4;4;4;4;4	4;4;4;4;4	U4/U6 small	PRPF4
O75934;B2R	O75934;B2R	2;2;1	2;2;1	2;2;1	Pre-mRNA-sp	BCAS2
Q13112;B2R	Q13112;B2R	3;3	3;3	3;3	Chromatin as	CHAF1B
F8WBJ6;Q9N	F8WBJ6;Q9N	1;1;1	1;1;1	1;1;1	RNA-binding	PNO1
P53350;B2R8	P53350;B2R8	12;12;9;8;6;6	12;12;9;8;6;6	12;12;9;8;6;6	Serine/threo	PLK1;Plk-1 de
B2R853;B4DI	B2R853;B4DI	34;31	2;2	0;0		
B4DQ46;B2R	B4DQ46;B2R	1;1;1;1;1;1	1;1;1;1;1;1	1;1;1;1;1;1	Cohesin subu	STAG2;DKFZp
Q96C00;B2R	Q96C00;B2R	9;9;3;2	9;9;3;2	9;9;3;2	Zinc finger ar	ZBTB9
E9PPX2;Q53	E9PPX2;Q53	1;1;1;1	1;1;1;1	1;1;1;1	Fos-related a	FOSL1
B7Z7P4;X5CK	B7Z7P4;X5CK	1;1;1;1;1;1	1;1;1;1;1;1	1;1;1;1;1;1	Antigen pept	TAP1
Q96HI0;B2R9	Q96HI0;B2R9	2;2	2;2	2;2	Sentrin-speci	SENP5
Q9UQR1;Q8	Q9UQR1;Q8	8;8;8;6;5;4;2	8;8;8;6;5;4;2	8;8;8;6;5;4;2	Zinc finger pr	ZNF148
Q5TI72;Q5TI	Q5TI72;Q5TI	1;1;1;1;1;1	1;1;1;1;1;1	1;1;1;1;1;1	E-selectin	SELE
H0YNU5;P54	H0YNU5;P54	7;7;7;6;5;3;3	7;7;7;6;5;3;3	7;7;7;6;5;3;3	Bloom syndro	BLM
B2RAX6;Q9Y	B2RAX6;Q9Y	2;2;1	2;2;1	2;2;1	U3 small nuc	UTP18
B2RB99;B4D	B2RB99;B4D	7;7;6;5	7;7;6;5	1;1;0;0		RTFDC1
O95391;B2R	O95391;B2R	7;7;1;1;1	7;7;1;1;1	7;7;1;1;1	Pre-mRNA-sp	SLU7
P33993;B2RE	P33993;B2RE	5;5;4;3;3;1;1	5;5;4;3;3;1;1	5;5;4;3;3;1;1	DNA replicati	MCM7
B3KMF2;Q9Y	B3KMF2;Q9Y	2;2;2	2;2;2	2;2;2	AMME syndr	AMMECR1
Q8WY64;B2F	Q8WY64;B2F	1;1	1;1	1;1	E3 ubiquitin-	MYLIP
Q9H2P0;Q6D	Q9H2P0;Q6D	7;7;7;1	7;7;7;1	7;7;7;1	Activity-depe	ADNP
Q14974;B2R	Q14974;B2R	6;6;4;2;2;2;1	6;6;4;2;2;2;1	6;6;4;2;2;2;1	Importin sub	KPNB1
B2RBV5	B2RBV5	1	1	1		LOC93622
B2RD09;Q63	B2RD09	20;5;3;3;1	20;5;3;3;1	1;0;0;0;0		
I3L2G3;Q6FI(	I3L2G3;Q6FI(	3;3;3;3;3;2;2	3;3;3;3;3;2;2	3;3;3;3;3;2;2	Ketosamine-3	FN3KRP
B3KXD6;B3K	B3KXD6;B3K	4;4;4;4;4;1;1	4;4;4;4;4;1;1	4;4;4;4;4;1;1	Pescadillo ho	PES1
E9PNW8;Q9	E9PNW8;Q9	2;2;2;2	2;2;2;2	2;2;2;2	Fatty acyl-Co	FAR1
Q8N1H4;Q68	Q8N1H4;Q68	9;9;9;9;9;6;3	9;9;9;9;9;6;3	7;7;7;7;7;4;3	Transformer-	DKFZp686F18
Q9BRL5;P621	Q9BRL5;P621	2;2;2;2;2;2;2	2;2;2;2;2;2;2	2;2;2;2;2;2;2	Calmodulin	CALM1;CALM
Q5RKT7;P62	Q5RKT7;P62	8;8;8;6	8;8;8;6	2;2;2;2	Ubiquitin-40	RPS27A
P04844;B2RE	P04844;B2RE	2;2;1;1;1	2;2;1;1;1	2;2;1;1;1	Dolichyl-diph	RPN2
B2RNR6;Q96	B2RNR6;Q96	25;24;12;5;3	25;24;12;5;3	25;24;12;5;3	Zinc finger RI	ZFR

B2RTX8;Q7Z5	B2RTX8;Q7Z5	7;7;4;4	7;7;4;4	7;7;4;4	Wings apart-	WAPAL
B2RWN5;A0A	B2RWN5;A0A	17;15;15;15;	17;15;15;15;	17;15;15;15;	HEAT repeat-	HEATR1
B2RWP5;Q96	B2RWP5;Q96	16;16;13;11;	16;16;13;11;	15;15;12;10;	Histone-lysin	NSD1;DKFZp6
P78364;B2RX	P78364;B2RX	21;21;21;20;	21;21;21;20;	21;21;21;20;	Polyhomeoti	PHC1;DKFZp6
B7Z6D5;B3G	B7Z6D5;B3G	4;4;4;2;1;1;1	4;4;4;2;1;1;1	4;4;4;2;1;1;1	Probable ATF	DDX27
Q5RI15;B3KN	Q5RI15;B3KN	1;1	1;1	1;1	Cytochrome	COX20;FAM3
O95816;B3KI	O95816;B3KI	8;8;8	8;8;8	8;8;8	BAG family m	BAG2
B3KM81;Q59	B3KM81;Q59	5;5;5;5;1	5;5;5;5;1	5;5;5;5;1	DnaJ homolo	DNAJA3
M0R3C3;Q9N	M0R3C3;Q9N	6;6;6;4;1	6;6;6;4;1	6;6;6;4;1	Very-long-ch	TECR
B4DZC3;Q9H	B4DZC3;Q9H	10;10;10;9	10;10;10;9	10;10;10;9	5-3 exoribon	XRN2
Q8N2W9;Q5	Q8N2W9;Q5	6;6;6;1	6;6;6;1	6;6;6;1	E3 SUMO-pro	PIAS4
Q5JTH9;B3KN	Q5JTH9;B3KN	20;20;10;6;3;	20;20;10;6;3;	20;20;10;6;3;	RRP12-like pr	RRP12
B3KMZ7;Q96	B3KMZ7;Q96	15;15;14;1	15;15;14;1	15;15;14;1	Polycomb pro	SCMH1
B4DE78;B4DI	B4DE78;B4DI	3;3;3;3	1;1;1;1	1;1;1;1	14-3-3 protei	YWHAG
Q9Y3T9;B3KI	Q9Y3T9;B3KI	6;6;4;4;1	6;6;4;4;1	6;6;4;4;1	Nucleolar cor	NOC2L
B3KNE7;Q9U	B3KNE7;Q9U	2;2;2;1	2;2;2;1	2;2;2;1	Translocation	SEC63
P61619;B3KN	P61619;B3KN	4;4;4;1;1;1;1;	4;4;4;1;1;1;1;	4;4;4;1;1;1;1;	Protein trans	SEC61A1
B3KNK5;B4D	B3KNK5;B4D	22;20;6;4	1;1;0;0	1;1;0;0		
B3KNN7;B4D	B3KNN7;B4D	2;2;2;2;2;2;2;	2;2;2;2;2;2;2;	2;2;2;2;2;2;2;	26S proteaso	PSMD3
E9PC66;B3KN	E9PC66;B3KN	8;8;8;7;7;1;1;	8;8;8;7;7;1;1;	7;7;7;6;6;1;1;	Zinc finger pr	ZKSCAN1
Q969R5;B3KI	Q969R5;B3KI	1;1	1;1	1;1	Lethal(3)mali	L3MBTL2
B3KSR1;B3KF	B3KSR1;B3KF	1;1;1;1	1;1;1;1	1;1;1;1	Zinc finger pr	ZNF827
Q8N5C6;B3K	Q8N5C6;B3K	14;14;7;7	14;14;7;7	14;14;7;7	S1 RNA-bindi	SRBD1
B3KT06;B3KF	B3KT06;B3KF	20;20;20;19;	3;3;3;3;1;2;2;	0;0;0;0;0;0;0;	Tubulin alpha	TUBA1B
B3KQ51;Q8W	B3KQ51;Q8W	3;3;3;3;3;3;3;	3;3;3;3;3;3;3;	3;3;3;3;3;3;3;	Serine/threo	PPP2CA;PPP2
D1KF47;Q66	D1KF47;Q66	6;6;6;6;5;4;	6;6;6;6;5;4;	6;6;6;6;5;4;	Paired box pr	PAX6
B3KQG6;O95	B3KQG6;O95	3;3;2;1;1	3;3;2;1;1	3;3;2;1;1	Importin-7	IPO7
B4DWS6;Q53	B4DWS6;Q53	1;1;1;1	1;1;1;1	1;1;1;1	Estradiol 17-l	HSD17B12
B3KQX0;Q9N	B3KQX0;Q9N	1;1;1	1;1;1	1;1;1	Zinc finger pr	ZFP64
B3KRJ9;Q69Y	B3KRJ9;Q69Y	3;2;2	3;2;2	3;2;2	Splicing regul	DKFZp667P10
B3KRK2;Q9U	B3KRK2;Q9U	9;9;9;8;1	9;9;9;8;1	9;9;9;8;1	Probable ATF	DDX41
Q86XI2;B3KR	Q86XI2;B3KR	5;5;3;3;3;2;1	5;5;3;3;3;2;1	5;5;3;3;3;2;1	Condensin-2	NCAPG2
F2Z2B9;B3KR	F2Z2B9;B3KR	1;1;1;1	1;1;1;1	1;1;1;1	Gamma-tubu	TUBGCP2
B4DSF4;P170	B4DSF4;P170	3;3;3	3;3;3	3;3;3	Zinc finger pr	ZNF8
B4E0N9;B4DI	B4E0N9;B4DI	3;3;3;3;3;3;2;	3;3;3;3;3;3;2;	3;3;3;3;3;3;2;	Glutamate de	GLUD1;GLUD
Q8N163;B3K	Q8N163;B3K	8;8;4;4;3;3;2;	8;8;4;4;3;3;2;	8;8;4;4;3;3;2;	DBIRD compl	KIAA1967
B4DWZ7;Q9N	B4DWZ7;Q9N	2;2;2	2;2;2	2;2;2	LanC-like pro	LANCL2
H0YHD0;B3K	H0YHD0;B3K	1;1;1;1	1;1;1;1	1;1;1;1	Breast cance	BRMS1L
Q53FG6;Q15	Q53FG6;Q15	1;1;1	1;1;1	1;1;1	Splicing facto	SF3B4
B3KVL5;Q8TE	B3KVL5;Q8TE	1;1	1;1	1;1	Zinc finger CC	ZCCHC10
Q8TF66;B3KV	Q8TF66;B3KV	2;2	2;2	2;2	Leucine-rich	LRRC15
B3KWT5;X5D	B3KWT5;X5D	3;3;3;3;2;2;2;	3;3;3;3;2;2;2;	3;3;3;3;2;2;2;	Cytoplasmic	CYFIP1;DKFZp
F8W6D9;B3K	F8W6D9;B3K	3;3;3;2;2;2;1	3;3;3;2;2;2;1	3;3;3;2;2;2;1	Sentrin-speci	SENP6
E9PD53;B3K	E9PD53;B3K	19;19;19;12;	19;19;19;12;	19;19;19;12;	Structural ma	SMC4
B4DSV5;B3K	B4DSV5;B3K	4;4;4;3;3;2	4;4;4;3;3;2	4;4;4;3;3;2	DNA replicati	MCM2

B4DSH1;Q99	B4DSH1;Q99	17;17;17	17;17;17	17;17;17	Cell division	CDC5L
B4DDB6;B4E	B4DDB6;B4E	18;17;8;1;1;1	1;1;0;0;0;0	1;1;0;0;0;0		HNRPA3
B4DDF4;Q99	B4DDF4;Q99	10;10;10;10;	10;10;10;10;	10;10;10;10;	Calponin-2	CNN2
B4DDG3;Q05	B4DDG3;Q05	1;1	1;1	1;1	Cleavage stin	CSTF1
B7Z5U1;M0R	B7Z5U1;M0R	1;1;1;1	1;1;1;1	1;1;1;1	NAD-depend	SIRT6
B4DE59;C9JT	B4DE59	10;4;4;4;4;1;	4;4;4;4;4;1;1	0;0;0;0;0;0;0		
C9JRJ8;E9PF	C9JRJ8;E9PF	2;2;2;2;2;2;2	2;2;2;2;2;2;2	2;2;2;2;2;2;2	Sequestosom	SQSTM1;SQS
Q658M0;B4D	Q658M0;B4D	3;3;3;3;3;2	3;3;3;3;3;2	3;3;3;3;3;2	Zinc finger pr	DKFZp666L1
U3KQP3;B4D	U3KQP3;B4D	2;2;2;2;2;2	2;2;2;2;2;2	2;2;2;2;2;2	NADH dehyd	NDUFA13;YJ
B4DF70;V9H	B4DF70;V9H	3;3;3;1	2;2;2;1	2;2;2;1	Peroxiredoxi	PRDX2
B4DFE2;Q96	B4DFE2;Q96	13;13;12;9;8;	13;13;12;9;8;	13;13;12;9;8;	PHD finger p	PHF12
F5H669;B4D	F5H669;B4D	2;2;2;1;1;1;1	2;2;2;1;1;1;1	2;2;2;1;1;1;1	Cleavage and	CPSF7
B4DH46;P61	B4DH46;P61	3;3;1	3;3;1	3;3;1	DDB1- and C	DCAF7
C9J0K6;B4DH	C9J0K6;B4DH	1;1;1	1;1;1	1;1;1	Sorcin	SRI
C9JWJ8;D2D	C9JWJ8;D2D	6;6;6;6;5;5;5	4;4;4;4;3;3;3	4;4;4;4;3;3;3	Nuclear facto	NFIX;DKFZp6
Q9H6R0;B4D	Q9H6R0;B4D	10;10;8;3;2	10;10;8;3;2	10;10;8;3;2	Putative ATP	DHX33
B4DJ22;Q9C	B4DJ22;Q9C	1;1;1;1	1;1;1;1	1;1;1;1	INO80 compl	INO80B
K7EMR1;K7E	K7EMR1;K7E	1;1;1;1	1;1;1;1	1;1;1;1	Granulins;Ac	GRN
B4DJK4;B4E0	B4DJK4;B4E0	1;1;1;1	1;1;1;1	1;1;1;1	Transcription	SUPT5H
Q5QPE2;B4D	Q5QPE2;B4D	2;2;2;2;1;1;1	2;2;2;2;1;1;1	1;1;1;1;0;0;0	Protein trans	SEC23B;SEC2
B4DKT9;B4D	B4DKT9;B4D	2;2;2;2;2;1	2;2;2;2;2;1	1;1;1;1;1;1	Catenin alpha	CTNNA1
B4DKY9;Q9H	B4DKY9;Q9H	2;2;2;1;1	2;2;2;1;1	2;2;2;1;1	Sentrin-speci	SENP2
B4DL02;P293	B4DL02;P293	3;3;2;1;1;1;1	3;3;2;1;1;1;1	3;3;2;1;1;1;1	SHC-transfor	SHC1
I3L2Z8;I3L3H	I3L2Z8;I3L3H	1;1;1;1;1;1;1	1;1;1;1;1;1;1	1;1;1;1;1;1;1	Cell division	CDC27
B4DLG2;O75	B4DLG2;O75	10;10;9;6;4;3	10;10;9;6;4;3	10;10;9;6;4;3	Zinc finger C	ZC3H11A
Q96ID3;B4DL	Q96ID3;B4DL	1;1;1	1;1;1	1;1;1	Serine/threo	SAPS1;PPP6R
B4E1M5;E7E	B4E1M5;E7E	4;4;4;4;4;4;3	4;4;4;4;4;4;3	4;4;4;4;4;4;3	60S ribosoma	RPL9
B4DM62;P32	B4DM62;P32	1;1;1	1;1;1	1;1;1	Homeobox p	OTX1
B4DM94;M0	B4DM94;M0	7;7;7;7;7;7;6	7;7;7;7;7;7;6	7;7;7;7;7;7;6	60S ribosoma	RPL18A
B4DMG1;Q9	B4DMG1;Q9	2;2;1;1	2;2;1;1	2;2;1;1	High mobility	HMG20A
Q96RU7;J3K	Q96RU7;J3K	3;3;3;2;1	3;3;3;2;1	3;3;3;2;1	Tribbles hom	TRIB3
F8VQE1;B4D	F8VQE1;B4D	8;8;8;8;6;6;3	8;8;8;8;6;6;3	8;8;8;8;6;6;3	LIM domain a	LIMA1
B4DVK1;B4D	B4DVK1;B4D	3;3;3;3;3;3;3	3;3;3;3;3;3;3	3;3;3;3;3;3;3	Glioma tumo	GLTSCR2
C9J0J7;G5E9	C9J0J7;G5E9	2;2;2;2;2;1;1	2;2;2;2;2;1;1	2;2;2;2;2;1;1	Profilin;Profil	PFN2
B4DNH5;P58	B4DNH5;P58	7;7;1;1;1;1;1	7;7;1;1;1;1;1	5;5;0;0;1;0;0	Zinc finger pr	ZNF121
B4E0I8;B4DN	B4E0I8;B4DN	12;12;12;11;	12;12;12;11;	12;12;12;11;	Ribonucleosi	RRM1
B4DVN1;B4D	B4DVN1;B4D	4;4;4;3;2;2;2	4;4;4;3;2;2;2	4;4;4;3;2;2;2	DnaJ homolo	DNAJB6
B4DPZ5;B4D	B4DPZ5;B4D	5;5;5;4	5;5;5;4	5;5;5;4	Polymerase I	PTRF
B4DP20;J3KN	B4DP20;J3KN	3;3;3;1;1;1	3;3;3;1;1;1	3;3;3;1;1;1	Nuclear pore	NUP88
G3V4N7;B4D	G3V4N7;B4D	1;1;1;1;1	1;1;1;1;1	1;1;1;1;1	Creatine kina	CKB
B4DP61;Q16	B4DP61;Q16	3;3;3	3;3;3	3;3;3	Survival mot	SMN1
B4DQE4;B6Z	B4DQE4;B6Z	2;2;2;2	2;2;2;2	2;2;2;2	Nuclear rece	NR2F6
B4DQG8;Q9N	B4DQG8;Q9N	8;8;6;4;4;3;2	8;8;6;4;4;3;2	8;8;6;4;4;3;2	DNA methylt	DMAP1
F5H8L7;E9PH	F5H8L7;E9PH	1;1;1;1;1	1;1;1;1;1	1;1;1;1;1	Zinc finger pr	ZNF384
B4DRF6;Q4Z	B4DRF6;Q4Z	1;1;1	1;1;1	1;1;1	DNA replicati	MCM6

E9PLN6;O95(	E9PLN6;O95(	2;2;2;2;1;1;1	2;2;2;2;1;1;1	2;2;2;2;1;1;1	Eyes absent I	EYA4
B4DRS4;O43'	B4DRS4;O43'	2;2	2;2	2;2	HIV Tat-speci	HTATSF1
B7ZB05;B4DS	B7ZB05;B4DS	9;9;9;8;1;1	9;9;9;8;1;1	9;9;9;8;1;1	Lysine-specif	KDM2B
Q2F838;B4DS	Q2F838;B4DS	1;1;1;1;1	1;1;1;1;1	1;1;1;1;1	Elongation fa	EEF1G
B4DSR6;Q14.	B4DSR6;Q14.	1;1	1;1	1;1	Cytoplasmic (	DYNC1H1
B4DSU9;F8W	B4DSU9;F8W	3;3;3	3;3;3	3;3;3	Cleavage and	CPSF6
Q59FU3;B4E(	Q59FU3;B4E(	9;9;9;9;9;7;4	4;4;4;4;4;4;2	4;4;4;4;4;4;2	Far upstream	FUBP1
J3KN87;O147	J3KN87;O147	3;3;3;3;1	3;3;3;3;1	3;3;3;3;1	Serine/threo	CHEK1
Q86VM9;E7E	Q86VM9;E7E	18;18;18;8;6;	18;18;18;8;6;	18;18;18;8;6;	Zinc finger C(	ZC3H18
B4DUB8;P13(	B4DUB8;P13(	4;4	4;4	4;4	Zinc finger pr	ZNF35
B4DUT7;Q53	B4DUT7;Q53	7;7;7	7;7;7	7;7;7	GMP synthas	GMPS
B4DV47;Q6P	B4DV47;Q6P	5;5	5;5	5;5	Parafibromin	CDC73
B4DV51;F5H(	B4DV51;F5H(	3;3;3;3;3	3;3;3;3;3	3;3;3;3;3	GTP-binding	RAN
B4DW11;H0Y	B4DW11;H0Y	1;1;1	1;1;1	1;1;1	Clusterin;Clu:	CLU
Q8TAK2;B4D	Q8TAK2;B4D	1;1;1	1;1;1	1;1;1	Catalase	CAT
B4DWW8;E7	B4DWW8;E7	3;3;3;3;1;1;1	3;3;3;3;1;1;1	3;3;3;3;1;1;1	U4/U6 small	PRPF31
K7ELP2;M0Q	K7ELP2;M0Q	1;1;1;1	1;1;1;1	1;1;1;1	Protein lin-37	LIN37
B4DY09;Q12!	B4DY09;Q12!	8;8;8;7;7;4;3	8;8;8;7;7;4;3	8;8;8;7;7;4;3	Interleukin e	ILF2
Q9BTA4;B4D	Q9BTA4;B4D	5;5;5;5;4;4;4;	5;5;5;5;4;4;4;	5;5;5;5;4;4;4;	Cysteine and	CSRP1;DKFZp
Q8IVW5;F8W	Q8IVW5;F8W	1;1;1;1;1;1;1	1;1;1;1;1;1;1	1;1;1;1;1;1;1	Kelch domair	KLHDC3
B4DYP7;Q96(	B4DYP7;Q96(	4;4;3;3;1	4;4;3;3;1	4;4;3;3;1	Gamma-tubu	TUBGCP3
B4DYP9;Q2V	B4DYP9;Q2V	1;1;1	1;1;1	1;1;1	Cell division (	CDC20
Q8NAP3;B4D	Q8NAP3;B4D	2;2;1;1;1;1;1;	2;2;1;1;1;1;1;	2;2;1;1;1;1;1;	Zinc finger ar	ZBTB38;ZBTB
B4DYY5;F8W	B4DYY5;F8W	15;15;15;10;'	15;15;15;10;'	15;15;15;10;'	Ubiquitin-ass	UBAP2L
B7ZAX9;B4D.	B7ZAX9;B4D.	23;23;23;4;4	23;23;23;4;4	12;12;12;4;2	SWI/SNF-rela	SMARCA5
B4DZE5;O00	B4DZE5;O00	6;5;5;4;2	6;5;5;4;2	6;5;5;4;2	DNA-directec	POLRMT
B4DZM3;Q53	B4DZM3;Q53	4;4;4;4;3;1	4;4;4;4;3;1	4;4;4;4;3;1	Ribosomal RI	RRP1
B4DZP5;Q16!	B4DZP5;Q16!	9;9;8;7;7;4;3;	9;9;8;7;7;4;3;	9;9;8;7;7;4;3;	DNA damage	DDB1
B4DZR9;Q9U	B4DZR9;Q9U	8;8;6	8;8;6	8;8;6	Sex comb on	SCML2
Q9BY77;F6VI	Q9BY77;F6VI	11;11;11;10;(	11;11;11;10;(	11;11;11;10;(	Polymerase c	POLDIP3
B4E0S6;O431	B4E0S6;O431	18;18	18;18	18;18	Putative pre-	DHX15
B4E0T1;Q8N	B4E0T1;Q8N	2;2	2;2	2;2	Rho guanine	ARHGEF39
B4E157;X5D!	B4E157;X5D!	1;1;1	1;1;1	1;1;1	Myotubularir	MTMR10
B4E1Z2;H0YE	B4E1Z2	13;3;2;2	1;0;0;0	1;0;0;0		
B4E206;Q8W	B4E206;Q8W	3;3;2;2	3;3;2;2	3;3;2;2	Nuclear pore	NUP133
B4E257;P553	B4E257;P553	2;2;1;1	2;2;1;1	2;2;1;1	Hepatocyte r	FOXA1;FOXA
B4E265;Q8IY	B4E265;Q8IY	2;2	2;2	2;2	Structural ma	SMC5
P08579;B5BT	P08579;B5BT	1;1	1;1	1;1	U2 small nuc	SNRPB2
Q6IT96;Q135	Q6IT96;Q135	7;7;7;6;5;3;3;	3;3;3;2;1;1;2;	3;3;3;2;1;1;2;	Histone deac	HDAC1
Q9Y265;B5BI	Q9Y265;B5BI	17;17;10;9;7;	17;17;10;9;7;	17;17;10;9;7;	RuvB-like 1	RUVBL1
Q6FI27;P498	Q6FI27;P498	2;2;2	2;2;2	2;2;2	Glycogen syn	GSK3B
F5ATB8;E5KC	F5ATB8;E5KC	2;2;2;2;2;2;2;	2;2;2;2;2;2;2;	2;2;2;2;2;2;2;	Glucocorticoi	NR3C1
H7C2Y2;B7Z/	H7C2Y2;B7Z/	1;1;1;1;1;1	1;1;1;1;1;1	1;1;1;1;1;1	GPI transami	PIGT
E5RHY8;E5RI	E5RHY8;E5RI	1;1;1;1;1	1;1;1;1;1	1;1;1;1;1	UPF0544 pro	C5orf45
Q9H6S8;Q6D	Q9H6S8;Q6D	1;1;1;1;1;1	1;1;1;1;1;1	1;1;1;1;1;1	DNA-directec	POLR1B

F5H6U2;F5G	F5H6U2;F5G	2;2;2;2;1	2;2;2;2;1	2;2;2;2;1	Protein FAM	FAM60A
B7Z2E2;Q9U	B7Z2E2;Q9U	3;3;3;1;1;1	3;3;3;1;1;1	3;3;3;1;1;1	Calcium-bind	SLC25A13
C9JM75;B7Z	C9JM75;B7Z	1;1;1;1;1	1;1;1;1;1	1;1;1;1;1	RNA pseudou	RPUSD3
Q6FI30;P086	Q6FI30;P086	6;6;6;1	5;5;5;0	5;5;5;0	Nuclear facto	NFIC
B7Z4V2;V9H	B7Z4V2;V9H	23;23;23;23;	22;22;22;22;	22;22;22;22;	Stress-70 pro	HSPA9
H7C4B7;B7Z	H7C4B7;B7Z	1;1;1;1;1;1;1	1;1;1;1;1;1;1	1;1;1;1;1;1;1	Delta(24)-ste	DHCR24;Nbla
C9J813;Q9N	C9J813;Q9N	4;4;4;4;4;4;3	4;4;4;4;4;4;3	4;4;4;4;4;4;3	Caldesmon	CALD1
B7Z6P1;P681	B7Z6P1;P681	12;12;12;11;	1;1;1;1;1;1;1	1;1;1;1;1;1;1	Actin, alpha s	ACTA1;ACTC
F8W1R7;P60	F8W1R7;P60	5;5;5;5;5;5;4	5;5;5;5;5;5;4	5;5;5;5;5;5;4	Myosin light	MYL6
B7Z7S9;Q56	B7Z7S9;Q56	5;5	5;5	5;5	Shugoshin-lik	SGOL2
H3BND8;B7Z	H3BND8;B7Z	6;6;6;6;6;4;1	6;6;6;6;6;4;1	6;6;6;6;6;4;1	Ubiquitin car	USP7
E7EWK3;B7Z	E7EWK3;B7Z	7;7;7;1;1;1	7;7;7;1;1;1	7;7;7;1;1;1	Probable ATF	DHX36
E9PMS6;B7Z	E9PMS6;B7Z	8;8;7;7;7;7;5	8;8;7;7;7;7;5	8;8;7;7;7;7;5	LIM domain	LMO7
B7Z8Z8;O60	B7Z8Z8;O60	4;4;1;1;1	4;4;1;1;1	4;4;1;1;1	80 kDa MCM	MCM3AP
Q05CP4;Q8I	Q05CP4;Q8I	2;2;2;2;1	2;2;2;2;1	2;2;2;2;1	Interferon-in	EIF2AK2
B7ZKR8;Q86	B7ZKR8;Q86	4;4;4;1;1	4;4;4;1;1	4;4;4;1;1	Ubiquitin car	USP48
H0YLX2;B7ZK	H0YLX2;B7ZK	1;1;1	1;1;1	1;1;1	DNA-binding	RFX7
B7ZL72;E9PL	B7ZL72;E9PL	6;5;4;3	6;5;4;3	6;5;4;3	PR domain zi	PRDM10
Q9UL59;B7Z	Q9UL59;B7Z	2;2	1;1	1;1	Zinc finger pr	ZNF214
Q9NVI1;B7Z	Q9NVI1;B7Z	9;9;6;5;5;3;3	9;9;6;5;5;3;3	9;9;6;5;5;3;3	Fanconi aner	FANCI
Q3L8U1;B7Z	Q3L8U1;B7Z	37;37;31;15;	37;37;31;15;	27;27;21;7;6	Chromodoma	CHD9
B8ZZU8;Q15	B8ZZU8;Q15	2;2;2;1	2;2;2;1	2;2;2;1	Transcription	TCEB2
P11387;B9E	P11387;B9E	7;7;1;1;1;1;1	7;7;1;1;1;1;1	7;7;1;1;1;1;1	DNA topoiso	TOP1
J3QWB6;J3K	J3QWB6;J3K	1;1;1;1	1;1;1;1	1;1;1;1	SWI/SNF-rela	SMARCD2
B9EGR5;Q8I	B9EGR5;Q8I	17;17;14;14;	17;17;14;14;	14;14;14;14;	MAX gene-as	MGA
W6CJ52;Q12	W6CJ52;Q12	4;4;4;2	4;4;4;2	3;3;3;1	Forkhead bo	FOXC1
C8CHS3;Q16	C8CHS3;Q16	1;1	1;1	1;1	Extracellular	ECM1
F8WAU5;C9J	F8WAU5;C9J	2;2;2;1;1;1	2;2;2;1;1;1	2;2;2;1;1;1	Nuclear-inter	ZC3HC1
C9J352;C9JW	C9J352;C9JW	1;1;1;1;1;1;1	1;1;1;1;1;1;1	1;1;1;1;1;1;1	Importin sub	KPNA1
C9J384;D3D	C9J384;D3D	3;3;3;2;1	3;3;3;2;1	3;3;3;2;1	Protein CMS	CMSS1;hCG_
F8WCJ1;C9J7	F8WCJ1;C9J7	1;1;1;1;1;1;1	1;1;1;1;1;1;1	1;1;1;1;1;1;1	Eukaryotic tr	EIF5A2;EIF5A
C9J4Z3;P615	C9J4Z3;P615	2;2;1;1;1;1	2;2;1;1;1;1	2;2;1;1;1;1	60S ribosom	RPL37A
C9J808;Q9BY	C9J808;Q9BY	2;2;1;1	2;2;1;1	2;2;1;1	MKI67 FHA d	MKI67IP
C9J8B4;Q9C	C9J8B4;Q9C	1;1	1;1	1;1	pre-mRNA 3	WDR33
Q14061;C9J8	Q14061;C9J8	2;2;1	2;2;1	2;2;1	Cytochrome	COX17
C9JA28;Q9U	C9JA28;Q9U	1;1	1;1	1;1	Translocon-a	SSR3
C9JA69;Q8W	C9JA69;Q8W	21;21;20;16;	21;21;20;16;	21;21;20;16;	HMG box tra	BBX
C9JCD9;C9JN	C9JCD9;C9JN	1;1;1	1;1;1	1;1;1	Protein BUD	BUD31
C9JFR7;P999	C9JFR7;P999	2;2;2;1;1	2;2;2;1;1	2;2;2;1;1	Cytochrome	CYCS
C9JL85;Q69Y	C9JL85;Q69Y	1;1;1	1;1;1	1;1;1	Myotrophin	MTPN;DKFZp
C9JXB8;C9JN	C9JXB8;C9JN	5;5;5;5	5;5;5;5	5;5;5;5	60S ribosom	RPL24
C9JW69;Q5T	C9JW69;Q5T	5;5;5;4;4;4;4	5;5;5;4;4;4;4	5;5;5;4;4;4;4	Regulator of	RCC1
C9JYM0;O75	C9JYM0;O75	1;1	1;1	1;1	Ribonuclease	POP7
C9JZI1;P3524	C9JZI1;P3524	10;10;9;9;7;5	10;10;9;9;7;5	10;10;9;9;7;5	Replication f	RFC4
Q96FS1;Q68	Q96FS1;Q68	5;5;5;5;3;3;3	5;5;5;5;3;3;3	5;5;5;5;3;3;3	Catenin delta	CTNND1;DKF

CON__A2A5Y	CON__A2A5Y	15	5	1		
CON__A2AB7	CON__A2AB7	3	2	2		
CON__ENSEM	CON__ENSEM	3;2;2;2;2;2;2;	3;2;2;2;2;2;2;	3;2;2;2;2;2;2;	Complement	C4B;C4A
CON__ENSEM	CON__ENSEM	12	2	1		
O76013;CON	O76013;CON	11;11	3;3	3;3	Keratin, type	KRT36
O95678;CON	O95678;CON	15;15	1;1	1;1	Keratin, type	KRT75
CON__P0076	CON__P0076	10	10	10		
P02533;CON_	P02533;CON_	31;31;20;10;9	27;27;20;9;9;	12;12;8;4;2;0	Keratin, type	KRT14
CON__P0253	CON__P0253	10	1	1		
CON__P0266	CON__P0266	9	9	9		
CON__P0266	CON__P0266	6	6	6		
CON__P0266	CON__P0266	4	4	4		
CON__P0266	CON__P0266	6	6	6		
CON__P0275	CON__P0275	7	7	7		
CON__P0276	CON__P0276	83;6;6;6;6;6;6	83;6;6;6;6;6;6	83;6;6;6;6;6;6;6;6;5;5;5;4;4;1		
CON__P0277	CON__P0277	1	1	1		
CON__P0425	CON__P0425	36;29	25;21	0;0		
P05787;CON_	P05787;CON_	20;20;16;14;1	11;11;10;7;6;	11;11;10;7;6;	Keratin, type	KRT8
CON__P0686	CON__P0686	44;6;3;3;1;1	44;6;3;3;1;1	44;6;3;3;1;1		
P08779;CON_	P08779;CON_	30;30;12;11;1	17;17;10;4;4;	17;17;10;4;4;	Keratin, type	KRT16
P13645;CON_	P13645;CON_	38;38;4;4;3;2	38;38;4;4;3;2	26;26;1;1;0;0	Keratin, type	KRT10
P13647;CON_	P13647;CON_	36;36;14;6	19;19;2;6	2;2;0;2	Keratin, type	KRT5
CON__P1769	CON__P1769	1	1	1		
O75370;I0B0	O75370;I0B0	1;1;1;1;1;1;1	1;1;1;1;1;1;1	1;1;1;1;1;1;1	Filaggrin	FLG
P35527;CON_	P35527;CON_	32;32;20	32;32;20	32;32;20	Keratin, type	KRT9
CON__P3590	CON__P3590	51;3;2;1;1	1;0;0;0;0	1;0;0;0;0		
P78386;CON_	P78386;CON_	22;22;11;10	5;5;2;2	3;3;1;1	Keratin, type	KRT85
Q04695;CON	Q04695;CON	22;22;18;17;1	10;10;6;6;4;5	7;7;4;4;3;4;4;	Keratin, type	KRT17;JUP
Q15323;CON	Q15323;CON	16;16;16	15;15;15	0;0;0	Keratin, type	KRT31
CON__Q1RM	CON__Q1RM	6	6	6		
CON__Q1RM	CON__Q1RM	1	1	1		
CON__Q2UV	CON__Q2UV	1	1	1		
Q86Y46;CON	Q86Y46;CON	8;8;6;6;6;4;4;	3;3;1;1;1;1;1;	1;1;0;0;0;0;0;	Keratin, type	KRT73;KRT74
P08729;CON_	P08729;CON_	14;14;13;11;7	9;9;8;6;2;3;1;	7;7;6;4;1;2;0;	Keratin, type	KRT7
CON__Q3SX0	CON__Q3SX0	1	1	1		
CON__Q497I	CON__Q497I	15	3	1		
Q5D862;CON	Q5D862;CON	6;6	6;6	6;6	Filaggrin-2	FLG2
Q5XKE5;CON	Q5XKE5;CON	11;11	1;1	0;0	Keratin, type	KRT79
CON__Q5XQ	CON__Q5XQ	31;24	1;1	0;0		
CON__Q6172	CON__Q6172	20	4	3		
Q6A162;CON	Q6A162;CON	4;4;3;3;2	1;1;1;1;1	1;1;1;1;1	Keratin, type	KRT40
Q6KB66;CON	Q6KB66;CON	3;3;1	3;3;1	3;3;1	Keratin, type	KRT80
P78385;CON_	P78385;CON_	24;24;22;22	23;23;21;21	1;1;0;0	Keratin, type	KRT83
Q8N1N4;CON	Q8N1N4;CON	8;8;8;4;1	5;5;5;2;1	5;5;5;2;1	Keratin, type	KRT78
Q7Z3Z0;CON_	Q7Z3Z0;CON_	3;3;3;3	1;1;1;1	1;1;1;1	Keratin, type	KRT25;KRT27

Q7Z794;Q0III	Q7Z794;Q0III	13;13;13;7;3	9;9;9;6;2	6;6;6;3;2	Keratin, type	KRT77;KRT1E
Q86YZ3;CON	Q86YZ3;CON	11;11	11;11	11;11	Hornerin	HRNR
Q9BYT5;F5H:	Q9BYT5;F5H:	3;3;3;3;3;3;3	3;3;3;3;3;3;3	3;3;3;3;3;3;3	Keratin-assoc	KRTAP2-2;KR
Q9NSB4;Q70	Q9NSB4;Q70	6;6;6	2;2;2	2;2;2	Keratin, type	KRT82;KRTHE
CON__Q9U6'	CON__Q9U6'	8	8	8		
CON__REFSE	CON__REFSE	1	1	1		
CON__REFSE	CON__REFSE	15	2	2		
Q8N4P8;Q9B	Q8N4P8;Q9B	14;14;14;13;:	14;14;14;13;:	14;14;14;13;:	Nucleolar GT	GTPBP4
Q6NX58;D3D	Q6NX58;D3D	9;9;9;5;5;1;1	9;9;9;5;5;1;1	9;9;9;5;5;1;1	MAP7 domai	MAP7D1;RPF
D3DQF6;Q9P	D3DQF6;Q9P	3;3;2;1	3;3;2;1	3;3;2;1	RNA-binding	POU4F3;RBM
Q8N3X1;D3D	Q8N3X1;D3D	1;1	1;1	1;1	Formin-bindi	FNBP4
D3DS96;Q9N	D3DS96;Q9N	5;5;4;1;1;1;1	5;5;4;1;1;1;1	5;5;4;1;1;1;1	Bromodomai	BAZ1A
D3DT44;P48!	D3DT44;P48!	1;1	1;1	1;1	Glutamate--c	GCLM
H3BV80;H3B	H3BV80;H3B	3;3;3;3;3;2;2;	3;3;3;3;3;2;2;	3;3;3;3;3;2;2;	RNA-binding	RNPS1
K7EMM8;Q4!	K7EMM8;Q4!	5;5;5;4;1;1	5;5;5;4;1;1	5;5;5;4;1;1	Putative oxid	GLYR1;N-PAC
D3DVB3;Q6P	D3DVB3;Q6P	2;2;1;1	2;2;1;1	2;2;1;1	Polyamine-m	hCG_199935
D3DWL9;Q1(	D3DWL9;Q1(	3;3;1;1	3;3;1;1	3;3;1;1	Cleavage and	CPSF1
D6R9W4;Q1(	D6R9W4;Q1(	4;4;2;2;1;1;1	4;4;2;2;1;1;1	4;4;2;2;1;1;1	Drebrin	DBN1
D6RFI4;X5D9	D6RFI4;X5D9	2;2;2;2;1;1;1;	2;2;2;2;1;1;1;	2;2;2;2;1;1;1;	Pituitary hon	PITX2;PITX1;I
Q9UL40;D6R.	Q9UL40;D6R.	2;2;1;1;1;1	2;2;1;1;1;1	2;2;1;1;1;1	Zinc finger pr	ZNF346
Q8N720;D6V	Q8N720;D6V	5;5;4;4;1	5;5;4;4;1	5;5;4;4;1	Zinc finger pr	ZNF655
Q2TAK8;J3KN	Q2TAK8;J3KN	4;4;4;3;1;1	4;4;4;3;1;1	4;4;4;3;1;1	PWWP doma	MUM1
Q13111;D6W	Q13111;D6W	4;4;2;1	4;4;2;1	4;4;2;1	Chromatin as	CHAF1A
E1NZA1;A0A(	E1NZA1;A0A(	6;5;5;1	6;5;5;1	6;5;5;1	Translational	PRIC295;GCN
E5KLK2;E5KL	E5KLK2;E5KL	20;20;20;20;:	20;20;20;20;:	20;20;20;20;:	Dynamin-like	OPA1
P78549;E5KT	P78549;E5KT	7;7;4;3;1;1	7;7;4;3;1;1	7;7;4;3;1;1	Endonuclease	NTHL1
E5RFZ8;E5RG	E5RFZ8;E5RG	1;1;1;1;1;1	1;1;1;1;1;1	1;1;1;1;1;1	Zinc transpor	SLC39A14
P63208;E5RJ	P63208;E5RJ	5;5;1;1;1;1;1;	5;5;1;1;1;1;1;	5;5;1;1;1;1;1;	S-phase kinas	SKP1
P15090;E7D\	P15090;E7D\	1;1	1;1	1;1	Fatty acid-bir	FABP4
Q01469;E7D'	Q01469;E7D'	2;2	2;2	2;2	Fatty acid-bir	FABP5
E7EN86;P527	E7EN86;P527	3;3;2;1;1;1;1;	3;3;2;1;1;1;1;	3;3;2;1;1;1;1;	Zinc finger pr	ZNF143
E7ESY4;Q133	E7ESY4;Q133	7;7;6;6;5;5;5;	4;4;3;3;2;2;2;	1;1;0;1;0;0;0;	Metastasis-as	MTA1;MTA3;
E7ET15;O15(	E7ET15;O15(	8;8;4;4;4;2;1;	8;8;4;4;4;2;1;	8;8;4;4;4;2;1;	U2 snRNP-as:	U2SURP
E7ETY2;J3KQ	E7ETY2;J3KQ	17;16;9;6;5;3	1;0;0;0;0;0	1;0;0;0;0;0		TCOF1
Q2I0Y7;E7EU	Q2I0Y7;E7EU	7;7;7;7;6;6;6;	7;7;7;7;6;6;6;	7;7;7;7;6;6;6;	Casein kinase	CSNK2A1;CSI
P27816;E7EV	P27816;E7EV	3;3;2;2;2;2;2;	3;3;2;2;2;2;2;	3;3;2;2;2;2;2;	Microtubule-	MAP4
E9PC90;H1UI	E9PC90;H1UI	2;2;2;1	2;2;2;1	2;2;2;1	G2/mitotic-sp	CCNB1;CCNB
Q75MX6;Q9(	Q75MX6;Q9(	6;6;6;6;1	6;6;6;6;1	6;6;6;6;1	General trans	GTF2IRD1
E9PIE4;Q9Y6	E9PIE4;Q9Y6	4;4;3	4;4;3	4;4;3	Mitochondria	MTCH2
G3V1E0;E9PJ	G3V1E0;E9PJ	1;1;1;1	1;1;1;1	1;1;1;1	F-box only pr	FBXO3
E9PKG1;Q5U	E9PKG1;Q5U	4;4;4;4;3;1;1;	4;4;4;4;3;1;1;	4;4;4;4;3;1;1;	Protein argin	PRMT1;HRM
F8VSA6;E9PS	F8VSA6;E9PS	1;1;1;1;1	1;1;1;1;1	1;1;1;1;1	NEDD8	NEDD8;NEDD
Q9BQQ5;E9P	Q9BQQ5;E9P	5;5;5;5;4;1	5;5;5;5;4;1	5;5;5;5;4;1	60S ribosoma	L27a;RPL27A
E9PSI3;E9PR:	E9PSI3;E9PR:	2;2;2;2	2;2;2;2	2;2;2;2	Nuclear pore	NUP160
P62861;E9PR	P62861;E9PR	1;1	1;1	1;1	40S ribosoma	FAU

P24468;F1D8	P24468;F1D8	7;7	7;7	3;3	COUP transc	NR2F2
F1DAL7;F1DA	F1DAL7;F1DA	5;5;5;5;5	1;1;1;1;1	1;1;1;1;1	COUP transc	NR2F1
F1JVV5;Q53F	F1JVV5;Q53F	2;1;1;1;1;1;1	1;1;1;1;1;1;1	1;1;1;1;1;1;1	Cyclic AMP-d	EWSR1/ATF1
Q8N8A6;F1T0	Q8N8A6;F1T0	11;11	11;11	11;11	ATP-depende	DDX51
F2Z2U4;Q9Y4	F2Z2U4;Q9Y4	9;9;8;8;1	9;9;8;8;1	9;9;8;8;1	Transformati	TRRAP
F2Z2W6;F2Z2	F2Z2W6;F2Z2	1;1;1;1	1;1;1;1	1;1;1;1	Non-histone	HMGN1
I3L521;F5GX	I3L521;F5GX	2;2;2;2;2;2;2	2;2;2;2;2;2;2	2;2;2;2;2;2;2	RNA-binding	RBM7
F5GZS6;P081	F5GZS6;P081	18;18;18;17;1	18;18;18;17;1	18;18;18;17;1	4F2 cell-surfa	SLC3A2
P29375;F5H1	P29375;F5H1	7;7;4;3;3	7;7;4;3;3	7;7;4;3;3	Lysine-specif	KDM5A
F5H3Y4;Q8IY	F5H3Y4;Q8IY	4;4	4;4	4;4	Probable ATF	DHX37
F8VRX9;F5H4	F8VRX9;F5H4	1;1;1	1;1;1	1;1;1	Protein asun	ASUN
Q9BQE3;Q53	Q9BQE3;Q53	19;19;19;18;1	3;3;3;3;3	2;2;2;2;2	Tubulin alpha	TUBA1C
F6RFD5;V9HV	F6RFD5;V9HV	1;1;1	1;1;1	1;1;1	Destrin	DSTN
F8VPD4;P277	F8VPD4;P277	3;3;2;1;1	3;3;2;1;1	3;3;2;1;1	CAD protein;	CAD
J3QK86;J3KP	J3QK86;J3KP	2;2;2;2;1;1	2;2;2;2;1;1	2;2;2;2;1;1	Bromodomai	BAZ2A
F8VW34;F8V	F8VW34;F8V	1;1;1;1;1;1;1	1;1;1;1;1;1;1	1;1;1;1;1;1;1	Homeobox p	TGIF1
F8VXG7;Q99	F8VXG7;Q99	30;30;29;14;1	30;30;29;14;1	30;30;29;14;1	Protein SCAF	SCAF11
H3BRU6;F8V	H3BRU6;F8V	8;8;8;7;6;5;5	4;4;4;4;3;2;3	4;4;4;4;3;2;3	Poly(rC)-bind	PCBP2;PCBP3
F8W038;Q8I	F8W038;Q8I	1;1	1;1	1;1	Chromatin cc	C17orf49;BAI
F8W0Q9;Q8N	F8W0Q9;Q8N	12;11;9;7;7;7	12;11;9;7;7;7	12;11;9;7;7;7	Periphilin-1	PPHLN1
F8WAJ0;B4D	F8WAJ0;B4D	9;8;8	9;8;8	9;8;8	Probable ATF	DDX31
F8WEI3;F8W	F8WEI3;F8W	1;1;1;1;1	1;1;1;1;1	1;1;1;1;1	Probable ATF	DDX56
Q29RF7;G1U	Q29RF7;G1U	9;9;5;5;1	9;9;5;5;1	9;9;5;5;1	Sister chroma	PDS5A
G3V256;Q6P	G3V256;Q6P	6;6;5;5;5;2;2	6;6;5;5;5;2;2	6;6;5;5;5;2;2	Zinc finger C	ZC3H14
G3V2N2;Q9U	G3V2N2;Q9U	1;1	1;1	1;1	Homeobox p	SIX4
G3V4E7;G3V	G3V4E7;G3V	1;1;1;1;1	1;1;1;1;1	1;1;1;1;1	UPF0568 pro	C14orf166
G3V529;Q9G	G3V529;Q9G	22;22;20;19;1	22;22;20;19;1	22;22;20;19;1	ATP-depende	DDX24
G3V5L1;Q8T	G3V5L1;Q8T	4;4;4;2;2;2;2	4;4;4;2;2;2;2	4;4;4;2;2;2;2	Protein max	MAX
G5E9G6;B4D	G5E9G6;B4D	7;6;5;4;4;2;2	1;1;1;0;0;0;0	1;1;1;0;0;0;0		TRIP12
Q8NBL6;G5E	Q8NBL6;G5E	3;3;3;3;2;2;2	3;3;3;3;2;2;2	3;3;3;3;2;2;2	28S ribosoma	MRPS22
P31943;G8JL	P31943;G8JL	20;20;19;12;1	20;20;19;12;1	13;13;12;8;5;	Heterogeneo	HNRNPH1
V9HW98;P62	V9HW98;P62	3;3;3;3;2;2	1;1;1;1;1;1	1;1;1;1;1;1	14-3-3 protei	YWHAE;YWH
V9GY48;H0U	V9GY48;H0U	3;3;3;3	3;3;3;3	3;3;3;3	Zinc finger C	ZGPAT
H0Y2W2;Q96	H0Y2W2;Q96	11;7;6;6;5;4;4	11;7;6;6;5;4;4	1;1;0;1;0;0;0	ATPase famil	ATAD3A;ATA
H0Y4R1;P122	H0Y4R1;P122	4;4;2;1;1;1	4;4;2;1;1;1	4;4;2;1;1;1	Inosine-5-mc	IMPDH2
Q96EY4;H0Y9	Q96EY4;H0Y9	6;6;5;3;2;2	6;6;5;3;2;2	6;6;5;3;2;2	Translation n	TMA16
H0Y9Z8;Q9BI	H0Y9Z8;Q9BI	1;1;1	1;1;1	1;1;1	Condensin cc	NCAPG
P28347;H0YE	P28347;H0YE	3;3;2;2;2;2;2	3;3;2;2;2;2;2	3;3;2;2;2;2;2	Transcription	TEAD1;TEAD4
H0YEN5;P158	H0YEN5;P158	12;12;11;11;1	12;12;11;11;1	12;12;11;11;1	40S ribosoma	RPS2;rps2
H0YIN9	H0YIN9	10	1	1		KRT5
H0YJS3;Q8IYI	H0YJS3;Q8IYI	1;1	1;1	1;1	Fanconi aner	FANCM
P46779;H0YK	P46779;H0YK	7;7;6;5;5;1	7;7;6;5;5;1	7;7;6;5;5;1	60S ribosoma	RPL28
Q15004;H0YI	Q15004;H0YI	2;2;1;1	2;2;1;1	2;2;1;1	PCNA-associa	KIAA0101
Q6JHV3;H0YI	Q6JHV3;H0YI	11;11;11;10;9	11;11;11;10;9	11;11;11;10;9	Ubiquitin car	USP3
Q92841;H3BI	Q92841;H3BI	30;30;30;20;1	22;22;22;14;1	22;22;22;14;1	Probable ATF	DDX17

Q9UPN3;H3E	Q9UPN3;H3E	1;1;1	1;1;1	1;1;1	Microtubule-	MACF1
H3BPL5;Q96I	H3BPL5;Q96I	1;1	1;1	1;1	SAGA-associa	CCDC101
H3BR04;H3B	H3BR04;H3B	2;2;2;2;2;2;1;	2;2;2;2;2;2;1;	2;2;2;2;2;2;1;	Fructose-bisp	ALDOA
H3BU53;B4D	H3BU53;B4D	4;3	1;1	1;1		MGA
H3BUJ7;Q66I	H3BUJ7;Q66I	6;6;4;2;1;1;1	6;6;4;2;1;1;1	6;6;4;2;1;1;1	Transcription	E4F1
P04264;H6VF	P04264;H6VF	47;47;47;47;4	44;44;44;44;4	0;0;0;0;0;0;0	Keratin, type	KRT1
H7BXI1;A0FG	H7BXI1;A0FG	2;1;1	2;1;1	2;1;1	Extended syr	ESYT2
H7BYN4;Q02	H7BYN4;Q02	11;11;3;3;1	11;11;3;3;1	11;11;3;3;1	Kinesin-like p	KIF23
P48730;H7BY	P48730;H7BY	6;6;5;5;5;5;1	6;6;5;5;5;5;1	2;2;2;2;2;2;1	Casein kinase	CSNK1D
H7C0N4;L0R8	H7C0N4;L0R8	6;5	2;2	2;2		SF1
H7C1M2	H7C1M2	22	2	0		SON
Q99848;Q6IE	Q99848;Q6IE	10;10;10;5;4	10;10;10;5;4	10;10;10;5;4	Probable rRN	EBNA1BP2
Q7Z4Y3;H7C3	Q7Z4Y3;H7C3	1;1;1	1;1;1	1;1;1	Serrate RNA e	SRRT
H7C3P6;P529	H7C3P6;P529	4;4;3	4;4;3	4;4;3	Nuclear pore	NUP98
H7C561	H7C561	6	3	0		SF1
Q6W2J9;H9A	Q6W2J9;H9A	20;20;19;15;8	20;20;19;15;8	19;19;18;15;7	BCL-6 corepr	BCOR;BCOR-I
P10599;H9ZY	P10599;H9ZY	5;5	5;5	5;5	Thioredoxin	TXN
I3L0U2;I3L3C	I3L0U2;I3L3C	1;1;1	1;1;1	1;1;1	Testisin	PRSS21
I3L1P8;Q6IBH	I3L1P8;Q6IBH	3;3;3	3;3;3	3;3;3	Mitochondria	SLC25A11
Q8WUZ0;I3L1	Q8WUZ0;I3L1	1;1	1;1	1;1	B-cell CLL/lyr	BCL7C
I3L1Q5;Q2NL	I3L1Q5;Q2NL	1;1	1;1	1;1	Pre-rRNA-pro	TSR1
Q96CP2;I3L1	Q96CP2;I3L1	4;4;3	4;4;3	3;3;2	FLYWCH fam	FLYWCH2
I3L2A4;Q9NR	I3L2A4;Q9NR	1;1	1;1	1;1	NAD-depend	SIRT7
I3L2Z5;Q8IUI	I3L2Z5;Q8IUI	9;9;9;8;7;6;5;	8;8;8;7;6;6;5;	8;8;8;7;6;6;5;	Myc-associat	MAZ
I3VM54;Q9Y2	I3VM54;Q9Y2	11;11;11;3	11;11;11;3	11;11;11;3	Lysine-specif	KDM2A
P14174;I4AY8	P14174;I4AY8	1;1	1;1	1;1	Macrophage	MIF
I6ZVX6;Q999	I6ZVX6;Q999	2;2;1;1	1;1;0;0	1;1;0;0	Forkhead bo	FOXC2;FOXS1
J3KMX5;P622	J3KMX5;P622	7;7;3	7;7;3	7;7;3	40S ribosoma	RPS13
O43248;J3KN	O43248;J3KN	1;1	1;1	1;1	Homeobox p	HOXC11
Q9UKL0;J3KN	Q9UKL0;J3KN	2;2	2;2	2;2	REST corepre	RCOR1
J3KRG2;Q96Q	J3KRG2;Q96Q	1;1	1;1	1;1	Gasdermin-A	GSDMA
J3KSZ8;Q68D	J3KSZ8;Q68D	9;9;7;6;2;2;1	9;9;7;6;2;2;1	9;9;7;6;2;2;1	Male-specific	MSL1
P17844;J3KTA	P17844;J3KTA	37;37;36;35;3	37;37;36;35;3	29;29;28;27;2	Probable ATP	DDX5;DKFZp6
Q8IWR8;J3QI	Q8IWR8;J3QI	3;3;3;3;3;1	3;3;3;3;3;1	3;3;3;3;3;1	Ribosomal pr	RPL19
Q07955;J3KT	Q07955;J3KT	13;13;12;9;6;	13;13;12;9;6;	12;12;11;8;6;	Serine/argini	SRSF1
Q8IWX8;J3QI	Q8IWX8;J3QI	4;4;3;3;3	4;4;3;3;3	4;4;3;3;3	Calcium hom	CHERP
J3QL54;Q9BV	J3QL54;Q9BV	2;2;1;1;1;1;1;	2;2;1;1;1;1;1;	2;2;1;1;1;1;1;	Nuclear pore	NUP85
Q96MU7;J3C	Q96MU7;J3C	3;3;1	3;3;1	3;3;1	YTH domain-	YTHDC1
O43670;J3QF	O43670;J3QF	9;9;9;8;4;4;4;	9;9;9;8;4;4;4;	9;9;9;8;4;4;4;	Zinc finger pr	ZNF207
J3QSH4;Q141	J3QSH4;Q141	11;11;9	11;11;9	10;10;9	Vascular end	VEZF1
Q03252;J9JID	Q03252;J9JID	9;9;4	5;5;2	5;5;2	Lamin-B2	LMNB2
K7EID2;Q9BT	K7EID2;Q9BT	2;2	2;2	2;2	Chitobiosyldi	ALG1
K7EJH0;K7EN	K7EJH0;K7EN	7;7;6;6;6;5;2;	7;7;6;6;6;5;2;	7;7;6;6;6;5;2;	Kinetochore	SPC24
K7EQ55;K7EK	K7EQ55;K7EK	5;5;5;4;4;1	5;5;5;4;4;1	5;5;5;4;4;1	DAZ-associat	DAZAP1;DAZA
K7ELW0;V9H	K7ELW0;V9H	4;4;4;2	4;4;4;2	4;4;4;2	Protein DJ-1	PARK7

K7EMZ9;Q8N	K7EMZ9;Q8N	1;1	1;1	1;1	Protein LSM1	LSM14A
K7EQ03;K7EF	K7EQ03;K7EF	1;1;1	1;1;1	1;1;1	RNA-binding	RBM42
K7EPJ1;Q167	K7EPJ1;Q167	2;2;1;1	2;2;1;1	2;2;1;1	Ubiquitin-cor	UBE2S
K7ERD7;O95	K7ERD7;O95	3;3;2;2;1	3;3;2;2;1	3;3;2;2;1	Ribonuclease	POP4
K7ESE6;Q9BU	K7ESE6;Q9BU	1;1	1;1	1;1	Glucose-6-ph	G6PC3
Q2VPJ6;Q8TE	Q2VPJ6;Q8TE	2;2;2;2;1;1;1;	2;2;2;2;1;1;1;	1;1;1;1;0;0;0;	Heat shock p	HSP90AA1;EE
L7N2F3;W4V	L7N2F3;W4V	2;2;2;2;1	2;2;2;2;1	2;2;2;2;1	UPF0711 pro	C18orf21
M0QXA7;B9E	M0QXA7;B9E	37;34;28;22	37;34;28;22	37;34;28;22	Protein Wiz	WIZ
M0QXM4;Q7	M0QXM4;Q7	2;2;2;1;1	2;2;2;1;1	2;2;2;1;1	Neutral amin	SLC1A5
M0R050;Q9N	M0R050;Q9N	2;2;1	2;2;1	2;2;1	Exosome con	EXOSC5
M0R301	M0R301	1	1	1		TIMM44
Q9HCK1;N0D	Q9HCK1;N0D	1;1	1;1	1;1	DBF4-type zi	ZDBF2
P67870;N0E4	P67870;N0E4	7;7;7;6;5;5;5;	7;7;7;6;5;5;5;	7;7;7;6;5;5;5;	Casein kinase	CSNK2B;CSNI
O00165;E9PI	O00165;E9PI	5;3;2	5;3;2	5;3;2	HCLS1-associ	HAX1
O00257;F8W	O00257	25;3;3	25;3;3	25;3;3	E3 SUMO-pro	CBX4
Q6IB11;O002	Q6IB11;O002	1;1	1;1	1;1	Membrane-a	PGRMC1
O14646;H0Y	O14646	11;1;1;1	10;1;1;1	10;1;1;1	Chromodoma	CHD1
O14686;F8V	O14686;F8V	2;1	2;1	2;1	Histone-lysin	KMT2D
O14776;G3V	O14776;G3V	16;11;3	16;11;3	16;11;3	Transcription	TCERG1
O14965;A3KI	O14965;A3KI	16;14;13;12;	16;14;13;12;	16;14;13;12;	Aurora kinase	AURKA
O14980;B3K	O14980;B3K	7;4;4;4;2;2;1;	7;4;4;4;2;2;1;	7;4;4;4;2;2;1;	Exportin-1	XPO1
Q5T942;O15	Q5T942;O15	7;7	7;7	7;7	Zinc finger ar	ZBTB5
Q96HT3;O15	Q96HT3;O15	3;3;2;2;2;1	3;3;2;2;2;1	3;3;2;2;2;1	DNA-directed	POLR1C
O15294;Q54	O15294;Q54	13;10;3;2;2	13;10;3;2;2	13;10;3;2;2	UDP-N-acety	OGT
O15379	O15379	1	1	1	Histone deac	HDAC3
O43159;E9PF	O43159;E9PF	10;7;1	10;7;1	10;7;1	Ribosomal RI	RRP8
O43175;Q5S	O43175;Q5S	2;1;1;1	2;1;1;1	2;1;1;1	D-3-phospho	PHGDH
O43290;B4DI	O43290;B4DI	9;8;2;1;1	9;8;2;1;1	9;8;2;1;1	U4/U6.U5 tri	SART1
O43390;Q0V	O43390;Q0V	19;18;18;17;	19;18;18;17;	15;14;14;14;	Heterogeneo	HNRNPR;HNI
O43474;L0R	O43474;L0R	2;1;1;1	2;1;1;1	2;1;1;1	Krueppel-like	KLF4
O43660;B7Z	O43660;B7Z	4;3;3	4;3;3	4;3;3	Pleiotropic re	PLRG1
O43684;J3Q1	O43684;J3Q1	12;11;9;5	12;11;9;5	12;11;9;5	Mitotic check	BUB3
O43823;Q8N	O43823;Q8N	19;18;12;7;5	19;18;12;7;5	19;18;12;7;5	A-kinase ancl	AKAP8;DKFZ
O60287	O60287	5	5	5	Nucleolar pre	URB1
P23490;Q6FI	P23490;Q6FI	1;1;1	1;1;1	1;1;1	Loricrin	LOR
Q05CK9;O60	Q05CK9;O60	16;16;15;15;	12;12;11;11;	12;12;11;11;	Heterogeneo	SYNCRIP
O60832;C9IY	O60832;C9IY	12;8;7;6;6;5;	12;8;7;6;6;5;	12;8;7;6;6;5;	H/ACA ribonu	DKC1
O75132	O75132	5	5	5	Zinc finger BE	ZBED4
O75182;M0C	O75182	9;4;2;2	9;4;2;2	9;4;2;2	Paired amphi	SIN3B
O75319	O75319	4	4	4	RNA/RNP cor	DUSP11
Q53FC3;O75	Q53FC3;O75	3;3;2;1;1	3;3;2;1;1	3;3;2;1;1	Programmed	PDCD6
O75367;Q59	O75367;Q59	4;2;2;1	4;2;2;1	4;2;2;1	Core histone	H2AFY
O75400;B4DI	O75400;B4DI	7;5;3;3;2;2;1	7;5;3;3;2;2;1	7;5;3;3;2;2;1	Pre-mRNA-pr	PRPF40A
O75446	O75446	3	3	3	Histone deac	SAP30
O75475;Q05	O75475;Q05	9;8;2	9;8;2	8;7;1	PC4 and SFRS	PSIP1

Q5JRI1;Q53G	Q5JRI1;Q53G	4;4;4;2;2;1	4;4;4;2;2;1	4;4;4;2;2;1	Serine/argini	SRSF10;FUSIF
O75533;A0JL	O75533;A0JL	14;8;8;8;8;2;:	14;8;8;8;8;2;:	14;8;8;8;8;2;:	Splicing facto	SF3B1
O75607	O75607	3	3	3	Nucleoplasm	NPM3
O75643;A4FU	O75643;A4FU	13;11;5;2;2;2	13;11;5;2;2;2	13;11;5;2;2;2	U5 small nuc	SNRNP200
O75691;B4DI	O75691	15;4	15;4	15;4	Small subunit	UTP20
O75940	O75940	6	6	6	Survival of m	SMNDC1
O76076	O76076	2	2	2	WNT1-induci	WISP2
O94822	O94822	1	1	1	E3 ubiquitin-	LTN1
O94880;B4D(	O94880;B4D(	2;1	2;1	2;1	PHD finger pı	PHF14
O94906	O94906	13	13	13	Pre-mRNA-pı	PRPF6
O95071;E7EN	O95071;E7EN	14;13;2;1;1	14;13;2;1;1	14;13;2;1;1	E3 ubiquitin-	UBR5
Q6FI36;O951	Q6FI36;O951	5;5	5;5	5;5	Dual specifici	DUSP14
O95235;D6R!	O95235	15;2;2	15;2;2	15;2;2	Kinesin-like p	KIF20A
O95238	O95238	2	2	2	SAM pointed	SPDEF
O95239;Q59I	O95239;Q59I	18;18;11;8;8;	18;18;11;8;8;	18;18;11;8;8;	Chromosome	KIF4A
O95243;D6RI	O95243	11;1	11;1	11;1	Methyl-CpG-	MBD4
O95259	O95259	1	1	1	Potassium vc	KCNH1
O95365	O95365	1	1	1	Zinc finger ar	ZBTB7A
O95487	O95487	1	1	1	Protein trans	SEC24B
O95639;B7Z7	O95639;B7Z7	3;2;2;1;1;1	3;2;2;1;1;1	3;2;2;1;1;1	Cleavage and	CPSF4
Q53GY1;O95	Q53GY1;O95	1;1	1;1	1;1	BAG family m	BAG3
O95831;Q5R:	O95831;Q5R:	4;2;2;1	4;2;2;1	4;2;2;1	Apoptosis-in(	AIFM1
Q6FI97;Q53F	Q6FI97;Q53F	8;8;8;4;2;1;1	8;8;8;4;2;1;1	8;8;8;4;2;1;1	Actin-like prc	BAF53A;ACTL
O96028;Q05(	O96028;Q05(	9;8;5;4;1	9;8;5;4;1	9;8;5;4;1	Histone-lysin	WHSC1
Q6LET3;P004	Q6LET3;P004	2;2;1	2;2;1	2;2;1	Hypoxanthin	HPRT1
P02545;W8C	P02545;W8C	48;42;39;36;:	48;42;39;36;:	46;40;37;34;:	Prelamin-A/C	LMNA
P03973	P03973	4	4	4	Antileukopro	SLPI
P04350;B4DJ	P04350;B4DJ	19;17;16;5;2	1;1;1;1;1	0;0;0;0;0	Tubulin beta-	TUBB4A
V9HVZ4;Q2T:	V9HVZ4;Q2T:	8;8;8;6;6;4;3,	8;8;8;6;6;4;3,	8;8;8;6;6;4;3,	Glyceraldehy	GAPDH
V9HW43;P04	V9HW43;P04	6;6;5;3;2	6;6;5;3;2	6;6;5;3;2	Heat shock p	HSPB1
Q6IBR0;Q53E	Q6IBR0;Q53E	12;12;12;11;:	12;12;12;11;:	12;12;12;11;:	Dolichyl-diph	RPN1
P05023;B7Z3	P05023;B7Z3	9;8;5;5;5;5;5,	9;8;5;5;5;5;5,	9;8;5;5;5;5;5,	Sodium/pota	ATP1A1;ATP1
P05109	P05109	1	1	1	Protein S100-	S100A8
P05141;Q6N'	P05141;Q6N'	14;12	14;12	7;5	ADP/ATP trai	SLC25A5
Q0QEN7;V9H	Q0QEN7;V9H	6;6;6;5;2;1	6;6;6;5;2;1	6;6;6;5;2;1	ATP synthase	ATP5B
P06748;A4ZU	P06748;A4ZU	12;11;6;4;3;2	12;11;6;4;3;2	12;11;6;4;3;2	Nucleophosn	NPM1
Q5JP53;Q5SU	Q5JP53;Q5SU	23;23;23;22;:	6;6;6;5;5;6;3,	5;5;5;4;4;5;3,	Tubulin beta	TUBB;XTP3TF
P07814;B4DF	P07814;B4DF	6;3;2;2;2;1	6;3;2;2;2;1	6;3;2;2;2;1	Bifunctional g	EPRS
Q567R0;P07!	Q567R0;P07!	1;1	1;1	1;1	Cytochrome	UQCRH
P08047;Q59E	P08047;Q59E	6;5;4;1;1;1	6;5;4;1;1;1	6;5;4;1;1;1	Transcription	SP1;Sp1
V9HWE1;P08	V9HWE1;P08	26;26;25;24;:	25;25;24;23;:	23;23;22;21;:	Vimentin	VIM
P09382;F8W	P09382	7;2;1	7;2;1	7;2;1	Galectin-1	LGALS1
P09661;Q53(	P09661;Q53(	19;17;17;12;:	19;17;17;12;:	19;17;17;12;:	U2 small nuc	SNRPA1
Q71UI9;P0CC	Q71UI9;P0CC	4;4;3;2;2;2;1,	2;2;1;1;0;0;0,	2;2;1;1;0;0;0,	Histone H2A.	H2AFV;H2AF:
P0CW18;R4G	P0CW18;R4G	2;2	2;2	2;2	Serine protea	PRSS56

Q1PSX1;Q6R	Q1PSX1;Q6R	1;1;1;1	1;1;1;1	1;1;1;1	Zinc finger pr	Gli2;GLI2
V9HWB4;P11	V9HWB4;P11	40;40;15	40;40;15	38;38;13	78 kDa gluco	HSPA5
V9HW22;P11	V9HW22;P11	37;37;36;35;	35;35;34;34;	29;29;28;28;	Heat shock c	HSPA8
P11388;J3KT	P11388	41;4	41;4	31;1	DNA topoiso	TOP2A
P11441;Q5H	P11441;Q5H	2;2	2;2	2;2	Ubiquitin-like	UBL4A
Q6I9V5;P122	Q6I9V5;P122	11;11;10;6;6;	4;4;3;1;1;0;0;	4;4;3;1;1;0;0;	ADP/ATP tra	SLC25A6;SLC
P12273	P12273	2	2	2	Prolactin-ind	PIP
Q53T09;P13	Q53T09;P13	5;5	5;5	5;5	X-ray repair c	XRCC5
P13639;B4D	P13639;B4D	10;9;8;3;3;3;	9;8;7;3;3;3;2	9;8;7;3;3;3;2	Elongation fa	EEF2
Q6NTA2;P14	Q6NTA2;P14	24;24;23;22;	24;24;23;22;	23;23;22;21;	Heterogeneo	HNRNPL
P15924;Q4LE	P15924;Q4LE	66;51;26;14;	66;51;26;14;	66;51;26;14;	Desmoplakin	DSP;DSP vari
P16401;Q14	P16401	10;1	9;1	9;1	Histone H1.5	HIST1H1B
P16403	P16403	11	11	3	Histone H1.2	HIST1H1C
P16615;Q8N	P16615;Q8N	6;4;4;4;4;3;2	6;4;4;4;4;3;2	6;4;4;4;4;3;2	Sarcoplasmic	ATP2A2;DKFZ
Q5U079;P17	Q5U079;P17	2;2;1	2;2;1	2;2;1	Transcription	JUNB
P17535;U3K	P17535	3;1	3;1	3;1	Transcription	JUND
Q99557;Q9B	Q99557;Q9B	1;1;1	1;1;1	1;1;1	CCAAT/enha	CEBPB
P17812;B4E1	P17812;B4E1	12;10;10;6;1;	12;10;10;6;1;	12;10;10;6;1;	CTP synthase	CTPS1
P17987;E7EC	P17987;E7EC	9;5;5;4;3;2;2	9;5;5;4;3;2;2	9;5;5;4;3;2;2	T-complex pr	TCP1
P18077;F8W	P18077;F8W	7;5;5;5	7;5;5;5	7;5;5;5	60S ribosom	RPL35A
P18583;Q6Z	P18583	33;12;12;10;	33;12;12;10;	10;3;3;7;0;5;	Protein SON	SON
P19784;H3B	P19784;H3B	3;2;2;1	3;2;2;1	3;2;2;1	Casein kinase	CSNK2A2
P20700;E9PB	P20700;E9PB	33;30;27;27;	31;28;27;27;	29;26;25;25;	Lamin-B1	LMNB1
Q5HY54;P21	Q5HY54;P21	25;25;24;24;	25;25;24;24;	25;25;24;24;	Filamin-A	FLNA;FLJ001
P21912;A0A	P21912;A0A	2;1;1	2;1;1	2;1;1	Succinate de	SDHB
Q96BS4;P22	Q96BS4;P22	14;14;13;13;	14;14;13;13;	13;13;12;12;	rRNA 2-O-me	FBL
P22670;Q75	P22670	5;1;1;1;1;1	5;1;1;1;1;1	4;0;0;0;0;0	MHC class II	RFX1
Q9BSV4;Q86	Q9BSV4;Q86	7;7;7;5;2	7;7;7;5;2	7;7;7;5;2	Splicing facto	SFPQ
P23258;Q9N	P23258;Q9N	4;3;2;2	4;3;2;2	4;3;2;2	Tubulin gam	TUBG1;TUBG
V9HWC6;P23	V9HWC6;P23	4;4	4;4	4;4	Peptidyl-prol	PPIB
P23396;Q53	P23396;Q53	11;10;9;7;7;7	11;10;9;7;7;7	11;10;9;7;7;7	40S ribosom	RPS3
V9HWI5;P23	V9HWI5;P23	7;7;6;6;6;4;4	7;7;6;6;6;4;4	7;7;6;6;6;4;4	Cofilin-1	CFL1
P25398	P25398	5	5	5	40S ribosom	RPS12
V9HW26;P25	V9HW26;P25	8;8;7;6;5;4;4	8;8;7;6;5;4;4	8;8;7;6;5;4;4	ATP synthase	ATP5A1
P26368;B5B	P26368;B5B	9;8;6	9;8;6	9;8;6	Splicing facto	U2AF2
P26447	P26447	1	1	1	Protein S100	S100A4
P26599;A6N	P26599;A6N	15;13;8;6;5;4	15;13;8;6;5;4	13;11;7;5;4;3	Polypyrimidi	PTBP1
P27635;X1W	P27635;X1W	12;11;11;9;9;	12;11;11;9;9;	12;11;11;9;9;	60S ribosom	RPL10
P27824;D6R	P27824	5;2;2;2;2;1;1	5;2;2;2;2;1;1	5;2;2;2;2;1;1	Calnexin	CANX
Q5STP9;P28	Q5STP9;P28	7;7;6;6;6;4;4	7;7;6;6;6;4;4	7;7;6;6;6;4;4	Retinoic acid	NR2B2;RXRB
R4GN49;P29	R4GN49;P29	1;1	1;1	1;1	Protein S100	S100A2
Q1W6H1;P2	Q1W6H1;P2	13;13;12	13;13;12	13;13;12	DNA-3-meth	MPG
P30050;Q59	P30050;Q59	6;5;3;1	6;5;3;1	6;5;3;1	60S ribosom	RPL12;hCG_2
P31151	P31151	2	2	2	Protein S100	S100A7
P31276	P31276	3	3	3	Homeobox p	HOXC13

P31689;B7Z5	P31689	19;9;8	19;9;8	19;9;8	DnaJ homolo	DNAJA1
P31947	P31947	4	4	2	14-3-3 protei	SFN
P32320;Q71U	P32320;Q71U	4;2	4;2	3;1	Cytidine dea	CDA
P33981	P33981	9	9	9	Dual specifici	TTK
P35226;R4GN	P35226;R4GN	13;13;12;11;!	10;10;9;8;7;3	10;10;9;8;7;3	Polycomb co	BMI1;COMM
P35250;Q75N	P35250;Q75N	9;8;8;5;4;2;2,	9;8;8;5;4;2;2,	9;8;8;5;4;2;2,	Replication fa	RFC2
P35251;Q147	P35251;Q147	12;7;2;1;1	12;7;2;1;1	12;7;2;1;1	Replication fa	RFC1;LLDBP
P35659;B4DF	P35659;B4DF	13;11;11;8;7;	13;11;11;8;7;	13;11;11;8;7;	Protein DEK	DEK
P35908	P35908	51	51	1	Keratin, type	KRT2
Q8TAS0;P365	Q8TAS0;P365	5;5;4;2	5;5;4;2	5;5;4;2	ATP synthase	ATP5C1
P36578;Q59(	P36578;Q59(	20;18;17;14;:	20;18;17;14;:	20;18;17;14;:	60S ribosoma	RPL4
P37108;H0YL	P37108;H0YL	6;3;1	6;3;1	6;3;1	Signal recogr	SRP14
P38159;H3BT	P38159;H3BT	13;10;10;9;8;	13;10;10;9;8;	13;10;10;9;8;	RNA-binding	RBMX;RBMX
P38432;I3L36	P38432	14;1	14;1	14;1	Coilin	COIL
P39023;Q8TE	P39023;Q8TE	16;15;14;13;:	16;15;14;13;:	16;15;14;13;:	60S ribosoma	RPL3;rpl3
P39880;Q3LI	P39880	4;1;1	4;1;1	4;1;1	Homeobox p	CUX1
Q53H34;P404	Q53H34;P404	6;6;5;5;5;3;3,	6;6;5;5;5;3;3,	6;6;5;5;5;3;3,	60S ribosoma	RPL13A;RPL1
P41162	P41162	2	2	2	ETS transloca	ETV3
P41208	P41208	1	1	1	Centrin-2	CETN2
P42166	P42166	30	30	21	Lamina-assoc	TMPO
P42285;A8K6	P42285;A8K6	7;6;5;1	7;6;5;1	7;6;5;1	Superkiller vi	SKIV2L2
P42677;C9J1	P42677;C9J1	2;1;1;1;1;1	2;1;1;1;1;1	2;1;1;1;1;1	40S ribosoma	RPS27;RPS27
P42695;G3V1	P42695;G3V1	13;8;5;4	13;8;5;4	13;8;5;4	Condensin-2	NCAPD3
P43246;V9H(	P43246;V9H(	15;13;13;13;:	15;13;13;13;:	15;13;13;13;:	DNA mismatc	MSH2
P46109	P46109	12	12	12	Crk-like prote	CRKL
Q6IAX2;P467	Q6IAX2;P467	3;3;2;2;2	3;3;2;2;2	3;3;2;2;2	60S ribosoma	RPL21
P46783;Q59(	P46783;Q59(	3;3;2;2;1;1	3;3;2;2;1;1	3;3;2;2;1;1	40S ribosoma	RPS10
P48059;B4DU	P48059;B4DU	3;2;2;2;2;1;1,	3;2;2;2;2;1;1,	3;2;2;2;2;1;1,	LIM and sene	LIMS1
Q8NE89;P48	Q8NE89;P48	2;2;1	2;2;1	2;2;1	Tissue factor	TFPI2
P48380	P48380	2	1	1	Transcription	RFX3
P49327	P49327	4	4	4	Fatty acid syr	FASN
P49411;H3BN	P49411	7;1	7;1	7;1	Elongation fa	TUFM
P49454;Q9UI	P49454	3;1;1	3;1;1	3;1;1	Centromere	CENPF
P49458;Q8W	P49458;Q8W	4;3;1;1;1;1	4;3;1;1;1;1	4;3;1;1;1;1	Signal recogr	SRP9
Q5U045;Q53	Q5U045;Q53	6;6;6;4;4;4;3,	2;2;2;0;0;1;0,	2;2;2;0;0;1;0,	Casein kinase	CSNK1E
P49711	P49711	1	1	1	Transcription	CTCF
P49750;F8VU	P49750;F8VU	8;5;5;5;3;2;2	8;5;5;5;3;2;2	8;5;5;5;3;2;2	YLP motif-co	YLPM1;FLJ00
P49756;E9PC	P49756	14;5;3;1;1	14;5;3;1;1	14;5;3;1;1	RNA-binding	RBM25
P49792;A6NI	P49792	36;10;10;9;9;	36;10;10;9;9;	36;10;10;9;9;	E3 SUMO-pro	RANBP2
P50402;Q5H	P50402;Q5H	11;8;1	11;8;1	11;8;1	Emerin	EMD
V9GYF7;V9G	V9GYF7;V9G	1;1;1;1;1	1;1;1;1;1	1;1;1;1;1	28S ribosoma	DAP3
P51571;A6NI	P51571;A6NI	5;3	5;3	5;3	Translocon-a	SSR4
P51587;A1YE	P51587;A1YE	32;27;7;4;3;3	32;27;7;4;3;3	32;27;7;4;3;3	Breast cance	BRCA2
P51858;B2RE	P51858;B2RE	7;6;4;4;4;1;1,	6;5;4;4;3;0;0,	6;5;4;4;3;0;0,	Hepatoma-de	HDGF
P51991;B4E0	P51991	19;3	19;3	2;2	Heterogeneo	HNRNPA3

P52272;A0A(	P52272;A0A(	44;43;33;31;:	44;43;33;31;:	44;43;33;31;:	Heterogeneo	HNRNPM;OR
P52298;F8W	P52298;F8W	2;1;1;1;1;1	2;1;1;1;1;1	2;1;1;1;1;1	Nuclear cap-l	NCBP2;DKFZ|
P52701;A0A(	P52701;A0A(	21;17;17;4;3;	21;17;17;4;3;	21;17;17;4;3;	DNA mismat	MSH6;GTBP
P52952;A1E(	P52952;A1E(	8;4;4;4;4;4;4	8;4;4;4;4;4;4	8;4;4;4;4;4;4	Homeobox p	NKX2-5
P53621	P53621	1	1	1	Coatomer su	COPA
Q6IBA2;P539	Q6IBA2;P539	4;4;4;3;2	4;4;4;3;2	4;4;4;3;2	Activated RN	PC4;SUB1
P54136	P54136	1	1	1	Arginine--tRN	RARS
P55000	P55000	1	1	1	Secreted Ly-6	SLURP1
V9HW80;P55	V9HW80;P55	10;10;6;5;4;4	10;10;6;5;4;4	10;10;6;5;4;4	Transitional e	VCP
P55081	P55081	3	3	3	Microfibrillar	MFAP1
P55265;A0A(	P55265;A0A(	36;34;33;33	36;34;33;33	2;2;2;2	Double-stran	ADAR
P55795;B4DF	P55795;B4DF	13;11;10	7;6;6	7;6;6	Heterogeneo	HNRNPH2
P56537;B7ZE	P56537;B7ZE	6;3;1;1;1;1	6;3;1;1;1;1	6;3;1;1;1;1	Eukaryotic tr	EIF6
P57740	P57740	1	1	1	Nuclear pore	NUP107
Q53ZD3;P58(	Q53ZD3;P58(	3;3;3;2;2;2	2;2;2;1;1;1	2;2;2;1;1;1	Forkhead bo:	FOXL2
Q6EZE9;P596	Q6EZE9;P596	2;2;2	2;2;2	2;2;2	Neutrophil de	DEFA3;DEFA1
P60468	P60468	1	1	1	Protein trans	SEC61B
Q53GK6;Q53	Q53GK6;Q53	20;20;20;20;:	1;1;1;1;1;1;0;	1;1;1;1;1;1;0;	Actin, cytopla	PS1TP5BP1;A
P60866;E5RJ	P60866;E5RJ	4;3;2;1	4;3;2;1	4;3;2;1	40S ribosoma	RPS20
Q6IBH6;P612	Q6IBH6;P612	8;8;7;7;7;7;6;	8;8;7;7;7;7;6;	8;8;7;7;7;7;6;	60S ribosoma	RPL26;KRBA2
P61964;V9G;	P61964	10;4;2	10;4;2	10;4;2	WD repeat-c	WDR5
P62136;E9PM	P62136;E9PM	6;4;4;4;4;4;4;	2;2;0;0;0;0;0;	2;2;0;0;0;0;0;	Serine/threo	PPP1CA;PPP1
Q5JR94;P622	Q5JR94;P622	9;9;8;4	9;9;8;4	9;9;8;4	40S ribosoma	RPS8
P62263;H0YE	P62263;H0YE	8;6;6;2	8;6;6;2	8;6;6;2	40S ribosoma	RPS14
P62269;J3JS6	P62269	9;3;2;2	9;3;2;2	9;3;2;2	40S ribosoma	RPS18
P62273	P62273	1	1	1	40S ribosoma	RPS29
P62280;M0Q	P62280;M0Q	10;9;4;3	10;9;4;3	10;9;4;3	40S ribosoma	RPS11
P62306	P62306	2	2	2	Small nuclea	SNRPF
P62314;J3QL	P62314;J3QL	2;1;1;1	2;1;1;1	2;1;1;1	Small nuclea	SNRPD1
P62316;K7ER	P62316;K7ER	4;3;1	4;3;1	4;3;1	Small nuclea	SNRPD2
P62318;H3B1	P62318	3;1	3;1	3;1	Small nuclea	SNRPD3
P62424;Q5T8	P62424;Q5T8	12;7;6;6	12;7;6;6	12;7;6;6	60S ribosoma	RPL7A;RP-L7a
P62633;Q6T5	P62633;Q6T5	5;3	5;3	5;3	Cellular nucle	CNBP
P62750;H7BY	P62750;H7BY	9;8;8;8;8;6;2;	9;8;8;8;8;6;2;	9;8;8;8;8;6;2;	60S ribosoma	RPL23A
P62851	P62851	3	3	3	40S ribosoma	RPS25
P62877	P62877	1	1	1	E3 ubiquitin-	RBX1
Q59GN2;P62	Q59GN2;P62	1;1	1;1	1;1	Putative 60S	RPL39P5;RPL
P62906;Q1JC	P62906;Q1JC	6;5;2	6;5;2	6;5;2	60S ribosoma	RPL10A
Q5VVD0;P62	Q5VVD0;P62	9;9;8;7;5	9;9;8;7;5	9;9;8;7;5	60S ribosoma	RPL11
P62917;B4D\	P62917;B4D\	10;9;9;8;8;2	10;9;9;8;8;2	10;9;9;8;8;2	60S ribosoma	RPL8
Q3MIH3;P62	Q3MIH3;P62	7;7;6;6;6;6;6;	1;1;0;0;0;0;0;	1;1;0;0;0;0;0;	Ubiquitin-60S	UBA52;UBB;I
Q5FWY2;P63	Q5FWY2;P63	8;8;8;7;7;4;3;	8;8;8;7;7;4;3;	8;8;8;7;7;4;3;	Guanine nucl	GNAS;GSA
Q56VW8;P63	Q56VW8;P63	3;3;2;1;1;1;1;	3;3;2;1;1;1;1;	3;3;2;1;1;1;1;	Serine/threo	PPP2R2A;PPF
Q6FGH9;P63	Q6FGH9;P63	5;5;4;4;1;1	5;5;4;4;1;1	5;5;4;4;1;1	Dynein light (	DNCL1;DYNLI
Q8WVC2;Q6I	Q8WVC2;Q6I	2;2;2;1	2;2;2;1	2;2;2;1	40S ribosoma	RPS21

P63261;B4E3	P63261;B4E3	20;19;18;11;:	20;19;18;11;:	1;1;1;1;1;1;1,	Actin, cytopla	ACTG1
P67809;A0A(	P67809;A0A(	6;5;5;4;4;3;3,	6;5;5;4;4;3;3,	6;5;5;4;4;3;3,	Nuclease-ser	YBX1
Q6IPT9;Q6IP:	Q6IPT9;Q6IP:	21;21;21;21;:	21;21;21;21;:	13;13;13;13;:	Elongation fa	EEF1A1;EEF1
P68366;C9JE'	P68366	21;8;8;8;6	21;8;8;8;6	4;3;3;3;2	Tubulin alpha	TUBA4A
Q8IZ29;P683	Q8IZ29;P683	24;24;23;18;:	24;24;23;18;:	0;0;0;0;0	Tubulin beta-	TUBB2C;TUB
Q5VUC3;P78	Q5VUC3;P78	2;2;1	2;2;1	2;2;1	Ribonuclease	RPP38
P78346;Q5V(	P78346;Q5V(	7;5;5;5	7;5;5;5	7;5;5;5	Ribonuclease	RPP30
Q5J8M4;P78.	Q5J8M4;P78.	6;6;5;2	6;6;5;2	6;6;5;2	mRNA export	RAE1
P78413	P78413	2	2	2	Iroquois-clas:	IRX4
P78527;B4DL	P78527	131;35;2;1	131;35;2;1	131;35;2;1	DNA-depend	PRKDC
P80723	P80723	1	1	1	Brain acid sol	BASP1
P81605	P81605	2	2	2	Dermcidin;Su	DCD
P82979;Q567	P82979;Q567	4;3;3;3;2;1	4;3;3;3;2;1	4;3;3;3;2;1	SAP domain-	SARNP;CIP29
Q00839;B3K)	Q00839;B3K)	21;20;20;18;:	21;20;20;18;:	21;20;20;18;:	Heterogeneo	HNRNPU;HNI
Q01081;B5B(	Q01081;B5B(	10;9;5;4;4;4;.	10;9;5;4;4;4;.	10;9;5;4;4;4;.	Splicing facto	U2AF1;U2AF:
Q01196;A8M	Q01196;A8M	7;4;4;4;2;2;2,	7;4;4;4;2;2;2,	7;4;4;4;2;2;2,	Runt-related	RUNX1
Q01650;Q2M	Q01650	5;2;2;2;1;1;1,	5;2;2;2;1;1;1,	5;2;2;2;1;1;1,	Large neutral	SLC7A5
Q59GG3;Q6F	Q59GG3;Q6F	2;2;2;1	2;2;2;1	2;2;2;1	Transcription	TFAP4
Q01780;Q59(	Q01780;Q59(	8;7;7;7;3	8;7;7;7;3	8;7;7;7;3	Exosome con	EXOSC10
Q02413	Q02413	9	9	9	Desmoglein-:	DSG1
Q71UH4;Q02	Q71UH4;Q02	36;36;33;31;:	26;26;23;21;:	26;26;23;21;:	DNA topoisoı	TOP2B
Q03164;E9PF	Q03164	29;14;4;4;4;3	29;14;4;4;4;3	28;14;4;4;4;3	Histone-lysin	KMT2A
Q05BU6;Q6P	Q05BU6;Q6P	2;2;2;2;2;2;1,	2;2;2;2;2;2;1,	2;2;2;2;2;2;1,	Serine/argini	SFRS11;SRSF:
Q05639;Q59(	Q05639	11;5	3;1	3;1	Elongation fa	EEF1A2
Q05CR6;Q86	Q05CR6;Q86	9;9;8;5;1;1	9;9;8;5;1;1	9;9;8;5;1;1	Serine/threo	VRK2
Q06330;A0A(	Q06330;A0A(	5;4;3;3;2;2;2,	5;4;3;3;2;2;2,	5;4;3;3;2;2;2,	Recombining	RBPJ
Q06587;B4D'	Q06587;B4D'	17;14;14;14;:	13;10;10;11;:	13;10;10;11;:	E3 ubiquitin-	RING1
Q06830;A0A(	Q06830;A0A(	15;14;14;6	15;14;14;6	12;11;11;3	Peroxiredoxi	PRDX1
Q8IUG1;Q07(	Q8IUG1;Q07(	2;2;1;1	2;2;1;1	2;2;1;1	Keratin-assoc	KRTAP1-3;KR
Q08211;Q58	Q08211;Q58	10;7;7;3	10;7;7;3	10;7;7;3	ATP-depende	DHX9
Q9HB00;Q08	Q9HB00;Q08	3;3	3;3	3;3	Desmocollin-	DSC1
Q09028;H0YI	Q09028;H0YI	9;5;4;4;4;3;1,	9;5;4;4;4;3;1,	5;2;2;2;2;1;1,	Histone-bind	RBBP4
Q09666;B4D'	Q09666;B4D'	12;7;3;2;1;1;:	12;7;3;2;1;1;:	12;7;3;2;1;1;:	Neuroblast d	AHNAK
Q6AHZ7;Q12	Q6AHZ7;Q12	17;17;16;3;2	17;17;16;3;2	17;17;16;3;2	General tran:	DKFZp686A1
Q12830;E7E1	Q12830;E7E1	22;20;19;4;4;	22;20;19;4;4;	22;20;19;4;4;	Nucleosome-	BPTF
Q12872;Q59	Q12872;Q59	3;2;1;1;1;1;1	3;2;1;1;1;1;1	3;2;1;1;1;1;1	Splicing facto	SFSWAP
Q12873;B4DI	Q12873;B4DI	11;10;6;5;5;3	5;4;4;3;3;1;1,	5;4;4;3;3;1;1,	Chromodoma	CHD3
Q12947;Q12:	Q12947;Q12:	4;2	4;2	3;1	Forkhead bo;	FOXF2;FOXF1
Q13151	Q13151	6	6	6	Heterogeneo	HNRNPA0
Q13263;B2R8	Q13263;B2R8	14;13;5;5;4	14;13;5;5;4	14;13;5;5;4	Transcription	TRIM28
Q96F82;Q13.	Q96F82;Q13.	19;19;18;18	19;19;18;18	19;19;18;18	Origin recogr	ORC1L;ORC1
Q13428;Q59	Q13428	18;6	18;6	2;2	Treacle prote	TCOF1
Q13509;Q9B'	Q13509;Q9B'	18;17;17;17;:	6;5;5;6;5;4;1,	3;2;2;3;2;1;1,	Tubulin beta-	TUBB3
Q13535;H0Y8	Q13535	7;1;1;1	7;1;1;1	7;1;1;1	Serine/threo	ATR
Q13573;B7Z9	Q13573;B7Z9	11;10;10;10;:	11;10;10;10;:	11;10;10;10;:	SNW domain	SNW1

Q549U1;Q13	Q549U1;Q13	7;7	5;5	5;5	Transformer-	HSU53209;TF
Q13751;B4DI	Q13751;B4DI	5;3;1	5;3;1	5;3;1	Laminin subu	LAMB3
Q13761;A0A(	Q13761;A0A(	6;5;4;3;1;1;1	6;5;4;3;1;1;1	6;5;4;3;1;1;1	Runt-related	RUNX3
Q5T0F3;Q13	Q5T0F3;Q13	11;11;4;3	11;11;4;3	11;11;4;3	Nucleolar GT	GNL2
Q9BVA1;Q13	Q9BVA1;Q13	19;19;18;18;	2;2;2;1;2;2;2;	1;1;1;0;1;1;1;	Tubulin beta-	TUBB2B;TUB
Q14151;A0PJ	Q14151;A0PJ	14;8;1	14;8;1	7;3;1	Scaffold atta	SAFB2
Q14257;H0YI	Q14257;H0YI	6;4;2	6;4;2	6;4;2	Reticulocalbi	RCN2
Q14258;Q59	Q14258;Q59	4;3;2	4;3;2	4;3;2	E3 ubiquitin/	TRIM25
Q14331;E9PF	Q14331;E9PF	4;3;2;2;2;1;1	4;3;2;2;2;1;1	4;3;2;2;2;1;1	Protein FRG1	FRG1;FRG1B
Q14432;Q13	Q14432;Q13	2;1;1	2;1;1	2;1;1	cGMP-inhibit	PDE3A;PDE3I
Q14498;G3X	Q14498;G3X	23;22;21;21;	23;22;21;21;	23;22;21;21;	RNA-binding	RBM39;DKFZ
Q14532;CON	Q14532;CON	8;7	1;1	1;1	Keratin, type	KRT32
Q14669;Q57	Q14669	25;1;1	25;1;1	19;1;1	E3 ubiquitin-	TRIP12
Q14674	Q14674	1	1	1	Separin	ESPL1
Q14676;B4D	Q14676;B4D	12;9;1;1;1	12;9;1;1;1	12;9;1;1;1	Mediator of I	MDC1
Q14677	Q14677	2	2	2	Clathrin inter	CLINT1
Q14683;G8JL	Q14683;G8JL	26;25;20;20;	26;25;20;20;	26;25;20;20;	Structural ma	SMC1A;DKFZ
Q14684;Q6P	Q14684	26;9	26;9	26;9	Ribosomal RI	RRP1B
Q14690;B4DI	Q14690;B4DI	7;4;4	7;4;4	7;4;4	Protein RRP5	PDCD11
Q14692	Q14692	2	2	2	Ribosome bio	BMS1
Q14781	Q14781	47	47	47	Chromobox p	CBX2
Q14807;A0A(	Q14807;A0A(	19;18;18;17;	19;18;18;17;	19;18;18;17;	Kinesin-like p	KIF22
Q14865	Q14865	4	4	4	AT-rich intera	ARID5B
Q14966;A8K	Q14966;A8K	20;18;16;12;	20;18;16;12;	20;18;16;12;	Zinc finger pr	ZNF638
Q14980;Q4LI	Q14980;Q4LI	133;133;96;4	1;1;0;0;0;0;0;	1;1;0;0;0;0;0;	Nuclear mito	NUMA1;NUM
Q15050	Q15050	2	2	2	Ribosome bio	RRS1
Q15058	Q15058	1	1	1	Kinesin-like p	KIF14
Q15061	Q15061	1	1	1	WD repeat-co	WDR43
Q15072;A8K	Q15072	5;1;1	5;1;1	5;1;1	Zinc finger pr	ZNF146
Q15149;D3D	Q15149;D3D	18;11;2;1;1	18;11;2;1;1	15;8;2;1;1	Plectin	PLEC;PLEC1
Q53SS8;Q15	Q53SS8;Q15	12;12;2;1;1;1	12;12;2;1;1;1	8;8;0;0;0;0;0;	Poly(rC)-bind	PCBP1
Q15654;F2Z(	Q15654;F2Z(	2;1;1;1	2;1;1;1	2;1;1;1	Thyroid recep	TRIP6;TRIP6i
Q15717;M0C	Q15717;M0C	18;10;3;1;1;1	18;10;3;1;1;1	18;10;3;1;1;1	ELAV-like pro	ELAVL1
Q6FHQ0;Q16	Q6FHQ0;Q16	9;9;8;6;6;3;2	5;5;4;4;3;1;1	5;5;4;4;3;1;1	Histone-bind	RBBP7
Q1ED39;H3B	Q1ED39	5;2;1;1	5;2;1;1	5;2;1;1	Lysine-rich nu	KNOP1
Q1KMD3;H3I	Q1KMD3;H3I	8;7	8;7	8;7	Heterogeneo	HNRNPUL2;h
Q1RMC9;Q9	Q1RMC9;Q9	6;6;2	6;6;2	6;6;2	Protein LAP2	ERBB2IP
Q1W6G4;Q9	Q1W6G4;Q9	11;11;9;8;7;7	4;4;4;2;2;2;1;	4;4;4;2;2;2;1;	Putative RNA	LUC7L
Q2KHR3;Q9H	Q2KHR3	40;14;14;12;	40;14;14;12;	40;14;14;12;	Glutamine ar	QSER1
Q2L6I2;Q8NE	Q2L6I2;Q8NE	9;9;6;6;6;5;4;	9;9;6;6;6;5;4;	9;9;6;6;6;5;4;	ATP-binding	ABCF1
Q8IUC1;Q3LI	Q8IUC1;Q3LI	2;2	2;2	2;2	Keratin-asso	KRTAP11-1
Q3ZCM7;A0A	Q3ZCM7;A0A	11;10;10	2;2;2	2;2;2	Tubulin beta-	TUBB8
Q4VC44;I3L1	Q4VC44;I3L1	4;2;1;1;1;1;1	3;2;0;0;1;1;1	3;2;0;0;1;1;1	FLYWCH-type	FLYWCH1
Q8IUC0;Q52I	Q8IUC0;Q52I	1;1	1;1	1;1	Keratin-asso	KRTAP13-1;K
Q53EQ6	Q53EQ6	1	1	1	Tigger transp	TIGD5

Q92908;Q53	Q92908;Q53	3;3	3;3	3;3	Transcription	GATA6
Q9BUN6;Q9I	Q9BUN6;Q9I	1;1;1;1	1;1;1;1	1;1;1;1	28S ribosoma	MRPS30
Q9NYD6;Q53	Q9NYD6;Q53	6;6;3	6;6;3	6;6;3	Homeobox p	HOXC10
Q9Y5M8;Q54	Q9Y5M8;Q54	3;3;2	3;3;2	3;3;2	Signal recogr	SRPRB
Q56NI9;H0YE	Q56NI9	7;3;1;1;1	7;3;1;1;1	7;3;1;1;1	N-acetyltrans	ESCO2
Q92922;Q58	Q92922;Q58	16;16;14;14;	8;8;7;6;7;4;3	8;8;7;6;7;4;3	SWI/SNF com	SMARCC1
Q59EC0;B7Z8	Q59EC0;B7Z8	35;27	1;1	1;1		
Q59EJ3	Q59EJ3	23	1	0		
Q9NRW3;Q5	Q9NRW3;Q5	10;10;9;1;1;1	10;10;9;1;1;1	10;10;9;1;1;1	DNA dC->dU-	APOBEC3C
Q5BKZ1;A0A	Q5BKZ1;A0A	13;7;1	13;7;1	13;7;1	DBIRD compl	ZNF326
Q5C9Z4	Q5C9Z4	7	7	7	Nucleolar MI	NOM1
Q5H928;Q99	Q5H928;Q99	1;1	1;1	1;1	3-hydroxyacy	HSD17B10
Q5H9F3	Q5H9F3	12	1	1	BCL-6 corepr	BCORL1
Q5QJE6;J3KP	Q5QJE6;J3KP	5;3;3;1	5;3;3;1	5;3;3;1	Deoxynucleo	DNTTIP2
Q5RKV6	Q5RKV6	4	4	4	Exosome con	EXOSC6
Q5SNT2;H0Y	Q5SNT2;H0Y	3;2;1	3;2;1	3;2;1	Transmembr	TMEM201
Q5SRE5;Q9B	Q5SRE5;Q9B	4;2;1	4;2;1	4;2;1	Nucleoporin	NUP188
X6RGJ2;Q5SS	X6RGJ2;Q5SS	5;5;4;4;1;1	5;5;4;4;1;1	5;5;4;4;1;1	Heterochrom	HP1BP3
Q5SVZ6;B4D	Q5SVZ6;B4D	10;9;7;7;2	10;9;7;7;2	10;9;7;7;2	Zinc finger M	ZMYM1
Q5SY16	Q5SY16	13	13	13	Polynucleotic	NOL9
Q5T280;Q9P	Q5T280	9;3;3;1	9;3;3;1	9;3;3;1	Uncharacteri	C9orf114
Q5T3I0;A8KA	Q5T3I0;A8KA	11;10;9;9;7;3	11;10;9;9;7;3	11;10;9;9;7;3	G patch dom	GPATCH4
Q5T3J3	Q5T3J3	3	3	3	Ligand-deper	LRIF1
Q5T440	Q5T440	1	1	1	Putative tran	IBA57
Q5T5X7	Q5T5X7	3	3	3	BEN domain-	BEND3
Q5T670;Q7L	Q5T670;Q7L	2;2	2;2	2;2	Protein MCM	MCM10
Q5T749	Q5T749	11	11	11	Keratinocyte	KPRP
Q5T750	Q5T750	2	2	2	Skin-specific	XP32
Q9BSD7;Q5T	Q9BSD7;Q5T	1;1	1;1	1;1	Cancer-relate	NTPCR;C1orf
Q9UNX4;Q5T	Q9UNX4;Q5T	2;2;1	2;2;1	2;2;1	WD repeat-c	WDR3
Q5TEC6	Q5TEC6	3	3	1		HIST2H3PS2
Q5TGY3	Q5TGY3	8	8	8	AT-hook DNA	AHDC1
Q5THK1;H7B	Q5THK1;H7B	6;3;2;1	6;3;2;1	6;3;2;1	Protein PRR1	PRR14L
Q5UIP0;H7C	Q5UIP0	54;23;11;8;6	54;23;11;8;6	54;23;11;8;6	Telomere-ass	RIF1
Q5VTL8;A0A	Q5VTL8;A0A	8;4;2	8;4;2	8;4;2	Pre-mRNA-sp	PRPF38B
Q5VUA4	Q5VUA4	8	8	8	Zinc finger pr	ZNF318
Q5VV42	Q5VV42	1	1	1	Threonylcarb	CDKAL1
Q5VWN6;Q6	Q5VWN6	20;4	20;4	20;4	Protein FAM	FAM208B
Q5VZL5;H7C	Q5VZL5;H7C	13;10	13;10	13;10	Zinc finger M	ZMYM4
Q96I25;Q5W	Q96I25;Q5W	3;3;2;2;1	3;3;2;2;1	3;3;2;2;1	Splicing facto	RBM17
Q5W0B1	Q5W0B1	8	8	8	RING finger p	RNF219
Q68CP0;Q96	Q68CP0;Q96	6;6;6;4;2;1	6;6;6;4;2;1	6;6;6;4;2;1	Zinc finger pr	DKFZp686B2
Q63ZY3;K7EF	Q63ZY3;K7EF	3;2	3;2	3;2	KN motif and	KANK2
Q9ULW0;Q6	Q9ULW0;Q6	33;33;30;12	33;33;30;12	33;33;30;12	Targeting prc	TPX2;HCTP4
Q68CP9;F8W	Q68CP9;F8W	8;6;6;4;4;2;1	8;6;6;4;4;2;1	8;6;6;4;4;2;1	AT-rich intera	ARID2

Q69YH5;E9PI	Q69YH5;E9PI	29;28;26;19;!	29;28;26;19;!	29;28;26;19;!	Cell division (	CDCA2
Q9H0U9;Q6F	Q9H0U9;Q6F	3;3;2	3;3;2	3;3;2	Testis-specifi	TSPYL1;TSPYI
Q9Y285;Q6IE	Q9Y285;Q6IE	5;5;4;4;3	5;5;4;4;3	5;5;4;4;3	Phenylalanin	FARSA;FARSL
Q6IQ49	Q6IQ49	1	1	1	Protein SDE2	SDE2
Q6NW34;B4I	Q6NW34;B4I	5;4;4;4;4;3;3;	5;4;4;4;4;3;3;	5;4;4;4;4;3;3;	Uncharacteri	C3orf17
Q6NZY4;A8K:	Q6NZY4;A8K!	9;8;5;5;4;3	9;8;5;5;4;3	9;8;5;5;4;3	Zinc finger C(	ZCCHC8
Q6P0N0;G5E	Q6P0N0;G5E	6;3;1;1	6;3;1;1	6;3;1;1	Mis18-bindin	MIS18BP1
Q6P2Q9;B4D	Q6P2Q9;B4D	15;10;5;5;2;2	15;10;5;5;2;2	15;10;5;5;2;2	Pre-mRNA-pr	PRPF8
Q6P4F7;H3BI	Q6P4F7;H3BI	6;4;2	6;4;2	6;4;2	Rho GTPase-a	ARHGAP11A
Q6PK81	Q6PK81	1	1	1	Zinc finger pr	ZNF773
Q6RFH5;E9P!	Q6RFH5;E9P!	5;4;2;1;1	5;4;2;1;1	5;4;2;1;1	WD repeat-co	WDR74
Q6UB99	Q6UB99	1	1	1	Ankyrin repe	ANKRD11
Q6UN15;A0A	Q6UN15;A0A	7;6;5	7;6;5	7;6;5	Pre-mRNA 3-	FIP1L1
Q6UWP8	Q6UWP8	2	2	2	Suprabasin	SBSN
Q6ZRF7	Q6ZRF7	1	1	1	Putative zinc	ZNF818P
Q6ZVX9	Q6ZVX9	1	1	1	Progestin and	PAQR9
Q70L73	Q70L73	16	1	1		MKI67
Q71RB6;Q8N	Q71RB6;Q8N	2;2	2;2	2;2	F-box/LRR-re	FBXL6
Q7L3T8	Q7L3T8	4	4	4	Probable pro	PARS2
Q7L4I2;B3KM	Q7L4I2;B3KM	3;2;1;1;1	3;2;1;1;1	3;2;1;1;1	Arginine/seri	RSRC2;FLJ11(
Q7Z2Z1;H0YI	Q7Z2Z1	12;1	12;1	12;1	Treslin	TICRR
Q7Z333;A0A(	Q7Z333;A0A(	11;10;3;1	11;10;3;1	11;10;3;1	Probable heli	SETX
Q7Z3K3;M0R	Q7Z3K3	4;1;1	4;1;1	4;1;1	Pogo transpo	POGZ
Q7Z589;E9PI	Q7Z589;E9PI	18;16;16;8;6;	18;16;16;8;6;	6;5;5;1;1;1	Protein EMSY	EMSY;C11orf
Q7Z5J4;A8M	Q7Z5J4;A8M	7;6;1	7;6;1	7;6;1	Retinoic acid	RAI1
Q7Z6E9;A0A(	Q7Z6E9	3;1;1;1;1	3;1;1;1;1	3;1;1;1;1	E3 ubiquitin-	RBBP6
Q7Z7G8	Q7Z7G8	1	1	1	Vacuolar pro	VPS13B
Q86SE9	Q86SE9	1	1	1	Polycomb gro	PCGF5
Q86SJ6	Q86SJ6	3	3	3	Desmoglein-4	DSG4
Q86T29;Q6ZI	Q86T29;Q6ZI	2;1;1;1;1;1	1;1;1;1;1;1	0;0;0;0;0;0	Zinc finger pr	ZNF605;ZSCA
Q86TJ2;B3KX	Q86TJ2;B3KX	2;1	2;1	2;1	Transcription	TADA2B
Q86U42;B4D	Q86U42;B4D	3;2;2;1	3;2;2;1	3;2;2;1	Polyadenylat	PABPN1
Q86U86;E7E'	Q86U86;E7E'	18;17;13;6;3;	18;17;13;6;3;	18;17;13;6;3;	Protein polyb	PBRM1
Q86UT8;E9P.	Q86UT8;E9P.	8;5;3;2	8;5;3;2	8;5;3;2	Coiled-coil do	CCDC84
Q86V81;E9PI	Q86V81;E9PI	7;6	7;6	7;6	THO complex	ALYREF
Q86Y79;A0A(	Q86Y79;A0A(	6;5;4;4;3	6;5;4;4;3	6;5;4;4;3	Probable pep	PTRH1
Q86YC2;B4DI	Q86YC2;B4DI	4;3	4;3	4;3	Partner and l	PALB2
Q8IUF8;H0Y9	Q8IUF8;H0Y9	2;1;1	2;1;1	2;1;1	Bifunctional l	MINA
Q8IVT2	Q8IVT2	11	11	11	Mitotic intera	MISP
Q8IWP6;B4D	Q8IWP6	24;11;7;5;5;5	1;0;0;0;0;0;0;	1;0;0;0;0;0;0;	0;0;0;0;0;0;0	
Q8IWZ3;Q8T	Q8IWZ3;Q8T	4;3;3;2;2;2;1;	4;3;3;2;2;2;1;	4;3;3;2;2;2;1;	Ankyrin repe	ANKHD1;FLJ(
Q8IXB1;Q6YF	Q8IXB1	28;11;10;10;!	28;11;10;10;!	7;6;7;7;5;5	DnaJ homolo	DNAJC10
Q8IY57;G3V1	Q8IY57;G3V1	2;1;1;1;1;1	2;1;1;1;1;1	2;1;1;1;1;1	YY1-associate	YAF2
Q8IY81;B4DK	Q8IY81;B4DK	7;6	7;6	7;6	pre-rRNA pro	FTSJ3
Q8IYH5;C9J2.	Q8IYH5	3;1;1	3;1;1	3;1;1	ZZ-type zinc f	ZZZ3

Q8IYL3	Q8IYL3	3	3	3	UPF0688 pro	C1orf174
Q8IZT6;B3KV	Q8IZT6;B3KV	12;10;5;5;4;2	12;10;5;5;4;2	12;10;5;5;4;2	Abnormal spi	ASPM
Q8NFV1;Q8N	Q8NFV1;Q8N	3;3;2	1;1;1	1;1;1		KRTHB6;KRTH
Q8N1F7;A8K	Q8N1F7;A8K	7;6;6;3;3;3;2;	7;6;6;3;3;3;2;	7;6;6;3;3;3;2;	Nuclear pore	NUP93
Q8N556;Q6Z	Q8N556;Q6Z	8;4;1;1	8;4;1;1	8;4;1;1	Actin filamen	AFAP1
Q8N5F7;Q5N	Q8N5F7	6;2;1;1	6;2;1;1	6;2;1;1	NF-kappa-B-a	NKAP
Q8N5L8	Q8N5L8	2	2	2	Ribonuclease	RPP25L
Q8N5Y2;B4E	Q8N5Y2;B4E	3;2;2;2;1;1;1	3;2;2;2;1;1;1	3;2;2;2;1;1;1	Male-specific	MSL3
Q8N726	Q8N726	5	5	2	Cyclin-depen	CDKN2A
Q8N883;P0C	Q8N883;P0C	3;2	2;1	0;0	Zinc finger pr	ZNF614;ZNF8
Q8NAF0	Q8NAF0	3	3	3	Zinc finger pr	ZNF579
Q8NAV1	Q8NAV1	2	2	2	Pre-mRNA-sp	PRPF38A
Q8NB16;I3L2	Q8NB16;I3L2	4;2;2;2;1	4;2;2;2;1	4;2;2;2;1	Mixed lineag	MLKL;DKFZp6
Q8NB78	Q8NB78	1	1	1	Lysine-specif	KDM1B
Q8NB90;J3Q	Q8NB90	4;1;1;1;1;1;	4;1;1;1;1;1;	4;1;1;1;1;1;	Spermatoger	SPATA5
Q8NBI6;B4D	Q8NBI6	3;1	3;1	3;1	Xyloside xylo	XXYLT1
Q8NC51;Q63	Q8NC51;Q63	6;5;5;4;4	6;5;5;4;4	6;5;5;4;4	Plasminogen	SERBP1;DKFZ
Q8NCD3;C9J	Q8NCD3;C9J	4;3;2	4;3;2	4;3;2	Holliday junc	HJURP
Q8NCN4;H0Y	Q8NCN4	8;1	8;1	8;1	E3 ubiquitin-	RNF169
Q8NDX5;A5Y	Q8NDX5;A5Y	25;24;21;18;	22;21;21;18;	22;21;21;18;	Polyhomeoti	PHC3
Q8NDX6	Q8NDX6	6	6	6	Zinc finger pr	ZNF740
Q8NEP9	Q8NEP9	1	1	1	Zinc finger pr	ZNF555
Q8NFC6	Q8NFC6	2	2	2	Biorientation	BOD1L1
Q8NG31;E9P	Q8NG31;E9P	30;24;19;3;2	30;24;19;3;2	30;24;19;3;2	Protein CASC	CASC5
Q8TD26;B4D	Q8TD26	23;11;7;6	17;6;6;5	15;5;5;4	Chromodoma	CHD6
Q8TDD1;H0Y	Q8TDD1	6;1;1	6;1;1	6;1;1	ATP-depende	DDX54
Q8TDN6;A0J	Q8TDN6;A0J	10;7;6;4	10;7;6;4	10;7;6;4	Ribosome bio	BRIX1;BXDC2
Q8TE73	Q8TE73	1	1	1	Dynein heavy	DNAH5
Q8TEM1	Q8TEM1	2	2	2	Nuclear pore	NUP210
Q8WUA4;Q5	Q8WUA4;Q5	3;2;2;1;1;1	3;2;2;1;1;1	3;2;2;1;1;1	General trans	GTF3C2
S5FMB0;Q8V	S5FMB0;Q8V	7;7;6;6;4	7;7;6;6;4	7;7;6;6;4	PHD finger pr	PHF10
Q8WUU5	Q8WUU5	4	4	4	GATA zinc fin	GATAD1
Q8WX93;B2F	Q8WX93;B2F	24;20;8;6;6;2	24;20;8;6;6;2	23;19;7;5;5;2	Palladin	PALLD
Q8WXI9;B3K	Q8WXI9;B3K	6;5	6;5	6;5	Transcription	GATAD2B
Q8WYP5;B3K	Q8WYP5;B3K	76;49;38;31;	76;49;38;31;	76;49;38;31;	Protein ELYS	AHCTF1
Q92466;A0A	Q92466;A0A	2;1;1;1;1	2;1;1;1;1	2;1;1;1;1	DNA damage	DDB2
Q92522	Q92522	4	4	4	Histone H1x	H1FX
Q92576;E7E	Q92576;E7E	27;14;11;11;	27;14;11;11;	27;14;11;11;	PHD finger pr	PHF3
Q92769;B3K	Q92769;B3K	10;9;5;5;5;4;	10;9;5;5;5;4;	6;6;2;2;2;1;1;	Histone deac	HDAC2
Q92878;E7E	Q92878;E7E	8;6;6;5;4;1;1;	8;6;6;5;4;1;1;	8;6;6;5;4;1;1;	DNA repair p	RAD50
Q92945;A0A	Q92945;A0A	37;35;21;21;	37;35;21;21;	33;31;19;19;	Far upstream	KHSRP
Q93074;B4D	Q93074;B4D	5;4;2;2	5;4;2;2	5;4;2;2	Mediator of I	MED12
Q969H6	Q969H6	1	1	1	Ribonuclease	POP5
Q96AG4	Q96AG4	1	1	1	Leucine-rich	LRRC59
Q96AY2	Q96AY2	1	1	1	Crossover jur	EME1

Q96BN2	Q96BN2	2	2	2	Transcription	TADA1
Q96DY7	Q96DY7	4	4	4	Mdm2-bindir	MTBP
Q96EK4;B5AI	Q96EK4;B5AI	4;2	4;2	4;2	THAP domair	THAP11
Q99575;Q96I	Q99575;Q96I	14;14;5	14;14;5	14;14;5	Ribonuclease	POP1
Q96FQ6	Q96FQ6	3	3	3	Protein S100	S100A16
Q96GD4;J3Q	Q96GD4;J3Q	6;5;5;5;5;4;3;	6;5;5;5;5;4;3;	6;5;5;5;5;4;3;	Aurora kinas	AURKB
Q96GM8;B4I	Q96GM8;B4I	14;7;5	14;7;5	14;7;5	Target of EGI	TOE1
Q96H79	Q96H79	4	4	4	Zinc finger C(	ZC3HAV1L
Q96HA1;A8C	Q96HA1;A8C	6;5;5;4;2;1;1;	6;5;5;4;2;1;1;	6;5;5;4;2;1;1;	Nuclear enve	POM121;POI
Q96HS1;F5G	Q96HS1	8;2	8;2	8;2	Serine/threo	PGAM5
Q96IR2	Q96IR2	1	1	1	Zinc finger pr	ZNF845
Q96IZ0	Q96IZ0	1	1	1	PRKC apopto	PAWR
Q96JM3;S4R	Q96JM3	6;2	6;2	6;2	Chromosome	CHAMP1
Q96K97;Q9U	Q96K97;Q9U	2;2;1;1;1	2;2;1;1;1	2;2;1;1;1	Signal recogr	SRP68
Q96KM6	Q96KM6	4	4	4	Zinc finger pr	ZNF512B
Q96L91;A7E2	Q96L91;A7E2	15;8;2	15;8;2	15;8;2	E1A-binding	EP400
Q96MG7	Q96MG7	2	2	2	Melanoma-a	NDNL2
Q96PK6;Q59	Q96PK6	24;11;10;3;3;	24;11;10;3;3;	24;11;10;3;3;	RNA-binding	RBM14
Q96PU4;B1A	Q96PU4	3;1;1	2;1;1	2;1;1	E3 ubiquitin-	UHRF2
Q96PU8;F5H	Q96PU8;F5H	2;1;1;1;1;1;1;	2;1;1;1;1;1;1;	2;1;1;1;1;1;1;	Protein quak	QKI
Q96QE3;A0A	Q96QE3;A0A	22;19;15	22;19;15	22;19;15	ATPase famil	ATAD5
Q96SK2;C9J5	Q96SK2	3;1;1	3;1;1	3;1;1	Transmembr	TMEM209
Q96SL8;K7EC	Q96SL8	5;1;1;1;1	5;1;1;1;1	5;1;1;1;1	Flt3-interacti	FIZ1
Q96ST3;B3K(	Q96ST3;B3K(	8;4	8;4	8;4	Paired amphi	SIN3A
Q96T58;H0Y	Q96T58	16;1;1	16;1;1	16;1;1	Msx2-interac	SPEN
Q99496;X6RI	Q99496;X6RI	22;15	22;15	18;11	E3 ubiquitin-	RNF2
Q99549;H0Y(	Q99549	4;1;1;1	4;1;1;1	4;1;1;1	M-phase pho	MPHOSPH8
Q9BQ04	Q9BQ04	5	1	1	RNA-binding	RBM4B
Q9BQ61;B2R	Q9BQ61;B2R	6;5;3;2;2	6;5;3;2;2	6;5;3;2;2	Uncharacteri	C19orf43
Q9BQ69	Q9BQ69	3	3	3	O-acetyl-ADF	MACROD1
Q9BQG0;I3L1	Q9BQG0;I3L1	38;35;15;14;	38;35;15;14;	38;35;15;14;	Myb-binding	MYBBP1A
Q9BR63;Q9N	Q9BR63;Q9N	11;11;10;8	11;11;10;8	11;11;10;8	Phenylalanin	FARSB
Q9BRJ7;K7EI	Q9BRJ7;K7EI	12;11;11;8;7;	12;11;11;8;7;	12;11;11;8;7;	Protein synd	NUDT16L1
Q9BRP1;U3K	Q9BRP1	7;3;3	7;3;3	7;3;3	Programmed	PDCD2L
Q9BTC0	Q9BTC0	25	25	25	Death-induce	DIDO1
Q9BTL3	Q9BTL3	1	1	1	RNMT-activa	FAM103A1
Q9BU76	Q9BU76	10	10	10	Multiple mye	MMTAG2
Q9BUF5;B4D	Q9BUF5;B4D	16;15;14;12;	5;5;3;1;1;4;4;	3;3;2;1;1;2;2;	Tubulin beta	TUBB6
U3KQC1;Q9B	U3KQC1;Q9B	8;8;7;7;2;2;1;	8;8;7;7;2;2;1;	8;8;7;7;2;2;1;	WD repeat-c	WDR18
Q9BVJ6;B4E3	Q9BVJ6;B4E3	10;9;5;4;4;4;	10;9;5;4;4;4;	10;9;5;4;4;4;	U3 small nuc	UTP14A
Q9BVQ7;H0Y	Q9BVQ7	6;1	6;1	6;1	Spermatoger	SPATA5L1
Q9BWF3;E9P	Q9BWF3;E9P	7;6;6;4;4;3;3;	7;6;6;4;4;3;3;	3;2;2;1;1;0;0;	RNA-binding	RBM4
Q9BWJ5	Q9BWJ5	4	4	4	Splicing facto	SF3B5
Q9BXK1;D6W	Q9BXK1;D6W	4;3;1	4;3;1	4;3;1	Krueppel-like	KLF16
Q9BY42;A2A	Q9BY42;A2A	7;4;3	1;1;1	1;1;1	Protein RTF2	RTFDC1

Q9BY89	Q9BY89	18	18	18	Uncharacteri	KIAA1671
Q9BYB4;C9JF	Q9BYB4	6;2	6;2	6;2	Guanine nucl	GNB1L
Q9BYE7	Q9BYE7	2	2	2	Polycomb gr	PCGF6
Q9P1X0;Q9B	Q9P1X0;Q9B	2;2;2	2;2;2	2;2;2	Cystine/gluta	xCT;hxCT;SLC
Q9BYJ9;Q65	Q9BYJ9;Q65	5;4;4;4;4;4;	3;2;2;2;2;2;2;	3;2;2;2;2;2;2;	YTH domain	YTHDF1;DKFZ
Q9BYW2;C9J	Q9BYW2;C9J	25;20;20;18;	25;20;20;18;	25;20;20;18;	Histone-lysin	SETD2
Q9BZ95;E9PK	Q9BZ95	11;2;1	10;2;1	10;2;1	Histone-lysin	WHSC1L1
Q9C005;B4D	Q9C005;B4D	3;2	3;2	3;2	Protein dpy-3	DPY30;LOC84
Q9GZS1;B4E	Q9GZS1;B4E	5;3;2;2;2	5;3;2;2;2	5;3;2;2;2	DNA-directe	POLR1E
Q9H0A0;A0A	Q9H0A0;A0A	10;8;4;4;2	10;8;4;4;2	10;8;4;4;2	N-acetyltrans	NAT10
Q9H0C8;H7C	Q9H0C8;H7C	2;1;1;1	2;1;1;1	2;1;1;1	Integrin-linke	ILKAP;ILKAP3
Q9H147;H7C	Q9H147;H7C	7;6;6;3;2	7;6;6;3;2	7;6;6;3;2	Deoxynucleo	DNTTIP1
Q9H1A4;H0Y	Q9H1A4;H0Y	3;2;1	3;2;1	3;2;1	Anaphase-pr	ANAPC1
Q9H3K6;H3B	Q9H3K6	5;2;2;2;2	5;2;2;2;2	5;2;2;2;2	BolA-like pro	BOLA2
Q9H4L4;A0A	Q9H4L4;A0A	9;8;5;5;4;2	9;8;5;5;4;2	9;8;5;5;4;2	Sentrin-speci	SENP3
Q9H4Z2;Q8I\	Q9H4Z2;Q8I\	3;2	3;2	3;2	Zinc finger pr	ZNF335
Q9H501;A0A	Q9H501;A0A	3;2;2;2	3;2;2;2	3;2;2;2	ESF1 homolo	ESF1
Q9H582;A7E	Q9H582;A7E	43;42;24;24;	43;42;24;24;	43;42;24;24;	Zinc finger pr	ZNF644
Q9H5V9;D3D	Q9H5V9	3;1	3;1	3;1	UPF0428 pro	CXorf56
Q9H6F5	Q9H6F5	2	2	2	Coiled-coil d	CCDC86
Q9H6R4;A0A	Q9H6R4;A0A	25;23;23;13;	25;23;23;13;	25;23;23;13;	Nucleolar pro	NOL6
Q9H7E9	Q9H7E9	3	3	3	UPF0488 pro	C8orf33
Q9H7L9	Q9H7L9	1	1	1	Sin3 histone	SUDS3
Q9H7Z6	Q9H7Z6	1	1	1	Histone acet	KAT8
Q9H8M2;Q8I	Q9H8M2	8;2;1;1;1;1	8;2;1;1;1;1	8;2;1;1;1;1	Bromodomai	BRD9
Q9H9B1;A0A	Q9H9B1;A0A	40;24;13;12;	40;24;13;12;	39;23;13;12;	Histone-lysin	EHMT1
Q9H9L3	Q9H9L3	8	8	8	Interferon-st	ISG20L2
Q9HA92;B4D	Q9HA92;B4D	3;2;1;1	3;2;1;1	3;2;1;1	Radical S-ade	RSAD1
Q9HC52;C9J	Q9HC52;C9J	21;11;5;1	21;11;5;1	20;11;5;0	Chromobox p	CBX8
Q9HCD6;A0A	Q9HCD6;A0A	3;2;2;1	3;2;2;1	3;2;2;1	Protein TANC	TANC2
Q9HCI7;A8K	Q9HCI7;A8K	6;5;1;1;1	6;5;1;1;1	6;5;1;1;1	E3 ubiquitin-	MSL2
Q9HCK8;H0Y	Q9HCK8;H0Y	11;9	1;0	1;0	Chromodoma	CHD8
Q9NPC8;Q8T	Q9NPC8;Q8T	3;2;1	3;2;1	3;2;1	Homeobox p	SIX2
Q9NPD3;E9P	Q9NPD3;E9P	4;2;2	4;2;2	4;2;2	Exosome con	EXOSC4
Q9NQS7	Q9NQS7	1	1	1	Inner centror	INCENP
Q9NQT5;Q9	Q9NQT5;Q9	2;1	2;1	2;1	Exosome con	EXOSC3;PNA
Q9NR12;D6R	Q9NR12;D6R	2;1;1;1	2;1;1;1	2;1;1;1	PDZ and LIM	PDLIM7
Q9NR30;Q3S	Q9NR30	36;3;3	36;3;3	34;3;3	Nucleolar RN	DDX21
Q9NR48;F8V	Q9NR48	15;1	15;1	15;1	Histone-lysin	ASH1L
Q9NRE2	Q9NRE2	1	1	1	Teashirt hom	TSHZ2
Q9NSI2	Q9NSI2	2	2	2	Protein FAM	FAM207A
Q9NU22;Q5T	Q9NU22	31;15;8;8;1	31;15;8;8;1	31;15;8;8;1	Midasin	MDN1
Q9NUI1;Q4V	Q9NUI1;Q4V	8;7;5;2;2;1;1	8;7;5;2;2;1;1	8;7;5;2;2;1;1	Peroxisomal	DECR2
Q9NV56	Q9NV56	2	2	2	MRG/MORF4	MRGBP
Q9NVV4	Q9NVV4	3	3	3	Poly(A) RNA	MTPAP

Q9NVW2	Q9NVW2	3	3	3	E3 ubiquitin-	RLIM
Q9NWH9;H7	Q9NWH9;H7	6;4;4;4;3;2;2;	6;4;4;4;3;2;2;	6;4;4;4;3;2;2;	SAFB-like tra	SLTM
Q9NWT1	Q9NWT1	1	1	1	p21-activate	PAK1IP1
Q9NXV6;B3K	Q9NXV6	5;2;1;1	5;2;1;1	5;2;1;1	CDKN2A-inte	CDKN2AIP
Q9NXW2;J3K	Q9NXW2;J3K	3;2;1;1;1	3;2;1;1;1	3;2;1;1;1	DnaJ homolo	DNAJB12
Q9NXX6	Q9NXX6	1	1	1	Non-structur	NSMCE4A
Q9NY61;A0A	Q9NY61	5;1;1	5;1;1	5;1;1	Protein AATF	AATF
Q9NY74	Q9NY74	6	6	6	Ewings tumo	ETAA1
Q9NYF8;E9PI	Q9NYF8;E9PI	19;16;15;15;	18;16;15;15;	18;16;15;15;	Bcl-2-associa	BCLAF1
Q9NYP9	Q9NYP9	2	2	2	Protein Mis1	MIS18A
Q9NZB2;A0A	Q9NZB2;A0A	2;1	2;1	2;1	Constitutive	FAM120A
Q9NZT1;Q53	Q9NZT1;Q53	2;1	2;1	2;1	Calmodulin-li	CALML5
Q9P0U3;F8V	Q9P0U3	12;4;2;2;2	12;4;2;2;2	12;4;2;2;2	Sentrin-speci	SENP1
Q9UBD5;B7Z	Q9UBD5;B7Z	5;4;4;4;3;2	5;4;4;4;3;2	5;4;4;4;3;2	Origin recogr	ORC3;ORC3L
Q9UBL6;H0Y	Q9UBL6;H0Y	4;2;1;1;1;1;1;	4;2;1;1;1;1;1;	4;2;1;1;1;1;1;	Copine-7	CPNE7
Q9UBS4;B3K	Q9UBS4;B3K	2;1	2;1	2;1	DnaJ homolo	DNAJB11
Q9UBV8	Q9UBV8	5	5	5	Peflin	PEF1
Q9UEG4	Q9UEG4	2	2	2	Zinc finger pr	ZNF629
Q9UFC0;H7C	Q9UFC0	7;3;3;2;2	7;3;3;2;2	7;3;3;2;2	Leucine-rich	LRWD1
Q9UGM3	Q9UGM3	1	1	1	Deleted in m	DMBT1
Q9UHF7;H0Y	Q9UHF7;H0Y	2;1;1;1	2;1;1;1	2;1;1;1	Zinc finger tr	TRPS1
Q9UHR4	Q9UHR4	1	1	1	Brain-specific	BAIAP2L1
Q9UIG0	Q9UIG0	13	13	13	Tyrosine-pro	BAZ1B
Q9UK61	Q9UK61	20	20	3	Protein FAM:	FAM208A
Q9UKJ3;K7Ek	Q9UKJ3	6;1	6;1	6;1	G patch dom	GPATCH8
Q9UKN8;Q0!	Q9UKN8;Q0!	4;3;2;1	4;3;2;1	4;3;2;1	General trans	GTF3C4
Q9ULL5	Q9ULL5	8	8	8	Proline-rich p	PRR12
Q9ULX6;B3K	Q9ULX6;B3K	12;6;6;2;2;2;	12;6;6;2;2;2;	12;6;6;2;2;2;	A-kinase ancl	AKAP8L
Q9UMS4;F5G	Q9UMS4	14;6;2;2;2	14;6;2;2;2	14;6;2;2;2	Pre-mRNA-pr	PRPF19
Q9UQ35;O6(	Q9UQ35;O6(	31;27;10;4;3;	31;27;10;4;3;	31;27;10;4;3;	Serine/argini	SRRM2;KIAA(
Q9Y230;B3K	Q9Y230;B3K	20;18;13;7;6;	20;18;13;7;6;	20;18;13;7;6;	RuvB-like 2	RUVBL2
Q9Y232	Q9Y232	1	1	1	Chromodoma	CDYL
Q9Y2F5;A0A(	Q9Y2F5	8;1	8;1	8;1	Uncharacteri	KIAA0947
Q9Y2K1;Q5U	Q9Y2K1;Q5U	4;3	4;3	4;3	Zinc finger ar	ZBTB1
Q9Y2R4;Q59	Q9Y2R4;Q59	16;13;12;3;3;	16;13;12;3;3;	16;13;12;3;3;	Probable ATF	DDX52
Q9Y2S7;B4DI	Q9Y2S7;B4DI	13;11;1	13;11;1	13;11;1	Polymerase c	POLDIP2
Q9Y2W1;Q7Z	Q9Y2W1	22;6;6;6;6	22;6;6;6;6	21;6;6;6;6	Thyroid horm	THRAP3
Q9Y2X3;B3K	Q9Y2X3;B3K	19;16;6;4;2	19;16;6;4;2	19;16;6;4;2	Nucleolar pro	NOP58
Q9Y2X9;B4DI	Q9Y2X9;B4DI	27;19;15;15	27;19;15;15	27;19;15;15	Zinc finger pr	ZNF281
Q9Y383;B3K!	Q9Y383;B3K!	17;16;15;15;	17;16;15;15;	10;9;9;9;8;1;	Putative RNA	LUC7L2
Q9Y3A2	Q9Y3A2	1	1	1	Probable U3	UTP11L
Q9Y3A4;Q9N	Q9Y3A4	10;3;1	10;3;1	10;3;1	Ribosomal RI	RRP7A
Q9Y3B4	Q9Y3B4	2	2	2	Pre-mRNA br	SF3B14
Q9Y3I0;B4DX	Q9Y3I0;B4DX	3;2;2;2	3;2;2;2	3;2;2;2	tRNA-splicing	C22orf28
Q9Y3U8;J3Q!	Q9Y3U8	3;1	3;1	3;1	60S ribosoma	RPL36

X6R700;Q9Y3	X6R700;Q9Y3	3;3;2;1	3;3;2;1	3;3;2;1	Chromatin ta	CHTOP
Q9Y446;E9PK	Q9Y446	11;3;2;1;1;1;	11;3;2;1;1;1;	11;3;2;1;1;1;	Plakophilin-3	PKP3
Q9Y4B5;J3QL	Q9Y4B5	24;8;1;1;1;1;	24;8;1;1;1;1;	24;8;1;1;1;1;	Protein SOGA	SOGA2
Q9Y4E5;Q4KI	Q9Y4E5;Q4KI	4;3;3;2;1	4;3;3;2;1	4;3;3;2;1	Zinc finger pr	ZNF451
Q9Y4W2;B7Z	Q9Y4W2	18;8;6;4;2	18;8;6;4;2	18;8;6;4;2	Ribosomal bi	LAS1L
Q9Y5B9;Q0V	Q9Y5B9	21;4;1;1;1	21;4;1;1;1	21;4;1;1;1	FACT comple	SUPT16H
Q9Y5Q8;H7B	Q9Y5Q8;H7B	6;5;5;5;4;4;2;	6;5;5;5;4;4;2;	6;5;5;5;4;4;2;	General tran	GTF3C5
Q9Y5Q9;F8W	Q9Y5Q9	6;2;2;1	6;2;2;1	6;2;2;1	General tran	GTF3C3
Q9Y6A4	Q9Y6A4	1	1	1	UPF0468 pro	C16orf80
REV__A0A07	REV__A0A07	1;1	1;1	1;1		
REV__CON__	REV__CON__	1;1;1;1;1;1;1;	1;1;1;1;1;1;1;	1;1;1;1;1;1;1;1;1;1;1;1		
REV__A0A0A	REV__A0A0A	1;1	1;1	1;1		
REV__A1L45	REV__A1L45	1	1	1		
REV__A7MD	REV__A7MD	1	1	1		
REV__Q2KHI	REV__Q2KHI	2;2;2;2;1;1;1	2;2;2;2;1;1;1	2;2;2;2;1;1;1		
REV__B4E2A	REV__B4E2A	1;1;1;1;1	1;1;1;1;1	1;1;1;1;1		
REV__Q9Y4T	REV__Q9Y4T	1;1;1;1;1;1;1;	1;1;1;1;1;1;1;	1;1;1;1;1;1;1;1;1;1		
REV__E9PG8	REV__E9PG8	1;1	1;1	1;1		
REV__H0YKZ	REV__H0YKZ	1;1;1	1;1;1	1;1;1		
REV__Q59H4	REV__Q59H4	1;1	1;1	1;1		
REV__P6060	REV__P6060	1	1	1		
REV__Q5UW	REV__Q5UW	1;1	1;1	1;1		
REV__Q8N93	REV__Q8N93	1	1	1		
REV__Q9H7N	REV__Q9H7N	1	1	1		
REV__Q9NU	REV__Q9NU	1	1	1		
REV__Q9P21	REV__Q9P21	1	1	1		
S4R341	S4R341	3	1	1		
S4R3H4;E7EC	S4R3H4;E7EC	14;13;13;12;	14;13;13;12;	14;13;13;12;	Apoptotic ch	ACIN1;DKFZp
X6RAL5;O00	X6RAL5;O00	8;7;5;2;1	8;7;5;2;1	8;7;5;2;1	Histone deac	SAP18

Fasta headers	Number of pı	Peptides	Razor + uniq	Unique pepti	Peptides CBX
>tr|F5H6N1|F5H6N1_HU	7	5	5	5	4
>sp|Q9Y5S9|RBM8A_HUI	4	4	4	4	3
>sp|P98179|RBM3_HUM	2	2	2	2	2
>tr|B3KNA1|B3KNA1_HU	19	10	10	10	5
>tr|A0A087X1I3|A0A087X	9	5	5	5	5
>tr|A0A087WVT1|A0A08	3	3	3	3	2
>tr|B3KTZ7|B3KTZ7_HUN	4	1	1	1	1
>tr|Q0QEW2|Q0QEW2_H	10	6	6	6	6
>sp|P08621|RU17_HUMA	6	7	7	7	5
>tr|M0QXN5|M0QXN5_H	3	2	2	2	2
>sp|Q8IV63|VRK3_HUMA	12	4	4	4	2
>tr|A0A024QZJ8|A0A024	7	3	2	2	2
>tr|Q53F48|Q53F48_HUI	5	5	5	5	4
>tr|B4DSN3|B4DSN3_HU	4	2	2	2	1
>sp|O95229|ZWINT_HUN	5	3	3	3	3
>tr|B4DKM5|B4DKM5_HI	5	1	1	1	1
>sp|Q9BRX2|PELO_HUM	3	11	11	11	9
>tr|H7C2B1|H7C2B1_HUI	3	1	1	1	1
>sp|P49790|NU153_HUN	2	25	25	25	22
>sp|Q13523|PRP4B_HUN	5	7	7	7	5
>sp|Q99880|H2B1L_HUN	26	6	6	6	5
>sp|Q93077|H2A1C_HUN	5	4	1	1	4
>tr|E9PF10|E9PF10_HUN	5	18	18	18	16
>tr|A0A024R0A9|A0A024	5	2	2	2	2
>tr|B3KQ42|B3KQ42_HU	7	9	9	9	9
>tr|C9JUG7|C9JUG7_HUI	10	1	1	1	1
>sp|Q8N7H5|PAF1_HUM	3	8	8	8	8
>tr|B4DJP0|B4DJP0_HUN	3	1	1	1	1
>tr|A8K3W4|A8K3W4_HI	13	6	6	6	4
>tr|B7Z6N1|B7Z6N1_HUI	3	8	8	8	8
>sp|Q3ZCQ8|TIM50_HUN	13	9	9	9	9
>sp|Q14588|ZN234_HUN	2	2	1	1	2
>sp|Q9NZL3|ZN224_HUN	90	2	1	1	2
>sp|Q8IV08|PLD3_HUMA	5	2	2	2	2
>tr|B3KY11|B3KY11_HUN	4	3	3	3	2
>tr|F8VZ70|F8VZ70_HUN	7	1	1	1	1
>sp|Q12800|TFCP2_HUN	7	5	5	4	5
>tr|Q8N3V2|Q8N3V2_HL	11	2	2	2	2
>tr|H0YID1|H0YID1_HUN	7	1	1	1	1
>sp|Q9H0H5|RGAP1_HUI	19	5	5	5	4
>sp|Q9Y2D5|AKAP2_HUN	4	1	1	1	1
>sp|O95347|SMC2_HUM	16	16	16	16	15
>tr|B2R923|B2R923_HUN	3	2	2	2	1
>sp|O95758|PTBP3_HUN	3	7	5	5	3

>sp|Q9NXF1|TEX10_HUM	5	7	7	7	7
>sp|Q09161|NCBP1_HUN	3	2	2	2	1
>sp|Q13356|PPIL2_HUM.	4	3	3	3	2
>tr|A0A087WWW8|A0A0	8	2	2	2	2
>tr|Q9HD72|Q9HD72_HU	8	9	8	8	9
>tr|A0A024R1J3|A0A024	2	2	2	2	2
>sp|Q04917|1433F_HUM	36	3	1	1	2
>sp|Q15459|SF3A1_HUM	3	3	3	3	1
>tr|B4DE32|B4DE32_HUI	7	6	6	6	4
>sp|Q9UH99|SUN2_HUM	12	4	4	4	4
>tr|Q9BTQ7|Q9BTQ7_HU	8	7	7	7	6
>sp|Q99741|CDC6_HUM.	3	2	2	2	2
>tr|J3QKS7|J3QKS7_HUM	9	7	7	7	7
>tr|F8WFE7|F8WFE7_HU	3	1	1	1	1
>Q92764 SWISS-PROT:Q9	5	15	10	3	1
>tr|A0A024R1Y2|A0A024	6	3	3	3	2
>sp|P46060|RAGP1_HUM	6	9	9	9	4
>sp|Q7RTV0|PHF5A_HUM	2	4	4	4	4
>tr|E9PC22|E9PC22_HUM	22	1	1	1	1
>tr|E4W6B6|E4W6B6_HU	8	8	8	8	8
>sp|P35227|PCGF2_HUM	4	16	16	13	16
>sp|P14923|PLAK_HUMA	2	9	5	4	5
>tr|B3KVB0|B3KVB0_HUI	4	1	1	1	1
>tr|J7FRP4|J7FRP4_HUM	10	1	1	1	1
>tr|K7EIY6|K7EIY6_HUM	8	4	4	4	4
>sp|P61978|HNRPK_HUM	10	24	24	24	18
>sp|Q16649|NFIL3_HUM	3	2	2	2	2
>sp|Q76FK4|NOL8_HUM.	16	10	10	10	8
>tr|A0A087WXM6|A0A08	14	5	5	5	5
>tr|K7EL15|K7EL15_HUM	10	1	1	1	1
>sp|O15269|SPTC1_HUM	3	2	2	2	2
>tr|A0A024R2D7|A0A024	4	2	2	2	2
>sp|Q9BXW9|FACD2_HU	4	9	9	9	9
>tr|D3YTB1|D3YTB1_HUI	5	6	6	6	6
>tr|Q53H10|Q53H10_HU	4	5	5	5	5
>tr|C9JAW5|C9JAW5_HU	5	1	1	1	1
>tr|C9J9K3|C9J9K3_HUM	9	6	6	6	5
>sp|Q15024|EXOS7_HUM	4	5	5	5	3
>tr|B4DSW9|B4DSW9_HU	7	3	2	2	3
>sp|P61313|RL15_HUMA	10	8	8	8	7
>tr|A0A0D9SEH2|A0A0D9	4	2	2	2	2
>sp|P52756|RBM5_HUM	11	5	5	5	5
>tr|A0A024R2Z6|A0A024	2	6	6	6	6
>tr|F8W6N3|F8W6N3_HU	4	2	2	2	2
>tr|A0A024R326|A0A024	3	2	2	2	2

<table>
<tr><td>>tr|Q9BS79|Q9BS79_HUI</td><td>8</td><td>2</td><td>2</td><td>2</td><td>0</td></tr>
<tr><td>>sp|Q9BTE7|DCNL5_HUN</td><td>10</td><td>4</td><td>4</td><td>4</td><td>3</td></tr>
<tr><td>>sp|Q9BRL6|SRSF8_HUM</td><td>2</td><td>4</td><td>2</td><td>2</td><td>4</td></tr>
<tr><td>>tr|A0A024R3B4|A0A024</td><td>3</td><td>2</td><td>2</td><td>2</td><td>1</td></tr>
<tr><td>>sp|Q13315|ATM_HUMA</td><td>11</td><td>8</td><td>8</td><td>8</td><td>7</td></tr>
<tr><td>>sp|Q9NVF7|FBX28_HUN</td><td>2</td><td>1</td><td>1</td><td>1</td><td>1</td></tr>
<tr><td>>sp|P09874|PARP1_HUN</td><td>9</td><td>21</td><td>21</td><td>21</td><td>16</td></tr>
<tr><td>>sp|Q8WWC4|CB047_HU</td><td>2</td><td>1</td><td>1</td><td>1</td><td>1</td></tr>
<tr><td>>tr|A0A0A0MSG5|A0A0A</td><td>4</td><td>4</td><td>4</td><td>4</td><td>3</td></tr>
<tr><td>>sp|Q9H2H8|PPIL3_HUN</td><td>6</td><td>5</td><td>5</td><td>5</td><td>5</td></tr>
<tr><td>>sp|P24534|EF1B_HUMA</td><td>6</td><td>2</td><td>2</td><td>2</td><td>2</td></tr>
<tr><td>>sp|P10809|CH60_HUMA</td><td>16</td><td>13</td><td>13</td><td>13</td><td>13</td></tr>
<tr><td>>tr|B8ZZJ0|B8ZZJ0_HUM</td><td>6</td><td>2</td><td>2</td><td>2</td><td>2</td></tr>
<tr><td>>tr|E9PHS0|E9PHS0_HUN</td><td>8</td><td>5</td><td>5</td><td>5</td><td>3</td></tr>
<tr><td>>sp|Q13416|ORC2_HUM</td><td>5</td><td>4</td><td>4</td><td>4</td><td>3</td></tr>
<tr><td>>tr|Q5R210|Q5R210_HU</td><td>13</td><td>28</td><td>28</td><td>28</td><td>27</td></tr>
<tr><td>>tr|B3KN05|B3KN05_HUI</td><td>6</td><td>2</td><td>2</td><td>2</td><td>2</td></tr>
<tr><td>>sp|P19338|NUCL_HUMA</td><td>11</td><td>20</td><td>20</td><td>20</td><td>10</td></tr>
<tr><td>>tr|A0A0A0N0L3|A0A0A0</td><td>24</td><td>7</td><td>7</td><td>7</td><td>6</td></tr>
<tr><td>>tr|K7EM90|K7EM90_HU</td><td>15</td><td>2</td><td>2</td><td>2</td><td>0</td></tr>
<tr><td>>tr|Q0QF37|Q0QF37_HU</td><td>7</td><td>5</td><td>5</td><td>5</td><td>5</td></tr>
<tr><td>>sp|P46781|RS9_HUMAN</td><td>8</td><td>6</td><td>6</td><td>6</td><td>6</td></tr>
<tr><td>>tr|M0R0F0|M0R0F0_HU</td><td>7</td><td>8</td><td>8</td><td>8</td><td>6</td></tr>
<tr><td>>sp|P61081|UBC12_HUN</td><td>3</td><td>2</td><td>2</td><td>2</td><td>2</td></tr>
<tr><td>>tr|A0A024R4V8|A0A024</td><td>3</td><td>4</td><td>4</td><td>4</td><td>4</td></tr>
<tr><td>>sp|Q9UKX7|NUP50_HUI</td><td>2</td><td>2</td><td>2</td><td>2</td><td>2</td></tr>
<tr><td>>tr|A0A0A6YYC7|A0A0A6</td><td>6</td><td>2</td><td>2</td><td>2</td><td>2</td></tr>
<tr><td>>sp|Q08945|SSRP1_HUN</td><td>5</td><td>12</td><td>12</td><td>12</td><td>12</td></tr>
<tr><td>>sp|O94776|MTA2_HUM</td><td>2</td><td>16</td><td>16</td><td>13</td><td>16</td></tr>
<tr><td>>tr|A0A024R566|A0A024</td><td>11</td><td>15</td><td>15</td><td>12</td><td>14</td></tr>
<tr><td>>tr|Q9UE24|Q9UE24_HU</td><td>7</td><td>3</td><td>3</td><td>3</td><td>3</td></tr>
<tr><td>>sp|Q92785|REQU_HUM</td><td>4</td><td>3</td><td>3</td><td>3</td><td>2</td></tr>
<tr><td>>sp|Q9Y6J9|TAF6L_HUM</td><td>5</td><td>2</td><td>2</td><td>2</td><td>2</td></tr>
<tr><td>>tr|Q9H880|Q9H880_HU</td><td>11</td><td>1</td><td>1</td><td>1</td><td>1</td></tr>
<tr><td>>sp|P50416|CPT1A_HUN</td><td>3</td><td>2</td><td>2</td><td>2</td><td>1</td></tr>
<tr><td>>tr|X5DNI9|X5DNI9_HUN</td><td>13</td><td>2</td><td>2</td><td>2</td><td>2</td></tr>
<tr><td>>tr|C9JB90|C9JB90_HUM</td><td>12</td><td>1</td><td>1</td><td>1</td><td>1</td></tr>
<tr><td>>tr|E9PQ34|E9PQ34_HUI</td><td>14</td><td>1</td><td>1</td><td>1</td><td>1</td></tr>
<tr><td>>tr|A0A024R5M9|A0A02</td><td>7</td><td>134</td><td>134</td><td>2</td><td>126</td></tr>
<tr><td>>tr|A0A024R5R8|A0A024</td><td>2</td><td>2</td><td>2</td><td>2</td><td>0</td></tr>
<tr><td>>tr|B4DGI9|B4DGI9_HUN</td><td>20</td><td>4</td><td>4</td><td>4</td><td>4</td></tr>
<tr><td>>tr|H3BMQ2|H3BMQ2_H</td><td>4</td><td>1</td><td>1</td><td>1</td><td>1</td></tr>
<tr><td>>tr|H0YL80|H0YL80_HUN</td><td>44</td><td>1</td><td>1</td><td>1</td><td>1</td></tr>
<tr><td>>tr|V9HW65|V9HW65_H</td><td>28</td><td>19</td><td>19</td><td>19</td><td>19</td></tr>
<tr><td>>tr|A0A024R609|A0A024</td><td>21</td><td>2</td><td>2</td><td>2</td><td>1</td></tr>
</table>

>tr|Q7Z612|Q7Z612_HUI	5	1	1	1	1
>sp|Q6PJG2|EMSA1_HUI	6	14	14	14	12
>sp|P84090|ERH_HUMAI	3	6	6	6	5
>sp|Q13243|SRSF5_HUM	4	4	3	3	2
>tr|B3KMI0|B3KMI0_HUI	5	2	2	2	2
>tr|B4DRJ7|B4DRJ7_HUM	7	1	1	1	1
>tr|A0A024R6Q8|A0A024	2	2	2	2	2
>sp|O60884|DNJA2_HUM	6	8	8	8	8
>tr|Q8WVB5|Q8WVB5_H	9	1	1	1	1
>tr|Q5HYH5|Q5HYH5_HU	4	2	2	2	1
>sp|Q13951|PEBB_HUMA	7	4	4	4	4
>tr|A0A024R734|A0A024	3	3	3	3	3
>sp|Q9NW13|RBM28_HU	6	4	4	4	3
>tr|Q6IAW5|Q6IAW5_HU	7	3	3	3	3
>tr|A0A024R7C4|A0A024	7	3	3	3	1
>tr|K7EKI4|K7EKI4_HUMA	7	1	1	1	1
>sp|Q12906|ILF3_HUMA	18	18	18	2	12
>sp|Q96RE7|NACC1_HUI	4	2	2	2	2
>sp|P26358|DNMT1_HUI	8	28	28	4	23
>sp|Q8IX01|SUGP2_HUM	8	26	26	26	23
>tr|B4DKZ7|B4DKZ7_HUI	5	3	3	3	3
>tr|A0A024R7N3|A0A024	4	1	1	1	1
>sp|Q96RL1|UIMC1_HUM	8	6	6	6	5
>sp|P52597|HNRPF_HUM	3	13	11	11	12
>sp|P17026|ZNF22_HUM	2	2	1	1	2
>tr|B4DLA6|B4DLA6_HUI	7	4	4	4	4
>sp|Q9P2D1|CHD7_HUM	3	26	17	15	24
>tr|R4GMY8|R4GMY8_HU	4	2	2	2	1
>sp|P18124|RL7_HUMAN	5	9	9	5	7
>sp|Q8NHZ8|CDC26_HUI	2	1	1	1	1
>tr|A0A024R856|A0A024	4	3	3	3	2
>sp|O00743|PPP6_HUMA	3	2	2	2	2
>tr|F2Z388|F2Z388_HUM	4	2	2	2	2
>tr|H0Y5D5|H0Y5D5_HU	10	6	6	6	6
>tr|A0A024R889|A0A024	8	3	3	3	1
>tr|A0A0C4DFV9|A0A0C4	7	2	2	2	2
>tr|Q5TBH0|Q5TBH0_HU	10	1	1	1	1
>sp|Q96I24|FUBP3_HUM	2	11	10	9	10
>tr|Q7Z3C4|Q7Z3C4_HUI	11	8	8	8	8
>sp|Q15361|TTF1_HUMA	4	3	3	3	3
>sp|Q9BV86|NTM1A_HU	4	2	2	2	1
>sp|P04183|KITH_HUMA	7	2	2	2	0
>tr|J3KT73|J3KT73_HUM	5	4	4	4	4
>sp|P61956|SUMO2_HUI	6	3	3	3	0
>tr|B4DNL5|B4DNL5_HU	18	13	13	13	11

>tr|J3KP15|J3KP15_HUM	14	9	9	7	7
>sp|Q9P275|UBP36_HUN	16	14	14	14	6
>sp|P38919|IF4A3_HUMA	14	7	7	6	5
>tr|A0A0C4DG88|A0A0C4	4	2	2	2	2
>tr|A0A087WYY6|A0A08	5	3	3	3	0
>sp|Q15291|RBBP5_HUN	4	5	5	5	5
>tr|A0A024R9E2|A0A024	39	11	11	11	11
>sp|P62888|RL30_HUMA	6	10	10	10	9
>tr|E5RHX8|E5RHX8_HUI	7	2	2	2	2
>sp|Q6PL18|ATAD2_HUN	3	6	6	6	6
>sp|O60216|RAD21_HUN	10	8	8	8	7
>sp|Q96QD9|UIF_HUMA	3	2	2	2	2
>tr|B4E1N7|B4E1N7_HUI	57	5	5	5	5
>tr|H0YLY7|H0YLY7_HUN	7	1	1	1	1
>sp|Q9H2Y7|ZN106_HUN	10	17	17	17	17
>tr|D6RAA6|D6RAA6_HU	4	1	1	1	1
>tr|Q05DU1|Q05DU1_HU	3	10	10	10	9
>sp|O00483|NDUA4_HUI	2	2	2	2	2
>sp|P35869|AHR_HUMAI	2	2	2	2	2
>tr|A8K5D9|A8K5D9_HUI	10	25	25	25	24
>sp|Q96GN5|CDA7L_HUI	8	10	10	10	9
>sp|P22626|ROA2_HUMA	6	20	20	20	17
>sp|Q86V48|LUZP1_HUN	2	2	2	2	2
>sp|Q9P258|RCC2_HUMA	3	27	27	27	23
>tr|A0A0C4DGS1|A0A0C4	4	2	2	2	2
>sp|Q9NVP1|DDX18_HUI	6	10	10	10	8
>tr|B7Z8S8|B7Z8S8_HUN	28	2	2	2	0
>sp|Q99878|H2A1J_HUN	14	5	5	2	5
>sp|Q9H0S4|DDX47_HUN	8	11	11	11	6
>tr|F8VZY9|F8VZY9_HUN	7	12	11	10	11
>tr|F8W6I7|F8W6I7_HUN	18	15	15	15	10
>tr|E7ESI2|E7ESI2_HUMA	11	6	6	5	5
>sp|P62854|RS26_HUMA	6	3	3	3	3
>tr|Q59G16|Q59G16_HU	7	16	16	8	15
>sp|Q15185|TEBP_HUMA	4	1	1	1	0
>tr|F8W1N5|F8W1N5_HU	9	1	1	1	1
>sp|Q9UL25|RAB21_HUN	3	2	2	2	2
>sp|Q16527|CSRP2_HUN	4	12	12	12	12
>sp|Q9Y2U8|MAN1_HUN	3	4	4	4	2
>sp|P42167|LAP2B_HUM	7	14	5	5	12
>tr|Q8NCF7|Q8NCF7_HU	13	9	9	9	9
>tr|H0YHI8|H0YHI8_HUN	4	1	1	1	0
>sp|Q9ULV4|COR1C_HUI	21	7	7	7	5
>tr|B3KMP2|B3KMP2_HU	7	1	1	1	1
>tr|Q8TBK5|Q8TBK5_HU	13	12	12	12	11

>tr|Q9UPN1|Q9UPN1_HU	7	6	6	2	6
>tr|F8VVL1|F8VVL1_HUM	3	3	3	3	2
>sp|Q8N3J9|ZN664_HUM	3	3	2	2	2
>tr|Q53HW2|Q53HW2_H	20	5	5	5	5
>tr|C9J7T7|C9J7T7_HUM	10	10	10	10	10
>tr|Q53HK3|Q53HK3_HU	8	6	6	6	6
>sp|P53701|CCHL_HUMA	3	4	4	4	2
>sp|Q15555|MARE2_HUI	2	1	1	1	1
>sp|P17028|ZNF24_HUM	2	3	3	2	2
>tr|Q6P1N4|Q6P1N4_HU	10	4	4	4	0
>sp|O43663|PRC1_HUMA	7	7	7	7	7
>tr|A0A0J9YX88|A0A0J9Y	9	2	2	2	1
>sp|P05387|RLA2_HUMA	3	2	2	2	0
>sp|Q7L311|ARMX2_HUI	8	4	4	4	4
>sp|O43309|ZSC12_HUM	3	2	2	1	1
>tr|Q59HG5|Q59HG5_HU	5	8	8	8	7
>tr|C9J423|C9J423_HUM	8	4	4	3	3
>sp|Q13838|DX39B_HUM	34	6	6	6	4
>tr|B4DMT1|B4DMT1_HU	8	2	2	2	2
>tr|A0A0G2JIS2|A0A0G2J	12	38	37	37	38
>tr|A0A024RCR6|A0A024	19	6	6	6	6
>tr|B4E063|B4E063_HUM	6	11	11	11	10
>sp|P17096|HMGA1_HU	3	1	1	1	0
>tr|B4DZZ8|B4DZZ8_HUM	5	1	1	1	1
>sp|Q9Y3C6|PPIL1_HUM	2	1	1	1	1
>tr|C9J7M8|C9J7M8_HUI	4	1	1	1	1
>tr|A8K2K2|A8K2K2_HUM	8	2	2	2	1
>tr|A0A0A0MTI6|A0A0A0	5	1	1	1	1
>sp|O75376|NCOR1_HUI	22	7	7	7	7
>tr|B3KXK2|B3KXK2_HUM	4	2	2	2	1
>tr|Q6PK50|Q6PK50_HUI	5	2	1	1	2
>tr|A0A024RDA6|A0A024	2	2	2	2	2
>sp|Q9UNQ0|ABCG2_HU	10	5	5	5	0
>tr|A0A024RDF4|A0A024	13	11	11	10	10
>tr|B4DTA2|B4DTA2_HU	4	3	2	2	3
>sp|P49207|RL34_HUMA	3	2	2	2	1
>sp|Q9NY12|GAR1_HUM	2	2	2	2	1
>tr|A0A087WXN4|A0A08	8	2	2	2	2
>tr|A1JUI8|A1JUI8_HUM	7	3	3	3	3
>tr|A0A024RDP4|A0A024	3	1	1	1	1
>tr|B4DKE6|B4DKE6_HUI	6	12	12	12	11
>tr|Q5T7C4|Q5T7C4_HUI	12	4	4	3	0
>sp|Q9UBW7|ZMYM2_H	5	13	13	13	13
>tr|R4GND2|R4GND2_HU	7	1	1	1	1
>tr|B4E3J6|B4E3J6_HUM	3	4	4	4	2

	>tr|B3KWG6|B3KWG6_H	6	4	4	4	4
5CHJ2;X4YT82;X2J8Y7;X2J3V4;W8YM39;W8Y4A2;W8SKC0;W8NKY4;W8NKP1;W8E988;W6SQV0;W6SNC0;W6SIV4;W6CJ57;						
0;F8RHE9;F8RHE8;F8RHE2;F8RHE1;F8RHE0;F8RHD9;F8RHD8;F8RHD7;F8RHD6;F8RHD5;F8RHD4;F8RHD3;F8RHD2;F8RHD1;F						
J4;X5EM81;X5CHJ2;X4YT82;X2J8Y7;X2J3V4;W8YM39;W8Y4A2;W8SKC0;W8NKY4;W8NKP1;W8E988;W6SQV0;W6SNC0;W6S						
0;F8RHE9;F8RHE8;F8RHE2;F8RHE1;F8RHE0;F8RHD9;F8RHD8;F8RHD7;F8RHD6;F8RHD5;F8RHD4;F8RHD3;F8RHD2;F8RHD1;F						
	10555	2	2	2	2	2
	By MS/MS	By matching	By MS/MS	By matching	18.4	18.4
	>sp|Q9Y314|NOSIP_HUM	9	7	7	7	5
	>tr|A0A075B6Q3|A0A075	4	14	13	2	14
	>tr|A0A075B6Q5|A0A075	1	1	1	1	1
	>tr|A0A075B6T1|A0A075	5	2	2	2	1
	>tr|H0YN73|H0YN73_HU	4	1	1	1	1
	>tr|A0A075B730|A0A075	3	4	1	1	4
	>tr|B4E312|B4E312_HUM	4	2	2	2	2
	>tr|K7ERP4|K7ERP4_HUM	12	4	4	4	3
	>tr|A0A087WT19|A0A08	7	1	1	1	0
	>tr|A0A087WTU8|A0A08	4	1	1	1	1
	>sp|Q96T88|UHRF1_HUM	5	20	20	19	15
	>tr|A0A087WTY9|A0A08	3	1	1	1	1
	>tr|E9PL01|E9PL01_HUM	6	2	2	2	2
	>tr|Q14BN2|Q14BN2_HU	14	2	2	2	2
	>tr|D9ZGF5|D9ZGF5_HU	3	4	4	4	4
	>tr|A0A087WZG9|A0A08	6	1	1	1	1
	>tr|A0A087WUL5|A0A08	2	4	4	4	4
	>tr|E7ETK0|E7ETK0_HUM	3	2	2	2	2
	>tr|A0A087WV02|A0A08	13	1	1	1	1
	>tr|A0A087WV66|A0A08	5	162	162	147	145
	>sp|P46087|NOP2_HUM	4	10	10	10	2
	>tr|A0A087WYH1|A0A08	3	1	1	1	1
	>tr|B4DED6|B4DED6_HU	7	8	6	6	6
	>tr|B4DI61|B4DI61_HUM	6	4	4	4	4
	>tr|B2RD24|B2RD24_HU	7	3	3	3	3
	>sp|Q14517|FAT1_HUM	2	1	1	1	1
	>tr|B3KUD6|B3KUD6_HU	13	2	2	2	2
	>tr|A0A087WYS3|A0A08	5	1	1	1	1
	>tr|A0A087WVZ9|A0A08	4	1	1	1	1
	>tr|Q86W17|Q86W17_H	22	1	1	1	1
	>sp|P24928|RPB1_HUM	4	6	6	6	6
	>tr|D3DR86|D3DR86_HU	6	9	9	9	0
	>tr|A0A087WWP4|A0A0	3	3	3	3	1
	>tr|A0A0C4DH79|A0A0C	4	1	1	1	1
	>tr|Q68E00|Q68E00_HU	3	1	1	1	1
	>tr|A0A087WX60|A0A08	3	3	2	2	3
	>tr|F8WBF5|F8WBF5_HU	7	1	1	1	1
	>tr|A0A087WXF7|A0A08	3	1	1	1	1

>tr|A0A087WXU5|A0A08	8	3	3	3	3
>sp|Q9H4H8|FA83D_HUI	2	3	3	3	1
>tr|E9PM37|E9PM37_HL	8	1	1	1	1
>tr|E9KY40|E9KY40_HUN	6	1	1	1	0
>tr|A0A087WXZ3|A0A08	7	6	6	4	6
>tr|A0A087WXZ8|A0A08	2	1	1	1	1
>tr|A0A087WY89|A0A08	3	1	1	1	0
>tr|A0A087WYZ0|A0A08	20	3	1	0	3
>tr|K7EKR9|K7EKR9_HUN	4	1	1	1	1
>tr|M0R210|M0R210_HL	8	4	4	4	4
>tr|D6R9P3|D6R9P3_HUI	7	4	4	4	4
>tr|A0A087WZZ5|A0A08	9	16	16	16	13
>tr|A0A087X1K6|A0A087	3	1	1	1	0
>tr|B2R5U7|B2R5U7_HU	4	6	6	6	3
>tr|B2RDK5|B2RDK5_HUI	5	2	2	2	2
>tr|H7C2D3|H7C2D3_HU	4	1	1	1	1
>tr|Q6PKC6|Q6PKC6_HU	11	1	1	1	1
>tr|B4DP70|B4DP70_HUI	5	1	1	1	1
>tr|D6RIC3|D6RIC3_HUN	8	4	4	4	3
>sp|Q14525|KT33B_HUN	4	16	4	3	1
>tr|S4S3R8|S4S3R8_HUN	11	4	4	4	4
>sp|Q8TA86|RP9_HUMA	5	7	7	7	7
>tr|F5H0Q6|F5H0Q6_HU	4	1	1	1	1
>tr|C9JP00|C9JP00_HUM	13	3	3	3	2
>sp|P98175|RBM10_HUN	4	6	6	6	5
>tr|K7ENH8|K7ENH8_HU	10	2	2	2	2
>tr|F8WDT3|F8WDT3_HL	7	1	1	1	1
>sp|Q14978|NOLC1_HUN	7	15	15	13	12
>tr|A0A0A0MRP0|A0A0A	2	4	4	4	3
>tr|Q86UA8|Q86UA8_HL	5	14	3	3	12
>tr|Q5TAL4|Q5TAL4_HUI	4	1	1	1	1
>tr|B6VEX5|B6VEX5_HUN	6	1	1	1	1
>sp|Q9P2E9|RRBP1_HUN	12	4	4	4	4
>tr|Q5VW28|Q5VW28_H	10	5	3	3	3
>tr|Q6PJR5|Q6PJR5_HUN	13	2	2	2	1
>tr|Q9BV39|Q9BV39_HU	6	1	1	1	1
>tr|Q6I9R8|Q6I9R8_HUN	12	9	9	9	9
>tr|B4DG61|B4DG61_HU	6	33	33	30	31
>tr|A7E2S2|A7E2S2_HUN	8	1	1	1	0
>tr|A0A0A0MTB8|A0A0A	2	5	5	5	2
>tr|V9HWF0|V9HWF0_H	4	5	5	5	4
>tr|Q2M1V9|Q2M1V9_H	3	1	1	1	1
>tr|Q5JRY4|Q5JRY4_HUN	25	1	1	1	1
>tr|B4E2I1|B4E2I1_HUM	5	2	2	2	2
>tr|Q86VU2|Q86VU2_HL	6	1	1	1	1

>tr|A0A0A6YYG7|A0A0A(	4	4	4	4	4
>sp|Q9NQ55|SSF1_HUM/	6	4	4	4	1
>sp|Q9H6W3|NO66_HUI	2	2	2	2	2
>tr|A0A0B4J1Z1|A0A0B4.	6	8	7	7	8
>tr|E9PLD3|E9PLD3_HUI	4	1	1	1	1
>tr|F8VV32|F8VV32_HUI	4	1	1	1	0
>tr|E7EUL6|E7EUL6_HUI	11	3	3	3	3
>tr|B7Z7W2|B7Z7W2_HL	7	1	1	1	1
>tr|A0A0C4DFX7|A0A0C4	3	9	9	9	9
>tr|A0PJ71|A0PJ71_HUN	5	1	1	1	1
>tr|F5GYV5|F5GYV5_HUI	10	7	7	7	5
>tr|A8K6X3|A8K6X3_HUI	6	20	20	20	12
>tr|Q59GQ7|Q59GQ7_HL	8	11	11	11	4
>tr|B3KY63|B3KY63_HUI	15	26	26	20	24
>tr|A0A0C4DGL3|A0A0C4	8	3	3	3	3
>tr|A0A0C4DGP2|A0A0C4	4	4	4	4	4
>sp|O95218|ZRAB2_HUN	2	5	5	5	4
>tr|B4DKV5|B4DKV5_HU	6	1	1	1	1
>tr|A0A0D9SFB3|A0A0D9	23	32	31	31	28
>tr|A0A0D9SF63|A0A0D9	25	7	7	7	7
>sp|Q8IWS0|PHF6_HUM/	4	9	9	9	7
>tr|A0A0F6QNT9|A0A0F6	2	2	2	2	1
>tr|E9PFF5|E9PFF5_HUN	13	3	3	3	3
>sp|Q8N8Y5|ZFP41_HUN	3	4	4	4	4
>sp|P0DMV9|HS71B_HUI	15	25	21	1	25
>tr|H0Y6G3|H0Y6G3_HU	8	1	1	1	1
>sp|Q9BYR2|KRA45_HUN	22	3	3	3	0
>sp|Q14160|SCRIB_HUM	6	12	12	11	11
>tr|A0A0G2JRI7|A0A0G2.	4	1	1	1	1
>tr|B0QYV1|B0QYV1_HU	31	9	9	9	9
>tr|A0A0G2JRY5|A0A0G2	9	2	2	2	2
>tr|Q86WI7|Q86WI7_HU	6	1	1	1	1
>tr|A0A0J9YYL3|A0A0J9Y	13	11	11	11	6
>tr|J3QT77|J3QT77_HUN	6	1	1	1	1
>tr|A0A0J9YYJ0|A0A0J9Y	2	2	2	2	2
>tr|A7E2X7|A7E2X7_HUI	3	3	3	3	3
>sp|Q99728|BARD1_HUI	6	3	3	3	3
>tr|A0JLS5|A0JLS5_HUM/	4	4	4	4	0
>tr|A0JLU2|A0JLU2_HUN	4	4	4	4	3
>sp|Q2TAY7|SMU1_HUN	2	2	2	2	1
>sp|Q9BQ67|GRWD1_HL	7	8	8	8	7
>sp|Q96DI7|SNR40_HUN	4	4	4	4	4
>tr|A0PJ52|A0PJ52_HUN	3	2	2	2	2
>tr|J3QSV6|J3QSV6_HUN	9	14	14	14	11
>tr|E7EPB3|E7EPB3_HUN	6	4	4	1	4

>tr|A0PJ70|A0PJ70_HUM	3	12	12	12	12
>sp|Q2TAL8|QRIC1_HUM	4	5	5	5	5
>tr|Q96DV6|Q96DV6_HU	6	6	6	6	6
>tr|B4DHJ4|B4DHJ4_HUM	4	1	1	1	0
>tr|B3KTM6|B3KTM6_HU	6	11	11	11	11
>tr|A2VCR1|A2VCR1_HU	5	1	1	1	1
>tr|Q6NUN2|Q6NUN2_H	12	12	12	12	8
>tr|Q4VB24|Q4VB24_HU	9	10	2	2	9
>tr|A4D110|A4D110_HU	1	1	1	1	1
>sp|Q13185|CBX3_HUM	10	6	6	5	6
>sp|Q8N201|INT1_HUM	4	4	4	4	4
>tr|A4D2A4|A4D2A4_HU	5	2	1	0	2
>sp|O94901|SUN1_HUM	11	3	3	3	3
>tr|Q1XBU8|Q1XBU8_HU	10	2	2	2	2
>tr|H0YMJ0|H0YMJ0_HU	10	3	3	3	3
>tr|A5D8Z7|A5D8Z7_HU	2	1	1	1	1
>tr|A7YDK7|A7YDK7_HU	15	1	1	1	0
>tr|B4DXG8|B4DXG8_HU	4	1	1	1	1
>tr|A6H8X9|A6H8X9_HU	13	3	3	3	3
>sp|Q9NTG1|PKDRE_HU	2	2	2	2	0
>sp|A6NCN2|KR87P_HUM	1	13	2	2	0
>sp|P51610|HCFC1_HUM	4	24	24	24	22
>tr|A6NEM5|A6NEM5_H	3	2	2	2	2
>sp|Q14202|ZMYM3_HU	5	17	17	17	15
>sp|A6NHR9|SMHD1_HU	2	8	8	8	4
>tr|B7Z3E7|B7Z3E7_HUM	7	8	8	7	2
>tr|Q9NSJ7|Q9NSJ7_HUM	3	1	1	1	1
>tr|Q9H8A8|Q9H8A8_HU	8	2	2	2	0
>sp|P51532|SMCA4_HUM	15	22	22	22	20
>tr|B2RNX8|B2RNX8_HU	3	1	1	1	1
>tr|A7MD96|A7MD96_H	3	1	1	1	0
>tr|Q05DQ6|Q05DQ6_HU	4	4	4	4	4
>tr|G3XAN4|G3XAN4_HU	5	1	1	1	1
>sp|Q68CQ4|DIEXF_HUM	2	3	3	3	0
>tr|A8K194|A8K194_HUM	4	6	6	6	6
>tr|Q5U0N1|Q5U0N1_HU	4	3	3	3	3
>sp|Q9H081|MIS12_HUM	2	2	2	2	2
>tr|H7C128|H7C128_HU	14	3	3	3	3
>tr|J3QQW9|J3QQW9_H	3	4	4	4	4
>tr|A8K201|A8K201_HUM	3	5	5	5	5
>sp|O75600|KBL_HUMAI	2	1	1	1	1
>tr|B2RCW4|B2RCW4_HU	4	2	2	2	2
>tr|B4DRR0|B4DRR0_HU	6	36	3	2	27
>tr|B7Z4C6|B7Z4C6_HUM	7	5	5	5	5
>sp|Q6MZZ7|CAN13_HU	2	1	1	1	1

>tr|A8K2R3|A8K2R3_HUI	4	9	9	9	9
>tr|B7Z1C7|B7Z1C7_HUN	9	10	3	3	6
>tr|A8K330|A8K330_HUI	3	3	3	3	3
>tr|A8K394|A8K394_HUI	1	49	1	1	47
>tr|B7Z2F4|B7Z2F4_HUN	5	3	3	3	2
>sp|O95232|LC7L3_HUM	13	10	10	10	10
>sp|Q96NJ6|ZFP3_HUMA	2	2	1	1	2
>tr|B4DQY1|B4DQY1_HU	16	3	3	3	3
>sp|Q13242|SRSF9_HUN	5	7	6	6	3
>tr|A8K3Q9|A8K3Q9_HU	1	4	1	1	4
>tr|Q6DKJ9|Q6DKJ9_HUI	6	1	1	1	1
>sp|P40937|RFC5_HUMA	10	5	5	5	5
>sp|Q99661|KIF2C_HUM	10	26	26	25	23
>tr|Q4G1A1|Q4G1A1_HU	6	3	3	3	3
>sp|Q9NX58|LYAR_HUM.	3	3	3	3	3
>tr|C9JW96|C9JW96_HU	5	1	1	1	0
>tr|V9HWF5|V9HWF5_HI	16	9	9	9	7
>tr|Q6AHX7|Q6AHX7_HU	3	1	1	1	1
>sp|Q9BXS6|NUSAP_HUI	2	3	3	3	3
>tr|Q6NZ55|Q6NZ55_HU	9	9	9	9	8
>tr|Q59H57|Q59H57_HU	9	5	5	5	3
>sp|Q96G23|CERS2_HUN	9	3	3	3	3
>sp|Q14146|URB2_HUM.	3	6	6	6	4
>tr|B4DSJ1|B4DSJ1_HUN	8	3	3	3	3
>tr|F5H0J3|F5H0J3_HUM	4	1	1	1	1
>tr|Q6NXR8|Q6NXR8_HU	15	9	9	9	8
>tr|H3BPC4|H3BPC4_HUI	6	2	2	2	0
>sp|P62266|RS23_HUMA	6	4	4	4	4
>tr|B1AHB1|B1AHB1_HU	11	15	15	15	10
>sp|Q15233|NONO_HUN	5	11	11	11	4
>sp|Q8IZL8|PELP1_HUM.	11	18	18	18	16
>tr|B4DW90|B4DW90_H	6	2	2	2	2
>tr|Q59GY3|Q59GY3_HU	10	7	7	4	7
>sp|Q9P0V3|SH3B4_HUN	2	1	1	1	1
>sp|Q6IQ21|ZN770_HUN	2	2	2	2	2
>tr|Q59EF5|Q59EF5_HUI	3	4	1	1	4
>sp|Q07021|C1QBP_HUI	2	1	1	1	1
>sp|Q9UBL3|ASH2L_HUN	8	8	8	8	7
>sp|Q15393|SF3B3_HUN	5	10	10	10	10
>tr|B4DMI9|B4DMI9_HU	3	6	6	6	6
>tr|Q5STV6|Q5STV6_HUI	5	3	3	3	3
>tr|Q7Z726|Q7Z726_HUI	6	5	5	5	5
>sp|P60842|IF4A1_HUM.	19	3	2	2	3
>tr|B3KUU7|B3KUU7_HU	15	10	10	10	5
>tr|H3BVC7|H3BVC7_HU	5	1	1	1	1

<table>
<tr><td>>sp|O43929|ORC4_HUM</td><td>9</td><td>2</td><td>2</td><td>2</td><td>2</td></tr>
<tr><td>>tr|B7Z8N6|B7Z8N6_HU</td><td>5</td><td>1</td><td>1</td><td>1</td><td>1</td></tr>
<tr><td>>sp|Q14592|ZN460_HUN</td><td>2</td><td>3</td><td>3</td><td>2</td><td>3</td></tr>
<tr><td>>sp|Q9H7B2|RPF2_HUM</td><td>4</td><td>3</td><td>3</td><td>3</td><td>3</td></tr>
<tr><td>>tr|A8K818|A8K818_HUN</td><td>4</td><td>4</td><td>4</td><td>4</td><td>3</td></tr>
<tr><td>>sp|O43790|KRT86_HUN</td><td>9</td><td>22</td><td>2</td><td>0</td><td>2</td></tr>
<tr><td>>sp|Q9BTT6|LRRC1_HUN</td><td>3</td><td>4</td><td>3</td><td>3</td><td>4</td></tr>
<tr><td>>tr|C9IYN3|C9IYN3_HUM</td><td>8</td><td>13</td><td>12</td><td>12</td><td>8</td></tr>
<tr><td>>tr|C9JL92|C9JL92_HUM</td><td>5</td><td>1</td><td>1</td><td>1</td><td>0</td></tr>
<tr><td>>sp|Q8WTT2|NOC3L_HU</td><td>3</td><td>3</td><td>3</td><td>3</td><td>3</td></tr>
<tr><td>>tr|K7EP06|K7EP06_HUN</td><td>7</td><td>3</td><td>3</td><td>3</td><td>1</td></tr>
<tr><td>>tr|E9PKP7|E9PKP7_HUN</td><td>10</td><td>14</td><td>14</td><td>14</td><td>8</td></tr>
<tr><td>>sp|Q9H307|PININ_HUN</td><td>6</td><td>12</td><td>12</td><td>12</td><td>8</td></tr>
<tr><td>>sp|O00567|NOP56_HUN</td><td>7</td><td>24</td><td>24</td><td>24</td><td>23</td></tr>
<tr><td>>sp|Q7Z2W4|ZCCHV_HU</td><td>4</td><td>2</td><td>2</td><td>2</td><td>1</td></tr>
<tr><td>>tr|X5D2J9|X5D2J9_HUN</td><td>17</td><td>10</td><td>10</td><td>10</td><td>10</td></tr>
<tr><td>>tr|E9PFK5|E9PFK5_HUN</td><td>4</td><td>2</td><td>2</td><td>2</td><td>2</td></tr>
<tr><td>>tr|B4DYU4|B4DYU4_HU</td><td>4</td><td>1</td><td>1</td><td>1</td><td>1</td></tr>
<tr><td>>tr|J3KNR6|J3KNR6_HUN</td><td>3</td><td>1</td><td>1</td><td>1</td><td>1</td></tr>
<tr><td>>tr|Q70T21|Q70T21_HU</td><td>4</td><td>2</td><td>2</td><td>2</td><td>2</td></tr>
<tr><td>>sp|P55199|ELL_HUMAN</td><td>7</td><td>5</td><td>5</td><td>5</td><td>5</td></tr>
<tr><td>>tr|Q6IBM8|Q6IBM8_HU</td><td>11</td><td>11</td><td>11</td><td>10</td><td>9</td></tr>
<tr><td>>tr|Q68CX8|Q68CX8_HU</td><td>4</td><td>2</td><td>2</td><td>2</td><td>2</td></tr>
<tr><td>>tr|J3QLE5|J3QLE5_HUM</td><td>14</td><td>3</td><td>3</td><td>3</td><td>2</td></tr>
<tr><td>>tr|Q49AN9|Q49AN9_HL</td><td>4</td><td>2</td><td>2</td><td>2</td><td>2</td></tr>
<tr><td>>sp|P43243|MATR3_HUN</td><td>25</td><td>43</td><td>43</td><td>43</td><td>42</td></tr>
<tr><td>>tr|F2Z2C0|F2Z2C0_HUN</td><td>3</td><td>1</td><td>1</td><td>1</td><td>1</td></tr>
<tr><td>>tr|B0AZM1|B0AZM1_HL</td><td>9</td><td>6</td><td>6</td><td>6</td><td>6</td></tr>
<tr><td>>sp|Q9UQE7|SMC3_HUN</td><td>3</td><td>21</td><td>21</td><td>21</td><td>19</td></tr>
<tr><td>>tr|B0AZS5|B0AZS5_HUN</td><td>3</td><td>2</td><td>1</td><td>1</td><td>2</td></tr>
<tr><td>>tr|B0QXZ6|B0QXZ6_HU</td><td>2</td><td>4</td><td>3</td><td>3</td><td>4</td></tr>
<tr><td>>tr|B0QYK0|B0QYK0_HU</td><td>10</td><td>5</td><td>5</td><td>4</td><td>5</td></tr>
<tr><td>>tr|E9PAP1|E9PAP1_HUN</td><td>8</td><td>1</td><td>1</td><td>1</td><td>1</td></tr>
<tr><td>>sp|P39019|RS19_HUMA</td><td>8</td><td>4</td><td>4</td><td>4</td><td>3</td></tr>
<tr><td>>tr|Q6FHM6|Q6FHM6_H</td><td>3</td><td>3</td><td>3</td><td>3</td><td>3</td></tr>
<tr><td>>tr|B1AK63|B1AK63_HU</td><td>2</td><td>1</td><td>1</td><td>1</td><td>1</td></tr>
<tr><td>>tr|B1AKC8|B1AKC8_HU</td><td>3</td><td>2</td><td>2</td><td>2</td><td>2</td></tr>
<tr><td>>tr|B1AKN7|B1AKN7_HU</td><td>10</td><td>9</td><td>9</td><td>6</td><td>3</td></tr>
<tr><td>>tr|B1ALC2|B1ALC2_HUN</td><td>3</td><td>2</td><td>2</td><td>2</td><td>2</td></tr>
<tr><td>>tr|B1AMU7|B1AMU7_H</td><td>6</td><td>2</td><td>2</td><td>2</td><td>1</td></tr>
<tr><td>>tr|H0Y757|H0Y757_HUN</td><td>6</td><td>1</td><td>1</td><td>1</td><td>0</td></tr>
<tr><td>>tr|Q96IR1|Q96IR1_HUN</td><td>12</td><td>13</td><td>13</td><td>13</td><td>12</td></tr>
<tr><td>>sp|P62899|RL31_HUMA</td><td>8</td><td>7</td><td>7</td><td>7</td><td>4</td></tr>
<tr><td>>sp|P06702|S10A9_HUM</td><td>2</td><td>1</td><td>1</td><td>1</td><td>0</td></tr>
<tr><td>>tr|Q0VAS5|Q0VAS5_HU</td><td>4</td><td>6</td><td>6</td><td>6</td><td>6</td></tr>
</table>

>sp|P62857|RS28_HUMA	2	3	3	3	3
>sp|Q969Q0|RL36L_HUM	7	2	2	2	2
>tr|G3V4W0|G3V4W0_H	31	11	11	11	11
>tr|Q9UK43|Q9UK43_HU	9	4	4	4	3
>sp|P84103|SRSF3_HUM	3	10	10	9	10
>tr|Q59EG3|Q59EG3_HU	6	1	1	1	1
>tr|Q53FC7|Q53FC7_HU	6	9	1	1	9
>sp|Q14244|MAP7_HUM	6	8	8	8	8
>tr|B4DXX1|B4DXX1_HU	3	12	12	12	2
>tr|E5RIZ4|E5RIZ4_HUM	3	1	1	1	1
>tr|B4DDX2|B4DDX2_HU	4	6	6	6	6
>tr|Q53HJ4|Q53HJ4_HU	8	24	24	24	14
>tr|Q6FGU7|Q6FGU7_HU	10	2	2	2	2
>tr|Q6IAP9|Q6IAP9_HUM	5	4	4	4	3
>sp|O75934|SPF27_HUM	3	2	2	2	2
>sp|Q13112|CAF1B_HUM	2	3	3	3	1
>tr|F8WBJ6|F8WBJ6_HU	3	1	1	1	1
>sp|P53350|PLK1_HUMA	8	12	12	12	12
>tr|B2R853|B2R853_HU	2	34	2	0	24
>tr|B4DQ46|B4DQ46_HU	6	1	1	1	1
>sp|Q96C00|ZBTB9_HUM	4	9	9	9	9
>tr|E9PPX2|E9PPX2_HUM	4	1	1	1	1
>tr|B7Z7P4|B7Z7P4_HUM	6	1	1	1	0
>sp|Q96HI0|SENP5_HUM	2	2	2	2	2
>sp|Q9UQR1|ZN148_HU	11	8	8	8	8
>tr|Q5TI72|Q5TI72_HUM	6	1	1	1	1
>tr|H0YNU5|H0YNU5_HU	8	7	7	7	5
>tr|B2RAX6|B2RAX6_HU	3	2	2	2	2
>tr|B2RB99|B2RB99_HU	4	7	7	1	6
>sp|O95391|SLU7_HUMA	5	7	7	7	7
>sp|P33993|MCM7_HUM	7	5	5	5	5
>tr|B3KMF2|B3KMF2_HU	3	2	2	2	1
>sp|Q8WY64|MYLIP_HU	2	1	1	1	0
>sp|Q9H2P0|ADNP_HUM	4	7	7	7	7
>sp|Q14974|IMB1_HUMA	7	6	6	6	6
>sp|B2RBV5|MRUPP_HU	1	1	1	1	1
>tr|B2RD09|B2RD09_HU	5	20	20	1	16
>tr|I3L2G3|I3L2G3_HUM	7	3	3	3	1
>tr|B3KXD6|B3KXD6_HU	7	4	4	4	2
>tr|E9PNW8|E9PNW8_H	4	2	2	2	2
>tr|Q8N1H4|Q8N1H4_HU	7	9	9	7	9
>tr|Q9BRL5|Q9BRL5_HU	13	2	2	2	2
>tr|Q5RKT7|Q5RKT7_HU	4	8	8	2	8
>sp|P04844|RPN2_HUMA	5	2	2	2	2
>tr|B2RNR6|B2RNR6_HU	7	25	25	25	19

>tr|B2RTX8|B2RTX8_HUI	4	7	7	7	6
>tr|B2RWN5|B2RWN5_H	11	17	17	17	16
>tr|B2RWP5|B2RWP5_HI	8	16	16	15	16
>sp|P78364|PHC1_HUMA	15	21	21	21	21
>tr|B7Z6D5|B7Z6D5_HUI	7	4	4	4	4
>sp|Q5RI15|COX20_HUM	2	1	1	1	1
>sp|O95816|BAG2_HUM	3	8	8	8	8
>tr|B3KM81|B3KM81_HU	5	5	5	5	5
>tr|M0R3C3|M0R3C3_HU	5	6	6	6	6
>tr|B4DZC3|B4DZC3_HUI	4	10	10	10	8
>sp|Q8N2W9|PIAS4_HUI	4	6	6	6	2
>sp|Q5JTH9|RRP12_HUM	8	20	20	20	19
>tr|B3KMZ7|B3KMZ7_HU	4	15	15	15	15
>tr|B4DE78|B4DE78_HUI	4	3	1	1	2
>sp|Q9Y3T9|NOC2L_HUI	5	6	6	6	5
>tr|B3KNE7|B3KNE7_HUI	4	2	2	2	1
>sp|P61619|S61A1_HUM	17	4	4	4	4
>tr|B3KNK5|B3KNK5_HU	4	22	1	1	20
>tr|B3KNN7|B3KNN7_HU	8	2	2	2	2
>tr|E9PC66|E9PC66_HUI	9	8	8	7	8
>sp|Q969R5|LMBL2_HUI	2	1	1	1	1
>tr|B3KSR1|B3KSR1_HUI	4	1	1	1	0
>sp|Q8N5C6|SRBD1_HUI	4	14	14	14	10
>tr|B3KT06|B3KT06_HUI	22	20	3	0	20
>tr|B3KQ51|B3KQ51_HU	10	3	3	3	3
>tr|D1KF47|D1KF47_HUI	15	6	6	6	4
>tr|B3KQG6|B3KQG6_HU	5	3	3	3	3
>tr|B4DWS6|B4DWS6_HI	4	1	1	1	1
>tr|B3KQX0|B3KQX0_HU	3	1	1	1	1
>tr|B3KRJ9|B3KRJ9_HUM	3	3	3	3	1
>tr|B3KRK2|B3KRK2_HUI	5	9	9	9	9
>sp|Q86XI2|CNDG2_HUI	7	5	5	5	5
>tr|F2Z2B9|F2Z2B9_HUI	4	1	1	1	1
>tr|B4DSF4|B4DSF4_HUI	3	3	3	3	3
>tr|B4E0N9|B4E0N9_HUI	11	3	3	3	3
>sp|Q8N163|CCAR2_HUI	10	8	8	8	8
>tr|B4DWZ7|B4DWZ7_HI	3	2	2	2	2
>tr|H0YHD0|H0YHD0_HU	4	1	1	1	1
>tr|Q53FG6|Q53FG6_HU	3	1	1	1	1
>tr|B3KVL5|B3KVL5_HUI	2	1	1	1	1
>sp|Q8TF66|LRC15_HUM	2	2	2	2	0
>tr|B3KWT5|B3KWT5_HU	14	3	3	3	3
>tr|F8W6D9|F8W6D9_HU	7	3	3	3	2
>tr|E9PD53|E9PD53_HUI	11	19	19	19	19
>tr|B4DSV5|B4DSV5_HUI	6	4	4	4	4

>tr|B4DSH1|B4DSH1_HU	3	17	17	17	15
>tr|B4DDB6|B4DDB6_HU	6	18	1	1	11
>tr|B4DDF4|B4DDF4_HU	14	10	10	10	9
>tr|B4DDG3|B4DDG3_HU	2	1	1	1	1
>tr|B7Z5U1|B7Z5U1_HUI	4	1	1	1	0
>tr|B4DE59|B4DE59_HUI	7	10	4	0	7
>tr|C9JRJ8|C9JRJ8_HUMA	7	2	2	2	2
>tr|Q658M0|Q658M0_HI	6	3	3	3	3
>tr|U3KQP3|U3KQP3_HU	6	2	2	2	2
>tr|B4DF70|B4DF70_HUI	4	3	2	2	3
>tr|B4DFE2|B4DFE2_HUI	11	13	13	13	12
>tr|F5H669|F5H669_HUI	15	2	2	2	0
>tr|B4DH46|B4DH46_HU	3	3	3	3	3
>tr|C9J0K6|C9J0K6_HUM	3	1	1	1	0
>tr|C9JWJ8|C9JWJ8_HUN	10	6	4	4	6
>sp|Q9H6R0|DHX33_HUI	5	10	10	10	5
>tr|B4DJ22|B4DJ22_HUN	4	1	1	1	1
>tr|K7EMR1|K7EMR1_HU	4	1	1	1	1
>tr|B4DJK4|B4DJK4_HUN	4	1	1	1	0
>tr|Q5QPE2|Q5QPE2_HU	8	2	2	1	1
>tr|B4DKT9|B4DKT9_HUI	6	2	2	1	2
>tr|B4DKY9|B4DKY9_HUI	5	2	2	2	1
>tr|B4DL02|B4DL02_HUI	9	3	3	3	2
>tr|I3L2Z8|I3L2Z8_HUMA	7	1	1	1	1
>tr|B4DLG2|B4DLG2_HUI	7	10	10	10	6
>tr|Q96ID3|Q96ID3_HUN	3	1	1	1	1
>tr|B4E1M5|B4E1M5_HU	8	4	4	4	3
>tr|B4DM62|B4DM62_HI	3	1	1	1	0
>tr|B4DM94|B4DM94_HI	12	7	7	7	7
>tr|B4DMG1|B4DMG1_H	4	2	2	2	2
>sp|Q96RU7|TRIB3_HUN	5	3	3	3	0
>tr|F8VQE1|F8VQE1_HUI	7	8	8	8	0
>tr|B4DVK1|B4DVK1_HU	13	3	3	3	3
>tr|C9J0J7|C9J0J7_HUMA	8	2	2	2	2
>tr|B4DNH5|B4DNH5_HU	31	7	7	5	7
>tr|B4E0I8|B4E0I8_HUM	10	12	12	12	4
>tr|B4DVN1|B4DVN1_HU	24	4	4	4	3
>tr|B4DPZ5|B4DPZ5_HUI	4	5	5	5	4
>tr|B4DP20|B4DP20_HUI	6	3	3	3	3
>tr|G3V4N7|G3V4N7_HU	5	1	1	1	1
>tr|B4DP61|B4DP61_HUI	3	3	3	3	3
>tr|B4DQE4|B4DQE4_HU	4	2	2	2	2
>tr|B4DQG8|B4DQG8_HU	10	8	8	8	8
>tr|F5H8L7|F5H8L7_HUN	5	1	1	1	1
>tr|B4DRF6|B4DRF6_HUI	3	1	1	1	1

>tr|E9PLN6|E9PLN6_HUI	7	2	2	2	2
>tr|B4DRS4|B4DRS4_HUI	2	2	2	2	0
>tr|B7ZB05|B7ZB05_HUI	6	9	9	9	8
>tr|Q2F838|Q2F838_HUI	5	1	1	1	0
>tr|B4DSR6|B4DSR6_HUI	2	1	1	1	1
>tr|B4DSU9|B4DSU9_HU	3	3	3	3	2
>tr|Q59FU3|Q59FU3_HU	7	9	4	4	6
>tr|J3KN87|J3KN87_HUI	5	3	3	3	2
>sp|Q86VM9|ZCH18_HU	10	18	18	18	17
>tr|B4DUB8|B4DUB8_HU	2	4	4	4	4
>tr|B4DUT7|B4DUT7_HU	3	7	7	7	3
>tr|B4DV47|B4DV47_HU	2	5	5	5	3
>tr|B4DV51|B4DV51_HU	5	3	3	3	2
>tr|B4DW11|B4DW11_H	3	1	1	1	1
>tr|Q8TAK2|Q8TAK2_HU	3	1	1	1	1
>tr|B4DWW8|B4DWW8_	7	3	3	3	2
>tr|K7ELP2|K7ELP2_HUI	4	1	1	1	1
>tr|B4DY09|B4DY09_HUI	7	8	8	8	7
>tr|Q9BTA4|Q9BTA4_HU	12	5	5	5	5
>tr|Q8IVW5|Q8IVW5_HU	7	1	1	1	1
>tr|B4DYP7|B4DYP7_HUI	5	4	4	4	4
>tr|B4DYP9|B4DYP9_HUI	3	1	1	1	1
>sp|Q8NAP3|ZBT38_HUI	8	2	2	2	1
>tr|B4DYY5|B4DYY5_HUI	11	15	15	15	15
>tr|B7ZAX9|B7ZAX9_HUI	5	23	23	12	20
>tr|B4DZE5|B4DZE5_HUI	5	6	6	6	6
>tr|B4DZM3|B4DZM3_HU	6	4	4	4	0
>tr|B4DZP5|B4DZP5_HUI	13	9	9	9	9
>tr|B4DZR9|B4DZR9_HUI	3	8	8	8	4
>sp|Q9BY77|PDIP3_HUI	11	11	11	11	5
>tr|B4E0S6|B4E0S6_HUI	2	18	18	18	16
>tr|B4E0T1|B4E0T1_HUI	2	2	2	2	0
>tr|B4E157|B4E157_HUI	3	1	1	1	1
>tr|B4E1Z2|B4E1Z2_HUI	4	13	1	1	13
>tr|B4E206|B4E206_HUI	4	3	3	3	3
>tr|B4E257|B4E257_HUI	4	2	2	2	0
>tr|B4E265|B4E265_HUI	2	2	2	2	1
>sp|P08579|RU2B_HUM	2	1	1	1	1
>tr|Q6IT96|Q6IT96_HUI	8	7	3	3	7
>sp|Q9Y265|RUVB1_HUI	7	17	17	17	16
>tr|Q6FI27|Q6FI27_HUM	3	2	2	2	2
>tr|F5ATB8|F5ATB8_HUI	12	2	2	2	2
>tr|H7C2Y2|H7C2Y2_HUI	6	1	1	1	1
>tr|E5RHY8|E5RHY8_HUI	5	1	1	1	0
>tr|Q9H6S8|Q9H6S8_HU	6	1	1	1	1

>tr|F5H6U2|F5H6U2_HU	5	2	2	2	2
>tr|B7Z2E2|B7Z2E2_HUM	6	3	3	3	3
>tr|C9JM75|C9JM75_HU	5	1	1	1	0
>tr|Q6FI30|Q6FI30_HUM	4	6	5	5	5
>tr|B7Z4V2|B7Z4V2_HUM	12	23	22	22	23
>tr|H7C4B7|H7C4B7_HU	7	1	1	1	1
>tr|C9J813|C9J813_HUM	10	4	4	4	3
>tr|B7Z6P1|B7Z6P1_HUM	52	12	1	1	12
>tr|F8W1R7|F8W1R7_HU	17	5	5	5	5
>tr|B7Z7S9|B7Z7S9_HUM	2	5	5	5	5
>tr|H3BND8|H3BND8_HU	9	6	6	6	6
>tr|E7EWK3|E7EWK3_HU	6	7	7	7	6
>tr|E9PMS6|E9PMS6_HU	14	8	8	8	8
>tr|B7Z8Z8|B7Z8Z8_HUM	5	4	4	4	4
>tr|Q05CP4|Q05CP4_HU	5	2	2	2	0
>tr|B7ZKR8|B7ZKR8_HUM	5	4	4	4	4
>tr|H0YLX2|H0YLX2_HUM	3	1	1	1	1
>tr|B7ZL72|B7ZL72_HUM	4	6	6	6	6
>sp|Q9UL59|ZN214_HUM	2	2	1	1	2
>sp|Q9NVI1|FANCI_HUM	9	9	9	9	9
>sp|Q3L8U1|CHD9_HUM	16	37	37	27	35
>tr|B8ZZU8|B8ZZU8_HUM	4	2	2	2	1
>sp|P11387|TOP1_HUMA	13	7	7	7	4
>tr|J3QWB6|J3QWB6_HU	4	1	1	1	1
>tr|B9EGR5|B9EGR5_HU	6	17	17	14	16
>tr|W6CJ52|W6CJ52_HU	4	4	4	3	2
>tr|C8CHS3|C8CHS3_HU	2	1	1	1	0
>tr|F8WAU5|F8WAU5_H	6	2	2	2	1
>tr|C9J352|C9J352_HUM	7	1	1	1	1
>tr|C9J384|C9J384_HUM	5	3	3	3	3
>tr|F8WCJ1|F8WCJ1_HU	8	1	1	1	0
>tr|C9J4Z3|C9J4Z3_HUM	6	2	2	2	2
>tr|C9J808|C9J808_HUM	4	2	2	2	2
>tr|C9J8B4|C9J8B4_HUM	2	1	1	1	1
>sp|Q14061|COX17_HUM	3	2	2	2	2
>tr|C9JA28|C9JA28_HUM	2	1	1	1	1
>tr|C9JA69|C9JA69_HUM	15	21	21	21	21
>tr|C9JCD9|C9JCD9_HUM	3	1	1	1	1
>tr|C9JFR7|C9JFR7_HUM	5	2	2	2	2
>tr|C9JL85|C9JL85_HUMA	3	1	1	1	1
>tr|C9JXB8|C9JXB8_HUM	4	5	5	5	5
>tr|C9JW69|C9JW69_HU	7	5	5	5	3
>tr|C9JYM0|C9JYM0_HU	2	1	1	1	0
>tr|C9JZI1|C9JZI1_HUMA	8	10	10	10	9
>tr|Q96FS1|Q96FS1_HUM	8	5	5	5	5

>A2A5Y0 TREMBL:A2A5Y(	1	15	5	1	1
>A2AB72 TREMBL:A2AB7Z	1	3	2	2	1
>ENSEMBL:ENSBTAP0000	8	3	3	3	3
>ENSEMBL:ENSBTAP0000	1	12	2	1	12
>sp|O76013|KRT36_HUM	2	11	3	3	1
>sp|O95678|K2C75_HUM	2	15	1	1	13
>P00761 SWISS-PROT:P0(	1	10	10	10	8
>sp|P02533|K1C14_HUM	14	31	27	12	23
>P02535-1 SWISS-PROT:P	1	10	1	1	9
>P02662 SWISS-PROT:P02	1	9	9	9	5
>P02663 SWISS-PROT:P02	1	6	6	6	1
>P02666 SWISS-PROT:P02	1	4	4	4	3
>P02668 SWISS-PROT:P02	1	6	6	6	2
>P02754 SWISS-PROT:P02	1	7	7	7	2
>P02769 SWISS-PROT:P02	16	83	83	83	74
>P02777 SWISS-PROT:P02	1	1	1	1	1
>P04259 SWISS-PROT:P04	2	36	25	0	25
>sp|P05787|K2C8_HUMA	13	20	11	11	18
>P06868 SWISS-PROT:P06	6	44	44	44	31
>sp|P08779|K1C16_HUM	8	30	17	17	14
>sp|P13645|K1C10_HUM	13	38	38	26	35
>sp|P13647|K2C5_HUMA	4	36	19	2	31
>P17690 SWISS-PROT:P17	1	1	1	1	0
>tr|O75370|O75370_HUI	7	1	1	1	1
>sp|P35527|K1C9_HUMA	3	32	32	32	31
>P35908 SWISS-PROT:P35	5	51	1	1	46
>sp|P78386|KRT85_HUM	4	22	5	3	2
>sp|Q04695|K1C17_HUM	12	22	10	7	17
>sp|Q15323|K1H1_HUMA	3	16	15	0	1
>Q1RMK2 TREMBL:Q1RM	1	6	6	6	5
>Q1RMN8 TREMBL:Q1RM	1	1	1	1	1
>Q2UVX4 SWISS-PROT:Q2	1	1	1	1	1
>sp|Q86Y46|K2C73_HUM	9	8	3	1	6
>sp|P08729|K2C7_HUMA	8	14	9	7	13
>Q3SX09 TREMBL:Q3SX09	1	1	1	1	1
>Q497I4 TREMBL:Q497I4	1	15	3	1	1
>sp|Q5D862|FILA2_HUM	2	6	6	6	4
>sp|Q5XKE5|K2C79_HUM	2	11	1	0	10
>Q5XQN5 SWISS-PROT:Q5	2	31	1	0	27
>Q61726 TREMBL:Q61726	1	20	4	3	2
>sp|Q6A162|K1C40_HUM	5	4	1	1	0
>sp|Q6KB66|K2C80_HUM	3	3	3	3	2
>sp|P78385|KRT83_HUM	4	24	23	1	2
>sp|Q8N1N4|K2C78_HUI	5	8	5	5	7
>sp|Q7Z3Z0|K1C25_HUM	4	3	1	1	2

>sp|Q7Z794|K2C1B_HUM	5	13	9	6	7
>sp|Q86YZ3|HORN_HUM	2	11	11	11	9
>sp|Q9BYT5|KRA22_HUM	7	3	3	3	0
>sp|Q9NSB4|KRT82_HUM	3	6	2	2	0
>Q9U6Y5 SWISS-PROT:Q9	1	8	8	8	8
>REFSEQ:XP_001474382 T	1	1	1	1	0
>REFSEQ:XP_986630 Tax_	1	15	2	2	1
>tr|Q8N4P8|Q8N4P8_HU	7	14	14	14	10
>tr|Q6NX58|Q6NX58_HU	7	9	9	9	9
>tr|D3DQF6|D3DQF6_HU	4	3	3	3	3
>sp|Q8N3X1|FNBP4_HUM	2	1	1	1	1
>tr|D3DS96|D3DS96_HU	7	5	5	5	4
>tr|D3DT44|D3DT44_HU	2	1	1	1	1
>tr|H3BV80|H3BV80_HU	8	3	3	3	2
>tr|K7EMM8|K7EMM8_H	6	5	5	5	4
>tr|D3DVB3|D3DVB3_HU	4	2	2	2	2
>tr|D3DWL9|D3DWL9_H	4	3	3	3	3
>tr|D6R9W4|D6R9W4_H	7	4	4	4	0
>tr|D6RFI4|D6RFI4_HUM	19	2	2	2	2
>sp|Q9UL40|ZN346_HUM	6	2	2	2	1
>sp|Q8N720|ZN655_HUM	5	5	5	5	4
>sp|Q2TAK8|MUM1_HUM	6	4	4	4	4
>sp|Q13111|CAF1A_HUM	4	4	4	4	3
>tr|E1NZA1|E1NZA1_HU	4	6	6	6	5
>tr|E5KLK2|E5KLK2_HUM	14	20	20	20	14
>sp|P78549|NTH_HUMA	6	7	7	7	3
>tr|E5RFZ8|E5RFZ8_HUM	6	1	1	1	1
>sp|P63208|SKP1_HUMA	8	5	5	5	5
>sp|P15090|FABP4_HUM	2	1	1	1	0
>sp|Q01469|FABP5_HUM	2	2	2	2	1
>tr|E7EN86|E7EN86_HUM	20	3	3	3	3
>tr|E7ESY4|E7ESY4_HUM	13	7	4	1	6
>tr|E7ET15|E7ET15_HUM	9	8	8	8	6
>tr|E7ETY2|E7ETY2_HUM	6	17	1	1	13
>tr|Q2I0Y7|Q2I0Y7_HUM	10	7	7	7	7
>sp|P27816|MAP4_HUM	15	3	3	3	3
>tr|E9PC90|E9PC90_HUM	4	2	2	2	1
>tr|Q75MX6|Q75MX6_H	5	6	6	6	5
>tr|E9PIE4|E9PIE4_HUMA	3	4	4	4	2
>tr|G3V1E0|G3V1E0_HU	4	1	1	1	1
>tr|E9PKG1|E9PKG1_HU	8	4	4	4	4
>tr|F8VSA6|F8VSA6_HUM	5	1	1	1	1
>tr|Q9BQQ5|Q9BQQ5_H	6	5	5	5	5
>tr|E9PSI3|E9PSI3_HUMA	4	2	2	2	2
>sp|P62861|RS30_HUMA	2	1	1	1	1

>sp|P24468|COT2_HUMA	2	7	7	3	7
>tr|F1DAL7|F1DAL7_HUN	5	5	1	1	5
>tr|F1JVV5|F1JVV5_HUM	11	2	1	1	1
>sp|Q8N8A6|DDX51_HUI	2	11	11	11	9
>tr|F2Z2U4|F2Z2U4_HUN	5	9	9	9	9
>tr|F2Z2W6|F2Z2W6_HU	4	1	1	1	0
>tr|I3L521|I3L521_HUMA	8	2	2	2	2
>tr|F5GZS6|F5GZS6_HUN	12	18	18	18	18
>sp|P29375|KDM5A_HUI	5	7	7	7	7
>tr|F5H3Y4|F5H3Y4_HUN	2	4	4	4	2
>tr|F8VRX9|F8VRX9_HUN	3	1	1	1	1
>sp|Q9BQE3|TBA1C_HUN	5	19	3	2	19
>tr|F6RFD5|F6RFD5_HUN	3	1	1	1	1
>tr|F8VPD4|F8VPD4_HUI	5	3	3	3	3
>tr|J3QK86|J3QK86_HUN	6	2	2	2	2
>tr|F8VW34|F8VW34_HU	7	1	1	1	0
>tr|F8VXG7|F8VXG7_HUI	9	30	30	30	27
>tr|H3BRU6|H3BRU6_HU	15	8	4	4	8
>tr|F8W038|F8W038_HU	2	1	1	1	1
>tr|F8W0Q9|F8W0Q9_HI	13	12	12	12	12
>tr|F8WAJ0|F8WAJ0_HU	3	9	9	9	6
>tr|F8WEI3|F8WEI3_HUN	5	1	1	1	1
>sp|Q29RF7|PDS5A_HUN	5	9	9	9	6
>tr|G3V256|G3V256_HUI	14	6	6	6	6
>tr|G3V2N2|G3V2N2_HU	2	1	1	1	1
>tr|G3V4E7|G3V4E7_HUI	5	1	1	1	1
>tr|G3V529|G3V529_HUI	12	22	22	22	19
>tr|G3V5L1|G3V5L1_HUN	11	4	4	4	4
>tr|G5E9G6|G5E9G6_HU	7	7	1	1	6
>tr|Q8NBL6|Q8NBL6_HU	9	3	3	3	3
>sp|P31943|HNRH1_HUN	26	20	20	13	19
>tr|V9HW98|V9HW98_H	6	3	1	1	1
>tr|V9GY48|V9GY48_HUI	4	3	3	3	2
>tr|H0Y2W2|H0Y2W2_HI	8	11	11	1	11
>tr|H0Y4R1|H0Y4R1_HUI	6	4	4	4	4
>sp|Q96EY4|TMA16_HUI	6	6	6	6	3
>tr|H0Y9Z8|H0Y9Z8_HUN	3	1	1	1	1
>sp|P28347|TEAD1_HUN	20	3	3	3	2
>tr|H0YEN5|H0YEN5_HU	17	12	12	12	10
>tr|H0YIN9|H0YIN9_HUN	1	10	1	1	8
>tr|H0YJS3|H0YJS3_HUM	2	1	1	1	1
>sp|P46779|RL28_HUMA	6	7	7	7	7
>sp|Q15004|PAF15_HUN	4	2	2	2	1
>tr|Q6JHV3|Q6JHV3_HUI	16	11	11	11	4
>sp|Q92841|DDX17_HUN	5	30	22	22	28

>sp|Q9UPN3|MACF1_HU	3	1	1	1	1
>tr|H3BPL5|H3BPL5_HUI	2	1	1	1	1
>tr|H3BR04|H3BR04_HUI	12	2	2	2	1
>tr|H3BU53|H3BU53_HU	2	4	1	1	3
>tr|H3BUJ7|H3BUJ7_HUI	7	6	6	6	6
>sp|P04264|K2C1_HUMA	7	47	44	0	44
>tr|H7BXI1|H7BXI1_HUM	3	2	2	2	2
>tr|H7BYN4|H7BYN4_HU	5	11	11	11	9
>sp|P48730|KC1D_HUMA	7	6	6	2	4
>tr|H7C0N4|H7C0N4_HU	2	6	2	2	6
>tr|H7C1M2|H7C1M2_HI	1	22	2	0	20
>sp|Q99848|EBP2_HUMA	5	10	10	10	9
>tr|Q7Z4Y3|Q7Z4Y3_HUI	3	1	1	1	0
>tr|H7C3P6|H7C3P6_HUI	3	4	4	4	4
>tr|H7C561|H7C561_HUI	1	6	3	0	6
>sp|Q6W2J9|BCOR_HUM	10	20	20	19	19
>sp|P10599|THIO_HUMA	2	5	5	5	5
>tr|I3L0U2|I3L0U2_HUM	3	1	1	1	1
>tr|I3L1P8|I3L1P8_HUMA	3	3	3	3	2
>sp|Q8WUZ0|BCL7C_HUI	2	1	1	1	1
>tr|I3L1Q5|I3L1Q5_HUM	2	1	1	1	1
>sp|Q96CP2|FWCH2_HU	3	4	4	3	0
>tr|I3L2A4|I3L2A4_HUMA	2	1	1	1	0
>tr|I3L2Z5|I3L2Z5_HUMA	13	9	8	8	8
>tr|I3VM54|I3VM54_HUI	4	11	11	11	11
>sp|P14174|MIF_HUMAN	2	1	1	1	0
>tr|I6ZVX6|I6ZVX6_HUM	4	2	1	1	2
>tr|J3KMX5|J3KMX5_HUI	3	7	7	7	7
>sp|O43248|HXC11_HUM	2	1	1	1	1
>sp|Q9UKL0|RCOR1_HUI	2	2	2	2	2
>tr|J3KRG2|J3KRG2_HUM	2	1	1	1	0
>tr|J3KSZ8|J3KSZ8_HUMA	7	9	9	9	9
>sp|P17844|DDX5_HUMA	15	37	37	29	32
>tr|Q8IWR8|Q8IWR8_HU	6	3	3	3	3
>sp|Q07955|SRSF1_HUM	8	13	13	12	12
>sp|Q8IWX8|CHERP_HUI	5	4	4	4	2
>tr|J3QL54|J3QL54_HUM	11	2	2	2	2
>sp|Q96MU7|YTDC1_HU	3	3	3	3	2
>sp|O43670|ZN207_HUM	12	9	9	9	7
>tr|J3QSH4|J3QSH4_HUI	3	11	11	10	11
>sp|Q03252|LMNB2_HUI	3	9	5	5	8
>tr|K7EID2|K7EID2_HUM	2	2	2	2	2
>tr|K7EJH0|K7EJH0_HUM	8	7	7	7	6
>tr|K7EQ55|K7EQ55_HUI	6	5	5	5	5
>tr|K7ELW0|K7ELW0_HU	4	4	4	4	4

>tr|K7EMZ9|K7EMZ9_HU	2	1	1	1	1
>tr|K7EQ03|K7EQ03_HUI	3	1	1	1	0
>tr|K7EPJ1|K7EPJ1_HUM	4	2	2	2	2
>tr|K7ERD7|K7ERD7_HUI	5	3	3	3	3
>tr|K7ESE6|K7ESE6_HUM	2	1	1	1	1
>tr|Q2VPJ6|Q2VPJ6_HUM	12	2	2	1	2
>tr|L7N2F3|L7N2F3_HUM	5	2	2	2	2
>tr|M0QXA7|M0QXA7_H	4	37	37	37	35
>tr|M0QXM4|M0QXM4_	5	2	2	2	2
>tr|M0R050|M0R050_HU	3	2	2	2	1
>tr|M0R301|M0R301_HU	1	1	1	1	1
>sp|Q9HCK1|ZDBF2_HUM	2	1	1	1	1
>sp|P67870|CSK2B_HUM	13	7	7	7	7
>sp|O00165|HAX1_HUM	3	5	5	5	5
>sp|O00257|CBX4_HUMA	3	25	25	25	24
>tr|Q6IB11|Q6IB11_HUM	2	1	1	1	1
>sp|O14646|CHD1_HUM	4	11	10	10	9
>sp|O14686|KMT2D_HUI	2	2	2	2	2
>sp|O14776|TCRG1_HUM	3	16	16	16	15
>sp|O14965|AURKA_HUI	9	16	16	16	15
>sp|O14980|XPO1_HUM	10	7	7	7	7
>tr|Q5T942|Q5T942_HUI	2	7	7	7	7
>tr|Q96HT3|Q96HT3_HU	6	3	3	3	2
>sp|O15294|OGT1_HUM	5	13	13	13	11
>sp|O15379|HDAC3_HUI	1	1	1	1	1
>sp|O43159|RRP8_HUMA	3	10	10	10	7
>sp|O43175|SERA_HUMA	4	2	2	2	2
>sp|O43290|SNUT1_HUM	5	9	9	9	8
>sp|O43390|HNRPR_HUI	8	19	19	15	15
>sp|O43474|KLF4_HUMA	4	2	2	2	0
>sp|O43660|PLRG1_HUM	3	4	4	4	2
>sp|O43684|BUB3_HUM	4	12	12	12	8
>sp|O43823|AKAP8_HUM	5	19	19	19	18
>sp|O60287|NPA1P_HUM	1	5	5	5	3
>sp|P23490|LORI_HUMA	3	1	1	1	1
>tr|Q05CK9|Q05CK9_HU	7	16	12	12	12
>sp|O60832|DKC1_HUMA	9	12	12	12	12
>sp|O75132|ZBED4_HUM	1	5	5	5	5
>sp|O75182|SIN3B_HUM	4	9	9	9	9
>sp|O75319|DUS11_HUM	1	4	4	4	2
>tr|Q53FC3|Q53FC3_HUI	5	3	3	3	2
>sp|O75367|H2AY_HUMA	4	4	4	4	4
>sp|O75400|PR40A_HUM	7	7	7	7	7
>sp|O75446|SAP30_HUM	1	3	3	3	3
>sp|O75475|PSIP1_HUM	3	9	9	8	5

>tr|Q5JRI1|Q5JRI1_HUM/	6	4	4	4	2
>sp|O75533|SF3B1_HUM	10	14	14	14	11
>sp|O75607|NPM3_HUM	1	3	3	3	3
>sp|O75643|U520_HUM/	9	13	13	13	12
>sp|O75691|UTP20_HUM	2	15	15	15	15
>sp|O75940|SPF30_HUM	1	6	6	6	4
>sp|O76076|WISP2_HUM	1	2	2	2	0
>sp|O94822|LTN1_HUM/	1	1	1	1	1
>sp|O94880|PHF14_HUM	2	2	2	2	1
>sp|O94906|PRP6_HUM/	1	13	13	13	12
>sp|O95071|UBR5_HUM.	5	14	14	14	14
>tr|Q6FI36|Q6FI36_HUM	2	5	5	5	0
>sp|O95235|KI20A_HUM	3	15	15	15	14
>sp|O95238|SPDEF_HUM	1	2	2	2	1
>sp|O95239|KIF4A_HUM	20	18	18	18	15
>sp|O95243|MBD4_HUM	2	11	11	11	7
>sp|O95259|KCNH1_HUI	1	1	1	1	0
>sp|O95365|ZBT7A_HUM	1	1	1	1	1
>sp|O95487|SC24B_HUM	1	1	1	1	0
>sp|O95639|CPSF4_HUM	6	3	3	3	3
>tr|Q53GY1|Q53GY1_HU	2	1	1	1	1
>sp|O95831|AIFM1_HUM	4	4	4	4	4
>tr|Q6FI97|Q6FI97_HUM	7	8	8	8	8
>sp|O96028|NSD2_HUM.	5	9	9	9	7
>tr|Q6LET3|Q6LET3_HUM	3	2	2	2	1
>sp|P02545|LMNA_HUM	10	48	48	46	47
>sp|P03973|SLPI_HUMAI	1	4	4	4	3
>sp|P04350|TBB4A_HUM	5	19	1	0	19
>tr|V9HVZ4|V9HVZ4_HU	11	8	8	8	6
>tr|V9HW43|V9HW43_H	5	6	6	6	6
>tr|Q6IBR0|Q6IBR0_HUM	8	12	12	12	12
>sp|P05023|AT1A1_HUM	32	9	9	9	8
>sp|P05109|S10A8_HUM	1	1	1	1	0
>sp|P05141|ADT2_HUM/	2	14	14	7	13
>tr|Q0QEN7|Q0QEN7_HI	6	6	6	6	6
>sp|P06748|NPM_HUMA	9	12	12	12	11
>tr|Q5JP53|Q5JP53_HUM	20	23	6	5	23
>sp|P07814|SYEP_HUMA	6	6	6	6	6
>tr|Q567R0|Q567R0_HU	2	1	1	1	1
>sp|P08047|SP1_HUMAN	6	6	6	6	5
>tr|V9HWE1|V9HWE1_H	20	26	25	23	24
>sp|P09382|LEG1_HUMA	3	7	7	7	6
>sp|P09661|RU2A_HUM/	9	19	19	19	7
>sp|Q71UI9|H2AV_HUM/	8	4	2	2	4
>sp|P0CW18|PRS56_HUI	2	2	2	2	0

>tr|Q1PSX1|Q1PSX1_HUI	4	1	1	1	1
>tr|V9HWB4|V9HWB4_H	3	40	40	38	40
>tr|V9HW22|V9HW22_H	29	37	35	29	37
>sp|P11388|TOP2A_HUM	2	41	41	31	41
>sp|P11441|UBL4A_HUM	2	2	2	2	2
>tr|Q6I9V5|Q6I9V5_HUM	9	11	4	4	11
>sp|P12273|PIP_HUMAN	1	2	2	2	1
>tr|Q53T09|Q53T09_HUI	2	5	5	5	5
>sp|P13639|EF2_HUMAN	7	10	9	9	10
>tr|Q6NTA2|Q6NTA2_HU	10	24	24	23	20
>sp|P15924|DESP_HUMA	7	66	66	66	45
>sp|P16401|H15_HUMAI	2	10	9	9	9
>sp|P16403|H12_HUMAI	1	11	11	3	9
>sp|P16615|AT2A2_HUM	10	6	6	6	6
>tr|Q5U079|Q5U079_HU	3	2	2	2	2
>sp|P17535|JUND_HUMA	2	3	3	3	2
>tr|Q99557|Q99557_HUI	3	1	1	1	1
>sp|P17812|PYRG1_HUM	6	12	12	12	12
>sp|P17987|TCPA_HUMA	10	9	9	9	9
>sp|P18077|RL35A_HUM	4	7	7	7	4
>sp|P18583|SON_HUMA	9	33	33	10	31
>sp|P19784|CSK22_HUM	4	3	3	3	3
>sp|P20700|LMNB1_HUM	6	33	31	29	29
>tr|Q5HY54|Q5HY54_HU	17	25	25	25	21
>sp|P21912|SDHB_HUMA	3	2	2	2	1
>tr|Q96BS4|Q96BS4_HUI	10	14	14	13	14
>sp|P22670|RFX1_HUMA	6	5	5	4	4
>tr|Q9BSV4|Q9BSV4_HU	5	7	7	7	4
>sp|P23258|TBG1_HUMA	4	4	4	4	4
>tr|V9HWC6|V9HWC6_H	2	4	4	4	0
>sp|P23396|RS3_HUMAN	17	11	11	11	9
>tr|V9HWI5|V9HWI5_HU	11	7	7	7	6
>sp|P25398|RS12_HUMA	1	5	5	5	5
>tr|V9HW26|V9HW26_H	9	8	8	8	8
>sp|P26368|U2AF2_HUM	3	9	9	9	9
>sp|P26447|S10A4_HUM	1	1	1	1	0
>sp|P26599|PTBP1_HUM	11	15	15	13	11
>sp|P27635|RL10_HUMA	13	12	12	12	11
>sp|P27824|CALX_HUMA	12	5	5	5	5
>tr|Q5STP9|Q5STP9_HUI	20	7	7	7	6
>tr|R4GN49|R4GN49_HU	2	1	1	1	0
>tr|Q1W6H1|Q1W6H1_H	3	13	13	13	9
>sp|P30050|RL12_HUMA	4	6	6	6	6
>sp|P31151|S10A7_HUM	1	2	2	2	0
>sp|P31276|HXC13_HUM	1	3	3	3	3

<table>
<tr><td>>sp|P31689|DNJA1_HUM</td><td>3</td><td>19</td><td>19</td><td>19</td><td>19</td></tr>
<tr><td>>sp|P31947|1433S_HUM</td><td>1</td><td>4</td><td>4</td><td>2</td><td>1</td></tr>
<tr><td>>sp|P32320|CDD_HUMA</td><td>2</td><td>4</td><td>4</td><td>3</td><td>0</td></tr>
<tr><td>>sp|P33981|TTK_HUMAN</td><td>1</td><td>9</td><td>9</td><td>9</td><td>8</td></tr>
<tr><td>>sp|P35226|BMI1_HUMA</td><td>7</td><td>13</td><td>10</td><td>10</td><td>13</td></tr>
<tr><td>>sp|P35250|RFC2_HUMA</td><td>11</td><td>9</td><td>9</td><td>9</td><td>8</td></tr>
<tr><td>>sp|P35251|RFC1_HUMA</td><td>5</td><td>12</td><td>12</td><td>12</td><td>12</td></tr>
<tr><td>>sp|P35659|DEK_HUMAN</td><td>8</td><td>13</td><td>13</td><td>13</td><td>9</td></tr>
<tr><td>>sp|P35908|K22E_HUMA</td><td>1</td><td>51</td><td>51</td><td>1</td><td>46</td></tr>
<tr><td>>tr|Q8TAS0|Q8TAS0_HU</td><td>4</td><td>5</td><td>5</td><td>5</td><td>5</td></tr>
<tr><td>>sp|P36578|RL4_HUMAN</td><td>10</td><td>20</td><td>20</td><td>20</td><td>18</td></tr>
<tr><td>>sp|P37108|SRP14_HUM</td><td>3</td><td>6</td><td>6</td><td>6</td><td>4</td></tr>
<tr><td>>sp|P38159|RBMX_HUM</td><td>13</td><td>13</td><td>13</td><td>13</td><td>11</td></tr>
<tr><td>>sp|P38432|COIL_HUMA</td><td>2</td><td>14</td><td>14</td><td>14</td><td>8</td></tr>
<tr><td>>sp|P39023|RL3_HUMAN</td><td>15</td><td>16</td><td>16</td><td>16</td><td>13</td></tr>
<tr><td>>sp|P39880|CUX1_HUMA</td><td>3</td><td>4</td><td>4</td><td>4</td><td>4</td></tr>
<tr><td>>tr|Q53H34|Q53H34_HU</td><td>11</td><td>6</td><td>6</td><td>6</td><td>6</td></tr>
<tr><td>>sp|P41162|ETV3_HUMA</td><td>1</td><td>2</td><td>2</td><td>2</td><td>2</td></tr>
<tr><td>>sp|P41208|CETN2_HUM</td><td>1</td><td>1</td><td>1</td><td>1</td><td>1</td></tr>
<tr><td>>sp|P42166|LAP2A_HUM</td><td>1</td><td>30</td><td>30</td><td>21</td><td>28</td></tr>
<tr><td>>sp|P42285|SK2L2_HUM</td><td>4</td><td>7</td><td>7</td><td>7</td><td>6</td></tr>
<tr><td>>sp|P42677|RS27_HUMA</td><td>6</td><td>2</td><td>2</td><td>2</td><td>2</td></tr>
<tr><td>>sp|P42695|CNDD3_HUM</td><td>4</td><td>13</td><td>13</td><td>13</td><td>13</td></tr>
<tr><td>>sp|P43246|MSH2_HUM</td><td>21</td><td>15</td><td>15</td><td>15</td><td>15</td></tr>
<tr><td>>sp|P46109|CRKL_HUMA</td><td>1</td><td>12</td><td>12</td><td>12</td><td>9</td></tr>
<tr><td>>tr|Q6IAX2|Q6IAX2_HUM</td><td>5</td><td>3</td><td>3</td><td>3</td><td>3</td></tr>
<tr><td>>sp|P46783|RS10_HUMA</td><td>6</td><td>3</td><td>3</td><td>3</td><td>3</td></tr>
<tr><td>>sp|P48059|LIMS1_HUM</td><td>14</td><td>3</td><td>3</td><td>3</td><td>3</td></tr>
<tr><td>>tr|Q8NE89|Q8NE89_HU</td><td>3</td><td>2</td><td>2</td><td>2</td><td>2</td></tr>
<tr><td>>sp|P48380|RFX3_HUMA</td><td>1</td><td>2</td><td>1</td><td>1</td><td>2</td></tr>
<tr><td>>sp|P49327|FAS_HUMAN</td><td>1</td><td>4</td><td>4</td><td>4</td><td>4</td></tr>
<tr><td>>sp|P49411|EFTU_HUMA</td><td>2</td><td>7</td><td>7</td><td>7</td><td>7</td></tr>
<tr><td>>sp|P49454|CENPF_HUM</td><td>3</td><td>3</td><td>3</td><td>3</td><td>2</td></tr>
<tr><td>>sp|P49458|SRP09_HUM</td><td>6</td><td>4</td><td>4</td><td>4</td><td>3</td></tr>
<tr><td>>tr|Q5U045|Q5U045_HU</td><td>13</td><td>6</td><td>2</td><td>2</td><td>3</td></tr>
<tr><td>>sp|P49711|CTCF_HUMA</td><td>1</td><td>1</td><td>1</td><td>1</td><td>1</td></tr>
<tr><td>>sp|P49750|YLPM1_HUM</td><td>7</td><td>8</td><td>8</td><td>8</td><td>8</td></tr>
<tr><td>>sp|P49756|RBM25_HUM</td><td>5</td><td>14</td><td>14</td><td>14</td><td>12</td></tr>
<tr><td>>sp|P49792|RBP2_HUMA</td><td>24</td><td>36</td><td>36</td><td>36</td><td>23</td></tr>
<tr><td>>sp|P50402|EMD_HUMA</td><td>3</td><td>11</td><td>11</td><td>11</td><td>11</td></tr>
<tr><td>>tr|V9GYF7|V9GYF7_HUI</td><td>5</td><td>1</td><td>1</td><td>1</td><td>1</td></tr>
<tr><td>>sp|P51571|SSRD_HUMA</td><td>2</td><td>5</td><td>5</td><td>5</td><td>5</td></tr>
<tr><td>>sp|P51587|BRCA2_HUM</td><td>18</td><td>32</td><td>32</td><td>32</td><td>30</td></tr>
<tr><td>>sp|P51858|HDGF_HUMA</td><td>10</td><td>7</td><td>6</td><td>6</td><td>4</td></tr>
<tr><td>>sp|P51991|ROA3_HUMA</td><td>2</td><td>19</td><td>19</td><td>2</td><td>12</td></tr>
</table>

>sp|P52272|HNRPM_HU	16	44	44	44	42
>sp|P52298|NCBP2_HUM	6	2	2	2	1
>sp|P52701|MSH6_HUM	11	21	21	21	20
>sp|P52952|NKX25_HUM	10	8	8	8	8
>sp|P53621|COPA_HUM	1	1	1	1	1
>tr|Q6IBA2|Q6IBA2_HUM	5	4	4	4	2
>sp|P54136|SYRC_HUMA	1	1	1	1	1
>sp|P55000|SLUR1_HUM	1	1	1	1	1
>tr|V9HW80|V9HW80_H	9	10	10	10	10
>sp|P55081|MFAP1_HUM	1	3	3	3	2
>sp|P55265|DSRAD_HUM	4	36	36	2	30
>sp|P55795|HNRH2_HUM	3	13	7	7	12
>sp|P56537|IF6_HUMAN	6	6	6	6	5
>sp|P57740|NU107_HUM	1	1	1	1	1
>tr|Q53ZD3|Q53ZD3_HU	6	3	2	2	2
>tr|Q6EZE9|Q6EZE9_HUM	3	2	2	2	0
>sp|P60468|SC61B_HUM	1	1	1	1	1
>tr|Q53GK6|Q53GK6_HU	53	20	1	1	18
>sp|P60866|RS20_HUMA	4	4	4	4	4
>tr|Q6IBH6|Q6IBH6_HUM	13	8	8	8	8
>sp|P61964|WDR5_HUM	3	10	10	10	10
>sp|P62136|PP1A_HUMA	14	6	2	2	6
>tr|Q5JR94|Q5JR94_HUM	4	9	9	9	8
>sp|P62263|RS14_HUMA	4	8	8	8	8
>sp|P62269|RS18_HUMA	4	9	9	9	8
>sp|P62273|RS29_HUMA	1	1	1	1	1
>sp|P62280|RS11_HUMA	4	10	10	10	9
>sp|P62306|RUXF_HUMA	1	2	2	2	0
>sp|P62314|SMD1_HUM	4	2	2	2	1
>sp|P62316|SMD2_HUM	3	4	4	4	3
>sp|P62318|SMD3_HUM	2	3	3	3	2
>sp|P62424|RL7A_HUMA	4	12	12	12	12
>sp|P62633|CNBP_HUMA	2	5	5	5	4
>sp|P62750|RL23A_HUM	9	9	9	9	9
>sp|P62851|RS25_HUMA	1	3	3	3	2
>sp|P62877|RBX1_HUMA	1	1	1	1	1
>sp|Q59GN2|R39L5_HUM	2	1	1	1	1
>sp|P62906|RL10A_HUM	3	6	6	6	6
>tr|Q5VVD0|Q5VVD0_HU	5	9	9	9	8
>sp|P62917|RL8_HUMAN	6	10	10	10	9
>tr|Q3MIH3|Q3MIH3_HU	41	7	1	1	7
>tr|Q5FWY2|Q5FWY2_HU	33	8	8	8	8
>tr|Q56VW8|Q56VW8_H	8	3	3	3	3
>tr|Q6FGH9|Q6FGH9_HU	6	5	5	5	4
>tr|Q8WVC2|Q8WVC2_H	4	2	2	2	1

>sp|P63261|ACTG_HUMA	10	20	20	1	18
>sp|P67809|YBOX1_HUM	18	6	6	6	6
>tr|Q6IPT9|Q6IPT9_HUM	38	21	21	13	20
>sp|P68366|TBA4A_HUM	5	21	21	4	20
>tr|Q8IZ29|Q8IZ29_HUM	5	24	24	0	24
>tr|Q5VUC3|Q5VUC3_HU	3	2	2	2	2
>sp|P78346|RPP30_HUM	4	7	7	7	7
>tr|Q5J8M4|Q5J8M4_HU	4	6	6	6	6
>sp|P78413|IRX4_HUMA	1	2	2	2	2
>sp|P78527|PRKDC_HUM	4	131	131	131	120
>sp|P80723|BASP1_HUM	1	1	1	1	0
>sp|P81605|DCD_HUMA	1	2	2	2	2
>sp|P82979|SARNP_HUM	6	4	4	4	0
>sp|Q00839|HNRPU_HUI	7	21	21	21	19
>sp|Q01081|U2AF1_HUM	9	10	10	10	7
>sp|Q01196|RUNX1_HUI	9	7	7	7	6
>sp|Q01650|LAT1_HUMA	10	5	5	5	4
>tr|Q59GG3|Q59GG3_HU	4	2	2	2	2
>sp|Q01780|EXOSX_HUM	5	8	8	8	7
>sp|Q02413|DSG1_HUM	1	9	9	9	7
>tr|Q71UH4|Q71UH4_HU	9	36	26	26	34
>sp|Q03164|KMT2A_HUI	43	29	29	28	27
>tr|Q05BU6|Q05BU6_HU	11	2	2	2	1
>sp|Q05639|EF1A2_HUM	2	11	3	3	10
>tr|Q05CR6|Q05CR6_HU	6	9	9	9	9
>sp|Q06330|SUH_HUMA	21	5	5	5	4
>sp|Q06587|RING1_HUM	5	17	13	13	16
>sp|Q06830|PRDX1_HUM	4	15	15	12	13
>sp|Q8IUG1|KRA13_HUM	4	2	2	2	0
>sp|Q08211|DHX9_HUM	4	10	10	10	6
>tr|Q9HB00|Q9HB00_HU	2	3	3	3	1
>sp|Q09028|RBBP4_HUM	9	9	9	5	9
>sp|Q09666|AHNK_HUM	12	12	12	12	11
>tr|Q6AHZ7|Q6AHZ7_HU	5	17	17	17	14
>sp|Q12830|BPTF_HUMA	13	22	22	22	20
>sp|Q12872|SFSWA_HUI	7	3	3	3	2
>sp|Q12873|CHD3_HUM	8	11	5	5	11
>sp|Q12947|FOXF2_HUM	2	4	4	3	4
>sp|Q13151|ROA0_HUM	1	6	6	6	6
>sp|Q13263|TIF1B_HUM	5	14	14	14	14
>tr|Q96F82|Q96F82_HUI	4	19	19	19	11
>sp|Q13428|TCOF_HUMA	2	18	18	2	14
>sp|Q13509|TBB3_HUMA	13	18	6	3	18
>sp|Q13535|ATR_HUMA	4	7	7	7	6
>sp|Q13573|SNW1_HUM	12	11	11	11	9

>tr|Q549U1|Q549U1_HU	2	7	5	5	6
>sp|Q13751|LAMB3_HUI	3	5	5	5	0
>sp|Q13761|RUNX3_HUI	7	6	6	6	6
>tr|Q5T0F3|Q5T0F3_HUI	4	11	11	11	11
>sp|Q9BVA1|TBB2B_HUI	14	19	2	1	19
>sp|Q14151|SAFB2_HUN	3	14	14	7	5
>sp|Q14257|RCN2_HUM.	3	6	6	6	5
>sp|Q14258|TRI25_HUM	3	4	4	4	2
>sp|Q14331|FRG1_HUM/	7	4	4	4	3
>sp|Q14432|PDE3A_HUN	3	2	2	2	2
>sp|Q14498|RBM39_HUI	31	23	23	23	22
>sp|Q14532|K1H2_HUM/	2	8	1	1	1
>sp|Q14669|TRIPC_HUM	3	25	25	19	22
>sp|Q14674|ESPL1_HUM	1	1	1	1	1
>sp|Q14676|MDC1_HUN	5	12	12	12	5
>sp|Q14677|EPN4_HUM/	1	2	2	2	2
>sp|Q14683|SMC1A_HUI	12	26	26	26	25
>sp|Q14684|RRP1B_HUN	2	26	26	26	21
>sp|Q14690|RRP5_HUM/	3	7	7	7	4
>sp|Q14692|BMS1_HUM	1	2	2	2	1
>sp|Q14781|CBX2_HUM/	1	47	47	47	46
>sp|Q14807|KIF22_HUM	12	19	19	19	16
>sp|Q14865|ARI5B_HUN	1	4	4	4	4
>sp|Q14966|ZN638_HUN	16	20	20	20	19
>sp|Q14980|NUMA1_HU	23	133	1	1	125
>sp|Q15050|RRS1_HUM/	1	2	2	2	2
>sp|Q15058|KIF14_HUM	1	1	1	1	1
>sp|Q15061|WDR43_HU	1	1	1	1	1
>sp|Q15072|OZF_HUMAI	3	5	5	5	5
>sp|Q15149|PLEC_HUM/	5	18	18	15	16
>tr|Q53SS8|Q53SS8_HUN	17	12	12	8	12
>sp|Q15654|TRIP6_HUM	4	2	2	2	1
>sp|Q15717|ELAV1_HUN	10	18	18	18	14
>tr|Q6FHQ0|Q6FHQ0_HU	7	9	5	5	9
>sp|Q1ED39|KNOP1_HUI	4	5	5	5	5
>sp|Q1KMD3|HNRL2_HU	2	8	8	8	6
>tr|Q1RMC9|Q1RMC9_H	3	6	6	6	6
>tr|Q1W6G4|Q1W6G4_H	9	11	4	4	9
>sp|Q2KHR3|QSER1_HUN	5	40	40	40	39
>tr|Q2L6I2|Q2L6I2_HUM	9	9	9	9	4
>sp|Q8IUC1|KR111_HUN	2	2	2	2	0
>sp|Q3ZCM7|TBB8_HUM	3	11	2	2	11
>sp|Q4VC44|FWCH1_HU	7	4	3	3	1
>sp|Q8IUC0|KR131_HUN	2	1	1	1	0
>sp|Q53EQ6|TIGD5_HUN	1	1	1	1	1

>sp|Q92908|GATA6_HUM	2	3	3	3	2
>tr|Q9BUN6|Q9BUN6_HU	4	1	1	1	1
>sp|Q9NYD6|HXC10_HUI	3	6	6	6	6
>sp|Q9Y5M8|SRPRB_HUI	3	3	3	3	3
>sp|Q56NI9|ESCO2_HUM	5	7	7	7	6
>sp|Q92922|SMRC1_HUI	7	16	8	8	16
>tr|Q59EC0|Q59EC0_HUI	2	35	1	1	30
>tr|Q59EJ3|Q59EJ3_HUM	1	23	1	0	23
>sp|Q9NRW3|ABC3C_HU	10	10	10	10	7
>sp|Q5BKZ1|ZN326_HUM	3	13	13	13	13
>sp|Q5C9Z4|NOM1_HUM	1	7	7	7	6
>tr|Q5H928|Q5H928_HU	2	1	1	1	0
>sp|Q5H9F3|BCORL_HUM	1	12	1	1	12
>sp|Q5QJE6|TDIF2_HUM	4	5	5	5	5
>sp|Q5RKV6|EXOS6_HUM	1	4	4	4	3
>sp|Q5SNT2|TM201_HUI	3	3	3	3	3
>sp|Q5SRE5|NU188_HUM	3	4	4	4	3
>tr|X6RGJ2|X6RGJ2_HUM	6	5	5	5	0
>sp|Q5SVZ6|ZMYM1_HU	5	10	10	10	10
>sp|Q5SY16|NOL9_HUM.	1	13	13	13	13
>sp|Q5T280|CI114_HUM	4	9	9	9	8
>sp|Q5T3I0|GPTC4_HUM	8	11	11	11	11
>sp|Q5T3J3|LRIF1_HUM/	1	3	3	3	3
>sp|Q5T440|CAF17_HUM	1	1	1	1	0
>sp|Q5T5X7|BEND3_HUM	1	3	3	3	3
>tr|Q5T670|Q5T670_HUI	2	2	2	2	2
>sp|Q5T749|KPRP_HUM/	1	11	11	11	10
>sp|Q5T750|XP32_HUM/	1	2	2	2	2
>sp|Q9BSD7|NTPCR_HUM	2	1	1	1	0
>sp|Q9UNX4|WDR3_HUM	3	2	2	2	0
>tr|Q5TEC6|Q5TEC6_HUI	1	3	3	1	3
>sp|Q5TGY3|AHDC1_HUI	1	8	8	8	7
>sp|Q5THK1|PR14L_HUM	4	6	6	6	6
>sp|Q5UIP0|RIF1_HUMA	5	54	54	54	49
>sp|Q5VTL8|PR38B_HUM	3	8	8	8	4
>sp|Q5VUA4|ZN318_HUI	1	8	8	8	8
>sp|Q5VV42|CDKAL_HUM	1	1	1	1	1
>sp|Q5VWN6|F208B_HU	2	20	20	20	17
>sp|Q5VZL5|ZMYM4_HU	2	13	13	13	13
>sp|Q96I25|SPF45_HUM.	5	3	3	3	2
>sp|Q5W0B1|RN219_HU	1	8	8	8	6
>tr|Q68CP0|Q68CP0_HU	6	6	6	6	5
>sp|Q63ZY3|KANK2_HUM	2	3	3	3	3
>sp|Q9ULW0|TPX2_HUM	4	33	33	33	32
>sp|Q68CP9|ARID2_HUM	8	8	8	8	8

>sp|Q69YH5|CDCA2_HUI	5	29	29	29	28
>sp|Q9H0U9|TSYL1_HUN	3	3	3	3	3
>sp|Q9Y285|SYFA_HUMA	5	5	5	5	4
>sp|Q6IQ49|SDE2_HUMA	1	1	1	1	0
>sp|Q6NW34|NEPRO_HU	11	5	5	5	5
>sp|Q6NZY4|ZCHC8_HUN	6	9	9	9	9
>sp|Q6P0N0|M18BP_HU	4	6	6	6	6
>sp|Q6P2Q9|PRP8_HUM	7	15	15	15	14
>sp|Q6P4F7|RHGBA_HUI	3	6	6	6	6
>sp|Q6PK81|ZN773_HUN	1	1	1	1	1
>sp|Q6RFH5|WDR74_HU	5	5	5	5	4
>sp|Q6UB99|ANR11_HUI	1	1	1	1	1
>sp|Q6UN15|FIP1_HUMA	3	7	7	7	7
>sp|Q6UWP8|SBSN_HUN	1	2	2	2	2
>sp|Q6ZRF7|ZN818_HUN	1	1	1	1	0
>sp|Q6ZVX9|PAQR9_HUI	1	1	1	1	0
>tr|Q70L73|Q70L73_HUI	1	16	1	1	15
>tr|Q71RB6|Q71RB6_HU	2	2	2	2	2
>sp|Q7L3T8|SYPM_HUM.	1	4	4	4	0
>sp|Q7L4I2|RSRC2_HUM	5	3	3	3	2
>sp|Q7Z2Z1|TICRR_HUM	2	12	12	12	12
>sp|Q7Z333|SETX_HUMA	4	11	11	11	11
>sp|Q7Z3K3|POGZ_HUM	3	4	4	4	3
>sp|Q7Z589|EMSY_HUM	6	18	18	6	18
>sp|Q7Z5J4|RAI1_HUMA	3	7	7	7	6
>sp|Q7Z6E9|RBBP6_HUN	5	3	3	3	1
>sp|Q7Z7G8|VP13B_HUN	1	1	1	1	1
>sp|Q86SE9|PCGF5_HUN	1	1	1	1	1
>sp|Q86SJ6|DSG4_HUMA	1	3	3	3	0
>sp|Q86T29|ZN605_HUN	6	2	1	0	2
>sp|Q86TJ2|TAD2B_HUN	2	2	2	2	2
>sp|Q86U42|PABP2_HUI	4	3	3	3	3
>sp|Q86U86|PB1_HUMA	11	18	18	18	17
>sp|Q86UT8|CCD84_HUI	4	8	8	8	4
>sp|Q86V81|THOC4_HUI	2	7	7	7	5
>sp|Q86Y79|PTH_HUMA	5	6	6	6	4
>sp|Q86YC2|PALB2_HUN	2	4	4	4	4
>sp|Q8IUF8|MINA_HUMA	3	2	2	2	0
>sp|Q8IVT2|MISP_HUMA	1	11	11	11	11
>tr|Q8IWP6|Q8IWP6_HU	14	24	1	1	24
>sp|Q8IWZ3|ANKH1_HUI	16	4	4	4	4
>sp|Q8IXB1|DJC10_HUM	6	28	28	7	26
>sp|Q8IY57|YAF2_HUMA	6	2	2	2	2
>sp|Q8IY81|SPB1_HUMA	2	7	7	7	5
>sp|Q8IYH5|ZZZ3_HUMA	3	3	3	3	2

<table>
<tr><td>>sp|Q8IYL3|CA174_HUM</td><td>1</td><td>3</td><td>3</td><td>3</td><td>3</td></tr>
<tr><td>>sp|Q8IZT6|ASPM_HUM</td><td>6</td><td>12</td><td>12</td><td>12</td><td>10</td></tr>
<tr><td>>tr|Q8NFV1|Q8NFV1_HU</td><td>3</td><td>3</td><td>1</td><td>1</td><td>0</td></tr>
<tr><td>>sp|Q8N1F7|NUP93_HU</td><td>11</td><td>7</td><td>7</td><td>7</td><td>6</td></tr>
<tr><td>>sp|Q8N556|AFAP1_HU</td><td>4</td><td>8</td><td>8</td><td>8</td><td>6</td></tr>
<tr><td>>sp|Q8N5F7|NKAP_HUM</td><td>4</td><td>6</td><td>6</td><td>6</td><td>5</td></tr>
<tr><td>>sp|Q8N5L8|RP25L_HU</td><td>1</td><td>2</td><td>2</td><td>2</td><td>2</td></tr>
<tr><td>>sp|Q8N5Y2|MS3L1_HU</td><td>7</td><td>3</td><td>3</td><td>3</td><td>3</td></tr>
<tr><td>>sp|Q8N726|ARF_HUMA</td><td>1</td><td>5</td><td>5</td><td>2</td><td>5</td></tr>
<tr><td>>sp|Q8N883|ZN614_HU</td><td>2</td><td>3</td><td>2</td><td>0</td><td>3</td></tr>
<tr><td>>sp|Q8NAF0|ZN579_HU</td><td>1</td><td>3</td><td>3</td><td>3</td><td>3</td></tr>
<tr><td>>sp|Q8NAV1|PR38A_HU</td><td>1</td><td>2</td><td>2</td><td>2</td><td>2</td></tr>
<tr><td>>sp|Q8NB16|MLKL_HUM</td><td>5</td><td>4</td><td>4</td><td>4</td><td>1</td></tr>
<tr><td>>sp|Q8NB78|KDM1B_HU</td><td>1</td><td>1</td><td>1</td><td>1</td><td>1</td></tr>
<tr><td>>sp|Q8NB90|SPAT5_HU</td><td>9</td><td>4</td><td>4</td><td>4</td><td>4</td></tr>
<tr><td>>sp|Q8NBI6|XXLT1_HUM</td><td>2</td><td>3</td><td>3</td><td>3</td><td>1</td></tr>
<tr><td>>sp|Q8NC51|PAIRB_HU</td><td>5</td><td>6</td><td>6</td><td>6</td><td>5</td></tr>
<tr><td>>sp|Q8NCD3|HJURP_HU</td><td>3</td><td>4</td><td>4</td><td>4</td><td>3</td></tr>
<tr><td>>sp|Q8NCN4|RN169_HU</td><td>2</td><td>8</td><td>8</td><td>8</td><td>7</td></tr>
<tr><td>>sp|Q8NDX5|PHC3_HUM</td><td>13</td><td>25</td><td>22</td><td>22</td><td>25</td></tr>
<tr><td>>sp|Q8NDX6|ZN740_HU</td><td>1</td><td>6</td><td>6</td><td>6</td><td>4</td></tr>
<tr><td>>sp|Q8NEP9|ZN555_HU</td><td>1</td><td>1</td><td>1</td><td>1</td><td>1</td></tr>
<tr><td>>sp|Q8NFC6|BD1L1_HU</td><td>1</td><td>2</td><td>2</td><td>2</td><td>0</td></tr>
<tr><td>>sp|Q8NG31|CASC5_HU</td><td>5</td><td>30</td><td>30</td><td>30</td><td>28</td></tr>
<tr><td>>sp|Q8TD26|CHD6_HUM</td><td>4</td><td>23</td><td>17</td><td>15</td><td>23</td></tr>
<tr><td>>sp|Q8TDD1|DDX54_HU</td><td>3</td><td>6</td><td>6</td><td>6</td><td>4</td></tr>
<tr><td>>sp|Q8TDN6|BRX1_HUM</td><td>4</td><td>10</td><td>10</td><td>10</td><td>6</td></tr>
<tr><td>>sp|Q8TE73|DYH5_HUM</td><td>1</td><td>1</td><td>1</td><td>1</td><td>1</td></tr>
<tr><td>>sp|Q8TEM1|PO210_HU</td><td>1</td><td>2</td><td>2</td><td>2</td><td>2</td></tr>
<tr><td>>sp|Q8WUA4|TF3C2_HU</td><td>6</td><td>3</td><td>3</td><td>3</td><td>3</td></tr>
<tr><td>>tr|S5FMB0|S5FMB0_HU</td><td>5</td><td>7</td><td>7</td><td>7</td><td>7</td></tr>
<tr><td>>sp|Q8WUU5|GATD1_HU</td><td>1</td><td>4</td><td>4</td><td>4</td><td>4</td></tr>
<tr><td>>sp|Q8WX93|PALLD_HU</td><td>10</td><td>24</td><td>24</td><td>23</td><td>16</td></tr>
<tr><td>>sp|Q8WXI9|P66B_HUM</td><td>2</td><td>6</td><td>6</td><td>6</td><td>5</td></tr>
<tr><td>>sp|Q8WYP5|ELYS_HUM</td><td>7</td><td>76</td><td>76</td><td>76</td><td>75</td></tr>
<tr><td>>sp|Q92466|DDB2_HUM</td><td>5</td><td>2</td><td>2</td><td>2</td><td>1</td></tr>
<tr><td>>sp|Q92522|H1X_HUMA</td><td>1</td><td>4</td><td>4</td><td>4</td><td>4</td></tr>
<tr><td>>sp|Q92576|PHF3_HUM</td><td>12</td><td>27</td><td>27</td><td>27</td><td>20</td></tr>
<tr><td>>sp|Q92769|HDAC2_HU</td><td>12</td><td>10</td><td>10</td><td>6</td><td>9</td></tr>
<tr><td>>sp|Q92878|RAD50_HU</td><td>8</td><td>8</td><td>8</td><td>8</td><td>4</td></tr>
<tr><td>>sp|Q92945|FUBP2_HU</td><td>12</td><td>37</td><td>37</td><td>33</td><td>31</td></tr>
<tr><td>>sp|Q93074|MED12_HU</td><td>4</td><td>5</td><td>5</td><td>5</td><td>3</td></tr>
<tr><td>>sp|Q969H6|POP5_HUM</td><td>1</td><td>1</td><td>1</td><td>1</td><td>1</td></tr>
<tr><td>>sp|Q96AG4|LRC59_HU</td><td>1</td><td>1</td><td>1</td><td>1</td><td>1</td></tr>
<tr><td>>sp|Q96AY2|EME1_HUM</td><td>1</td><td>1</td><td>1</td><td>1</td><td>1</td></tr>
</table>

>sp|Q96BN2|TADA1_HUI	1	2	2	2	2
>sp|Q96DY7|MTBP_HUM	1	4	4	4	4
>sp|Q96EK4|THA11_HUN	2	4	4	4	4
>sp|Q99575|POP1_HUM.	3	14	14	14	7
>sp|Q96FQ6|S10AG_HUN	1	3	3	3	0
>sp|Q96GD4|AURKB_HU	8	6	6	6	6
>sp|Q96GM8|TOE1_HUN	3	14	14	14	11
>sp|Q96H79|ZCCHL_HUN	1	4	4	4	2
>sp|Q96HA1|P121A_HUN	10	6	6	6	6
>sp|Q96HS1|PGAM5_HU	2	8	8	8	8
>sp|Q96IR2|ZN845_HUM	1	1	1	1	1
>sp|Q96IZ0|PAWR_HUM.	1	1	1	1	1
>sp|Q96JM3|CHAP1_HUI	2	6	6	6	6
>tr|Q96K97|Q96K97_HUI	5	2	2	2	2
>sp|Q96KM6|Z512B_HUI	1	4	4	4	4
>sp|Q96L91|EP400_HUN	3	15	15	15	15
>sp|Q96MG7|MAGG1_H	1	2	2	2	2
>sp|Q96PK6|RBM14_HUI	7	24	24	24	20
>sp|Q96PU4|UHRF2_HUI	3	3	2	2	1
>sp|Q96PU8|QKI_HUMAI	8	2	2	2	2
>sp|Q96QE3|ATAD5_HUI	3	22	22	22	22
>sp|Q96SK2|TM209_HUN	3	3	3	3	3
>sp|Q96SL8|FIZ1_HUMAI	5	5	5	5	5
>sp|Q96ST3|SIN3A_HUM	2	8	8	8	6
>sp|Q96T58|MINT_HUM.	3	16	16	16	14
>sp|Q99496|RING2_HUN	2	22	22	18	22
>sp|Q99549|MPP8_HUM	4	4	4	4	4
>sp|Q9BQ04|RBM4B_HU	1	5	1	1	4
>sp|Q9BQ61|CS043_HUN	5	6	6	6	3
>sp|Q9BQ69|MACD1_HL	1	3	3	3	2
>sp|Q9BQG0|MBB1A_HL	7	38	38	38	32
>tr|Q9BR63|Q9BR63_HU	4	11	11	11	10
>sp|Q9BRJ7|SDOS_HUM/	8	12	12	12	9
>sp|Q9BRP1|PDD2L_HUN	3	7	7	7	5
>sp|Q9BTC0|DIDO1_HUN	1	25	25	25	23
>sp|Q9BTL3|RAM_HUMA	1	1	1	1	1
>sp|Q9BU76|MMTA2_HL	1	10	10	10	8
>sp|Q9BUF5|TBB6_HUM.	14	16	5	3	16
>tr|U3KQC1|U3KQC1_HL	8	8	8	8	8
>sp|Q9BVJ6|UT14A_HUN	7	10	10	10	8
>sp|Q9BVQ7|SPA5L_HUN	2	6	6	6	6
>sp|Q9BWF3|RBM4_HUN	8	7	7	3	6
>sp|Q9BWJ5|SF3B5_HUN	1	4	4	4	4
>sp|Q9BXK1|KLF16_HUN	3	4	4	4	3
>sp|Q9BY42|RTF2_HUM/	3	7	1	1	5

<table>
<tr><td>>sp|Q9BY89|K1671_HUM</td><td>1</td><td>18</td><td>18</td><td>18</td><td>18</td></tr>
<tr><td>>sp|Q9BYB4|GNB1L_HUI</td><td>2</td><td>6</td><td>6</td><td>6</td><td>3</td></tr>
<tr><td>>sp|Q9BYE7|PCGF6_HUM</td><td>1</td><td>2</td><td>2</td><td>2</td><td>2</td></tr>
<tr><td>>tr|Q9P1X0|Q9P1X0_HUI</td><td>3</td><td>2</td><td>2</td><td>2</td><td>2</td></tr>
<tr><td>>sp|Q9BYJ9|YTHD1_HUM</td><td>10</td><td>5</td><td>3</td><td>3</td><td>3</td></tr>
<tr><td>>sp|Q9BYW2|SETD2_HUI</td><td>5</td><td>25</td><td>25</td><td>25</td><td>25</td></tr>
<tr><td>>sp|Q9BZ95|NSD3_HUM</td><td>3</td><td>11</td><td>10</td><td>10</td><td>11</td></tr>
<tr><td>>sp|Q9C005|DPY30_HUM</td><td>2</td><td>3</td><td>3</td><td>3</td><td>3</td></tr>
<tr><td>>sp|Q9GZS1|RPA49_HUM</td><td>5</td><td>5</td><td>5</td><td>5</td><td>2</td></tr>
<tr><td>>sp|Q9H0A0|NAT10_HUI</td><td>5</td><td>10</td><td>10</td><td>10</td><td>7</td></tr>
<tr><td>>sp|Q9H0C8|ILKAP_HUM</td><td>4</td><td>2</td><td>2</td><td>2</td><td>1</td></tr>
<tr><td>>sp|Q9H147|TDIF1_HUM</td><td>5</td><td>7</td><td>7</td><td>7</td><td>7</td></tr>
<tr><td>>sp|Q9H1A4|APC1_HUM</td><td>3</td><td>3</td><td>3</td><td>3</td><td>2</td></tr>
<tr><td>>sp|Q9H3K6|BOLA2_HUI</td><td>5</td><td>5</td><td>5</td><td>5</td><td>5</td></tr>
<tr><td>>sp|Q9H4L4|SENP3_HUM</td><td>6</td><td>9</td><td>9</td><td>9</td><td>8</td></tr>
<tr><td>>sp|Q9H4Z2|ZN335_HUM</td><td>2</td><td>3</td><td>3</td><td>3</td><td>3</td></tr>
<tr><td>>sp|Q9H501|ESF1_HUMA</td><td>4</td><td>3</td><td>3</td><td>3</td><td>2</td></tr>
<tr><td>>sp|Q9H582|ZN644_HUM</td><td>9</td><td>43</td><td>43</td><td>43</td><td>43</td></tr>
<tr><td>>sp|Q9H5V9|CX056_HUM</td><td>2</td><td>3</td><td>3</td><td>3</td><td>2</td></tr>
<tr><td>>sp|Q9H6F5|CCD86_HUM</td><td>1</td><td>2</td><td>2</td><td>2</td><td>2</td></tr>
<tr><td>>sp|Q9H6R4|NOL6_HUM</td><td>5</td><td>25</td><td>25</td><td>25</td><td>10</td></tr>
<tr><td>>sp|Q9H7E9|CH033_HUM</td><td>1</td><td>3</td><td>3</td><td>3</td><td>3</td></tr>
<tr><td>>sp|Q9H7L9|SDS3_HUMA</td><td>1</td><td>1</td><td>1</td><td>1</td><td>1</td></tr>
<tr><td>>sp|Q9H7Z6|KAT8_HUMA</td><td>1</td><td>1</td><td>1</td><td>1</td><td>1</td></tr>
<tr><td>>sp|Q9H8M2|BRD9_HUM</td><td>6</td><td>8</td><td>8</td><td>8</td><td>8</td></tr>
<tr><td>>sp|Q9H9B1|EHMT1_HU</td><td>15</td><td>40</td><td>40</td><td>39</td><td>39</td></tr>
<tr><td>>sp|Q9H9L3|I20L2_HUM</td><td>1</td><td>8</td><td>8</td><td>8</td><td>6</td></tr>
<tr><td>>sp|Q9HA92|RSAD1_HUI</td><td>4</td><td>3</td><td>3</td><td>3</td><td>1</td></tr>
<tr><td>>sp|Q9HC52|CBX8_HUM</td><td>4</td><td>21</td><td>21</td><td>20</td><td>21</td></tr>
<tr><td>>sp|Q9HCD6|TANC2_HUI</td><td>4</td><td>3</td><td>3</td><td>3</td><td>3</td></tr>
<tr><td>>sp|Q9HCI7|MSL2_HUMA</td><td>5</td><td>6</td><td>6</td><td>6</td><td>6</td></tr>
<tr><td>>sp|Q9HCK8|CHD8_HUM</td><td>2</td><td>11</td><td>1</td><td>1</td><td>11</td></tr>
<tr><td>>sp|Q9NPC8|SIX2_HUMA</td><td>3</td><td>3</td><td>3</td><td>3</td><td>2</td></tr>
<tr><td>>sp|Q9NPD3|EXOS4_HUI</td><td>3</td><td>4</td><td>4</td><td>4</td><td>3</td></tr>
<tr><td>>sp|Q9NQS7|INCE_HUMA</td><td>1</td><td>1</td><td>1</td><td>1</td><td>1</td></tr>
<tr><td>>sp|Q9NQT5|EXOS3_HUI</td><td>2</td><td>2</td><td>2</td><td>2</td><td>2</td></tr>
<tr><td>>sp|Q9NR12|PDLI7_HUM</td><td>4</td><td>2</td><td>2</td><td>2</td><td>2</td></tr>
<tr><td>>sp|Q9NR30|DDX21_HUI</td><td>3</td><td>36</td><td>36</td><td>34</td><td>28</td></tr>
<tr><td>>sp|Q9NR48|ASH1L_HUM</td><td>2</td><td>15</td><td>15</td><td>15</td><td>15</td></tr>
<tr><td>>sp|Q9NRE2|TSH2_HUM</td><td>1</td><td>1</td><td>1</td><td>1</td><td>1</td></tr>
<tr><td>>sp|Q9NSI2|F207A_HUM</td><td>1</td><td>2</td><td>2</td><td>2</td><td>2</td></tr>
<tr><td>>sp|Q9NU22|MDN1_HUI</td><td>5</td><td>31</td><td>31</td><td>31</td><td>27</td></tr>
<tr><td>>sp|Q9NUI1|DECR2_HUM</td><td>7</td><td>8</td><td>8</td><td>8</td><td>2</td></tr>
<tr><td>>sp|Q9NV56|MRGBP_HU</td><td>1</td><td>2</td><td>2</td><td>2</td><td>1</td></tr>
<tr><td>>sp|Q9NVV4|PAPD1_HUI</td><td>1</td><td>3</td><td>3</td><td>3</td><td>1</td></tr>
</table>

>sp|Q9NVW2|RNF12_HU	1	3	3	3	3
>sp|Q9NWH9|SLTM_HUI	12	6	6	6	5
>sp|Q9NWT1|PK1IP_HUI	1	1	1	1	1
>sp|Q9NXV6|CARF_HUM	4	5	5	5	0
>sp|Q9NXW2|DJB12_HUI	5	3	3	3	3
>sp|Q9NXX6|NSE4A_HUI	1	1	1	1	1
>sp|Q9NY61|AATF_HUM	3	5	5	5	5
>sp|Q9NY74|ETAA1_HUI	1	6	6	6	6
>sp|Q9NYF8|BCLF1_HUI	12	19	18	18	18
>sp|Q9NYP9|MS18A_HU	1	2	2	2	2
>sp|Q9NZB2|F120A_HUI	2	2	2	2	2
>sp|Q9NZT1|CALL5_HUI	2	2	2	2	1
>sp|Q9P0U3|SENP1_HUI	5	12	12	12	12
>sp|Q9UBD5|ORC3_HUI	6	5	5	5	5
>sp|Q9UBL6|CPNE7_HUI	17	4	4	4	4
>sp|Q9UBS4|DJB11_HUI	2	2	2	2	2
>sp|Q9UBV8|PEF1_HUM	1	5	5	5	4
>sp|Q9UEG4|ZN629_HUI	1	2	2	2	2
>sp|Q9UFC0|LRWD1_HU	5	7	7	7	5
>sp|Q9UGM3|DMBT1_HI	1	1	1	1	0
>sp|Q9UHF7|TRPS1_HUI	4	2	2	2	2
>sp|Q9UHR4|BI2L1_HUI	1	1	1	1	0
>sp|Q9UIG0|BAZ1B_HUI	1	13	13	13	12
>sp|Q9UK61|TASOR_HUI	1	20	20	3	19
>sp|Q9UKJ3|GPTC8_HUI	2	6	6	6	6
>sp|Q9UKN8|TF3C4_HUI	4	4	4	4	4
>sp|Q9ULL5|PRR12_HUI	1	8	8	8	6
>sp|Q9ULX6|AKP8L_HUI	7	12	12	12	11
>sp|Q9UMS4|PRP19_HUI	5	14	14	14	14
>sp|Q9UQ35|SRRM2_HU	12	31	31	31	27
>sp|Q9Y230|RUVB2_HUI	7	20	20	20	20
>sp|Q9Y232|CDYL1_HUI	1	1	1	1	0
>sp|Q9Y2F5|ICE1_HUMA	2	8	8	8	8
>sp|Q9Y2K1|ZBTB1_HUI	2	4	4	4	4
>sp|Q9Y2R4|DDX52_HUI	7	16	16	16	11
>sp|Q9Y2S7|PDIP2_HUM	3	13	13	13	11
>sp|Q9Y2W1|TR150_HUI	5	22	22	21	17
>sp|Q9Y2X3|NOP58_HUI	5	19	19	19	19
>sp|Q9Y2X9|ZN281_HUI	4	27	27	27	27
>sp|Q9Y383|LC7L2_HUM	10	17	17	10	15
>sp|Q9Y3A2|UTP11_HUI	1	1	1	1	1
>sp|Q9Y3A4|RRP7A_HUI	3	10	10	10	4
>sp|Q9Y3B4|SF3B6_HUI	1	2	2	2	2
>sp|Q9Y3I0|RTCB_HUMA	4	3	3	3	2
>sp|Q9Y3U8|RL36_HUMA	2	3	3	3	3

>tr|X6R700|X6R700_HUM	4	3	3	3	2
>sp|Q9Y446|PKP3_HUMA	7	11	11	11	6
>sp|Q9Y4B5|MTCL1_HUM	11	24	24	24	22
>sp|Q9Y4E5|ZN451_HUM	5	4	4	4	3
>sp|Q9Y4W2|LAS1L_HUM	5	18	18	18	18
>sp|Q9Y5B9|SP16H_HUM	5	21	21	21	18
>sp|Q9Y5Q8|TF3C5_HUM	8	6	6	6	6
>sp|Q9Y5Q9|TF3C3_HUM	4	6	6	6	4
>sp|Q9Y6A4|CFA20_HUM	1	1	1	1	0
>tr|A0A076MIK7|A0A076	2	1	1	1	1
>Q9D646 TREMBL:Q9D64	13	1	1	1	0
>tr|A0A0A0MSD6|A0A0A	2	1	1	1	1
>sp|A1L453|PRS38_HUM	1	1	1	1	0
>tr|A7MD96|A7MD96_H	1	1	1	1	1
>tr|Q2KHM5|Q2KHM5_H	7	2	2	2	1
>tr|B4E2A9|B4E2A9_HUM	5	1	1	1	0
>tr|Q9Y4T0|Q9Y4T0_HUM	10	1	1	1	0
>tr|E9PG84|E9PG84_HUM	2	1	1	1	1
>tr|H0YKZ9|H0YKZ9_HUM	3	1	1	1	0
>tr|Q59H44|Q59H44_HU	2	1	1	1	1
>sp|P60602|ROMO1_HU	1	1	1	1	1
>tr|Q5UW39|Q5UW39_H	2	1	1	1	0
>tr|Q8N930|Q8N930_HU	1	1	1	1	1
>sp|Q9H7M6|ZSWM4_HU	1	1	1	1	1
>sp|Q9NUG4|CCM2L_HU	1	1	1	1	1
>sp|Q9P219|DAPLE_HUM	1	1	1	1	1
>tr|S4R341|S4R341_HUM	1	3	1	1	3
>tr|S4R3H4|S4R3H4_HUM	8	14	14	14	7
>tr|X6RAL5|X6RAL5_HUM	5	8	8	8	6

Peptides CBX	Peptides CBX	Peptides WT:	Peptides WT:	Peptides WT:	Razor + uniqu	Razor + uniqu
4	4	2	3	3	4	4
3	3	1	1	2	3	3
2	2	1	1	1	2	2
5	5	10	10	10	5	5
5	5	5	4	5	5	5
2	2	1	1	1	2	2
1	1	0	0	0	1	1
5	6	5	4	5	6	5
5	5	6	6	7	5	5
1	2	0	0	0	2	1
2	2	3	4	4	2	2
2	2	3	3	3	1	1
4	4	4	3	4	4	4
1	1	1	1	1	1	1
3	3	0	0	0	3	3
0	1	0	0	0	1	0
7	9	4	5	8	9	7
0	0	0	0	0	1	0
21	21	5	5	6	22	21
6	6	4	3	3	5	6
6	5	5	6	6	5	6
4	4	3	3	3	1	1
16	16	18	15	18	16	16
2	2	0	0	0	2	2
8	8	0	0	0	9	8
1	1	1	1	1	1	1
7	8	2	2	2	8	7
1	1	0	0	0	1	1
5	3	1	2	2	4	5
8	8	0	0	0	8	8
8	9	1	0	0	9	8
2	1	0	0	0	1	1
1	2	0	0	0	1	0
2	2	2	2	2	2	2
3	3	2	2	2	2	3
1	1	0	0	0	1	1
5	5	4	5	5	5	5
2	2	0	0	0	2	2
1	1	0	0	0	1	1
4	4	3	2	4	4	4
1	1	0	0	0	1	1
16	16	0	0	0	15	16
1	1	1	0	2	1	1
3	3	5	5	7	2	2

7	7	2	3	3	7	7
1	1	1	1	1	1	1
2	2	3	3	3	2	2
2	2	0	0	0	2	2
9	9	0	0	0	8	8
2	2	0	0	0	2	2
2	2	2	1	1	1	1
1	1	3	3	3	1	1
5	4	5	4	5	4	5
4	4	0	0	0	4	4
6	6	1	1	1	6	6
1	2	1	0	1	2	1
7	7	5	5	5	7	7
1	1	0	0	0	1	1
2	1	15	3	2	0	0
2	2	0	0	1	2	2
2	4	5	7	6	4	2
4	3	3	2	2	4	4
1	1	0	0	0	1	1
8	8	6	6	6	8	8
16	16	0	0	0	16	16
5	5	7	2	4	3	3
1	1	1	0	1	1	1
1	1	0	0	0	1	1
4	4	0	0	0	4	4
19	21	23	23	23	18	19
2	2	0	0	0	2	2
8	9	5	6	5	8	8
4	5	3	3	3	5	4
1	1	0	0	0	1	1
2	2	0	0	0	2	2
1	2	0	0	0	2	1
9	9	0	0	0	9	9
6	6	4	4	4	6	6
4	3	1	1	1	5	4
1	1	0	0	0	1	1
5	5	3	3	3	5	5
3	3	5	4	5	3	3
3	3	1	0	0	2	2
7	7	4	4	4	7	7
2	2	0	0	0	2	2
5	5	1	1	1	5	5
6	6	1	1	0	6	6
2	2	0	0	0	2	2
2	2	1	0	1	2	2

0	0	2	2	2	0	0
3	3	3	3	4	3	3
4	4	3	3	4	2	2
1	1	1	2	2	1	1
8	7	0	0	0	7	8
1	1	1	1	1	1	1
16	16	18	17	18	16	16
0	1	0	0	0	1	0
3	3	4	4	3	3	3
5	5	0	0	0	5	5
2	1	0	0	0	2	2
12	12	3	3	3	13	12
2	1	1	1	1	2	2
2	2	5	4	4	3	2
3	3	4	4	4	3	3
27	27	0	0	0	27	27
2	2	0	0	0	2	2
12	12	15	14	18	10	12
5	6	5	5	6	6	5
0	0	2	1	1	0	0
4	5	0	0	0	5	4
6	6	2	1	2	6	6
7	8	3	4	4	6	7
1	1	1	1	1	2	1
4	4	0	0	0	4	4
2	2	0	0	0	2	2
2	2	1	2	2	2	2
11	10	5	5	6	12	11
16	16	8	8	8	16	16
14	13	12	12	13	14	14
3	3	0	0	0	3	3
3	1	1	1	1	2	3
1	2	0	0	0	2	1
1	1	0	0	0	1	1
2	1	0	0	0	1	2
2	2	0	0	0	2	2
0	1	0	0	0	1	0
1	1	1	1	1	1	1
129	132	70	72	77	126	129
0	0	1	1	2	0	0
4	4	0	0	0	4	4
1	1	1	1	1	1	1
1	1	0	0	0	1	1
18	19	14	13	14	19	18
1	0	1	1	0	1	1

1	1	1	1	1	1	1
13	14	4	4	4	12	13
6	6	5	5	5	5	6
3	2	3	4	4	1	2
2	2	0	0	0	2	2
1	1	1	1	1	1	1
1	1	0	0	0	2	1
8	8	0	0	0	8	8
1	1	0	0	0	1	1
1	1	2	2	2	1	1
4	4	0	0	0	4	4
3	3	0	0	0	3	3
3	3	1	1	1	3	3
3	3	0	0	0	3	3
1	1	3	3	3	1	1
1	1	0	0	0	1	1
13	13	16	14	17	12	13
2	2	1	0	1	2	2
24	23	20	17	21	23	24
26	26	5	5	6	23	26
3	3	1	1	1	3	3
1	1	1	0	1	1	1
5	6	3	3	4	5	5
11	13	10	10	12	10	9
2	2	0	0	0	1	1
3	3	0	0	0	4	3
25	25	0	0	0	15	16
1	1	0	0	1	1	1
8	7	4	3	4	7	8
1	1	1	1	1	1	1
2	3	0	0	0	2	2
2	2	0	0	0	2	2
2	2	1	1	1	2	2
6	6	0	0	0	6	6
1	0	2	2	2	1	1
2	2	1	0	1	2	2
0	1	0	0	0	1	0
7	8	3	2	4	9	6
8	8	0	0	0	8	8
3	3	1	2	2	3	3
2	2	1	2	2	1	2
2	2	1	1	1	0	2
4	4	2	3	3	4	4
0	0	3	2	2	0	0
11	11	10	9	13	11	11

7	7	9	9	8	7	7
5	6	11	13	13	6	5
5	5	6	6	7	5	5
2	2	0	0	0	2	2
0	0	3	0	0	0	0
5	4	0	0	0	5	5
10	10	4	5	5	11	10
10	10	10	10	10	9	10
2	2	1	1	1	2	2
5	5	0	0	0	6	5
8	8	1	2	2	7	8
2	2	0	0	0	2	2
5	5	0	0	0	5	5
1	1	0	0	0	1	1
16	16	1	1	1	17	16
1	1	0	0	0	1	1
10	10	1	2	2	9	10
2	2	0	0	0	2	2
2	2	0	0	0	2	2
25	22	8	7	8	24	25
8	9	1	0	0	9	8
17	19	16	16	18	17	17
2	2	0	0	0	2	2
23	22	23	22	25	23	23
2	2	0	0	0	2	2
8	8	9	10	10	8	8
0	0	2	2	2	0	0
5	5	4	4	4	5	5
5	6	8	10	11	6	5
12	12	5	6	6	10	11
10	11	13	11	12	10	10
5	5	6	6	5	5	5
3	2	2	2	2	3	3
15	14	10	12	11	15	15
0	0	1	1	1	0	0
1	1	1	1	1	1	1
2	2	1	1	1	2	2
11	12	10	10	10	12	11
2	2	4	4	3	2	2
12	13	13	13	13	3	3
8	9	3	4	6	9	8
0	0	1	1	1	0	0
5	5	5	3	5	5	5
1	1	1	1	1	1	1
12	11	6	6	6	11	12

6	6	6	5	6	6	6
2	0	1	1	0	2	2
2	2	1	1	1	1	1
5	5	0	0	1	5	5
10	10	0	0	0	10	10
6	6	1	1	0	6	6
2	2	4	4	4	2	2
1	1	0	1	1	1	1
2	3	0	0	0	2	2
0	0	4	4	4	0	0
7	6	1	0	1	7	7
1	2	0	0	0	1	1
0	0	2	2	2	0	0
4	4	0	0	0	4	4
2	1	0	0	0	1	2
8	8	0	0	0	7	8
3	3	3	3	3	3	3
5	4	4	3	4	4	5
2	2	0	0	0	2	2
36	38	2	1	2	37	35
6	6	0	0	0	6	6
11	11	1	1	1	10	11
0	1	1	1	1	0	0
1	1	0	0	0	1	1
1	1	0	0	0	1	1
1	1	0	0	0	1	1
1	1	2	2	2	1	1
1	1	0	0	0	1	1
7	7	0	0	0	7	7
1	2	0	0	0	1	1
2	2	0	0	0	1	1
2	2	0	0	0	2	2
0	0	5	5	5	0	0
10	10	9	7	8	10	10
3	3	2	1	3	2	2
1	1	2	1	2	1	1
1	2	1	1	1	1	1
1	2	0	0	0	2	1
2	3	0	0	0	3	2
1	1	0	0	0	1	1
12	11	2	2	2	11	12
0	0	3	2	4	0	0
13	13	0	0	0	13	13
1	1	0	0	0	1	1
2	2	4	4	4	2	2

4	4	0	0	0	4	4
V0;W6SNC0;W6SIV4;W6CJ57;W6CHX3;W6CGW3;W1IB93;W0T236;W0HE66;W0G8I7;W0FF27;W0FBM8;V9W2L5;V9VYY9;V						
D4;F8RHD3;F8RHD2;F8RHD1;F8RHD0;F8RHC9;F8RHC8;F8RHC7;F8RHC6;F8RHC5;F8RHC4;F8RHC3;F8RHC2;F8RHC0;F8RHB9;						
8E988;W6SQV0;W6SNC0;W6SIV4;W6CJ57;W6CHX3;W6CGW3;W1IB93;W0T236;W0HE66;W0G8I7;W0FF27;W0FBM8;V9W						
D4;F8RHD3;F8RHD2;F8RHD1;F8RHD0;F8RHC9;F8RHC8;F8RHC7;F8RHC6;F8RHC5;F8RHC4;F8RHC3;F8RHC2;F8RHC0;F8RHB9;						
2	1	1	1	2	2	2
18.4	6.8	6.8	6.8	44536000	9117800	11324000
5	5	4	3	3	5	5
14	13	0	0	0	13	13
0	0	0	0	0	1	0
1	2	0	0	0	1	1
1	1	0	0	0	1	1
4	4	0	0	0	1	1
2	2	2	2	2	2	2
4	4	0	0	1	3	4
0	0	1	0	0	0	0
0	1	0	1	1	1	0
17	16	14	14	14	15	17
1	1	0	0	0	1	1
2	1	1	1	1	2	2
2	2	0	0	0	2	2
4	4	1	1	1	4	4
1	1	0	0	0	1	1
4	4	0	0	0	4	4
2	2	0	0	0	2	2
1	1	1	1	1	1	1
145	149	144	138	149	145	145
3	2	9	9	10	2	3
1	1	0	0	0	1	1
6	6	6	8	8	4	4
4	4	0	0	0	4	4
3	3	0	0	0	3	3
1	1	0	0	0	1	1
2	2	0	0	0	2	2
1	1	0	0	0	1	1
1	1	0	0	0	1	1
1	1	1	1	1	1	1
6	6	0	0	0	6	6
0	0	9	9	9	0	0
1	1	3	3	3	1	1
1	1	0	0	0	1	1
1	1	0	0	0	1	1
2	2	0	0	1	2	1
1	1	1	1	1	1	1
1	1	0	0	1	1	1

3	3	3	3	3	3	3
3	2	0	0	0	1	3
1	1	0	0	0	1	1
0	0	1	1	1	0	0
6	6	0	0	0	6	6
1	1	1	1	1	1	1
0	0	1	0	0	0	0
3	3	1	1	1	1	1
1	0	0	0	0	1	1
4	4	2	2	2	4	4
4	4	3	2	2	4	4
15	16	8	7	9	13	15
0	0	1	1	1	0	0
3	3	5	5	6	3	3
2	2	1	1	1	2	2
1	1	0	0	0	1	1
1	1	0	0	0	1	1
1	1	1	1	1	1	1
3	3	3	4	4	3	3
4	1	15	5	3	0	0
4	4	1	1	1	4	4
7	7	5	5	5	7	7
1	1	1	1	1	1	1
3	3	1	0	1	2	3
6	6	0	0	0	5	6
1	2	0	0	0	2	1
1	1	0	0	0	1	1
12	13	14	14	14	12	12
4	4	0	0	0	3	4
13	11	4	4	4	3	3
1	1	1	1	1	1	1
1	1	0	0	0	1	1
4	4	0	0	0	4	4
2	3	5	5	5	1	1
1	1	1	1	1	1	1
1	1	0	0	0	1	1
9	8	6	6	6	9	9
31	31	0	0	0	31	31
0	0	1	1	1	0	0
2	2	4	4	3	2	2
4	5	0	0	0	4	4
1	1	0	0	0	1	1
1	1	0	0	0	1	1
2	2	1	2	2	2	2
1	1	0	0	0	1	1

4	4	0	0	0	4	4
2	1	4	3	2	1	2
2	2	0	0	0	2	2
8	8	7	6	7	7	7
1	1	0	0	0	1	1
0	0	1	1	1	0	0
3	3	0	0	0	3	3
1	1	0	0	0	1	1
9	9	1	2	3	9	9
1	1	1	1	1	1	1
5	5	6	6	6	5	5
12	12	18	18	20	12	12
3	3	10	9	11	4	3
25	25	12	12	12	24	25
3	3	3	2	3	3	3
4	3	0	0	0	4	4
3	4	5	4	5	4	3
1	1	0	0	0	1	1
27	28	28	29	32	27	26
6	7	0	0	0	7	6
7	7	6	7	7	7	7
1	2	0	0	0	1	1
2	3	0	0	0	3	2
4	4	3	2	3	4	4
24	24	9	10	10	21	20
1	1	0	0	0	1	1
0	0	3	0	0	0	0
10	12	3	3	2	11	10
1	1	0	0	0	1	1
9	9	1	1	1	9	9
2	2	1	1	1	2	2
1	1	1	1	1	1	1
5	4	7	4	7	6	5
1	1	0	0	0	1	1
2	2	2	2	2	2	2
3	2	2	2	2	3	3
3	3	2	3	3	3	3
0	0	3	4	4	0	0
4	4	0	0	0	3	4
1	1	1	1	1	1	1
7	7	5	4	5	7	7
4	4	0	0	0	4	4
2	2	0	0	0	2	2
10	11	13	14	14	11	10
4	4	4	3	4	4	4

11	11	0	0	0	12	11
5	5	0	0	0	5	5
6	5	2	2	2	6	6
0	0	0	1	1	0	0
10	11	3	4	4	11	10
1	1	1	1	1	1	1
6	8	11	11	11	8	6
9	9	10	10	10	2	2
0	1	0	0	0	1	0
6	6	6	4	6	6	6
4	4	0	0	0	4	4
2	2	1	1	1	1	1
3	3	0	0	0	3	3
2	2	0	0	0	2	2
3	3	1	1	1	3	3
1	1	0	0	0	1	1
1	1	1	1	1	0	1
1	1	1	1	1	1	1
3	3	0	0	0	3	3
0	0	2	2	2	0	0
0	0	13	3	1	0	0
20	22	12	14	16	22	20
2	2	0	0	0	2	2
14	17	0	0	0	15	14
4	4	3	5	6	4	4
2	2	6	7	7	2	2
1	1	0	0	0	1	1
0	0	2	1	1	0	0
19	20	11	12	12	20	19
1	1	0	0	0	1	1
0	0	1	1	1	0	0
4	4	0	0	0	4	4
1	1	0	0	0	1	1
0	0	1	3	3	0	0
6	6	1	0	1	6	6
3	3	2	0	0	3	3
2	2	0	0	0	2	2
3	3	1	1	1	3	3
4	4	1	2	2	4	4
4	5	5	5	5	5	4
1	1	0	0	0	1	1
2	2	0	0	0	2	2
26	28	34	27	30	2	2
4	4	0	0	0	5	4
1	1	1	1	1	1	1

9	9	2	2	3	9	9
6	7	9	9	8	2	2
2	2	0	0	0	3	2
48	48	22	22	23	1	1
3	2	0	0	0	2	3
10	9	9	9	9	10	10
2	2	1	1	1	1	1
3	3	0	0	0	3	3
3	3	4	3	7	2	2
4	4	3	3	4	1	1
1	1	0	0	0	1	1
5	5	0	0	0	5	5
22	23	19	18	20	23	22
2	3	0	0	0	3	2
3	3	0	0	0	3	3
0	0	1	0	1	0	0
7	7	9	9	9	7	7
1	1	1	1	1	1	1
3	3	0	0	0	3	3
8	9	4	4	4	8	8
4	4	3	4	4	3	4
3	3	0	0	0	3	3
4	4	3	3	5	4	4
3	3	2	2	2	3	3
1	1	0	0	0	1	1
8	8	5	5	5	8	8
0	0	1	1	2	0	0
4	4	2	2	3	4	4
10	10	12	13	13	10	10
4	4	11	9	8	4	4
17	18	5	5	5	16	17
2	2	2	2	2	2	2
6	7	6	6	7	7	6
1	1	0	0	0	1	1
2	2	0	0	0	2	2
3	4	3	4	4	1	0
1	1	1	1	1	1	1
5	7	1	1	1	7	5
10	9	4	4	4	10	10
6	6	0	0	1	6	6
2	3	0	0	0	3	2
5	5	0	1	1	5	5
3	3	1	2	2	2	2
5	5	9	10	10	5	5
1	1	0	0	0	1	1

2	2	2	2	2	2	2
1	1	1	1	1	1	1
3	3	0	0	0	3	3
3	3	2	2	2	3	3
3	3	3	2	2	3	3
2	2	20	9	3	0	0
4	4	1	1	1	3	3
8	8	11	12	13	7	7
0	1	0	0	0	0	0
3	3	0	0	0	3	3
1	1	2	2	2	1	1
8	8	10	8	13	8	8
8	7	10	8	10	8	8
23	23	14	14	14	23	23
1	0	2	2	2	1	1
9	10	2	1	1	10	9
2	2	1	1	1	2	2
1	1	0	0	0	1	1
1	1	0	0	0	1	1
2	2	0	0	0	2	2
5	5	0	0	0	5	5
8	9	8	8	8	9	8
0	1	0	0	0	2	0
2	1	1	1	1	2	2
2	2	1	2	2	2	2
42	42	27	28	33	42	42
1	1	1	1	1	1	1
6	6	0	0	0	6	6
19	19	4	3	4	19	19
2	2	2	2	2	1	1
4	4	1	1	1	3	3
5	5	1	1	1	5	5
1	1	0	0	0	1	1
3	3	1	1	3	3	3
3	3	3	2	3	3	3
1	1	0	0	0	1	1
2	2	0	0	0	2	2
2	3	9	7	8	3	2
2	2	1	1	1	2	2
1	1	2	2	2	1	1
0	0	1	1	1	0	0
12	13	7	7	7	12	12
7	7	2	3	2	4	7
0	0	1	1	1	0	0
6	6	4	4	4	6	6

3	3	2	0	1	3	3
2	2	1	1	1	2	2
11	11	11	11	11	11	11
3	3	2	2	2	3	3
10	10	10	8	10	10	10
1	1	0	0	0	1	1
9	9	6	5	6	1	1
7	8	0	0	0	8	7
2	2	11	9	11	2	2
1	1	0	0	0	1	1
6	6	6	5	6	6	6
17	15	21	16	23	14	17
2	2	0	0	0	2	2
3	3	2	1	1	3	3
2	2	1	1	1	2	2
1	1	2	2	2	1	1
1	1	1	1	1	1	1
12	11	3	3	3	12	12
24	26	33	24	27	0	0
1	1	0	0	0	1	1
7	8	1	1	1	9	7
1	1	0	0	0	1	1
1	1	0	0	0	0	1
2	2	0	0	0	2	2
7	8	1	1	2	8	7
1	1	0	0	0	1	1
5	6	3	4	4	5	5
2	2	2	1	2	2	2
6	6	5	5	5	6	6
7	7	0	0	0	7	7
5	5	0	0	0	5	5
1	1	1	1	1	1	1
1	0	0	0	0	0	1
7	7	0	0	0	7	7
5	6	2	3	3	6	5
0	0	1	1	1	1	0
16	16	15	13	17	16	16
1	1	1	3	3	1	1
3	3	1	3	3	2	3
2	2	0	0	0	2	2
9	9	8	9	9	9	9
2	2	1	0	1	2	2
8	8	5	5	6	8	8
2	2	0	0	0	2	2
19	19	15	17	17	19	19

6	6	3	2	3	6	6
15	15	0	0	0	16	15
15	16	0	0	0	16	15
20	21	0	0	0	21	20
3	4	0	0	0	4	3
1	1	0	1	1	1	1
6	7	1	2	2	8	6
5	5	1	1	1	5	5
5	6	1	1	1	6	5
8	8	8	7	7	8	8
2	2	6	6	6	2	2
18	19	15	14	16	19	18
15	15	0	0	0	15	15
2	2	2	1	1	1	1
5	4	3	4	4	5	5
1	1	0	0	1	1	1
4	4	1	1	1	4	4
19	20	21	17	21	1	1
2	2	0	0	0	2	2
8	8	0	0	0	8	8
1	1	0	0	0	1	1
0	1	0	0	0	0	0
9	10	12	12	12	10	9
20	20	10	12	12	3	3
3	3	1	0	1	3	3
4	4	4	4	5	4	4
3	3	0	0	0	3	3
1	1	0	0	0	1	1
1	1	0	0	0	1	1
1	1	2	1	2	1	1
8	8	7	6	6	9	8
4	4	0	0	0	5	4
1	1	0	0	0	1	1
3	3	0	0	0	3	3
3	2	0	0	0	3	3
6	5	0	0	0	8	6
2	2	2	2	2	2	2
1	1	0	0	0	1	1
1	1	1	1	1	1	1
1	1	0	1	1	1	1
0	0	2	0	0	0	0
3	3	3	3	3	3	3
3	3	0	0	0	2	3
18	18	2	1	2	19	18
3	4	0	0	0	4	3

15	15	7	6	7	15	15
12	12	16	15	16	0	0
10	10	2	2	2	9	10
1	1	1	1	1	1	1
0	0	1	1	1	0	0
5	7	10	5	6	2	2
2	2	0	0	0	2	2
3	3	2	2	2	3	3
2	2	0	0	0	2	2
1	2	1	2	2	2	0
13	13	0	0	0	12	13
0	0	1	1	2	0	0
3	3	1	0	2	3	3
0	0	1	0	0	0	0
6	6	2	2	2	4	4
5	5	6	7	9	5	5
1	1	0	0	0	1	1
1	1	1	1	1	1	1
0	0	0	1	1	0	0
1	1	1	2	2	1	1
2	2	0	0	0	2	2
1	1	1	1	2	1	1
2	2	1	3	3	2	2
1	1	0	0	0	1	1
5	6	7	8	7	6	5
1	1	0	0	0	1	1
3	4	3	3	3	3	3
0	0	1	1	1	0	0
6	7	4	3	4	7	6
2	2	1	0	0	2	2
0	0	3	1	3	0	0
0	0	8	8	7	0	0
3	3	0	0	0	3	3
2	2	0	0	0	2	2
7	7	2	2	2	7	7
4	4	10	11	12	4	4
3	4	0	0	0	3	3
4	4	1	1	0	4	4
3	3	0	0	0	3	3
1	1	0	0	0	1	1
3	3	0	0	0	3	3
2	1	0	0	0	2	2
8	8	0	1	1	8	8
1	1	0	0	0	1	1
1	1	0	0	0	1	1

2	2	0	0	0	2	2
1	1	2	2	2	0	1
8	9	0	0	0	8	8
0	0	1	1	1	0	0
1	1	0	0	0	1	1
2	2	3	2	3	2	2
6	6	6	6	8	2	2
2	2	3	3	3	2	2
17	18	2	2	2	17	17
4	4	0	0	0	4	4
2	3	5	5	6	3	2
4	4	1	1	0	3	4
1	2	2	2	2	2	1
1	1	0	0	0	1	1
0	1	0	0	0	1	0
2	2	1	2	2	2	2
1	1	0	0	0	1	1
8	8	6	6	6	7	8
5	5	3	4	3	5	5
1	1	0	0	0	1	1
3	4	0	0	0	4	3
1	1	1	1	1	1	1
2	1	0	0	0	1	2
15	15	0	0	0	15	15
21	19	7	6	8	20	21
5	6	1	1	1	6	5
0	0	4	4	4	0	0
9	8	2	3	3	9	9
3	2	6	6	6	4	3
5	5	9	10	10	5	5
15	17	11	8	10	16	15
1	0	1	1	1	0	1
1	1	1	1	1	1	1
13	13	0	0	0	1	1
1	2	0	0	0	3	1
0	0	2	1	2	0	0
2	2	0	0	0	1	2
1	1	1	1	1	1	1
7	7	4	4	4	3	3
16	16	7	7	8	16	16
2	2	1	1	1	2	2
2	2	0	0	0	2	2
1	1	0	0	0	1	1
0	0	1	1	1	0	0
1	1	1	1	1	1	1

2	2	1	1	1	2	2
3	3	0	0	0	3	3
0	0	0	0	1	0	0
6	5	3	4	4	4	5
22	23	8	7	8	22	21
0	0	0	0	0	1	0
3	4	0	0	0	3	3
11	11	9	9	9	1	1
4	5	4	5	4	5	4
5	5	0	0	0	5	5
6	6	0	0	0	6	6
6	6	3	4	4	6	6
8	7	1	2	2	8	8
4	4	0	0	0	4	4
0	0	2	2	1	0	0
4	4	0	0	0	4	4
1	1	0	0	0	1	1
6	6	0	0	0	6	6
2	2	0	0	0	1	1
9	9	0	0	0	9	9
35	34	0	0	0	35	35
1	1	0	0	1	1	1
4	6	4	4	4	4	4
1	1	1	1	1	1	1
17	15	0	0	0	16	17
2	2	2	2	4	2	2
0	0	1	1	1	0	0
1	1	0	0	0	1	1
1	1	0	0	0	1	1
3	3	1	1	1	3	3
0	0	1	1	1	0	0
2	2	1	1	1	2	2
2	2	0	0	0	2	2
1	1	1	1	1	1	1
2	2	0	0	0	2	2
1	1	1	0	1	1	1
21	21	2	3	3	21	21
1	1	1	1	1	1	1
2	2	2	2	2	2	2
1	1	0	0	0	1	1
5	5	2	2	2	5	5
3	3	5	4	5	3	3
0	0	1	1	1	0	0
9	9	3	2	3	9	9
5	5	0	0	1	5	5

1	1	15	4	2	0	0
1	1	3	1	1	0	0
3	3	2	2	3	3	3
11	11	10	7	9	2	1
1	1	11	1	1	0	0
12	13	14	13	14	0	0
7	8	10	10	10	8	7
20	21	29	21	23	20	17
9	9	9	10	9	0	0
5	6	3	8	5	5	5
1	1	4	6	3	1	1
3	3	2	3	3	3	3
2	3	2	5	2	2	2
2	6	3	7	5	2	2
74	73	80	80	80	74	74
1	1	0	0	0	1	1
24	27	34	25	28	14	13
16	18	12	13	13	10	9
32	32	43	42	44	31	32
12	16	30	19	20	5	4
35	36	37	36	37	35	35
30	32	26	25	27	16	15
0	0	1	1	1	0	0
1	1	1	1	1	1	1
32	31	26	25	25	31	32
46	47	46	41	45	1	0
1	2	21	7	4	0	0
14	16	19	13	13	7	5
4	1	15	5	2	0	3
6	5	2	2	2	5	6
1	1	0	1	1	1	1
1	1	1	1	1	1	1
5	6	8	5	5	1	1
12	13	5	5	6	9	9
1	1	0	0	0	1	1
2	1	15	3	2	0	0
4	6	3	3	3	4	4
10	10	7	7	8	0	1
25	27	23	23	24	1	1
1	2	19	8	5	0	0
0	0	4	0	0	0	0
2	2	2	2	2	2	2
2	2	22	8	5	1	2
5	8	3	4	4	4	4
2	2	2	2	3	0	0

6	13	5	5	5	3	2
10	10	7	6	7	9	10
0	0	3	1	0	0	0
0	1	5	2	1	0	0
7	7	0	0	0	8	7
0	0	1	0	0	0	0
1	1	15	4	3	0	0
11	12	10	10	10	10	11
9	9	0	0	0	9	9
3	3	0	0	0	3	3
1	1	0	0	0	1	1
4	4	0	0	1	4	4
1	1	1	1	1	1	1
2	3	3	1	2	2	2
4	4	5	5	5	4	4
2	2	0	0	0	2	2
3	3	2	2	2	3	3
0	0	2	3	4	0	0
2	2	2	2	2	2	2
0	1	2	1	1	1	0
5	4	0	0	0	4	5
3	3	3	3	3	4	3
3	2	3	2	2	3	3
5	6	0	0	0	5	5
15	16	19	18	19	14	15
3	3	4	6	7	3	3
1	1	0	0	0	1	1
4	5	1	1	1	5	4
0	0	1	0	0	0	0
1	1	1	2	2	1	1
2	3	0	0	0	3	2
7	6	1	1	1	3	4
5	6	8	8	8	6	5
12	13	15	15	16	1	1
7	7	3	3	4	7	7
3	3	2	2	3	3	3
1	1	1	1	1	1	1
5	5	1	1	2	5	5
2	2	2	2	2	2	2
1	1	0	0	0	1	1
4	4	2	2	2	4	4
1	1	1	0	1	1	1
5	5	2	2	2	5	5
2	2	0	0	0	2	2
1	1	1	1	1	1	1

7	7	0	0	0	7	7
5	5	0	0	0	1	1
1	1	1	1	1	0	0
9	9	8	7	9	9	9
9	9	1	2	2	9	9
0	0	1	1	1	0	0
2	2	0	0	0	2	2
17	18	2	2	2	18	17
7	7	0	0	0	7	7
2	2	2	2	2	2	2
1	1	0	0	0	1	1
18	19	7	9	9	3	2
1	1	1	1	1	1	1
1	3	0	0	0	3	1
2	1	1	1	1	2	2
0	0	0	0	1	0	0
29	27	17	17	19	27	29
8	8	6	7	7	4	4
1	1	0	0	0	1	1
11	12	2	2	2	12	11
5	6	6	4	7	6	5
1	1	0	0	0	1	1
6	6	8	9	9	6	6
6	6	1	1	1	6	6
1	1	0	0	0	1	1
1	1	0	0	0	1	1
21	21	9	10	10	19	21
4	3	0	0	0	4	4
7	7	4	4	5	1	1
3	3	0	0	0	3	3
20	20	16	16	17	19	20
1	1	3	2	2	0	0
3	3	0	0	0	2	3
11	9	1	1	1	11	11
4	4	0	0	0	4	4
3	3	5	5	5	3	3
0	1	0	0	0	1	0
2	2	1	0	2	2	2
11	11	6	7	8	10	11
9	8	7	7	7	0	1
1	1	0	0	0	1	1
7	7	4	4	4	7	7
2	2	0	1	1	1	2
4	4	10	9	11	4	4
29	28	25	24	27	20	21

1	1	1	0	0	1	1
1	1	0	0	0	1	1
1	1	1	1	1	1	1
4	4	0	0	0	0	1
6	5	0	0	0	6	6
44	44	38	38	40	42	41
2	2	0	0	0	2	2
10	10	2	4	3	9	10
4	4	5	5	5	4	4
6	5	6	5	6	2	2
21	22	1	0	0	2	2
10	9	8	8	9	9	10
0	0	1	1	1	0	0
4	4	1	1	1	4	4
6	5	6	5	6	3	3
19	20	0	0	0	19	19
5	5	4	4	4	5	5
1	1	0	0	0	1	1
2	3	0	0	0	2	2
1	1	1	1	1	1	1
1	1	0	1	1	1	1
0	0	4	4	4	0	0
0	0	1	1	1	0	0
9	9	6	6	6	7	8
11	11	4	4	4	11	11
0	0	1	1	1	0	0
2	2	1	1	2	1	1
7	7	2	2	2	7	7
1	1	0	0	0	1	1
2	2	1	1	1	2	2
0	0	1	0	0	0	0
8	9	3	3	3	9	8
32	32	28	27	32	32	32
3	3	2	2	2	3	3
12	12	13	12	13	12	12
2	3	2	2	2	2	2
2	2	0	0	0	2	2
2	2	1	1	1	2	2
7	6	5	5	7	7	7
11	11	8	8	8	11	11
9	8	3	3	3	4	5
2	2	0	0	0	2	2
6	7	0	0	0	6	6
5	4	4	4	4	5	5
4	4	2	2	2	4	4

1	1	0	0	0	1	1
0	0	1	1	1	0	0
2	2	1	1	1	2	2
3	3	0	0	0	3	3
1	1	0	0	0	1	1
2	2	0	0	0	2	2
2	2	2	2	2	2	2
33	34	10	9	10	35	33
2	2	0	0	0	2	2
1	1	1	0	1	1	1
1	1	1	1	1	1	1
1	0	0	0	0	1	1
7	7	2	2	2	7	7
5	5	0	0	0	5	5
24	24	0	1	2	24	24
1	1	0	0	0	1	1
9	9	2	2	3	8	8
2	2	0	0	0	2	2
15	15	2	3	3	15	15
13	15	10	10	10	15	13
7	7	1	1	1	7	7
7	7	0	0	0	7	7
2	3	0	1	1	2	2
11	12	2	2	2	11	11
1	1	0	0	0	1	1
7	7	9	9	9	7	7
2	2	0	0	0	2	2
7	8	2	1	3	8	7
15	16	15	13	13	15	15
0	0	2	2	2	0	0
3	3	2	2	2	2	3
9	9	10	8	12	8	9
18	18	1	0	1	18	18
3	3	5	5	5	3	3
0	1	0	0	0	1	0
13	14	11	10	12	8	9
12	12	5	7	7	12	12
3	4	0	0	0	5	3
9	9	0	0	0	9	9
3	3	1	2	2	2	3
1	1	2	2	2	2	1
4	4	2	1	2	4	4
6	7	6	7	7	7	6
3	3	1	1	1	3	3
5	4	7	9	9	5	5

2	2	4	3	3	2	2
10	12	6	8	9	11	10
2	3	3	3	2	3	2
12	13	3	4	4	12	12
15	14	1	1	1	15	15
4	4	5	5	6	4	4
0	0	1	1	2	0	0
1	1	0	0	0	1	1
2	2	0	0	0	1	2
12	12	2	2	2	12	12
13	13	0	0	0	14	13
0	0	5	0	1	0	0
13	14	6	8	9	14	13
1	1	1	1	1	1	1
15	16	9	10	12	15	15
7	7	7	8	8	7	7
0	0	1	0	1	0	0
1	1	0	0	0	1	1
0	0	1	1	1	0	0
3	3	0	0	0	3	3
1	1	0	0	0	1	1
4	4	0	0	0	4	4
8	8	3	3	3	8	8
8	8	5	6	5	7	8
2	2	0	0	0	1	2
47	47	36	35	36	47	47
3	3	3	3	3	3	3
18	19	12	11	12	1	1
6	6	6	5	6	6	6
5	6	2	2	2	6	5
12	12	1	1	0	12	12
8	9	0	0	0	8	8
0	0	1	1	1	0	0
14	13	11	11	12	13	14
5	5	3	3	3	6	5
11	11	12	12	12	11	11
22	23	15	14	16	6	6
5	6	0	0	0	6	5
1	1	0	0	0	1	1
5	5	2	2	2	5	5
24	25	3	4	6	23	24
7	7	7	7	7	6	7
8	8	19	15	18	7	8
4	4	1	1	2	2	2
0	0	1	1	2	0	0

1	1	0	0	0	1	1
40	40	11	9	9	40	40
37	37	15	14	16	35	35
40	41	18	17	17	41	40
2	2	0	0	0	2	2
11	11	9	7	9	4	4
1	1	2	0	1	1	1
5	5	2	2	2	5	5
9	9	5	6	6	9	8
21	21	21	19	19	20	21
46	44	50	42	42	45	46
10	9	9	9	8	8	9
9	9	10	11	11	9	9
6	6	0	0	0	6	6
2	2	1	1	1	2	2
2	2	2	2	2	2	2
1	1	0	0	0	1	1
12	12	6	6	6	12	12
9	9	0	0	0	9	9
4	5	3	2	3	4	4
31	32	2	1	1	31	31
3	3	0	0	0	3	3
30	30	14	13	15	27	28
21	21	10	9	9	21	21
1	1	1	0	1	1	1
14	14	7	6	7	14	14
5	4	0	0	0	4	5
4	4	5	3	4	4	4
4	4	1	1	1	4	4
0	0	4	3	2	0	0
10	10	4	5	6	9	10
6	6	4	4	4	6	6
5	5	3	3	3	5	5
8	8	5	4	5	8	8
9	9	9	7	9	9	9
0	0	1	1	1	0	0
10	11	13	13	15	11	10
11	12	8	9	10	11	11
5	5	0	0	0	5	5
6	6	2	1	2	6	6
0	0	1	1	0	0	0
9	9	11	13	13	9	9
5	6	4	5	5	6	5
0	0	2	2	2	0	0
3	2	0	0	0	3	3

18	18	0	0	0	19	18
1	1	4	2	2	1	1
0	0	3	4	4	0	0
8	9	0	0	0	8	8
13	13	0	0	0	10	10
8	8	4	2	4	8	8
12	12	4	4	5	12	12
9	9	12	12	12	9	9
47	47	47	42	46	46	47
4	4	0	0	0	5	4
19	19	9	10	10	18	19
4	4	5	6	6	4	4
12	12	9	11	11	11	12
8	8	11	8	11	8	8
13	13	8	8	9	13	13
4	4	2	2	2	4	4
6	5	5	6	6	6	6
2	2	0	0	0	2	2
1	1	0	0	0	1	1
27	28	26	26	27	28	27
7	7	1	1	1	6	7
1	2	1	1	1	2	1
12	13	0	0	0	13	12
14	15	7	8	9	15	14
10	9	10	10	12	9	10
3	3	2	2	2	3	3
3	3	2	2	2	3	3
3	3	0	0	0	3	3
2	2	0	0	0	2	2
2	2	0	0	0	1	1
3	4	1	0	1	4	3
7	7	0	0	0	7	7
2	3	0	0	0	2	2
3	3	4	4	4	3	3
3	3	5	5	5	1	1
1	1	1	1	1	1	1
8	8	1	1	2	8	8
12	12	8	10	10	12	12
23	22	28	29	30	23	23
11	11	1	2	2	11	11
0	1	0	0	0	1	0
4	5	0	0	0	5	4
31	28	0	0	0	30	31
4	5	5	5	6	3	3
13	13	17	16	17	12	13

43	43	15	16	19	42	43
1	1	2	2	2	1	1
20	18	0	0	0	20	20
8	8	0	0	0	8	8
1	1	0	0	0	1	1
2	2	4	3	3	2	2
1	1	0	0	0	1	1
1	1	1	1	1	1	1
9	9	0	0	0	10	9
2	2	3	3	3	2	2
29	31	29	29	31	30	29
10	12	11	11	12	6	4
5	5	3	3	5	5	5
1	1	0	0	0	1	1
2	2	2	2	1	1	1
0	0	2	1	1	0	0
1	1	0	0	0	1	1
17	16	16	16	16	1	1
4	4	3	3	3	4	4
8	7	3	2	2	8	8
10	10	3	2	2	10	10
6	6	5	4	5	2	2
8	8	7	7	7	8	8
8	8	2	2	2	8	8
9	9	2	4	3	8	9
1	1	1	1	1	1	1
10	10	7	6	6	9	10
0	0	2	1	2	0	0
2	1	2	1	2	1	2
3	3	3	3	3	3	3
2	2	3	3	2	2	2
11	12	6	7	7	12	11
5	5	5	5	5	4	5
9	8	3	3	3	9	9
3	3	0	0	0	2	3
1	0	0	0	0	1	1
1	1	0	1	1	1	1
6	6	6	6	6	6	6
8	8	7	6	6	8	8
9	9	6	5	6	9	9
7	7	4	5	5	1	1
8	8	6	7	7	8	8
3	3	0	0	0	3	3
4	4	4	5	5	4	4
1	1	2	1	1	1	1

17	17	16	16	17	18	17
6	6	1	1	1	6	6
18	19	12	12	11	20	18
21	21	10	12	12	20	21
23	24	15	15	16	24	23
2	2	0	0	0	2	2
6	6	2	2	2	7	6
6	6	1	1	2	6	6
2	2	0	0	0	2	2
120	122	69	69	81	120	120
0	0	1	1	1	0	0
2	2	2	2	2	2	2
0	0	4	4	3	0	0
19	20	18	18	19	19	19
5	7	10	10	9	7	5
6	7	0	0	0	6	6
5	5	1	1	1	4	5
2	2	2	1	1	2	2
7	7	6	5	5	7	7
6	7	5	5	4	7	6
35	36	20	21	23	24	25
29	27	2	2	2	27	29
1	1	2	2	2	1	1
10	10	7	7	6	3	3
9	9	5	4	5	9	9
4	4	2	2	3	4	4
16	17	4	5	5	12	12
13	13	15	14	15	13	13
0	0	2	1	1	0	0
7	7	9	6	8	6	7
2	2	3	2	2	1	2
9	9	5	5	5	9	9
12	10	1	1	1	11	12
14	13	6	6	7	14	14
22	22	0	0	0	20	22
2	2	2	3	3	2	2
11	11	8	8	8	5	5
4	4	0	0	0	4	4
6	6	5	5	5	6	6
14	13	0	0	0	14	14
11	10	14	16	16	11	11
13	14	16	16	17	14	13
17	18	8	7	8	6	6
7	7	0	0	0	6	7
10	10	6	7	7	9	10

6	6	7	6	6	4	4
0	0	5	4	5	0	0
6	5	0	0	0	6	6
11	11	1	2	1	11	11
18	19	11	11	11	2	2
5	7	13	12	12	5	5
6	5	0	0	0	5	6
2	2	3	2	3	2	2
3	2	4	3	4	3	3
2	2	0	0	0	2	2
22	22	16	18	18	22	22
2	1	8	3	2	0	0
21	23	13	12	15	22	21
1	1	0	0	0	1	1
7	6	11	11	11	5	7
2	2	0	0	0	2	2
25	26	4	4	5	25	25
20	20	23	23	24	21	20
7	7	5	5	5	4	7
1	2	0	1	1	1	1
47	47	0	0	0	46	47
17	16	13	11	14	16	17
3	4	0	0	0	4	3
19	18	5	4	6	19	19
128	131	70	73	77	1	1
2	2	2	1	2	2	2
1	1	0	0	0	1	1
1	1	1	1	1	1	1
5	5	0	0	0	5	5
17	15	1	0	0	16	17
12	11	11	11	10	12	12
1	1	1	1	1	1	1
14	14	17	18	17	14	14
9	9	4	7	6	5	5
5	5	4	5	5	5	5
7	6	2	2	2	6	7
5	6	0	0	0	6	5
9	9	11	11	11	3	3
36	38	0	0	0	39	36
5	4	6	8	8	4	5
0	0	2	0	0	0	0
11	11	6	6	6	2	2
0	1	4	4	4	1	0
0	0	1	0	0	0	0
1	1	0	0	0	1	1

2	2	2	2	3	2	2
1	1	0	0	0	1	1
6	6	1	1	1	6	6
3	3	0	0	0	3	3
6	6	3	3	4	6	6
16	15	8	9	8	8	8
29	30	29	29	31	1	1
22	22	9	10	10	1	1
7	7	8	9	10	7	7
13	13	8	7	9	13	13
5	7	0	0	0	6	5
0	0	1	0	1	0	0
12	11	0	0	0	1	1
5	5	0	0	0	5	5
3	3	3	3	4	3	3
3	3	0	0	0	3	3
4	3	0	0	0	3	4
0	0	3	5	5	0	0
10	10	0	0	0	10	10
12	13	1	2	3	13	12
7	8	6	6	7	8	7
11	11	2	2	2	11	11
3	3	0	0	0	3	3
0	0	1	1	1	0	0
3	3	0	0	0	3	3
2	2	0	0	0	2	2
8	11	4	6	5	10	8
1	2	2	2	2	2	1
0	0	0	0	1	0	0
0	0	2	2	2	0	0
3	3	3	3	3	3	3
7	6	1	2	2	7	7
6	6	0	0	0	6	6
49	50	17	18	17	49	49
4	4	7	8	7	4	4
6	8	0	0	0	8	6
1	1	0	0	0	1	1
18	19	0	0	1	17	18
13	13	0	0	0	13	13
2	2	3	3	3	2	2
8	8	0	0	0	6	8
6	5	0	0	0	5	6
3	3	0	0	0	3	3
31	33	26	24	25	32	31
8	8	1	1	1	8	8

25	27	1	1	1	28	25
3	3	0	0	0	3	3
3	5	1	2	2	4	3
0	0	1	1	1	0	0
5	5	0	0	0	5	5
8	8	0	0	0	9	8
5	6	0	0	0	6	5
13	14	8	8	8	14	13
6	6	0	0	0	6	6
1	1	0	0	0	1	1
5	5	0	0	0	4	5
1	1	0	0	0	1	1
7	7	1	1	1	7	7
2	2	2	2	2	2	2
1	1	0	0	0	0	1
0	0	1	1	1	0	0
15	15	16	14	16	1	1
2	1	0	0	0	2	2
0	0	4	3	4	0	0
2	2	1	1	1	2	2
12	10	0	0	0	12	12
11	11	1	1	1	11	11
1	3	1	0	0	3	1
17	17	0	0	0	18	17
6	6	2	2	2	6	6
1	1	2	2	3	1	1
1	1	0	0	0	1	1
1	1	0	0	0	1	1
0	0	3	1	0	0	0
2	2	0	0	0	1	1
2	2	0	0	0	2	2
3	3	2	2	2	3	3
17	17	3	3	3	17	17
4	4	6	7	7	4	4
5	7	4	4	4	5	5
4	4	6	6	6	4	4
4	4	0	0	0	4	4
0	0	2	2	2	0	0
10	11	4	4	4	11	10
23	24	15	15	16	1	1
4	4	0	0	0	4	4
25	26	25	22	26	26	25
2	2	0	0	0	2	2
6	7	3	2	3	5	6
2	2	0	1	1	2	2

3	3	0	0	0	3	3
11	12	0	0	0	10	11
0	0	3	0	0	0	0
7	7	1	0	1	6	7
6	6	5	4	5	6	6
5	4	1	2	2	5	5
2	2	0	0	0	2	2
3	3	0	0	0	3	3
5	5	1	1	1	5	5
3	2	1	1	1	2	2
3	1	1	1	1	3	3
2	2	2	2	2	2	2
1	1	4	4	4	1	1
1	1	0	0	0	1	1
4	4	1	1	1	4	4
1	1	2	2	2	1	1
4	5	4	4	5	5	4
4	3	0	0	0	3	4
7	7	6	5	6	7	7
25	25	0	0	0	22	22
4	4	4	3	5	4	4
1	1	0	0	0	1	1
0	0	1	1	1	0	0
27	28	0	0	0	28	27
23	23	0	0	0	17	17
4	4	6	5	6	4	4
6	7	7	7	7	6	6
1	1	1	0	1	1	1
2	2	0	0	0	2	2
2	3	1	1	2	3	2
7	7	0	0	0	7	7
4	4	0	0	0	4	4
16	16	18	17	19	16	16
5	5	2	2	3	5	5
75	75	9	11	10	75	75
1	1	2	2	2	1	1
4	4	4	4	4	4	4
23	21	11	14	15	20	23
9	10	2	2	2	9	9
3	3	5	6	4	4	3
32	32	36	34	36	31	32
3	5	0	0	0	3	3
1	1	1	1	1	1	1
1	1	0	0	0	1	1
1	1	1	1	1	1	1

2	2	0	0	0	2	2
3	4	0	0	0	4	3
4	3	0	0	0	4	4
6	8	12	11	13	7	6
0	0	3	2	3	0	0
5	5	3	4	4	6	5
11	11	14	13	13	11	11
2	2	3	3	3	2	2
6	6	0	0	0	6	6
8	8	0	0	0	8	8
1	1	0	0	0	1	1
1	1	1	1	1	1	1
5	6	3	2	3	6	5
2	2	0	0	0	2	2
3	3	1	1	2	4	3
14	14	0	0	0	15	14
2	2	0	0	0	2	2
23	23	12	12	14	20	23
1	1	3	3	3	0	0
2	2	0	0	0	2	2
21	20	1	1	1	22	21
3	3	0	0	0	3	3
5	5	0	1	1	5	5
6	8	1	1	1	6	6
14	16	0	0	0	14	14
22	21	2	2	3	22	22
4	4	0	0	0	4	4
3	3	2	2	2	1	1
3	3	5	5	5	3	3
2	2	2	3	3	2	2
34	30	23	24	22	32	34
10	9	4	7	6	10	10
9	9	9	9	11	9	9
5	5	7	7	7	5	5
24	25	5	6	7	23	24
1	1	1	0	1	1	1
8	8	10	10	10	8	8
15	16	5	5	5	5	5
7	7	2	2	2	8	7
9	10	0	0	0	8	9
5	5	0	0	0	6	5
5	5	5	5	5	6	5
4	4	1	1	1	4	4
4	3	1	2	2	3	4
5	5	5	5	5	0	0

16	18	0	0	0	18	16
3	3	6	6	6	3	3
2	2	0	0	0	2	2
2	2	0	0	0	2	2
4	4	1	1	1	1	2
25	25	0	0	0	25	25
9	11	0	0	0	10	9
3	3	1	1	1	3	3
3	3	4	5	4	2	3
7	10	2	1	2	7	7
0	1	1	1	1	1	0
6	6	1	1	1	7	6
2	2	0	0	0	2	2
5	5	5	5	5	5	5
9	9	2	2	4	8	9
3	3	0	0	0	3	3
2	2	1	0	1	2	2
41	43	0	0	1	43	41
2	2	2	2	1	2	2
2	2	2	1	2	2	2
10	10	21	21	21	10	10
3	3	2	3	2	3	3
1	1	0	0	0	1	1
1	1	0	0	0	1	1
7	8	0	0	0	8	7
36	38	2	2	2	39	36
5	6	7	6	7	6	5
1	1	2	3	2	1	1
21	21	7	6	7	21	21
3	3	0	0	0	3	3
5	6	0	0	0	6	5
11	10	0	0	0	1	1
2	2	3	2	3	2	2
3	4	2	2	3	3	3
1	1	0	0	0	1	1
2	2	2	0	2	2	2
1	1	0	0	0	2	1
27	28	31	33	33	28	27
12	15	0	0	0	15	12
1	1	0	0	0	1	1
2	2	0	0	0	2	2
29	29	0	0	0	27	29
3	3	6	7	8	2	3
2	2	1	1	1	1	2
1	1	2	2	3	1	1

3	3	0	0	0	3	3
6	6	3	4	3	5	6
0	1	1	0	1	1	0
0	0	5	5	4	0	0
3	3	0	0	0	3	3
1	1	0	0	0	1	1
5	5	0	0	0	5	5
6	6	0	0	0	6	6
17	18	13	14	15	17	16
2	1	0	0	0	2	2
2	2	1	1	1	2	2
1	1	2	2	2	1	1
12	12	0	1	1	12	12
5	4	3	4	3	5	5
4	4	0	0	0	4	4
2	1	0	0	0	2	2
4	4	3	4	4	4	4
2	2	0	0	0	2	2
6	5	6	6	6	5	6
0	0	1	0	0	0	0
2	2	0	0	0	2	2
0	0	1	1	1	0	0
13	12	4	4	4	12	13
19	20	0	1	1	19	19
6	6	0	0	0	6	6
4	4	3	3	2	4	4
8	7	0	0	0	6	8
12	12	0	0	0	11	12
14	14	10	8	10	14	14
27	28	25	21	27	27	27
18	18	9	10	12	20	18
1	1	0	0	0	0	1
8	8	0	0	0	8	8
3	4	0	0	0	4	3
10	11	11	11	15	11	10
10	12	7	6	8	11	10
18	19	14	15	16	17	18
18	18	10	10	10	19	18
26	27	0	0	0	27	26
15	15	13	14	15	15	15
1	1	0	0	0	1	1
4	4	7	9	10	4	4
2	2	1	1	1	2	2
2	2	1	2	2	2	2
3	3	2	2	2	3	3

2	2	3	3	3	2	2
6	5	9	9	10	6	6
22	24	0	0	0	22	22
3	3	2	2	2	3	3
17	17	5	5	5	18	17
20	21	8	9	10	18	20
5	4	3	3	1	6	5
6	5	2	2	2	4	6
0	1	1	0	0	0	0
1	1	1	1	1	1	1
0	0	0	0	1	0	0
1	1	0	0	0	1	1
0	0	1	1	1	0	0
1	1	1	1	1	1	1
1	1	1	1	0	1	1
0	0	1	1	1	0	0
0	1	0	0	0	0	0
1	1	1	1	0	1	1
0	0	0	1	0	0	0
1	1	0	0	0	1	1
0	0	0	0	0	1	0
0	0	1	1	0	0	0
0	1	0	0	0	1	0
1	1	1	1	1	1	1
1	1	1	1	1	1	1
1	1	1	1	1	1	1
3	3	2	2	2	1	1
8	9	11	11	13	7	8
5	6	4	5	5	6	5

Razor + uniq	Razor + uniq	Razor + uniq	Razor + uniq	Unique pepti	Unique pepti	Unique pepti
4	2	3	3	4	4	4
3	1	1	2	3	3	3
2	1	1	1	2	2	2
5	10	10	10	5	5	5
5	5	4	5	5	5	5
2	1	1	1	2	2	2
1	0	0	0	1	1	1
6	5	4	5	6	5	6
5	6	6	7	5	5	5
2	0	0	0	2	1	2
2	3	4	4	2	2	2
1	2	2	2	1	1	1
4	4	3	4	4	4	4
1	1	1	1	1	1	1
3	0	0	0	3	3	3
1	0	0	0	1	0	1
9	4	5	8	9	7	9
0	0	0	0	1	0	0
21	5	5	6	22	21	21
6	4	3	3	5	6	6
5	5	6	6	5	6	5
1	1	1	1	1	1	1
16	18	15	18	16	16	16
2	0	0	0	2	2	2
8	0	0	0	9	8	8
1	1	1	1	1	1	1
8	2	2	2	8	7	8
1	0	0	0	1	1	1
3	1	2	2	4	5	3
8	0	0	0	8	8	8
9	1	0	0	9	8	9
0	0	0	0	1	1	0
1	0	0	0	1	0	1
2	2	2	2	2	2	2
3	2	2	2	2	3	3
1	0	0	0	1	1	1
5	4	5	5	4	4	4
2	0	0	0	2	2	2
1	0	0	0	1	1	1
4	3	2	4	4	4	4
1	0	0	0	1	1	1
16	0	0	0	15	16	16
1	1	0	2	1	1	1
2	4	4	5	2	2	2

7	2	3	3	7	7	7
1	1	1	1	1	1	1
2	3	3	3	2	2	2
2	0	0	0	2	2	2
8	0	0	0	8	8	8
2	0	0	0	2	2	2
1	0	0	0	1	1	1
1	3	3	3	1	1	1
4	5	4	5	4	5	4
4	0	0	0	4	4	4
6	1	1	1	6	6	6
2	1	0	1	2	1	2
7	5	5	5	7	7	7
1	0	0	0	1	1	1
0	10	0	1	0	0	0
2	0	0	1	2	2	2
4	5	7	6	4	2	4
3	3	2	2	4	4	3
1	0	0	0	1	1	1
8	6	6	6	8	8	8
16	0	0	0	13	13	13
3	3	1	2	2	2	2
1	1	0	1	1	1	1
1	0	0	0	1	1	1
4	0	0	0	4	4	4
21	23	23	23	18	19	21
2	0	0	0	2	2	2
9	5	6	5	8	8	9
5	3	3	3	5	4	5
1	0	0	0	1	1	1
2	0	0	0	2	2	2
2	0	0	0	2	1	2
9	0	0	0	9	9	9
6	4	4	4	6	6	6
3	1	1	1	5	4	3
1	0	0	0	1	1	1
5	3	3	3	5	5	5
3	5	4	5	3	3	3
2	0	0	0	2	2	2
7	4	4	4	7	7	7
2	0	0	0	2	2	2
5	1	1	1	5	5	5
6	1	1	0	6	6	6
2	0	0	0	2	2	2
2	1	0	1	2	2	2

0	2	2	2	0	0	0
3	3	3	4	3	3	3
2	1	1	2	2	2	2
1	1	2	2	1	1	1
7	0	0	0	7	8	7
1	1	1	1	1	1	1
16	18	17	18	16	16	16
1	0	0	0	1	0	1
3	4	4	3	3	3	3
5	0	0	0	5	5	5
1	0	0	0	2	2	1
12	3	3	3	13	12	12
1	1	1	1	2	2	1
2	5	4	4	3	2	2
3	4	4	4	3	3	3
27	0	0	0	27	27	27
2	0	0	0	2	2	2
12	15	14	18	10	12	12
6	5	5	6	6	5	6
0	2	1	1	0	0	0
5	0	0	0	5	4	5
6	2	1	2	6	6	6
8	3	4	4	6	7	8
1	1	1	1	2	1	1
4	0	0	0	4	4	4
2	0	0	0	2	2	2
2	1	2	2	2	2	2
10	5	5	6	12	11	10
16	8	8	8	13	13	13
13	12	12	13	11	11	10
3	0	0	0	3	3	3
1	1	1	1	2	3	1
2	0	0	0	2	1	2
1	0	0	0	1	1	1
1	0	0	0	1	2	1
2	0	0	0	2	2	2
1	0	0	0	1	0	1
1	1	1	1	1	1	1
132	70	72	77	2	2	2
0	1	1	2	0	0	0
4	0	0	0	4	4	4
1	1	1	1	1	1	1
1	0	0	0	1	1	1
19	14	13	14	19	18	19
0	1	1	0	1	1	0

1	1	1	1	1	1	1
14	4	4	4	12	13	14
6	5	5	5	5	6	6
1	2	3	3	1	2	1
2	0	0	0	2	2	2
1	1	1	1	1	1	1
1	0	0	0	2	1	1
8	0	0	0	8	8	8
1	0	0	0	1	1	1
1	2	2	2	1	1	1
4	0	0	0	4	4	4
3	0	0	0	3	3	3
3	1	1	1	3	3	3
3	0	0	0	3	3	3
1	3	3	3	1	1	1
1	0	0	0	1	1	1
13	16	14	17	0	0	0
2	1	0	1	2	2	2
23	20	17	21	3	2	3
26	5	5	6	23	26	26
3	1	1	1	3	3	3
1	1	0	1	1	1	1
6	3	3	4	5	5	6
11	8	8	10	10	9	11
1	0	0	0	1	1	1
3	0	0	0	4	3	3
16	0	0	0	13	14	14
1	0	0	1	1	1	1
7	4	3	4	3	4	3
1	1	1	1	1	1	1
3	0	0	0	2	2	3
2	0	0	0	2	2	2
2	1	1	1	2	2	2
6	0	0	0	6	6	6
0	2	2	2	1	1	0
2	1	0	1	2	2	2
1	0	0	0	1	0	1
7	2	1	3	9	6	7
8	0	0	0	8	8	8
3	1	2	2	3	3	3
2	1	2	2	1	2	2
2	1	1	1	0	2	2
4	2	3	3	4	4	4
0	3	2	2	0	0	0
11	10	9	13	11	11	11

7	9	9	8	5	5	5
6	11	13	13	6	5	6
5	6	6	7	4	4	4
2	0	0	0	2	2	2
0	3	0	0	0	0	0
4	0	0	0	5	5	4
10	4	5	5	11	10	10
10	10	10	10	9	10	10
2	1	1	1	2	2	2
5	0	0	0	6	5	5
8	1	2	2	7	8	8
2	0	0	0	2	2	2
5	0	0	0	5	5	5
1	0	0	0	1	1	1
16	1	1	1	17	16	16
1	0	0	0	1	1	1
10	1	2	2	9	10	10
2	0	0	0	2	2	2
2	0	0	0	2	2	2
22	8	7	8	24	25	22
9	1	0	0	9	8	9
19	16	16	18	17	17	19
2	0	0	0	2	2	2
22	23	22	25	23	23	22
2	0	0	0	2	2	2
8	9	10	10	8	8	8
0	2	2	2	0	0	0
5	4	4	4	2	2	2
6	8	10	11	6	5	6
11	4	5	5	9	10	10
11	13	11	12	10	10	11
5	6	6	5	4	4	4
2	2	2	2	3	3	2
14	10	12	11	7	7	6
0	1	1	1	0	0	0
1	1	1	1	1	1	1
2	1	1	1	2	2	2
12	10	10	10	12	11	12
2	4	4	3	2	2	2
4	4	4	4	3	3	4
9	3	4	6	9	8	9
0	1	1	1	0	0	0
5	5	3	5	5	5	5
1	1	1	1	1	1	1
11	6	6	6	11	12	11

6	6	5	6	2	2	2
0	1	1	0	2	2	0
1	1	1	1	1	1	1
5	0	0	1	5	5	5
10	0	0	0	10	10	10
6	1	1	0	6	6	6
2	4	4	4	2	2	2
1	0	1	1	1	1	1
3	0	0	0	1	1	2
0	4	4	4	0	0	0
6	1	0	1	7	7	6
2	0	0	0	1	1	2
0	2	2	2	0	0	0
4	0	0	0	4	4	4
1	0	0	0	0	1	0
8	0	0	0	7	8	8
3	3	3	3	2	2	2
4	4	3	4	4	5	4
2	0	0	0	2	2	2
37	2	1	2	37	35	37
6	0	0	0	6	6	6
11	1	1	1	10	11	11
1	1	1	1	0	0	1
1	0	0	0	1	1	1
1	0	0	0	1	1	1
1	0	0	0	1	1	1
1	2	2	2	1	1	1
1	0	0	0	1	1	1
7	0	0	0	7	7	7
2	0	0	0	1	1	2
1	0	0	0	1	1	1
2	0	0	0	2	2	2
0	5	5	5	0	0	0
10	9	7	8	9	9	9
2	1	0	2	2	2	2
1	2	1	2	1	1	1
2	1	1	1	1	1	2
2	0	0	0	2	1	2
3	0	0	0	3	2	3
1	0	0	0	1	1	1
11	2	2	2	11	12	11
0	3	2	4	0	0	0
13	0	0	0	13	13	13
1	0	0	0	1	1	1
2	4	4	4	2	2	2

4	0	0	0	4	4	4
;W0FBM8;V9W2L5;V9VYY9;V6CJI7;V5V1E3;V5NE49;V5L1W2;V5L197;U6EV21;U6ER51;U6BZR6;U6BZQ8;U5XMI1;U4QB70;U						
C3;F8RHC2;F8RHC0;F8RHB9;F8RHB8;F8R8M1;F8R8L8;F8R8L4;F8R8L1;F8R8K9;F8R8K7;F8R8K0;F8R8J6;F8R8J2;F8R8I9;F8R8						
G8I7;W0FF27;W0FBM8;V9W2L5;V9VYY9;V6CJI7;V5V1E3;V5NE49;V5L1W2;V5L197;U6EV21;U6ER51;U6BZR6;U6BZQ8;U5XM						
C3;F8RHC2;F8RHC0;F8RHB9;F8RHB8;F8R8M1;F8R8L8;F8R8L4;F8R8L1;F8R8K9;F8R8K7;F8R8K0;F8R8J6;F8R8J2;F8R8I9;F8R8						
1	1	1	2	2	2	1
11347000	4204700	2752300	5790800	3181200	651270	808870
5	4	3	3	5	5	5
12	0	0	0	2	2	2
0	0	0	0	1	0	0
2	0	0	0	1	1	2
1	0	0	0	1	1	1
1	0	0	0	1	1	1
2	2	2	2	2	2	2
4	0	0	1	3	4	4
0	1	0	0	0	0	0
1	0	1	1	1	0	1
16	14	14	14	14	16	15
1	0	0	0	1	1	1
1	1	1	1	2	2	1
2	0	0	0	2	2	2
4	1	1	1	4	4	4
1	0	0	0	1	1	1
4	0	0	0	4	4	4
2	0	0	0	2	2	2
1	1	1	1	1	1	1
149	144	138	149	131	131	135
2	9	9	10	2	3	2
1	0	0	0	1	1	1
4	4	6	6	4	4	4
4	0	0	0	4	4	4
3	0	0	0	3	3	3
1	0	0	0	1	1	1
2	0	0	0	2	2	2
1	0	0	0	1	1	1
1	0	0	0	1	1	1
1	1	1	1	1	1	1
6	0	0	0	6	6	6
0	9	9	9	0	0	0
1	3	3	3	1	1	1
1	0	0	0	1	1	1
1	0	0	0	1	1	1
1	0	0	0	2	1	1
1	1	1	1	1	1	1
1	0	0	1	1	1	1

3	3	3	3	3	3	3
2	0	0	0	1	3	2
1	0	0	0	1	1	1
0	1	1	1	0	0	0
6	0	0	0	4	4	4
1	1	1	1	1	1	1
0	1	0	0	0	0	0
1	0	0	0	0	0	0
0	0	0	0	1	1	0
4	2	2	2	4	4	4
4	3	2	2	4	4	4
16	8	7	9	13	15	16
0	1	1	1	0	0	0
3	5	5	6	3	3	3
2	1	1	1	2	2	2
1	0	0	0	1	1	1
1	0	0	0	1	1	1
1	1	1	1	1	1	1
3	3	4	4	3	3	3
0	4	1	1	0	0	0
4	1	1	1	4	4	4
7	5	5	5	7	7	7
1	1	1	1	1	1	1
3	1	0	1	2	3	3
6	0	0	0	5	6	6
2	0	0	0	2	1	2
1	0	0	0	1	1	1
13	14	14	14	10	10	11
4	0	0	0	3	4	4
3	0	0	0	3	3	3
1	1	1	1	1	1	1
1	0	0	0	1	1	1
4	0	0	0	4	4	4
1	3	3	3	1	1	1
1	1	1	1	1	1	1
1	0	0	0	1	1	1
8	6	6	6	9	9	8
31	0	0	0	28	28	28
0	1	1	1	0	0	0
2	4	4	3	2	2	2
5	0	0	0	4	4	5
1	0	0	0	1	1	1
1	0	0	0	1	1	1
2	1	2	2	2	2	2
1	0	0	0	1	1	1

4	0	0	0	4	4	4
1	4	3	2	1	2	1
2	0	0	0	2	2	2
7	6	6	6	7	7	7
1	0	0	0	1	1	1
0	1	1	1	0	0	0
3	0	0	0	3	3	3
1	0	0	0	1	1	1
9	1	2	3	9	9	9
1	1	1	1	1	1	1
5	6	6	6	5	5	5
12	18	18	20	12	12	12
3	10	9	11	4	3	3
25	12	12	12	18	19	19
3	3	2	3	3	3	3
3	0	0	0	4	4	3
4	5	4	5	4	3	4
1	0	0	0	1	1	1
27	28	29	31	27	26	27
7	0	0	0	7	6	7
7	6	7	7	7	7	7
2	0	0	0	1	1	2
3	0	0	0	3	2	3
4	3	2	3	4	4	4
20	8	9	9	1	1	1
1	0	0	0	1	1	1
0	3	0	0	0	0	0
12	3	3	2	10	9	11
1	0	0	0	1	1	1
9	1	1	1	9	9	9
2	1	1	1	2	2	2
1	1	1	1	1	1	1
4	7	4	7	6	5	4
1	0	0	0	1	1	1
2	2	2	2	2	2	2
2	2	2	2	3	3	2
3	2	3	3	3	3	3
0	3	4	4	0	0	0
4	0	0	0	3	4	4
1	1	1	1	1	1	1
7	5	4	5	7	7	7
4	0	0	0	4	4	4
2	0	0	0	2	2	2
11	13	14	14	11	10	11
4	4	3	4	1	1	1

11	0	0	0	12	11	11
5	0	0	0	5	5	5
5	2	2	2	6	6	5
0	0	1	1	0	0	0
11	3	4	4	11	10	11
1	1	1	1	1	1	1
8	11	11	11	8	6	8
2	2	2	2	2	2	2
1	0	0	0	1	0	1
6	6	4	6	5	5	5
4	0	0	0	4	4	4
1	0	0	0	0	0	0
3	0	0	0	3	3	3
2	0	0	0	2	2	2
3	1	1	1	3	3	3
1	0	0	0	1	1	1
1	1	1	1	0	1	1
1	1	1	1	1	1	1
3	0	0	0	3	3	3
0	2	2	2	0	0	0
0	2	0	0	0	0	0
22	12	14	16	22	20	22
2	0	0	0	2	2	2
17	0	0	0	15	14	17
4	3	5	6	4	4	4
2	6	7	7	2	2	2
1	0	0	0	1	1	1
0	2	1	1	0	0	0
20	11	12	12	20	19	20
1	0	0	0	1	1	1
0	1	1	1	0	0	0
4	0	0	0	4	4	4
1	0	0	0	1	1	1
0	1	3	3	0	0	0
6	1	0	1	6	6	6
3	2	0	0	3	3	3
2	0	0	0	2	2	2
3	1	1	1	3	3	3
4	1	2	2	4	4	4
5	5	5	5	5	4	5
1	0	0	0	1	1	1
2	0	0	0	2	2	2
2	3	2	2	1	1	1
4	0	0	0	5	4	4
1	1	1	1	1	1	1

9	2	2	3	9	9	9
2	3	3	3	2	2	2
2	0	0	0	3	2	2
1	1	1	1	1	1	1
2	0	0	0	2	3	2
9	9	9	9	10	10	9
1	0	0	0	1	1	1
3	0	0	0	3	3	3
2	3	3	6	2	2	2
1	0	1	1	1	1	1
1	0	0	0	1	1	1
5	0	0	0	5	5	5
23	19	18	20	22	21	22
3	0	0	0	3	2	3
3	0	0	0	3	3	3
0	1	0	1	0	0	0
7	9	9	9	7	7	7
1	1	1	1	1	1	1
3	0	0	0	3	3	3
9	4	4	4	8	8	9
4	3	4	4	3	4	4
3	0	0	0	3	3	3
4	3	3	5	4	4	4
3	2	2	2	3	3	3
1	0	0	0	1	1	1
8	5	5	5	8	8	8
0	1	1	2	0	0	0
4	2	2	3	4	4	4
10	12	13	13	10	10	10
4	11	9	8	4	4	4
18	5	5	5	16	17	18
2	2	2	2	2	2	2
7	6	6	7	4	3	4
1	0	0	0	1	1	1
2	0	0	0	2	2	2
1	1	1	1	1	0	1
1	1	1	1	1	1	1
7	1	1	1	7	5	7
9	4	4	4	10	10	9
6	0	0	1	6	6	6
3	0	0	0	3	2	3
5	0	1	1	5	5	5
2	0	1	1	2	2	2
5	9	10	10	5	5	5
1	0	0	0	1	1	1

2	2	2	2	2	2	2
1	1	1	1	1	1	1
3	0	0	0	2	2	2
3	2	2	2	3	3	3
3	3	2	2	3	3	3
0	2	1	0	0	0	0
3	0	0	0	3	3	3
7	10	11	12	7	7	7
1	0	0	0	0	0	1
3	0	0	0	3	3	3
1	2	2	2	1	1	1
8	10	8	13	8	8	8
7	10	8	10	8	8	7
23	14	14	14	23	23	23
0	2	2	2	1	1	0
10	2	1	1	10	9	10
2	1	1	1	2	2	2
1	0	0	0	1	1	1
1	0	0	0	1	1	1
2	0	0	0	2	2	2
5	0	0	0	5	5	5
9	8	8	8	8	7	8
1	0	0	0	2	0	1
1	1	1	1	2	2	1
2	1	2	2	2	2	2
42	27	28	33	42	42	42
1	1	1	1	1	1	1
6	0	0	0	6	6	6
19	4	3	4	19	19	19
1	1	1	1	1	1	1
3	0	0	0	3	3	3
5	1	1	1	4	4	4
1	0	0	0	1	1	1
3	1	1	3	3	3	3
3	3	2	3	3	3	3
1	0	0	0	1	1	1
2	0	0	0	2	2	2
3	9	7	8	0	0	0
2	1	1	1	2	2	2
1	2	2	2	1	1	1
0	1	1	1	0	0	0
13	7	7	7	12	12	13
7	2	3	2	4	7	7
0	1	1	1	0	0	0
6	4	4	4	6	6	6

3	2	0	1	3	3	3
2	1	1	1	2	2	2
11	11	11	11	11	11	11
3	2	2	2	3	3	3
10	10	8	10	9	9	9
1	0	0	0	1	1	1
1	1	1	1	1	1	1
8	0	0	0	8	7	8
2	11	9	11	2	2	2
1	0	0	0	1	1	1
6	6	5	6	6	6	6
15	21	16	23	14	17	15
2	0	0	0	2	2	2
3	2	1	1	3	3	3
2	1	1	1	2	2	2
1	2	2	2	1	1	1
1	1	1	1	1	1	1
11	3	3	3	12	12	11
0	2	0	0	0	0	0
1	0	0	0	1	1	1
8	1	1	1	9	7	8
1	0	0	0	1	1	1
1	0	0	0	0	1	1
2	0	0	0	2	2	2
8	1	1	2	8	7	8
1	0	0	0	1	1	1
6	3	4	4	5	5	6
2	2	1	2	2	2	2
6	5	5	5	1	1	1
7	0	0	0	7	7	7
5	0	0	0	5	5	5
1	1	1	1	1	1	1
0	0	0	0	0	1	0
7	0	0	0	7	7	7
6	2	3	3	6	5	6
0	1	1	1	1	0	0
16	15	13	17	1	1	1
1	1	3	3	1	1	1
3	1	3	3	2	3	3
2	0	0	0	2	2	2
9	8	9	9	7	7	7
2	1	0	1	2	2	2
8	5	5	6	2	2	2
2	0	0	0	2	2	2
19	15	17	17	19	19	19

6	3	2	3	6	6	6
15	0	0	0	16	15	15
16	0	0	0	15	15	15
21	0	0	0	21	20	21
4	0	0	0	4	3	4
1	0	1	1	1	1	1
7	1	2	2	8	6	7
5	1	1	1	5	5	5
6	1	1	1	6	5	6
8	8	7	7	8	8	8
2	6	6	6	2	2	2
19	15	14	16	19	18	19
15	0	0	0	15	15	15
1	0	0	0	1	1	1
4	3	4	4	5	5	4
1	0	0	1	1	1	1
4	1	1	1	4	4	4
1	1	1	1	1	1	1
2	0	0	0	2	2	2
8	0	0	0	7	7	7
1	0	0	0	1	1	1
1	0	0	0	0	0	1
10	12	12	12	10	9	10
3	2	2	2	0	0	0
3	1	0	1	3	3	3
4	4	4	5	4	4	4
3	0	0	0	3	3	3
1	0	0	0	1	1	1
1	0	0	0	1	1	1
1	2	1	2	1	1	1
8	7	6	6	9	8	8
4	0	0	0	5	4	4
1	0	0	0	1	1	1
3	0	0	0	3	3	3
2	0	0	0	3	3	2
5	0	0	0	8	6	5
2	2	2	2	2	2	2
1	0	0	0	1	1	1
1	1	1	1	1	1	1
1	0	1	1	1	1	1
0	2	0	0	0	0	0
3	3	3	3	3	3	3
3	0	0	0	2	3	3
18	2	1	2	19	18	18
4	0	0	0	4	3	4

15	7	6	7	15	15	15
0	1	1	1	0	0	0
10	2	2	2	9	10	10
1	1	1	1	1	1	1
0	1	1	1	0	0	0
2	4	1	2	0	0	0
2	0	0	0	2	2	2
3	2	2	2	3	3	3
2	0	0	0	2	2	2
1	0	1	1	2	0	1
13	0	0	0	12	13	13
0	1	1	2	0	0	0
3	1	0	2	3	3	3
0	1	0	0	0	0	0
4	0	0	0	4	4	4
5	6	7	9	5	5	5
1	0	0	0	1	1	1
1	1	1	1	1	1	1
0	0	1	1	0	0	0
1	1	2	2	0	0	0
2	0	0	0	1	1	1
1	1	1	2	1	1	1
2	1	3	3	2	2	2
1	0	0	0	1	1	1
6	7	8	7	6	5	6
1	0	0	0	1	1	1
4	3	3	3	3	3	4
0	1	1	1	0	0	0
7	4	3	4	7	6	7
2	1	0	0	2	2	2
0	3	1	3	0	0	0
0	8	8	7	0	0	0
3	0	0	0	3	3	3
2	0	0	0	2	2	2
7	2	2	2	5	5	5
4	10	11	12	4	4	4
4	0	0	0	3	3	4
4	1	1	0	4	4	4
3	0	0	0	3	3	3
1	0	0	0	1	1	1
3	0	0	0	3	3	3
1	0	0	0	2	2	1
8	0	1	1	8	8	8
1	0	0	0	1	1	1
1	0	0	0	1	1	1

2	0	0	0	2	2	2
1	2	2	2	0	1	1
9	0	0	0	8	8	9
0	1	1	1	0	0	0
1	0	0	0	1	1	1
2	3	2	3	2	2	2
2	2	2	3	2	2	2
2	3	3	3	2	2	2
18	2	2	2	17	17	18
4	0	0	0	4	4	4
3	5	5	6	3	2	3
4	1	1	0	3	4	4
2	2	2	2	2	1	2
1	0	0	0	1	1	1
1	0	0	0	1	0	1
2	1	2	2	2	2	2
1	0	0	0	1	1	1
8	6	6	6	7	8	8
5	3	4	3	5	5	5
1	0	0	0	1	1	1
4	0	0	0	4	3	4
1	1	1	1	1	1	1
1	0	0	0	1	2	1
15	0	0	0	15	15	15
19	7	6	8	11	11	11
6	1	1	1	6	5	6
0	4	4	4	0	0	0
8	2	3	3	9	9	8
2	6	6	6	4	3	2
5	9	10	10	5	5	5
17	11	8	10	16	15	17
0	1	1	1	0	1	0
1	1	1	1	1	1	1
1	0	0	0	1	1	1
2	0	0	0	3	1	2
0	2	1	2	0	0	0
2	0	0	0	1	2	2
1	1	1	1	1	1	1
3	2	2	2	3	3	3
16	7	7	8	16	16	16
2	1	1	1	2	2	2
2	0	0	0	2	2	2
1	0	0	0	1	1	1
0	1	1	1	0	0	0
1	1	1	1	1	1	1

2	1	1	1	2	2	2
3	0	0	0	3	3	3
0	0	0	1	0	0	0
4	2	3	3	4	5	4
22	8	7	8	22	21	22
0	0	0	0	1	0	0
4	0	0	0	3	3	4
1	0	0	0	1	1	1
5	4	5	4	5	4	5
5	0	0	0	5	5	5
6	0	0	0	6	6	6
6	3	4	4	6	6	6
7	1	2	2	8	8	7
4	0	0	0	4	4	4
0	2	2	1	0	0	0
4	0	0	0	4	4	4
1	0	0	0	1	1	1
6	0	0	0	6	6	6
1	0	0	0	1	1	1
9	0	0	0	9	9	9
34	0	0	0	25	25	24
1	0	0	1	1	1	1
6	4	4	4	4	4	6
1	1	1	1	1	1	1
15	0	0	0	13	14	12
2	2	2	4	1	1	1
0	1	1	1	0	0	0
1	0	0	0	1	1	1
1	0	0	0	1	1	1
3	1	1	1	3	3	3
0	1	1	1	0	0	0
2	1	1	1	2	2	2
2	0	0	0	2	2	2
1	1	1	1	1	1	1
2	0	0	0	2	2	2
1	1	0	1	1	1	1
21	2	3	3	21	21	21
1	1	1	1	1	1	1
2	2	2	2	2	2	2
1	0	0	0	1	1	1
5	2	2	2	5	5	5
3	5	4	5	3	3	3
0	1	1	1	0	0	0
9	3	2	3	9	9	9
5	0	0	1	5	5	5

0	5	0	0	0	0	0
0	2	0	0	0	0	0
3	2	2	3	3	3	3
1	1	0	0	1	1	0
0	3	0	0	0	0	0
0	1	0	0	0	0	0
8	10	10	10	8	7	8
18	25	17	19	9	7	7
0	0	1	0	0	0	0
6	3	8	5	5	5	6
1	4	6	3	1	1	1
3	2	3	3	3	3	3
3	2	5	2	2	2	3
6	3	7	5	2	2	6
73	80	80	80	74	74	73
1	0	0	0	1	1	1
16	23	15	17	0	0	0
10	4	4	4	10	9	10
32	43	42	44	31	32	32
7	17	7	10	5	4	7
36	37	36	37	24	24	25
16	11	11	11	2	2	2
0	1	1	1	0	0	0
1	1	1	1	1	1	1
31	26	25	25	31	32	31
1	0	0	0	1	0	1
0	5	1	1	0	0	0
6	8	4	4	4	3	3
0	14	4	1	0	0	0
5	2	2	2	5	6	5
1	0	1	1	1	1	1
1	1	1	1	1	1	1
1	3	1	1	0	0	0
9	1	0	1	7	7	7
1	0	0	0	1	1	1
0	3	0	0	0	0	0
6	3	3	3	4	4	6
0	0	0	0	0	0	0
1	1	1	1	0	0	0
0	4	1	0	0	0	0
0	1	0	0	0	0	0
2	2	2	2	2	2	2
1	22	7	4	0	0	0
5	1	2	2	4	4	5
0	0	0	1	0	0	0

9	1	1	1	2	1	6
10	7	6	7	9	10	10
0	3	1	0	0	0	0
1	1	1	0	0	0	1
7	0	0	0	8	7	7
0	1	0	0	0	0	0
0	2	0	1	0	0	0
12	10	10	10	10	11	12
9	0	0	0	9	9	9
3	0	0	0	3	3	3
1	0	0	0	1	1	1
4	0	0	1	4	4	4
1	1	1	1	1	1	1
3	3	1	2	2	2	3
4	5	5	5	4	4	4
2	0	0	0	2	2	2
3	2	2	2	3	3	3
0	2	3	4	0	0	0
2	2	2	2	2	2	2
1	2	1	1	1	0	1
4	0	0	0	4	5	4
3	3	3	3	4	3	3
2	3	2	2	3	3	2
6	0	0	0	5	5	6
16	19	18	19	14	15	16
3	4	6	7	3	3	3
1	0	0	0	1	1	1
5	1	1	1	5	4	5
0	1	0	0	0	0	0
1	1	2	2	1	1	1
3	0	0	0	3	2	3
3	0	0	0	1	1	1
6	8	8	8	6	5	6
1	1	1	1	1	1	1
7	3	3	4	7	7	7
3	2	2	3	3	3	3
1	1	1	1	1	1	1
5	1	1	2	5	5	5
2	2	2	2	2	2	2
1	0	0	0	1	1	1
4	2	2	2	4	4	4
1	1	0	1	1	1	1
5	2	2	2	5	5	5
2	0	0	0	2	2	2
1	1	1	1	1	1	1

7	0	0	0	3	3	3
1	0	0	0	1	1	1
0	1	1	1	0	0	0
9	8	7	9	9	9	9
9	1	2	2	9	9	9
0	1	1	1	0	0	0
2	0	0	0	2	2	2
18	2	2	2	18	17	18
7	0	0	0	7	7	7
2	2	2	2	2	2	2
1	0	0	0	1	1	1
3	0	0	0	2	2	2
1	1	1	1	1	1	1
3	0	0	0	3	1	3
1	1	1	1	2	2	1
0	0	0	1	0	0	0
27	17	17	19	27	29	27
4	2	3	3	4	4	4
1	0	0	0	1	1	1
12	2	2	2	12	11	12
6	6	4	7	6	5	6
1	0	0	0	1	1	1
6	8	9	9	6	6	6
6	1	1	1	6	6	6
1	0	0	0	1	1	1
1	0	0	0	1	1	1
21	9	10	10	19	21	21
3	0	0	0	4	4	3
1	1	1	1	1	1	1
3	0	0	0	3	3	3
20	16	16	17	12	13	13
0	1	1	1	0	0	0
3	0	0	0	2	3	3
9	1	1	1	1	1	1
4	0	0	0	4	4	4
3	5	5	5	3	3	3
1	0	0	0	1	0	1
2	1	0	2	2	2	2
11	6	7	8	10	11	11
0	0	0	0	0	1	0
1	0	0	0	1	1	1
7	4	4	4	7	7	7
2	0	1	1	1	2	2
4	10	9	11	4	4	4
20	19	20	20	20	21	20

1	1	0	0	1	1	1
1	0	0	0	1	1	1
1	1	1	1	1	1	1
1	0	0	0	0	1	1
5	0	0	0	6	6	5
41	35	36	37	0	0	0
2	0	0	0	2	2	2
10	2	4	3	9	10	10
4	5	5	5	2	2	2
2	2	2	2	2	2	2
2	0	0	0	0	0	0
9	8	8	9	9	10	9
0	1	1	1	0	0	0
4	1	1	1	4	4	4
2	3	2	3	0	0	0
20	0	0	0	18	18	19
5	4	4	4	5	5	5
1	0	0	0	1	1	1
3	0	0	0	2	2	3
1	1	1	1	1	1	1
1	0	1	1	1	1	1
0	4	4	4	0	0	0
0	1	1	1	0	0	0
8	6	6	6	7	8	8
11	4	4	4	11	11	11
0	1	1	1	0	0	0
1	1	1	1	1	1	1
7	2	2	2	7	7	7
1	0	0	0	1	1	1
2	1	1	1	2	2	2
0	1	0	0	0	0	0
9	3	3	3	9	8	9
32	28	27	32	24	24	24
3	2	2	2	3	3	3
12	13	12	13	11	11	11
3	2	2	2	2	2	3
2	0	0	0	2	2	2
2	1	1	1	2	2	2
6	5	5	7	7	7	6
11	8	8	8	10	10	10
4	0	0	0	4	5	4
2	0	0	0	2	2	2
7	0	0	0	6	6	7
4	4	4	4	5	5	4
4	2	2	2	4	4	4

1	0	0	0	1	1	1
0	1	1	1	0	0	0
2	1	1	1	2	2	2
3	0	0	0	3	3	3
1	0	0	0	1	1	1
2	0	0	0	1	1	1
2	2	2	2	2	2	2
34	10	9	10	35	33	34
2	0	0	0	2	2	2
1	1	0	1	1	1	1
1	1	1	1	1	1	1
0	0	0	0	1	1	0
7	2	2	2	7	7	7
5	0	0	0	5	5	5
24	0	1	2	24	24	24
1	0	0	0	1	1	1
8	2	2	3	8	8	8
2	0	0	0	2	2	2
15	2	3	3	15	15	15
15	10	10	10	15	13	15
7	1	1	1	7	7	7
7	0	0	0	7	7	7
3	0	1	1	2	2	3
12	2	2	2	11	11	12
1	0	0	0	1	1	1
7	9	9	9	7	7	7
2	0	0	0	2	2	2
8	2	1	3	8	7	8
16	15	13	13	11	11	12
0	2	2	2	0	0	0
3	2	2	2	2	3	3
9	10	8	12	8	9	9
18	1	0	1	18	18	18
3	5	5	5	3	3	3
1	0	0	0	1	0	1
10	8	7	9	8	9	10
12	5	7	7	12	12	12
4	0	0	0	5	3	4
9	0	0	0	9	9	9
3	1	2	2	2	3	3
1	2	2	2	2	1	1
4	2	1	2	4	4	4
7	6	7	7	7	6	7
3	1	1	1	3	3	3
4	7	9	9	4	4	3

2	4	3	3	2	2	2
12	6	8	9	11	10	12
3	3	3	2	3	2	3
13	3	4	4	12	12	13
14	1	1	1	15	15	14
4	5	5	6	4	4	4
0	1	1	2	0	0	0
1	0	0	0	1	1	1
2	0	0	0	1	2	2
12	2	2	2	12	12	12
13	0	0	0	14	13	13
0	5	0	1	0	0	0
14	6	8	9	14	13	14
1	1	1	1	1	1	1
16	9	10	12	15	15	16
7	7	8	8	7	7	7
0	1	0	1	0	0	0
1	0	0	0	1	1	1
0	1	1	1	0	0	0
3	0	0	0	3	3	3
1	0	0	0	1	1	1
4	0	0	0	4	4	4
8	3	3	3	8	8	8
8	5	6	5	7	8	8
2	0	0	0	1	2	2
47	36	35	36	45	45	45
3	3	3	3	3	3	3
1	0	0	0	0	0	0
6	6	5	6	6	6	6
6	2	2	2	6	5	6
12	1	1	0	12	12	12
9	0	0	0	8	8	9
0	1	1	1	0	0	0
13	11	11	12	6	7	6
5	3	3	3	6	5	5
11	12	12	12	11	11	11
6	3	3	4	5	5	5
6	0	0	0	6	5	6
1	0	0	0	1	1	1
5	2	2	2	5	5	5
24	3	3	5	22	23	23
7	7	7	7	6	7	7
8	19	15	18	7	8	8
2	0	0	1	2	2	2
0	1	1	2	0	0	0

1	0	0	0	1	1	1
40	11	9	9	38	38	38
35	14	14	16	29	29	29
41	18	17	17	31	30	31
2	0	0	0	2	2	2
4	3	2	3	4	4	4
1	2	0	1	1	1	1
5	2	2	2	5	5	5
8	5	5	5	9	8	8
21	21	19	19	19	20	20
44	50	42	42	45	46	44
8	8	8	7	8	9	8
9	10	11	11	2	2	2
6	0	0	0	6	6	6
2	1	1	1	2	2	2
2	2	2	2	2	2	2
1	0	0	0	1	1	1
12	6	6	6	12	12	12
9	0	0	0	9	9	9
5	3	2	3	4	4	5
32	2	1	1	10	9	9
3	0	0	0	3	3	3
28	12	11	13	25	26	26
21	10	9	9	21	21	21
1	1	0	1	1	1	1
14	7	6	7	13	13	13
4	0	0	0	3	4	3
4	5	3	4	4	4	4
4	1	1	1	4	4	4
0	4	3	2	0	0	0
10	4	5	6	9	10	10
6	4	4	4	6	6	6
5	3	3	3	5	5	5
8	5	4	5	8	8	8
9	9	7	9	9	9	9
0	1	1	1	0	0	0
11	13	13	15	10	9	10
12	8	9	10	11	11	12
5	0	0	0	5	5	5
6	2	1	2	6	6	6
0	1	1	0	0	0	0
9	11	13	13	9	9	9
6	4	5	5	6	5	6
0	2	2	2	0	0	0
2	0	0	0	3	3	2

18	0	0	0	19	18	18
1	4	2	2	0	0	0
0	3	4	4	0	0	0
9	0	0	0	8	8	9
10	0	0	0	10	10	10
8	4	2	4	8	8	8
12	4	4	5	12	12	12
9	12	12	12	9	9	9
47	47	42	46	1	1	1
4	0	0	0	5	4	4
19	9	10	10	18	19	19
4	5	6	6	4	4	4
12	9	11	11	11	12	12
8	11	8	11	8	8	8
13	8	8	9	13	13	13
4	2	2	2	4	4	4
5	5	6	6	6	6	5
2	0	0	0	2	2	2
1	0	0	0	1	1	1
28	26	26	27	19	18	19
7	1	1	1	6	7	7
2	1	1	1	2	1	2
13	0	0	0	13	12	13
15	7	8	9	15	14	15
9	10	10	12	9	10	9
3	2	2	2	3	3	3
3	2	2	2	3	3	3
3	0	0	0	3	3	3
2	0	0	0	2	2	2
1	0	0	0	1	1	1
4	1	0	1	4	3	4
7	0	0	0	7	7	7
3	0	0	0	2	2	3
3	4	4	4	3	3	3
1	2	2	2	1	1	1
1	1	1	1	1	1	1
8	1	1	2	8	8	8
12	8	10	10	12	12	12
22	28	29	30	23	23	22
11	1	2	2	11	11	11
1	0	0	0	1	0	1
5	0	0	0	5	4	5
28	0	0	0	30	31	28
4	4	4	5	3	3	4
13	17	16	17	1	1	1

43	15	16	19	42	43	43
1	2	2	2	1	1	1
18	0	0	0	20	20	18
8	0	0	0	8	8	8
1	0	0	0	1	1	1
2	4	3	3	2	2	2
1	0	0	0	1	1	1
1	1	1	1	1	1	1
9	0	0	0	10	9	9
2	3	3	3	2	2	2
31	29	29	31	1	1	2
6	6	6	7	6	4	6
5	3	3	5	5	5	5
1	0	0	0	1	1	1
1	2	2	1	1	1	1
0	2	1	1	0	0	0
1	0	0	0	1	1	1
0	1	1	0	1	1	0
4	3	3	3	4	4	4
7	3	2	2	8	8	7
10	3	2	2	10	10	10
2	1	1	1	2	2	2
8	7	7	7	8	8	8
8	2	2	2	8	8	8
9	2	4	3	8	9	9
1	1	1	1	1	1	1
10	7	6	6	9	10	10
0	2	1	2	0	0	0
1	2	1	2	1	2	1
3	3	3	3	3	3	3
2	3	3	2	2	2	2
12	6	7	7	12	11	12
5	5	5	5	4	5	5
8	3	3	3	9	9	8
3	0	0	0	2	3	3
0	0	0	0	1	1	0
1	0	1	1	1	1	1
6	6	6	6	6	6	6
8	7	6	6	8	8	8
9	6	5	6	9	9	9
1	1	1	1	1	1	1
8	6	7	7	8	8	8
3	0	0	0	3	3	3
4	4	5	5	4	4	4
1	2	1	1	1	1	1

17	16	16	17	1	1	1
6	1	1	1	6	6	6
19	12	12	11	13	11	12
21	10	12	12	3	4	4
24	15	15	16	0	0	0
2	0	0	0	2	2	2
6	2	2	2	7	6	6
6	1	1	2	6	6	6
2	0	0	0	2	2	2
122	69	69	81	120	120	122
0	1	1	1	0	0	0
2	2	2	2	2	2	2
0	4	4	3	0	0	0
20	18	18	19	19	19	20
7	10	10	9	7	5	7
7	0	0	0	6	6	7
5	1	1	1	4	5	5
2	2	1	1	2	2	2
7	6	5	5	7	7	7
7	5	5	4	7	6	7
26	15	16	17	24	25	26
27	2	2	2	26	28	26
1	2	2	2	1	1	1
3	0	0	0	3	3	3
9	5	4	5	9	9	9
4	2	2	3	4	4	4
13	3	4	4	12	12	13
13	15	14	15	10	10	10
0	2	1	1	0	0	0
7	9	6	8	6	7	7
2	3	2	2	1	2	2
9	5	5	5	5	5	5
10	1	1	1	11	12	10
13	6	6	7	14	14	13
22	0	0	0	20	22	22
2	2	3	3	2	2	2
5	2	2	2	5	5	5
4	0	0	0	3	3	3
6	5	5	5	6	6	6
13	0	0	0	14	14	13
10	14	16	16	11	11	10
14	16	16	17	2	2	2
6	0	0	0	3	3	3
7	0	0	0	6	7	7
10	6	7	7	9	10	10

4	5	4	4	4	4	4
0	5	4	5	0	0	0
5	0	0	0	6	6	5
11	1	2	1	11	11	11
2	0	0	0	1	1	1
7	13	12	12	1	1	2
5	0	0	0	5	6	5
2	3	2	3	2	2	2
2	4	3	4	3	3	2
2	0	0	0	2	2	2
22	16	18	18	22	22	22
0	1	0	0	0	0	0
23	13	12	15	17	15	17
1	0	0	0	1	1	1
6	11	11	11	5	7	6
2	0	0	0	2	2	2
26	4	4	5	25	25	26
20	23	23	24	21	20	20
7	5	5	5	4	7	7
2	0	1	1	1	1	2
47	0	0	0	46	47	47
16	13	11	14	16	17	16
4	0	0	0	4	3	4
18	5	4	6	19	19	18
1	0	1	0	1	1	1
2	2	1	2	2	2	2
1	0	0	0	1	1	1
1	1	1	1	1	1	1
5	0	0	0	5	5	5
15	1	0	0	13	14	12
11	11	11	10	8	8	7
1	1	1	1	1	1	1
14	17	18	17	14	14	14
5	2	4	4	5	5	5
5	4	5	5	5	5	5
6	2	2	2	6	7	6
6	0	0	0	6	5	6
3	4	4	4	3	3	3
38	0	0	0	39	36	38
4	6	8	8	4	5	4
0	2	0	0	0	0	0
2	0	0	0	2	2	2
1	3	3	3	1	0	1
0	1	0	0	0	0	0
1	0	0	0	1	1	1

2	2	2	3	2	2	2
1	0	0	0	1	1	1
6	1	1	1	6	6	6
3	0	0	0	3	3	3
6	3	3	4	6	6	6
7	3	2	2	8	8	7
1	1	1	1	1	1	1
1	0	0	0	0	0	0
7	8	9	10	7	7	7
13	8	7	9	13	13	13
7	0	0	0	6	5	7
0	1	0	1	0	0	0
1	0	0	0	1	1	1
5	0	0	0	5	5	5
3	3	3	4	3	3	3
3	0	0	0	3	3	3
3	0	0	0	3	4	3
0	3	5	5	0	0	0
10	0	0	0	10	10	10
13	1	2	3	13	12	13
8	6	6	7	8	7	8
11	2	2	2	11	11	11
3	0	0	0	3	3	3
0	1	1	1	0	0	0
3	0	0	0	3	3	3
2	0	0	0	2	2	2
11	4	6	5	10	8	11
2	2	2	2	2	1	2
0	0	0	1	0	0	0
0	2	2	2	0	0	0
3	3	3	3	1	1	1
6	1	2	2	7	7	6
6	0	0	0	6	6	6
50	17	18	17	49	49	50
4	7	8	7	4	4	4
8	0	0	0	8	6	8
1	0	0	0	1	1	1
19	0	0	1	17	18	19
13	0	0	0	13	13	13
2	3	3	3	2	2	2
8	0	0	0	6	8	8
5	0	0	0	5	6	5
3	0	0	0	3	3	3
33	26	24	25	32	31	33
8	1	1	1	8	8	8

27	1	1	1	28	25	27
3	0	0	0	3	3	3
5	1	2	2	4	3	5
0	1	1	1	0	0	0
5	0	0	0	5	5	5
8	0	0	0	9	8	8
6	0	0	0	6	5	6
14	8	8	8	14	13	14
6	0	0	0	6	6	6
1	0	0	0	1	1	1
5	0	0	0	4	5	5
1	0	0	0	1	1	1
7	1	1	1	7	7	7
2	2	2	2	2	2	2
1	0	0	0	0	1	1
0	1	1	1	0	0	0
1	1	1	1	1	1	1
1	0	0	0	2	2	1
0	4	3	4	0	0	0
2	1	1	1	2	2	2
10	0	0	0	12	12	10
11	1	1	1	11	11	11
3	1	0	0	3	1	3
17	0	0	0	6	5	5
6	2	2	2	6	6	6
1	2	2	3	1	1	1
1	0	0	0	1	1	1
1	0	0	0	1	1	1
0	3	1	0	0	0	0
1	0	0	0	0	0	0
2	0	0	0	2	2	2
3	2	2	2	3	3	3
17	3	3	3	17	17	17
4	6	7	7	4	4	4
7	4	4	4	5	5	7
4	6	6	6	4	4	4
4	0	0	0	4	4	4
0	2	2	2	0	0	0
11	4	4	4	11	10	11
1	0	0	0	1	1	1
4	0	0	0	4	4	4
26	25	22	26	7	7	7
2	0	0	0	2	2	2
7	3	2	3	5	6	7
2	0	1	1	2	2	2

3	0	0	0	3	3	3
12	0	0	0	10	11	12
0	1	0	0	0	0	0
7	1	0	1	6	7	7
6	5	4	5	6	6	6
4	1	2	2	5	5	4
2	0	0	0	2	2	2
3	0	0	0	3	3	3
5	1	1	1	2	2	2
1	0	0	0	0	0	0
1	1	1	1	3	3	1
2	2	2	2	2	2	2
1	4	4	4	1	1	1
1	0	0	0	1	1	1
4	1	1	1	4	4	4
1	2	2	2	1	1	1
5	4	4	5	5	4	5
3	0	0	0	3	4	3
7	6	5	6	7	7	7
22	0	0	0	22	22	22
4	4	3	5	4	4	4
1	0	0	0	1	1	1
0	1	1	1	0	0	0
28	0	0	0	28	27	28
17	0	0	0	15	15	15
4	6	5	6	4	4	4
7	7	7	7	6	6	7
1	1	0	1	1	1	1
2	0	0	0	2	2	2
3	1	1	2	3	2	3
7	0	0	0	7	7	7
4	0	0	0	4	4	4
16	18	17	19	15	15	15
5	2	2	3	5	5	5
75	9	11	10	75	75	75
1	2	2	2	1	1	1
4	4	4	4	4	4	4
21	11	14	15	20	23	21
10	2	2	2	5	5	6
3	5	6	4	4	3	3
32	36	34	36	27	28	28
5	0	0	0	3	3	5
1	1	1	1	1	1	1
1	0	0	0	1	1	1
1	1	1	1	1	1	1

2	0	0	0	2	2	2
4	0	0	0	4	3	4
3	0	0	0	4	4	3
8	12	11	13	7	6	8
0	3	2	3	0	0	0
5	3	4	4	6	5	5
11	14	13	13	11	11	11
2	3	3	3	2	2	2
6	0	0	0	6	6	6
8	0	0	0	8	8	8
1	0	0	0	1	1	1
1	1	1	1	1	1	1
6	3	2	3	6	5	6
2	0	0	0	2	2	2
3	1	1	2	4	3	3
14	0	0	0	15	14	14
2	0	0	0	2	2	2
23	12	12	14	20	23	23
0	2	2	2	0	0	0
2	0	0	0	2	2	2
20	1	1	1	22	21	20
3	0	0	0	3	3	3
5	0	1	1	5	5	5
8	1	1	1	6	6	8
16	0	0	0	14	14	16
21	2	2	3	18	18	17
4	0	0	0	4	4	4
1	0	0	0	1	1	1
3	5	5	5	3	3	3
2	2	3	3	2	2	2
30	23	24	22	32	34	30
9	4	7	6	10	10	9
9	9	9	11	9	9	9
5	7	7	7	5	5	5
25	5	6	7	23	24	25
1	1	0	1	1	1	1
8	10	10	10	8	8	8
5	0	0	0	3	3	3
7	2	2	2	8	7	7
10	0	0	0	8	9	10
5	0	0	0	6	5	5
5	5	5	5	3	3	3
4	1	1	1	4	4	4
3	1	2	2	3	4	3
0	1	1	1	0	0	0

18	0	0	0	18	16	18
3	6	6	6	3	3	3
2	0	0	0	2	2	2
2	0	0	0	2	2	2
2	1	1	1	1	2	2
25	0	0	0	25	25	25
10	0	0	0	10	9	10
3	1	1	1	3	3	3
3	4	5	4	2	3	3
10	2	1	2	7	7	10
1	1	1	1	1	0	1
6	1	1	1	7	6	6
2	0	0	0	2	2	2
5	5	5	5	5	5	5
9	2	2	4	8	9	9
3	0	0	0	3	3	3
2	1	0	1	2	2	2
43	0	0	1	43	41	43
2	2	2	1	2	2	2
2	2	1	2	2	2	2
10	21	21	21	10	10	10
3	2	3	2	3	3	3
1	0	0	0	1	1	1
1	0	0	0	1	1	1
8	0	0	0	8	7	8
38	2	2	2	38	35	37
6	7	6	7	6	5	6
1	2	3	2	1	1	1
21	7	6	7	20	20	20
3	0	0	0	3	3	3
6	0	0	0	6	5	6
0	0	0	0	1	1	0
2	3	2	3	2	2	2
4	2	2	3	3	3	4
1	0	0	0	1	1	1
2	2	0	2	2	2	2
1	0	0	0	2	1	1
28	31	33	33	26	25	26
15	0	0	0	15	12	15
1	0	0	0	1	1	1
2	0	0	0	2	2	2
29	0	0	0	27	29	29
3	6	7	8	2	3	3
2	1	1	1	1	2	2
1	2	2	3	1	1	1

3	0	0	0	3	3	3
6	3	4	3	5	6	6
1	1	0	1	1	0	1
0	5	5	4	0	0	0
3	0	0	0	3	3	3
1	0	0	0	1	1	1
5	0	0	0	5	5	5
6	0	0	0	6	6	6
17	12	13	14	17	16	17
1	0	0	0	2	2	1
2	1	1	1	2	2	2
1	2	2	2	1	1	1
12	0	1	1	12	12	12
4	3	4	3	5	5	4
4	0	0	0	4	4	4
1	0	0	0	2	2	1
4	3	4	4	4	4	4
2	0	0	0	2	2	2
5	6	6	6	5	6	5
0	1	0	0	0	0	0
2	0	0	0	2	2	2
0	1	1	1	0	0	0
12	4	4	4	12	13	12
20	0	1	1	2	2	3
6	0	0	0	6	6	6
4	3	3	2	4	4	4
7	0	0	0	6	8	7
12	0	0	0	11	12	12
14	10	8	10	14	14	14
28	25	21	27	27	27	28
18	9	10	12	20	18	18
1	0	0	0	0	1	1
8	0	0	0	8	8	8
4	0	0	0	4	3	4
11	11	11	15	11	10	11
12	7	6	8	11	10	12
19	14	15	16	16	17	18
18	10	10	10	19	18	18
27	0	0	0	27	26	27
15	13	14	15	9	9	9
1	0	0	0	1	1	1
4	7	9	10	4	4	4
2	1	1	1	2	2	2
2	1	2	2	2	2	2
3	2	2	2	3	3	3

2	3	3	3	2	2	2
5	9	9	10	6	6	5
24	0	0	0	22	22	24
3	2	2	2	3	3	3
17	5	5	5	18	17	17
21	8	9	10	18	20	21
4	3	3	1	6	5	4
5	2	2	2	4	6	5
1	1	0	0	0	0	1
1	1	1	1	1	1	1
0	0	0	1	0	0	0
1	0	0	0	1	1	1
0	1	1	1	0	0	0
1	1	1	1	1	1	1
1	1	1	0	1	1	1
0	1	1	1	0	0	0
1	0	0	0	0	0	1
1	1	1	0	1	1	1
0	0	1	0	0	0	0
1	0	0	0	1	1	1
0	0	0	0	1	0	0
0	1	1	0	0	0	0
1	0	0	0	1	0	1
1	1	1	1	1	1	1
1	1	1	1	1	1	1
1	1	1	1	1	1	1
1	0	0	0	1	1	1
9	11	11	13	7	8	9
6	4	5	5	6	5	6

Unique pepti	Unique pepti	Unique pepti	Sequence co	Unique + raz	Unique sequ	Mol. weight [
2	3	3	60	60	60	10.715
1	1	2	37.4	37.4	37.4	19.889
1	1	1	17.8	17.8	17.8	17.17
10	10	10	15.1	15.1	15.1	89.748
5	4	5	16.8	16.8	16.8	56.662
1	1	1	6.7	6.7	6.7	53.987
0	0	0	4.8	4.8	4.8	38.86
5	4	5	44.5	44.5	44.5	18.732
6	6	7	19.9	19.9	19.9	51.556
0	0	0	5.6	5.6	5.6	45.615
3	4	4	9.7	9.7	9.7	52.881
2	2	2	13.8	9.2	9.2	27.502
4	3	4	18.8	18.8	18.8	36.925
1	1	1	3	3	3	106.56
0	0	0	13.4	13.4	13.4	31.293
0	0	0	7.8	7.8	7.8	27.478
4	5	8	34.3	34.3	34.3	43.359
0	0	0	12	12	12	96.631
5	5	6	26.4	26.4	26.4	153.94
4	3	3	7.7	7.7	7.7	116.99
5	6	6	41.3	41.3	41.3	13.952
1	1	1	35.4	8.5	8.5	14.105
18	15	18	18.3	18.3	18.3	148.09
0	0	0	10.6	10.6	10.6	39.607
0	0	0	23.3	23.3	23.3	67.32
1	1	1	6.8	6.8	6.8	16.698
2	2	2	20.5	20.5	20.5	59.975
0	0	0	13.4	13.4	13.4	12.051
1	2	2	13.1	13.1	13.1	84.821
0	0	0	28.3	28.3	28.3	57.436
1	0	0	29.5	29.5	29.5	39.646
0	0	0	11.1	2.1	2.1	80.559
0	0	0	4.7	2.1	2.1	82.279
2	2	2	3.9	3.9	3.9	54.705
2	2	2	3.9	3.9	3.9	93.233
0	0	0	4.7	4.7	4.7	30.262
3	4	4	14.7	14.7	11.2	57.255
0	0	0	4.2	4.2	4.2	64.446
0	0	0	16.2	16.2	16.2	78.608
3	2	4	10.4	10.4	10.4	71.026
0	0	0	1.2	1.2	1.2	94.659
0	0	0	15.8	15.8	15.8	135.65
1	0	2	9	9	9	37.306
4	4	5	15.2	12	12	59.689

2	3	3	10.8	10.8	10.8	105.67
1	1	1	3.7	3.7	3.7	91.838
3	3	3	9.8	9.8	9.8	58.823
0	0	0	1.3	1.3	1.3	240.76
0	0	0	26.7	25	25	45.257
0	0	0	9.6	9.6	9.6	39.686
0	0	0	13	5.7	5.7	28.218
3	3	3	4.5	4.5	4.5	88.885
5	4	5	13.4	13.4	13.4	64.24
0	0	0	11	11	11	80.31
1	1	1	63.4	63.4	63.4	14.149
1	0	1	3.9	3.9	3.9	62.72
5	5	5	27.1	27.1	27.1	33.433
0	0	0	30.4	30.4	30.4	73.806
3	0	0	34.8	23.5	8.9	47.596
0	0	1	4	4	4	119.77
5	7	6	18.6	18.6	18.6	63.541
3	2	2	48.2	48.2	48.2	12.405
0	0	0	30.5	30.5	30.5	6.678
6	6	6	53.2	53.2	53.2	14.257
0	0	0	41	41	35.8	37.788
2	1	2	16.6	9.3	7.9	81.744
1	0	1	1.7	1.7	1.7	50.965
0	0	0	8.8	8.8	8.8	19.83
0	0	0	24	24	24	30.785
23	23	23	54.6	54.6	54.6	50.976
0	0	0	6.1	6.1	6.1	51.471
5	6	5	12	12	12	131.61
3	3	3	28.4	28.4	28.4	19.586
0	0	0	8.3	8.3	8.3	13.655
0	0	0	4.7	4.7	4.7	52.743
0	0	0	7.9	7.9	7.9	41.392
0	0	0	7.9	7.9	7.9	164.13
4	4	4	48.1	48.1	48.1	15.616
1	1	1	12.3	12.3	12.3	56.275
0	0	0	21.7	21.7	21.7	88.131
3	3	3	34.6	34.6	34.6	29.404
5	4	5	25.1	25.1	25.1	31.821
0	0	0	6.1	4.7	4.7	77.518
4	4	4	39.7	39.7	39.7	24.146
0	0	0	6.5	6.5	6.5	50.303
1	1	1	7.4	7.4	7.4	92.153
1	1	0	15.3	15.3	15.3	60.572
0	0	0	3.1	3.1	3.1	78.15
1	0	1	14.6	14.6	14.6	17.553

2	2	2	11.2	11.2	11.2	23.144
3	3	4	20.3	20.3	20.3	27.508
1	1	2	16.7	11.3	11.3	32.287
1	2	2	24	24	24	11.065
0	0	0	3.6	3.6	3.6	350.68
1	1	1	3.8	3.8	3.8	41.149
18	17	18	27	27	27	113.08
0	0	0	4.8	4.8	4.8	32.544
4	4	3	11.4	11.4	11.4	52.425
0	0	0	41	41	41	18.154
0	0	0	12.4	12.4	12.4	24.763
3	3	3	28.8	28.8	28.8	61.054
1	1	1	48.3	48.3	48.3	66.846
5	4	4	37.8	37.8	37.8	22.042
4	4	4	8.3	8.3	8.3	65.971
0	0	0	24.5	24.5	24.5	164.9
0	0	0	4.7	4.7	4.7	79.222
15	14	18	34.5	34.5	34.5	76.613
5	5	6	25.5	25.5	25.5	41.632
2	1	1	15.9	15.9	15.9	21.02
0	0	0	20.7	20.7	20.7	31.969
2	1	2	25.8	25.8	25.8	22.591
3	4	4	47	47	47	22.391
1	1	1	9.3	9.3	9.3	20.9
0	0	0	9.6	9.6	9.6	61.573
0	0	0	6.8	6.8	6.8	50.144
1	2	2	4.2	4.2	4.2	59.57
5	5	6	24.3	24.3	24.3	81.074
7	7	7	28.1	28.1	23.5	75.022
9	9	10	32.2	32.2	25.6	61.888
0	0	0	12.9	12.9	12.9	37.723
1	1	1	10.2	10.2	10.2	44.155
0	0	0	4.5	4.5	4.5	67.814
0	0	0	8.5	8.5	8.5	20.996
0	0	0	3.2	3.2	3.2	88.367
0	0	0	4.6	4.6	4.6	54.461
0	0	0	22.4	22.4	22.4	58.575
1	1	1	10.6	10.6	10.6	15.301
0	0	0	68.3	68.3	0.6	236.51
1	1	2	7.8	7.8	7.8	27.151
0	0	0	8	8	8	71.855
1	1	1	18.2	18.2	18.2	81.336
0	0	0	20.6	20.6	20.6	8.014
14	13	14	57.5	57.5	57.5	38.576
1	1	0	6.4	6.4	6.4	37.558

1	1	1	14.2	14.2	14.2	11.399
4	4	4	23.3	23.3	23.3	114.99
5	5	5	50	50	50	12.259
2	3	3	16.5	13.2	13.2	31.263
0	0	0	5.1	5.1	5.1	73.013
1	1	1	5.9	5.9	5.9	29.654
0	0	0	5.7	5.7	5.7	57.464
0	0	0	27.7	27.7	27.7	45.745
0	0	0	19.7	19.7	19.7	60.542
2	2	2	25.7	25.7	25.7	12.37
0	0	0	24.7	24.7	24.7	21.508
0	0	0	2.4	2.4	2.4	157.98
1	1	1	5.9	5.9	5.9	85.737
0	0	0	16.8	16.8	16.8	37.106
3	3	3	8.2	8.2	8.2	67.423
0	0	0	12.6	12.6	12.6	14.056
2	1	2	22.8	22.8	1.8	95.337
1	0	1	5.9	5.9	5.9	57.258
3	3	3	22.6	22.6	2.8	183.16
5	5	6	30.1	30.1	30.1	120.21
1	1	1	8.7	8.7	8.7	46.806
1	0	1	3.4	3.4	3.4	49.516
3	3	4	10.4	10.4	10.4	79.727
8	8	10	46.5	40	40	45.671
0	0	0	12.1	5.4	5.4	25.915
0	0	0	7.9	7.9	7.9	92.722
0	0	0	9.6	7.2	6.5	335.92
0	0	1	36.9	36.9	36.9	6.968
2	2	2	36.3	36.3	19.4	29.225
1	1	1	14.1	14.1	14.1	97.769
0	0	0	1.5	1.5	1.5	287.31
0	0	0	7.9	7.9	7.9	35.144
1	1	1	24	24	24	10.645
0	0	0	9.9	9.9	9.9	91.124
2	2	2	1.9	1.9	1.9	282.25
1	0	1	8.6	8.6	8.6	31.124
0	0	0	8	8	8	22.655
2	1	2	29	27.6	26	61.64
0	0	0	7.4	7.4	7.4	152.47
1	2	2	4.3	4.3	4.3	103.05
1	2	2	13	13	13	25.387
1	1	1	12	12	12	25.468
2	3	3	39.1	39.1	39.1	75.649
3	2	2	33.7	33.7	33.7	10.871
10	9	13	29.5	29.5	29.5	55.381

7	7	6	56.4	56.4	50.4	15.371
11	13	13	17.2	17.2	17.2	122.65
5	5	6	18.7	18.7	14.8	46.871
0	0	0	7	7	7	58.683
3	0	0	4.1	4.1	4.1	80.532
0	0	0	12.8	12.8	12.8	59.152
4	5	5	30.5	30.5	30.5	47.334
10	10	10	68.7	68.7	68.7	12.784
1	1	1	24	24	24	11.315
0	0	0	6.8	6.8	6.8	158.55
1	2	2	16.3	16.3	16.3	71.689
0	0	0	14.5	14.5	14.5	35.818
0	0	0	15.7	15.7	15.7	46.573
0	0	0	17.6	17.6	17.6	10.632
1	1	1	13.1	13.1	13.1	208.88
0	0	0	5.4	5.4	5.4	25.223
1	2	2	23.7	23.7	23.7	65.352
0	0	0	27.2	27.2	27.2	93.697
0	0	0	2.5	2.5	2.5	96.146
8	7	8	29.4	29.4	29.4	119.9
1	0	0	24.2	24.2	24.2	52.206
16	16	18	53.5	53.5	53.5	37.429
0	0	0	2.5	2.5	2.5	120.27
23	22	25	65.3	65.3	65.3	56.084
0	0	0	5.5	5.5	5.5	48.799
9	10	10	21	21	21	75.406
2	2	2	10.7	10.7	10.7	37.75
2	2	2	35.9	35.9	14.1	13.936
8	10	11	36.5	36.5	36.5	50.646
3	4	4	33.2	31.5	29.2	43.774
13	11	12	53.4	53.4	53.4	33.155
5	5	4	33.6	33.6	29	27.164
2	2	2	31.3	31.3	31.3	13.015
5	5	5	15.2	15.2	9.6	127.38
1	1	1	10.6	10.6	10.6	18.697
1	1	1	21.1	21.1	21.1	7.813
1	1	1	12	12	12	24.347
10	10	10	67.9	67.9	67.9	20.954
4	4	3	8.5	8.5	8.5	99.996
4	4	4	45.4	18.7	18.7	50.67
3	4	6	32.1	32.1	32.1	39.932
1	1	1	4.6	4.6	4.6	40.504
5	3	5	19.2	19.2	19.2	53.248
1	1	1	4.8	4.8	4.8	56.313
6	6	6	39.6	39.6	39.6	32.741

2	2	2	28.2	28.2	8.2	33.773
1	1	0	25	25	25	17.77
1	1	1	18.4	14.9	14.9	30.283
0	0	1	28.4	28.4	28.4	34.301
0	0	0	6.9	6.9	6.9	221
1	1	0	18.4	18.4	18.4	51.082
4	4	4	13.4	13.4	13.4	30.601
0	1	1	6.1	6.1	6.1	37.031
0	0	0	8.7	8.7	6.2	42.155
4	4	4	6.7	6.7	6.7	107.54
1	0	1	17.7	17.7	17.7	71.606
0	0	0	1.5	1.5	1.5	161.91
2	2	2	24.3	24.3	24.3	11.665
0	0	0	8.9	8.9	8.9	65.683
0	0	0	4.1	4.1	2.5	70.222
0	0	0	23.4	23.4	23.4	49.854
3	3	3	22.9	22.9	15.6	23.782
4	3	4	15.2	15.2	15.2	48.991
0	0	0	8.5	8.5	8.5	31.483
2	1	2	46.5	45.8	45.8	129.03
0	0	0	5.8	5.8	5.8	118.7
1	1	1	23.2	23.2	23.2	73.045
1	1	1	23.4	23.4	23.4	11.676
0	0	0	3.7	3.7	3.7	39.599
0	0	0	10.2	10.2	10.2	18.237
0	0	0	12.2	12.2	12.2	13.172
2	2	2	5.8	5.8	5.8	56.626
0	0	0	5	5	5	30.742
0	0	0	4.2	4.2	4.2	270.21
0	0	0	2.4	2.4	2.4	129.52
0	0	0	7.7	4.3	4.3	40.295
0	0	0	17	17	17	20.351
5	5	5	9.5	9.5	9.5	72.313
8	6	7	41.8	41.8	39.2	32.834
1	0	2	10.7	7.7	7.7	30.214
2	1	2	14.5	14.5	14.5	13.293
1	1	1	17.5	17.5	17.5	22.348
0	0	0	6.8	6.8	6.8	46.666
0	0	0	7.6	7.6	7.6	53.68
0	0	0	3.6	3.6	3.6	45.57
2	2	2	45.2	45.2	45.2	34.832
3	2	3	32.3	32.3	24.7	18.311
0	0	0	11.7	11.7	11.7	154.91
0	0	0	2.9	2.9	2.9	58.781
4	4	4	16.1	16.1	16.1	26.6

0	0	0	8.6	8.6	8.6	81.23
6;U6BZQ8;U5XMI1;U4QB70;U3R9Y1;T2MKA2;T2MJM6;T2MJ84;T2ET92;T2BQE4;T2B9H9;T1WQ06;T1WGK5;T1WE98;T1RT9						
;F8R8J6;F8R8J2;F8R8I9;F8R8I8;F8R8I7;F8R8I6;F8R8I5;F8R8I4;F8R8I3;F8R8I2;F8R8I1;F8R8H9;F8R8H8;F8R8H7;F8R8H6;F8R8						
5ER51;U6BZR6;U6BZQ8;U5XMI1;U4QB70;U3R9Y1;T2MKA2;T2MJM6;T2MJ84;T2ET92;T2BQE4;T2B9H9;T1WQ06;T1WGK5;T						
;F8R8J6;F8R8J2;F8R8I9;F8R8I8;F8R8I7;F8R8I6;F8R8I5;F8R8I4;F8R8I3;F8R8I2;F8R8I1;F8R8H9;F8R8H8;F8R8H7;F8R8H6;F8R8						
1	1	18.4	18.4	18.4	23.486	206
810470	300330	196590	413630	9392300	9799300	8313500
4	3	3	41.5	41.5	41.5	33.172
0	0	0	14.9	14.2	2.1	150.6
0	0	0	19.5	19.5	19.5	12.891
0	0	0	3.9	3.9	3.9	129.6
0	0	0	20.3	20.3	20.3	67.559
0	0	0	5.3	1.8	1.8	553.12
2	2	2	8.9	8.9	8.9	40.078
0	0	1	36.1	36.1	36.1	17.629
1	0	0	5.2	5.2	5.2	19.72
0	1	1	6.8	6.8	6.8	24.625
13	13	13	31.3	31.3	30	89.813
0	0	0	23.3	23.3	23.3	11.852
1	1	1	11.5	11.5	11.5	17.027
0	0	0	2.4	2.4	2.4	169.09
1	1	1	25.2	25.2	25.2	17.254
0	0	0	3.9	3.9	3.9	40.471
0	0	0	24.9	24.9	24.9	27.741
0	0	0	17.6	17.6	17.6	15.197
1	1	1	8.5	8.5	8.5	30.27
129	125	134	59	59	52.8	358.62
9	9	10	14.7	14.7	14.7	89.301
0	0	0	10.9	10.9	10.9	15.159
4	6	6	16.6	12.4	12.4	72.157
0	0	0	13.7	13.7	13.7	39.679
0	0	0	4.9	4.9	4.9	79.638
0	0	0	0.3	0.3	0.3	506.27
0	0	0	2.9	2.9	2.9	79.825
0	0	0	1.9	1.9	1.9	121.25
0	0	0	9.2	9.2	9.2	21.459
1	1	1	23.8	23.8	23.8	91.995
0	0	0	4	4	4	217.17
9	9	9	34.2	34.2	34.2	44.253
3	3	3	6.5	6.5	6.5	102.13
0	0	0	3.9	3.9	3.9	45.705
0	0	0	5.1	5.1	5.1	32.579
0	0	0	3.7	2.6	2.6	112.72
1	1	1	3.9	3.9	3.9	41.851
0	0	1	21.6	21.6	21.6	12.566

3	3	3	30.1	30.1	30.1	16.523
0	0	0	8	8	8	64.424
0	0	0	6.4	6.4	6.4	19.054
1	1	1	7.7	7.7	7.7	22.642
0	0	0	12	12	8.8	61.163
1	1	1	12.2	12.2	12.2	11.405
1	0	0	15.7	15.7	15.7	13.242
0	0	0	10.6	2.8	0	60.453
0	0	0	13.5	13.5	13.5	10.831
2	2	2	30.2	30.2	30.2	14.419
3	2	2	15.7	15.7	15.7	30.302
8	7	9	24.5	24.5	24.5	97.584
1	1	1	5.9	5.9	5.9	31.225
5	5	6	9.1	9.1	9.1	114.2
1	1	1	5.7	5.7	5.7	47.708
0	0	0	6.8	6.8	6.8	20.225
0	0	0	4.2	4.2	4.2	32.111
1	1	1	3	3	3	59.196
3	4	4	36	36	36	17.198
3	1	1	36.4	8.7	5.9	46.213
1	1	1	8.1	8.1	8.1	81.037
5	5	5	33.9	33.9	33.9	26.106
1	1	1	2	2	2	74.824
1	0	1	8	8	8	37.898
0	0	0	7	7	7	103.53
0	0	0	10.5	10.5	10.5	33.488
0	0	0	12.5	12.5	12.5	12.651
12	12	12	22.2	22.2	18.6	73.602
0	0	0	6.4	6.4	6.4	90.131
0	0	0	14	4.1	4.1	113.36
1	1	1	11.3	11.3	11.3	17.394
0	0	0	2.8	2.8	2.8	42.637
0	0	0	4	4	4	152.47
3	3	3	14	9	9	47.441
1	1	1	8.3	8.3	8.3	42.591
0	0	0	7	7	7	35.334
6	6	6	41.2	41.2	41.2	32.193
0	0	0	49.8	49.8	47.9	61.899
1	1	1	9.3	9.3	9.3	16.687
4	4	3	6.7	6.7	6.7	99.365
0	0	0	8.4	8.4	8.4	51.419
0	0	0	0.8	0.8	0.8	206.89
0	0	0	8.5	8.5	8.5	20.238
1	2	2	8.7	8.7	8.7	47.471
0	0	0	2.7	2.7	2.7	58.096

0	0	0		8		8		8		66.257
4	3	2	10.8		10.8		10.8			53.193
0	0	0		5		5		5		71.085
6	6	6	53.3		53.3		53.3			15.763
0	0	0	13.1		13.1		13.1		11.6	
1	1	1	11.5		11.5		11.5			11.488
0	0	0	4.2		4.2		4.2		112.96	
0	0	0	2.8		2.8		2.8			54.943
1	2	3	18.9		18.9		18.9			62.553
1	1	1	3.6		3.6		3.6			53.225
6	6	6	33.2		33.2		33.2			23.687
18	18	20	21.8		21.8		21.8		117.47	
10	9	11	13.8		13.8		13.8		111.97	
6	6	6	17.7		17.7		14.7		215.28	
3	2	3	22.7		22.7		22.7			15.395
0	0	0	5.8		5.8		5.8			91.054
5	4	5	15.2		15.2		15.2			37.404
0	0	0	1.7		1.7		1.7			81.459
28	29	31	60.8		59.1		59.1			70.839
0	0	0	19.2		19.2		19.2			51.556
6	7	7	33.7		33.7		33.7		41.29	
0	0	0	3.4		3.4		3.4		119.1	
0	0	0		8		8		8		55.105
3	2	3	23.2		23.2		23.2			22.806
0	0	0	42.6			37	1.6			70.051
0	0	0	5.3		5.3		5.3			35.817
3	0	0		16		16		16		19.363
2	2	1		11		11	10.2		174.88	
0	0	0	5.1		5.1		5.1		25.74	
1	1	1	32.5		32.5		32.5			37.865
1	1	1	5.9		5.9		5.9			50.294
1	1	1	16.9		16.9		16.9			10.077
7	4	7	32.7		32.7		32.7			54.626
0	0	0	4.7		4.7		4.7		37.98	
2	2	2	17.8		17.8		17.8			13.171
2	2	2	3.9		3.9		3.9		160.55	
2	3	3	5.8		5.8		5.8			86.647
3	4	4	11.8		11.8		11.8			63.091
0	0	0	7.5		7.5		7.5			92.485
1	1	1	5.3		5.3		5.3			57.543
5	4	5	29.8		29.8		29.8			49.419
0	0	0	18.8		18.8		18.8		39.31	
0	0	0	5.7		5.7		5.7		51.88	
13	14	14	40.7		40.7		40.7			48.208
1	1	1	36.3		36.3		9.7			14.558

0	0	0	23.5	23.5	23.5	85.497
0	0	0	9.7	9.7	9.7	86.435
2	2	2	27.7	27.7	27.7	28.708
0	1	1	2.3	2.3	2.3	99.277
3	4	4	49.8	49.8	49.8	28.044
1	1	1	11.4	11.4	11.4	10.206
11	11	11	22.5	22.5	22.5	77.703
2	2	2	29.7	9.1	9.1	21.893
0	0	0	18.9	18.9	18.9	13.387
5	3	5	36.6	36.6	30.1	20.811
0	0	0	2.6	2.6	2.6	244.29
0	0	0	11.6	3.9	0	44.163
0	0	0	6.5	6.5	6.5	90.063
0	0	0	4.4	4.4	4.4	70.861
1	1	1	14.7	14.7	14.7	28.207
0	0	0	0.8	0.8	0.8	180.44
1	1	1	28	28	28	55.053
1	1	1	2.8	2.8	2.8	43.561
0	0	0	2.3	2.3	2.3	161.44
2	2	2	0.9	0.9	0.9	255.45
2	0	0	33.7	7.8	7.8	29.117
12	14	16	15.4	15.4	15.4	208.73
0	0	0	6.9	6.9	6.9	37.785
0	0	0	16.4	16.4	16.4	152.38
3	5	6	5.2	5.2	5.2	226.37
5	7	7	8.1	8.1	7.4	189.92
0	0	0	6.8	6.8	6.8	29.783
2	1	1	10.2	10.2	10.2	25.953
11	12	12	16.4	16.4	16.4	184.64
0	0	0	2	2	2	113.95
1	1	1	1.6	1.6	1.6	94.71
0	0	0	6.9	6.9	6.9	60.307
0	0	0	6.9	6.9	6.9	33.435
1	3	3	5.2	5.2	5.2	87.054
1	0	1	9.1	9.1	9.1	107.07
2	0	0	16.5	16.5	16.5	28.147
0	0	0	12.2	12.2	12.2	24.14
1	1	1	5.1	5.1	5.1	93.293
1	2	2	9.6	9.6	9.6	80.321
5	5	5	22.8	22.8	22.8	28.915
0	0	0	5	5	5	45.285
0	0	0	6	6	6	73.347
2	1	1	53.3	6.9	3.7	57.838
0	0	0	21.9	21.9	21.9	30.972
1	1	1	1.8	1.8	1.8	76.696

2	2	3	18.6	18.6	18.6	81.169
3	3	3	18.9	5.3	5.3	81.167
0	0	0	5.9	5.9	5.9	92.249
1	1	1	70.7	2.5	2.5	97.037
0	0	0	12.3	12.3	12.3	42.299
9	9	9	29.2	29.2	29.2	51.466
0	0	0	6	3	3	57.661
0	0	0	8.1	8.1	8.1	61.521
3	3	6	32.1	29	29	25.542
0	1	1	21.8	6.5	6.5	23.455
0	0	0	2.7	2.7	2.7	58.785
0	0	0	18.2	18.2	18.2	38.496
18	17	19	44.8	44.8	43.6	81.312
0	0	0	14.1	14.1	14.1	38.295
0	0	0	10.6	10.6	10.6	43.614
1	0	1	8.1	8.1	8.1	26.891
9	9	9	66.7	66.7	66.7	18.012
1	1	1	6.1	6.1	6.1	26.616
0	0	0	8.8	8.8	8.8	49.451
4	4	4	42.2	42.2	42.2	24.265
3	4	4	27.7	27.7	27.7	31.994
0	0	0	11.3	11.3	11.3	44.876
3	3	5	4.5	4.5	4.5	170.54
2	2	2	36.5	36.5	36.5	14.077
0	0	0	14.7	14.7	14.7	15.733
5	5	5	34.5	34.5	34.5	29.975
1	1	2	37.1	37.1	37.1	78.891
2	2	3	23.8	23.8	23.8	15.807
12	13	13	28.2	28.2	28.2	77.592
11	9	8	24.2	24.2	24.2	54.231
5	5	5	25.2	25.2	25.2	119.7
2	2	2	4.7	4.7	4.7	52.427
4	3	4	27.2	27.2	21.5	31.865
0	0	0	1.5	1.5	1.5	107.49
0	0	0	4.9	4.9	4.9	80.006
1	1	1	10.5	3.8	3.8	48.084
1	1	1	5	5	5	31.362
1	1	1	18.2	18.2	18.2	68.722
4	4	4	11.2	11.2	11.2	135.58
0	0	1	13.1	13.1	13.1	88.928
0	0	0	14.2	14.2	14.2	40.827
0	1	1	16.1	16.1	16.1	57.875
0	1	1	10.1	6.2	6.2	46.153
9	10	10	36.2	36.2	36.2	48.632
0	0	0	17.2	17.2	17.2	66.689

2	2	2	6.7	6.7	6.7	50.377
1	1	1	5.2	5.2	5.2	32.313
0	0	0	6.9	6.9	5	63.664
2	2	2	13.1	13.1	13.1	35.582
3	2	2	11.8	11.8	11.8	54.958
0	0	0	41.8	6.2	0	53.5
0	0	0	9.9	7.4	7.4	59.241
10	11	12	32.4	31.1	31.1	59.663
0	0	0	9.2	9.2	9.2	16.601
0	0	0	6.1	6.1	6.1	92.547
2	2	2	14.1	14.1	14.1	34.839
10	8	13	21.3	21.3	21.3	87.435
10	8	10	19.4	19.4	19.4	81.613
14	14	14	45.5	45.5	45.5	66.049
2	2	2	4.1	4.1	4.1	101.43
2	1	1	14.9	14.9	14.9	107.97
1	1	1	2.4	2.4	2.4	88.865
0	0	0	1.3	1.3	1.3	110.12
0	0	0	9.3	9.3	9.3	12.523
0	0	0	8.1	8.1	8.1	42.418
0	0	0	8.1	8.1	8.1	68.264
8	7	7	16.3	16.3	15.2	109.46
0	0	0	7.2	7.2	7.2	41.586
1	1	1	13	13	13	17.546
1	2	2	20.3	20.3	20.3	71.013
27	28	33	46.8	46.8	46.8	94.622
1	1	1	2.3	2.3	2.3	63.616
0	0	0	10.5	10.5	10.5	84.993
4	3	4	22.8	22.8	22.8	141.54
1	1	1	3	1.7	1.7	75.028
0	0	0	17.3	15	15	41.918
1	1	1	14.1	14.1	10.2	64.929
0	0	0	10.8	10.8	10.8	12.422
1	1	3	29.7	29.7	29.7	16.06
3	2	3	31.2	31.2	31.2	14.173
0	0	0	13.6	13.6	13.6	19.164
0	0	0	6.4	6.4	6.4	49.858
6	4	5	23.8	23.8	16.5	51.451
1	1	1	10.6	10.6	10.6	33.616
2	2	2	16.4	16.4	16.4	17.789
1	1	1	16.5	16.5	16.5	11.528
7	7	7	51.4	51.4	51.4	27.259
2	3	2	45.6	45.6	45.6	14.463
1	1	1	13.2	13.2	13.2	13.242
4	4	4	51.5	51.5	51.5	11.314

2	0	1	46.4	46.4	46.4	78.409
1	1	1	15.1	15.1	15.1	12.469
11	11	11	40.8	40.8	40.8	28.916
2	2	2	6.8	6.8	6.8	65.585
9	8	9	46.3	46.3	46.3	19.329
0	0	0	2.2	2.2	2.2	62.369
1	1	1	15.2	1.7	1.7	71.003
0	0	0	12.7	12.7	12.7	84.051
11	9	11	28.6	28.6	28.6	65.033
0	0	0	5.9	5.9	5.9	27.236
6	5	6	14.9	14.9	14.9	46.459
21	16	23	35.9	35.9	35.9	90.98
0	0	0	10.8	10.8	10.8	23.155
2	1	1	10.4	10.4	10.4	58.306
1	1	1	11.6	11.6	11.6	26.131
2	2	2	6.4	6.4	6.4	61.492
1	1	1	11.8	11.8	11.8	15.175
3	3	3	27.4	27.4	27.4	68.254
0	0	0	47.9	2.8	0	60.026
0	0	0	1.7	1.7	1.7	90.345
1	1	1	26.6	26.6	26.6	50.602
0	0	0	5.5	5.5	5.5	25.005
0	0	0	2.6	2.6	2.6	59.939
0	0	0	4.4	4.4	4.4	86.692
1	1	2	15.2	15.2	15.2	88.975
0	0	0	2.5	2.5	2.5	52.91
3	4	4	8.4	8.4	8.4	144.47
2	1	2	6.1	6.1	6.1	57.707
1	1	1	34.6	34.6	9.8	33.798
0	0	0	17.9	17.9	17.9	68.386
0	0	0	10.2	10.2	10.2	81.307
1	1	1	17.1	17.1	17.1	18.377
0	0	0	1.6	1.6	1.6	49.91
0	0	0	8.3	8.3	8.3	123.56
2	3	3	8.3	8.3	8.3	97.169
1	1	1	10.1	10.1	10.1	13.369
1	1	0	56	56	2.4	50.394
1	3	3	20.5	20.5	20.5	14.064
1	3	3	10.9	10.9	10.9	51.464
0	0	0	7.8	7.8	7.8	37.481
6	7	7	36.9	36.9	32.9	29.201
1	0	1	22.4	22.4	22.4	16.506
2	1	2	51.9	51.9	20.5	17.905
0	0	0	6.5	6.5	6.5	69.283
15	17	17	32.4	32.4	32.4	117.01

3	2	3	10.6	10.6	10.6	132.22
0	0	0	9.7	9.7	9.7	242.26
0	0	0	9.9	9.9	9.1	267.35
0	0	0	35	35	35	105.53
0	0	0	6.3	6.3	6.3	86.604
0	1	1	11.9	11.9	11.9	13.291
1	2	2	45	45	45	23.772
1	1	1	15	15	15	49.583
1	1	1	38.1	38.1	38.1	11.773
8	7	7	13.5	13.5	13.5	102.42
6	6	6	13.9	13.9	13.9	56.503
15	14	16	19.2	19.2	19.2	143.7
0	0	0	34.3	34.3	34.3	71.992
0	0	0	15.5	6.8	6.8	23.502
3	4	4	9.6	9.6	9.6	84.918
0	0	1	5.7	5.7	5.7	47.93
1	1	1	10.5	10.5	10.5	52.264
1	1	1	39.6	2	2	68.964
0	0	0	5.8	5.8	5.8	57.232
0	0	0	21.4	21.4	18.6	59.674
0	0	0	1.7	1.7	1.7	79.109
0	0	0	3.4	3.4	3.4	39.406
12	12	12	18.6	18.6	18.6	111.77
0	0	0	57.2	10.8	0	46.341
1	0	1	16.4	16.4	16.4	28.089
4	4	5	21.2	21.2	21.2	44.314
0	0	0	5	5	5	82.515
0	0	0	4.9	4.9	4.9	33.528
0	0	0	2.7	2.7	2.7	57.763
2	1	2	6.8	6.8	6.8	58.202
7	6	6	22.6	22.6	22.6	55.409
0	0	0	5.3	5.3	5.3	130.96
0	0	0	1.5	1.5	1.5	82.603
0	0	0	10.7	10.7	10.7	33.128
0	0	0	11.4	11.4	11.4	54.258
0	0	0	12.6	12.6	12.6	102.9
2	2	2	8.1	8.1	8.1	48.884
0	0	0	6.5	6.5	6.5	24.974
1	1	1	3.3	3.3	3.3	44.386
0	1	1	7.5	7.5	7.5	20.308
2	0	0	3.3	3.3	3.3	64.365
3	3	3	7.6	7.6	7.6	51.505
0	0	0	5.8	5.8	5.8	76.917
2	1	2	17.3	17.3	17.3	144.45
0	0	0	7	7	7	87.363

7	6	7	35.5	35.5	35.5	89.225
1	1	1	50	3.7	3.7	37.029
2	2	2	44	44	44	32.616
1	1	1	2.9	2.9	2.9	46.836
1	1	1	7.8	7.8	7.8	18.545
0	0	0	17.8	9.8	0	62.615
0	0	0	20	20	20	15.74
2	2	2	17.8	17.8	17.8	31.739
0	0	0	30.7	30.7	30.7	85.979
0	1	1	19.7	13.7	13.7	20.107
0	0	0	18.1	18.1	18.1	107.6
1	1	2	6.4	6.4	6.4	41.265
1	0	2	26.8	26.8	26.8	19.155
1	0	0	12.9	12.9	12.9	17.605
0	0	0	17.9	11.9	11.9	44.655
6	7	9	16.4	16.4	16.4	78.873
0	0	0	3.5	3.5	3.5	37.01
1	1	1	9.3	9.3	9.3	18.07
0	1	1	2.2	2.2	2.2	96.913
1	1	1	7.6	7.6	3.9	37.143
0	0	0	3.8	3.8	1.7	86.64
1	1	2	7	7	7	48.17
1	3	3	8.1	8.1	8.1	56.177
0	0	0	17.4	17.4	17.4	96.181
7	8	7	16.1	16.1	16.1	82.771
0	0	0	10.3	10.3	10.3	24.559
3	3	3	42.2	42.2	42.2	16.944
1	1	1	6.2	6.2	6.2	29.843
4	3	4	38.1	38.1	38.1	17.456
1	0	0	10.7	10.7	10.7	27.751
3	1	3	13.4	13.4	13.4	39.577
8	8	7	17.2	17.2	17.2	66.99
0	0	0	12.9	12.9	12.9	30.243
0	0	0	25.3	25.3	25.3	98.402
1	1	1	27.1	27.1	19.5	39.076
10	11	12	23.3	23.3	23.3	79.229
0	0	0	19.8	19.8	19.8	29.487
1	1	0	17.4	17.4	17.4	40.483
0	0	0	6.7	6.7	6.7	71.284
0	0	0	7.4	7.4	7.4	24.116
0	0	0	13.7	13.7	13.7	24.38
0	0	0	9	9	9	40.42
0	1	1	23.4	23.4	23.4	51.861
0	0	0	4.7	4.7	4.7	29.649
0	0	0	3.6	3.6	3.6	49.562

0	0	0	5.4	5.4	5.4	66.976
2	2	2	3.5	3.5	3.5	82.021
0	0	0	12.8	12.8	12.8	87.991
1	1	1	14.9	14.9	14.9	96.306
0	0	0	5.8	5.8	5.8	28.396
3	2	3	13	13	13	33.572
2	2	3	16	9.5	9.5	52.064
3	3	3	8.3	8.3	8.3	49.019
2	2	2	23.4	23.4	23.4	106.38
0	0	0	14.2	14.2	14.2	41.764
5	5	6	16.2	16.2	16.2	71.24
1	1	0	19.3	19.3	19.3	35.936
2	2	2	24.2	24.2	24.2	14.731
0	0	0	7.3	7.3	7.3	25.683
0	0	0	5.7	5.7	5.7	25.938
1	2	2	7.8	7.8	7.8	50.159
0	0	0	68.4	68.4	68.4	21.315
6	6	6	35.2	35.2	35.2	38.91
3	4	3	48.2	48.2	48.2	17.822
0	0	0	6.9	6.9	6.9	28.062
0	0	0	7	7	7	102.57
1	1	1	8.2	8.2	8.2	15.823
0	0	0	1.5	1.5	1.5	134.26
0	0	0	23.5	23.5	23.5	113.56
3	2	4	23.6	23.6	14.1	116.77
1	1	1	8.1	8.1	8.1	104.65
4	4	4	17.1	17.1	17.1	46.15
2	3	3	18.8	18.8	18.8	91.985
6	6	6	20.1	20.1	20.1	63.261
9	10	10	41.1	41.1	41.1	46.089
11	8	10	22.7	22.7	22.7	89.546
1	1	1	15.8	15.8	15.8	24.953
1	1	1	6.8	6.8	6.8	21.319
0	0	0	18.1	1.3	1.3	108.2
0	0	0	5	5	5	127.4
2	1	2	6.4	6.4	6.4	45.439
0	0	0	3.4	3.4	3.4	85.503
1	1	1	4.9	4.9	4.9	25.486
2	2	2	16.6	7.3	7.3	55.102
7	7	8	50	50	50	50.227
1	1	1	9.8	9.8	9.8	46.744
0	0	0	4.5	4.5	4.5	72.131
0	0	0	11.9	11.9	11.9	12.239
1	1	1	17.1	17.1	17.1	92.467
1	1	1	3.3	3.3	3.3	51.618

1	1	1	16.7	16.7	16.7	14.363
0	0	0	7.9	7.9	7.9	62.286
0	0	1	8	8	8	22.596
2	3	3	20.2	18	18	54.653
8	7	8	44.2	43.2	43.2	72.4
0	0	0	8.4	8.4	8.4	18.059
0	0	0	10.7	10.7	10.7	54.158
0	0	0	33.2	4.7	4.7	38.579
4	5	4	38.6	38.6	38.6	16.29
0	0	0	7.1	7.1	7.1	143.97
0	0	0	10.2	10.2	10.2	105.55
3	4	4	12.8	12.8	12.8	91.43
1	2	2	8.9	8.9	8.9	145.42
0	0	0	4	4	4	179.04
2	2	1	7.2	7.2	7.2	40.68
0	0	0	5.3	5.3	5.3	112.99
0	0	0	1.3	1.3	1.3	137.46
0	0	0	7	7	7	121.41
0	0	0	7.4	1.5	1.5	70.991
0	0	0	10.6	10.6	10.6	149.32
0	0	0	16.9	16.9	13.6	326.02
0	0	1	29.2	29.2	29.2	12.527
4	4	4	12.2	12.2	12.2	90.725
1	1	1	6.1	6.1	6.1	26.714
0	0	0	9.1	9.1	7.5	315.13
2	2	3	6.3	6.3	4.3	56.788
1	1	1	2.2	2.2	2.2	45.513
0	0	0	33.7	33.7	33.7	10.053
0	0	0	6.5	6.5	6.5	19.389
1	1	1	16.4	16.4	16.4	26.228
1	1	1	11.4	11.4	11.4	11.687
1	1	1	44.1	44.1	44.1	7.624
0	0	0	19.5	19.5	19.5	19.363
1	1	1	5.4	5.4	5.4	23.241
0	0	0	46	46	46	69.151
1	0	1	8	8	8	20.161
2	3	3	30	30	30	99.328
1	1	1	9.7	9.7	9.7	11.905
2	2	2	18.8	18.8	18.8	11.333
0	0	0	32.7	32.7	32.7	57.044
2	2	2	41.3	41.3	41.3	14.369
5	4	5	24.5	24.5	24.5	39.584
1	1	1	10.9	10.9	10.9	15.324
3	2	3	39.9	39.9	39.9	36.877
0	0	1	7.2	7.2	7.2	92.387

1	0	0	34.4		13.5		2.4			47.117
2	0	0	6.2		4.6		4.6			51.152
2	2	3	1.4		1.4		1.4		192.99	
0	0	0		16	3.5		1.8			63.165
3	0	0	18.4		4.5		4.5			52.247
1	0	0	19.4			2		2	59.56	
10	10	10	51.5		51.5		51.5			24.409
11	5	9	67.8		62.5			39		51.561
0	1	0	16.5			3		3		57.769
3	8	5	46.7		46.7		46.7			22.975
4	6	3	25.1		25.1		25.1			24.348
2	3	3	23.9		23.9		23.9			23.583
2	5	2	49.1		49.1		49.1			18.974
3	7	5	40.7		40.7		40.7			18.281
80	80	80	87.5		87.5		87.5			69.293
0	0	0	10.4		10.4		10.4			24.241
0	0	0	50.5		39.9			0		59.998
4	4	4	34.6		25.5		25.5			53.704
43	42	44	60.2		60.2		60.2			91.215
17	7	10	62.8		43.1		43.1			51.267
25	24	25	62.7		62.7		50.9			58.826
2	2	2	46.6			28	4.4			62.378
1	1	1	2.6		2.6		2.6			38.252
1	1	1	5.6		5.6		5.6			34.084
26	25	25	67.6		67.6		67.6			62.064
0	0	0	79.2		5.6		5.6			65.865
3	0	0	39.3		10.5		6.7			55.802
5	4	4	45.6		26.2			19		48.105
0	0	0	37.3		35.6			0		47.237
2	2	2	12.4		12.4		12.4			65.056
0	1	1	6.4		6.4		6.4			24.536
1	1	1	0.8		0.8		0.8		187.37	
1	0	0	14.6		9.4			5		58.923
0	0	0	28.8		23.5		18.8			51.385
0	0	0	9.5		9.5		9.5		22.06	
1	0	0		31	6.8		4.2			50.529
3	3	3	5.4		5.4		5.4		248.07	
0	0	0	16.1		1.9			0		57.835
0	0	0	35.6		1.5			0		62.936
3	1	0	33.8		9.6		7.7			52.809
1	0	0	9.3		2.3		2.3			48.139
2	2	2	6.9		6.9		6.9			50.525
1	0	1	42.2		40.8		2.4			54.195
1	2	2		16	11.2		11.2			56.865
0	0	1	6.7		2.7		2.7			49.317

1	1	1	19.7	16.1	10.7	61.901
7	6	7	14.3	14.3	14.3	282.39
3	1	0	21.1	21.1	21.1	12.957
1	1	0	11.5	3.7	3.7	56.652
0	0	0	41.4	41.4	41.4	28.106
1	0	0	13.2	13.2	13.2	11.964
2	0	1	27.1	2.5	2.5	53.728
10	10	10	25.6	25.6	25.6	73.708
0	0	0	16.2	16.2	16.2	75.667
0	0	0	4.4	4.4	4.4	84.105
0	0	0	1.2	1.2	1.2	110.26
0	0	1	4.1	4.1	4.1	178.57
1	1	1	6.3	6.3	6.3	28.615
3	1	2	25.6	25.6	25.6	24.561
5	5	5	14.3	14.3	14.3	57.215
0	0	0	27.6	27.6	27.6	15.619
2	2	2	2.9	2.9	2.9	151.98
2	3	4	17	17	17	36.471
2	2	2	9.7	9.7	9.7	32.518
2	1	1	9.9	9.9	9.9	32.932
0	0	0	11.6	11.6	11.6	57.406
3	3	3	7.2	7.2	7.2	78.635
3	2	2	6	6	6	106.92
0	0	0	3	3	3	292.74
19	18	19	26.5	26.5	26.5	107.57
4	6	7	33.7	33.7	33.7	34.389
0	0	0	13.5	13.5	13.5	9.632
1	1	1	26.4	26.4	26.4	18.658
1	0	0	15.9	15.9	15.9	14.719
1	2	2	13.3	13.3	13.3	15.164
0	0	0	5.6	5.6	5.6	65.885
0	0	0	11.1	6.7	1.4	79.372
8	8	8	11.8	11.8	11.8	118.25
1	1	1	14.9	0.8	0.8	152.3
3	3	4	27.5	27.5	27.5	45.28
2	2	3	4.7	4.7	4.7	121
1	1	1	5	5	5	44.932
1	1	2	10.7	10.7	10.7	86.979
2	2	2	22.5	22.5	22.5	28.549
0	0	0	4.7	4.7	4.7	41.09
2	2	2	17.8	17.8	17.8	37.709
1	0	1	28	28	28	58.668
2	2	2	43.4	43.4	43.4	12.015
0	0	0	27.5	27.5	27.5	11.158
1	1	1	16.9	16.9	16.9	66.478

0	0	0	22	22	11.6	45.571
0	0	0	18	6.5	6.5	41.627
1	1	1	6.1	1.7	1.7	57.066
8	7	9	22.5	22.5	22.5	72.456
1	2	2	3	3	3	436.13
1	1	1	29.5	29.5	29.5	47.446
0	0	0	16.2	16.2	16.2	16.436
2	2	2	34.2	34.2	34.2	64.872
0	0	0	5.6	5.6	5.6	192.09
2	2	2	6.4	6.4	6.4	107.3
0	0	0	16.1	16.1	16.1	13.743
0	0	0	53	12.2	9.1	49.895
1	1	1	8.9	8.9	8.9	15.397
0	0	0	1.9	1.9	1.9	236.02
1	1	1	1.2	1.2	1.2	207.81
0	0	1	10.7	10.7	10.7	14.918
17	17	19	29.4	29.4	29.4	142.6
2	3	3	33.6	17.9	17.9	31.6
0	0	0	8.9	8.9	8.9	16.29
2	2	2	33.8	33.8	33.8	44.682
6	4	7	17	17	17	83.515
0	0	0	18.4	18.4	18.4	11.132
8	9	9	10.4	10.4	10.4	150.83
1	1	1	13.2	13.2	13.2	68.349
0	0	0	5.9	5.9	5.9	52.679
0	0	0	14.7	14.7	14.7	84.285
9	10	10	28.7	28.7	28.7	91.48
0	0	0	42.7	42.7	42.7	14.057
1	1	1	22.3	2.6	2.6	45.321
0	0	0	21.1	21.1	21.1	25.139
10	10	11	53	53	35	49.229
1	1	1	11.4	4.3	4.3	29.174
0	0	0	7.9	7.9	7.9	45.112
0	0	0	24.1	24.1	3	64.243
0	0	0	11.9	11.9	11.9	50.957
5	5	5	31	31	31	23.864
0	0	0	5.2	5.2	5.2	30.274
1	0	2	7	7	7	47.945
6	7	8	60	60	60	21.154
0	0	0	46.9	4.1	4.1	21.855
0	0	0	1.1	1.1	1.1	178.14
4	4	4	45.3	45.3	45.3	15.747
0	1	1	28.8	28.8	28.8	11.986
10	9	11	30.3	30.3	30.3	56.648
19	20	20	47.5	37.4	37.4	80.272

1	0	0	0.2	0.2	0.2	838.3
0	0	0	9.8	9.8	9.8	14.619
1	1	1	21	21	21	17.793
0	0	0	5.3	1.4	1.4	127.14
0	0	0	16.4	16.4	16.4	70.38
0	0	0	54.5	54.5	0	66.038
0	0	0	2.7	2.7	2.7	98.008
2	4	3	17	17	17	109.13
2	2	2	18.8	18.8	6.7	47.33
2	2	2	45.6	21.1	21.1	18.639
0	0	0	22.4	1.8	0	159.91
8	8	9	41.2	41.2	41.2	34.852
1	1	1	4.4	4.4	4.4	25.514
1	1	1	6.9	6.9	6.9	85.676
0	0	0	23.7	10.7	0	30.159
0	0	0	17.5	17.5	17	192.19
4	4	4	30.5	30.5	30.5	11.737
0	0	0	6	6	6	24.706
0	0	0	13.9	13.9	13.9	32.182
1	1	1	12.4	12.4	12.4	23.468
0	1	1	4.1	4.1	4.1	41.461
3	3	3	40	40	30	14.563
1	1	1	12.9	12.9	12.9	13.261
6	6	6	55	51.5	51.5	22.187
4	4	4	11.3	11.3	11.3	130.66
1	1	1	7.8	7.8	7.8	12.476
1	1	1	18.8	10.5	10.5	15.041
2	2	2	40.5	40.5	40.5	16.733
0	0	0	3.3	3.3	3.3	33.747
1	1	1	5.8	5.8	5.8	53.027
1	0	0	5.1	5.1	5.1	17.791
3	3	3	26.8	26.8	26.8	65.539
22	23	25	52.4	52.4	40.6	69.147
2	2	2	19.7	19.7	19.7	20.819
12	12	12	47.2	47.2	44.4	27.744
2	2	2	7.2	7.2	7.2	103.7
0	0	0	3.9	3.9	3.9	62.101
1	1	1	4.5	4.5	4.5	84.699
5	5	7	18.2	18.2	18.2	50.75
8	8	8	24.8	24.8	23.4	55.793
0	0	0	13.1	9.5	9.5	69.948
0	0	0	13.2	13.2	13.2	19.264
0	0	0	51.2	51.2	51.2	19.001
4	4	4	19	19	19	43.312
2	2	2	26.6	26.6	26.6	17.91

0	0	0	3.2	3.2	3.2	34.488
1	1	1	4	4	4	44.638
1	1	1	13.2	13.2	13.2	22.447
0	0	0	18.2	18.2	18.2	20.903
0	0	0	8.6	8.6	8.6	27.607
0	0	0	3.9	3.9	1.9	68.371
2	2	2	20	20	20	12.069
10	9	10	47.2	47.2	47.2	103.28
0	0	0	9	9	9	39.443
1	0	1	18.3	18.3	18.3	21.256
1	1	1	7.9	7.9	7.9	14.301
0	0	0	0.7	0.7	0.7	265.61
2	2	2	37.7	37.7	37.7	24.942
0	0	0	24.4	24.4	24.4	31.62
0	1	2	47.5	47.5	47.5	61.367
0	0	0	7.2	7.2	7.2	21.671
2	2	3	8.6	8.1	8.1	196.69
0	0	0	0.9	0.9	0.9	593.38
2	3	3	16	16	16	123.9
10	10	10	53.8	53.8	53.8	45.809
1	1	1	9.1	9.1	9.1	123.38
0	0	0	12.9	12.9	12.9	74.277
0	1	1	12.4	12.4	12.4	38.461
2	2	2	19	19	19	116.92
0	0	0	2.8	2.8	2.8	48.847
9	9	9	28.5	28.5	28.5	50.714
0	0	0	5.1	5.1	5.1	56.65
2	1	3	16	16	16	90.254
12	10	10	33.6	33.6	27.3	70.942
2	2	2	6.4	6.4	6.4	54.67
2	2	2	12.3	12.3	12.3	57.193
10	8	12	47.3	47.3	47.3	37.154
1	0	1	37.4	37.4	37.4	76.107
5	5	5	3.8	3.8	3.8	254.39
0	0	0	4.2	4.2	4.2	25.76
8	7	9	39.3	30.5	30.5	50.65
5	7	7	30.2	30.2	30.2	57.673
0	0	0	5.1	5.1	5.1	130.32
0	0	0	11.1	11.1	11.1	133.06
1	2	2	16.7	16.7	16.7	38.938
2	2	2	16.2	16.2	16.2	21.896
2	1	2	16.9	16.9	16.9	39.617
6	7	7	10.1	10.1	10.1	108.8
1	1	1	17.7	17.7	17.7	23.306
6	8	8	18.3	18.3	16.8	60.103

4	3	3	29.1	29.1	29.1	20.913
6	8	9	14.3	14.3	14.3	145.83
3	3	2	29.2	29.2	29.2	19.343
3	4	4	7.7	7.7	7.7	244.5
1	1	1	7.5	7.5	7.5	318.38
5	5	6	43.3	43.3	43.3	26.711
1	1	2	18.8	18.8	18.8	26.825
0	0	0	0.9	0.9	0.9	200.55
0	0	0	2.5	2.5	2.5	100.05
2	2	2	15.5	15.5	15.5	106.92
0	0	0	7.1	7.1	7.1	309.35
5	0	1	28.8	28.8	28.8	22.255
6	8	9	25.5	25.5	25.5	100.28
1	1	1	5.4	5.4	5.4	37.517
9	10	12	18.8	18.8	18.8	139.88
7	8	8	22.2	22.2	22.2	66.05
1	0	1	2	2	2	111.42
0	0	0	2.4	2.4	2.4	61.438
1	1	1	1.1	1.1	1.1	137.42
0	0	0	11.9	11.9	11.9	30.255
0	0	0	2.6	2.6	2.6	61.603
0	0	0	7.3	7.3	7.3	66.9
3	3	3	27.3	27.3	27.3	47.38
5	6	5	9.5	9.5	9.5	152.26
0	0	0	10.1	10.1	10.1	24.588
34	33	34	69.9	69.9	68.7	74.139
3	3	3	33.3	33.3	33.3	14.326
0	0	0	54.7	4.1	0	49.585
6	5	6	29	29	29	36.053
2	2	2	37.1	37.1	37.1	22.782
1	1	0	24.9	24.9	24.9	68.606
0	0	0	12.2	12.2	12.2	112.89
1	1	1	11.8	11.8	11.8	10.834
5	6	6	40.9	40.9	19.8	32.852
3	3	3	19.8	19.8	19.8	48.113
12	12	12	37.1	37.1	37.1	32.575
2	2	3	64.6	20.4	16.9	47.766
0	0	0	6.8	6.8	6.8	170.59
0	0	0	21.2	21.2	21.2	99.533
2	2	2	9.9	9.9	9.9	80.692
1	1	3	56.9	55.4	53.4	53.651
7	7	7	65.9	65.9	65.9	14.716
19	15	18	61.6	61.6	61.6	28.415
0	0	1	31.2	24.2	24.2	13.509
1	1	2	3.3	3.3	3.3	64.596

0	0	0	5.5	5.5	5.5	31.054
10	9	9	58.7	58.7	57.6	72.332
12	13	14	55.7	54.6	46.7	70.897
13	12	11	27.4	27.4	21.9	174.38
0	0	0	13.4	13.4	13.4	17.776
3	2	3	38.6	17.4	17.4	32.866
2	0	1	15.1	15.1	15.1	16.572
2	2	2	10.6	10.6	10.6	64.243
5	5	5	15.5	14.3	14.3	95.337
20	18	18	65.9	65.9	64.7	61.926
50	42	42	26.3	26.3	26.3	331.77
8	8	7	27.9	24.8	24.8	22.58
2	3	3	31	31	9.9	21.364
0	0	0	9.7	9.7	9.7	114.76
1	1	1	11.5	11.5	11.5	35.879
2	2	2	14.1	14.1	14.1	35.173
0	0	0	20.9	20.9	20.9	97.801
6	6	6	27.4	27.4	27.4	66.69
0	0	0	21	21	21	60.343
3	2	3	30	30	30	12.538
1	1	1	20.4	20.4	6.4	263.83
0	0	0	12	12	12	41.213
11	10	12	51.2	49.7	47.4	66.408
10	9	9	14.4	14.4	14.4	276.55
1	0	1	7.1	7.1	7.1	31.629
6	6	6	51.5	51.5	47.3	28.449
0	0	0	6.9	6.9	5.7	104.76
5	3	4	17	17	17	68.63
1	1	1	11.8	11.8	11.8	51.169
4	3	2	24	24	24	22.742
4	5	6	54.7	54.7	54.7	26.688
4	4	4	64.5	64.5	64.5	18.502
3	3	3	47	47	47	14.515
5	4	5	15.9	15.9	15.9	59.75
9	7	9	23.2	23.2	23.2	53.5
1	1	1	10.9	10.9	10.9	11.728
12	12	13	46	46	42.6	57.221
8	9	10	51.9	51.9	51.9	24.604
0	0	0	12.3	12.3	12.3	67.567
2	1	2	14.8	14.8	14.8	56.921
1	1	0	25	25	25	73.243
11	13	13	56.7	56.7	56.7	32.868
4	5	5	54.5	54.5	54.5	17.818
2	2	2	11.9	11.9	11.9	11.471
0	0	0	17.9	17.9	17.9	35.378

0	0	0	53.1	53.1	53.1	44.868
2	1	1	15.3	15.3	8.1	27.774
2	3	3	47.9	47.9	32.9	16.185
0	0	0	16.1	16.1	16.1	97.071
0	0	0	42.6	37.1	37.1	36.949
4	2	4	25.7	25.7	25.7	39.157
4	4	5	15	15	15	128.25
12	12	12	28.3	28.3	28.3	42.674
1	1	1	79	79	4.7	65.432
0	0	0	21	21	21	32.247
9	10	10	46.8	46.8	46.8	47.697
5	6	6	57.4	57.4	57.4	14.57
9	11	11	33.2	33.2	33.2	42.331
11	8	11	30.9	30.9	30.9	62.608
8	8	9	41.9	41.9	41.9	46.108
2	2	2	3.1	3.1	3.1	164.19
5	6	6	29.1	29.1	29.1	23.558
0	0	0	6.4	6.4	6.4	57.001
0	0	0	9.9	9.9	9.9	19.738
17	17	18	52.6	52.6	35.2	75.491
1	1	1	10.7	10.7	10.7	117.8
1	1	1	25	25	25	9.461
0	0	0	14.4	14.4	14.4	168.89
7	8	9	22.9	22.9	22.9	104.74
10	10	12	47.9	47.9	47.9	33.777
2	2	2	20.6	20.6	20.6	18.565
2	2	2	19.4	19.4	19.4	18.898
0	0	0	12.9	12.9	12.9	37.251
0	0	0	21	21	21	19.365
0	0	0	6.7	5.1	5.1	83.529
1	0	1	2.6	2.6	2.6	273.42
0	0	0	21.7	21.7	21.7	49.541
0	0	0	1.1	1.1	1.1	367.76
4	4	4	41.9	41.9	41.9	10.112
2	2	2	17.3	5.3	5.3	47.315
1	1	1	1.2	1.2	1.2	82.785
1	1	2	5.9	5.9	5.9	219.98
8	10	10	21.1	21.1	21.1	100.18
28	29	30	16.3	16.3	16.3	358.2
1	2	2	64.6	64.6	64.6	28.994
0	0	0	33.3	33.3	33.3	53.239
0	0	0	26	26	26	18.998
0	0	0	14.2	14.2	14.2	384.2
4	4	5	29.6	26.2	26.2	26.788
2	2	2	51.6	51.6	8.2	39.594

15	16	19	61.8	61.8	61.8	77.515
2	2	2	15.4	15.4	15.4	18.001
0	0	0	18.8	18.8	18.8	152.78
0	0	0	27.2	27.2	27.2	34.917
0	0	0	1.2	1.2	1.2	138.34
4	3	3	26.8	26.8	26.8	14.395
0	0	0	2.1	2.1	2.1	75.378
1	1	1	24.3	24.3	24.3	11.186
0	0	0	20.6	20.6	20.6	89.321
3	3	3	6.8	6.8	6.8	51.958
1	1	1	34.5	34.5	1.5	136.06
6	6	7	38.5	24.3	24.3	49.263
3	3	5	42.9	42.9	42.9	26.599
0	0	0	2.5	2.5	2.5	106.37
2	2	1	10.4	7.4	7.4	38.772
2	1	1	19.1	19.1	19.1	10.245
0	0	0	11.5	11.5	11.5	99.743
1	1	0	63.2	4.5	4.5	41.722
3	3	3	28.6	28.6	28.6	13.373
3	2	2	42.1	42.1	42.1	17.244
3	2	2	43.4	43.4	43.4	36.588
1	1	1	25.5	7.6	7.6	37.512
7	7	7	51.4	51.4	51.4	24.205
2	2	2	41.1	41.1	41.1	16.273
2	4	3	51.3	51.3	51.3	17.718
1	1	1	19.6	19.6	19.6	66.767
7	6	6	52.5	52.5	52.5	18.431
2	1	2	24.4	24.4	24.4	97.251
2	1	2	20.2	20.2	20.2	13.281
3	3	3	37.3	37.3	37.3	13.527
3	3	2	31.7	31.7	31.7	13.916
6	7	7	42.1	42.1	42.1	29.995
5	5	5	32.2	32.2	32.2	19.463
3	3	3	44.9	44.9	44.9	17.695
0	0	0	22.4	22.4	22.4	13.742
0	0	0	7.4	7.4	7.4	12.274
0	1	1	19.6	19.6	19.6	63.225
6	6	6	31.3	31.3	31.3	24.831
7	6	6	47.8	47.8	47.8	20.252
6	5	6	40.1	40.1	40.1	28.024
1	1	1	46.1	7.8	7.8	14.728
6	7	7	27.9	27.9	27.9	44.25
0	0	0	9.6	9.6	9.6	51.647
4	5	5	51.7	51.7	51.7	10.366
2	1	1	23.5	23.5	23.5	8.85

1	1	1	63.2	63.2	4.5	41.792
1	1	1	32.4	32.4	32.4	35.924
5	5	5	58.2	58.2	35.7	50.184
2	2	2	55.1	55.1	12.1	49.924
0	0	0	65.8	65.8	0	49.839
0	0	0	11.3	11.3	11.3	18.957
2	2	2	36.2	36.2	36.2	29.321
1	1	2	23.9	23.9	23.9	39.478
0	0	0	5.4	5.4	5.4	54.444
69	69	81	34.2	34.2	34.2	469.08
1	1	1	9.3	9.3	9.3	22.693
2	2	2	22.7	22.7	22.7	11.284
4	4	3	22.9	22.9	22.9	23.671
18	18	19	32.6	32.6	32.6	90.583
10	10	9	45.4	45.4	45.4	27.872
0	0	0	22.3	22.3	22.3	48.736
1	1	1	9.9	9.9	9.9	55.01
2	1	1	8.9	8.9	8.9	33.091
6	5	5	12	12	12	100.83
5	5	4	12.4	12.4	12.4	113.75
15	16	17	25.3	19.7	19.7	180.61
2	2	2	10.9	10.9	10.8	431.76
2	2	2	14.2	14.2	14.2	24.847
0	0	0	42.5	20.1	20.1	50.47
5	4	5	28.1	28.1	28.1	48.817
2	2	3	12.6	12.6	12.6	55.637
3	4	4	57.6	47.5	47.5	42.429
12	12	12	68.3	68.3	56.8	22.11
2	1	1	9.6	9.6	9.6	18.184
9	6	8	9.6	9.6	9.6	140.96
3	2	2	3.9	3.9	3.9	93.834
3	2	3	34.1	34.1	25.2	47.655
1	1	1	6.9	6.9	6.9	629.09
6	6	7	12.1	12.1	12.1	238.8
0	0	0	9.9	9.9	9.9	338.26
2	3	3	3.5	3.5	3.5	104.82
2	2	2	6.2	3.4	3.4	226.59
0	0	0	11.3	11.3	8.8	45.993
5	5	5	30.5	30.5	30.5	30.84
0	0	0	30.9	30.9	30.9	88.549
14	16	16	30.2	30.2	30.2	97.322
2	2	2	15.7	15.7	1.6	152.1
0	0	0	43.8	18.2	10.4	50.432
0	0	0	3.5	3.5	3.5	301.36
6	7	7	23.3	23.3	23.3	61.494

5	4	4	25.9	22.3	22.3	32.688
5	4	5	4.5	4.5	4.5	129.57
0	0	0	21.2	21.2	21.2	44.355
1	2	1	21.6	21.6	21.6	83.654
0	0	0	50.8	6.1	3.4	49.953
7	6	7	19.5	19.5	9.3	107.47
0	0	0	22.1	22.1	22.1	36.876
3	2	3	9.4	9.4	9.4	70.973
4	3	4	18.2	18.2	18.2	29.172
0	0	0	2.1	2.1	2.1	124.98
16	18	18	43.6	43.6	43.6	59.379
1	0	0	15.2	2.5	2.5	50.343
10	9	11	16.4	16.4	12.1	220.43
0	0	0	0.5	0.5	0.5	233.17
11	11	11	10.9	10.9	10.9	226.66
0	0	0	4.2	4.2	4.2	68.259
4	4	5	27	27	27	143.23
23	23	24	41.7	41.7	41.7	84.427
5	5	5	4.9	4.9	4.9	208.7
0	1	1	2	2	2	145.81
0	0	0	67.1	67.1	67.1	56.08
13	11	14	42.4	42.4	42.4	73.261
0	0	0	5.2	5.2	5.2	132.37
5	4	6	15.2	15.2	15.2	220.62
0	1	0	67.9	0.6	0.6	238.26
2	1	2	7.7	7.7	7.7	41.193
0	0	0	0.7	0.7	0.7	186.49
1	1	1	1.6	1.6	1.6	74.89
0	0	0	21.2	21.2	21.2	33.307
1	0	0	4.9	4.9	3.9	531.78
7	7	6	59	59	45.8	37.497
1	1	1	6.5	6.5	6.5	50.287
17	18	17	54.9	54.9	54.9	36.091
2	4	4	35.3	29.4	29.4	47.82
4	5	5	16.8	16.8	16.8	51.588
2	2	2	11.6	11.6	11.6	85.104
0	0	0	6.9	6.9	6.9	153.51
4	4	4	34.8	15.7	15.7	38.405
0	0	0	37.9	37.9	37.9	189.97
6	8	8	13.9	13.9	13.9	91.679
2	0	0	7.4	7.4	7.4	17.085
0	0	0	24.5	7.4	7.4	49.775
3	3	3	8	6	6	80.107
1	0	0	7	7	7	18.32
0	0	0	2.2	2.2	2.2	69.221

2	2	3	7.6	7.6	7.6	60.032
0	0	0	3.4	3.4	3.4	50.004
1	1	1	36.5	36.5	36.5	38.072
0	0	0	16.6	16.6	16.6	29.702
3	3	4	17.8	17.8	17.8	68.306
3	2	2	18.7	12.9	12.9	122.87
1	1	1	33.9	1.4	1.4	137.83
0	0	0	36.4	1.4	0	77.495
8	9	10	68.9	68.9	68.9	22.826
8	7	9	32.5	32.5	32.5	65.653
0	0	0	12.2	12.2	12.2	96.256
1	0	1	11.8	11.8	11.8	17.224
0	0	0	10.6	0.8	0.8	182.52
0	0	0	9.7	9.7	9.7	84.468
3	3	4	25	25	25	28.235
0	0	0	5.4	5.4	5.4	72.235
0	0	0	2.9	2.9	2.9	196.04
3	5	5	16.2	16.2	16.2	36.514
0	0	0	12.5	12.5	12.5	128.72
1	2	3	24.6	24.6	24.6	79.322
6	6	7	31.6	31.6	31.6	42.008
2	2	2	37.7	37.7	37.7	50.381
0	0	0	5.3	5.3	5.3	84.567
1	1	1	4.2	4.2	4.2	38.155
0	0	0	5.1	5.1	5.1	94.474
0	0	0	3.6	3.6	3.6	96.122
4	6	5	30.7	30.7	30.7	64.135
2	2	2	3.6	3.6	3.6	26.238
0	0	1	7.9	7.9	7.9	20.713
2	2	2	2.2	2.2	2.2	106.1
1	1	1	19.9	19.9	8.1	15.43
1	2	2	7.2	7.2	7.2	168.35
0	0	0	5.3	5.3	5.3	237.3
17	18	17	29.8	29.8	29.8	274.46
7	8	7	13.6	13.6	13.6	64.467
0	0	0	3.9	3.9	3.9	251.11
0	0	0	2.2	2.2	2.2	65.111
0	0	1	11.3	11.3	11.3	268.84
0	0	0	11.3	11.3	11.3	172.79
3	3	3	9.2	9.2	9.2	44.961
0	0	0	15.7	15.7	15.7	81.116
0	0	0	3	3	3	273.43
0	0	0	4.8	4.8	4.8	91.173
26	24	25	49.1	49.1	49.1	85.652
1	1	1	5.5	5.5	5.5	197.39

1	1	1	45.8	45.8	45.8	112.68
0	0	0	7.1	7.1	7.1	49.192
1	2	2	12.4	12.4	12.4	57.563
1	1	1	4.7	4.7	4.7	49.741
0	0	0	13.8	13.8	13.8	64.551
0	0	0	23.8	23.8	23.8	78.576
0	0	0	7.6	7.6	7.6	129.08
8	8	8	8	8	8	273.6
0	0	0	8.5	8.5	8.5	113.86
0	0	0	3.4	3.4	3.4	50.501
0	0	0	19	19	19	42.441
0	0	0	1	1	1	297.91
1	1	1	18.2	18.2	18.2	66.526
2	2	2	15.3	15.3	15.3	60.54
0	0	0	6.6	6.6	6.6	15.513
1	1	1	3.7	3.7	3.7	42.692
1	1	1	52.6	4.1	4.1	46.132
0	0	0	7	7	7	32.571
4	3	4	16.2	16.2	16.2	53.262
1	1	1	8.8	8.8	8.8	50.559
0	0	0	8.4	8.4	8.4	210.85
1	1	1	4.5	4.5	4.5	302.88
1	0	0	3	3	3	155.34
0	0	0	19.7	19.7	7	141.47
2	2	2	5.5	5.5	5.5	203.35
2	2	3	2.1	2.1	2.1	201.56
0	0	0	0.2	0.2	0.2	448.66
0	0	0	4.3	4.3	4.3	29.714
3	1	0	4	4	4	113.82
0	0	0	4.7	2.3	0	74.375
0	0	0	5.7	5.7	5.7	48.47
2	2	2	18	18	18	32.749
3	3	3	15.9	15.9	15.9	192.95
6	7	7	28	28	28	37.974
4	4	4	40.1	40.1	40.1	26.888
6	6	6	39.7	39.7	39.7	22.936
0	0	0	5.4	5.4	5.4	131.29
2	2	2	5.2	5.2	5.2	52.8
4	4	4	24.2	24.2	24.2	75.356
0	0	0	66.1	4	4	49.752
0	0	0	1.9	1.9	1.9	269.45
5	6	6	38.8	38.8	10.2	91.079
0	0	0	24.4	24.4	24.4	19.901
3	2	3	13	13	13	96.557
0	1	1	4.1	4.1	4.1	102.02

0	0	0	16.9	16.9	16.9	25.977
0	0	0	4.5	4.5	4.5	409.8
1	0	0	50.9	14.5	14.5	62.852
1	0	1	13.1	13.1	13.1	93.487
5	4	5	11.9	11.9	11.9	80.724
1	2	2	19.5	19.5	19.5	47.138
0	0	0	11.7	11.7	11.7	17.631
0	0	0	6.5	6.5	6.5	59.823
0	0	0	39.4	39.4	12.9	13.903
0	0	0	7.7	5.1	0	67.214
1	1	1	8.9	8.9	8.9	60.509
2	2	2	6.4	6.4	6.4	37.476
4	4	4	10.4	10.4	10.4	54.478
0	0	0	2.1	2.1	2.1	92.097
1	1	1	5	5	5	97.903
2	2	2	11.7	11.7	11.7	43.806
4	4	5	17.4	17.4	17.4	44.965
0	0	0	7.5	7.5	7.5	83.538
6	5	6	17.8	17.8	17.8	77.193
0	0	0	27.4	24.9	24.9	106.16
4	3	5	35.8	35.8	35.8	21.857
0	0	0	1.8	1.8	1.8	73.084
1	1	1	1.6	1.6	1.6	330.46
0	0	0	18.1	18.1	18.1	265.39
0	0	0	11.5	9.4	8.5	305.41
6	5	6	10.6	10.6	10.6	98.594
7	7	7	42.5	42.5	42.5	41.401
1	0	1	0.3	0.3	0.3	529.01
0	0	0	2.3	2.3	2.3	205.11
1	1	2	4.3	4.3	4.3	100.68
0	0	0	19.1	19.1	19.1	51.289
0	0	0	18.2	18.2	18.2	28.69
18	17	18	22.7	22.7	21.9	150.56
2	2	3	15.3	15.3	15.3	65.26
9	11	10	42.8	42.8	42.8	252.5
2	2	2	4	4	4	47.863
4	4	4	17.8	17.8	17.8	22.487
11	14	15	19.9	19.9	19.9	229.48
0	0	0	28.3	28.3	19.1	55.364
5	6	4	7.9	7.9	7.9	153.89
32	30	32	53.9	53.9	49.8	73.114
0	0	0	2.5	2.5	2.5	243.08
1	1	1	7.4	7.4	7.4	18.819
0	0	0	5.2	5.2	5.2	34.93
1	1	1	2.1	2.1	2.1	63.251

0	0	0	8.7	8.7	8.7	37.381
0	0	0	6.1	6.1	6.1	102.19
0	0	0	13.4	13.4	13.4	34.455
12	11	13	19.9	19.9	19.9	114.71
3	2	3	33	33	33	11.801
3	4	4	21.5	21.5	21.5	39.31
14	13	13	36.1	36.1	36.1	56.547
3	3	3	13.7	13.7	13.7	32.962
0	0	0	6.9	6.9	6.9	127.72
0	0	0	37.4	37.4	37.4	32.004
0	0	0	1.5	1.5	1.5	113.13
1	1	1	3.8	3.8	3.8	36.567
3	2	3	8.7	8.7	8.7	89.098
0	0	0	11	11	11	38.223
1	1	2	8.6	8.6	8.6	97.263
0	0	0	7.3	7.3	7.3	343.48
0	0	0	9.5	9.5	9.5	34.308
12	12	14	35.9	35.9	35.9	69.491
2	2	2	4.7	3.5	3.5	89.984
0	0	0	8.8	8.8	8.8	37.67
1	1	1	15.5	15.5	15.5	207.57
0	0	0	8.9	8.9	8.9	62.921
0	1	1	12.9	12.9	12.9	51.995
1	1	1	7.4	7.4	7.4	145.17
0	0	0	6.4	6.4	6.4	402.24
1	1	2	68.2	68.2	56	37.655
0	0	0	7.3	7.3	7.3	97.181
0	0	0	15.6	4.5	4.5	40.149
5	5	5	44.3	44.3	44.3	18.419
2	3	3	14.8	14.8	14.8	35.505
23	24	22	33.3	33.3	33.3	148.85
4	7	6	20.3	20.3	20.3	65.7
9	9	11	66.8	66.8	66.8	23.338
7	7	7	34.1	34.1	34.1	39.416
5	6	7	17.7	17.7	17.7	243.87
1	0	1	11	11	11	14.381
10	10	10	39.5	39.5	39.5	29.411
0	0	0	43.7	19.3	12.1	49.857
2	2	2	23.4	23.4	23.4	43.237
0	0	0	18	18	18	87.977
0	0	0	11.2	11.2	11.2	80.709
3	3	3	26.6	26.6	15.7	40.313
1	1	1	55.8	55.8	55.8	10.135
1	2	2	24.6	24.6	24.6	25.43
1	1	1	32	7.2	7.2	33.886

0	0	0	14.5	14.5	14.5	196.71
6	6	6	27.2	27.2	27.2	35.618
0	0	0	6.9	6.9	6.9	39.047
0	0	0	21.7	21.7	21.7	96.754
1	1	1	9.8	6.6	6.6	60.873
0	0	0	12.4	12.4	12.4	287.59
0	0	0	12.5	11.2	11.2	161.61
1	1	1	38.4	38.4	38.4	11.25
4	5	4	11	11	11	53.961
2	1	2	12.9	12.9	12.9	115.73
1	1	1	6.4	6.4	6.4	42.906
1	1	1	25.8	25.8	25.8	37.013
0	0	0	1.4	1.4	1.4	216.5
5	5	5	72.1	72.1	72.1	10.116
2	2	4	20.9	20.9	20.9	65.009
0	0	0	4.1	4.1	4.1	144.89
1	0	1	4.5	4.5	4.5	98.795
0	0	1	39.3	39.3	39.3	149.56
2	2	1	16.2	16.2	16.2	25.624
2	1	2	5.6	5.6	5.6	40.235
21	21	21	26	26	26	127.59
2	3	2	21.4	21.4	21.4	24.992
0	0	0	4.6	4.6	4.6	38.136
0	0	0	4.1	4.1	4.1	52.402
0	0	0	23.5	23.5	23.5	66.999
2	2	2	46.7	46.7	46.1	141.46
7	6	7	30.9	30.9	30.9	39.153
2	3	2	8.1	8.1	8.1	48.713
6	5	6	60.4	60.4	58.1	43.395
0	0	0	2.3	2.3	2.3	219.65
0	0	0	14	14	14	62.54
0	0	0	4.8	0.9	0.9	290.52
3	2	3	15.5	15.5	15.5	32.286
2	2	3	20	20	20	26.383
0	0	0	1.1	1.1	1.1	105.43
2	0	2	11.3	11.3	11.3	29.572
0	0	0	4.8	4.8	4.8	49.844
29	31	31	54.3	54.3	50.8	87.343
0	0	0	7.6	7.6	7.6	332.79
0	0	0	1.1	1.1	1.1	115
0	0	0	10.4	10.4	10.4	25.456
0	0	0	7.6	7.6	7.6	632.81
6	7	8	26.7	26.7	26.7	30.777
1	1	1	13.7	13.7	13.7	22.417
2	2	3	10	10	10	66.171

0	0	0	7.2	7.2	7.2	68.548
3	4	3	7.4	7.4	7.4	117.15
1	0	1	3.6	3.6	3.6	43.963
5	5	4	11.9	11.9	11.9	61.124
0	0	0	10.9	10.9	10.9	41.818
0	0	0	3.6	3.6	3.6	44.301
0	0	0	12.7	12.7	12.7	63.132
0	0	0	12.6	12.6	12.6	103.44
12	13	14	23.4	23.4	23.4	106.12
0	0	0	19.3	19.3	19.3	25.863
1	1	1	3.1	3.1	3.1	121.89
2	2	2	25.3	25.3	25.3	15.892
0	1	1	25.8	25.8	25.8	73.48
3	4	3	8.2	8.2	8.2	82.253
0	0	0	7.6	7.6	7.6	70.293
0	0	0	9.2	9.2	9.2	40.513
3	4	4	15.8	15.8	15.8	30.381
0	0	0	3.1	3.1	3.1	96.619
6	6	6	17.6	17.6	17.6	70.86
1	0	0	7	7	7	260.73
0	0	0	2	2	2	141.52
1	1	1	2.9	2.9	2.9	56.882
4	4	4	12.7	12.7	12.7	170.9
0	0	0	13.8	13.8	2.9	189.03
0	0	0	5.7	5.7	5.7	164.2
3	3	2	7.9	7.9	7.9	91.981
0	0	0	8.7	8.7	8.7	129.99
0	0	0	23.8	23.8	23.8	71.648
10	8	10	45.6	45.6	45.6	55.18
25	21	27	16.5	16.5	16.5	299.61
9	10	12	54.6	54.6	54.6	51.156
0	0	0	2.7	2.7	2.7	66.481
0	0	0	5.3	5.3	5.3	247.89
0	0	0	6.5	6.5	6.5	82.015
11	11	15	41.1	41.1	41.1	67.497
7	6	8	48.9	48.9	48.9	42.033
13	14	15	24.9	24.9	23.8	108.66
10	10	10	44	44	44	59.578
0	0	0	38.7	38.7	38.7	96.914
6	7	8	43.6	43.6	27.8	46.513
0	0	0	7.9	7.9	7.9	30.446
7	9	10	36.1	36.1	36.1	32.334
1	1	1	20	20	20	14.585
1	2	2	9.5	9.5	9.5	55.21
2	2	2	21.9	21.9	21.9	12.254

3	3	3	17.5	17.5	17.5	23.661
9	9	10	18.1	18.1	18.1	87.081
0	0	0	16.1	16.1	16.1	209.52
2	2	2	5.1	5.1	5.1	121.48
5	5	5	33.2	33.2	33.2	83.064
8	9	10	19.7	19.7	19.7	119.91
3	3	1	13.3	13.3	13.3	59.57
2	2	2	7.4	7.4	7.4	101.27
1	0	0	6.2	6.2	6.2	22.774
1	1	1	0	0	0	24.674
0	0	1	0	0	0	44.56
0	0	0	0	0	0	281.76
1	1	1	0	0	0	35.356
1	1	1	0	0	0	94.71
1	1	0	0	0	0	129.95
1	1	1	0	0	0	37.726
0	0	0	0	0	0	22.769
1	1	0	0	0	0	33.593
0	1	0	0	0	0	20.649
0	0	0	0	0	0	154.59
0	0	0	0	0	0	81.828
1	1	0	0	0	0	73.695
0	0	0	0	0	0	23.652
1	1	1	0	0	0	110.14
1	1	1	0	0	0	62.179
1	1	1	0	0	0	228.23
0	0	0	67.1	32.9	32.9	80.509
11	11	13	16.4	16.4	16.4	145.44
4	5	5	42.4	42.4	42.4	19.526

Sequence ler	Sequence ler	Fraction aver	Fraction 1	Fraction 3	Q-value	Identificatior
90	90;148;148;1	1.7	15	8	0	By MS/MS
174	174;174;106;	1.62	9	4	0	By MS/MS
157	157;157	1.67	6	3	0	By MS/MS
817	817;1209;12:	2.4	16	37	0	By MS/MS
519	519;583;664;	1.97	15	14	0	By MS/MS
495	495;618;619	1.67	6	3	0	By MS/MS
372	372;372;460;	1	3		0	By MS/MS
164	164;167;188;	1.91	24	20	0	By MS/MS
437	437;437;437;	2.17	17	24	0	By MS/MS
446	446;522;522	1	5		0	By MS/MS
474	474;474;362;	2.29	6	11	0	By MS/MS
240	240;297;297;	2.33	3	6	0	By matching
346	346;346;346;	2.08	12	14	0	By MS/MS
975	975;1042;10!	2	3	3	0.0079628	By matching
277	277;318;157;	1	9		0	By MS/MS
255	255;282;294;	1	2		0	By MS/MS
385	385;385;385	1.81	25	17	0	By MS/MS
83	83;257;257	1	1		0.0054091	By MS/MS
1475	1475;1475	1.38	69	16	0	By MS/MS
1007	1007;1007;9!	1.74	17	10	0	By MS/MS
126	126;126;126;	2	25	25	0	By MS/MS
130	130;130;130;	2	3	3	0	By matching
1327	1327;1353;1:	2.01	62	63	0	By MS/MS
348	348;362;362;	1	6		0	By MS/MS
589	589;589;623;	1	34		0	By MS/MS
146	146;174;174;	1.86	4	3	0	By MS/MS
531	531;531;288	1.41	23	6	0	By MS/MS
112	112;342;342	1	3		0.00072674	By MS/MS
756	756;756;766;	1.59	12	5	0	By MS/MS
538	538;548;548	1	24		0	By MS/MS
353	353;456;456;	1.06	35	1	0	By MS/MS
700	700;700	1	2		0.0041068	By MS/MS
707	707;707;707;	1	2		0	By MS/MS
490	490;490;71;2	2	6	6	0.0021112	By MS/MS
800	800;820;820;	1.9	11	9	0	By MS/MS
278	278;289;292;	1	3		0	By MS/MS
502	502;502;381;	1.97	15	14	0	By MS/MS
598	598;598;598;	1	6		0	By matching
74	74;133;155;3	1	3		0.00073692	By MS/MS
632	632;632;632;	1.86	12	9	0	By MS/MS
859	859;923;942;	1	3		0	By MS/MS
1197	1197;1197;1:	1	47		0	By MS/MS
345	345;345;418	2	3	3	0	By MS/MS
552	552;552;182	2.45	6	16	0	By matching

929	929;929;797;	1.5		24	8	0	By MS/MS
790	790;790;82	2.33		3	6	0	By MS/MS
520	520;520;520;	2.2		6	9	0	By matching
2170	2170;2220;2:		1	7		0	By MS/MS
424	424;537;537;		1	24		0	By MS/MS
384	384;391		1	6		0	By MS/MS
246	246;246;245;		1	3		1	By MS/MS
793	793;793;690	2.5		3	9	0	By matching
559	559;559;609;	2.04		13	14	0	By MS/MS
717	717;717;752;		1	15		0	By MS/MS
134	134;140;140;	1.22		24	3	0	By MS/MS
560	560;560;560	1.57		5	2	0	By MS/MS
288	288;393;411;	1.67		30	15	0	By MS/MS
69	69;136;136		1	3		0	By matching
425	425;425;455;		3		14	0	By matching
1091	1091;1101;1:	1.29		6	1	0	By matching
587	587;587;373;	2.29		10	18	0	By MS/MS
110	110;110	1.7		13	7	0	By MS/MS
59	59;98;222;47		1	3		0	By matching
126	126;136;136;	1.67		36	18	0	By MS/MS
344	344;344;257;		1	67		0	By MS/MS
745	745;745	1.71		11	6	0	By MS/MS
474	474;479;530;	1.8		3	2	0.0066578	By MS/MS
170	170;170;179;		1	6		0	By MS/MS
283	283;311;326;		1	13		0	By MS/MS
463	463;463;464;	2.17		72	101	0	By MS/MS
462	462;462;462		1	6		0	By MS/MS
1167	1167;1173;9(	1.78		25	16	0	By MS/MS
169	169;171;174;	1.67		24	12	0	By MS/MS
120	120;126;140;		1	3		0.0098814	By MS/MS
473	473;473;513		1	6		0.0007391	By MS/MS
369	369;432;432;		1	5		0	By MS/MS
1451	1451;1451;7⁄		1	33		0	By MS/MS
133	133;135;135;	1.83		21	15	0	By MS/MS
495	495;495;495;	1.4		12	3	0	By MS/MS
83	83;93;93;93;!		1	3		0	By matching
263	263;295;295;	1.75		15	9	0	By MS/MS
291	291;291;291;	2.08		12	14	0	By MS/MS
709	709;739;774;		1	6		0	By MS/MS
204	204;204;164;	1.62		33	15	0	By MS/MS
464	464;764;791;		1	9		0	By MS/MS
815	815;815;505;	1.33		15	3	0	By MS/MS
537	537;549	1.19		19	2	0	By MS/MS
711	711;729;729;		1	6		0	By MS/MS
157	157;159;161	1.5		6	2	0	By MS/MS

206	206;221;517;		3		6	0	By matching
237	237;237;152;	2.04		12	13	0	By matching
282	282;282	1.8		6	4	0	By MS/MS
96	96;200;229	2.33		3	6	0	By MS/MS
3056	3056;3056;1(		1	22		0	By MS/MS
368	368;368		2	3	3	0	By MS/MS
1014	1014;1014;9!	2.07		54	62	0	By MS/MS
291	291;291		1	2		0.0099141	By MS/MS
475	475;498;558;	2.1		9	11	0	By MS/MS
161	161;161;180;		1	15		0	By MS/MS
225	225;225;29;6		1	5		0.00070922	By MS/MS
573	573;573;517;	1.36		41	9	0	By MS/MS
58	58;77;101;10	1.6		7	3	0	By MS/MS
196	196;399;399;	2.3		7	13	0	By MS/MS
577	577;577;514;	2.14		9	12	0	By MS/MS
1500	1500;1500;1!		1	81		0	By MS/MS
709	709;720;720;		1	6		0	By matching
710	710;710;633;	2.24		34	55	0	By MS/MS
372	372;414;414;	1.97		17	16	0	By MS/MS
195	195;434;434;		3		4	0	By matching
305	305;316;338;		1	14		0	By MS/MS
194	194;194;113;	1.43		18	5	0	By MS/MS
200	200;204;204;	1.71		22	12	0	By MS/MS
183	183;183;69	1.86		4	3	0.0021053	By matching
586	586;699;720		1	12		0	By MS/MS
468	468;468		1	6		0	By MS/MS
529	529;569;570;	1.91		6	5	0	By MS/MS
709	709;709;154;	1.64		34	16	0	By MS/MS
668	668;668	1.69		51	27	0	By MS/MS
571	571;548;630;	1.92		53	45	0	By MS/MS
341	341;555;575;		1	9		0	By MS/MS
391	391;391;405;	1.67		6	3	0	By matching
622	622;622;622;		1	5		0.006689	By MS/MS
201	201;336;551;		1	3		0.0020921	By MS/MS
773	773;773;773		1	4		0	By MS/MS
475	475;475;475;		1	6		0	By MS/MS
49	49;83;124;14		1	2		0.0098749	By MS/MS
142	142;150;165;		2	3	3	0	By MS/MS
2101	2101;2099;1!	1.68		522	267	0	By MS/MS
230	230;396		3		4	0.0034317	By matching
666	666;681;682;		1	12		0	By MS/MS
66	66;163;163;1		2	3	3	0	By MS/MS
68	68;95;138;14		1	3		0.0067024	By MS/MS
339	339;339;339;	1.87		69	53	0	By MS/MS
345	345;457;485;		2	2	2	0	By MS/MS

113	113;114;114;		2	3	3	0	By MS/MS
1045	1045;1045;1(	1.42		45	12	0	By MS/MS
104	104;104;71	1.94		17	15	0	By MS/MS
272	272;272;326;	2.33		4	8	0	By MS/MS
644	644;690;782;		1	6		0.00072516	By MS/MS
271	271;390;466;		2	3	3	0.00072833	By MS/MS
508	508;651		1	4		0	By MS/MS
412	412;412;128;		1	30		0	By MS/MS
61	61;61;61;61;(		1	3		0	By MS/MS
105	105;227;227;	2.33		3	6	0	By MS/MS
182	182;182;82;1		1	18		0	By MS/MS
1455	1455;1531;14		1	9		0	By MS/MS
759	759;759;387;	1.5		9	3	0	By MS/MS
315	315;315;315;		1	9		0	By MS/MS
574	574;703;757;	2.5		3	9	0	By MS/MS
127	127;163;220;		1	3		0	By MS/MS
894	894;894;702;	2.16		44	61	0	By MS/MS
527	527;527;587;	1.5		6	2	0	By MS/MS
1616	1616;1678;1(	1.89		73	58	0	By MS/MS
1082	1082;1082;1(	1.3		91	16	0	By MS/MS
436	436;633;633;	1.5		9	3	0	By MS/MS
435	435;645;645;	1.8		3	2	0.00071073	By matching
719	719;719;596;	1.77		16	10	0	By MS/MS
415	415;415;338	1.88		60	47	0	By MS/MS
224	224;224		1	3		0.00070872	By matching
823	823;863;863;		1	10		0	By MS/MS
2997	2997;3011;1		1	49		0	By MS/MS
65	65;89;112;11	1.5		3	1	0.00073153	By matching
248	248;259;208;	1.56		28	11	0	By MS/MS
85	85;85		2	3	3	0	By MS/MS
2685	2685;2968;2!		1	7		0	By MS/MS
305	305;305;158		1	6		0	By MS/MS
96	96;123;169;9	1.5		9	3	0	By MS/MS
820	820;837;870;		1	18		0	By MS/MS
2452	2452;2457;24	2.5		2	6	0	By matching
266	266;277;277;	1.5		6	2	0	By matching
200	200;207;219;		1	2		0	By MS/MS
572	572;572	1.4		24	6	0	By MS/MS
1519	1519;1519;2(		1	24		0	By MS/MS
905	905;905;905;	1.71		9	5	0	By MS/MS
223	223;223;70;1		2	5	5	0	By MS/MS
234	234;234;234;	1.86		4	3	0	By matching
64	64;67;70;70;:	1.73		21	12	0	By MS/MS
95	95;95;95;103		3		7	0	By matching
492	492;508;508;	2.01		36	37	0	By MS/MS

133	133;221;221;	2.19		24	35	0	By MS/MS
1121	1121;1123;9(	2.35		20	42	0	By MS/MS
411	411;411;125;	2.1		18	22	0	By MS/MS
561	561;741;743;		1	6		0	By MS/MS
725	725;726;747;		3		3	0	By matching
538	538;538;573;		1	15		0	By MS/MS
419	419;522;604;	1.55		37	14	0	By MS/MS
115	115;115;114;	1.96		55	51	0	By MS/MS
100	100;101;101;	1.46		10	3	0	By MS/MS
1390	1390;1390;7(		1	16		0	By MS/MS
631	631;631;181;	1.36		23	5	0	By MS/MS
318	318;318;176		1	6		0	By matching
420	420;439;439;		1	15		0	By MS/MS
91	91;123;128;1		1	3		0.00071942	By MS/MS
1883	1883;1894;1(	1.12		49	3	0	By MS/MS
222	222;247;247;		1	3		0.0079681	By MS/MS
575	575;582;582	1.29		29	5	0	By MS/MS
81	81;81		1	6		0	By MS/MS
848	848;848		1	6		0.0021142	By MS/MS
1086	1086;1124;1:	1.49		71	23	0	By MS/MS
454	454;454;454;	1.07		26	1	0	By MS/MS
353	353;353;341;	2.07		64	74	0	By MS/MS
1076	1076;1076		1	6		0	By MS/MS
522	522;522;457	2.02		98	102	0	By MS/MS
439	439;456;456;		1	6		0	By MS/MS
670	670;670;546;	1.92		34	29	0	By MS/MS
346	346;423;423;		3		6	0	By matching
128	128;128;128;	1.83		21	15	0	By MS/MS
455	455;455;448;	2.31		17	32	0	By MS/MS
391	391;430;430;	1.6		33	14	0	By MS/MS
307	307;320;372;	2.06		51	58	0	By MS/MS
238	238;264;298;	2.09		15	18	0	By MS/MS
115	115;115;115;	1.86		8	6	0	By MS/MS
1156	1156;1214;1:	1.89		55	44	0	By MS/MS
160	160;160;164;		3		3	0	By matching
71	71;136;148;1		2	3	3	0	By MS/MS
225	225;225;175	1.67		6	3	0	By MS/MS
193	193;193;187;	1.75		64	38	0	By MS/MS
911	911;911;274	2.29		6	11	0	By MS/MS
454	454;454;414;	2.13		13	17	0	By MS/MS
361	361;361;361;	1.62		29	13	0	By MS/MS
367	367;914;103(		3		3	0	By matching
474	474;474;474;	1.93		15	13	0	By MS/MS
503	503;563;564;		2	3	3	0	By MS/MS
288	288;288;288;	1.66		43	21	0	By MS/MS

294	294;304;323;	1.98		21	20	0	By MS/MS
160	160;198;198	1.67		4	2	0	By MS/MS
261	261;261;200		2	3	3	0	By matching
317	317;317;317;	1.11		18	1	0	By MS/MS
2062	2062;2458;2		1	30		0	By MS/MS
472	472;472;472;	1.2		18	2	0	By MS/MS
268	268;268;268	2.43		6	15	0	By MS/MS
327	327;327	1.8		3	2	0	By MS/MS
368	368;368		1	7		0	By MS/MS
943	943;1191;16		3		12	0	By matching
620	620;620;195;	1.18		20	2	0	By MS/MS
1494	1494;1645;1		1	4		0.0099272	By matching
115	115;115;92		3		6	0	By matching
632	632;632;632;		1	12		0	By MS/MS
604	604;604;604		1	4		0	By MS/MS
441	441;578;578;		1	24		0	By MS/MS
205	205;209;209;		2	9	9	0	By MS/MS
428	428;428;425;	1.81		16	11	0	By MS/MS
270	270;325;344;		1	6		0.0021127	By matching
1176	1176;1210;1	1.07		140	5	0	By MS/MS
1126	1126;1132;1		1	18		0	By MS/MS
665	665;673;673;	1.17		32	3	0	By MS/MS
107	107;328;328	2.5		1	3	0	By matching
374	374;429;450;		1	3		0	By MS/MS
166	166;166		1	3		0	By matching
123	123;174;310;		1	3		0.00072622	By MS/MS
514	514;602;602;	2.33		3	6	0	By MS/MS
262	262;289;299;		1	3		0	By MS/MS
2440	2440;2440;2		1	21		0	By MS/MS
1176	1176;1189;1		1	4		0	By MS/MS
351	351;686;714;		1	3		0.0027759	By matching
194	194;282		1	6		0	By MS/MS
655	655;655;655;		3		18	0	By matching
306	306;355;355;	1.91		41	34	0	By MS/MS
271	271;363;420;	1.67		6	3	0	By matching
117	117;117;62	2.25		3	5	0.0021157	By MS/MS
217	217;217	1.86		4	3	0	By matching
444	444;462;462;		1	5		0	By MS/MS
488	488;500;529;		1	8		0	By MS/MS
393	393;463;523		1	3		0.0021082	By MS/MS
305	305;356;356;	1.28		37	6	0	By MS/MS
158	158;174;176;		3		11	0	By matching
1377	1377;1377;1		1	39		0	By MS/MS
522	522;533;733;		1	3		0	By MS/MS
223	223;273;273	2.14		9	12	0	By MS/MS

718	718;972;109[illegible]	1	12		0	By MS/MS
Q06;T1WGK5;T1WE98;T1RT90;S6CRG7;S6BEP5;S6B3R2;S6B0Q5;S5U3G8;S5TLC0;S5NIY1;S5DU27;S5DN96;S5DN91;S5DLI1;						
8R8H8;F8R8H7;F8R8H6;F8R8H5;F8R8H4;F8R8H3;F8R8H2;F8R8H1;F8R8H0;F8R8G9;F8R8G8;F8R8G7;F8R8G6;F8R8G5;F8R8[illegible]						
T2B9H9;T1WQ06;T1WGK5;T1WE98;T1RT90;S6CRG7;S6BEP5;S6B3R2;S6B0Q5;S5U3G8;S5TLC0;S5NIY1;S5DU27;S5DN96;S5[illegible]						
8R8H8;F8R8H7;F8R8H6;F8R8H5;F8R8H4;F8R8H3;F8R8H2;F8R8H1;F8R8H0;F8R8G9;F8R8G8;F8R8G7;F8R8G6;F8R8G5;F8R8[illegible]						
206;242;251;264;264;269;271;272;273;273;273;273;273;273;273;273;273;273;273;273;273;2						
0	0	0	0	1	2	0
301	301;301;304;	1.89	15	12	0	By MS/MS
1385	1385;741;39[illegible]	1	38		0	By MS/MS
118	118	1	1		0	By MS/MS
1179	1179;1298;1[illegible]	1	4		0	By matching
59	59;135;136;1	1	3		0.00073046	By matching
5063	5063;5088;5[illegible]	1	3		0	By MS/MS
395	395;449;592;	2	6	6	0	By MS/MS
155	155;196;197;	1.17	11	1	0	By MS/MS
192	192;221;244;	3		1	0	By matching
207	207;216;220;	2	2	2	1	By matching
793	793;806;857;	1.91	50	42	0	By MS/MS
103	103;127;127	1	3		0	By matching
157	157;226;226;	1.75	5	3	0	By matching
1529	1529;1542;1[illegible]	1	6		0	By MS/MS
155	155;288;288	1.4	12	3	0	By MS/MS
359	359;401;707;	1	3		0.00072359	By MS/MS
237	237;259	1	12		0	By MS/MS
131	131;132;133	1	6		0	By MS/MS
258	258;434;485;	1.67	6	3	0	By MS/MS
3255	3255;3256;1[illegible]	1.98	577	557	0	By MS/MS
812	812;855;275;	2.62	7	30	0	By MS/MS
129	129;137;224[illegible]	1	3		0.0027797	By MS/MS
643	643;730;737;	2.14	12	16	0	By MS/MS
357	357;379;381;	1	12		0	By MS/MS
733	733;913;978;	1	9		0	By MS/MS
4588	4588;4590	1	3		0.0054237	By MS/MS
749	749;917;938;	1	6		0.0021023	By matching
1087	1087;1095;1[illegible]	1	3		0	By matching
184	184;204;210;	1	3		0	By matching
84	84;84;84;84;[illegible]	2	6	6	0	By MS/MS
1970	1970;1970;1[illegible]	1	18		0	By MS/MS
403	403;415;900;	3		32	0	By matching
933	933;977;316	2.5	3	9	0	By MS/MS
411	411;685;754;	1	3		0.009901	By MS/MS
315	315;368;407	1	3		0	By MS/MS
1026	1026;1320;6[illegible]	1	4		0.0047619	By MS/MS
355	355;373;373;	2	3	3	0	By MS/MS
111	111;422;422	1.5	3	1	0	By MS/MS

146	146;212;241;		2	12	12	0	By MS/MS
585	585;615		1	6		0.00073099	By matching
172	172;253;488;		1	3		0.00071891	By MS/MS
208	208;341;341;		3		3	0	By matching
569	569;570;579;		1	21		0	By MS/MS
98	98;155		2	3	3	0	By matching
127	127;146;146		3		1	0	By matching
529	529;543;306;		1	3		0	By MS/MS
96	96;606;739;7		1	2		0	By MS/MS
129	129;129;146;	1.67		12	6	0	By MS/MS
280	280;283;323;	1.74		12	7	0	By MS/MS
871	871;895;799;	1.68		47	24	0	By MS/MS
286	286;288;288		3		3	0.00070822	By matching
998	998;998;105	2.14		12	16	0	By MS/MS
440	440;457;467;	1.67		6	3	0	By MS/MS
177	177;199;843;		1	3		0.00072886	By matching
289	289;340;484;		1	3		0	By MS/MS
529	529;659;659;		2	3	3	0.0020935	By MS/MS
150	150;178;231;	1.88		14	11	0	By MS/MS
404	404;404;404;		3		8	0	By matching
707	707;746;751;	1.4		12	3	0	By MS/MS
221	221;221;315;	1.82		26	18	0	By MS/MS
655	655;655;674;		2	3	3	0.0066845	By MS/MS
348	348;388;400;	1.4		8	2	0	By MS/MS
930	930;995;995;		1	19		0	By MS/MS
314	314;591;600;		1	5		0	By MS/MS
120	120;124;130;		1	3		0	By MS/MS
699	699;699;708;	2.06		50	56	0	By MS/MS
802	802;876		1	14		0	By MS/MS
965	965;1054;10		1	9		0	By MS/MS
159	159;159;159;		2	3	3	0	By matching
388	388;393;422;		1	3		0	By matching
1410	1410;1410;1		1	12		0	By MS/MS
420	420;420;446;	2.5		3	9	0	By MS/MS
362	362;482;482;		2	3	3	0	By matching
316	316;1215;12		1	3		0	By MS/MS
279	279;279;389;	1.87		26	20	0	By MS/MS
572	572;830;464;		1	183		0	By MS/MS
150	150;224;306;		3		3	0.0047716	By matching
895	895;951	2.29		6	11	0	By MS/MS
452	452;452;483;		1	13		0	By MS/MS
1849	1849;1849;1		1	3		0.00070771	By matching
176	176;193;206;		1	3		0	By MS/MS
426	426;437;450;	1.91		6	5	0	By MS/MS
524	524;556;704;		1	3		0	By matching

589	589;605;292;		1	12		0	By MS/MS
473	473;520;794;	2.47		4	11	0	By matching
641	641;641		1	6		0	By MS/MS
137	137;165;235;	1.92		28	24	0	By MS/MS
99	99;122;123;1		1	3		0	By MS/MS
104	104;137;148;		3		3	0	By matching
1003	1003;1051;1(		1	9		0	By MS/MS
498	498;610;740;		1	3		0	By matching
562	562;626;228	1.33		30	6	0	By MS/MS
478	478;672;754;		2	3	3	0	By MS/MS
211	211;216;218;	1.95		21	19	0	By MS/MS
1032	1032;1032;1(	2.22		36	56	0	By MS/MS
992	992;1008;10(	2.4		13	30	0	By MS/MS
1886	1886;1905;1!	1.65		84	40	0	By MS/MS
141	141;167;221;	1.94		9	8	0	By MS/MS
833	833;852;864;		1	11		0	By MS/MS
330	330;337	2.12		11	14	0	By MS/MS
718	718;910;922;		1	3		0	By MS/MS
640	640;647;662;	1.99		110	108	0	By MS/MS
474	474;514;376;		1	20		0	By MS/MS
365	365;366;324;	1.98		21	20	0	By MS/MS
1044	1044;1045		1	4		0	By matching
490	490;490;595;		1	8		0	By MS/MS
198	198;199;540	1.8		12	8	0	By MS/MS
641	641;641;641;	1.52		82	29	0	By MS/MS
322	322;322;556;		1	3		0.00073421	By matching
181	181;201;205;		3		4	0	By matching
1630	1630;1630;1(	1.36		36	8	0	By MS/MS
234	234;486;489;		1	3		0	By MS/MS
357	357;389;390;	1.16		35	3	0	By MS/MS
457	457;1085;79;	1.67		6	3	0	By matching
89	89;227;227;2		2	3	3	0	By MS/MS
505	505;522;534;	2.08		18	21	0	By MS/MS
342	342;354;354;		1	3		0.0027548	By MS/MS
118	118;248	1.8		9	6	0	By MS/MS
1435	1435;1435;1!	1.86		8	6	0	By MS/MS
777	777;777;777;	1.94		9	8	0	By matching
570	570;808;518;		3		11	0	By matching
827	827;1065;10!		1	11		0	By MS/MS
513	513;513		2	3	3	0	By MS/MS
446	446;446;446;	1.83		21	15	0	By MS/MS
357	357;357;357;		1	12		0	By MS/MS
459	459;466;118!		1	6		0	By MS/MS
430	430;440;464;	2.14		32	42	0	By MS/MS
124	124;124;128;	1.93		15	13	0	By MS/MS

773	773;798;798		1	37		0	By MS/MS
776	776;776;416;		1	18		0	By MS/MS
249	249;249;249;	1.52		17	6	0	By MS/MS
868	868;938;939;		3		2	0.0021038	By matching
247	247;297;297;	1.45		38	11	0	By MS/MS
88	88;181;237;2		2	3	3	0.0060362	By MS/MS
690	690;690;690;	2.18		25	36	0	By MS/MS
219	219;219;219;		2	6	6	0	By MS/MS
127	127		1	2		0.00071276	By MS/MS
183	183;183;62;1	2.05		18	20	0	By MS/MS
2190	2190;2394;2		1	12		0	By MS/MS
387	387;410;410;		1	4		0	By MS/MS
812	812;822;974;		1	9		0	By MS/MS
642	642;651;651;		1	6		0	By matching
245	245;269;323;	1.4		12	3	0	By MS/MS
1688	1688;1709		1	3		0.002095	By MS/MS
50	50;270;329;3	2.2		2	3	0.00071736	By matching
392	392;423;439;		2	3	3	0	By MS/MS
1460	1460;1486;1		1	9		0	By MS/MS
2253	2253;2255		3		6	0.0054018	By matching
255	255		3		4	0	By matching
2035	2035;2080;5	1.79		71	46	0	By MS/MS
332	332;395;301		1	8		0	By MS/MS
1370	1370;1372;1		1	47		0	By MS/MS
2005	2005;1388	2.08		12	14	0	By MS/MS
1760	1760;1927;1	2.54		6	20	0	By MS/MS
264	264;438;525		1	5		0.0098555	By matching
235	235;295;472;		3		4	0.00071023	By matching
1647	1647;1647;1	1.74		60	35	0	By MS/MS
1065	1065;1065;1		1	3		0	By MS/MS
887	887;929;929		3		3	0	By matching
524	524;642;642;		1	12		0	By MS/MS
288	288;374;374;		1	3		0	By MS/MS
756	756;756		3		7	0	By matching
973	973;1017;80	1.2		18	2	0	By MS/MS
248	248;248;248;	1.36		9	2	0	By MS/MS
205	205;205		1	6		0	By MS/MS
857	857;861;878;	1.5		9	3	0	By MS/MS
716	716;739;739	1.5		15	5	0	By MS/MS
259	259;282;128	2.03		14	15	0	By MS/MS
419	419;419		1	3		0	By matching
668	668;668;741;		1	8		0	By MS/MS
535	535;564;564;	2.08		6	7	0	By MS/MS
278	278;332;461;		1	17		0	By MS/MS
669	669;669		2	3	3	0.0041096	By MS/MS

730	730;852;189;	1.41		27	7	0	By MS/MS
716	716;759;811;	2.2		6	9	0	By MS/MS
812	812;830;81		1	7		0	By MS/MS
852	852		2	3	3	0	By MS/MS
389	389;483;483;		1	7		0	By matching
432	432;432;251;	1.97		32	30	0	By MS/MS
502	502;502		1	6		0	By MS/MS
555	555;579;589;		1	10		0	By MS/MS
221	221;221;119;	2.33		6	12	0	By matching
216	216	1.8		3	2	0	By MS/MS
521	521;567;634;		1	3		0.0053908	By MS/MS
340	340;340;351;		1	15		0	By MS/MS
725	725;725;684;	1.9		80	66	0	By MS/MS
340	340;356;356;		1	8		0	By MS/MS
379	379;379;79		1	9		0	By matching
246	246;272;272;		3		2	0	By matching
165	165;165;165;	2.11		33	41	0	By MS/MS
246	246;396;396		2	3	3	0	By MS/MS
441	441;441		1	9		0	By MS/MS
211	211;211;211;	1.65		31	15	0	By MS/MS
300	300;429;525;	1.94		17	15	0	By MS/MS
380	380;380;128;		1	10		0	By MS/MS
1524	1524;1524;9	1.96		12	11	0	By MS/MS
126	126;181;237;	1.8		9	6	0	By MS/MS
136	136;338;377;		1	3		0	By MS/MS
264	264;264;264;	1.67		36	18	0	By MS/MS
70	70;137;158;1		3		4	0	By matching
143	143;143;134;	1.73		14	8	0	By MS/MS
691	691;734;734;	2.15		30	41	0	By MS/MS
471	471;471;234;	2.33		18	36	0	By MS/MS
1130	1130;1130;1	1.39		62	15	0	By MS/MS
448	448;501;501;		2	6	6	0	By MS/MS
279	279;344;344;	1.98		23	22	0	By MS/MS
963	963;963		1	3		0.0047587	By matching
691	691;691		1	6		0	By MS/MS
419	419;494;494	2.2		2	3	0	By matching
282	282;282		2	3	3	0	By MS/MS
628	628;628;489;	1.24		22	3	0	By MS/MS
1217	1217;1217;8	1.62		29	13	0	By MS/MS
792	792;846;846	1.11		18	1	0	By MS/MS
358	358;381;381;		1	8		0	By matching
529	529;529;529;	1.24		15	2	0	By MS/MS
406	406;406;134;	1.5		6	2	0	By MS/MS
417	417;490;565;	2.26		23	39	0	By MS/MS
58	58;100;130;1		1	3		0.00073475	By MS/MS

436	436;436;127;		2	6	6	0	By MS/MS
271	271;338;497;		2	3	3	0.0027682	By matching
562	562;562		1	12		0	By MS/MS
306	306;306;214;	1.8		9	6	0	By MS/MS
510	510;510;392;	1.88		9	7	0	By MS/MS
486	486;486;486;		3		5	0	By matching
524	524;524;154		1	9		0	By MS/MS
537	537;537;542;	2.32		21	41	0	By MS/MS
152	152;157;486;		1	1		0	By matching
800	800;800;501		1	9		0	By MS/MS
298	298;410;476;	2.33		3	6	0	By MS/MS
745	745;764;764;	2.07		27	31	0	By MS/MS
717	717;717;117;	2.09		32	38	0	By MS/MS
594	594;594;447;	1.69		82	43	0	By MS/MS
902	902;902;447;	2.5		2	6	0	By MS/MS
957	957;976;977;	1.22		32	4	0	By MS/MS
788	788;857;857;	1.67		6	3	0	By MS/MS
946	946;1068;10(		1	3		1	By MS/MS
108	108;473;473		1	3		0	By MS/MS
396	396;396;520;		1	6		0	By MS/MS
621	621;621;565;		1	15		0	By MS/MS
972	972;972;972;	1.87		31	24	0	By MS/MS
375	375;410;476;		1	3		0	By MS/MS
169	169;231;231;	1.75		5	3	0.00072098	By MS/MS
64	64;68;76;76	1.91		6	5	0	By MS/MS
847	847;895;794;	1.69		204	108	0	By MS/MS
559	559;958;958		2	3	3	0	By MS/MS
774	774;789;740;		1	19		0	By MS/MS
1217	1217;1217;1:	1.31		60	11	0	By MS/MS
660	660;706;744		2	3	3	0	By MS/MS
394	394;412		1	9		0	By MS/MS
618	618;656;584;	1.25		21	3	0	By MS/MS
111	111;150;156;		1	3		0.0027836	By matching
145	145;145;157;	1.71		9	5	0	By MS/MS
128	128;128;132	1.91		12	10	0	By MS/MS
169	169;191		1	3		0.0047522	By matching
453	453;465;123!		1	6		0	By MS/MS
466	466;487;509;	2.56		8	28	0	By MS/MS
303	303;375;954	1.67		6	3	0	By MS/MS
165	165;170;195;	2.33		3	6	0	By MS/MS
103	103;202;202;		3		3	0.00668	By matching
243	243;263;263;	1.62		47	21	0	By MS/MS
125	125;125;108;	1.56		18	7	0	By MS/MS
114	114;114		3		3	0.0060565	By matching
103	103;103;103;	1.79		32	21	0	By MS/MS

69	69;69	1.5		9	3	0	By MS/MS
106	106;106;106;	1.67		6	3	0	By MS/MS
262	262;288;290;	1.98		50	48	0	By MS/MS
557	557;557;557;	1.8		9	6	0	By MS/MS
164	164;164;95	1.88		47	37	0	By MS/MS
552	552;679;755;		1	3		0.0027643	By MS/MS
643	643;643;643;	1.86		4	3	0	By MS/MS
749	749;749;771;		1	23		0	By MS/MS
577	577;733;733	2.68		6	31	0	By MS/MS
237	237;296;296		1	3		0.0041124	By matching
423	423;443;443;		2	27	27	0	By MS/MS
808	808;808;808;	2.16		51	70	0	By MS/MS
203	203;203;203;		1	6		0.00074019	By MS/MS
521	521;521;522;	1.62		9	4	0	By MS/MS
225	225;225;225	1.67		6	3	0	By MS/MS
559	559;559	2.33		3	6	0	By MS/MS
136	136;252;252		2	3	3	0	By MS/MS
603	603;603;506;	1.41		35	9	0	By MS/MS
564	564;547		3		2	0	By matching
785	785;1162;12		1	3		0.004788	By matching
473	473;473;138;	1.22		24	3	0	By MS/MS
235	235;271;271;		1	3		0.0047814	By matching
547	547;748;748;		1	2		0.00071124	By matching
755	755;755		1	6		0.00072939	By MS/MS
794	794;794;794;	1.25		28	4	0	By MS/MS
484	484;485;547;		1	3		1	By matching
1286	1286;1417;1	1.81		16	11	0	By MS/MS
521	521;556;43	1.91		6	5	0	By MS/MS
306	306;336;336;	1.84		25	18	0	By MS/MS
586	586;586;101;		1	22		0	By MS/MS
719	719;719;719;		1	15		0	By MS/MS
158	158;333;333		2	3	3	0	By matching
445	445;445		1	1		1	By matching
1102	1102;1102;1		1	21		0	By MS/MS
876	876;876;148;	1.64		17	8	0	By MS/MS
119	119	2.5		1	3	0.0066934	By MS/MS
466	466;315;117;	1.89		63	51	0	By MS/MS
127	127;197;259;	2.4		3	7	0	By MS/MS
449	449;449;571;	1.78		11	7	0	By MS/MS
335	335;342;515;		1	6		0	By MS/MS
252	252;276;278;	2.01		36	37	0	By MS/MS
147	147;149;149;	1.29		12	2	0	By MS/MS
156	156;156;156;	1.71		33	18	0	By MS/MS
631	631;631;166;		1	6		0	By MS/MS
1074	1074;1074;6	1.88		68	53	0	By MS/MS

1184	1184;1190;4(	1.62		18	8	0	By MS/MS
2144	2144;2036;2(		1	49		0	By MS/MS
2427	2427;2696;1(		1	48		0	By MS/MS
1004	1004;1004;1(		1	83		0	By MS/MS
765	765;765;796;		1	11		0	By MS/MS
118	118;118	1.8		3	2	0	By MS/MS
211	211;211;211	1.38		21	5	0	By MS/MS
453	453;478;480;	1.29		18	3	0	By MS/MS
105	105;308;308;	1.23		23	3	0	By MS/MS
896	896;950;950;	1.96		24	22	0	By MS/MS
510	510;510;510;	2.5		6	18	0	By MS/MS
1297	1297;1297;1(	1.81		66	45	0	By MS/MS
648	648;660;559;		1	57		0	By MS/MS
207	207;225;247;		1	3		0.009849	By matching
749	749;749;216;	1.88		14	11	0	By matching
418	418;760;760;	1.5		3	1	0.0007148	By MS/MS
476	476;476;482;	1.3		17	3	0	By MS/MS
603	603;557;78;1		2	6	6	0	By MS/MS
498	498;509;517;		1	6		0	By MS/MS
527	527;527;563;		1	33		0	By MS/MS
705	705;705		1	3		0.00071685	By matching
351	351;425;731;		1	1		0.0066534	By matching
995	995;995;514;	2.18		32	46	0	By MS/MS
416	416;416;451;	1.67		12	6	0	By MS/MS
244	244;309;309;	1.36		9	2	0	By MS/MS
401	401;422;422;	2.08		12	14	0	By MS/MS
722	722;1038;20:		1	9		0	By MS/MS
304	304;312;312;		1	3		0	By matching
523	523;645;681		1	3		0.0047554	By MS/MS
514	514;331;508	2.25		3	5	0	By matching
496	496;622;640;	1.86		25	19	0	By MS/MS
1143	1143;1143;5:		1	19		0	By MS/MS
721	721;721;901;		1	3		0.0020906	By matching
290	290;575;575		1	9		0	By MS/MS
490	490;501;509;		1	8		0	By MS/MS
923	923;923;598;		1	22		0	By MS/MS
432	432;450;450		2	6	6	0	By MS/MS
215	215;265;275;		1	3		0.00072993	By MS/MS
424	424;424;424		2	3	3	0	By MS/MS
186	186;192	1.8		3	2	0	By MS/MS
581	581;581		3		2	0	By matching
447	447;1253;12!		2	9	9	0	By MS/MS
685	685;1105;11:		1	8		0	By MS/MS
1263	1263;1263;1:	1.16		59	5	0	By MS/MS
774	774;808;904;		1	11		0	By MS/MS

775	775;802;802	1.62		45	20	0	By MS/MS
356	356;323;269;		3		3	0	By matching
298	298;309;309;	1.32		32	6	0	By MS/MS
418	418;431		2	3	3	0	By MS/MS
167	167;176;283;		3		3	0.00071994	By matching
563	563;235;276;	2.14		6	8	0	By MS/MS
140	140;167;190;		1	6		0	By MS/MS
275	275;462;490;	1.8		9	6	0	By matching
75	75;120;144;1		1	6		0	By MS/MS
183	183;198;198;	1.8		3	2	0	By MS/MS
986	986;1004;10(		1	43		0	By MS/MS
374	374;462;471;		3		4	0	By matching
164	164;342;206	1.5		9	3	0	By MS/MS
155	155;158;198		3		1	0.00071225	By matching
403	403;494;502;		1	15		0	By MS/MS
707	707;766;617;	2.19		15	22	0	By MS/MS
341	341;356;363;		1	3		0.0053981	By MS/MS
172	172;388;530;		2	3	3	0	By MS/MS
879	879;946;106!		3		2	0.00071378	By matching
331	331;742;749;	2.25		3	5	0	By MS/MS
783	783;803;841;		1	6		0	By MS/MS
413	413;589;664;	2.14		3	4	0	By MS/MS
520	520;583;354;	2.08		6	7	0	By MS/MS
86	86;233;343;3		1	3		0	By MS/MS
756	756;810;652;	2.13		17	22	0	By MS/MS
243	243;396;881		1	3		0	By matching
147	147;166;168;	1.78		14	9	0	By MS/MS
288	288;354;371		3		3	0	By matching
147	147;154;154;	1.68		27	14	0	By MS/MS
234	234;347;118;	1.29		6	1	0	By MS/MS
358	358;385;385;		3		7	0	By matching
598	598;678;759;		3		26	0	By matching
263	263;426;474;		1	9		0	By MS/MS
91	91;188;188;3		1	6		0	By MS/MS
343	343;390;41;6	1.4		24	6	0	By MS/MS
695	695;762;792;	2.42		15	37	0	By MS/MS
268	268;294;326;		1	10		0	By MS/MS
363	363;372;390;	1.29		12	2	0	By MS/MS
626	626;696;741;		1	9		0	By MS/MS
217	217;346;381;		1	3		0.0027529	By MS/MS
227	227;294;294		1	9		0	By MS/MS
377	377;401;404;		1	5		0	By MS/MS
457	457;467;302;	1.15		24	2	0	By MS/MS
277	277;377;418;		1	3		0.0021008	By matching
440	440;785;821		1	3		0.00073964	By matching

616	616;639;645;		1	6		0	By MS/MS
721	721;755	2.33		3	6	0	By matching
776	776;779;133		1	25		0	By MS/MS
87	87;166;208;4		3		3	0	By matching
257	257;4646		1	3		0.0027816	By matching
300	300;478;551	2.14		6	8	0	By MS/MS
493	493;629;644;	2.08		6	7	0	By MS/MS
432	432;476;492;	2.2		6	9	0	By MS/MS
953	953;977;977;	1.18		62	6	0	By MS/MS
367	367;527		1	12		0	By MS/MS
642	642;693;693	2.33		8	16	0	By matching
316	316;531	1.31		11	2	0	By MS/MS
128	128;198;216;	2.09		5	6	0	By MS/MS
220	220;284;449		1	3		0	By MS/MS
228	228;467;527		1	2		0.00071582	By matching
450	450;493;499;	1.91		6	5	0	By matching
19	19;54;178;24		1	3		0	By matching
352	352;390;390;	1.94		26	23	0	By MS/MS
168	168;187;191;	1.97		15	14	0	By MS/MS
248	248;248;323;		1	3		0	By MS/MS
897	897;907;437;		1	11		0	By MS/MS
146	146;456;499		2	3	3	0.0066979	By matching
1195	1195;1196;3		1	4		0.0027701	By matching
1078	1078;1079;1		1	48		0	By MS/MS
995	995;995;105	1.47		69	21	0	By MS/MS
927	927;1230;12	1.3		17	3	0	By MS/MS
404	404;461;461;		3		12	0	By matching
826	826;1140;10	1.4		32	8	0	By MS/MS
577	577;700;345	2.33		9	18	0	By MS/MS
421	421;438;438;	2.32		15	29	0	By MS/MS
784	784;795	1.83		51	36	0	By MS/MS
215	215;335	2.5		1	3	0	By matching
191	191;777;777		2	3	3	0.00070972	By MS/MS
1004	1004;295;15		1	3		0	By MS/MS
1140	1140;1156;9		1	6		0	By MS/MS
439	439;472;350;		3		5	0	By matching
729	729;1101		1	5		0	By MS/MS
225	225;225		2	3	3	0.00071633	By MS/MS
482	482;482;482;	1.67		12	6	0	By MS/MS
456	456;456;315;	1.64		60	28	0	By MS/MS
420	420;420;420	1.67		6	3	0	By MS/MS
662	662;742;745;		1	6		0.00071327	By matching
109	109;316;316;		1	3		0.0066622	By MS/MS
82	82;113;192;2		3		3	0.0098879	By matching
459	459;560;582;		2	3	3	0	By matching

126	126;134;221;	1.67		6	3	0	By MS/MS
567	567;675;675;		1	9		0	By MS/MS
212	212;230;246;		3		1	0.00071429	By matching
499	499;508;508;	1.67		16	8	0	By MS/MS
665	665;679;679;	1.51		67	23	0	By MS/MS
154	154;159;242;		1	1		0.0034294	By MS/MS
459	459;481;536;		1	10		0	By MS/MS
343	343;377;377;		1	3		0	By matching
145	145;151;152;	1.96		14	13	0	By MS/MS
1260	1260;1265		1	15		0	By MS/MS
909	909;1003;10		1	18		0	By MS/MS
797	797;873;100	1.76		18	11	0	By MS/MS
1275	1275;1275;1	1.36		23	5	0	By MS/MS
1606	1606;1980;4		1	12		0	By MS/MS
363	363;513;546;		3		5	0.00072569	By matching
983	983;1035;10		1	12		0	By MS/MS
1276	1276;1292;1		1	3		0	By MS/MS
1070	1070;1117;1		1	21		0	By MS/MS
606	606;606		1	3		0	By MS/MS
1328	1328;1328;1		1	27		0	By MS/MS
2897	2897;2897;1		1	110		0	By MS/MS
113	113;118;140;	1.5		3	1	0	By MS/MS
765	765;765;164;	1.92		14	12	0	By MS/MS
231	231;456;494;		2	3	3	0	By MS/MS
2856	2856;3026;1		1	48		0	By MS/MS
553	553;553;555;	2.07		7	8	0	By matching
403	403;540		3		3	0.0066711	By matching
95	95;459;502;4		1	3		0	By MS/MS
169	169;174;182;		1	3		0	By MS/MS
225	225;245;279;	1.5		9	3	0	By MS/MS
105	105;109;115;		3		3	0.0054127	By matching
68	68;92;60;72;	1.5		9	3	0	By MS/MS
164	164;293;102;		1	6		0	By MS/MS
205	205;1336		2	3	3	0.00070621	By MS/MS
63	63;98;58		1	6		0	By MS/MS
174	174;185	1.5		6	2	0	By MS/MS
891	891;941;941;	1.2		72	8	0	By MS/MS
103	103;115;144		2	3	3	0	By matching
101	101;105;105;		2	6	6	0	By matching
52	52;118;118		1	3		0	By MS/MS
121	121;150;157;	1.57		15	6	0	By MS/MS
372	372;421;421;	2.25		9	15	0	By MS/MS
137	137;140		3		3	0.0060525	By matching
336	336;363;167;	1.42		30	8	0	By MS/MS
830	830;938;938;	1.11		18	1	0	By MS/MS

416	416		3		7	0	By matching
453	453		3		2	0	By matching
1742	1742;763;16	1.88		9	7	0	By MS/MS
606	606	1.4		4	1	0	By MS/MS
467	467;467		3		5	0	By matching
551	551;551		3		1	0	By matching
231	231	2.08		34	40	0	By MS/MS
472	472;472;261;	2.07		71	82	0	By MS/MS
570	570		3		1	1	By matching
199	199	1.98		22	21	0	By MS/MS
207	207	2.67		3	15	0	By MS/MS
209	209		2	14	14	0	By MS/MS
169	169	2.14		9	12	0	By MS/MS
162	162	2.2		14	21	0	By MS/MS
607	607;454;523;	1.99		581	565	0	By MS/MS
230	230		1	3		0.0047651	By matching
564	564;526	2.17		48	68	0	By MS/MS
483	483;483;365;	1.59		29	12	0	By MS/MS
812	812;809;810;	2.25		107	178	0	By MS/MS
473	473;473;244;	2.42		20	49	0	By MS/MS
584	584;593;464;	1.95		198	179	0	By MS/MS
590	590;590;178;	1.8		50	33	0	By MS/MS
345	345		3		3	0.0007278	By matching
322	322;2416;25		2	3	3	0	By MS/MS
623	623;623;390	1.8		175	117	0	By MS/MS
645	645;119;102		1	2		0	By MS/MS
507	507;507;295;		3		12	0	By matching
432	432;432;433;	1.92		20	17	0	By MS/MS
416	416;416;416	2.81		3	28	0	By matching
597	597	1.64		19	9	0	By MS/MS
234	234	1.8		3	2	0	By MS/MS
1662	1662		2	3	3	0	By MS/MS
540	540;540;529;		2	5	5	0	By MS/MS
469	469;469;469;	1.13		28	2	0	By MS/MS
201	201		1	3		0	By MS/MS
455	455		3		4	0	By matching
2391	2391;2391	1.78		14	9	0	By MS/MS
535	535;535		1	1		0.00072464	By matching
601	601;580	2.14		3	4	1	By MS/MS
479	479		3		9	0	By matching
431	431;431;236;		3		1	0	By matching
452	452;452;452		2	6	6	0	By MS/MS
493	493;493;447;	2.85		4	49	0	By matching
520	520;521;521;	1.56		13	5	0	By matching
450	450;450;459;		3		1	1	By matching

578	578;578;578;	1.35		14	3	0	By matching
2850	2850;2850	1.79		38	25	0	By MS/MS
123	123;124;128;		3		8	0	By matching
513	513;513;513	2.33		1	2	0	By matching
249	249		1	31		0	By MS/MS
114	114		3		1	0	By matching
476	476		3		4	0	By matching
632	632;634;634;	1.95		33	30	0	By MS/MS
678	678;840;841;		1	27		0	By MS/MS
771	771;1060;10		1	9		0	By MS/MS
1017	1017;1017		1	3		0.0034388	By matching
1555	1555;1556;1	1.15		12	1	0	By MS/MS
254	254;274		2	3	3	0	By MS/MS
211	211;284;302;	1.82		10	7	0	By MS/MS
524	524;553;558;	2.09		15	18	0	By MS/MS
134	134;205;142;		1	6		0	By MS/MS
1365	1365;1443;1	1.8		9	6	0	By MS/MS
317	317;649;124;		3		9	0	By matching
290	290;314;314;	1.92		7	6	0	By MS/MS
294	294;310;118;	2.33		2	4	0	By matching
491	491;597;298;		1	13		0	By MS/MS
710	710;711;711;	1.86		12	9	0	By MS/MS
956	956;956;291;	1.93		8	7	0	By matching
2671	2671;2432;2		1	19		0	By MS/MS
924	924;942;960;	2.12		49	62	0	By MS/MS
312	312;312;218;	2.26		10	17	0	By MS/MS
89	89;90;122;12		1	3		0	By MS/MS
163	163;163;39;4	1.3		17	3	0	By MS/MS
132	132;132		3		1	0	By matching
135	135;135		2	5	5	0.0027855	By MS/MS
610	610;638;531;		1	8		0	By MS/MS
703	703;715;392;		1	13		0	By MS/MS
1028	1028;1029;4	2.06		23	26	0	By MS/MS
1488	1488;1414;9	2.33		3	6	0	By MS/MS
385	385;385;391;	1.76		21	13	0	By MS/MS
1152	1152;2297;2	1.88		9	7	0	By MS/MS
401	401;408;433;		2	3	3	0	By MS/MS
788	788;959;960;	1.42		15	4	0	By MS/MS
262	262;303;303		2	6	6	0	By matching
358	358;430;446;		1	3		0	By matching
325	325;327;361;	1.57		15	6	0	By MS/MS
50	50;67;81;84;	1.5		6	2	0	By MS/MS
106	106;108;148;	1.5		18	6	0	By MS/MS
102	102;1123;13		1	6		0.00073314	By MS/MS
59	59;98		2	3	3	0.0020994	By MS/MS

414	414;414		1	24			0	By MS/MS
372	372;382;397;		1	3			0	By MS/MS
537	537;47;95;16		3		3	0.002772		By matching
666	666;666	1.96		27	25		0	By MS/MS
3848	3848;3859;3	1.31		27	5		0	By MS/MS
44	44;45;48;100		3		6		0	By matching
148	148;149;155;		1	6			0	By MS/MS
599	599;630;631;	1.16		68	6		0	By MS/MS
1690	1690;1722;1		1	21			0	By MS/MS
962	962;1157		2	6	6		0	By MS/MS
124	124;225;706		1	3			0	By MS/MS
449	449;449;519;		1	13			0	By MS/MS
135	135;165;165		2	3	3		0	By MS/MS
2162	2162;2225;2		1	7			0	By MS/MS
1873	1873;1875;1	1.75		5	3		0	By MS/MS
131	131;133;137;		3		1	0.0098425		By matching
1271	1271;1463;1	1.72		100	56		0	By MS/MS
301	301;321;365;	1.76		13	8		0	By MS/MS
157	157;172		1	3			0	By matching
385	385;458;340;	1.23		47	6		0	By MS/MS
746	746;722;851	1.9		22	18		0	By MS/MS
103	103;185;375;		1	3			0	By matching
1337	1337;1337;3	2.23		18	29		0	By MS/MS
605	605;736;420;	1.26		20	3		0	By MS/MS
494	494;781		1	3			0	By MS/MS
68	68;193;244;2		1	3			0	By MS/MS
816	816;859;766;	1.63		72	33		0	By MS/MS
124	124;151;160;		1	14			0	By MS/MS
430	430;346;190;		2	3	3	0.004749		By MS/MS
218	218;319;359;		1	9			0	By MS/MS
449	449;472;429;	1.83		109	78		0	By MS/MS
255	255;255;384;		3		3		1	By matching
417	417;502;511;		1	10			0	By MS/MS
572	572;578;411;	1.17		32	3		0	By MS/MS
470	470;514;357;		1	12			0	By MS/MS
203	203;242;144;	2.2		12	18		0	By MS/MS
269	269;1015;10		1	2		0.0027778		By MS/MS
426	426;426;175;	1.67		6	3		0	By matching
195	195;293;233;	1.77		35	22		0	By MS/MS
196	196		1	1			1	By matching
1564	1564;2048		1	3			0	By MS/MS
137	137;170;112;	1.72		23	13		0	By MS/MS
111	111;115;42;8	1.57		5	2		0	By MS/MS
498	498;503;520;	2.48		12	34		0	By MS/MS
729	729;731;737;	1.99		79	77		0	By MS/MS

7388	7388;7555;7!	1.5		3	1	0.0047749	By matching
132	132;293		1	3		0	By MS/MS
162	162;278;361;		2	3	3	0	By MS/MS
1174	1174;1142		1	2		0	By matching
654	654;784;607;		1	20		0	By MS/MS
644	644;644;644;	1.83		236	169	0	By MS/MS
884	884;921;921		1	6		0	By MS/MS
952	952;960;267;	1.41		35	9	0	By MS/MS
415	415;427;298;	2.11		12	15	0	By MS/MS
180	180;111	1.67		12	6	0	By MS/MS
1454	1454		1	6		0	By MS/MS
306	306;306;361;	1.9		34	28	0	By MS/MS
226	226;499;876		3		3	0.00071174	By matching
753	753;1817;13	1.4		12	3	0	By MS/MS
291	291	2.12		11	14	0	By MS/MS
1755	1755;3038;1!		1	63		0	By MS/MS
105	105;105	1.97		18	17	0	By MS/MS
218	218;256;314		1	3		0	By MS/MS
296	296;314;314		1	7		0	By MS/MS
217	217;293		2	3	3	0	By MS/MS
368	368;804	1.8		3	2	0	By MS/MS
140	140;144;91		3		16	0	By matching
116	116;400		3		3	0.0020964	By matching
202	202;264;477;	1.92		32	27	0	By MS/MS
1145	1145;1162;1	1.5		36	12	0	By MS/MS
115	115;115		3		4	0.0007215	By matching
133	133;501;70;3		2	3	3	0	By MS/MS
148	148;151;116	1.4		24	6	0	By MS/MS
304	304;378		1	3		0	By MS/MS
482	482;485	1.67		6	3	0	By MS/MS
158	158;445		3		1	1	By matching
598	598;614;461;	1.51		26	9	0	By MS/MS
614	614;614;614;	1.9		133	108	0	By MS/MS
173	173;193;194;	1.8		9	6	0	By MS/MS
248	248;253;233;	2.04		39	42	0	By MS/MS
916	916;927;399;	1.86		8	6	0	By MS/MS
544	544;656;98;1		1	6		0.0098945	By matching
727	727;735;93	1.67		6	3	0	By MS/MS
478	478;493;496;	1.92		20	17	0	By MS/MS
512	512;521;340	1.77		48	30	0	By MS/MS
620	620;620;483		1	16		0	By MS/MS
167	167;464		1	6		0	By MS/MS
168	168;171;172;		1	19		0	By MS/MS
406	406;406;407;	1.94		17	15	0	By MS/MS
169	169;189;189;	1.76		13	8	0	By MS/MS

314	314;463	1	3		0	By MS/MS
426	426;458;480	3		3	0.0053945	By matching
204	204;222;222;	1.5	9	3	0	By MS/MS
181	181;220;40;1	1	10		0	By MS/MS
243	243;346	1	3		0	By MS/MS
585	585;638;732;	1	6		0	By MS/MS
110	110;132;220;	2	6	6	0	By MS/MS
968	968;832;165	1.39	126	31	0	By MS/MS
365	365;541;541;	1	6		0	By MS/MS
197	197;235;235	1.8	3	2	0	By MS/MS
126	126	2	3	3	0.0099404	By matching
2354	2354;2354	1	2		0	By matching
215	215;215;234;	1.44	21	6	0	By MS/MS
279	279;151;255	1	15		0	By MS/MS
560	560;60;90	1.07	87	3	0	By MS/MS
195	195;195	1	3		0.00070671	By matching
1710	1710;113;20	1.41	27	7	0	By MS/MS
5537	5537;145	1	6		0.0020979	By matching
1098	1098;1032;6	1.28	49	8	0	By MS/MS
403	403;347;403;	1.87	58	45	0	By MS/MS
1071	1071;718;78	1.22	24	3	0	By MS/MS
677	677;677	1	24		0	By MS/MS
338	338;346;140;	1.44	7	2	0	By MS/MS
1046	1046;920;18	1.3	34	6	0	By MS/MS
428	428	1	3		0	By MS/MS
456	456;306;140	2.19	21	31	0	By MS/MS
533	533;499;499;	1	6		0.00073529	By MS/MS
800	800;642;249;	1.41	23	6	0	By MS/MS
633	633;607;633;	1.96	50	46	0	By MS/MS
513	513;111;145;	3		6	0	By matching
514	514;131;212	1.86	8	6	0	By MS/MS
328	328;278;248;	2.03	32	34	0	By MS/MS
692	692;692;358;	1.06	60	2	0	By MS/MS
2271	2271	2.25	9	15	0	By MS/MS
312	312;316;338	1	2		0.00070721	By matching
453	453;623;534;	1.94	33	29	0	By MS/MS
514	514;258;313;	1.62	42	19	0	By MS/MS
1171	1171	1	12		0	By MS/MS
1162	1162;720;30	1	33		0	By MS/MS
330	330	1.77	8	5	0	By MS/MS
191	191;191;121;	2.38	4	9	0	By MS/MS
372	372;192;200;	1.59	12	5	0	By MS/MS
957	957;816;346;	1.93	23	20	0	By MS/MS
220	220	1.5	9	3	0	By MS/MS
530	530;263;50	2.3	16	30	0	By MS/MS

172	172;262;262;	2.25		6	10	0	By MS/MS
1304	1304;496;50	1.8		39	26	0	By MS/MS
178	178		2	8	8	0	By MS/MS
2136	2136;1887;7	1.46		37	11	0	By MS/MS
2785	2785;541	1.12		49	3	0	By MS/MS
238	238	2.14		12	16	0	By MS/MS
250	250		3		4	0	By matching
1766	1766		1	3		0	By MS/MS
888	888;603		1	5		0	By matching
941	941	1.29		36	6	0	By MS/MS
2799	2799;2792;1		1	41		0	By MS/MS
198	198;198		3		6	0	By matching
890	890;159;225	1.67		46	23	0	By MS/MS
335	335		2	3	3	0.0047782	By matching
1232	1232;1235;5	1.84		46	33	0	By MS/MS
580	580;52	1.96		27	25	0	By MS/MS
989	989		3		2	0	By matching
584	584		1	3		0.00072046	By matching
1268	1268		3		3	0.00073746	By matching
269	269;191;211;		1	9		0	By MS/MS
575	575;575		1	3		0	By MS/MS
613	613;225;226;		1	12		0	By MS/MS
429	429;429;429;	1.62		27	12	0	By MS/MS
1365	1365;568;16	1.82		23	16	0	By MS/MS
218	218;218;65		1	5		0.0007326	By MS/MS
664	664;572;480;	1.86		201	152	0	By MS/MS
132	132		2	9	9	0	By MS/MS
444	444;362;409;		1	3		0	By MS/MS
335	335;335;335;	1.97		18	17	0	By MS/MS
205	205;205;170;	1.48		19	6	0	By MS/MS
607	607;607;607;	1.1		39	2	0	By MS/MS
1023	1023;1020;9		1	25		0	By MS/MS
93	93		3		3	0.0060443	By matching
298	298;323	1.92		46	39	0	By MS/MS
445	445;529;529;	1.77		19	12	0	By MS/MS
294	294;274;228;	1.92		71	60	0	By MS/MS
426	426;444;444;	1.62		36	16	0	By MS/MS
1512	1512;476;86		1	17		0	By MS/MS
85	85;91		1	3		0	By MS/MS
785	785;563;299;	1.57		15	6	0	By MS/MS
466	466;466;431;	1.23		85	11	0	By MS/MS
135	135;38;31	2.06		29	33	0	By MS/MS
255	255;255;255;	2.46		23	62	0	By MS/MS
128	128;128;122;	1.29		6	1	0	By MS/MS
603	603;604		3		4	0	By matching

290	290;306;725;		1	3		0.00070572	By MS/MS
654	654;654;278	1.25		201	29	0	By MS/MS
646	646;646;627;	1.51		183	62	0	By MS/MS
1531	1531;147	1.56		135	52	0	By MS/MS
157	157;180		1	6		0	By MS/MS
298	298;298;323;	1.8		12	8	0	By MS/MS
146	146		2	3	3	0	By MS/MS
568	568;732	1.57		15	6	0	By MS/MS
858	858;566;517;	1.7		28	15	0	By MS/MS
572	572;589;558;	2.04		76	82	0	By MS/MS
2871	2871;2319;1	2.01		144	146	0	By MS/MS
226	226;59	1.97		32	30	0	By MS/MS
213	213	2.03		38	40	0	By MS/MS
1042	1042;844;99		1	18		0	By MS/MS
347	347;347;347	1.67		6	3	0	By MS/MS
347	347;166		2	6	6	0	By matching
86	86;137;345		1	3		0	By MS/MS
591	591;435;497;	1.62		42	19	0	By MS/MS
556	556;401;433;		1	30		0	By MS/MS
110	110;55;57;94	1.76		13	8	0	By MS/MS
2426	2426;454;45	1.07		114	4	0	By MS/MS
350	350;152;230;		1	12		0	By MS/MS
586	586;387;329;	1.53		99	36	0	By MS/MS
2607	2607;2647;2	1.6		66	28	0	By MS/MS
280	280;123;200	1.8		3	2	0.0047684	By matching
260	260;321;244;	1.58		51	21	0	By MS/MS
979	979;104;124;		1	16		0	By MS/MS
634	634;707;707;	2.08		12	14	0	By matching
451	451;451;135;	1.4		12	3	0	By MS/MS
208	208;216		3		9	0	By matching
243	243;243;231;	1.68		29	15	0	By MS/MS
166	166;166;149;	1.88		19	15	0	By MS/MS
132	132	1.65		21	10	0	By MS/MS
553	553;553;207;	1.72		25	14	0	By MS/MS
475	475;471;307		2	39	39	0	By MS/MS
101	101		3		3	0.0066756	By matching
531	531;527;197;	2.07		52	60	0	By MS/MS
214	214;200;214;	1.89		51	41	0	By MS/MS
592	592;134;144;		1	15		0	By MS/MS
533	533;533;347;	1.62		18	8	0	By MS/MS
64	64;98		3		2	0.0099206	By matching
298	298;298;251	2.2		33	49	0	By MS/MS
165	165;197;94;1	1.82		20	14	0	By MS/MS
101	101		3		6	0	By matching
330	330		1	8		0	By MS/MS

397	397;240;258		1	75		0	By MS/MS
248	248	2.45		3	8	0	By matching
146	146;51		3		13	0	By matching
857	857		1	28		0	By MS/MS
326	326;469;326;		1	56		0	By MS/MS
354	354;320;354;	1.57		25	10	0	By MS/MS
1148	1148;521;18	1.53		36	13	0	By MS/MS
375	375;347;351;	2.24		29	47	0	By MS/MS
639	639	1.95		186	167	0	By MS/MS
291	291;298;250;		1	13		0	By MS/MS
427	427;441;414;	1.69		61	32	0	By MS/MS
136	136;115;56	1.97		18	17	0	By MS/MS
391	391;296;352;	1.91		41	34	0	By MS/MS
576	576;37	2.11		24	30	0	By MS/MS
403	403;374;403;	1.77		46	29	0	By MS/MS
1505	1505;484;67	1.67		12	6	0	By MS/MS
203	203;203;201;	1.92		20	17	0	By MS/MS
512	512		1	6		0	By MS/MS
172	172		1	3		0.00071839	By MS/MS
694	694	1.92		115	97	0	By MS/MS
1042	1042;1042;9	1.23		23	3	0	By MS/MS
84	84;52;66;84;	1.67		6	3	0	By MS/MS
1498	1498;1084;5		1	38		0	By MS/MS
934	934;810;865;	1.71		44	24	0	By MS/MS
303	303	2.07		31	36	0	By MS/MS
160	160;160;87;1	1.63		13	6	0	By MS/MS
165	165;174;172;	1.8		9	6	0	By MS/MS
325	325;122;176;		1	12		0	By MS/MS
167	167;235;107		1	6		0	By MS/MS
749	749		1	3		0	By matching
2511	2511	1.31		11	2	0	By MS/MS
452	452;101		1	21		0	By MS/MS
3210	3210;175;17		1	7		0	By MS/MS
86	86;45;49;52;	2.11		12	15	0	By MS/MS
416	416;416;416;	2.33		3	6	0	By MS/MS
727	727		2	3	3	0.0099338	By matching
1951	1951;1440;1	1.26		27	4	0	By MS/MS
843	843;243;85;7	1.87		39	30	0	By MS/MS
3224	3224;1758;1	2.1		74	90	0	By MS/MS
254	254;219;21	1.24		36	5	0	By MS/MS
45	45;106;140;2		1	2		0.0060484	By MS/MS
173	173;148		1	14		0	By MS/MS
3418	3418;2649;7		1	92		0	By MS/MS
240	240;240;208;	2.13		10	13	0	By MS/MS
378	378;139	2.16		48	66	0	By MS/MS

730	730;730;615;	1.48		174	55	0	By MS/MS
156	156;54;83;86	2.33		3	6	0	By MS/MS
1360	1360;1067;1		1	64		0	By MS/MS
324	324;100;100;		1	24		0	By MS/MS
1224	1224		1	3		0	By matching
127	127;127;134;	2.25		6	10	0	By MS/MS
660	660		1	3		0	By matching
103	103		2	3	3	0	By matching
806	806;806;644;		1	28		0	By MS/MS
439	439	2.2		8	12	0	By MS/MS
1226	1226;1226;8	2.03		117	125	0	By MS/MS
449	449;404;355	2.11		17	21	0	By MS/MS
245	245;108;59;1	1.85		15	11	0	By MS/MS
925	925		1	3		0	By matching
376	376;376;386;	2.25		3	5	0	By MS/MS
94	94;94;94		3		4	0.0027739	By matching
96	96		1	3		0.00071788	By MS/MS
375	375;375;375;		2	2	2	0	By MS/MS
119	119;61;46;64	1.75		15	9	0	By MS/MS
145	145;145;107;	1.43		29	8	0	By MS/MS
334	334;96;56	1.33		35	7	0	By MS/MS
330	330;253;299;	1.5		9	3	0	By MS/MS
208	208;208;188;	1.87		30	23	0	By MS/MS
151	151;120;134;	1.33		30	6	0	By MS/MS
152	152;82;33;34	1.47		29	9	0	By MS/MS
56	56		2	3	3	0	By MS/MS
158	158;118;79;8	1.76		36	22	0	By MS/MS
86	86		3		5	0	By matching
119	119;44;75;11	1.83		7	5	0	By MS/MS
118	118;78;54		2	9	9	0	By MS/MS
126	126;62	2.1		9	11	0	By matching
266	266;191;142;	1.67		46	23	0	By MS/MS
177	177;54	2.02		20	21	0	By MS/MS
156	156;158;170;	1.68		29	15	0	By MS/MS
125	125		1	11		0	By MS/MS
108	108		1	2		0.0073138	By MS/MS
51	51;51	1.8		3	2	0	By MS/MS
217	217;206;133	1.94		27	24	0	By MS/MS
178	178;178;177;	1.88		24	19	0	By MS/MS
257	257;188;205;	1.64		38	18	0	By MS/MS
128	128;128;93;1		2	3	3	0.00073584	By MS/MS
380	380;394;103	1.91		24	20	0	By MS/MS
447	447;447;453;		1	9		0	By MS/MS
89	89;89;47;67;	1.86		21	16	0	By MS/MS
81	81;83;83;90	2.14		3	4	0	By MS/MS

375	375;356;333;	1.89		77	62	0	By MS/MS
324	324;320;374;	1.29		18	3	0	By MS/MS
462	462;462;462;	1.73		85	49	0	By MS/MS
448	448;153;180;	1.54		116	43	0	By MS/MS
445	445;445;445;	1.5		151	50	0	By MS/MS
168	168;283;145		1	6		0.00073206	By MS/MS
268	268;212;220;	1.43		22	6	0	By MS/MS
352	352;368;437;	1.36		18	4	0	By MS/MS
519	519		1	6		0	By MS/MS
4128	4128;1130;7(	1.69		468	244	0	By MS/MS
227	227		3		3	0	By matching
110	110	1.92		7	6	0	By MS/MS
210	210;150;213;		3		11	0	By matching
825	825;750;784;	1.97		75	71	0	By MS/MS
240	240;240;220;	2.17		25	35	0	By MS/MS
453	453;256;389;		1	19		0	By MS/MS
507	507;71;241;5	1.46		20	6	0	By MS/MS
293	293;338;338;	1.8		6	4	0	By MS/MS
885	885;431;529;	1.86		21	16	0	By MS/MS
1049	1049	1.89		20	16	0	By MS/MS
1598	1598;1626;1:	1.82		82	57	0	By MS/MS
3969	3969;1439;3:	1.13		87	6	0	By MS/MS
246	246;247;321;	2.33		3	6	0	By MS/MS
463	463;340		1	9		0	By MS/MS
427	427;508;396;	1.65		29	14	0	By MS/MS
500	500;425;379;	1.74		12	7	0	By MS/MS
406	406;282;377;	1.37		76	17	0	By MS/MS
199	199;171;199;	2.08		45	53	0	By MS/MS
177	177;177;121;		3		4	0	By matching
1270	1270;596;59!	2.07		20	23	0	By MS/MS
840	840;894	2.17		5	7	0	By MS/MS
425	425;207;173;	1.74		36	21	0	By MS/MS
5890	5890;1079;7!	1.15		36	3	0	By MS/MS
2109	2109;2109;2:	1.66		41	20	0	By MS/MS
3046	3046;2764;2/		1	67		0	By MS/MS
951	951;636;121;	2.14		6	8	0	By MS/MS
2000	2000;979;66:	1.57		15	6	0	By MS/MS
444	444;379		1	14		0	By MS/MS
305	305	1.92		21	18	0	By MS/MS
835	835;835;162;		1	50		0	By MS/MS
861	861;861;856;	2.28		32	57	0	By MS/MS
1488	1488;712	2.16		46	63	0	By MS/MS
450	450;406;450;		1	18		0	By MS/MS
2644	2644;39;80;4		1	20		0	By MS/MS
536	536;461;461;	1.75		33	20	0	By MS/MS

282	282;282	2.12		15	19	0	By MS/MS
1172	1172;579;18		3		14	0	By matching
415	415;429;322;		1	18		0	By MS/MS
731	731;731;233;	1.18		41	4	0	By MS/MS
445	445;445;373;		1	6		0	By MS/MS
953	953;526;201	2.38		17	38	0	By MS/MS
317	317;155;216		1	17		0	By MS/MS
630	630;644;422	2.2		6	9	0	By MS/MS
258	258;116;75;1	2.16		8	11	0	By MS/MS
1141	1141;1112;1		1	6		0.00072202	By matching
530	530;423;502;	1.76		88	54	0	By MS/MS
448	448;448		3		1	0	By matching
1992	1992;132;18	1.78		66	42	0	By MS/MS
2120	2120		1	3		0.0098684	By matching
2089	2089;1655;1	2.31		18	34	0	By MS/MS
625	625		1	6		0	By MS/MS
1233	1233;1211;8	1.27		85	13	0	By MS/MS
758	758;408	2.13		66	86	0	By MS/MS
1871	1871;771;12	1.91		18	15	0	By MS/MS
1282	1282	1.67		4	2	0	By matching
532	532		1	297		0	By MS/MS
665	665;597;611;	1.86		54	41	0	By MS/MS
1188	1188		1	11		0	By MS/MS
1978	1978;1578;1	1.38		63	15	0	By MS/MS
2115	2115;2121;1	1.33		5	1	0	By MS/MS
365	365	1.91		6	5	0	By MS/MS
1648	1648		1	3		0.0098619	By MS/MS
677	677		2	3	3	0	By MS/MS
292	292;417;486		1	17		0	By MS/MS
4684	4684;2105;1	1.04		48	1	0	By MS/MS
356	356;356;64;3	1.96		50	46	0	By MS/MS
476	476;165;257;		2	3	3	0.00072254	By MS/MS
326	326;153;133;	2.11		57	71	0	By MS/MS
425	425;425;416;	1.63		26	12	0	By MS/MS
458	458;199;79;1	1.97		15	14	0	By MS/MS
747	747;746	1.48		19	6	0	By MS/MS
1367	1367;1412;6		1	17		0	By MS/MS
325	325;371;371;	2.18		9	13	0	By MS/MS
1735	1735;724;78		1	141		0	By MS/MS
807	807;845;532;	2.22		16	25	0	By MS/MS
163	163;163		3		7	0	By matching
444	444;407;410		1	12		0	By MS/MS
716	716;467;47;7	2.64		2	9	0	By MS/MS
172	172;175		3		5	0	By matching
642	642		1	3		0.0060606	By MS/MS

595	595;595	2.08		6	7	0	By MS/MS
435	435;439;439;		1	3		0	By matching
342	342;342;127	1.25		21	3	0	By MS/MS
271	271;271;160		1	9		0	By MS/MS
601	601;176;133;	1.69		19	10	0	By MS/MS
1105	1105;1105;8:	1.56		26	10	0	By MS/MS
1244	1244;970		2	3	3	0	By MS/MS
709	709		1	3		0	By MS/MS
190	190;212;190;	2.28		29	52	0	By MS/MS
582	582;493;53	1.74		44	26	0	By MS/MS
860	860		1	18		0	By MS/MS
169	169;261		3		3	0.0054054	By matching
1711	1711		1	3		0	By MS/MS
756	756;601;601;		1	15		0	By MS/MS
272	272	2.05		9	10	0	By MS/MS
666	666;552;166		1	9		0	By MS/MS
1749	1749;853;10		1	13		0	By MS/MS
327	327;553;253;		3		13	0	By matching
1142	1142;1123;4		1	33		0	By MS/MS
702	702	1.27		39	6	0	By MS/MS
376	376;388;388;	1.9		23	19	0	By MS/MS
446	446;446;370;	1.23		46	6	0	By MS/MS
769	769		1	9		0	By MS/MS
356	356		3		3	0	By matching
828	828		1	9		0	By MS/MS
855	855;875		1	6		0	By matching
579	579	1.67		30	15	0	By MS/MS
250	250	2.09		5	6	0	By matching
190	190;190		3		1	0	By matching
943	943;943;626		3		6	0	By matching
136	136		2	9	9	0	By MS/MS
1603	1603	1.4		20	5	0	By MS/MS
2151	2151;675;20		1	18		0	By MS/MS
2472	2472;746;10	1.5		155	52	0	By MS/MS
546	546;435;93	2.31		12	23	0	By MS/MS
2279	2279		1	23		0	By MS/MS
579	579		1	3		0.00072307	By MS/MS
2430	2430;752	1.04		54	1	0	By MS/MS
1548	1548;1207		1	41		0	By MS/MS
401	401;401;212;	2.2		6	9	0	By MS/MS
726	726		1	22		0	By MS/MS
2403	2403;2506;2		1	16		0	By MS/MS
851	851;122		1	12		0	By MS/MS
747	747;747;605;	1.84		133	96	0	By MS/MS
1835	1835;1445;1	1.19		28	3	0	By MS/MS

1023	1023;1008;1(	1.07		83	3	0	By MS/MS
437	437;438;173		1	9		0	By MS/MS
508	508;508;235;	1.53		14	5	0	By MS/MS
451	451		3		3	0.00073855	By matching
567	567;400;431;		1	15		0	By MS/MS
707	707;469;185;		1	30		0	By MS/MS
1132	1132;496;78;		1	17		0	By MS/MS
2335	2335;1065;3!	1.73		42	24	0	By MS/MS
1023	1023;473;20		1	18		0	By MS/MS
442	442		1	3		0	By MS/MS
385	385;328;200;		1	14		0	By MS/MS
2663	2663		1	3		0	By MS/MS
594	594;849;221	1.25		21	3	0	By MS/MS
590	590	1.96		12	11	0	By MS/MS
136	136		1	2		0.0099075	By matching
377	377		3		6	0.0054164	By matching
416	416	1.67		6	3	0	By MS/MS
300	300;539		1	5		0	By MS/MS
475	475		3		11	0	By matching
434	434;203;176;	1.67		6	3	0	By MS/MS
1910	1910;214		1	37		0	By MS/MS
2677	2677;1776;9	1.17		33	3	0	By MS/MS
1410	1410;109;77	1.25		7	1	0	By MS/MS
1322	1322;1224;1		1	53		0	By MS/MS
1906	1906;942;65	1.5		18	6	0	By MS/MS
1792	1792;138;30(	2.4		3	7	0	By MS/MS
4022	4022		1	3		0.0079576	By MS/MS
256	256		1	3		0.0060403	By matching
1040	1040		3		5	0	By matching
641	641;464;464;		1	3		0.0021097	By matching
420	420;333		1	6		0	By MS/MS
306	306;168;178;	1.8		9	6	0	By MS/MS
1689	1689;1460;1(	1.3		52	9	0	By MS/MS
332	332;194;148;	2.33		12	24	0	By MS/MS
257	257;264	1.85		23	17	0	By MS/MS
214	214;172;137;	2.23		15	24	0	By MS/MS
1186	1186;891		1	12		0	By MS/MS
465	465;108;112		3		6	0.00072727	By matching
679	679	1.51		35	12	0	By MS/MS
445	445;236;155;		1	3		0	By MS/MS
2542	2542;1486;1!		1	12		0	By MS/MS
793	793;332;275;	1.97		104	98	0	By MS/MS
180	180;56;67;72		1	6		0	By MS/MS
847	847;726	1.6		21	9	0	By MS/MS
903	903;36;57	1.5		6	2	0	By MS/MS

243	243		1	11		0	By MS/MS
3477	3477;1277;1		1	36		0	By MS/MS
55	55;73;55		3		1	1	By matching
819	819;819;880;	1.18		20	2	0	By MS/MS
730	730;413;435;	1.88		18	14	0	By MS/MS
415	415;402;208;	1.45		17	5	0	By MS/MS
163	163		1	6		0.00072411	By MS/MS
521	521;137;196;		1	9		0	By MS/MS
132	132	1.22		24	3	0	By MS/MS
585	585;379		1	5		0	By MS/MS
562	562	1.6		7	3	0	By MS/MS
312	312		2	6	6	0	By MS/MS
471	471;102;211;	2.65		3	14	0	By matching
822	822		1	3		0.00073368	By MS/MS
893	893;127;143;	1.4		12	3	0	By MS/MS
393	393;189	2.33		3	6	0	By matching
408	408;387;387;	2.07		14	16	0	By MS/MS
748	748;570;268		1	10		0	By MS/MS
708	708;79	1.89		21	17	0	By MS/MS
983	983;983;828;		1	108		0	By MS/MS
193	193	1.97		16	15	0	By MS/MS
628	628		1	3		0	By matching
3051	3051		3		3	0.00073638	By matching
2342	2342;1746;1		1	83		0	By MS/MS
2715	2715;1014;5		1	51		0	By MS/MS
881	881;160;208	2.17		12	17	0	By MS/MS
353	353;335;177;	1.98		28	27	0	By MS/MS
4624	4624	1.5		6	2	0.0027586	By MS/MS
1887	1887		1	6		0	By MS/MS
911	911;377;420;	1.67		8	4	0	By MS/MS
451	451;498;330;		1	21		0	By MS/MS
269	269		1	15		0	By MS/MS
1383	1383;1106;1	2.06		54	61	0	By MS/MS
593	593;593	1.64		15	7	0	By MS/MS
2266	2266;1322;9	1.18		296	30	0	By MS/MS
427	427;91;93;16	2.33		3	6	0.00073801	By MS/MS
213	213		2	15	15	0	By MS/MS
2039	2039;1072;8	1.74		68	40	0	By MS/MS
488	488;458;100;	1.28		37	6	0	By MS/MS
1312	1312;660;72	2.23		10	16	0	By MS/MS
711	711;711;279;	2.1		116	143	0	By MS/MS
2177	2177;2000;1		1	11		0	By MS/MS
163	163		2	3	3	0	By MS/MS
307	307		1	3		0	By MS/MS
570	570		2	3	3	0.0027567	By matching

335	335		1	6		0	By MS/MS
904	904		1	11		0	By MS/MS
314	314;185		1	11		0	By MS/MS
1024	1024;1024;1	2.26		21	36	0	By MS/MS
103	103		3		8	0	By matching
344	344;172;228;	1.81		16	11	0	By MS/MS
510	510;262;234	2.08		39	46	0	By MS/MS
300	300	2.29		6	11	0	By matching
1249	1249;1229;1		1	18		0	By MS/MS
289	289;140		1	27		0	By MS/MS
970	970		1	3		0	By MS/MS
340	340		2	3	3	0	By MS/MS
812	812;145	1.64		17	8	0	By MS/MS
335	335;627;327;		1	6		0	By MS/MS
892	892	1.57		10	4	0	By MS/MS
3159	3159;1731;3		1	43		0	By MS/MS
304	304		1	6		0	By MS/MS
669	669;552;213;	1.68		86	44	0	By MS/MS
802	802;136;323		3		6	0.0079734	By matching
341	341;47;100;1		1	6		0	By matching
1844	1844;1224;1	1.09		66	3	0	By MS/MS
561	561;56;189		1	9		0	By MS/MS
496	496;44;68;13	1.24		15	2	0	By MS/MS
1273	1273;378	1.26		20	3	0	By MS/MS
3664	3664;297;37		1	44		0	By MS/MS
336	336;245	1.12		130	8	0	By MS/MS
860	860;150;311;		1	12		0	By MS/MS
359	359		1	3		0	By matching
176	176;176;150;	2.17		15	21	0	By MS/MS
325	325	2.14		6	8	0	By MS/MS
1328	1328;1252;3	1.84		114	83	0	By MS/MS
585	585;589;589;	1.74		29	17	0	By MS/MS
211	211;198;211;	2.09		32	38	0	By MS/MS
358	358;132;209	2.23		15	24	0	By MS/MS
2240	2240	1.39		75	18	0	By MS/MS
118	118	1.8		3	2	0	By matching
263	263	2.11		25	31	0	By MS/MS
446	446;418;338;		1	18		0	By MS/MS
394	394;432;409;	1.35		28	6	0	By MS/MS
771	771;717;320;		1	27		0	By MS/MS
753	753;258		1	16		0	By MS/MS
364	364;240;364;	1.97		18	17	0	By MS/MS
86	86	1.4		12	3	0	By MS/MS
252	252;211;115	1.67		10	5	0	By MS/MS
306	306;230;122		3		3	0	By matching

1806	1806		1	57		0	By MS/MS
327	327;93	2.36		9	19	0	By MS/MS
350	350		1	6		0	By MS/MS
92	92;494;501		1	6		0	By MS/MS
559	559;395;509;	1.75		5	3	0	By matching
2564	2564;1340;1		1	78		0	By MS/MS
1437	1437;282;77		1	29		0	By MS/MS
99	99;120	1.5		9	3	0	By MS/MS
481	481;349;141;	2.24		8	13	0	By MS/MS
1025	1025;834;28	1.31		27	5	0	By MS/MS
392	392;203;212;	2.2		2	3	0.0034341	By MS/MS
329	329;232;280;	1.21		25	3	0	By MS/MS
1944	1944;1452;1		1	6		0	By MS/MS
86	86;88;89;124		2	18	18	0	By MS/MS
574	574;540;177;	1.37		35	8	0	By MS/MS
1342	1342;1121		1	9		0	By MS/MS
851	851;456;552;	1.5		6	2	0	By MS/MS
1327	1327;1326;6	1.01		160	1	0	By MS/MS
222	222;222	1.91		6	5	0	By MS/MS
360	360	1.91		6	5	0	By MS/MS
1146	1146;1008;1	2.42		33	81	0	By MS/MS
229	229	1.88		9	7	0	By MS/MS
328	328		1	3		0	By matching
458	458		1	3		0.0054201	By MS/MS
597	597;189;92;1		1	32		0	By MS/MS
1298	1298;803;36	1.07		155	6	0	By MS/MS
353	353	2.16		19	26	0	By MS/MS
442	442;187;32;8	2.4		3	7	0	By MS/MS
389	389;242;175;	1.38		84	20	0	By MS/MS
1990	1990;1100;1		1	9		0	By MS/MS
577	577;577;95;1		1	17		0	By MS/MS
2581	2581;873		1	2		0	By MS/MS
291	291;291;284	2.14		6	8	0	By MS/MS
245	245;156;261		2	10	10	0	By MS/MS
918	918		1	3		0.0066667	By matching
275	275;79		2	6	6	0	By MS/MS
457	457;173;195;		1	4		0.00071531	By MS/MS
783	783;82;145	2.07		106	122	0	By MS/MS
2969	2969;177		1	42		0	By MS/MS
1034	1034		1	3		0.0027624	By MS/MS
230	230		1	6		0	By MS/MS
5596	5596;1181;5		1	100		0	By MS/MS
292	292;280;238;	2.45		8	21	0	By MS/MS
204	204	1.75		5	3	0	By matching
582	582	2.4		3	7	0	By matching

624	624		1	9		0	By MS/MS
1034	1034;453;80(	1.74		17	10	0	By MS/MS
392	392		2	2	2	0	By MS/MS
580	580;325;126;		3		14	0	By matching
375	375;409;60;1		1	9		0	By MS/MS
385	385		1	3		0.0021067	By MS/MS
560	560;98;154		1	15		0	By MS/MS
926	926		1	18		0	By MS/MS
920	920;871;725;	1.86		68	51	0	By MS/MS
233	233		1	5		0	By MS/MS
1118	1118;495	1.67		6	3	0	By MS/MS
146	146;146	2.33		3	6	0	By MS/MS
644	644;142;123;	1.09		43	2	0	By MS/MS
711	711;552;553;	1.74		17	10	0	By MS/MS
633	633;173;98;1		1	12		0	By MS/MS
358	358;184		1	5		0	By MS/MS
284	284	2.08		12	14	0	By MS/MS
869	869		1	6		0	By MS/MS
647	647;179;245;	1.97		19	18	0	By MS/MS
2413	2413		3		1	0.0047847	By matching
1281	1281;271;90:		1	6		0	By MS/MS
511	511		3		3	0.0027663	By matching
1483	1483	1.54		41	15	0	By MS/MS
1670	1670	1.06		62	2	0	By MS/MS
1502	1502;221		1	21		0	By MS/MS
822	822;567;149;	1.7		15	8	0	By MS/MS
1215	1215		1	21		0	By MS/MS
646	646;386;431;		1	38		0	By MS/MS
504	504;252;45;1	1.79		55	36	0	By MS/MS
2752	2752;1791;2:	1.92		100	85	0	By MS/MS
463	463;429;354;	1.67		68	34	0	By MS/MS
598	598		1	2		0.0027605	By matching
2266	2266;145		1	24		0	By MS/MS
713	713;644		1	11		0	By MS/MS
599	599;512;413;	2.04		45	49	0	By MS/MS
368	368;350;95	1.8		36	24	0	By MS/MS
955	955;373;373;	1.91		71	59	0	By MS/MS
529	529;466;145;	1.67		70	35	0	By MS/MS
895	895;600;462;		1	96		0	By MS/MS
392	392;392;389;	1.94		51	45	0	By MS/MS
253	253		1	3		0	By MS/MS
280	280;103;160	2.4		12	28	0	By MS/MS
125	125	1.67		6	3	0	By MS/MS
505	505;156;175;	1.91		6	5	0	By MS/MS
105	105;94	1.67		12	6	0	By MS/MS

223	223;248;76;1	2.2		6	9	0	By MS/MS
797	797;197;212;	2.29		18	33	0	By MS/MS
1905	1905;509;65		1	77		0	By MS/MS
1061	1061;1041;1	1.8		9	6	0	By MS/MS
734	734;197;295;	1.39		62	15	0	By MS/MS
1047	1047;398;66;	1.6		70	30	0	By MS/MS
519	519;345;412;	1.64		15	7	0	By MS/MS
886	886;38;148;4	1.57		15	6	0	By MS/MS
193	193		2	1	1	0.0034364	By matching
226	226;226	2.09		5	6	0.0014114	By matching
392	392;404;404;		3		1	0.0099469	By matching
2529	2529;2699		1	4		1	By matching
326	326		3		4	1	By matching
887	887	2.09		5	6	0.0073187	By MS/MS
1136	1136;1136;1		2	3	3	0.0034412	By matching
336	336;404;452;		3		4	0.0060647	By matching
211	211;291;329;		1	1		0.0021171	By matching
305	305;305	1.8		6	4	0.0047912	By MS/MS
188	188;222;257		3		1	0.00074074	By matching
1340	1340;1722		1	5		0.0027875	By MS/MS
79	79		1	1		0.0067069	By MS/MS
644	644;713		3		4	0.0041152	By matching
215	215		1	3		0.0079787	By MS/MS
989	989	1.91		6	5	0.0086207	By MS/MS
571	571	1.91		6	5	0.0054274	By MS/MS
2028	2028		2	6	6	0.0092838	By MS/MS
73	73		1	5		0	By MS/MS
1283	1283;1301;1	2.15		26	35	0	By MS/MS
172	172;153;143;	1.9		17	14	0	By MS/MS

Identificatior	Identificatior	Identificatior	Identificatior	Identificatior	Sequence co	Sequence co
By MS/MS	By MS/MS	By MS/MS	By MS/MS	By MS/MS	51.1	51.1
By MS/MS	By MS/MS	By MS/MS	By MS/MS	By MS/MS	26.4	26.4
By MS/MS	By MS/MS	By MS/MS	By matching	By matching	17.8	17.8
By MS/MS	By MS/MS	By MS/MS	By MS/MS	By MS/MS	10.8	10.8
By MS/MS	By MS/MS	By MS/MS	By MS/MS	By MS/MS	16.8	16.8
By MS/MS	By MS/MS	By matching	By matching	By MS/MS	4.4	4.4
By MS/MS	By matching	By matching	By matching	By matching	4.8	4.8
By MS/MS	By MS/MS	By MS/MS	By MS/MS	By MS/MS	44.5	36.6
By MS/MS	By MS/MS	By MS/MS	By MS/MS	By MS/MS	15.6	15.6
By matching	By MS/MS	By matching	By matching	By matching	5.6	2.5
By MS/MS	By MS/MS	By MS/MS	By MS/MS	By MS/MS	5.7	5.7
By MS/MS	By MS/MS	By MS/MS	By MS/MS	By matching	8.8	8.8
By MS/MS	By MS/MS	By MS/MS	By MS/MS	By MS/MS	16.2	16.2
By matching	By MS/MS	By MS/MS	By matching	By matching	1.4	1.4
By MS/MS	By MS/MS	By matching	By matching	By matching	13.4	13.4
By matching	By matching	By matching	By matching	By matching	7.8	0
By MS/MS	By MS/MS	By MS/MS	By MS/MS	By MS/MS	28.8	19.5
By matching	By matching	By matching	By matching	By matching	12	0
By MS/MS	By MS/MS	By MS/MS	By MS/MS	By MS/MS	23.7	20.9
By MS/MS	By MS/MS	By MS/MS	By MS/MS	By MS/MS	6.1	6.3
By MS/MS	By MS/MS	By MS/MS	By MS/MS	By MS/MS	41.3	41.3
By MS/MS	By matching	By matching	By matching	By MS/MS	35.4	35.4
By MS/MS	By MS/MS	By MS/MS	By MS/MS	By MS/MS	17	17
By MS/MS	By MS/MS	By matching	By matching	By matching	10.6	10.6
By MS/MS	By MS/MS	By matching	By matching	By matching	23.3	20
By matching	By MS/MS	By MS/MS	By MS/MS	By MS/MS	6.8	6.8
By MS/MS	By MS/MS	By MS/MS	By matching	By MS/MS	20.5	18.5
By matching	By matching	By matching	By matching	By matching	13.4	13.4
By MS/MS	By MS/MS	By matching	By matching	By MS/MS	8.6	11.5
By MS/MS	By MS/MS	By matching	By matching	By matching	28.3	28.3
By MS/MS	By MS/MS	By MS/MS	By matching	By matching	29.5	26.6
By matching	By matching	By matching	By matching	By matching	11.1	11.1
By matching	By MS/MS	By matching	By matching	By matching	4.7	2.5
By MS/MS	By MS/MS	By MS/MS	By matching	By MS/MS	3.9	3.9
By MS/MS	By MS/MS	By MS/MS	By MS/MS	By MS/MS	2.5	3.9
By matching	By matching	By matching	By matching	By matching	4.7	4.7
By MS/MS	By MS/MS	By MS/MS	By MS/MS	By MS/MS	14.7	14.7
By MS/MS	By MS/MS	By matching	By matching	By matching	4.2	4.2
By matching	By matching	By matching	By matching	By matching	16.2	16.2
By MS/MS	By MS/MS	By MS/MS	By matching	By MS/MS	9.2	9.2
By MS/MS	By MS/MS	By matching	By matching	By matching	1.2	1.2
By MS/MS	By MS/MS	By matching	By matching	By matching	14.9	15.8
By matching	By matching	By matching	By matching	By MS/MS	3.8	3.8
By MS/MS	By MS/MS	By MS/MS	By MS/MS	By MS/MS	5.1	5.1

By MS/MS	By MS/MS	By MS/MS	By MS/MS	By MS/MS	10.8	10.8
By MS/MS	By MS/MS	By MS/MS	By MS/MS	By MS/MS	2.2	2.2
By matching	By MS/MS	By matching	By matching	By MS/MS	7.9	7.9
By matching	By MS/MS	By matching	By matching	By matching	1.3	1.3
By MS/MS	By MS/MS	By matching	By matching	By matching	26.7	26.7
By MS/MS	By MS/MS	By matching	By matching	By matching	9.6	9.6
By matching	By matching	By matching	By matching	By matching	8.9	8.9
By matching	By MS/MS	By MS/MS	By matching	By MS/MS	1	1
By MS/MS	By MS/MS	By MS/MS	By MS/MS	By MS/MS	9.8	11.3
By MS/MS	By MS/MS	By matching	By matching	By matching	11	11
By MS/MS	By MS/MS	By MS/MS	By matching	By matching	56	56
By MS/MS	By MS/MS	By MS/MS	By matching	By MS/MS	3.9	1.8
By MS/MS	By MS/MS	By MS/MS	By MS/MS	By MS/MS	27.1	27.1
By MS/MS	By MS/MS	By matching	By matching	By matching	30.4	30.4
By matching	By matching	By MS/MS	By matching	By matching	1.6	4.5
By MS/MS	By MS/MS	By matching	By matching	By MS/MS	2.3	2.3
By matching	By MS/MS	By matching	By MS/MS	By MS/MS	9.9	4.9
By MS/MS	By MS/MS	By MS/MS	By MS/MS	By MS/MS	48.2	48.2
By matching	By MS/MS	By matching	By matching	By matching	30.5	30.5
By MS/MS	By MS/MS	By MS/MS	By MS/MS	By MS/MS	53.2	53.2
By MS/MS	By MS/MS	By matching	By matching	By matching	41	41
By MS/MS	By MS/MS	By MS/MS	By matching	By MS/MS	9	9
By MS/MS	By MS/MS	By MS/MS	By matching	By MS/MS	1.7	1.7
By MS/MS	By MS/MS	By matching	By matching	By matching	8.8	8.8
By MS/MS	By MS/MS	By matching	By matching	By matching	24	24
By MS/MS	By MS/MS	By MS/MS	By MS/MS	By MS/MS	48.2	48.2
By MS/MS	By MS/MS	By matching	By matching	By matching	6.1	6.1
By MS/MS	By MS/MS	By MS/MS	By MS/MS	By MS/MS	9.9	9.8
By MS/MS	By MS/MS	By MS/MS	By MS/MS	By MS/MS	28.4	18.9
By MS/MS	By matching	By matching	By matching	By matching	8.3	8.3
By MS/MS	By MS/MS	By matching	By matching	By matching	4.7	4.7
By MS/MS	By MS/MS	By matching	By matching	By matching	7.9	4.1
By MS/MS	By MS/MS	By matching	By matching	By matching	7.9	7.9
By MS/MS	By MS/MS	By MS/MS	By MS/MS	By MS/MS	48.1	48.1
By MS/MS	By MS/MS	By MS/MS	By matching	By MS/MS	12.3	10.3
By matching	By MS/MS	By matching	By matching	By matching	21.7	21.7
By MS/MS	By MS/MS	By MS/MS	By MS/MS	By MS/MS	28.1	28.1
By MS/MS	By MS/MS	By MS/MS	By matching	By MS/MS	16.8	16.8
By MS/MS	By MS/MS	By matching	By matching	By matching	6.1	6.1
By MS/MS	By MS/MS	By MS/MS	By MS/MS	By MS/MS	36.3	36.3
By MS/MS	By MS/MS	By matching	By matching	By matching	6.5	6.5
By MS/MS	By MS/MS	By matching	By matching	By MS/MS	7.4	7.4
By MS/MS	By MS/MS	By MS/MS	By matching	By matching	15.3	15.3
By MS/MS	By MS/MS	By matching	By matching	By matching	3.1	3.1
By MS/MS	By MS/MS	By matching	By matching	By MS/MS	14.6	14.6

By matching	By matching	By matching	By matching	By MS/MS	0	0
By MS/MS	By MS/MS	By matching	By MS/MS	By MS/MS	15.6	15.6
By MS/MS	By MS/MS	By matching	By matching	By MS/MS	16.7	16.7
By matching	By MS/MS	By matching	By MS/MS	By MS/MS	11.5	11.5
By MS/MS	By MS/MS	By matching	By matching	By matching	2.9	3.6
By MS/MS	By MS/MS	By MS/MS	By MS/MS	By MS/MS	3.8	3.8
By MS/MS	By MS/MS	By MS/MS	By MS/MS	By MS/MS	19.3	19.3
By matching	By matching	By matching	By matching	By matching	4.8	0
By MS/MS	By MS/MS	By MS/MS	By MS/MS	By MS/MS	9.3	9.3
By MS/MS	By MS/MS	By matching	By matching	By matching	41	41
By MS/MS	By matching	By matching	By matching	By matching	12.4	12.4
By MS/MS	By MS/MS	By MS/MS	By matching	By MS/MS	28.8	26.7
By MS/MS	By MS/MS	By MS/MS	By MS/MS	By MS/MS	48.3	48.3
By matching	By MS/MS	By MS/MS	By MS/MS	By MS/MS	21.4	15.3
By MS/MS	By MS/MS	By MS/MS	By MS/MS	By MS/MS	6.1	6.1
By MS/MS	By MS/MS	By matching	By matching	By matching	23.8	23.8
By MS/MS	By matching	By matching	By matching	By matching	4.7	4.7
By MS/MS	By MS/MS	By MS/MS	By MS/MS	By MS/MS	17.9	21.1
By MS/MS	By MS/MS	By MS/MS	By MS/MS	By MS/MS	22.3	15.9
By matching	By matching	By MS/MS	By matching	By matching	0	0
By MS/MS	By MS/MS	By matching	By matching	By matching	20.7	17.4
By MS/MS	By MS/MS	By MS/MS	By matching	By MS/MS	25.8	25.8
By MS/MS	By MS/MS	By MS/MS	By MS/MS	By MS/MS	28.5	37
By MS/MS	By MS/MS	By MS/MS	By matching	By matching	9.3	4.9
By MS/MS	By MS/MS	By matching	By matching	By matching	9.6	9.6
By MS/MS	By MS/MS	By matching	By matching	By matching	6.8	6.8
By MS/MS	By MS/MS	By MS/MS	By MS/MS	By MS/MS	4.2	4.2
By MS/MS	By MS/MS	By MS/MS	By MS/MS	By MS/MS	24.3	21.9
By MS/MS	By MS/MS	By MS/MS	By MS/MS	By MS/MS	28.1	28.1
By MS/MS	By MS/MS	By MS/MS	By MS/MS	By MS/MS	32.2	32.2
By MS/MS	By MS/MS	By matching	By matching	By matching	12.9	12.9
By MS/MS	By MS/MS	By matching	By MS/MS	By MS/MS	6.1	10.2
By matching	By MS/MS	By matching	By matching	By matching	4.5	1.8
By MS/MS	By matching	By matching	By matching	By matching	8.5	8.5
By MS/MS	By MS/MS	By matching	By matching	By matching	1.4	3.2
By MS/MS	By matching	By matching	By matching	By matching	4.6	4.6
By matching	By matching	By matching	By matching	By matching	22.4	0
By matching	By MS/MS	By MS/MS	By MS/MS	By MS/MS	10.6	10.6
By MS/MS	By MS/MS	By MS/MS	By MS/MS	By MS/MS	65.6	67.3
By matching	By matching	By matching	By MS/MS	By MS/MS	0	0
By MS/MS	By MS/MS	By matching	By matching	By matching	8	8
By MS/MS	By matching	By MS/MS	By MS/MS	By MS/MS	18.2	18.2
By matching	By matching	By matching	By matching	By matching	20.6	20.6
By MS/MS	By MS/MS	By MS/MS	By MS/MS	By MS/MS	57.5	54.6
By matching	By matching	By MS/MS	By matching	By matching	3.2	3.2

By matching	By MS/MS	By MS/MS	By matching	By MS/MS	14.2	14.2
By MS/MS	By MS/MS	By MS/MS	By MS/MS	By MS/MS	18.2	21.1
By MS/MS	By MS/MS	By MS/MS	By MS/MS	By MS/MS	50	50
By MS/MS	By MS/MS	By MS/MS	By MS/MS	By MS/MS	8.8	14
By matching	By MS/MS	By matching	By matching	By matching	5.1	5.1
By matching	By MS/MS	By MS/MS	By matching	By matching	5.9	5.9
By MS/MS	By MS/MS	By matching	By matching	By matching	5.7	2.4
By MS/MS	By MS/MS	By matching	By matching	By matching	27.7	27.7
By MS/MS	By MS/MS	By matching	By matching	By matching	19.7	19.7
By matching	By MS/MS	By MS/MS	By MS/MS	By MS/MS	11.4	11.4
By MS/MS	By MS/MS	By matching	By matching	By matching	24.7	24.7
By MS/MS	By MS/MS	By matching	By matching	By matching	2.4	2.4
By MS/MS	By MS/MS	By MS/MS	By matching	By matching	4.5	4.5
By MS/MS	By MS/MS	By matching	By matching	By matching	16.8	16.8
By MS/MS	By MS/MS	By MS/MS	By MS/MS	By MS/MS	2.4	2.4
By MS/MS	By MS/MS	By matching	By matching	By matching	12.6	12.6
By MS/MS	By MS/MS	By MS/MS	By MS/MS	By MS/MS	16	17.3
By MS/MS	By MS/MS	By MS/MS	By matching	By matching	5.9	5.9
By MS/MS	By MS/MS	By MS/MS	By MS/MS	By MS/MS	17.8	20.3
By MS/MS	By MS/MS	By MS/MS	By MS/MS	By MS/MS	27.1	30.1
By MS/MS	By MS/MS	By MS/MS	By matching	By matching	8.7	8.7
By MS/MS	By matching	By MS/MS	By matching	By MS/MS	3.4	3.4
By MS/MS	By MS/MS	By MS/MS	By MS/MS	By MS/MS	9.2	9.2
By MS/MS	By MS/MS	By MS/MS	By MS/MS	By MS/MS	43.4	36.4
By matching	By MS/MS	By matching	By matching	By matching	12.1	12.1
By MS/MS	By MS/MS	By matching	By matching	By matching	7.9	6.3
By MS/MS	By MS/MS	By matching	By matching	By matching	8.9	9.3
By MS/MS	By matching	By matching	By matching	By MS/MS	21.5	21.5
By MS/MS	By MS/MS	By MS/MS	By MS/MS	By MS/MS	29.8	32.7
By MS/MS	By MS/MS	By matching	By matching	By MS/MS	14.1	14.1
By matching	By MS/MS	By matching	By matching	By matching	1.1	1.1
By MS/MS	By MS/MS	By matching	By matching	By matching	7.9	7.9
By MS/MS	By MS/MS	By MS/MS	By MS/MS	By MS/MS	24	24
By MS/MS	By MS/MS	By matching	By matching	By matching	9.9	9.9
By MS/MS	By matching	By MS/MS	By matching	By matching	0.4	0.4
By matching	By MS/MS	By matching	By matching	By MS/MS	8.6	8.6
By matching	By MS/MS	By matching	By matching	By matching	8	0
By MS/MS	By MS/MS	By MS/MS	By matching	By MS/MS	27.4	20.1
By MS/MS	By MS/MS	By matching	By matching	By matching	7.4	7.4
By MS/MS	By MS/MS	By matching	By matching	By MS/MS	4.3	4.3
By MS/MS	By MS/MS	By MS/MS	By MS/MS	By MS/MS	6.7	13
By MS/MS	By MS/MS	By MS/MS	By matching	By MS/MS	0	12
By MS/MS	By MS/MS	By MS/MS	By MS/MS	By MS/MS	39.1	39.1
By matching	By matching	By MS/MS	By matching	By matching	0	0
By MS/MS	By MS/MS	By MS/MS	By MS/MS	By MS/MS	24.4	24.4

By MS/MS	By MS/MS	By MS/MS	By MS/MS	By MS/MS	56.4	56.4
By MS/MS	By MS/MS	By MS/MS	By MS/MS	By MS/MS	7.6	6.7
By MS/MS	By MS/MS	By MS/MS	By MS/MS	By MS/MS	15.8	15.8
By MS/MS	By MS/MS	By matching	By matching	By matching	7	7
By matching	By matching	By MS/MS	By matching	By matching	0	0
By MS/MS	By MS/MS	By matching	By matching	By matching	12.8	12.8
By MS/MS	By MS/MS	By MS/MS	By MS/MS	By MS/MS	30.5	28.6
By MS/MS	By MS/MS	By MS/MS	By MS/MS	By MS/MS	68.7	68.7
By MS/MS	By MS/MS	By MS/MS	By MS/MS	By MS/MS	24	24
By MS/MS	By MS/MS	By matching	By matching	By matching	6.8	6
By MS/MS	By MS/MS	By matching	By matching	By MS/MS	16.2	16.3
By MS/MS	By MS/MS	By matching	By matching	By matching	14.5	14.5
By MS/MS	By MS/MS	By matching	By matching	By matching	15.7	15.7
By matching	By MS/MS	By matching	By matching	By matching	17.6	17.6
By MS/MS	By MS/MS	By matching	By MS/MS	By MS/MS	13.1	12.4
By matching	By MS/MS	By matching	By matching	By matching	5.4	5.4
By MS/MS	By MS/MS	By matching	By matching	By MS/MS	22.1	23.7
By MS/MS	By MS/MS	By matching	By matching	By matching	27.2	27.2
By MS/MS	By MS/MS	By matching	By matching	By matching	2.5	2.5
By MS/MS	By MS/MS	By MS/MS	By MS/MS	By MS/MS	28.4	29.4
By MS/MS	By MS/MS	By MS/MS	By matching	By matching	21.6	18.1
By MS/MS	By MS/MS	By MS/MS	By MS/MS	By MS/MS	43.9	44.2
By MS/MS	By matching	By matching	By matching	By matching	2.5	2.5
By MS/MS	By MS/MS	By MS/MS	By MS/MS	By MS/MS	55.2	55.2
By matching	By MS/MS	By matching	By matching	By matching	5.5	5.5
By MS/MS	By MS/MS	By MS/MS	By MS/MS	By MS/MS	17.8	17.8
By matching	By matching	By MS/MS	By matching	By MS/MS	0	0
By MS/MS	By MS/MS	By MS/MS	By MS/MS	By MS/MS	35.9	35.9
By MS/MS	By MS/MS	By MS/MS	By MS/MS	By MS/MS	20.9	16
By MS/MS	By MS/MS	By matching	By MS/MS	By MS/MS	29.9	33.2
By MS/MS	By MS/MS	By MS/MS	By MS/MS	By MS/MS	45.6	45.6
By MS/MS	By MS/MS	By MS/MS	By MS/MS	By MS/MS	29.4	29.4
By MS/MS	By MS/MS	By MS/MS	By MS/MS	By MS/MS	31.3	31.3
By MS/MS	By MS/MS	By MS/MS	By MS/MS	By MS/MS	14.5	14.5
By matching	By matching	By matching	By matching	By MS/MS	0	0
By MS/MS	By MS/MS	By matching	By MS/MS	By MS/MS	21.1	21.1
By MS/MS	By MS/MS	By matching	By matching	By MS/MS	12	12
By MS/MS	By MS/MS	By MS/MS	By MS/MS	By MS/MS	67.9	66.3
By MS/MS	By MS/MS	By MS/MS	By MS/MS	By MS/MS	3.6	3.6
By MS/MS	By MS/MS	By MS/MS	By MS/MS	By MS/MS	37	37
By MS/MS	By MS/MS	By MS/MS	By MS/MS	By MS/MS	32.1	24.7
By matching	By matching	By MS/MS	By MS/MS	By MS/MS	0	0
By MS/MS	By MS/MS	By MS/MS	By MS/MS	By MS/MS	14.3	14.3
By MS/MS	By MS/MS	By matching	By matching	By MS/MS	4.8	4.8
By MS/MS	By MS/MS	By MS/MS	By MS/MS	By MS/MS	34.4	39.6

By MS/MS	By MS/MS	By MS/MS	By MS/MS	By MS/MS	28.2	28.2
By MS/MS	By matching	By matching	By MS/MS	By matching	20	20
By MS/MS	By matching	By matching	By matching	By MS/MS	13.4	13.4
By MS/MS	By MS/MS	By matching	By matching	By MS/MS	28.4	28.4
By MS/MS	By MS/MS	By matching	By matching	By matching	6.9	6.9
By MS/MS	By MS/MS	By MS/MS	By matching	By matching	18.4	18.4
By MS/MS	By MS/MS	By MS/MS	By MS/MS	By MS/MS	9.3	9.3
By MS/MS	By matching	By matching	By MS/MS	By MS/MS	6.1	6.1
By MS/MS	By MS/MS	By matching	By matching	By matching	6.8	6.8
By matching	By matching	By MS/MS	By MS/MS	By MS/MS	0	0
By MS/MS	By MS/MS	By matching	By matching	By MS/MS	17.7	17.7
By MS/MS	By MS/MS	By matching	By matching	By matching	0.7	0.7
By matching	By matching	By MS/MS	By MS/MS	By MS/MS	0	0
By MS/MS	By MS/MS	By matching	By matching	By matching	8.9	8.9
By MS/MS	By MS/MS	By matching	By matching	By matching	1.7	4.1
By MS/MS	By MS/MS	By matching	By matching	By matching	20	23.4
By MS/MS	By MS/MS	By MS/MS	By MS/MS	By MS/MS	16.6	16.6
By MS/MS	By MS/MS	By MS/MS	By MS/MS	By MS/MS	9.6	12.4
By MS/MS	By MS/MS	By matching	By matching	By matching	8.5	8.5
By MS/MS	By MS/MS	By matching	By matching	By MS/MS	46.5	44
By MS/MS	By MS/MS	By matching	By matching	By matching	5.8	5.8
By MS/MS	By MS/MS	By matching	By MS/MS	By MS/MS	21.8	23.2
By matching	By MS/MS	By MS/MS	By matching	By MS/MS	0	0
By MS/MS	By MS/MS	By matching	By matching	By matching	3.7	3.7
By MS/MS	By MS/MS	By matching	By matching	By matching	10.2	10.2
By matching	By matching	By matching	By matching	By matching	12.2	12.2
By matching	By MS/MS	By MS/MS	By MS/MS	By MS/MS	2.7	2.7
By MS/MS	By MS/MS	By matching	By matching	By matching	5	5
By MS/MS	By MS/MS	By matching	By matching	By matching	4.2	4.2
By matching	By MS/MS	By matching	By matching	By matching	1.2	1.2
By matching	By MS/MS	By matching	By matching	By matching	7.7	7.7
By MS/MS	By MS/MS	By matching	By matching	By matching	17	17
By matching	By matching	By MS/MS	By MS/MS	By MS/MS	0	0
By MS/MS	By MS/MS	By MS/MS	By MS/MS	By MS/MS	38.9	38.9
By MS/MS	By MS/MS	By MS/MS	By matching	By MS/MS	10.7	10.7
By matching	By MS/MS	By MS/MS	By matching	By MS/MS	7.7	7.7
By matching	By MS/MS	By MS/MS	By MS/MS	By matching	9.2	9.2
By matching	By MS/MS	By matching	By matching	By matching	6.8	3.6
By MS/MS	By MS/MS	By matching	By matching	By matching	7.6	5.1
By matching	By MS/MS	By matching	By matching	By matching	3.6	3.6
By MS/MS	By MS/MS	By MS/MS	By MS/MS	By MS/MS	43.3	45.2
By matching	By matching	By MS/MS	By matching	By MS/MS	0	0
By MS/MS	By MS/MS	By matching	By matching	By matching	11.7	11.7
By matching	By MS/MS	By matching	By matching	By matching	2.9	2.9
By MS/MS	By MS/MS	By MS/MS	By MS/MS	By MS/MS	8.5	8.5

By MS/MS	By MS/MS	By matching	By matching	By matching	8.6	8.6
U27;S5DN96;S5DN91;S5DLI1;S5DI47;S5DHS8;S5DHS4;S5CRT6;S5CGR3;S4VAM7;S4VA55;S0F3H7;S0F3A9;S0F2I4;S0F2B7;S0F						
R8G7;F8R8G6;F8R8G5;F8R8G4;F8R1C5;F8R1C3;F8R1B9;F8R1B8;F8R1B6;F8R1B5;F8R1B1;F8R1A9;F8R1A8;F8R1A5;F8R1A3;						
);S5NIY1;S5DU27;S5DN96;S5DN91;S5DLI1;S5DI47;S5DHS8;S5DHS4;S5CRT6;S5CGR3;S4VAM7;S4VA55;S0F3H7;S0F3A9;S0F2						
R8G7;F8R8G6;F8R8G5;F8R8G4;F8R1C5;F8R1C3;F8R1B9;F8R1B8;F8R1B6;F8R1B5;F8R1B1;F8R1A9;F8R1A8;F8R1A5;F8R1A3;						
3;273;273;273;273;273;273;273;273;273;273;273;273;273;273;273;273;273;273;273;273;273;273;273;273;273;27						
1	0	4				270
By MS/MS	By MS/MS	By MS/MS	By MS/MS	By MS/MS	27.9	27.9
By MS/MS	By MS/MS	By matching	By matching	By matching	14.9	14.9
By matching	By matching	By matching	By matching	By matching	19.5	0
By matching	By MS/MS	By matching	By matching	By matching	2	2
By MS/MS	By MS/MS	By matching	By matching	By matching	20.3	20.3
By matching	By MS/MS	By matching	By matching	By matching	5.3	5.3
By MS/MS	By MS/MS	By MS/MS	By MS/MS	By MS/MS	8.9	8.9
By MS/MS	By MS/MS	By matching	By matching	By MS/MS	29	36.1
By matching	By matching	By MS/MS	By matching	By matching	0	0
By matching	By MS/MS	By matching	By matching	By MS/MS	6.8	0
By MS/MS	By MS/MS	By MS/MS	By MS/MS	By MS/MS	24.2	27.2
By matching	By MS/MS	By matching	By matching	By matching	23.3	23.3
By MS/MS	By MS/MS	By MS/MS	By matching	By matching	11.5	11.5
By MS/MS	By MS/MS	By matching	By matching	By matching	2.4	2.4
By MS/MS	By MS/MS	By matching	By matching	By MS/MS	25.2	25.2
By matching	By MS/MS	By matching	By matching	By matching	3.9	3.9
By MS/MS	By MS/MS	By matching	By matching	By matching	24.9	24.9
By MS/MS	By MS/MS	By matching	By matching	By matching	17.6	17.6
By MS/MS	By MS/MS	By MS/MS	By MS/MS	By MS/MS	8.5	8.5
By MS/MS	By MS/MS	By MS/MS	By MS/MS	By MS/MS	56.6	56.2
By MS/MS	By MS/MS	By MS/MS	By MS/MS	By MS/MS	3.8	4.9
By MS/MS	By MS/MS	By matching	By matching	By matching	10.9	10.9
By MS/MS	By MS/MS	By MS/MS	By MS/MS	By MS/MS	11.8	11.8
By MS/MS	By MS/MS	By matching	By matching	By matching	13.7	13.7
By MS/MS	By MS/MS	By matching	By matching	By matching	4.9	4.9
By MS/MS	By MS/MS	By matching	By matching	By matching	0.3	0.3
By MS/MS	By MS/MS	By matching	By matching	By matching	2.9	2.9
By MS/MS	By MS/MS	By matching	By matching	By matching	1.9	1.9
By MS/MS	By MS/MS	By matching	By matching	By matching	9.2	9.2
By MS/MS	By MS/MS	By MS/MS	By MS/MS	By MS/MS	23.8	23.8
By MS/MS	By MS/MS	By matching	By matching	By matching	4	4
By matching	By matching	By MS/MS	By MS/MS	By MS/MS	0	0
By matching	By matching	By MS/MS	By matching	By MS/MS	2	2
By matching	By matching	By matching	By matching	By matching	3.9	3.9
By MS/MS	By MS/MS	By matching	By matching	By matching	5.1	5.1
By matching	By MS/MS	By matching	By matching	By matching	3.7	2.5
By MS/MS	By MS/MS	By MS/MS	By MS/MS	By MS/MS	3.9	3.9
By MS/MS	By MS/MS	By matching	By matching	By MS/MS	21.6	21.6

By MS/MS	By MS/MS	By MS/MS	By MS/MS	By MS/MS	30.1		30.1	
By MS/MS	By MS/MS	By matching	By matching	By matching	1.5			8
By matching	By MS/MS	By matching	By matching	By matching	6.4		6.4	
By matching	By matching	By matching	By matching	By MS/MS		0		0
By MS/MS	By MS/MS	By matching	By matching	By matching		12		12
By MS/MS	By MS/MS	By matching	By MS/MS	By MS/MS	12.2		12.2	
By matching	By matching	By MS/MS	By matching	By matching		0		0
By matching	By matching	By matching	By matching	By matching	10.6		10.6	
By MS/MS	By matching	By matching	By matching	By matching	13.5		13.5	
By MS/MS	By MS/MS	By MS/MS	By MS/MS	By MS/MS	30.2		30.2	
By MS/MS	By MS/MS	By MS/MS	By MS/MS	By MS/MS	15.7		15.7	
By MS/MS	By MS/MS	By MS/MS	By MS/MS	By MS/MS		22	24.3	
By matching	By matching	By matching	By MS/MS	By matching		0		0
By MS/MS	By MS/MS	By MS/MS	By MS/MS	By MS/MS	4.7		4.7	
By MS/MS	By MS/MS	By MS/MS	By MS/MS	By MS/MS	5.7		5.7	
By matching	By MS/MS	By matching	By matching	By matching	6.8		6.8	
By MS/MS	By matching	By matching	By matching	By matching	4.2		4.2	
By MS/MS	By matching	By matching	By matching	By MS/MS		3		3
By MS/MS	By MS/MS	By MS/MS	By MS/MS	By MS/MS	28.7		28.7	
By matching	By matching	By MS/MS	By matching	By matching	1.7		11.1	
By MS/MS	By MS/MS	By matching	By MS/MS	By MS/MS	8.1		8.1	
By MS/MS	By MS/MS	By MS/MS	By MS/MS	By MS/MS	33.9		33.9	
By MS/MS	By MS/MS	By matching	By matching	By MS/MS		2		2
By MS/MS	By MS/MS	By MS/MS	By matching	By MS/MS	5.7			8
By MS/MS	By MS/MS	By matching	By matching	By matching	6.2			7
By MS/MS	By MS/MS	By matching	By matching	By matching	10.5		3.8	
By MS/MS	By MS/MS	By matching	By matching	By matching	12.5		12.5	
By MS/MS	By MS/MS	By MS/MS	By MS/MS	By MS/MS	17.6		17.6	
By MS/MS	By MS/MS	By matching	By matching	By matching	4.9		6.4	
By MS/MS	By MS/MS	By matching	By matching	By matching	13.1		13.1	
By matching	By MS/MS	By matching	By MS/MS	By MS/MS	11.3		11.3	
By matching	By MS/MS	By matching	By matching	By matching	2.8		2.8	
By MS/MS	By MS/MS	By matching	By matching	By matching		4		4
By MS/MS	By MS/MS	By MS/MS	By matching	By MS/MS	7.6		5.2	
By matching	By MS/MS	By matching	By matching	By MS/MS	3.6		3.6	
By matching	By MS/MS	By matching	By matching	By matching		7		7
By MS/MS	By MS/MS	By MS/MS	By MS/MS	By MS/MS	41.2		41.2	
By MS/MS	By MS/MS	By matching	By matching	By matching	49.7		44.6	
By matching	By matching	By matching	By matching	By MS/MS		0		0
By matching	By matching	By MS/MS	By MS/MS	By MS/MS	2.6		2.6	
By MS/MS	By MS/MS	By matching	By matching	By matching	6.6		6.6	
By MS/MS	By MS/MS	By matching	By matching	By matching	0.8		0.8	
By MS/MS	By matching	By matching	By matching	By matching	8.5		8.5	
By MS/MS	By MS/MS	By MS/MS	By MS/MS	By MS/MS	8.7		8.7	
By MS/MS	By matching	By matching	By matching	By matching	2.7		2.7	

By MS/MS	By MS/MS	By matching	By matching	By matching	8	8
By MS/MS	By MS/MS	By MS/MS	By MS/MS	By MS/MS	3.4	6.8
By matching	By MS/MS	By matching	By matching	By matching	5	5
By MS/MS	By MS/MS	By MS/MS	By MS/MS	By MS/MS	53.3	53.3
By MS/MS	By MS/MS	By matching	By matching	By matching	13.1	13.1
By matching	By matching	By MS/MS	By MS/MS	By matching	0	0
By matching	By MS/MS	By matching	By matching	By matching	4.2	4.2
By MS/MS	By matching	By matching	By matching	By matching	2.8	2.8
By MS/MS	By MS/MS	By MS/MS	By MS/MS	By MS/MS	18.9	18.9
By MS/MS	By MS/MS	By MS/MS	By MS/MS	By MS/MS	3.6	3.6
By MS/MS	By MS/MS	By MS/MS	By MS/MS	By MS/MS	28.9	28.9
By MS/MS	By MS/MS	By MS/MS	By MS/MS	By MS/MS	14.3	14.3
By MS/MS	By MS/MS	By MS/MS	By MS/MS	By MS/MS	5.3	4.3
By MS/MS	By MS/MS	By MS/MS	By MS/MS	By MS/MS	16	17.2
By MS/MS	By MS/MS	By MS/MS	By MS/MS	By MS/MS	22.7	22.7
By MS/MS	By MS/MS	By matching	By matching	By matching	5.8	5.8
By MS/MS	By MS/MS	By MS/MS	By MS/MS	By MS/MS	12.1	8.8
By matching	By MS/MS	By matching	By matching	By matching	1.7	1.7
By MS/MS	By MS/MS	By MS/MS	By MS/MS	By MS/MS	55.8	53.9
By MS/MS	By MS/MS	By matching	By matching	By matching	19.2	16.5
By MS/MS	By MS/MS	By MS/MS	By matching	By MS/MS	27.9	27.9
By MS/MS	By MS/MS	By matching	By matching	By matching	1.2	1.2
By matching	By MS/MS	By matching	By matching	By matching	8	5.3
By MS/MS	By MS/MS	By MS/MS	By matching	By MS/MS	23.2	23.2
By MS/MS	By MS/MS	By MS/MS	By MS/MS	By MS/MS	42.6	39.2
By MS/MS	By MS/MS	By matching	By matching	By matching	5.3	5.3
By matching	By matching	By MS/MS	By matching	By matching	0	0
By MS/MS	By MS/MS	By MS/MS	By MS/MS	By MS/MS	9.8	9
By MS/MS	By MS/MS	By matching	By matching	By matching	5.1	5.1
By MS/MS	By MS/MS	By matching	By matching	By MS/MS	32.5	32.5
By MS/MS	By MS/MS	By matching	By matching	By MS/MS	5.9	5.9
By matching	By MS/MS	By matching	By matching	By MS/MS	16.9	16.9
By MS/MS	By MS/MS	By MS/MS	By MS/MS	By MS/MS	19	12.9
By matching	By MS/MS	By matching	By matching	By matching	4.7	4.7
By MS/MS	By MS/MS	By MS/MS	By MS/MS	By MS/MS	17.8	17.8
By MS/MS	By matching	By MS/MS	By matching	By MS/MS	3.9	3.9
By MS/MS	By MS/MS	By matching	By MS/MS	By MS/MS	5.8	5.8
By matching	By matching	By MS/MS	By MS/MS	By MS/MS	0	0
By MS/MS	By MS/MS	By matching	By matching	By matching	6	7.5
By matching	By matching	By MS/MS	By matching	By matching	1.9	1.9
By MS/MS	By MS/MS	By MS/MS	By MS/MS	By MS/MS	27.8	27.8
By matching	By MS/MS	By matching	By matching	By matching	18.8	18.8
By matching	By MS/MS	By matching	By matching	By matching	5.7	5.7
By MS/MS	By MS/MS	By MS/MS	By MS/MS	By MS/MS	33	28.4
By MS/MS	By MS/MS	By MS/MS	By MS/MS	By MS/MS	36.3	36.3

By MS/MS	By MS/MS	By matching	By matching	By matching	23.5	20.6
By MS/MS	By MS/MS	By matching	By matching	By matching	9.7	9.7
By MS/MS	By MS/MS	By MS/MS	By matching	By MS/MS	27.7	27.7
By matching	By matching	By matching	By matching	By MS/MS	0	0
By MS/MS	By MS/MS	By MS/MS	By MS/MS	By MS/MS	49.8	42.9
By matching	By MS/MS	By matching	By matching	By MS/MS	11.4	11.4
By MS/MS	By MS/MS	By MS/MS	By MS/MS	By MS/MS	13.5	9.6
By MS/MS	By MS/MS	By MS/MS	By MS/MS	By MS/MS	29.7	29.7
By matching	By matching	By matching	By matching	By matching	18.9	0
By MS/MS	By MS/MS	By MS/MS	By MS/MS	By MS/MS	36.6	36.6
By MS/MS	By MS/MS	By matching	By matching	By matching	2.6	2.6
By MS/MS	By MS/MS	By matching	By matching	By matching	11.6	11.6
By MS/MS	By MS/MS	By matching	By matching	By matching	6.5	6.5
By MS/MS	By MS/MS	By matching	By matching	By matching	4.4	4.4
By MS/MS	By MS/MS	By matching	By matching	By MS/MS	14.7	14.7
By MS/MS	By matching	By matching	By matching	By matching	0.8	0.8
By MS/MS	By MS/MS	By matching	By MS/MS	By MS/MS	0	28
By MS/MS	By MS/MS	By MS/MS	By MS/MS	By MS/MS	2.8	2.8
By MS/MS	By MS/MS	By matching	By matching	By matching	2.3	2.3
By matching	By matching	By matching	By MS/MS	By MS/MS	0	0
By matching	By matching	By MS/MS	By matching	By matching	0	0
By MS/MS	By MS/MS	By MS/MS	By MS/MS	By MS/MS	14.4	13.4
By MS/MS	By MS/MS	By matching	By matching	By matching	6.9	6.9
By MS/MS	By MS/MS	By matching	By matching	By matching	13.6	12.8
By MS/MS	By MS/MS	By matching	By MS/MS	By MS/MS	2.3	2.3
By MS/MS	By MS/MS	By MS/MS	By MS/MS	By MS/MS	2.2	2.2
By matching	By MS/MS	By matching	By matching	By matching	6.8	6.8
By matching	By matching	By MS/MS	By matching	By matching	0	0
By MS/MS	By MS/MS	By MS/MS	By MS/MS	By MS/MS	15.2	14.1
By matching	By MS/MS	By matching	By matching	By matching	2	2
By matching	By matching	By MS/MS	By matching	By matching	0	0
By MS/MS	By MS/MS	By matching	By matching	By matching	6.9	6.9
By matching	By MS/MS	By matching	By matching	By matching	6.9	6.9
By matching	By matching	By matching	By MS/MS	By MS/MS	0	0
By MS/MS	By MS/MS	By matching	By matching	By MS/MS	9.1	9.1
By MS/MS	By MS/MS	By MS/MS	By matching	By matching	16.5	16.5
By MS/MS	By MS/MS	By matching	By matching	By matching	12.2	12.2
By MS/MS	By MS/MS	By MS/MS	By matching	By matching	5.1	5.1
By MS/MS	By MS/MS	By MS/MS	By MS/MS	By MS/MS	9.6	9.6
By MS/MS	By MS/MS	By MS/MS	By MS/MS	By MS/MS	22.8	18.5
By matching	By MS/MS	By matching	By matching	By matching	5	5
By MS/MS	By MS/MS	By matching	By matching	By matching	6	6
By MS/MS	By MS/MS	By MS/MS	By matching	By matching	42.8	41.1
By MS/MS	By MS/MS	By matching	By matching	By matching	21.9	15.8
By MS/MS	By MS/MS	By MS/MS	By MS/MS	By MS/MS	1.8	1.8

By MS/MS	By MS/MS	By matching	By MS/MS	By MS/MS	18.6	18.6
By MS/MS	By MS/MS	By MS/MS	By MS/MS	By MS/MS	11.5	11.5
By MS/MS	By MS/MS	By matching	By matching	By matching	5.9	3.6
By MS/MS	By MS/MS	By MS/MS	By MS/MS	By MS/MS	67	69.5
By MS/MS	By MS/MS	By matching	By matching	By matching	8.2	12.3
By MS/MS	By MS/MS	By MS/MS	By MS/MS	By MS/MS	29.2	29.2
By MS/MS	By MS/MS	By matching	By matching	By matching	6	6
By MS/MS	By MS/MS	By matching	By matching	By matching	8.1	8.1
By matching	By MS/MS	By matching	By MS/MS	By MS/MS	12.7	12.7
By MS/MS	By MS/MS	By matching	By MS/MS	By MS/MS	21.8	21.8
By MS/MS	By MS/MS	By matching	By matching	By matching	2.7	2.7
By MS/MS	By MS/MS	By matching	By matching	By matching	18.2	18.2
By MS/MS	By MS/MS	By MS/MS	By MS/MS	By MS/MS	41.9	39.7
By MS/MS	By MS/MS	By matching	By matching	By matching	14.1	9.7
By MS/MS	By MS/MS	By matching	By matching	By matching	10.6	10.6
By matching	By matching	By MS/MS	By matching	By matching	0	0
By MS/MS	By MS/MS	By MS/MS	By MS/MS	By MS/MS	52.1	52.1
By MS/MS	By MS/MS	By MS/MS	By MS/MS	By MS/MS	6.1	6.1
By MS/MS	By MS/MS	By matching	By matching	By matching	8.8	8.8
By MS/MS	By MS/MS	By MS/MS	By MS/MS	By MS/MS	42.2	42.2
By MS/MS	By MS/MS	By MS/MS	By MS/MS	By MS/MS	18.3	23.7
By MS/MS	By MS/MS	By matching	By matching	By matching	11.3	11.3
By MS/MS	By MS/MS	By matching	By matching	By MS/MS	2.6	2.6
By MS/MS	By MS/MS	By matching	By MS/MS	By MS/MS	36.5	36.5
By MS/MS	By MS/MS	By matching	By matching	By matching	14.7	14.7
By MS/MS	By MS/MS	By MS/MS	By MS/MS	By MS/MS	34.1	34.1
By matching	By matching	By matching	By matching	By MS/MS	0	0
By MS/MS	By MS/MS	By MS/MS	By MS/MS	By MS/MS	23.8	23.8
By MS/MS	By MS/MS	By MS/MS	By MS/MS	By MS/MS	19	19
By MS/MS	By MS/MS	By MS/MS	By MS/MS	By MS/MS	10.6	10.6
By MS/MS	By MS/MS	By MS/MS	By MS/MS	By MS/MS	21.2	23.9
By MS/MS	By MS/MS	By MS/MS	By matching	By MS/MS	4.7	4.7
By MS/MS	By MS/MS	By MS/MS	By MS/MS	By MS/MS	27.2	24
By MS/MS	By MS/MS	By matching	By matching	By matching	1.5	1.5
By MS/MS	By MS/MS	By matching	By matching	By matching	4.9	4.9
By matching	By MS/MS	By MS/MS	By matching	By matching	10.5	6.7
By MS/MS	By MS/MS	By MS/MS	By MS/MS	By MS/MS	5	5
By MS/MS	By MS/MS	By matching	By matching	By MS/MS	15.6	12.1
By MS/MS	By MS/MS	By MS/MS	By MS/MS	By MS/MS	11.2	11.2
By MS/MS	By MS/MS	By matching	By matching	By MS/MS	13.1	13.1
By MS/MS	By MS/MS	By matching	By matching	By matching	14.2	9.8
By MS/MS	By MS/MS	By matching	By matching	By MS/MS	16.1	16.1
By MS/MS	By MS/MS	By matching	By MS/MS	By matching	10.1	10.1
By MS/MS	By MS/MS	By MS/MS	By MS/MS	By MS/MS	18.2	18.2
By MS/MS	By MS/MS	By matching	By matching	By matching	17.2	17.2

By matching	By MS/MS	By MS/MS	By MS/MS	By MS/MS	6.7	6.7
By MS/MS	By matching	By matching	By matching	By MS/MS	5.2	5.2
By MS/MS	By MS/MS	By matching	By matching	By matching	6.9	6.9
By MS/MS	By MS/MS	By MS/MS	By matching	By MS/MS	13.1	13.1
By MS/MS	By MS/MS	By MS/MS	By MS/MS	By MS/MS	9.4	9.4
By matching	By matching	By MS/MS	By matching	By matching	3.7	5.6
By MS/MS	By MS/MS	By matching	By matching	By matching	9.9	9.9
By MS/MS	By MS/MS	By MS/MS	By MS/MS	By MS/MS	21.6	21.6
By matching	By MS/MS	By matching	By matching	By matching	0	0
By MS/MS	By MS/MS	By matching	By matching	By matching	6.1	6.1
By matching	By matching	By MS/MS	By MS/MS	By MS/MS	6.4	6.4
By MS/MS	By MS/MS	By MS/MS	By MS/MS	By MS/MS	12.1	12.1
By MS/MS	By MS/MS	By MS/MS	By MS/MS	By MS/MS	13.4	13.5
By MS/MS	By MS/MS	By MS/MS	By MS/MS	By MS/MS	43.9	43.9
By matching	By matching	By matching	By matching	By MS/MS	2.1	2.1
By MS/MS	By MS/MS	By MS/MS	By MS/MS	By MS/MS	14.9	12.9
By MS/MS	By MS/MS	By matching	By matching	By MS/MS	2.4	2.4
By matching	By matching	By matching	By matching	By matching	1.3	1.3
By MS/MS	By MS/MS	By matching	By matching	By matching	9.3	9.3
By MS/MS	By MS/MS	By matching	By matching	By matching	8.1	8.1
By MS/MS	By MS/MS	By matching	By matching	By matching	8.1	8.1
By MS/MS	By MS/MS	By MS/MS	By MS/MS	By MS/MS	11.5	10.7
By matching	By matching	By matching	By matching	By matching	7.2	0
By MS/MS	By matching	By matching	By matching	By MS/MS	8.9	8.9
By matching	By MS/MS	By MS/MS	By matching	By MS/MS	20.3	20.3
By MS/MS	By MS/MS	By MS/MS	By MS/MS	By MS/MS	45.7	45.7
By MS/MS	By MS/MS	By matching	By MS/MS	By MS/MS	2.3	2.3
By MS/MS	By MS/MS	By matching	By matching	By matching	10.5	10.5
By MS/MS	By MS/MS	By matching	By matching	By MS/MS	21.1	21.1
By MS/MS	By MS/MS	By matching	By MS/MS	By MS/MS	3	3
By MS/MS	By MS/MS	By matching	By matching	By matching	17.3	17.3
By MS/MS	By MS/MS	By MS/MS	By matching	By MS/MS	14.1	14.1
By MS/MS	By matching	By matching	By matching	By matching	10.8	10.8
By MS/MS	By MS/MS	By matching	By matching	By MS/MS	23.4	23.4
By MS/MS	By MS/MS	By MS/MS	By MS/MS	By MS/MS	31.2	31.2
By matching	By MS/MS	By matching	By matching	By matching	13.6	13.6
By MS/MS	By MS/MS	By matching	By matching	By matching	6.4	6.4
By MS/MS	By MS/MS	By MS/MS	By MS/MS	By MS/MS	7.3	5.2
By MS/MS	By MS/MS	By matching	By matching	By MS/MS	10.6	10.6
By MS/MS	By matching	By MS/MS	By MS/MS	By MS/MS	6.7	6.7
By matching	By matching	By matching	By matching	By MS/MS	0	0
By MS/MS	By MS/MS	By MS/MS	By MS/MS	By MS/MS	51.4	51.4
By MS/MS	By MS/MS	By MS/MS	By MS/MS	By MS/MS	32	45.6
By matching	By matching	By MS/MS	By matching	By matching	0	0
By MS/MS	By MS/MS	By MS/MS	By MS/MS	By MS/MS	51.5	51.5

By MS/MS	By MS/MS	By MS/MS	By matching	By MS/MS	46.4	46.4
By MS/MS	By MS/MS	By MS/MS	By MS/MS	By matching	15.1	15.1
By MS/MS	By MS/MS	By MS/MS	By MS/MS	By MS/MS	40.8	40.8
By MS/MS	By MS/MS	By MS/MS	By matching	By MS/MS	5.4	5.4
By MS/MS	By MS/MS	By MS/MS	By MS/MS	By MS/MS	46.3	46.3
By MS/MS	By MS/MS	By matching	By matching	By matching	2.2	2.2
By MS/MS	By MS/MS	By MS/MS	By MS/MS	By matching	15.2	15.2
By MS/MS	By MS/MS	By matching	By matching	By matching	12.7	11.2
By MS/MS	By MS/MS	By MS/MS	By MS/MS	By MS/MS	4.2	4.2
By MS/MS	By MS/MS	By matching	By matching	By matching	5.9	5.9
By MS/MS	By MS/MS	By MS/MS	By MS/MS	By MS/MS	14.9	14.9
By MS/MS	By MS/MS	By MS/MS	By MS/MS	By MS/MS	21.4	27.7
By MS/MS	By matching	By matching	By matching	By matching	10.8	10.8
By MS/MS	By MS/MS	By MS/MS	By MS/MS	By MS/MS	8.4	8.4
By MS/MS	By MS/MS	By MS/MS	By MS/MS	By matching	11.6	11.6
By matching	By matching	By matching	By MS/MS	By MS/MS	2	2
By MS/MS	By MS/MS	By MS/MS	By MS/MS	By MS/MS	11.8	11.8
By MS/MS	By MS/MS	By MS/MS	By MS/MS	By MS/MS	27.4	27.4
By matching	By matching	By MS/MS	By matching	By matching	37.2	36.9
By matching	By MS/MS	By matching	By matching	By matching	1.7	1.7
By MS/MS	By MS/MS	By matching	By matching	By MS/MS	26.6	20.1
By MS/MS	By MS/MS	By matching	By matching	By matching	5.5	5.5
By MS/MS	By matching	By matching	By matching	By matching	0	2.6
By MS/MS	By MS/MS	By matching	By matching	By matching	4.4	4.4
By MS/MS	By MS/MS	By MS/MS	By MS/MS	By MS/MS	15.2	14.2
By matching	By MS/MS	By matching	By matching	By matching	2.5	2.5
By MS/MS	By MS/MS	By MS/MS	By matching	By MS/MS	6.5	6.5
By MS/MS	By MS/MS	By MS/MS	By MS/MS	By MS/MS	6.1	6.1
By MS/MS	By MS/MS	By MS/MS	By MS/MS	By MS/MS	31.4	31.4
By MS/MS	By MS/MS	By matching	By matching	By matching	17.9	17.9
By MS/MS	By MS/MS	By matching	By matching	By matching	10.2	10.2
By matching	By MS/MS	By matching	By matching	By MS/MS	7.6	7.6
By MS/MS	By matching	By matching	By matching	By matching	0	1.6
By MS/MS	By MS/MS	By matching	By matching	By matching	8.3	8.3
By MS/MS	By MS/MS	By MS/MS	By MS/MS	By MS/MS	8.3	6.6
By matching	By matching	By matching	By matching	By MS/MS	10.1	0
By MS/MS	By MS/MS	By MS/MS	By MS/MS	By MS/MS	45.7	45.7
By MS/MS	By MS/MS	By MS/MS	By MS/MS	By MS/MS	11	11
By MS/MS	By MS/MS	By MS/MS	By MS/MS	By MS/MS	4.7	8.7
By MS/MS	By MS/MS	By matching	By matching	By matching	7.8	7.8
By MS/MS	By MS/MS	By MS/MS	By MS/MS	By MS/MS	36.9	36.9
By MS/MS	By MS/MS	By MS/MS	By matching	By MS/MS	22.4	22.4
By MS/MS	By MS/MS	By MS/MS	By MS/MS	By MS/MS	51.9	51.9
By MS/MS	By MS/MS	By matching	By matching	By matching	6.5	6.5
By MS/MS	By MS/MS	By MS/MS	By MS/MS	By MS/MS	25.4	25.4

By MS/MS	By MS/MS	By matching	By matching	By MS/MS	9.5		9.5	
By MS/MS	By MS/MS	By matching	By matching	By matching	9.3		8.4	
By MS/MS	By MS/MS	By matching	By matching	By matching	9.9		9.1	
By MS/MS	By MS/MS	By matching	By matching	By matching		35	32.2	
By MS/MS	By MS/MS	By matching	By matching	By matching	6.3			5
By MS/MS	By MS/MS	By matching	By matching	By MS/MS	11.9		11.9	
By MS/MS	By MS/MS	By matching	By matching	By MS/MS		45	31.8	
By MS/MS	By MS/MS	By MS/MS	By matching	By matching		15		15
By MS/MS	By MS/MS	By matching	By MS/MS	By MS/MS	38.1		38.1	
By MS/MS	By MS/MS	By MS/MS	By MS/MS	By MS/MS	11.3		11.3	
By MS/MS	By MS/MS	By MS/MS	By MS/MS	By MS/MS	4.3		4.3	
By MS/MS	By MS/MS	By MS/MS	By MS/MS	By MS/MS	18.4		16.6	
By MS/MS	By MS/MS	By matching	By matching	By matching	34.3		34.3	
By matching	By MS/MS	By matching	By matching	By matching	10.6		10.6	
By MS/MS	By MS/MS	By MS/MS	By MS/MS	By MS/MS	7.9		7.9	
By matching	By matching	By matching	By matching	By MS/MS	2.2		2.2	
By MS/MS	By MS/MS	By matching	By matching	By MS/MS	10.5		10.5	
By MS/MS	By MS/MS	By MS/MS	By MS/MS	By MS/MS	36.7		36.7	
By MS/MS	By matching	By matching	By matching	By matching	5.8		5.8	
By MS/MS	By MS/MS	By matching	By matching	By matching	21.4		21.4	
By matching	By MS/MS	By matching	By matching	By matching	1.7		1.7	
By matching	By MS/MS	By matching	By matching	By matching		0		0
By MS/MS	By MS/MS	By MS/MS	By MS/MS	By MS/MS	14.7		11.4	
By MS/MS	By MS/MS	By MS/MS	By MS/MS	By MS/MS	57.2		57.2	
By MS/MS	By MS/MS	By MS/MS	By matching	By matching	16.4		16.4	
By MS/MS	By MS/MS	By MS/MS	By MS/MS	By MS/MS	14.2		14.2	
By MS/MS	By MS/MS	By matching	By matching	By matching		5		5
By matching	By MS/MS	By matching	By matching	By matching	4.9		4.9	
By matching	By MS/MS	By matching	By matching	By matching	2.7		2.7	
By MS/MS	By matching	By matching	By MS/MS	By MS/MS	2.1		2.1	
By MS/MS	By MS/MS	By MS/MS	By MS/MS	By MS/MS	22.6		20.4	
By MS/MS	By MS/MS	By matching	By matching	By matching	5.3		4.5	
By matching	By MS/MS	By matching	By matching	By matching	1.5		1.5	
By MS/MS	By MS/MS	By matching	By matching	By matching	10.7		10.7	
By MS/MS	By MS/MS	By matching	By matching	By matching	11.4		11.4	
By MS/MS	By MS/MS	By matching	By matching	By matching	12.6		10.5	
By MS/MS	By MS/MS	By MS/MS	By MS/MS	By MS/MS	8.1		8.1	
By MS/MS	By MS/MS	By matching	By matching	By matching	6.5		6.5	
By MS/MS	By MS/MS	By MS/MS	By MS/MS	By MS/MS	3.3		3.3	
By MS/MS	By MS/MS	By matching	By matching	By MS/MS	7.5		7.5	
By matching	By matching	By MS/MS	By matching	By matching		0		0
By MS/MS	By MS/MS	By MS/MS	By MS/MS	By MS/MS	7.6		7.6	
By MS/MS	By MS/MS	By matching	By matching	By matching	4.4		5.8	
By MS/MS	By MS/MS	By matching	By matching	By MS/MS	17.3		16.8	
By MS/MS	By MS/MS	By matching	By matching	By matching		7	4.8	

By MS/MS	By MS/MS	By MS/MS	By MS/MS	By MS/MS	30.7	30.7
By matching	By matching	By matching	By matching	By MS/MS	33.1	37.6
By MS/MS	By MS/MS	By MS/MS	By MS/MS	By MS/MS	44	44
By MS/MS	By MS/MS	By matching	By MS/MS	By MS/MS	2.9	2.9
By matching	By matching	By MS/MS	By MS/MS	By matching	0	0
By MS/MS	By MS/MS	By MS/MS	By matching	By MS/MS	13.1	9.8
By MS/MS	By MS/MS	By matching	By matching	By matching	20	20
By MS/MS	By MS/MS	By matching	By matching	By MS/MS	17.8	17.8
By MS/MS	By MS/MS	By matching	By matching	By matching	30.7	30.7
By matching	By matching	By matching	By matching	By MS/MS	19.7	6
By MS/MS	By MS/MS	By matching	By matching	By matching	16.8	18.1
By matching	By matching	By MS/MS	By MS/MS	By MS/MS	0	0
By MS/MS	By MS/MS	By matching	By matching	By MS/MS	26.8	26.8
By matching	By matching	By MS/MS	By matching	By matching	0	0
By MS/MS	By MS/MS	By matching	By matching	By matching	17.9	17.9
By MS/MS	By MS/MS	By MS/MS	By MS/MS	By MS/MS	8.6	8.6
By matching	By MS/MS	By matching	By matching	By matching	3.5	3.5
By MS/MS	By MS/MS	By MS/MS	By MS/MS	By MS/MS	9.3	9.3
By matching	By matching	By matching	By matching	By MS/MS	0	0
By matching	By MS/MS	By matching	By matching	By MS/MS	3.6	3.6
By matching	By MS/MS	By matching	By matching	By matching	3.8	3.8
By matching	By matching	By matching	By MS/MS	By MS/MS	3.6	3.6
By MS/MS	By MS/MS	By MS/MS	By MS/MS	By MS/MS	5.8	5.8
By MS/MS	By MS/MS	By matching	By matching	By matching	17.4	17.4
By MS/MS	By MS/MS	By MS/MS	By MS/MS	By MS/MS	10.2	10.2
By MS/MS	By MS/MS	By matching	By matching	By matching	10.3	10.3
By MS/MS	By MS/MS	By MS/MS	By MS/MS	By MS/MS	27.2	27.2
By matching	By matching	By matching	By matching	By MS/MS	0	0
By MS/MS	By MS/MS	By MS/MS	By MS/MS	By MS/MS	38.1	37.4
By MS/MS	By MS/MS	By MS/MS	By matching	By matching	10.7	10.7
By matching	By matching	By MS/MS	By matching	By MS/MS	0	0
By matching	By matching	By MS/MS	By MS/MS	By MS/MS	0	0
By MS/MS	By MS/MS	By matching	By matching	By matching	12.9	12.9
By MS/MS	By MS/MS	By matching	By matching	By matching	25.3	25.3
By MS/MS	By MS/MS	By MS/MS	By matching	By MS/MS	27.1	27.1
By MS/MS	By MS/MS	By MS/MS	By MS/MS	By MS/MS	6.5	6.5
By MS/MS	By MS/MS	By matching	By matching	By matching	14.2	14.2
By MS/MS	By matching	By MS/MS	By MS/MS	By matching	14.3	14.3
By MS/MS	By MS/MS	By matching	By matching	By matching	6.7	6.7
By matching	By MS/MS	By matching	By matching	By matching	7.4	7.4
By MS/MS	By MS/MS	By matching	By matching	By matching	13.7	13.7
By MS/MS	By MS/MS	By matching	By matching	By matching	9	9
By MS/MS	By MS/MS	By matching	By MS/MS	By MS/MS	23.4	23.4
By MS/MS	By MS/MS	By matching	By matching	By matching	4.7	4.7
By matching	By MS/MS	By matching	By matching	By matching	3.6	3.6

By MS/MS	By MS/MS	By matching	By matching	By matching	5.4	5.4
By MS/MS	By MS/MS	By MS/MS	By matching	By MS/MS	0	2.1
By MS/MS	By MS/MS	By matching	By matching	By matching	11.7	11.7
By matching	By matching	By MS/MS	By matching	By matching	0	0
By matching	By MS/MS	By matching	By matching	By matching	5.8	5.8
By MS/MS	By MS/MS	By MS/MS	By MS/MS	By MS/MS	8.3	8.3
By matching	By MS/MS	By MS/MS	By MS/MS	By MS/MS	8.9	8.9
By MS/MS	By MS/MS	By MS/MS	By MS/MS	By MS/MS	5.6	5.6
By MS/MS	By MS/MS	By MS/MS	By matching	By MS/MS	23.4	21.5
By MS/MS	By MS/MS	By matching	By matching	By matching	14.2	14.2
By matching	By MS/MS	By MS/MS	By MS/MS	By MS/MS	7.3	4.2
By MS/MS	By MS/MS	By matching	By MS/MS	By matching	13	15.8
By MS/MS	By MS/MS	By MS/MS	By matching	By MS/MS	21.1	10.2
By MS/MS	By matching	By matching	By matching	By matching	7.3	7.3
By matching	By MS/MS	By matching	By matching	By matching	5.7	0
By MS/MS	By MS/MS	By matching	By matching	By MS/MS	4.4	4.4
By MS/MS	By matching	By matching	By matching	By matching	68.4	68.4
By MS/MS	By MS/MS	By MS/MS	By MS/MS	By MS/MS	33	35.2
By MS/MS	By MS/MS	By MS/MS	By MS/MS	By MS/MS	48.2	48.2
By matching	By matching	By matching	By matching	By matching	6.9	6.9
By MS/MS	By MS/MS	By matching	By matching	By matching	7	4.9
By MS/MS	By MS/MS	By matching	By MS/MS	By MS/MS	8.2	8.2
By MS/MS	By matching	By matching	By matching	By matching	0.8	1.5
By MS/MS	By MS/MS	By matching	By matching	By matching	23.5	23.5
By MS/MS	By MS/MS	By MS/MS	By MS/MS	By MS/MS	21.2	21.2
By MS/MS	By MS/MS	By matching	By matching	By MS/MS	8.1	6.6
By matching	By matching	By MS/MS	By MS/MS	By MS/MS	0	0
By MS/MS	By MS/MS	By MS/MS	By MS/MS	By MS/MS	18.8	18.8
By MS/MS	By matching	By MS/MS	By MS/MS	By MS/MS	8.7	7.3
By MS/MS	By MS/MS	By MS/MS	By MS/MS	By MS/MS	16.4	16.4
By MS/MS	By MS/MS	By MS/MS	By MS/MS	By MS/MS	20.9	19.8
By MS/MS	By matching	By matching	By MS/MS	By MS/MS	0	5.1
By MS/MS	By MS/MS	By MS/MS	By MS/MS	By MS/MS	6.8	6.8
By MS/MS	By MS/MS	By matching	By matching	By matching	18.1	18.1
By MS/MS	By MS/MS	By matching	By matching	By matching	5	1.4
By matching	By matching	By MS/MS	By matching	By MS/MS	0	0
By MS/MS	By MS/MS	By matching	By matching	By matching	1.9	3.4
By MS/MS	By MS/MS	By MS/MS	By matching	By matching	4.9	4.9
By MS/MS	By MS/MS	By MS/MS	By MS/MS	By MS/MS	16.6	16.6
By MS/MS	By MS/MS	By MS/MS	By MS/MS	By MS/MS	46.9	46.9
By MS/MS	By MS/MS	By matching	By matching	By MS/MS	9.8	9.8
By MS/MS	By MS/MS	By matching	By matching	By matching	4.5	4.5
By matching	By MS/MS	By matching	By matching	By matching	11.9	11.9
By matching	By matching	By matching	By matching	By MS/MS	0	0
By MS/MS	By matching	By matching	By matching	By MS/MS	3.3	3.3

By MS/MS	By MS/MS	By matching	By matching	By MS/MS	16.7	16.7
By MS/MS	By MS/MS	By matching	By matching	By matching	7.9	7.9
By matching	By matching	By matching	By matching	By MS/MS	0	0
By MS/MS	By MS/MS	By MS/MS	By MS/MS	By MS/MS	18.2	20.2
By MS/MS	By MS/MS	By MS/MS	By MS/MS	By MS/MS	44.2	44.2
By matching	By matching	By matching	By matching	By matching	8.4	0
By MS/MS	By MS/MS	By matching	By matching	By matching	8.9	8.9
By matching	By MS/MS	By matching	By matching	By matching	33.2	30
By MS/MS	By MS/MS	By MS/MS	By MS/MS	By MS/MS	38.6	31
By MS/MS	By MS/MS	By matching	By matching	By matching	7.1	7.1
By MS/MS	By MS/MS	By matching	By matching	By matching	10.2	10.2
By MS/MS	By MS/MS	By MS/MS	By MS/MS	By MS/MS	9.9	9.9
By MS/MS	By MS/MS	By matching	By MS/MS	By MS/MS	8.9	8.9
By MS/MS	By MS/MS	By matching	By matching	By matching	4	4
By matching	By matching	By matching	By MS/MS	By MS/MS	0	0
By matching	By MS/MS	By matching	By matching	By matching	5.3	5.3
By MS/MS	By MS/MS	By matching	By matching	By matching	1.3	1.3
By MS/MS	By MS/MS	By matching	By matching	By matching	7	7
By MS/MS	By matching	By matching	By matching	By matching	7.4	7.4
By MS/MS	By MS/MS	By matching	By matching	By matching	10.6	10.6
By MS/MS	By MS/MS	By matching	By matching	By matching	16.4	15.6
By MS/MS	By MS/MS	By matching	By matching	By MS/MS	22.1	22.1
By MS/MS	By MS/MS	By MS/MS	By MS/MS	By MS/MS	7.3	7.3
By MS/MS	By MS/MS	By MS/MS	By matching	By matching	6.1	6.1
By MS/MS	By MS/MS	By matching	By matching	By matching	8.7	9.1
By MS/MS	By MS/MS	By MS/MS	By MS/MS	By MS/MS	4.2	4.2
By matching	By matching	By MS/MS	By matching	By matching	0	0
By MS/MS	By MS/MS	By matching	By matching	By matching	15.8	17.9
By MS/MS	By MS/MS	By matching	By matching	By matching	6.5	6.5
By MS/MS	By MS/MS	By MS/MS	By matching	By MS/MS	16.4	16.4
By matching	By matching	By matching	By MS/MS	By MS/MS	0	0
By MS/MS	By MS/MS	By MS/MS	By matching	By MS/MS	44.1	44.1
By MS/MS	By MS/MS	By matching	By matching	By matching	19.5	19.5
By MS/MS	By matching	By matching	By matching	By MS/MS	5.4	5.4
By MS/MS	By MS/MS	By matching	By matching	By matching	46	46
By MS/MS	By MS/MS	By MS/MS	By matching	By matching	8	8
By MS/MS	By MS/MS	By MS/MS	By MS/MS	By MS/MS	30	30
By MS/MS	By MS/MS	By MS/MS	By MS/MS	By MS/MS	9.7	9.7
By MS/MS	By MS/MS	By MS/MS	By MS/MS	By MS/MS	18.8	18.8
By MS/MS	By MS/MS	By matching	By matching	By matching	32.7	32.7
By MS/MS	By MS/MS	By MS/MS	By MS/MS	By MS/MS	41.3	41.3
By MS/MS	By MS/MS	By MS/MS	By MS/MS	By MS/MS	12.4	12.4
By matching	By matching	By MS/MS	By matching	By matching	0	0
By MS/MS	By MS/MS	By MS/MS	By matching	By MS/MS	36.6	36.6
By MS/MS	By MS/MS	By matching	By matching	By MS/MS	7.2	7.2

By matching	By matching	By MS/MS	By matching	By matching	1.7	1.7
By matching	By matching	By MS/MS	By matching	By matching	1.5	1.5
By MS/MS	By MS/MS	By MS/MS	By MS/MS	By MS/MS	1.4	1.4
By MS/MS	By MS/MS	By MS/MS	By matching	By matching	16	14.4
By matching	By matching	By MS/MS	By matching	By matching	1.5	1.5
By matching	By matching	By MS/MS	By matching	By matching	15.8	14.5
By MS/MS	By MS/MS	By MS/MS	By MS/MS	By MS/MS	49.8	46.3
By MS/MS	By MS/MS	By MS/MS	By MS/MS	By MS/MS	58.5	48.7
By matching	By matching	By matching	By MS/MS	By matching	13.5	13.5
By MS/MS	By MS/MS	By MS/MS	By MS/MS	By MS/MS	33.7	33.7
By matching	By matching	By matching	By MS/MS	By MS/MS	5.3	5.3
By matching	By MS/MS	By MS/MS	By MS/MS	By MS/MS	20.1	20.1
By MS/MS	By MS/MS	By MS/MS	By MS/MS	By MS/MS	17.2	17.2
By MS/MS	By MS/MS	By MS/MS	By MS/MS	By MS/MS	21	21
By MS/MS	By MS/MS	By MS/MS	By MS/MS	By MS/MS	81.9	81.1
By MS/MS	By matching	By matching	By matching	By matching	10.4	10.4
By MS/MS	By MS/MS	By MS/MS	By MS/MS	By MS/MS	35.8	34.2
By MS/MS	By MS/MS	By matching	By MS/MS	By MS/MS	32.5	28.6
By MS/MS	By MS/MS	By MS/MS	By MS/MS	By MS/MS	50.5	51.6
By MS/MS	By MS/MS	By MS/MS	By matching	By MS/MS	32.3	26
By MS/MS	By MS/MS	By MS/MS	By MS/MS	By MS/MS	62.7	58.7
By MS/MS	By MS/MS	By MS/MS	By MS/MS	By MS/MS	42.7	40.5
By matching	By matching	By MS/MS	By matching	By MS/MS	0	0
By matching	By matching	By MS/MS	By MS/MS	By MS/MS	5.6	5.6
By MS/MS	By MS/MS	By MS/MS	By MS/MS	By MS/MS	67.6	67.6
By matching	By matching	By matching	By matching	By matching	77.8	72.2
By matching	By matching	By MS/MS	By matching	By matching	3.6	2.2
By MS/MS	By MS/MS	By MS/MS	By MS/MS	By MS/MS	35	27.5
By MS/MS	By matching	By MS/MS	By matching	By matching	1.7	10.8
By MS/MS	By MS/MS	By MS/MS	By MS/MS	By MS/MS	10.1	12.4
By MS/MS	By MS/MS	By matching	By matching	By MS/MS	6.4	6.4
By MS/MS	By MS/MS	By MS/MS	By matching	By MS/MS	0.8	0.8
By MS/MS	By MS/MS	By MS/MS	By matching	By MS/MS	7.4	5.6
By MS/MS	By MS/MS	By MS/MS	By matching	By matching	28.8	27.3
By matching	By MS/MS	By matching	By matching	By matching	9.5	9.5
By matching	By matching	By MS/MS	By matching	By matching	1.5	4.2
By MS/MS	By MS/MS	By MS/MS	By MS/MS	By MS/MS	4.1	4.1
By MS/MS	By matching	By matching	By matching	By matching	14.2	14.8
By MS/MS	By MS/MS	By MS/MS	By MS/MS	By MS/MS	31.8	29.6
By matching	By matching	By MS/MS	By matching	By matching	3.8	2.3
By matching	By matching	By MS/MS	By matching	By matching	0	0
By MS/MS	By MS/MS	By MS/MS	By MS/MS	By matching	4.6	4.6
By MS/MS	By matching	By MS/MS	By matching	By matching	3.7	5.5
By matching	By MS/MS	By MS/MS	By MS/MS	By matching	14.2	11
By matching	By matching	By matching	By matching	By MS/MS	4	4

By matching	By MS/MS	By MS/MS	By matching	By matching	10.6	7.4
By MS/MS	By MS/MS	By MS/MS	By MS/MS	By MS/MS	12.8	13.6
By matching	By matching	By MS/MS	By matching	By matching	0	0
By matching	By MS/MS	By MS/MS	By matching	By matching	0	0
By MS/MS	By MS/MS	By matching	By matching	By matching	41.4	34.9
By matching	By matching	By MS/MS	By matching	By matching	0	0
By matching	By matching	By MS/MS	By matching	By matching	1.5	1.5
By MS/MS	By MS/MS	By MS/MS	By MS/MS	By MS/MS	19.9	21
By MS/MS	By MS/MS	By matching	By matching	By matching	16.2	16.2
By MS/MS	By MS/MS	By matching	By matching	By matching	4.4	4.4
By MS/MS	By MS/MS	By matching	By matching	By matching	1.2	1.2
By MS/MS	By MS/MS	By matching	By matching	By MS/MS	3.2	3.2
By MS/MS	By MS/MS	By matching	By matching	By MS/MS	6.3	6.3
By MS/MS	By MS/MS	By MS/MS	By MS/MS	By MS/MS	12.8	12.8
By MS/MS	By MS/MS	By MS/MS	By MS/MS	By MS/MS	10.5	10.5
By MS/MS	By MS/MS	By matching	By matching	By matching	27.6	27.6
By matching	By MS/MS	By MS/MS	By matching	By MS/MS	2.9	2.9
By matching	By matching	By MS/MS	By matching	By MS/MS	0	0
By MS/MS	By MS/MS	By MS/MS	By MS/MS	By MS/MS	9.7	9.7
By matching	By MS/MS	By MS/MS	By MS/MS	By MS/MS	4.1	0
By MS/MS	By MS/MS	By matching	By matching	By matching	9	11.6
By MS/MS	By MS/MS	By matching	By MS/MS	By MS/MS	7.2	5.9
By MS/MS	By MS/MS	By MS/MS	By matching	By MS/MS	4.4	4.4
By MS/MS	By MS/MS	By matching	By matching	By matching	2.5	2.5
By MS/MS	By MS/MS	By MS/MS	By MS/MS	By MS/MS	18.8	18.7
By MS/MS	By MS/MS	By MS/MS	By MS/MS	By MS/MS	8.7	8.7
By MS/MS	By MS/MS	By matching	By matching	By matching	13.5	13.5
By MS/MS	By MS/MS	By MS/MS	By MS/MS	By MS/MS	26.4	16
By matching	By matching	By MS/MS	By matching	By matching	0	0
By MS/MS	By matching	By MS/MS	By MS/MS	By matching	6.7	6.7
By MS/MS	By MS/MS	By matching	By matching	By matching	5.6	4.1
By MS/MS	By MS/MS	By matching	By matching	By matching	9.8	11.1
By MS/MS	By MS/MS	By MS/MS	By MS/MS	By MS/MS	9.1	7.8
By MS/MS	By MS/MS	By MS/MS	By MS/MS	By MS/MS	10.1	9.5
By MS/MS	By MS/MS	By MS/MS	By MS/MS	By MS/MS	27.5	27.5
By MS/MS	By MS/MS	By MS/MS	By MS/MS	By MS/MS	4.7	4.7
By matching	By MS/MS	By matching	By matching	By MS/MS	2.7	2.7
By MS/MS	By MS/MS	By matching	By MS/MS	By MS/MS	9.3	9.3
By MS/MS	By MS/MS	By MS/MS	By matching	By MS/MS	11.5	11.5
By matching	By MS/MS	By matching	By matching	By matching	4.7	4.7
By MS/MS	By MS/MS	By matching	By matching	By MS/MS	17.8	17.8
By MS/MS	By MS/MS	By matching	By matching	By MS/MS	28	28
By MS/MS	By MS/MS	By MS/MS	By MS/MS	By MS/MS	43.4	43.4
By matching	By MS/MS	By matching	By matching	By matching	27.5	27.5
By MS/MS	By MS/MS	By MS/MS	By matching	By MS/MS	16.9	16.9

By MS/MS	By MS/MS	By matching	By matching	By matching	22	22
By MS/MS	By MS/MS	By matching	By matching	By matching	18	18
By matching	By matching	By MS/MS	By MS/MS	By matching	4.5	4.5
By MS/MS	By MS/MS	By MS/MS	By MS/MS	By MS/MS	18.3	18.3
By MS/MS	By MS/MS	By matching	By MS/MS	By MS/MS	3	3
By matching	By matching	By MS/MS	By MS/MS	By MS/MS	0	0
By MS/MS	By MS/MS	By matching	By matching	By matching	16.2	16.2
By MS/MS	By MS/MS	By MS/MS	By matching	By MS/MS	34.2	34.2
By MS/MS	By MS/MS	By matching	By matching	By matching	5.6	5.6
By MS/MS	By MS/MS	By MS/MS	By MS/MS	By MS/MS	3.7	3.7
By matching	By matching	By matching	By matching	By matching	16.1	16.1
By MS/MS	By MS/MS	By matching	By matching	By matching	53	49.9
By MS/MS	By MS/MS	By MS/MS	By MS/MS	By MS/MS	8.9	8.9
By matching	By MS/MS	By matching	By matching	By matching	1.9	0.6
By MS/MS	By matching	By matching	By matching	By MS/MS	1.2	1.2
By matching	By matching	By matching	By matching	By MS/MS	0	0
By MS/MS	By MS/MS	By MS/MS	By MS/MS	By MS/MS	27.7	29.4
By MS/MS	By MS/MS	By MS/MS	By MS/MS	By MS/MS	33.6	33.6
By MS/MS	By MS/MS	By matching	By matching	By matching	8.9	8.9
By MS/MS	By MS/MS	By matching	By matching	By MS/MS	33.8	31.2
By MS/MS	By MS/MS	By MS/MS	By MS/MS	By MS/MS	12.5	9
By matching	By MS/MS	By matching	By matching	By matching	18.4	18.4
By MS/MS	By MS/MS	By MS/MS	By MS/MS	By MS/MS	7.2	7.2
By MS/MS	By MS/MS	By MS/MS	By matching	By matching	13.2	13.2
By MS/MS	By matching	By matching	By matching	By matching	5.9	5.9
By MS/MS	By MS/MS	By matching	By matching	By matching	14.7	14.7
By MS/MS	By MS/MS	By MS/MS	By MS/MS	By MS/MS	24.9	28.6
By MS/MS	By MS/MS	By matching	By matching	By matching	42.7	42.7
By matching	By matching	By MS/MS	By matching	By MS/MS	19.3	22.3
By MS/MS	By MS/MS	By matching	By matching	By matching	21.1	21.1
By MS/MS	By MS/MS	By MS/MS	By MS/MS	By MS/MS	52.1	53
By matching	By matching	By MS/MS	By matching	By matching	3.1	3.1
By MS/MS	By MS/MS	By matching	By matching	By matching	5.3	7.9
By MS/MS	By MS/MS	By matching	By matching	By MS/MS	24.1	24.1
By MS/MS	By MS/MS	By matching	By matching	By matching	11.9	11.9
By MS/MS	By MS/MS	By MS/MS	By MS/MS	By MS/MS	15.8	15.8
By matching	By MS/MS	By matching	By matching	By matching	5.2	0
By MS/MS	By MS/MS	By MS/MS	By matching	By MS/MS	4.7	4.7
By MS/MS	By MS/MS	By MS/MS	By MS/MS	By MS/MS	48.2	52.3
By MS/MS	By matching	By matching	By matching	By matching	38.3	42.3
By MS/MS	By matching	By matching	By matching	By matching	1.1	1.1
By MS/MS	By MS/MS	By MS/MS	By MS/MS	By MS/MS	45.3	45.3
By MS/MS	By MS/MS	By matching	By MS/MS	By MS/MS	16.2	28.8
By MS/MS	By MS/MS	By MS/MS	By MS/MS	By MS/MS	10.8	10.8
By MS/MS	By MS/MS	By MS/MS	By MS/MS	By MS/MS	45.4	46.5

By MS/MS	By matching	By MS/MS	By matching	By matching	0.2	0.2
By matching	By MS/MS	By matching	By matching	By matching	9.8	9.8
By matching	By matching	By MS/MS	By matching	By matching	8.6	8.6
By matching	By MS/MS	By matching	By matching	By matching	3.8	5.3
By MS/MS	By MS/MS	By matching	By matching	By matching	16.4	16.4
By MS/MS	By MS/MS	By MS/MS	By MS/MS	By MS/MS	53.4	53.4
By MS/MS	By MS/MS	By matching	By matching	By matching	2.7	2.7
By MS/MS	By MS/MS	By matching	By MS/MS	By MS/MS	13.9	15.1
By MS/MS	By MS/MS	By MS/MS	By MS/MS	By MS/MS	13.5	13.5
By MS/MS	By MS/MS	By MS/MS	By MS/MS	By MS/MS	45.6	45.6
By MS/MS	By MS/MS	By matching	By matching	By matching	20.4	20.9
By MS/MS	By MS/MS	By MS/MS	By MS/MS	By MS/MS	38.6	41.2
By matching	By matching	By MS/MS	By matching	By MS/MS	0	0
By MS/MS	By MS/MS	By matching	By matching	By MS/MS	6.9	6.9
By MS/MS	By MS/MS	By MS/MS	By MS/MS	By MS/MS	23.7	23.7
By MS/MS	By MS/MS	By matching	By matching	By matching	16.8	16.8
By MS/MS	By MS/MS	By MS/MS	By MS/MS	By MS/MS	30.5	30.5
By MS/MS	By MS/MS	By matching	By matching	By matching	6	6
By MS/MS	By MS/MS	By matching	By matching	By matching	8.4	8.4
By MS/MS	By MS/MS	By matching	By matching	By MS/MS	12.4	12.4
By MS/MS	By MS/MS	By matching	By matching	By MS/MS	4.1	4.1
By matching	By matching	By MS/MS	By MS/MS	By MS/MS	0	0
By matching	By matching	By matching	By MS/MS	By MS/MS	0	0
By MS/MS	By MS/MS	By MS/MS	By MS/MS	By MS/MS	51	55
By MS/MS	By MS/MS	By MS/MS	By MS/MS	By MS/MS	11.3	11.3
By matching	By matching	By MS/MS	By MS/MS	By MS/MS	0	0
By MS/MS	By MS/MS	By matching	By matching	By MS/MS	18.8	18.8
By MS/MS	By MS/MS	By MS/MS	By MS/MS	By MS/MS	40.5	40.5
By MS/MS	By MS/MS	By matching	By matching	By matching	3.3	3.3
By MS/MS	By MS/MS	By matching	By matching	By MS/MS	5.8	5.8
By matching	By matching	By MS/MS	By matching	By matching	0	0
By MS/MS	By MS/MS	By MS/MS	By MS/MS	By MS/MS	26.8	23.4
By MS/MS	By MS/MS	By MS/MS	By MS/MS	By MS/MS	43.3	43.5
By MS/MS	By MS/MS	By MS/MS	By matching	By matching	19.7	19.7
By MS/MS	By MS/MS	By MS/MS	By MS/MS	By MS/MS	47.2	47.2
By MS/MS	By MS/MS	By MS/MS	By matching	By MS/MS	4.1	4.1
By MS/MS	By MS/MS	By matching	By matching	By matching	3.9	3.9
By MS/MS	By MS/MS	By MS/MS	By matching	By matching	3.4	3.4
By MS/MS	By MS/MS	By MS/MS	By MS/MS	By MS/MS	12.1	12.1
By MS/MS	By MS/MS	By MS/MS	By MS/MS	By MS/MS	24.8	24.8
By MS/MS	By MS/MS	By matching	By matching	By matching	11	13.1
By MS/MS	By MS/MS	By matching	By matching	By matching	13.2	13.2
By MS/MS	By MS/MS	By matching	By matching	By matching	45.2	45.2
By MS/MS	By MS/MS	By MS/MS	By MS/MS	By MS/MS	19	19
By MS/MS	By MS/MS	By MS/MS	By MS/MS	By MS/MS	26.6	26.6

By MS/MS	By MS/MS	By matching	By matching	By matching	3.2	3.2
By matching	By matching	By matching	By matching	By MS/MS	0	0
By MS/MS	By MS/MS	By matching	By MS/MS	By MS/MS	13.2	13.2
By MS/MS	By MS/MS	By matching	By matching	By matching	18.2	18.2
By MS/MS	By MS/MS	By matching	By matching	By matching	8.6	8.6
By MS/MS	By MS/MS	By matching	By matching	By matching	3.9	3.9
By MS/MS	By MS/MS	By MS/MS	By MS/MS	By MS/MS	20	20
By MS/MS	By MS/MS	By MS/MS	By MS/MS	By MS/MS	43.8	41.8
By MS/MS	By MS/MS	By matching	By matching	By matching	9	9
By MS/MS	By MS/MS	By MS/MS	By matching	By MS/MS	6.1	6.1
By MS/MS	By matching	By matching	By MS/MS	By MS/MS	7.9	7.9
By MS/MS	By matching	By matching	By matching	By matching	0.7	0.7
By MS/MS	By MS/MS	By matching	By matching	By MS/MS	37.7	37.7
By MS/MS	By MS/MS	By matching	By matching	By matching	24.4	24.4
By MS/MS	By MS/MS	By matching	By matching	By MS/MS	46.2	46.2
By matching	By MS/MS	By matching	By matching	By matching	7.2	7.2
By MS/MS	By MS/MS	By MS/MS	By MS/MS	By MS/MS	7.5	7.5
By MS/MS	By matching	By matching	By matching	By matching	0.9	0.9
By MS/MS	By MS/MS	By MS/MS	By MS/MS	By MS/MS	15.1	14.3
By MS/MS	By MS/MS	By MS/MS	By MS/MS	By MS/MS	49.1	47.1
By MS/MS	By MS/MS	By matching	By matching	By MS/MS	9.1	9.1
By MS/MS	By MS/MS	By matching	By matching	By matching	12.9	12.9
By MS/MS	By MS/MS	By matching	By matching	By MS/MS	7.1	7.1
By MS/MS	By MS/MS	By MS/MS	By MS/MS	By MS/MS	15.8	16.5
By MS/MS	By MS/MS	By matching	By matching	By matching	2.8	2.8
By MS/MS	By MS/MS	By MS/MS	By MS/MS	By MS/MS	21.5	21.5
By MS/MS	By MS/MS	By matching	By matching	By matching	5.1	5.1
By MS/MS	By MS/MS	By MS/MS	By matching	By MS/MS	14.5	13
By MS/MS	By MS/MS	By MS/MS	By MS/MS	By MS/MS	26.7	26.7
By matching	By matching	By MS/MS	By matching	By MS/MS	0	0
By MS/MS	By MS/MS	By MS/MS	By matching	By matching	7.6	10.5
By MS/MS	By MS/MS	By MS/MS	By MS/MS	By MS/MS	39	42.1
By MS/MS	By MS/MS	By matching	By matching	By MS/MS	35.7	35.7
By MS/MS	By MS/MS	By MS/MS	By MS/MS	By MS/MS	1.9	1.9
By matching	By MS/MS	By matching	By matching	By matching	4.2	0
By MS/MS	By MS/MS	By MS/MS	By MS/MS	By MS/MS	31.3	31.3
By MS/MS	By MS/MS	By MS/MS	By MS/MS	By MS/MS	30.2	30.2
By MS/MS	By MS/MS	By matching	By matching	By matching	5.1	2.7
By MS/MS	By MS/MS	By matching	By matching	By matching	11.1	11.1
By matching	By MS/MS	By matching	By MS/MS	By MS/MS	10	12.4
By MS/MS	By MS/MS	By MS/MS	By MS/MS	By MS/MS	10.5	5.8
By MS/MS	By MS/MS	By MS/MS	By matching	By MS/MS	16.9	16.9
By MS/MS	By MS/MS	By MS/MS	By MS/MS	By MS/MS	10.1	8.3
By MS/MS	By MS/MS	By matching	By matching	By MS/MS	17.7	17.7
By MS/MS	By MS/MS	By MS/MS	By MS/MS	By MS/MS	9.6	9.6

By MS/MS	By MS/MS	By MS/MS	By MS/MS	By MS/MS	13.4	13.4
By MS/MS	By MS/MS	By MS/MS	By MS/MS	By MS/MS	11.6	10.8
By matching	By MS/MS	By MS/MS	By MS/MS	By MS/MS	29.2	20.2
By MS/MS	By MS/MS	By MS/MS	By MS/MS	By MS/MS	7.4	7.4
By MS/MS	By MS/MS	By matching	By matching	By MS/MS	7.5	7.5
By MS/MS	By MS/MS	By MS/MS	By MS/MS	By MS/MS	29.4	29.4
By matching	By matching	By matching	By matching	By MS/MS	0	0
By MS/MS	By MS/MS	By matching	By matching	By matching	0.9	0.9
By MS/MS	By MS/MS	By matching	By matching	By matching	1.4	2.5
By MS/MS	By MS/MS	By matching	By matching	By MS/MS	14.7	14.7
By MS/MS	By MS/MS	By matching	By matching	By matching	7.1	6.3
By matching	By matching	By MS/MS	By matching	By matching	0	0
By MS/MS	By MS/MS	By MS/MS	By MS/MS	By MS/MS	24.3	21.8
By MS/MS	By matching	By matching	By MS/MS	By matching	2.4	2.4
By MS/MS	By MS/MS	By MS/MS	By MS/MS	By MS/MS	16	16
By MS/MS	By MS/MS	By MS/MS	By MS/MS	By MS/MS	17.6	17.6
By matching	By matching	By MS/MS	By matching	By matching	0	0
By MS/MS	By MS/MS	By matching	By matching	By matching	2.4	2.4
By matching	By matching	By matching	By MS/MS	By MS/MS	0	0
By MS/MS	By MS/MS	By matching	By matching	By matching	11.9	11.9
By MS/MS	By MS/MS	By matching	By matching	By matching	2.6	2.6
By MS/MS	By MS/MS	By matching	By matching	By matching	7.3	7.3
By MS/MS	By MS/MS	By MS/MS	By MS/MS	By MS/MS	27.3	27.3
By MS/MS	By MS/MS	By MS/MS	By MS/MS	By MS/MS	7.4	8.5
By matching	By MS/MS	By matching	By matching	By matching	5.5	10.1
By MS/MS	By MS/MS	By MS/MS	By MS/MS	By MS/MS	68.8	68.8
By MS/MS	By MS/MS	By MS/MS	By MS/MS	By MS/MS	26.5	26.5
By matching	By MS/MS	By matching	By matching	By matching	54.7	51.6
By MS/MS	By MS/MS	By MS/MS	By MS/MS	By MS/MS	23.3	23.3
By MS/MS	By MS/MS	By MS/MS	By MS/MS	By MS/MS	37.1	28.8
By MS/MS	By MS/MS	By matching	By MS/MS	By matching	24.9	24.9
By MS/MS	By MS/MS	By matching	By matching	By matching	10.9	10.9
By matching	By matching	By MS/MS	By matching	By matching	0	0
By MS/MS	By MS/MS	By MS/MS	By MS/MS	By MS/MS	40.6	40.9
By MS/MS	By MS/MS	By MS/MS	By matching	By MS/MS	19.8	16.6
By MS/MS	By MS/MS	By MS/MS	By MS/MS	By MS/MS	37.1	37.1
By MS/MS	By MS/MS	By MS/MS	By MS/MS	By MS/MS	64.6	61.3
By MS/MS	By MS/MS	By matching	By matching	By matching	6.8	5.7
By matching	By MS/MS	By matching	By matching	By matching	21.2	21.2
By MS/MS	By MS/MS	By MS/MS	By MS/MS	By MS/MS	7.4	7.4
By MS/MS	By MS/MS	By MS/MS	By matching	By MS/MS	55.4	55.4
By MS/MS	By MS/MS	By MS/MS	By MS/MS	By MS/MS	60	65.9
By MS/MS	By MS/MS	By MS/MS	By MS/MS	By MS/MS	32.5	37.3
By MS/MS	By MS/MS	By matching	By matching	By MS/MS	31.2	31.2
By matching	By matching	By MS/MS	By MS/MS	By MS/MS	0	0

By matching	By matching	By matching	By matching	By matching	5.5		5.5	
By MS/MS	By MS/MS	By MS/MS	By MS/MS	By MS/MS	58.7		58.7	
By MS/MS	By MS/MS	By MS/MS	By MS/MS	By MS/MS	55.7		55.7	
By MS/MS	By MS/MS	By MS/MS	By MS/MS	By MS/MS	27.4		26.6	
By MS/MS	By MS/MS	By matching	By matching	By matching	13.4		13.4	
By MS/MS	By MS/MS	By MS/MS	By matching	By MS/MS	38.6		38.6	
By MS/MS	By matching	By MS/MS	By matching	By MS/MS	8.2		8.2	
By MS/MS	By MS/MS	By MS/MS	By MS/MS	By MS/MS	10.6		10.6	
By MS/MS	By MS/MS	By MS/MS	By MS/MS	By MS/MS	15.5		13.3	
By MS/MS	By MS/MS	By MS/MS	By MS/MS	By MS/MS	63.5		63.5	
By MS/MS	By MS/MS	By MS/MS	By MS/MS	By MS/MS	18.7		19.1	
By MS/MS	By MS/MS	By MS/MS	By MS/MS	By MS/MS	26.1		27.9	
By MS/MS	By MS/MS	By MS/MS	By MS/MS	By MS/MS	30.5		30.5	
By MS/MS	By MS/MS	By matching	By matching	By matching	9.7		9.7	
By MS/MS	By MS/MS	By matching	By MS/MS	By MS/MS	11.5		11.5	
By matching	By MS/MS	By MS/MS	By MS/MS	By matching	10.1		10.1	
By matching	By MS/MS	By matching	By matching	By matching	20.9		20.9	
By MS/MS	By MS/MS	By MS/MS	By MS/MS	By MS/MS	27.4		27.4	
By MS/MS	By MS/MS	By matching	By matching	By matching		21		21
By MS/MS	By MS/MS	By MS/MS	By MS/MS	By MS/MS	21.8		21.8	
By MS/MS	By MS/MS	By MS/MS	By matching	By MS/MS	19.1		17.9	
By MS/MS	By MS/MS	By matching	By matching	By matching		12		12
By MS/MS	By MS/MS	By MS/MS	By MS/MS	By MS/MS	42.3		39.9	
By MS/MS	By MS/MS	By MS/MS	By MS/MS	By MS/MS	11.9		11.9	
By MS/MS	By matching	By matching	By matching	By MS/MS	3.2		3.2	
By MS/MS	By MS/MS	By MS/MS	By MS/MS	By MS/MS	51.5		51.5	
By MS/MS	By MS/MS	By matching	By matching	By matching	5.9		6.9	
By MS/MS	By MS/MS	By MS/MS	By MS/MS	By MS/MS	8.7		8.7	
By MS/MS	By MS/MS	By matching	By MS/MS	By MS/MS	11.8		11.8	
By matching	By matching	By MS/MS	By matching	By matching		0		0
By MS/MS	By MS/MS	By MS/MS	By MS/MS	By MS/MS		42	45.7	
By MS/MS	By MS/MS	By MS/MS	By MS/MS	By MS/MS		56		56
By MS/MS	By MS/MS	By MS/MS	By MS/MS	By MS/MS		47		47
By MS/MS	By MS/MS	By MS/MS	By MS/MS	By MS/MS	15.9		15.9	
By MS/MS	By MS/MS	By MS/MS	By MS/MS	By MS/MS	23.2		23.2	
By matching	By matching	By MS/MS	By matching	By MS/MS		0		0
By MS/MS	By MS/MS	By MS/MS	By MS/MS	By MS/MS	36.7		31.6	
By MS/MS	By MS/MS	By MS/MS	By MS/MS	By MS/MS	50.9		50.9	
By MS/MS	By MS/MS	By matching	By matching	By matching	12.3		12.3	
By MS/MS	By MS/MS	By MS/MS	By MS/MS	By MS/MS	12.6		12.6	
By matching	By matching	By MS/MS	By matching	By matching		0		0
By MS/MS	By MS/MS	By MS/MS	By MS/MS	By MS/MS	43.3		43.3	
By MS/MS	By MS/MS	By MS/MS	By MS/MS	By MS/MS	54.5		45.5	
By matching	By matching	By matching	By MS/MS	By matching		0		0
By MS/MS	By matching	By matching	By matching	By matching	17.9		17.9	

By MS/MS	By MS/MS	By matching	By matching	By matching	53.1	51.4
By MS/MS	By MS/MS	By MS/MS	By matching	By MS/MS	3.2	3.2
By matching	By matching	By MS/MS	By MS/MS	By MS/MS	0	0
By MS/MS	By MS/MS	By matching	By matching	By matching	14.6	14.6
By MS/MS	By MS/MS	By matching	By matching	By matching	42.6	42.6
By MS/MS	By MS/MS	By MS/MS	By MS/MS	By MS/MS	23.2	23.2
By MS/MS	By MS/MS	By MS/MS	By MS/MS	By MS/MS	15	15
By MS/MS	By MS/MS	By MS/MS	By MS/MS	By MS/MS	23.5	23.5
By MS/MS	By MS/MS	By MS/MS	By MS/MS	By MS/MS	77.6	77.6
By MS/MS	By MS/MS	By matching	By matching	By matching	21	17.5
By MS/MS	By MS/MS	By MS/MS	By MS/MS	By MS/MS	44	44
By MS/MS	By MS/MS	By MS/MS	By MS/MS	By MS/MS	48.5	48.5
By MS/MS	By MS/MS	By MS/MS	By MS/MS	By MS/MS	26.3	29.9
By MS/MS	By MS/MS	By MS/MS	By MS/MS	By MS/MS	17	17
By MS/MS	By MS/MS	By MS/MS	By MS/MS	By MS/MS	33.7	34.7
By MS/MS	By MS/MS	By MS/MS	By MS/MS	By MS/MS	3.1	3.1
By MS/MS	By MS/MS	By MS/MS	By MS/MS	By MS/MS	29.1	29.1
By MS/MS	By MS/MS	By matching	By matching	By matching	6.4	6.4
By MS/MS	By MS/MS	By matching	By matching	By matching	9.9	9.9
By MS/MS	By MS/MS	By MS/MS	By MS/MS	By MS/MS	51.4	47
By MS/MS	By MS/MS	By matching	By matching	By MS/MS	9.4	10.7
By matching	By MS/MS	By matching	By matching	By MS/MS	25	9.5
By MS/MS	By MS/MS	By matching	By matching	By matching	14.4	13.6
By MS/MS	By MS/MS	By MS/MS	By MS/MS	By MS/MS	22.9	20.1
By MS/MS	By MS/MS	By MS/MS	By MS/MS	By MS/MS	33	37.3
By MS/MS	By MS/MS	By MS/MS	By MS/MS	By MS/MS	20.6	20.6
By MS/MS	By MS/MS	By MS/MS	By MS/MS	By MS/MS	19.4	19.4
By MS/MS	By matching	By matching	By matching	By matching	12.9	12.9
By MS/MS	By MS/MS	By matching	By matching	By matching	21	21
By matching	By MS/MS	By matching	By matching	By matching	6.7	6.7
By matching	By MS/MS	By MS/MS	By matching	By MS/MS	2.6	1.8
By MS/MS	By MS/MS	By matching	By matching	By matching	21.7	21.7
By matching	By MS/MS	By matching	By matching	By matching	0.8	0.7
By MS/MS	By MS/MS	By MS/MS	By MS/MS	By MS/MS	33.7	33.7
By MS/MS	By MS/MS	By MS/MS	By MS/MS	By MS/MS	9.9	9.9
By matching	By MS/MS	By matching	By MS/MS	By matching	1.2	1.2
By MS/MS	By MS/MS	By matching	By matching	By MS/MS	5.9	5.9
By MS/MS	By MS/MS	By MS/MS	By MS/MS	By MS/MS	19.3	19.1
By MS/MS	By MS/MS	By MS/MS	By MS/MS	By MS/MS	11.7	11.7
By MS/MS	By MS/MS	By MS/MS	By matching	By MS/MS	64.6	64.6
By matching	By matching	By matching	By matching	By matching	33.3	0
By MS/MS	By MS/MS	By matching	By matching	By matching	26	20.2
By MS/MS	By MS/MS	By matching	By matching	By matching	13.3	13.5
By MS/MS	By MS/MS	By MS/MS	By MS/MS	By MS/MS	17.5	21.2
By MS/MS	By MS/MS	By MS/MS	By MS/MS	By MS/MS	34.9	39.2

By MS/MS	By MS/MS	By MS/MS	By MS/MS	By MS/MS	60.8	61.8
By MS/MS	By MS/MS	By MS/MS	By MS/MS	By MS/MS	7.1	7.1
By MS/MS	By MS/MS	By matching	By matching	By matching	18.8	18.1
By MS/MS	By MS/MS	By matching	By matching	By matching	27.2	27.2
By MS/MS	By MS/MS	By matching	By matching	By matching	1.2	1.2
By MS/MS	By MS/MS	By MS/MS	By matching	By MS/MS	18.9	18.9
By matching	By MS/MS	By matching	By matching	By matching	2.1	2.1
By MS/MS	By MS/MS	By MS/MS	By MS/MS	By MS/MS	24.3	24.3
By MS/MS	By MS/MS	By matching	By matching	By matching	20.6	18.9
By MS/MS	By MS/MS	By MS/MS	By MS/MS	By MS/MS	4.8	4.8
By MS/MS	By MS/MS	By MS/MS	By MS/MS	By MS/MS	31.7	31.2
By MS/MS	By MS/MS	By MS/MS	By MS/MS	By MS/MS	35.6	27.2
By MS/MS	By MS/MS	By MS/MS	By matching	By MS/MS	32.7	32.7
By MS/MS	By MS/MS	By matching	By matching	By matching	2.5	2.5
By MS/MS	By MS/MS	By MS/MS	By MS/MS	By MS/MS	6.6	6.6
By matching	By matching	By MS/MS	By MS/MS	By matching	0	0
By matching	By matching	By matching	By matching	By matching	11.5	11.5
By MS/MS	By matching	By MS/MS	By MS/MS	By matching	58.1	55.2
By MS/MS	By MS/MS	By MS/MS	By MS/MS	By MS/MS	28.6	28.6
By MS/MS	By MS/MS	By MS/MS	By MS/MS	By MS/MS	42.1	42.1
By MS/MS	By MS/MS	By MS/MS	By MS/MS	By MS/MS	43.4	43.4
By MS/MS	By MS/MS	By MS/MS	By matching	By MS/MS	25.5	25.5
By MS/MS	By MS/MS	By MS/MS	By MS/MS	By MS/MS	44.7	44.7
By MS/MS	By MS/MS	By MS/MS	By MS/MS	By MS/MS	41.1	41.1
By MS/MS	By MS/MS	By MS/MS	By MS/MS	By MS/MS	42.8	51.3
By MS/MS	By MS/MS	By matching	By MS/MS	By MS/MS	19.6	19.6
By MS/MS	By MS/MS	By MS/MS	By MS/MS	By MS/MS	52.5	52.5
By matching	By matching	By MS/MS	By matching	By MS/MS	0	0
By MS/MS	By MS/MS	By MS/MS	By MS/MS	By MS/MS	10.9	20.2
By MS/MS	By MS/MS	By MS/MS	By MS/MS	By MS/MS	22	22
By MS/MS	By MS/MS	By MS/MS	By matching	By MS/MS	23.8	23.8
By MS/MS	By MS/MS	By MS/MS	By MS/MS	By MS/MS	42.1	42.1
By MS/MS	By MS/MS	By MS/MS	By MS/MS	By MS/MS	27.7	32.2
By MS/MS	By MS/MS	By MS/MS	By MS/MS	By MS/MS	44.9	44.9
By MS/MS	By MS/MS	By matching	By matching	By matching	16.8	22.4
By MS/MS	By matching	By matching	By matching	By matching	7.4	7.4
By MS/MS	By MS/MS	By matching	By matching	By MS/MS	19.6	19.6
By MS/MS	By MS/MS	By MS/MS	By MS/MS	By MS/MS	31.3	31.3
By MS/MS	By MS/MS	By MS/MS	By MS/MS	By MS/MS	43.8	43.8
By MS/MS	By MS/MS	By MS/MS	By MS/MS	By MS/MS	39.7	39.7
By MS/MS	By MS/MS	By MS/MS	By MS/MS	By MS/MS	46.1	46.1
By MS/MS	By MS/MS	By MS/MS	By MS/MS	By MS/MS	27.9	27.9
By MS/MS	By MS/MS	By matching	By matching	By matching	9.6	9.6
By MS/MS	By MS/MS	By MS/MS	By MS/MS	By MS/MS	51.7	51.7
By MS/MS	By MS/MS	By MS/MS	By MS/MS	By matching	11.1	11.1

By MS/MS	By MS/MS	By MS/MS	By MS/MS	By MS/MS	58.1	55.2
By MS/MS	By MS/MS	By matching	By matching	By MS/MS	32.4	32.4
By MS/MS	By MS/MS	By MS/MS	By MS/MS	By MS/MS	56.1	51.9
By MS/MS	By MS/MS	By MS/MS	By MS/MS	By MS/MS	53.1	55.1
By MS/MS	By MS/MS	By MS/MS	By MS/MS	By MS/MS	65.8	62.7
By matching	By MS/MS	By matching	By matching	By matching	11.3	11.3
By MS/MS	By MS/MS	By MS/MS	By MS/MS	By MS/MS	36.2	28
By MS/MS	By MS/MS	By matching	By matching	By MS/MS	23.9	23.9
By MS/MS	By MS/MS	By matching	By matching	By matching	5.4	5.4
By MS/MS	By MS/MS	By MS/MS	By MS/MS	By MS/MS	32.2	31.6
By matching	By matching	By MS/MS	By matching	By MS/MS	0	0
By MS/MS	By MS/MS	By MS/MS	By MS/MS	By MS/MS	22.7	22.7
By matching	By matching	By MS/MS	By matching	By MS/MS	0	0
By MS/MS	By MS/MS	By MS/MS	By MS/MS	By MS/MS	29.3	30.5
By MS/MS	By MS/MS	By MS/MS	By MS/MS	By MS/MS	31.7	27.5
By MS/MS	By MS/MS	By matching	By matching	By matching	20.3	20.3
By MS/MS	By MS/MS	By MS/MS	By matching	By MS/MS	8.5	9.9
By MS/MS	By MS/MS	By MS/MS	By MS/MS	By MS/MS	8.9	8.9
By MS/MS	By MS/MS	By MS/MS	By MS/MS	By MS/MS	10.2	10.2
By MS/MS	By MS/MS	By MS/MS	By MS/MS	By matching	10	8.5
By MS/MS	By MS/MS	By MS/MS	By MS/MS	By MS/MS	24	24.8
By MS/MS	By MS/MS	By matching	By MS/MS	By MS/MS	10.4	10.9
By MS/MS	By MS/MS	By MS/MS	By MS/MS	By MS/MS	8.5	8.5
By MS/MS	By MS/MS	By matching	By matching	By matching	40.4	36.3
By MS/MS	By MS/MS	By MS/MS	By MS/MS	By MS/MS	28.1	28.1
By MS/MS	By MS/MS	By MS/MS	By MS/MS	By MS/MS	10.6	10.6
By MS/MS	By MS/MS	By MS/MS	By MS/MS	By MS/MS	56.4	56.4
By MS/MS	By MS/MS	By MS/MS	By MS/MS	By MS/MS	67.8	67.8
By matching	By matching	By MS/MS	By matching	By matching	0	0
By MS/MS	By MS/MS	By MS/MS	By MS/MS	By MS/MS	5.5	6.8
By MS/MS	By MS/MS	By MS/MS	By MS/MS	By MS/MS	1.3	2.9
By MS/MS	By MS/MS	By MS/MS	By MS/MS	By MS/MS	34.1	34.1
By MS/MS	By MS/MS	By MS/MS	By matching	By matching	6.7	6.9
By MS/MS	By MS/MS	By MS/MS	By MS/MS	By MS/MS	10.1	10.2
By MS/MS	By MS/MS	By matching	By matching	By matching	9.1	9.9
By MS/MS	By MS/MS	By MS/MS	By MS/MS	By MS/MS	2.1	2.1
By MS/MS	By MS/MS	By matching	By matching	By MS/MS	6.2	6.2
By MS/MS	By MS/MS	By matching	By matching	By matching	11.3	11.3
By MS/MS	By MS/MS	By MS/MS	By MS/MS	By MS/MS	30.5	30.5
By MS/MS	By MS/MS	By matching	By matching	By matching	30.9	30.9
By MS/MS	By MS/MS	By MS/MS	By MS/MS	By MS/MS	18	18
By MS/MS	By MS/MS	By MS/MS	By MS/MS	By MS/MS	11	10.3
By MS/MS	By MS/MS	By matching	By matching	By matching	43.8	40.7
By MS/MS	By MS/MS	By matching	By matching	By matching	3	3.5
By MS/MS	By MS/MS	By MS/MS	By MS/MS	By MS/MS	19	22.9

By MS/MS	By MS/MS	By MS/MS	By MS/MS	By MS/MS	20.9	20.9
By matching	By matching	By matching	By MS/MS	By MS/MS	0	0
By MS/MS	By MS/MS	By matching	By matching	By matching	21.2	21.2
By MS/MS	By MS/MS	By MS/MS	By MS/MS	By MS/MS	21.6	21.6
By MS/MS	By MS/MS	By matching	By matching	By matching	50.8	47.6
By MS/MS	By MS/MS	By MS/MS	By MS/MS	By MS/MS	7.2	7.2
By MS/MS	By MS/MS	By matching	By matching	By matching	18.3	22.1
By matching	By MS/MS	By MS/MS	By MS/MS	By MS/MS	4.4	4.4
By MS/MS	By MS/MS	By MS/MS	By MS/MS	By MS/MS	13.6	13.6
By MS/MS	By MS/MS	By matching	By matching	By matching	2.1	2.1
By MS/MS	By MS/MS	By MS/MS	By MS/MS	By MS/MS	43.6	43.6
By matching	By matching	By MS/MS	By matching	By matching	1.6	4.2
By MS/MS	By MS/MS	By MS/MS	By MS/MS	By MS/MS	14.2	13.5
By matching	By MS/MS	By matching	By matching	By matching	0.5	0.5
By MS/MS	By MS/MS	By MS/MS	By MS/MS	By MS/MS	4.7	7
By MS/MS	By MS/MS	By matching	By matching	By matching	4.2	4.2
By MS/MS	By MS/MS	By MS/MS	By matching	By MS/MS	26.2	26.4
By MS/MS	By MS/MS	By MS/MS	By MS/MS	By MS/MS	35.6	35.5
By MS/MS	By MS/MS	By MS/MS	By matching	By MS/MS	2.4	4.9
By MS/MS	By MS/MS	By matching	By matching	By MS/MS	0.9	0.9
By MS/MS	By MS/MS	By matching	By matching	By matching	65.8	67.1
By MS/MS	By MS/MS	By MS/MS	By MS/MS	By MS/MS	37.6	39.1
By MS/MS	By MS/MS	By matching	By matching	By matching	5.2	3.8
By MS/MS	By MS/MS	By MS/MS	By MS/MS	By MS/MS	14.4	14.4
By MS/MS	By MS/MS	By matching	By MS/MS	By matching	65.2	66.9
By MS/MS	By MS/MS	By MS/MS	By matching	By MS/MS	7.7	7.7
By matching	By MS/MS	By matching	By matching	By matching	0.7	0.7
By MS/MS	By MS/MS	By MS/MS	By MS/MS	By MS/MS	1.6	1.6
By MS/MS	By MS/MS	By matching	By matching	By matching	21.2	21.2
By MS/MS	By MS/MS	By MS/MS	By matching	By matching	4.4	4.7
By MS/MS	By MS/MS	By MS/MS	By MS/MS	By MS/MS	59	59
By matching	By MS/MS	By matching	By MS/MS	By matching	2.7	2.7
By MS/MS	By MS/MS	By MS/MS	By MS/MS	By MS/MS	49.7	49.7
By MS/MS	By MS/MS	By MS/MS	By MS/MS	By MS/MS	35.3	35.3
By MS/MS	By MS/MS	By MS/MS	By MS/MS	By MS/MS	16.8	16.8
By MS/MS	By MS/MS	By MS/MS	By MS/MS	By MS/MS	10.7	9.2
By MS/MS	By MS/MS	By matching	By matching	By matching	6.9	5
By MS/MS	By MS/MS	By MS/MS	By MS/MS	By MS/MS	29.5	29.5
By MS/MS	By MS/MS	By matching	By matching	By matching	37.9	32.5
By MS/MS	By MS/MS	By MS/MS	By MS/MS	By MS/MS	5.7	7.6
By matching	By matching	By MS/MS	By matching	By matching	0	0
By MS/MS	By MS/MS	By matching	By matching	By matching	24.5	24.5
By matching	By MS/MS	By MS/MS	By MS/MS	By MS/MS	2.1	0
By matching	By matching	By MS/MS	By matching	By matching	0	0
By MS/MS	By MS/MS	By matching	By matching	By matching	2.2	2.2

By MS/MS	By MS/MS	By MS/MS	By MS/MS	By MS/MS	4.7	4.7
By matching	By MS/MS	By matching	By matching	By matching	3.4	3.4
By MS/MS	By MS/MS	By MS/MS	By matching	By matching	36.5	36.5
By MS/MS	By MS/MS	By matching	By matching	By matching	16.6	16.6
By MS/MS	By MS/MS	By matching	By matching	By MS/MS	15.5	15.5
By MS/MS	By MS/MS	By MS/MS	By MS/MS	By MS/MS	18.7	18.7
By MS/MS	By MS/MS	By MS/MS	By matching	By MS/MS	31.3	30.7
By MS/MS	By MS/MS	By matching	By matching	By matching	36.4	33.3
By MS/MS	By MS/MS	By MS/MS	By MS/MS	By MS/MS	55.8	55.8
By MS/MS	By MS/MS	By MS/MS	By MS/MS	By MS/MS	32.5	32.5
By MS/MS	By MS/MS	By matching	By matching	By matching	10.8	7.8
By matching	By matching	By matching	By matching	By MS/MS	0	0
By MS/MS	By matching	By matching	By matching	By matching	10.6	10.6
By MS/MS	By MS/MS	By matching	By matching	By matching	9.7	9.7
By MS/MS	By matching	By MS/MS	By MS/MS	By MS/MS	14.7	14.7
By MS/MS	By MS/MS	By matching	By matching	By matching	5.4	5.4
By MS/MS	By MS/MS	By matching	By matching	By matching	2.2	2.9
By matching	By matching	By MS/MS	By MS/MS	By MS/MS	0	0
By MS/MS	By MS/MS	By matching	By matching	By matching	12.5	12.5
By MS/MS	By MS/MS	By MS/MS	By MS/MS	By MS/MS	24.6	22.1
By MS/MS	By MS/MS	By MS/MS	By MS/MS	By MS/MS	31.6	28.5
By MS/MS	By MS/MS	By MS/MS	By MS/MS	By MS/MS	37.7	37.7
By MS/MS	By MS/MS	By matching	By matching	By matching	5.3	5.3
By matching	By matching	By matching	By MS/MS	By MS/MS	0	0
By matching	By MS/MS	By matching	By matching	By matching	5.1	5.1
By MS/MS	By MS/MS	By matching	By matching	By matching	3.6	3.6
By MS/MS	By MS/MS	By MS/MS	By MS/MS	By MS/MS	26.6	23.1
By matching	By MS/MS	By MS/MS	By MS/MS	By matching	3.6	3.2
By matching	By matching	By matching	By matching	By MS/MS	0	0
By matching	By matching	By matching	By matching	By MS/MS	0	0
By MS/MS	By MS/MS	By MS/MS	By MS/MS	By MS/MS	19.9	19.9
By MS/MS	By MS/MS	By matching	By MS/MS	By MS/MS	6.5	6.5
By MS/MS	By MS/MS	By matching	By matching	By matching	5.3	5.3
By MS/MS	By MS/MS	By MS/MS	By MS/MS	By MS/MS	27.3	27.1
By MS/MS	By MS/MS	By MS/MS	By MS/MS	By MS/MS	7.1	7.1
By MS/MS	By MS/MS	By matching	By matching	By matching	3.9	3
By MS/MS	By MS/MS	By matching	By matching	By matching	2.2	2.2
By MS/MS	By MS/MS	By matching	By matching	By MS/MS	9.8	10.5
By MS/MS	By MS/MS	By matching	By matching	By matching	11.3	11.3
By MS/MS	By MS/MS	By MS/MS	By matching	By MS/MS	7	7
By MS/MS	By MS/MS	By matching	By matching	By matching	12.3	15.7
By MS/MS	By MS/MS	By matching	By matching	By matching	2.4	3
By matching	By MS/MS	By matching	By matching	By matching	4.8	4.8
By MS/MS	By MS/MS	By MS/MS	By MS/MS	By MS/MS	47.8	47.8
By MS/MS	By MS/MS	By matching	By MS/MS	By matching	5.5	5.5

By MS/MS	By MS/MS	By matching	By matching	By MS/MS	43.5		39.6	
By MS/MS	By MS/MS	By matching	By matching	By matching	7.1		7.1	
By MS/MS	By MS/MS	By MS/MS	By MS/MS	By MS/MS	10.2		8.1	
By matching	By matching	By MS/MS	By matching	By matching		0		0
By MS/MS	By MS/MS	By matching	By matching	By matching	13.8		13.8	
By MS/MS	By MS/MS	By matching	By matching	By matching	23.8		19.7	
By MS/MS	By MS/MS	By matching	By matching	By matching	7.6		6.7	
By MS/MS	By MS/MS	By MS/MS	By MS/MS	By MS/MS	7.7		7.3	
By MS/MS	By MS/MS	By matching	By matching	By matching	8.5		8.5	
By MS/MS	By MS/MS	By matching	By matching	By matching	3.4		3.4	
By MS/MS	By MS/MS	By matching	By matching	By matching	14.8			19
By matching	By MS/MS	By matching	By matching	By matching		1		1
By MS/MS	By MS/MS	By MS/MS	By matching	By MS/MS	18.2		18.2	
By MS/MS	By MS/MS	By MS/MS	By MS/MS	By MS/MS	15.3		15.3	
By matching	By MS/MS	By matching	By matching	By matching		0	6.6	
By matching	By matching	By matching	By MS/MS	By MS/MS		0		0
By MS/MS	By MS/MS	By matching	By matching	By MS/MS	48.1		48.1	
By MS/MS	By MS/MS	By matching	By matching	By matching		7		7
By matching	By matching	By MS/MS	By MS/MS	By MS/MS		0		0
By MS/MS	By matching	By matching	By matching	By MS/MS		6		6
By MS/MS	By MS/MS	By matching	By matching	By matching	8.4		8.4	
By MS/MS	By MS/MS	By matching	By matching	By MS/MS	4.5		4.5	
By matching	By MS/MS	By MS/MS	By matching	By matching	2.4			1
By MS/MS	By MS/MS	By matching	By matching	By matching	19.7		18.1	
By MS/MS	By MS/MS	By MS/MS	By MS/MS	By MS/MS	4.8		4.8	
By matching	By matching	By matching	By matching	By MS/MS	0.8		0.8	
By MS/MS	By matching	By matching	By matching	By matching	0.2		0.2	
By matching	By MS/MS	By matching	By matching	By matching	4.3		4.3	
By matching	By matching	By MS/MS	By matching	By matching		0		0
By matching	By MS/MS	By matching	By matching	By matching	4.7		4.7	
By MS/MS	By MS/MS	By matching	By matching	By matching	5.7		5.7	
By MS/MS	By MS/MS	By MS/MS	By MS/MS	By MS/MS		18		18
By MS/MS	By MS/MS	By MS/MS	By MS/MS	By MS/MS	14.8		14.8	
By MS/MS	By MS/MS	By MS/MS	By MS/MS	By MS/MS	15.4		15.4	
By MS/MS	By MS/MS	By MS/MS	By MS/MS	By MS/MS	31.9		35.8	
By MS/MS	By MS/MS	By MS/MS	By MS/MS	By MS/MS	33.6		33.6	
By MS/MS	By MS/MS	By matching	By matching	By matching	5.4		5.4	
By matching	By matching	By MS/MS	By MS/MS	By MS/MS		0		0
By MS/MS	By MS/MS	By MS/MS	By MS/MS	By MS/MS	24.2		22.8	
By MS/MS	By MS/MS	By matching	By matching	By matching	66.1		62.9	
By MS/MS	By MS/MS	By matching	By matching	By matching	1.9		1.9	
By MS/MS	By MS/MS	By MS/MS	By MS/MS	By MS/MS	36.6		36.6	
By MS/MS	By MS/MS	By matching	By matching	By matching	24.4		24.4	
By MS/MS	By MS/MS	By MS/MS	By MS/MS	By MS/MS	10.7		11.8	
By matching	By matching	By matching	By matching	By MS/MS	2.4		2.4	

By MS/MS	By MS/MS	By matching	By matching	By matching	16.9	16.9
By MS/MS	By MS/MS	By matching	By matching	By matching	3.9	4.2
By matching	By matching	By MS/MS	By matching	By matching	0	0
By MS/MS	By MS/MS	By MS/MS	By matching	By matching	10.5	13.1
By MS/MS	By MS/MS	By MS/MS	By MS/MS	By MS/MS	8.4	8.4
By MS/MS	By MS/MS	By matching	By matching	By MS/MS	15.9	15.9
By MS/MS	By matching	By matching	By matching	By matching	11.7	11.7
By MS/MS	By MS/MS	By matching	By matching	By matching	6.5	6.5
By MS/MS	By MS/MS	By MS/MS	By MS/MS	By MS/MS	39.4	39.4
By MS/MS	By matching	By matching	By matching	By matching	7.7	7.7
By MS/MS	By MS/MS	By MS/MS	By matching	By MS/MS	8.9	8.9
By MS/MS	By MS/MS	By MS/MS	By MS/MS	By MS/MS	6.4	6.4
By MS/MS	By MS/MS	By matching	By MS/MS	By MS/MS	2.3	2.3
By MS/MS	By MS/MS	By matching	By matching	By matching	2.1	2.1
By MS/MS	By MS/MS	By matching	By matching	By MS/MS	5	5
By MS/MS	By matching	By matching	By matching	By MS/MS	4.1	4.1
By MS/MS	By MS/MS	By MS/MS	By MS/MS	By MS/MS	15	12.3
By MS/MS	By MS/MS	By matching	By matching	By matching	5.9	7.5
By MS/MS	By MS/MS	By MS/MS	By matching	By MS/MS	16.2	16.2
By MS/MS	By MS/MS	By matching	By matching	By matching	27.4	27.4
By MS/MS	By MS/MS	By MS/MS	By MS/MS	By MS/MS	22.8	22.8
By MS/MS	By MS/MS	By matching	By matching	By matching	1.8	1.8
By matching	By matching	By matching	By MS/MS	By MS/MS	0	0
By MS/MS	By MS/MS	By matching	By matching	By matching	16.4	16.7
By MS/MS	By MS/MS	By matching	By matching	By matching	11.5	11.5
By MS/MS	By MS/MS	By MS/MS	By MS/MS	By MS/MS	7.4	7.4
By MS/MS	By MS/MS	By MS/MS	By MS/MS	By MS/MS	28.3	28.3
By MS/MS	By MS/MS	By MS/MS	By matching	By MS/MS	0.3	0.3
By matching	By MS/MS	By matching	By matching	By matching	2.3	2.3
By MS/MS	By MS/MS	By matching	By matching	By MS/MS	4.3	2.7
By MS/MS	By MS/MS	By matching	By matching	By matching	19.1	19.1
By MS/MS	By MS/MS	By matching	By matching	By matching	18.2	18.2
By MS/MS	By MS/MS	By MS/MS	By MS/MS	By MS/MS	16.5	16.5
By MS/MS	By MS/MS	By MS/MS	By matching	By MS/MS	13.2	13.2
By MS/MS	By MS/MS	By MS/MS	By MS/MS	By MS/MS	42.8	41.3
By matching	By matching	By MS/MS	By MS/MS	By MS/MS	1.9	1.9
By MS/MS	By MS/MS	By MS/MS	By MS/MS	By MS/MS	17.8	17.8
By MS/MS	By MS/MS	By MS/MS	By MS/MS	By MS/MS	16.2	17.3
By MS/MS	By MS/MS	By MS/MS	By MS/MS	By MS/MS	26.4	26.4
By MS/MS	By MS/MS	By MS/MS	By MS/MS	By MS/MS	4.2	3.4
By MS/MS	By MS/MS	By MS/MS	By MS/MS	By MS/MS	46.8	50.8
By MS/MS	By MS/MS	By matching	By matching	By matching	1.4	1.4
By MS/MS	By MS/MS	By matching	By MS/MS	By MS/MS	7.4	7.4
By MS/MS	By MS/MS	By matching	By matching	By matching	5.2	5.2
By MS/MS	By MS/MS	By MS/MS	By matching	By MS/MS	2.1	2.1

By MS/MS	By MS/MS	By matching	By matching	By matching	8.7	8.7
By MS/MS	By MS/MS	By matching	By matching	By matching	6.1	4.8
By MS/MS	By MS/MS	By matching	By matching	By matching	13.4	13.4
By MS/MS	By MS/MS	By MS/MS	By MS/MS	By MS/MS	11.2	9.6
By matching	By matching	By MS/MS	By MS/MS	By MS/MS	0	0
By MS/MS	By MS/MS	By matching	By MS/MS	By MS/MS	21.5	18
By MS/MS	By MS/MS	By MS/MS	By MS/MS	By MS/MS	28.8	28.8
By MS/MS	By MS/MS	By MS/MS	By MS/MS	By matching	8	8
By MS/MS	By MS/MS	By matching	By matching	By matching	6.9	6.9
By MS/MS	By MS/MS	By matching	By matching	By matching	37.4	37.4
By MS/MS	By MS/MS	By matching	By matching	By matching	1.5	1.5
By MS/MS	By MS/MS	By MS/MS	By MS/MS	By MS/MS	3.8	3.8
By MS/MS	By MS/MS	By MS/MS	By MS/MS	By MS/MS	8.7	7.8
By MS/MS	By MS/MS	By matching	By matching	By matching	11	11
By MS/MS	By MS/MS	By MS/MS	By matching	By MS/MS	8.6	6.4
By MS/MS	By MS/MS	By matching	By matching	By matching	7.3	7
By MS/MS	By MS/MS	By matching	By matching	By matching	9.5	9.5
By MS/MS	By MS/MS	By MS/MS	By MS/MS	By MS/MS	31.8	34.4
By matching	By matching	By matching	By matching	By MS/MS	1.2	1.2
By MS/MS	By MS/MS	By matching	By matching	By matching	8.8	8.8
By MS/MS	By MS/MS	By matching	By MS/MS	By matching	15.5	14.8
By MS/MS	By MS/MS	By matching	By matching	By matching	8.9	8.9
By MS/MS	By MS/MS	By matching	By MS/MS	By matching	12.9	12.9
By MS/MS	By MS/MS	By matching	By matching	By MS/MS	5.8	5.6
By MS/MS	By MS/MS	By matching	By matching	By matching	5.8	5.8
By MS/MS	By MS/MS	By MS/MS	By MS/MS	By MS/MS	68.2	68.2
By MS/MS	By MS/MS	By matching	By matching	By matching	7.3	7.3
By MS/MS	By MS/MS	By matching	By matching	By matching	12.3	10
By MS/MS	By MS/MS	By MS/MS	By MS/MS	By MS/MS	14.8	14.8
By MS/MS	By MS/MS	By matching	By MS/MS	By MS/MS	7.7	7.7
By MS/MS	By MS/MS	By MS/MS	By MS/MS	By MS/MS	29	29.6
By MS/MS	By MS/MS	By MS/MS	By MS/MS	By MS/MS	18.5	18.5
By MS/MS	By MS/MS	By MS/MS	By MS/MS	By MS/MS	51.7	51.7
By MS/MS	By MS/MS	By MS/MS	By MS/MS	By MS/MS	24	24
By MS/MS	By MS/MS	By MS/MS	By MS/MS	By MS/MS	16.2	16.8
By matching	By MS/MS	By MS/MS	By matching	By MS/MS	11	11
By MS/MS	By MS/MS	By MS/MS	By MS/MS	By MS/MS	36.1	36.1
By MS/MS	By MS/MS	By matching	By matching	By matching	43.7	40.6
By MS/MS	By MS/MS	By matching	By MS/MS	By MS/MS	23.4	20.3
By MS/MS	By MS/MS	By matching	By matching	By matching	14.7	16.5
By MS/MS	By MS/MS	By matching	By matching	By matching	11.2	10
By MS/MS	By MS/MS	By MS/MS	By MS/MS	By MS/MS	23.4	21.2
By MS/MS	By MS/MS	By matching	By MS/MS	By matching	55.8	55.8
By MS/MS	By MS/MS	By matching	By MS/MS	By MS/MS	17.9	24.6
By matching	By matching	By matching	By matching	By MS/MS	21.6	21.6

By MS/MS	By MS/MS	By matching	By matching	By matching	14.5	12.6
By MS/MS	By MS/MS	By MS/MS	By MS/MS	By MS/MS	15	15
By MS/MS	By MS/MS	By matching	By matching	By matching	6.9	6.9
By MS/MS	By MS/MS	By matching	By matching	By matching	21.7	21.7
By MS/MS	By MS/MS	By MS/MS	By matching	By MS/MS	5.7	7.5
By MS/MS	By MS/MS	By matching	By matching	By matching	12.4	12.4
By MS/MS	By MS/MS	By matching	By matching	By matching	12.5	10.3
By matching	By MS/MS	By matching	By matching	By MS/MS	38.4	38.4
By MS/MS	By MS/MS	By MS/MS	By matching	By MS/MS	4.8	6.4
By MS/MS	By MS/MS	By MS/MS	By matching	By MS/MS	10	8.4
By matching	By MS/MS	By matching	By matching	By MS/MS	2.8	0
By MS/MS	By MS/MS	By matching	By matching	By MS/MS	25.8	22.5
By MS/MS	By MS/MS	By matching	By matching	By matching	1	1
By MS/MS	By MS/MS	By MS/MS	By MS/MS	By MS/MS	72.1	72.1
By MS/MS	By MS/MS	By MS/MS	By MS/MS	By MS/MS	19.3	20.9
By MS/MS	By MS/MS	By matching	By matching	By matching	4.1	4.1
By matching	By MS/MS	By matching	By matching	By MS/MS	2.6	2.6
By MS/MS	By MS/MS	By matching	By matching	By MS/MS	39.3	38.7
By MS/MS	By MS/MS	By MS/MS	By matching	By MS/MS	10.8	10.8
By MS/MS	By MS/MS	By MS/MS	By matching	By MS/MS	5.6	5.6
By MS/MS	By MS/MS	By MS/MS	By MS/MS	By MS/MS	10.8	11.3
By MS/MS	By MS/MS	By MS/MS	By MS/MS	By MS/MS	21.4	21.4
By MS/MS	By MS/MS	By matching	By matching	By matching	4.6	4.6
By MS/MS	By MS/MS	By matching	By matching	By matching	4.1	4.1
By MS/MS	By MS/MS	By matching	By matching	By matching	23.5	20.1
By MS/MS	By MS/MS	By MS/MS	By MS/MS	By MS/MS	46.1	44.5
By MS/MS	By MS/MS	By MS/MS	By MS/MS	By MS/MS	24.1	20.4
By MS/MS	By MS/MS	By MS/MS	By MS/MS	By MS/MS	3.2	3.2
By MS/MS	By MS/MS	By MS/MS	By MS/MS	By MS/MS	60.4	60.4
By MS/MS	By MS/MS	By matching	By matching	By matching	2.3	2.3
By MS/MS	By MS/MS	By matching	By matching	By matching	14	12.3
By matching	By matching	By matching	By matching	By matching	4.8	4.8
By MS/MS	By MS/MS	By MS/MS	By matching	By MS/MS	10.7	10.7
By MS/MS	By MS/MS	By MS/MS	By MS/MS	By MS/MS	16.7	16.7
By MS/MS	By matching	By matching	By matching	By matching	1.1	1.1
By MS/MS	By MS/MS	By MS/MS	By matching	By MS/MS	11.3	11.3
By matching	By MS/MS	By matching	By matching	By matching	4.8	2.4
By MS/MS	By MS/MS	By MS/MS	By MS/MS	By MS/MS	47.4	44.6
By MS/MS	By MS/MS	By matching	By matching	By matching	7.6	6.3
By MS/MS	By MS/MS	By matching	By matching	By matching	1.1	1.1
By MS/MS	By MS/MS	By matching	By matching	By matching	10.4	10.4
By MS/MS	By MS/MS	By matching	By matching	By matching	6.7	7.2
By MS/MS	By MS/MS	By MS/MS	By MS/MS	By MS/MS	9.6	12.3
By MS/MS	By MS/MS	By MS/MS	By MS/MS	By matching	7.4	13.7
By matching	By MS/MS	By MS/MS	By MS/MS	By MS/MS	3.6	3.6

By MS/MS	By MS/MS	By matching	By matching	By matching	7.2	7.2
By MS/MS	By MS/MS	By MS/MS	By MS/MS	By MS/MS	6	7.4
By matching	By MS/MS	By matching	By matching	By MS/MS	3.6	0
By matching	By matching	By MS/MS	By MS/MS	By MS/MS	0	0
By MS/MS	By MS/MS	By matching	By matching	By matching	10.9	10.9
By MS/MS	By MS/MS	By matching	By matching	By matching	3.6	3.6
By MS/MS	By MS/MS	By matching	By matching	By matching	12.7	12.7
By MS/MS	By MS/MS	By matching	By matching	By matching	12.6	12.6
By MS/MS	By MS/MS	By MS/MS	By MS/MS	By MS/MS	22.4	21.2
By MS/MS	By MS/MS	By matching	By matching	By matching	19.3	19.3
By MS/MS	By MS/MS	By matching	By matching	By MS/MS	3.1	3.1
By MS/MS	By MS/MS	By MS/MS	By MS/MS	By matching	15.8	15.8
By MS/MS	By MS/MS	By matching	By matching	By MS/MS	25.8	25.8
By MS/MS	By MS/MS	By MS/MS	By MS/MS	By MS/MS	8.2	8.2
By MS/MS	By MS/MS	By matching	By matching	By matching	7.6	7.6
By MS/MS	By MS/MS	By matching	By matching	By matching	9.2	9.2
By MS/MS	By MS/MS	By MS/MS	By MS/MS	By MS/MS	12	12
By MS/MS	By MS/MS	By matching	By matching	By matching	3.1	3.1
By MS/MS	By MS/MS	By MS/MS	By MS/MS	By MS/MS	13.8	15.8
By matching	By matching	By MS/MS	By matching	By matching	0	0
By MS/MS	By MS/MS	By matching	By matching	By matching	2	2
By matching	By matching	By MS/MS	By MS/MS	By MS/MS	0	0
By MS/MS	By MS/MS	By MS/MS	By matching	By MS/MS	11.5	12.7
By MS/MS	By MS/MS	By matching	By matching	By MS/MS	12.3	12.3
By MS/MS	By MS/MS	By matching	By matching	By matching	5.7	5.7
By MS/MS	By MS/MS	By MS/MS	By MS/MS	By MS/MS	7.9	7.9
By MS/MS	By MS/MS	By matching	By matching	By matching	7.5	8.7
By MS/MS	By MS/MS	By matching	By matching	By matching	21.8	23.8
By MS/MS	By MS/MS	By MS/MS	By MS/MS	By MS/MS	45.6	45.6
By MS/MS	By MS/MS	By MS/MS	By MS/MS	By MS/MS	14	14
By MS/MS	By MS/MS	By MS/MS	By MS/MS	By MS/MS	54.6	48.2
By MS/MS	By MS/MS	By matching	By matching	By matching	0	2.7
By MS/MS	By MS/MS	By matching	By matching	By matching	5.3	5.3
By MS/MS	By MS/MS	By matching	By matching	By matching	6.5	4.8
By MS/MS	By MS/MS	By MS/MS	By MS/MS	By MS/MS	30.6	26.7
By MS/MS	By MS/MS	By MS/MS	By MS/MS	By MS/MS	36.4	31.2
By MS/MS	By MS/MS	By MS/MS	By MS/MS	By MS/MS	20.6	21.4
By MS/MS	By MS/MS	By MS/MS	By MS/MS	By MS/MS	44	40.6
By MS/MS	By MS/MS	By matching	By matching	By matching	38.7	37.2
By MS/MS	By MS/MS	By MS/MS	By MS/MS	By MS/MS	38.3	38.3
By MS/MS	By MS/MS	By matching	By matching	By matching	7.9	7.9
By MS/MS	By MS/MS	By MS/MS	By MS/MS	By MS/MS	24.3	24.3
By MS/MS	By MS/MS	By MS/MS	By matching	By MS/MS	20	20
By MS/MS	By MS/MS	By MS/MS	By MS/MS	By MS/MS	6.7	6.7
By MS/MS	By MS/MS	By MS/MS	By MS/MS	By MS/MS	21.9	21.9

By MS/MS	By MS/MS	By MS/MS	By MS/MS	By MS/MS	10.3	10.3
By MS/MS	By MS/MS	By MS/MS	By MS/MS	By MS/MS	11.3	11.3
By MS/MS	By MS/MS	By matching	By matching	By matching	14.9	15.1
By MS/MS	By MS/MS	By matching	By MS/MS	By MS/MS	4.2	4.2
By MS/MS	By MS/MS	By MS/MS	By MS/MS	By MS/MS	33.2	30.8
By MS/MS	By MS/MS	By MS/MS	By MS/MS	By MS/MS	17.1	18.8
By MS/MS	By MS/MS	By MS/MS	By MS/MS	By MS/MS	13.3	11.8
By MS/MS	By MS/MS	By MS/MS	By MS/MS	By MS/MS	4.9	7.4
By matching	By MS/MS	By MS/MS	By matching	By matching	0	0
By MS/MS	By MS/MS	By MS/MS	By MS/MS	By MS/MS	0	0
By matching	By matching	By matching	By matching	By MS/MS	0	0
By MS/MS	By matching	By matching	By matching	By matching	0	0
By matching	By matching	By matching	By MS/MS	By matching	0	0
By MS/MS	By matching	By MS/MS	By MS/MS	By MS/MS	0	0
By matching	By MS/MS	By MS/MS	By matching	By matching	0	0
By matching	By matching	By matching	By MS/MS	By matching	0	0
By matching	By MS/MS	By matching	By matching	By matching	0	0
By MS/MS	By MS/MS	By MS/MS	By MS/MS	By matching	0	0
By matching	By matching	By matching	By MS/MS	By matching	0	0
By MS/MS	By MS/MS	By matching	By matching	By matching	0	0
By matching	By matching	By matching	By matching	By matching	0	0
By matching	By matching	By MS/MS	By MS/MS	By matching	0	0
By matching	By matching	By matching	By matching	By matching	0	0
By MS/MS	By MS/MS	By matching	By MS/MS	By MS/MS	0	0
By MS/MS	By MS/MS	By MS/MS	By matching	By MS/MS	0	0
By MS/MS	By MS/MS	By MS/MS	By MS/MS	By MS/MS	0	0
By MS/MS	By MS/MS	By matching	By matching	By matching	67.1	67.1
By MS/MS	By MS/MS	By MS/MS	By MS/MS	By MS/MS	7.9	9.5
By MS/MS	By MS/MS	By MS/MS	By MS/MS	By MS/MS	34.3	26.7

Sequence co	Sequence co	Sequence co	Sequence co	Intensity	Intensity CBX	Intensity CBX
51.1	25.6	35.6	35.6	181920000	37997000	46480000
26.4	5.2	5.2	16.1	144830000	30288000	34019000
17.8	11.5	11.5	11.5	65663000	6822400	9961500
10.8	15.1	15.1	15.1	799800000	43606000	42116000
16.8	16.8	14.1	16.8	298500000	47583000	51376000
4.4	2.2	2.2	2.2	44183000	8016900	9819100
4.8	0	0	0	14242000	3718400	5756600
44.5	37.2	29.3	37.2	2235200000	376510000	583250000
15.6	16.2	16.2	19.9	319930000	37367000	52446000
5.6	0	0	0	27026000	9749300	7155500
5.7	7.6	9.7	9.7	122640000	13426000	14578000
8.8	13.8	13.8	13.8	74370000	10034000	10993000
16.2	15.3	10.4	15.3	365910000	54824000	48385000
1.4	1.5	1.5	1.5	15889000	2715200	2769400
13.4	0	0	0	110580000	35617000	37487000
7.8	0	0	0	9070500	6000500	0
28.8	12.7	14.8	26.2	556660000	136780000	79114000
0	0	0	0	16911000	16911000	0
22.2	5	5.1	6	1190200000	320230000	381060000
6.3	5.6	3.9	3.9	217330000	45462000	61995000
41.3	34.1	41.3	41.3	4833500000	1044800000	1186600000
35.4	30	30	30	62803000	10031000	14707000
17	18.3	15.1	18.3	2566700000	364460000	401910000
10.6	0	0	0	65953000	19558000	20878000
20	0	0	0	634540000	190430000	215790000
6.8	6.8	6.8	6.8	47113000	9568800	11690000
20.5	4.5	4.5	4.5	214280000	51903000	62597000
13.4	0	0	0	21401000	5961300	8498600
7.1	1.6	3.6	3.6	207390000	43921000	89125000
28.3	0	0	0	272460000	77789000	96017000
29.5	3.4	0	0	948310000	323970000	262640000
9	0	0	0	18272000	8362600	9909700
4.7	0	0	0	119230000	54751000	0
3.9	3.9	3.9	3.9	67232000	10761000	11218000
3.9	2.8	2.8	2.8	122380000	13672000	26971000
4.7	0	0	0	50690000	17650000	20335000
14.7	12.2	14.7	14.7	314140000	70025000	61283000
4.2	0	0	0	71727000	16675000	28498000
16.2	0	0	0	31385000	9744500	10353000
9.2	6.3	3.8	7.9	340510000	92030000	84966000
1.2	0	0	0	30564000	8806500	10891000
15.8	0	0	0	492880000	147120000	164670000
3.8	5.2	0	9	42104000	5565900	6679700
5.1	10.9	10.9	15.2	322040000	28848000	46776000

10.8	3.3	4.5	4.5	576750000	153090000	188540000
2.2	1.5	1.5	1.5	93531000	10592000	12621000
7.9	9.8	9.8	9.8	71724000	7486200	8367400
1.3	0	0	0	59160000	16710000	22593000
26.7	0	0	0	221140000	69139000	80804000
9.6	0	0	0	49320000	14275000	17454000
8.9	7.3	3.3	3.3	13549000	3695200	4126400
1	4.5	4.5	4.5	62402000	5904600	6123200
9.8	12	9.8	12	248070000	38701000	61857000
11	0	0	0	165280000	47507000	53908000
56	7.5	7.5	7.5	503230000	124620000	136690000
3.9	1.8	0	1.8	88325000	37161000	14475000
27.1	23.6	23.6	23.6	490130000	117720000	119340000
30.4	0	0	0	11689000	2922300	3084800
1.6	34.8	7.1	4	988740000	0	0
2.3	0	0	1.7	31408000	8084800	8984300
9.9	14.3	16.4	14.3	353760000	20120000	7482600
34.5	40	26.4	26.4	238900000	51664000	66569000
30.5	0	0	0	24613000	9451000	6176200
53.2	44.4	44.4	44.4	2982500000	585400000	797620000
41	0	0	0	5961100000	1922600000	1786000000
9	13	4.6	8.6	140860000	24407000	51134000
1.7	1.7	0	1.7	700680000	116730000	153510000
8.8	0	0	0	20770000	8768200	3037800
24	0	0	0	146220000	41623000	50271000
52.7	46.9	52.9	54.4	1,1381E+10	1560100000	1637300000
6.1	0	0	0	80430000	23095000	29162000
10.8	6.2	6.9	5.7	2842400000	58900000	59388000
28.4	20.1	20.1	20.1	616430000	148360000	102080000
8.3	0	0	0	17296000	4344800	4890800
4.7	0	0	0	26231000	7696200	11203000
7.9	0	0	0	43721000	16291000	6812600
7.9	0	0	0	356110000	116130000	134240000
48.1	31.6	31.6	31.6	981840000	196470000	293070000
8.5	2.8	2.8	2.8	108920000	30868000	36729000
21.7	0	0	0	49728000	14571000	18166000
28.1	18.3	18.3	18.3	240670000	52770000	53739000
16.8	25.1	19.9	25.1	245750000	31428000	38667000
6.1	1.4	0	0	41371000	12640000	13766000
36.3	20.1	20.1	20.1	1509100000	314140000	354220000
6.5	0	0	0	40710000	12163000	12022000
7.4	1.5	1.5	1.5	175020000	48208000	57017000
15.3	2	2	0	228970000	50230000	64162000
3.1	0	0	0	37607000	9211700	17253000
14.6	5.1	0	5.1	376230000	86894000	126190000

0	11.2	11.2	11.2	35776000	0	0
15.6	15.6	15.6	20.3	342340000	49272000	97538000
16.7	10.6	11.3	16.7	139600000	24889000	31352000
11.5	12.5	24	24	130570000	15667000	22460000
2.9	0	0	0	198520000	47422000	76827000
3.8	3.8	3.8	3.8	55849000	8125000	9284300
19.3	23.8	23.8	24.3	1965000000	255720000	305770000
4.8	0	0	0	12908000	5174600	0
9.3	11.4	11.4	8.6	189630000	20120000	26006000
41	0	0	0	210500000	58929000	76736000
6.7	0	0	0	26193000	10959000	9729300
26.7	8.6	8.6	8.6	931710000	286680000	291980000
27.6	20.7	20.7	20.7	135530000	32281000	40306000
15.3	37.8	31.6	32.1	209240000	17446000	16588000
6.1	8.3	8.3	8.3	168680000	18399000	22845000
23.9	0	0	0	1387400000	446570000	402800000
4.7	0	0	0	28299000	7605000	8888400
21.1	27	24.5	32.4	1543200000	107020000	143740000
22.3	19.9	16.7	23.1	789590000	175610000	166090000
0	15.9	9.2	9.2	14970000	0	0
20.7	0	0	0	103960000	37484000	21157000
25.8	10.3	5.7	10.3	278570000	67622000	79672000
47	19	23	23	1208700000	267750000	297220000
4.4	4.4	4.4	4.4	51432000	14372000	5372000
9.6	0	0	0	137420000	35051000	49151000
6.8	0	0	0	47040000	15132000	16615000
4.2	2.3	4.2	4.2	137210000	24605000	30902000
19.9	9.6	9.6	12	1140000000	320030000	314820000
28.1	14.7	15.9	15.9	922600000	192140000	243310000
32	27.7	27.7	30.5	2677900000	442290000	538920000
12.9	0	0	0	92558000	24410000	34633000
3.8	4.1	4.1	4.1	50737000	13079000	13491000
4.5	0	0	0	43705000	12362000	7462300
8.5	0	0	0	13376000	4197100	3925300
1.4	0	0	0	21559000	6104700	9353800
4.6	0	0	0	94465000	31612000	32450000
22.4	0	0	0	14390000	7072100	0
10.6	10.6	10.6	10.6	24263000	3178800	3773600
67.8	40.7	42.7	46	4,2024E+10	1,064E+10	1,178E+10
0	3.9	3.9	7.8	18659000	0	0
8	0	0	0	224020000	69874000	79782000
18.2	18.2	18.2	18.2	88393000	15914000	18981000
20.6	0	0	0	19090000	6165200	5540500
57.5	42.2	39.5	42.2	3298300000	679710000	653870000
0	3.2	3.2	0	24429000	6168700	5788200

14.2		14.2		14.2		14.2		150850000	55298000	15971000
23.3		5.3		5.3		5.3		730950000	159310000	216850000
	50	39.4		39.4		39.4		2837300000	467590000	546920000
8.8		11.4		16.5		16.5		173740000	10971000	36308000
5.1			0		0		0	40943000	11579000	13659000
5.9		5.9		5.9		5.9		51703000	7588100	9470900
2.4			0		0		0	35468000	13343000	10087000
27.7			0		0		0	953150000	289180000	340080000
19.7			0		0		0	139270000	38886000	49367000
11.4		25.7		25.7		25.7		92175000	7674300	9990500
24.7			0		0		0	289620000	80933000	103890000
2.4			0		0		0	142600000	27182000	32278000
4.5		1.4		1.4		1.4		59803000	13019000	14453000
16.8			0		0		0	132660000	41707000	52641000
2.4		8.2		8.2		8.2		98086000	7092300	7221900
12.6			0		0		0	17935000	5666200	4784700
17.3		20.2			19	21.5		1801800000	167890000	274320000
5.9		2.3			0	2.3		52419000	14422000	15465000
17.8		15.8		13.1		16.2		1158000000	195770000	244300000
30.1		6.9		6.3		8.2		2467300000	642540000	834350000
8.7		2.8		2.8		2.8		113360000	25190000	31517000
3.4		3.4			0	3.4		31539000	7201600	6550600
10.4			5		5	6.5		187470000	32799000	43565000
46.5		35.2		36.4		44.6		1,0382E+10	2245700000	2264900000
12.1			0		0		0	11839000	2443000	4646500
6.3			0		0		0	98001000	33960000	29230000
9.2			0		0		0	576570000	146340000	206650000
21.5			0		0	15.4		35180000	9406200	8592100
27.8		19.4		11.7		19.4		1320300000	286910000	373520000
14.1		14.1		14.1		14.1		29972000	6258600	7307600
1.5			0		0		0	36996000	9901800	13058000
7.9			0		0		0	35745000	10111000	12542000
	24	10.4		10.4		10.4		393710000	86917000	112360000
9.9			0		0		0	165790000	46669000	56304000
	0	1.5		1.5		1.5		48817000	9486200	10399000
8.6		4.9			0	4.9		53606000	10507000	12585000
	8		0		0		0	7672000	0	0
	24	5.2		3.1		6.8		369730000	94132000	115670000
7.4			0		0		0	258440000	76072000	86885000
4.3		1.4		2.9		2.9		124860000	25020000	34166000
	13	6.3			13		13	62275000	6687400	15051000
	12	6.4		6.4		6.4		51937000	0	12592000
39.1		20.3		39.1		39.1		1039400000	234570000	260010000
	0	33.7		23.2		23.2		130870000	0	0
24.4			22	22.8		29.5		1913100000	274060000	348700000

56.4	56.4	56.4	56.4	##########	301050000	389310000
7.6	13.6	16.3	16.3	509110000	43628000	55031000
15.8	16.3	16.3	18.7	950830000	159270000	180340000
7	0	0	0	44631000	13726000	14764000
0	4.1	0	0	47324000	0	0
9.1	0	0	0	195010000	59081000	68068000
28.6	11.7	14.3	14.3	619400000	153800000	172850000
68.7	68.7	68.7	68.7	2,2365E+10	4321400000	4694100000
24	17	17	17	154860000	42506000	50139000
6	0	0	0	80244000	24874000	25633000
16.3	2.1	3.8	3.8	244300000	64102000	73165000
14.5	0	0	0	37348000	11175000	13384000
15.7	0	0	0	276780000	90161000	87679000
17.6	0	0	0	8847900	3181000	2385900
12.3	0.9	0.9	0.9	614780000	177130000	195260000
5.4	0	0	0	10543000	3088800	3236500
23.7	2.4	4.3	4.3	490240000	141110000	160940000
27.2	0	0	0	99011000	32875000	32063000
2.5	0	0	0	40414000	11795000	13141000
26.2	8.5	7.6	8.5	1565800000	381770000	542820000
21.6	2.6	0	0	262310000	72563000	89658000
46.5	49	50.4	50.4	4436700000	267840000	401470000
2.5	0	0	0	38235000	11703000	13127000
53.3	55.9	54.2	63.6	1,1353E+10	1526300000	1904900000
5.5	0	0	0	78842000	26560000	24979000
17.8	17.3	21	21	798170000	121500000	138840000
0	10.7	10.7	10.7	21746000	0	0
35.9	35.9	35.9	35.9	5569800000	1706300000	836080000
20.9	26.6	32.5	36.5	945680000	93269000	74320000
33.2	15.3	19.9	19.9	716790000	172180000	231200000
45.6	45.9	38.1	40.7	3762100000	225990000	346600000
29.4	33.6	33.6	29.4	420940000	58520000	68843000
23.5	20.9	20.9	20.9	292960000	73593000	89141000
12.6	11.1	11.1	10	1150400000	244710000	267230000
0	10.6	10.6	10.6	12250000	0	0
21.1	21.1	21.1	21.1	48834000	8647100	10064000
12	6.7	6.7	6.7	66621000	12699000	12751000
67.9	66.3	66.3	66.3	1,9673E+10	5776900000	5901000000
3.6	8.5	8.5	5.7	97479000	10445000	14939000
43.2	39.2	39.2	39.2	319710000	41508000	57136000
32.1	7.5	11.9	16.9	3872800000	787860000	435450000
0	4.6	4.6	4.6	9905100	0	0
14.3	14.1	10.3	15.2	198680000	29474000	38780000
4.8	4.8	4.8	4.8	63400000	13707000	16435000
34.4	20.8	20.8	20.8	1717400000	355290000	496090000

28.2	28.2	25.5	28.2	###########	224040000	253990000
0	5	5	0	45798000	14666000	20075000
13.4	5	5	5	25151000	3621400	7151000
28.4	0	0	3.8	201080000	61312000	60591000
6.9	0	0	0	317120000	89821000	105330000
18.4	3.4	3.4	0	174040000	53695000	49173000
9.3	13.4	13.4	13.4	247500000	29682000	34861000
6.1	0	6.1	6.1	27465000	5467600	5214700
8.7	0	0	0	54662000	15305000	18711000
0	6.7	6.7	6.7	65704000	0	0
15.6	1.5	0	1.5	341510000	112980000	100060000
1.5	0	0	0	22073000	5562600	6851200
0	24.3	24.3	24.3	60324000	0	0
8.9	0	0	0	78181000	23077000	25520000
1.7	0	0	0	91210000	8286900	72849000
23.4	0	0	0	250830000	61484000	90037000
16.6	15.6	15.6	15.6	145290000	26047000	34454000
9.6	10.7	7.9	10.7	289760000	48981000	62080000
8.5	0	0	0	24921000	7475600	8716500
46.5	2	1.1	2	4590800000	1214400000	1457100000
5.8	0	0	0	213140000	63651000	70956000
23.2	2	2	2	338330000	83231000	107870000
23.4	23.4	23.4	23.4	59337000	0	0
3.7	0	0	0	33715000	11640000	11561000
10.2	0	0	0	55777000	13690000	19767000
12.2	0	0	0	21111000	6156700	7950900
2.7	5.8	5.8	5.8	49885000	5003600	5277500
5	0	0	0	37439000	13689000	11041000
4.2	0	0	0	142080000	38553000	44059000
2.4	0	0	0	15169000	4021300	5135400
7.7	0	0	0	21062000	7807300	6226200
17	0	0	0	51525000	14573000	22147000
0	9.5	9.5	9.5	137130000	0	0
38.9	37.6	27.1	35.3	1783600000	335900000	372870000
10.7	5.9	3	10.7	88061000	13358000	17502000
7.7	14.5	7.7	14.5	127630000	11427000	17936000
17.5	8.3	8.3	8.3	60953000	4668000	5050700
6.8	0	0	0	71811000	26146000	17990000
7.6	0	0	0	37852000	12307000	11624000
3.6	0	0	0	22415000	6202700	5868000
37	10.5	10.5	10.5	595410000	155630000	191950000
0	24.7	14.6	32.3	147790000	0	0
11.7	0	0	0	638360000	179090000	228090000
2.9	0	0	0	22794000	7733000	7701500
8.5	16.1	16.1	16.1	985740000	116620000	151800000

8.6	0	0	0	59816000	17413000	19714000
I7;S0F3A9;S0F2I4;S0F2B7;S0F2B1;R9WYX8;R9WXA9;R9WAS6;R9UU99;R9UTP9;R9UR01;R9RVK4;R9RIA8;R9RI97;R9RFL1;R7						
LA9;F8R1A8;F8R1A5;F8R1A3;F8R1A1;F8R1A0;F8R199;F8R197;F8R194;F8R190;F8R188;F8R186;F8R183;F8R181;F8R177;F8R						
54VA55;S0F3H7;S0F3A9;S0F2I4;S0F2B7;S0F2B1;R9WYX8;R9WXA9;R9WAS6;R9UU99;R9UTP9;R9UR01;R9RVK4;R9RIA8;R9RI9						
LA9;F8R1A8;F8R1A5;F8R1A3;F8R1A1;F8R1A0;F8R199;F8R197;F8R194;F8R190;F8R188;F8R186;F8R183;F8R181;F8R177;F8R						
;273;273;273;273;273;273;273;273;273;273;273;273;273;273;273;273;273;273;273;273;273;273;273;273;273;273;273;273						
1441;2692	True;True	1470;2744	6743;6744;6	5189;9522;9	5189;9523	
27.9	21.6	17.3	15.3	349220000	59110000	69046000
13.9	0	0	0	410520000	120830000	144630000
0	0	0	0	3145100	3145100	0
3.9	0	0	0	19389000	3958900	3554100
20.3	0	0	0	21890000	5986900	6782300
5.3	0	0	0	16466000	5360800	5206500
8.9	8.9	8.9	8.9	110250000	14540000	18024000
36.1	0	0	7.1	104400000	22690000	37652000
0	5.2	0	0	11993000	0	0
6.8	0	6.8	6.8	216390000	64945000	0
25.1	19.7	19	19	1597200000	241480000	327890000
23.3	0	0	0	28069000	9179000	8435600
5.7	5.7	5.7	5.7	44622000	11862000	15859000
2.4	0	0	0	28829000	8835400	10382000
25.2	6.5	6.5	6.5	219370000	57601000	72794000
3.9	0	0	0	15726000	4769800	5435900
24.9	0	0	0	317360000	100340000	115100000
17.6	0	0	0	199910000	56571000	45254000
8.5	8.5	8.5	8.5	193080000	44710000	61422000
57.3	53.6	52.7	55.3	3,8943E+10	5939300000	7729100000
3.1	13.8	12.8	14.7	314750000	7057200	11244000
10.9	0	0	0	9992100	2834700	3178600
11.8	13.5	16.6	16.6	317610000	39619000	50802000
13.7	0	0	0	114060000	33132000	38588000
4.9	0	0	0	62541000	18957000	20659000
0.3	0	0	0	45829000	11783000	17334000
2.9	0	0	0	72884000	19462000	25604000
1.9	0	0	0	15547000	3128600	5964700
9.2	0	0	0	8451400	2830800	2380200
23.8	23.8	23.8	23.8	840550000	155160000	109870000
4	0	0	0	109260000	32593000	34083000
0	34.2	34.2	34.2	322330000	0	0
2	6.5	6.5	6.5	69186000	6084000	7859600
3.9	0	0	0	13644000	3540400	4543800
5.1	0	0	0	35636000	11656000	11497000
2.5	0	0	1.1	32859000	18432000	6993900
3.9	3.9	3.9	3.9	40061000	6879600	8985400
21.6	0	0	21.6	27412000	7049900	7068700

30.1	30.1	30.1	30.1	199080000	29717000	36927000
3.6	0	0	0	23350000	2165100	12911000
6.4	0	0	0	9790800	3143400	3330400
0	7.7	7.7	7.7	7801300	0	0
12	0	0	0	602940000	164680000	222680000
12.2	12.2	12.2	12.2	114550000	14627000	23908000
0	15.7	0	0	6010800	0	0
10.6	5.7	5.7	5.7	14572000	4173100	5159000
0	0	0	0	41473000	19776000	21696000
30.2	16.3	16.3	16.3	316190000	76346000	59723000
15.7	12.9	10	10	236260000	25717000	34630000
24.5	10.2	8.4	12.3	946270000	194300000	245110000
0	5.9	5.9	5.9	10231000	0	0
4.7	6.9	6.9	9.1	200770000	24194000	26399000
5.7	2.7	2.7	2.7	134650000	31664000	32895000
6.8	0	0	0	40450000	11629000	13024000
4.2	0	0	0	14583000	3624200	5939400
3	3	3	3	33714000	3782300	5910700
28.7	26.7	36	36	231190000	28081000	48739000
1.7	33.9	12.1	6.4	801220000	0	0
8.1	1.6	1.6	1.6	116110000	27487000	37098000
33.9	26.2	26.2	26.2	932700000	140610000	197080000
2	2	2	2	42258000	8049500	9400100
8	2.6	0	3.2	108530000	29613000	43386000
7	0	0	0	225640000	63044000	85904000
10.5	0	0	0	20824000	7215500	3800100
12.5	0	0	0	28935000	7497000	11033000
20.9	18.9	18.9	18.9	3852800000	431440000	630490000
6.4	0	0	0	130830000	32092000	39324000
11.8	4	4	4	89512000	25339000	30188000
11.3	11.3	11.3	11.3	84440000	10881000	16593000
2.8	0	0	0	21816000	6216400	6886300
4	0	0	0	74644000	23108000	24103000
7.6	14	14	14	145060000	6318100	8745200
3.6	4.7	4.7	4.7	20983000	3784500	5906200
7	0	0	0	29201000	9835700	9204200
36.6	29.7	26.9	29.7	569100000	141810000	144620000
48.4	0	0	0	4,1724E+10	1,218E+10	1,5454E+10
0	9.3	9.3	9.3	10813000	0	0
2.6	5.7	5.7	4.4	105830000	10446000	8998800
8.4	0	0	0	67880000	20793000	22666000
0.8	0	0	0	7585800	2378500	2424600
8.5	0	0	0	28657000	8964800	9791400
8.7	5.4	8.7	8.7	101690000	21774000	23452000
2.7	0	0	0	11524000	3156800	3882600

8	0	0	0	###########	595760000	827750000
3.4	10.8	8.2	6.8	141110000	3493500	16704000
5	0	0	0	29779000	8844100	8597800
53.3	46.7	46.7	46.7	2263500000	287690000	324810000
13.1	0	0	0	68875000	15622000	21154000
0	11.5	11.5	11.5	29811000	0	0
4.2	0	0	0	74635000	21443000	27344000
2.8	0	0	0	29085000	9765800	8998600
18.9	3.2	5.7	7.1	452070000	126700000	129030000
3.6	3.6	3.6	3.6	50752000	8112600	10281000
28.9	28.9	23.2	33.2	769190000	116940000	141430000
14.3	18.8	19.8	21.8	889210000	105300000	129730000
4.3	12.4	11.4	13.8	268570000	20439000	22112000
17.2	7.5	7.5	7.5	2036400000	477330000	568530000
22.7	22.7	15.6	22.7	144620000	23344000	24054000
4	0	0	0	117160000	33053000	44412000
12.1	15.2	12.1	15.2	475000000	44123000	43782000
1.7	0	0	0	22157000	6636100	8252500
55.8	54.2	57	60.8	8616200000	1343500000	1332600000
19.2	0	0	0	235010000	74556000	69517000
27.9	22.7	26.3	26.3	409650000	56598000	95305000
3.4	0	0	0	39166000	6421800	9900100
8	0	0	0	50861000	15685000	14231000
23.2	18.2	10.6	18.2	236470000	45712000	74685000
39.2	17.8	19.2	19.2	4606500000	1326700000	1403300000
5.3	0	0	0	12540000	4037500	3688700
0	16	0	0	1285700000	0	0
11	3.1	3.1	2.3	334010000	99411000	81966000
5.1	0	0	0	20579000	6675000	6398000
32.5	4.2	4.2	4.2	1053800000	295540000	368160000
5.9	3.1	3.1	3.1	26774000	5514400	6502800
16.9	16.9	16.9	16.9	28256000	5298100	6034100
11.1	21.2	12.5	21	435920000	90570000	74019000
4.7	0	0	0	11470000	3281900	4586100
17.8	17.8	17.8	17.8	223480000	34489000	43991000
3.1	2.4	2.4	2.4	120180000	23278000	26692000
5.8	3.3	5.8	5.8	150740000	33013000	38712000
0	8.2	11.8	11.8	60503000	0	0
7.5	0	0	0	78256000	17861000	28326000
1.9	3.3	3.3	3.3	27792000	3159700	2179100
27.8	19.3	15.9	21.5	440590000	79047000	101100000
18.8	0	0	0	88110000	26429000	27099000
5.7	0	0	0	25086000	6952200	9097900
33	39.3	40.7	40.7	1067500000	157440000	175300000
36.3	36.3	27.4	36.3	1659900000	300840000	425410000

22	0	0	0	511370000	177090000	135720000
9.7	0	0	0	217290000	66546000	78417000
20.9	8	8	9.2	789820000	175910000	222100000
0	0	2.3	2.3	9903600	0	0
49.8	17.8	21.9	21.9	1062900000	260390000	309430000
11.4	11.4	11.4	11.4	26314000	5368900	3649600
13.5	20.9	20.9	20.9	627210000	72180000	83671000
29.7	29.7	29.7	29.7	275140000	24584000	51847000
18.9	0	0	0	39315000	20290000	0
36.6	36.6	20.8	36.6	1063700000	203040000	258730000
2.6	0	0	0	112310000	31162000	37146000
11.6	7.8	7.8	7.8	38885000	7837300	12634000
6.5	0	0	0	74526000	19265000	26865000
4.4	0	0	0	64457000	18297000	23915000
14.7	5.3	5.3	5.3	163950000	45676000	49049000
0.8	0	0	0	70278000	21400000	21762000
28	28	28	28	13828000	0	0
2.8	2.8	2.8	2.8	124200000	13718000	24754000
2.3	0	0	0	64278000	18918000	23089000
0	0.9	0.9	0.9	439550000	0	0
0	33.7	11.4	3.1	154330000	0	0
14.4	8.8	9.9	11.6	1773400000	441260000	386750000
6.9	0	0	0	43967000	13429000	12126000
16.4	0	0	0	707320000	184340000	213880000
2.3	1.8	3.4	4	143290000	21622000	12610000
2.2	5.9	7.4	7.4	165660000	11130000	13069000
6.8	0	0	0	11717000	3768700	3183200
0	10.2	4.3	4.3	26190000	0	0
15.2	8.2	8.9	8.9	975190000	216230000	264010000
2	0	0	0	21427000	6383100	6529500
0	1.6	1.6	1.6	24098000	0	0
6.9	0	0	0	162270000	47577000	58243000
6.9	0	0	0	43170000	18845000	10290000
0	1.5	5.2	5.2	41970000	0	0
9.1	1.1	0	1.1	135450000	37770000	43733000
16.5	8.5	0	0	170610000	37702000	45814000
12.2	0	0	0	48196000	19207000	13354000
5.1	1.5	1.5	1.5	83287000	17806000	21465000
9.6	1.7	3.1	3.1	168900000	38730000	48823000
22.8	22.8	22.8	22.8	273600000	39376000	38500000
5	0	0	0	9182900	2580900	3058800
6	0	0	0	60371000	10091000	22992000
42.8	50.3	44.1	44.7	732060000	49239000	20132000
18.3	0	0	0	213480000	94948000	26630000
1.8	1.8	1.8	1.8	273960000	25803000	34969000

18.6	3.8	4	5.9	270770000	77099000	79083000
15.1	15.2	15.2	10.8	119650000	11127000	16262000
3.6	0	0	0	72158000	27773000	22516000
69.5	37.7	40.3	41.8	205540000	43533000	49958000
8.2	0	0	0	42289000	10564000	17957000
27.3	23.6	23.6	23.6	1450100000	260630000	276350000
6	3	3	3	108350000	33428000	36971000
8.1	0	0	0	82617000	23003000	29686000
12.7	16.7	13.1	32.1	306370000	17326000	26996000
21.8	15.3	16.7	21.8	369960000	77968000	98528000
2.7	0	0	0	15975000	4789500	4932300
18.2	0	0	0	173690000	47167000	61545000
40.4	33	31.4	33.9	3577100000	650590000	719550000
14.1	0	0	0	54139000	22487000	15218000
10.6	0	0	0	50946000	10072000	17080000
0	8.1	0	8.1	7950100	0	0
52.1	66.7	66.7	66.7	1988600000	236560000	235900000
6.1	6.1	6.1	6.1	65101000	10882000	15978000
8.8	0	0	0	64461000	14555000	17079000
42.2	19.9	19.9	19.9	1796500000	360040000	495050000
22.3	18	23.7	23.7	512550000	92996000	117000000
11.3	0	0	0	128750000	44226000	46517000
2.6	2.4	2.4	3.9	190920000	37925000	43115000
36.5	19.8	19.8	19.8	219480000	34088000	46015000
14.7	0	0	0	92567000	30683000	24172000
34.1	20.5	20.5	20.5	854360000	184830000	245500000
0	18.6	18.6	37.1	15643000	0	0
23.8	16.1	16.1	23.8	518880000	98542000	151510000
19	24.6	25	25	1009400000	103660000	122220000
10.6	24.2	19.7	17.6	630680000	61792000	74371000
25.2	7.3	5.8	5.8	1858100000	486730000	510390000
4.7	4.7	4.7	4.7	63887000	9706800	11761000
27.2	24.7	24	27.2	1142800000	187790000	208300000
1.5	0	0	0	22910000	6518200	7486300
4.9	0	0	0	36953000	10102000	13024000
10.5	8.8	10.5	10.5	9361500	1829800	0
5	5	5	5	26430000	4252700	5958900
16.7	1.4	1.4	1.4	184060000	54845000	57889000
9.2	4.7	4.7	4.7	434870000	100340000	115490000
13.1	0	0	1.3	136830000	39556000	46379000
14.2	0	0	0	46909000	14278000	11115000
16.1	0	3.4	3.4	276860000	88508000	82709000
10.1	3.9	6.4	6.4	65510000	16939000	19101000
18.2	28.5	36.2	36.2	1041400000	138470000	99902000
17.2	0	0	0	19486000	4999200	6528300

6.7	6.7	6.7	6.7	81696000	12844000	12253000
5.2	5.2	5.2	5.2	15504000	2312100	3052600
6.9	0	0	0	130750000	36476000	46929000
13.1	8.8	8.8	8.8	182990000	43063000	46788000
9.4	9.8	7.5	7.5	177810000	25370000	35892000
3.7	37	16.3	5.3	910680000	0	0
9.9	2.5	2.5	2.5	45341000	12116000	15737000
21.6	27.7	29.1	32.4	726030000	64962000	75513000
9.2	0	0	0	0	0	0
6.1	0	0	0	120450000	37246000	36755000
6.4	7.7	7.7	7.7	89082000	5418300	5068800
12.1	16.8	13.4	20.4	665990000	84736000	111950000
11.9	16.3	13.2	16.3	1264300000	141270000	183470000
43.9	27.8	24.9	27.4	2787500000	720780000	711870000
0	4.1	4.1	4.1	29327000	3402100	5898000
14.9	3.4	1.4	1.4	442030000	121530000	122180000
2.4	1.1	1.1	1.1	38756000	8492500	11564000
1.3	0	0	0	86115000	21591000	29403000
9.3	0	0	0	31921000	8645900	11756000
8.1	0	0	0	60765000	24318000	18298000
8.1	0	0	0	165620000	45051000	59218000
11.5	12.4	11.8	11.8	652710000	159350000	117420000
4	0	0	0	31430000	18055000	0
4.7	4.1	4.1	4.1	104580000	17781000	21340000
20.3	18.8	20.3	20.3	66461000	14246000	13361000
45.7	37	38.3	43.4	2,2855E+10	6169500000	6044400000
2.3	2.3	2.3	2.3	14735000	2063500	2852400
10.5	0	0	0	228530000	63179000	89258000
21.1	4	3.3	4	647000000	182050000	196420000
3	3	3	3	31627000	5446300	5019200
17.3	2.3	2.3	2.3	51244000	14068000	18119000
14.1	2.4	2.4	2.4	352060000	92624000	110770000
10.8	0	0	0	94106000	3946800	79952000
23.4	6.2	7.6	20.7	146940000	27294000	40966000
31.2	31.2	18.8	31.2	751950000	110410000	138570000
13.6	0	0	0	8820100	2140900	3007300
6.4	0	0	0	27599000	8081900	9712100
7.3	23.8	19.1	23.6	753280000	92995000	84737000
10.6	5.6	5.6	5.6	45830000	8464600	11491000
6.7	16.4	16.4	16.4	52389000	5202300	5749500
0	16.5	16.5	16.5	11806000	0	0
51.4	27.2	27.2	27.2	1867600000	404080000	527470000
45.6	18.4	25.6	18.4	696800000	101140000	213580000
0	13.2	13.2	13.2	13361000	0	0
51.5	34	34	34	3331800000	754790000	938090000

46.4	30.4	0	17.4	258150000	55864000	60984000
15.1	8.5	8.5	8.5	1565200000	245830000	898610000
40.8	40.8	40.8	40.8	3078000000	414270000	506150000
5.4	3.1	3.1	3.1	82821000	15952000	22398000
46.3	46.3	33.5	46.3	9520500000	1409200000	1645900000
2.2	0	0	0	25457000	8216300	8564000
15.2	12	10.6	12	6068900000	2099000000	2404600000
12.7	0	0	0	249310000	70293000	74091000
4.2	26.9	22.4	26.9	440510000	10780000	15563000
5.9	0	0	0	6935600	1532400	2463400
14.9	14.9	14.9	14.9	2433600000	376130000	477070000
23.9	30.4	24.4	34	1927700000	217990000	298820000
10.8	0	0	0	28095000	8748500	9371000
8.4	5	3.1	3.1	61259000	11706000	14029000
11.6	5.8	5.8	5.8	86705000	18180000	22965000
2	4.5	4.5	4.5	59463000	5963600	3849000
11.8	11.8	11.8	11.8	65009000	10392000	14455000
24.5	6.5	6.5	6.5	514730000	146230000	161310000
39	46.3	38.5	39	47896000	0	0
1.7	0	0	0	12938000	3603700	4228200
24.3	3	3	3	531180000	162960000	185770000
5.5	0	0	0	10502000	2851000	4263300
2.6	0	0	0	6610900	0	2850100
4.4	0	0	0	35479000	8312500	15003000
15.2	1.5	1.5	3.7	370810000	98240000	119090000
2.5	0	0	0	1,3314E+10	3463400000	6193600000
7.5	4.6	5.5	5.5	3343100000	94018000	110110000
6.1	6.1	3.5	6.1	143270000	20981000	28821000
31.4	27.8	27.8	27.8	789340000	166820000	170360000
17.9	0	0	0	311370000	82970000	106920000
10.2	0	0	0	121770000	40119000	39226000
7.6	9.5	9.5	9.5	25864000	4822500	4022100
0	0	0	0	2444500	0	2444500
8.3	0	0	0	236110000	65456000	76376000
8.3	3.1	4.3	4.3	481050000	154780000	88303000
0	10.1	10.1	10.1	56530000	10066000	0
45.7	44.4	39.7	50	1540400000	281340000	306710000
11	11	20.5	20.5	96393000	4852400	5689600
8.7	2.2	6.9	6.9	135230000	19894000	38057000
7.8	0	0	0	35842000	10771000	11018000
36.9	30.6	36.9	36.9	2025700000	286590000	335270000
22.4	10.9	0	10.9	212720000	53333000	66625000
51.9	44.9	38.5	50.6	4356100000	1258700000	1398700000
6.5	0	0	0	63623000	24352000	19348000
25.4	22.9	25.3	24.7	2780000000	460920000	611470000

9.5	5	3	5	253150000	55285000	63656000
8.7	0	0	0	789880000	257760000	271860000
9.9	0	0	0	368220000	102780000	125780000
35	0	0	0	5280000000	1567100000	1793900000
6.3	0	0	0	48465000	13894000	12933000
11.9	0	11.9	11.9	21864000	5607200	6483300
44.5	4.3	9.5	9.5	846030000	277040000	239490000
15	2.6	2.6	2.6	232280000	71346000	70173000
38.1	16.2	16.2	16.2	593590000	195470000	174520000
11.3	10.6	9.3	9.3	668040000	99493000	123460000
4.3	13.9	13.9	13.9	168080000	16296000	16962000
18.4	13.6	13.6	15.1	2302500000	429540000	572590000
34.3	0	0	0	1368400000	355640000	494710000
10.6	8.7	3.9	3.9	19333000	5730400	6570200
6	5.7	7.1	7.1	166300000	30148000	39804000
2.2	0	0	3.6	7355200	1852000	1559000
10.5	4.2	4.2	4.2	793980000	289420000	218870000
36.7	38	32	39.6	503830000	71484000	70178000
5.8	0	0	0	30331000	8940700	9863300
21.4	0	0	0	524720000	158030000	178920000
1.7	0	0	0	28683000	6923100	10155000
3.4	0	0	0	0	0	0
14.7	14.6	14.6	14.6	957830000	143330000	139750000
57.2	36.1	41.3	41.3	1,0686E+10	3310200000	3435100000
16.4	5.7	0	5.7	98701000	30183000	31477000
14.2	14.7	14.7	17.2	296010000	35727000	43463000
5	0	0	0	36056000	11070000	11083000
4.9	0	0	0	56030000	22412000	12686000
2.7	0	0	0	22894000	6891500	7640500
2.1	4.7	2.7	4.7	40760000	5328100	5552800
20.4	19.2	16.5	16.9	506370000	96040000	105360000
4.6	0	0	0	94824000	29858000	26542000
1.5	0	0	0	10431000	2267100	4083400
10.7	0	0	0	77502000	24219000	26491000
9	0	0	0	60532000	20232000	22446000
8.9	0	0	0	225520000	91278000	63120000
8.1	8.1	8.1	8.1	142680000	24314000	28578000
6.5	0	0	0	11092000	3539600	3121600
3.3	3.3	3.3	3.3	99768000	18658000	22350000
7.5	0	7.5	7.5	25118000	5261100	6056500
0	3.3	0	0	38856000	0	0
7.6	7.6	7.6	7.6	112870000	18974000	22017000
5.8	0	0	0	43465000	7917200	18052000
16.3	1.7	1	1.7	925020000	275510000	304060000
7	0	0	0	51930000	18287000	10682000

30.7	12.8	10.5	12.8	672890000	126010000	182070000
37.6	45.2	38.8	45.2	46976000	0	0
44	9.1	9.1	9.1	933390000	239980000	282570000
2.9	2.9	2.9	2.9	36666000	4242300	5695500
0	7.8	7.8	7.8	16481000	0	0
13.1	17.8	9.2	11.4	195120000	20337000	30421000
20	0	0	0	52958000	16383000	18909000
17.8	12.4	12.4	12.4	64782000	11205000	13513000
30.7	0	0	0	33819000	10304000	11330000
15.8	6	15.8	15.8	28129000	8679300	0
18.1	0	0	0	575060000	159870000	204130000
0	3.2	3.2	6.4	16346000	0	0
26.8	9.8	0	17.7	89037000	23421000	26315000
0	12.9	0	0	8090200	0	0
17.9	6	6	6	149980000	39915000	57722000
8.6	11.3	12.9	15.1	281580000	32733000	41091000
3.5	0	0	0	14841000	4116900	5435100
9.3	9.3	9.3	9.3	74696000	7901300	10083000
0	0	2.2	2.2	3515800	0	0
3.6	3.9	7.6	7.6	68789000	8588400	10910000
3.8	0	0	0	47616000	12861000	16735000
3.6	3.6	3.6	7	30199000	3439700	4961200
5.8	2.3	8.1	8.1	58159000	10891000	11410000
17.4	0	0	0	22726000	7841300	7847300
10.2	12	13.5	12.3	327310000	37426000	47300000
10.3	0	0	0	24554000	9651500	6069200
42.2	37.4	37.4	37.4	224540000	44279000	51323000
0	6.2	6.2	6.2	11637000	0	0
38.1	23.1	17.7	23.1	765640000	170540000	207030000
10.7	4.7	0	0	49816000	13695000	17852000
0	13.4	3.9	13.4	44306000	0	0
0	17.2	17.2	15.2	174230000	0	0
12.9	0	0	0	81471000	24268000	29473000
25.3	0	0	0	64872000	17686000	23987000
27.1	8.2	8.2	8.2	374990000	89541000	113550000
6.5	19	23.3	23.3	663090000	35971000	39586000
19.8	0	0	0	351170000	95046000	113510000
14.3	3	3	0	96201000	30730000	31287000
6.7	0	0	0	67847000	21280000	21987000
7.4	0	0	0	8115400	2356100	2711400
13.7	0	0	0	58527000	14236000	23234000
6.4	0	0	0	24991000	9846300	9548000
23.4	0	2.6	2.6	224170000	64045000	66343000
4.7	0	0	0	12012000	3031900	4091600
3.6	0	0	0	17115000	4924900	6242200

5.4	0	0	0	63797000	19954000	23153000
2.1	3.5	3.5	3.5	33547000	0	4074200
12.8	0	0	0	281230000	77107000	80603000
0	14.9	14.9	14.9	9167000	0	0
5.8	0	0	0	12146000	3171400	3989500
8.3	13	8.3	13	137550000	14969000	16827000
8.9	9.9	9.9	14.4	90248000	10793000	13503000
5.6	8.3	8.3	8.3	116890000	15585000	20576000
23.4	3.3	3.3	3.3	2200100000	606010000	758080000
14.2	0	0	0	82659000	16483000	25690000
7.3	11.5	11.4	13.1	139460000	8783400	6957500
15.8	3.5	3.5	0	129900000	26225000	43320000
21.1	13.3	13.3	13.3	113450000	19206000	13282000
7.3	0	0	0	29259000	9735300	10600000
5.7	0	0	0	9407900	2609100	0
4.4	3.3	6.2	6.2	102350000	13734000	22707000
68.4	0	0	0	39315000	15393000	7789900
35.2	30.1	30.1	30.1	1281000000	219840000	221520000
48.2	29.2	35.1	29.2	287470000	52754000	55061000
6.9	0	0	0	10061000	3564300	3204100
7	0	0	0	98129000	32325000	26606000
8.2	8.2	8.2	8.2	32213000	5190400	9116300
0.8	0	0	0	24079000	6403300	10727000
23.5	0	0	0	1124000000	328150000	383880000
20	7.4	6.3	8.9	1077200000	264030000	322570000
8.1	1	1	1	117460000	36708000	36282000
0	17.1	17.1	17.1	113320000	0	0
16	4	6.1	6.1	753710000	181860000	278420000
5.4	16.8	16.8	16.8	163100000	17512000	18249000
16.4	34	36.8	38.5	435760000	41429000	42813000
22.4	15.8	12.2	15.6	1033100000	208470000	215470000
0	5.1	5.1	10.7	44873000	0	7549500
6.8	6.8	6.8	6.8	121110000	17505000	19960000
18.1	0	0	0	22025000	5598900	8163200
3.2	0	0	0	42092000	17583000	8987100
0	6.4	2.5	6.4	114230000	0	0
3.4	0	0	0	32059000	6076400	13323000
4.9	4.9	4.9	4.9	47635000	6361400	8465200
16.6	10.4	10.4	10.4	150090000	37395000	39330000
46.9	21.5	21.5	24.6	1617400000	450540000	443490000
9.8	4	4	4	36204000	8398900	10934000
4.5	0	0	0	30753000	8250500	13083000
11.9	0	0	0	30333000	9100000	10183000
0	17.1	17.1	17.1	2900500	0	0
3.3	3.3	3.3	3.3	26638000	5702000	5561100

16.7	9.5	9.5	9.5	80438000	18167000	25421000
7.9	0	0	0	45051000	13845000	14085000
0	0	0	8	4072600	0	0
18.2	7.6	14	14	442000000	82447000	125090000
44.2	15.3	13.5	15.3	2576400000	749650000	792440000
0	0	0	0	10662000	10662000	0
10.7	0	0	0	92465000	20451000	24231000
30	23	23	23	18904000	6090800	5022600
38.6	28.3	38.6	31	468640000	75412000	91154000
7.1	0	0	0	123410000	32910000	43684000
10.2	0	0	0	214860000	71658000	65479000
9.9	3.1	6	6	197430000	38392000	46860000
7.7	1.2	2.3	2.3	251680000	68368000	81179000
4	0	0	0	76259000	23210000	24166000
0	7.2	7.2	3.3	18606000	0	0
5.3	0	0	0	53247000	16048000	17928000
1.3	0	0	0	16752000	5000500	5521500
7	0	0	0	233260000	65113000	74430000
7.4	0	0	0	16422000	7831700	0
10.6	0	0	0	314880000	98610000	95441000
15.3	0	0	0	1574400000	449440000	511360000
22.1	0	0	7.1	58331000	18080000	13927000
10.8	7.2	7.2	7.2	222540000	19035000	30126000
6.1	6.1	6.1	6.1	55227000	6803500	12163000
8.1	0	0	0	467120000	131480000	168070000
4.2	4.3	2.2	6.3	188220000	35386000	38509000
0	2.2	2.2	2.2	26971000	0	0
15.8	0	0	0	19470000	9231700	0
6.5	0	0	0	27979000	8055100	10049000
16.4	5.8	5.8	5.8	108070000	25802000	27010000
0	11.4	11.4	11.4	37479000	0	0
44.1	26.5	26.5	26.5	232980000	48218000	68038000
19.5	0	0	0	29351000	8567300	10611000
5.4	5.4	5.4	5.4	37969000	8968800	8380100
46	0	0	0	55383000	13291000	21073000
8	8	0	8	77993000	23386000	20452000
30	3	4.6	4.6	1543400000	448680000	503400000
9.7	9.7	9.7	9.7	27736000	4679200	6401200
18.8	18.8	18.8	18.8	60249000	8628000	3675100
32.7	0	0	0	23515000	6943300	6471400
41.3	14	14	14	1453200000	380420000	478120000
12.4	24.5	19.9	24.5	284570000	43166000	47079000
0	10.9	10.9	10.9	12199000	0	0
36.6	12.5	11	14.3	406040000	103350000	105310000
7.2	0	0	1.8	142230000	38996000	46693000

1.7	34.4	9.9	3.6	403450000	0	0
1.5	6.2	1.5	1.5	64657000	0	0
1.4	1.4	1.4	1.4	288530000	42459000	57397000
14.2	13.7	9.9	12	30808000	11877000	6727200
1.5	18.4	1.5	1.5	1077800000	0	0
15.8	18.1	17.4	17.4	117180000	0	0
49.8	51.5	51.5	51.5	2,3268E+10	3885100000	3794800000
50.6	62.9	44.9	57.4	7757000000	1008700000	670570000
13.5	13.5	16.5	13.5	3439300	0	0
35.7	17.1	44.7	24.1	7625300000	477830000	393990000
5.3	19.8	25.1	15.5	1533800000	36777000	38817000
20.1	12.9	20.6	20.6	804050000	124630000	70414000
23.1	12.4	38.5	12.4	617690000	18400000	7134000
40.7	25.9	40.7	40.1	1397800000	51448000	53206000
82.7	85.2	85.3	85	7,6486E+11	1,3447E+11	1,3354E+11
10.4	0	0	0	18650000	6174700	5346800
37.6	47.7	37.1	37.6	5473400000	452820000	273350000
32.5	16.6	18	18	1064400000	304360000	303790000
51.6	60.2	57.9	60.2	1,3034E+10	817630000	1031800000
38.9	62.8	40.8	45.9	4869400000	66931000	59940000
62.7	62.7	62.7	62.7	8,3753E+10	1,257E+10	5760500000
42.7	32.9	33.7	34.1	3321100000	513840000	475870000
0	2.6	2.6	2.6	44902000	0	0
5.6	5.6	5.6	5.6	11492000	3118200	1121400
67.6	62.6	62.6	62.6	4,8249E+10	1,092E+10	1,6368E+10
77.8	64.2	62.9	65.3	41892000	8726800	0
3.6	37.9	13.4	7.1	3498800000	0	0
31.9	40.5	27.1	27.1	1005300000	192900000	166970000
1.7	34.9	12.7	3.6	8134100000	0	30232000
10.2	3.4	3.4	3.4	451930000	63842000	117080000
6.4	0	6.4	6.4	26392000	5089400	6377900
0.8	0.8	0.8	0.8	75509000	12012000	14212000
7.4	14.6	5.6	5.6	125360000	6549700	21506000
28.8	6.4	5.3	7.9	387340000	117330000	112390000
9.5	0	0	0	16710000	6434300	4149300
1.5	31	6.6	3.7	145640000	0	0
5.4	3.1	3.1	3.1	393470000	35126000	25236000
14.2	9.3	10.7	10.7	24740000	0	24740000
31.8	25.3	26.3	26.5	246490000	52159000	52391000
3.8	32.4	15	9.2	2526000000	0	0
0	9.3	0	0	5490300	0	0
4.6	4.9	4.9	4.9	147010000	26433000	31925000
3.7	37.5	12.8	9.3	1,6838E+10	1414000	19904000
16	5.8	7.7	7.7	154160000	30286000	30198000
4	4	4	6.7	0	0	0

19.7	7.1	7.1	7.1	299520000	8992100	5219300
13.6	10.8	10.1	10.8	1513000000	195380000	464280000
0	21.1	8.1	0	4767100000	0	0
1.8	9.7	3.9	1.9	922070000	0	0
34.9	0	0	0	1270200000	676780000	311890000
0	13.2	0	0	489360000	0	0
1.5	27.1	8.6	5.5	375680000	0	0
21	19.8	19.8	20.6	1356800000	217700000	295480000
16.2	0	0	0	396310000	137220000	149290000
4.4	0	0	0	75060000	19224000	26175000
1.2	0	0	0	22192000	6779200	8876900
3.2	0	0	1	64469000	17753000	18578000
6.3	6.3	6.3	6.3	28672000	5592100	6633300
25.6	25.6	5.7	18.5	280640000	47692000	62050000
10.5	14.3	14.3	14.3	294310000	29941000	35237000
27.6	0	0	0	64779000	17431000	23346000
2.9	1.7	1.7	1.7	57721000	16615000	14016000
0	7.6	13.9	17	49331000	0	0
9.7	9.7	9.7	9.7	182020000	29580000	41044000
4.1	9.9	4.1	4.1	51364000	7607600	0
9	0	0	0	159690000	45907000	55853000
5.9	5.9	5.9	5.9	217730000	43200000	62170000
3.5	5	2.7	2.7	200460000	45805000	44612000
3	0	0	0	217570000	57424000	62530000
21.1	25.4	23.5	25.4	956780000	103440000	118920000
8.7	14.1	22.4	33.7	272420000	18543000	14171000
13.5	0	0	0	17387000	6171800	4466300
26.4	7.4	7.4	7.4	436770000	115590000	115920000
0	15.9	0	0	10343000	0	0
6.7	6.7	13.3	13.3	86311000	4510900	7916000
5.6	0	0	0	245220000	108470000	12944000
9.8	1.4	1.4	1.4	67096000	14139000	25972000
9.1	11.8	11.8	11.8	397860000	56949000	66636000
10.1	12.6	12.6	14.2	115230000	10637000	13258000
27.5	12.7	12.7	18.2	445040000	99310000	113500000
4.7	3	3.2	4.7	205460000	29540000	43432000
2.7	2.2	2.2	2.2	82976000	13276000	13629000
9.3	1.4	1.4	2.8	163550000	43947000	45080000
11.5	11.1	11.1	11.1	34941000	4209300	4641100
4.7	0	0	0	24025000	3648900	5369200
17.8	8.6	8.6	8.6	134970000	34294000	38553000
28	28	0	28	87835000	21603000	24734000
43.4	23.6	23.6	23.6	776300000	199720000	243650000
27.5	0	0	0	33558000	11242000	8885100
16.9	16.9	16.9	16.9	92217000	16212000	24033000

22	0	0	0	478220000	157660000	165550000
18	0	0	0	55214000	15427000	22631000
4.5	1.7	1.7	1.7	85728000	0	0
18.3	17.3	14.7	19.2	365980000	61048000	79284000
3	0.4	0.7	0.7	169940000	48947000	47586000
0	29.5	29.5	29.5	49636000	0	0
16.2	0	0	0	70305000	19108000	25191000
34.2	5.8	5.8	5.8	2084100000	627900000	683690000
5.6	0	0	0	122910000	36882000	40272000
3.7	2.7	2.7	2.7	56787000	9138000	7638500
16.1	0	0	0	11261000	3023200	4149800
53	21.4	26.3	26.3	642090000	226000000	164040000
8.9	8.9	8.9	8.9	85560000	14821000	17162000
1.9	0	0	0	35483000	15574000	4293900
0.5	0.6	0.6	0.6	45096000	11144000	12350000
0	0	0	10.7	3432800	0	0
26.8	18.3	18.2	20.3	3132100000	666280000	836010000
33.6	25.6	29.2	29.2	1252000000	265870000	230440000
8.9	0	0	0	34149000	11291000	10285000
33.8	5.2	5.2	5.2	1075800000	312040000	347190000
12.5	10.6	7.1	11.9	405620000	74186000	83827000
18.4	0	0	0	15893000	3805300	5796200
7.2	9.4	10.4	10.4	309500000	39260000	42892000
13.2	2.6	2.6	2.6	247920000	66697000	79589000
5.9	0	0	0	37295000	11656000	11330000
14.7	0	0	0	15355000	4489100	5055000
28.6	13.1	13.2	13.2	2167800000	474390000	642780000
34.7	0	0	0	405180000	136050000	154250000
22.3	12.1	12.1	16	50482000	9634600	10447000
21.1	0	0	0	37064000	11525000	10145000
53	46.1	47.4	52.1	2,895E+10	5872800000	6106300000
3.1	11.4	7.5	7.5	18396000	0	0
7.9	0	0	0	80522000	17198000	27030000
18.5	2.3	2.3	2.3	393910000	113410000	138670000
11.9	0	0	0	106660000	34623000	32291000
15.8	27.6	27.6	27.6	564640000	69042000	88921000
5.2	0	0	0	8469000	4787600	0
4.7	1.9	0	4.2	283540000	53394000	71490000
52.3	36.4	41	46.7	1127500000	187430000	242980000
38.3	30.6	30.6	30.6	0	0	0
1.1	0	0	0	18707000	7802200	4887200
45.3	26.3	26.3	26.3	1624000000	368820000	448630000
28.8	0	16.2	16.2	137190000	20417000	45247000
10.8	26.9	23.9	30.3	536280000	26805000	36326000
45.4	41.2	40.1	42.7	8797500000	1211600000	1449100000

0.2	0.2	0	0	38050000	9350700	10439000
9.8	0	0	0	37353000	13315000	8068500
8.6	12.3	12.3	12.3	24549000	5263500	5669800
5.3	0	0	0	15949000	0	7058700
13.8	0	0	0	215970000	66062000	76618000
52.5	47.5	47	52.6	9,9493E+10	1,6469E+10	2,2115E+10
2.7	0	0	0	52226000	16533000	17263000
15.2	2.8	5.8	4.5	573880000	167440000	141120000
13.5	15.4	15.4	15.4	230160000	31665000	36243000
41.1	45.6	40	45.6	323930000	53955000	74091000
22.4	1	0	0	94653000	33138000	25671000
38.6	35.3	35.3	37.9	972040000	142090000	192810000
0	4.4	4.4	4.4	24165000	0	0
6.9	1.5	1.5	1.5	76900000	17988000	23352000
21	23.7	20.3	23.7	467460000	71712000	89257000
17.5	0	0	0	815450000	246190000	267290000
30.5	30.5	30.5	30.5	1779000000	309110000	393870000
6	0	0	0	47011000	16287000	13388000
13.9	0	0	0	108700000	10879000	10343000
12.4	12.4	12.4	12.4	38592000	6812300	6393500
4.1	0	4.1	4.1	9830500	2197600	1815700
0	40	40	40	219030000	0	0
0	12.9	12.9	12.9	9885600	0	0
55	40.6	40.6	40.6	1055400000	140310000	275290000
11.3	4.4	4.4	4.4	396680000	89874000	115070000
0	7.8	7.8	7.8	42422000	0	0
18.8	10.5	10.5	18.8	39095000	8929100	10287000
40.5	18.9	19.6	19.6	255020000	58405000	72650000
3.3	0	0	0	47283000	12521000	16204000
5.8	3.3	3.3	3.3	67186000	14234000	14449000
0	5.1	0	0	15396000	0	0
26.8	8	8	8	770770000	232830000	193240000
43.3	43.2	41.2	48	1,8511E+10	3686100000	4181800000
19.7	15	15	15	251610000	39289000	78222000
47.2	47.2	44.4	47.2	3085200000	356310000	441560000
5.6	3.6	3.6	3.6	84209000	13683000	20824000
3.9	0	0	0	34579000	10288000	11818000
3.4	1.1	1.1	1.1	60602000	12203000	18136000
10.9	13	14	15.7	504570000	71626000	100320000
24.8	20.5	20.5	20.5	1328200000	288360000	368200000
11	3.5	3.5	3.5	169920000	53094000	62853000
13.2	0	0	0	43629000	12364000	14766000
51.2	0	0	0	186970000	57438000	60732000
15.3	15.3	15.3	15.3	414640000	59018000	77843000
26.6	18.3	18.3	18.3	240450000	41482000	52478000

3.2	0	0	0	28895000	6436000	11268000
0	4	4	4	19083000	0	0
13.2	5.9	5.9	5.9	208610000	55971000	66186000
18.2	0	0	0	133400000	36307000	47474000
8.6	0	0	0	17176000	6680000	4897100
3.9	0	0	0	74808000	33592000	17246000
20	20	20	20	119270000	23015000	26357000
43.5	15.6	15.4	15.6	3719500000	1053300000	1162000000
9	0	0	0	53445000	17460000	15783000
6.1	6.1	0	12.2	66190000	11053000	12789000
7.9	7.9	7.9	7.9	459220000	63227000	71799000
0	0	0	0	11957000	5267400	6689700
37.7	9.3	9.3	9.3	783650000	255160000	235400000
24.4	0	0	0	194090000	61776000	48940000
47.1	0	3.2	5.2	3737000000	1091700000	1247200000
7.2	0	0	0	9865600	2597900	3004700
7.5	1.2	1.2	1.6	380590000	100840000	119580000
0.9	0	0	0	33511000	8911900	11614000
14.3	2.2	3.2	3.2	968640000	261590000	318290000
53.6	34.5	34.5	36	2229200000	448890000	560850000
9.1	1.7	1.7	1.7	336140000	114560000	86077000
12.9	0	0	0	278370000	81106000	100000000
12.4	0	5.3	5.3	48887000	8539600	12275000
17.4	3.2	3.2	3.2	519310000	154870000	144770000
2.8	0	0	0	11816000	2928400	5238300
21.5	27	27	27	1017000000	106310000	143060000
5.1	0	0	0	36415000	11194000	11956000
14.5	3.4	1.5	4.8	274290000	52544000	81527000
30.8	25.6	25.3	24	1654400000	172220000	256510000
0	6.4	6.4	6.4	29904000	0	0
10.5	7	7	7	101950000	14298000	25696000
42.1	40.9	34.5	47.3	2130800000	325970000	340970000
35.7	1.7	0	1.7	1769600000	463670000	629020000
1.9	3.8	3.8	3.8	182960000	22648000	20499000
4.2	0	0	0	8364300	1233300	0
33.8	27.6	25.8	32.5	1156200000	210930000	203860000
30.2	16	21.4	21.4	655890000	140690000	173860000
3.6	0	0	0	86378000	29054000	27893000
11.1	0	0	0	293730000	93762000	93906000
12.4	5.8	10	10	69275000	8339600	13404000
5.8	11.5	11.5	11.5	176320000	22073000	17520000
16.9	8.1	2.7	8.1	75581000	20373000	23279000
10.1	9.2	10.1	10.1	676440000	100360000	119910000
17.7	5.5	5.5	5.5	116760000	33592000	35612000
9.4	12.8	18.3	18.3	853370000	94689000	116510000

13.4	29.1	22.7	19.8	367280000	37433000	54844000
12.3	5.8	8	9	606790000	101550000	125030000
29.2	29.2	29.2	20.8	405460000	84715000	14396000
7.7	1.9	2.5	2.5	453040000	106930000	118070000
7.1	0.5	0.5	0.5	516290000	142530000	188300000
29.4	34.5	34.5	43.3	389890000	43249000	53705000
0	11.6	7.2	18.8	17828000	0	0
0.9	0	0	0	20321000	6050800	7102300
2.5	0	0	0	52083000	8209100	21174000
14.5	1.9	1.9	1.9	330940000	87810000	118280000
6.8	0	0	0	348380000	104630000	100130000
0	28.8	0	3.5	179400000	0	0
24.3	11	14.2	15.6	998340000	227960000	277080000
2.4	3	3	3	80729000	13738000	19366000
16.9	8.8	9.6	11.9	552520000	104340000	122270000
17.6	11.4	13.1	15.5	523560000	85507000	109000000
0	2	0	2	204680000	0	0
2.4	0	0	0	36994000	9973500	13939000
0	1.1	1.1	1.1	23601000	0	0
11.9	0	0	0	67478000	18942000	24162000
2.6	0	0	0	37575000	12708000	13799000
7.3	0	0	0	96078000	28817000	32556000
27.3	12.6	12.6	12.6	922680000	215610000	267010000
8.5	5.7	6.7	5.7	335220000	68921000	91175000
10.1	0	0	0	22032000	4748300	8132100
68.8	58.4	56.2	58.4	2,4403E+10	4512000000	5320200000
26.5	27.3	27.3	27.3	368120000	45440000	65646000
54.7	36.9	34.2	36.9	98534000	36485000	22689000
23.3	23.6	21.5	23.6	332990000	48086000	37553000
37.1	12.7	12.7	12.7	553040000	182680000	123670000
24.9	2.8	2.8	0	587860000	195760000	168810000
12.2	0	0	0	210300000	57492000	69006000
0	11.8	11.8	11.8	16682000	0	0
40.6	34.6	34.2	38.3	4620100000	983820000	1057600000
16.6	11.2	11.2	11.2	255130000	72191000	54914000
37.1	37.1	37.1	37.1	1,8006E+10	2787100000	3482400000
64.6	46.9	44.1	51.2	7403900000	2300000000	2277100000
6.8	0	0	0	119560000	40203000	31394000
21.2	0	0	0	53374000	9251400	23806000
7.4	3.9	3.9	3.9	285470000	77790000	86751000
56.9	6.4	7.9	12.2	2458900000	782320000	757300000
65.9	65.9	65.9	65.9	2655100000	400430000	496710000
37.3	61.6	58	61.6	5681800000	463990000	645260000
31.2	7	7	20.3	132020000	38615000	39644000
0	1.5	1.5	3.3	14460000	0	0

5.5	0	0	0	13277000	4009900	4107100
58.7	18.7	18.3	18.3	1,9518E+10	###########	6359800000
55.7	29.3	28.2	31.1	2,9139E+10	8584800000	9113900000
27.4	14.7	14.1	13.3	3845700000	915410000	1072900000
13.4	0	0	0	37350000	14051000	14728000
38.6	31.5	22.1	31.5	593120000	159920000	152490000
8.2	15.1	0	8.2	25393000	6045700	4108800
10.6	3.3	3.3	3.3	160380000	34259000	37532000
13.3	7.5	8.6	8.6	494740000	113670000	112680000
63.5	53.3	45.3	48.1	1,142E+10	1431300000	1760900000
18.4	20.1	16.5	16.6	4321700000	654310000	819470000
26.1	26.1	27.9	26.1	3978800000	472830000	1013300000
31	30.5	31	31	8415800000	716460000	1629700000
9.7	0	0	0	153260000	48453000	48089000
11.5	4.3	4.3	4.3	100300000	22682000	27574000
10.1	8.4	8.4	8.4	78544000	14147000	16842000
20.9	0	0	0	26490000	8389500	11438000
27.4	13.7	13.7	13.7	864560000	202580000	229410000
21	0	0	0	274020000	91317000	78231000
22.7	15.5	15.5	15.5	320360000	64540000	77845000
18.8	1.2	0.6	0.6	3404300000	992870000	1202100000
12	0	0	0	169600000	53345000	56433000
42.8	27.3	27.6	29.5	5551900000	1549200000	1711000000
11.9	6	5.6	5.6	917980000	197310000	245120000
3.2	3.9	0	3.9	23102000	6101900	5298900
51.5	31.9	27.7	31.9	2123400000	464620000	591610000
4.1	0	0	0	155180000	54183000	70359000
8.7	12.6	6.5	10.4	547400000	59181000	76863000
11.8	2	2	2	90641000	26474000	25127000
0	24	17.3	11.1	51801000	0	0
45.7	20.2	25.1	34.2	612710000	145110000	168770000
56	41	41	32.5	1964800000	490960000	481260000
47	27.3	27.3	27.3	554670000	133920000	131570000
15.9	11.4	9.2	11.4	645140000	168120000	153990000
23.2	23.2	14.3	23.2	2956100000	461450000	566420000
0	10.9	10.9	10.9	17702000	0	0
36.7	38.4	41.1	46	6310700000	1067400000	766850000
51.9	29.4	43	43	3205500000	604580000	763020000
12.3	0	0	0	99508000	31806000	31547000
12.6	4.7	2.3	4.7	5944700000	1302800000	3056500000
0	25	25	0	5114600	0	0
43.3	49.7	56.7	56.7	4372200000	441840000	498640000
54.5	33.9	43	44.8	1142900000	259160000	260480000
0	11.9	11.9	11.9	21977000	0	0
15.2	0	0	0	33809000	9544200	12057000

51.4	0	0	0	###########	952140000	942660000
3.2	15.3	6.5	6.5	205460000	16739000	18625000
0	33.6	47.9	47.9	164170000	0	0
16.1	0	0	0	393110000	114030000	128240000
42.6	0	0	0	2,2557E+10	7413500000	7549400000
23.2	13.8	8.8	13.6	565900000	139930000	160560000
15	4.7	4.7	5.7	585280000	140090000	168330000
23.5	28.3	28.3	28.3	3151800000	265430000	315730000
77.6	69.5	68.2	70.6	4,6153E+10	8209200000	5449000000
16.5	0	0	0	147650000	55357000	37737000
41.7	28.8	29	29	3148100000	631050000	861290000
48.5	36	57.4	57.4	1161100000	209910000	232800000
29.9	24.8	29.2	29.2	1813300000	153550000	305440000
17	26.4	20.8	26.4	808650000	111470000	172040000
38.2	20.6	24.3	26.1	1483600000	298160000	347020000
3.1	1.7	1.7	1.7	76748000	17501000	20703000
25.6	25.1	29.1	29.1	1519300000	286410000	412410000
6.4	0	0	0	41426000	11972000	14048000
9.9	0	0	0	14481000	4288600	4637200
51.6	48	46.4	49.6	8489000000	1602800000	1606800000
10.7	1.3	1.3	1.3	330740000	91962000	101170000
25	9.5	9.5	9.5	378350000	107430000	76338000
14.4	0	0	0	459580000	142350000	143880000
22.9	8.8	9.6	11.3	1240300000	324980000	283420000
33	37.3	37.3	47.9	1557500000	238860000	270840000
20.6	16.2	16.2	16.2	593280000	113440000	154650000
19.4	13.9	13.9	13.9	429480000	106100000	115510000
12.9	0	0	0	84674000	31047000	21341000
21	0	0	0	75308000	23454000	24589000
6.7	0	0	0	33049000	9932900	12396000
2.6	0.4	0	0.4	58773000	16243000	10094000
21.7	0	0	0	282140000	88996000	88292000
1.1	0	0	0	68982000	26303000	5510700
33.7	41.9	41.9	41.9	567810000	76139000	86303000
9.9	13.9	13.9	13.9	199810000	30200000	35973000
1.2	1.2	1.2	1.2	42242000	6141000	7854700
5.9	0.7	0.8	1.4	334170000	83101000	101760000
19.3	12.3	15.4	15.4	801340000	144860000	174270000
10.9	12.5	13	13.8	1396000000	181010000	229540000
64.6	4.7	10.6	10.6	2317000000	633110000	777590000
33.3	0	0	0	6624500	2461200	0
26	0	0	0	254270000	77321000	82243000
11.7	0	0	0	926060000	272140000	326460000
21.7	22.1	22.1	25.4	145850000	18866000	23655000
39.2	47.1	41	47.1	3409300000	299470000	428550000

61.8	23	25.2	33	1,1992E+10	#######################	
7.1	15.4	15.4	15.4	76521000	9956400	11367000
17.4	0	0	0	930000000	274980000	341780000
27.2	0	0	0	448640000	123400000	153590000
1.2	0	0	0	19095000	4650700	6836400
18.9	26.8	26.8	26.8	325920000	45337000	50204000
2.1	0	0	0	9355500	2970300	2450800
24.3	24.3	24.3	24.3	44387000	6846200	8486700
19.6	0	0	0	223110000	74176000	61639000
4.8	6.8	6.8	6.8	211930000	29120000	40396000
31.8	28.6	28.9	31.7	4427100000	566920000	638620000
35.6	34.7	33.9	38.5	1055000000	193170000	129250000
32.7	20	20	33.1	593090000	132410000	145490000
2.5	0	0	0	24287000	7161100	8442000
6.6	7.4	7.4	3.7	120570000	13448000	15142000
0	19.1	9.6	9.6	46214000	0	0
11.5	0	0	0	34929000	11332000	10757000
50.7	56.3	56.3	53.6	41516000	6972600	7075100
28.6	25.2	25.2	25.2	449100000	92797000	98868000
37.2	21.4	11.7	15.9	894380000	208800000	299690000
43.4	12	7.5	8.4	901560000	216120000	288390000
25.5	22.1	19.7	22.1	136480000	33962000	43304000
44.7	41.8	43.8	43.8	1237700000	251170000	305110000
41.1	8.6	8.6	8.6	657610000	140960000	196110000
51.3	13.8	25	19.1	910000000	225810000	279720000
19.6	19.6	19.6	19.6	72063000	14516000	19717000
52.5	43	36.1	36.1	1541300000	292390000	418610000
0	24.4	15.1	24.4	41013000	0	0
10.9	20.2	10.9	20.2	244550000	37428000	52810000
22	31.4	31.4	31.4	527250000	95405000	124450000
23.8	31.7	31.7	23.8	463020000	57280000	73164000
42.1	24.8	30.5	28.9	2819400000	638350000	772670000
32.2	32.2	32.2	32.2	2395900000	341550000	464480000
44.9	23.1	23.1	23.1	703260000	135840000	190850000
22.4	0	0	0	163250000	29648000	64519000
0	0	0	0	18737000	9524400	9212900
19.6	0	19.6	19.6	100290000	18472000	27306000
31.3	31.3	31.3	31.3	1341400000	253020000	342330000
43.8	39.3	35.4	35.4	1295900000	270670000	344280000
39.7	28.8	28.4	28.8	1585100000	354480000	425770000
46.1	37.5	44.5	44.5	193700000	27057000	52968000
27.9	20.8	23.4	23.4	669890000	134360000	131240000
9.6	0	0	0	70182000	23921000	23754000
51.7	50.6	51.7	51.7	888780000	219800000	189750000
11.1	23.5	11.1	11.1	80476000	14423000	12308000

55.2	56.3	56.3	58.1	###########	1505800000	1467400000
32.4	5.2	5.2	5.2	299590000	70431000	104560000
51.9	38.1	38.1	31.8	2,0617E+10	4789400000	4740200000
55.1	34.6	39.5	39.5	1,3786E+10	4248000000	4260600000
65.8	45.4	45.4	48.1	2,2628E+10	6945900000	6428400000
11.3	0	0	0	42520000	12469000	13551000
28	9	9	9	272200000	75643000	64488000
23.9	4.3	4.3	6.8	140800000	39434000	42600000
5.4	0	0	0	30639000	9094700	10526000
32.3	20.5	20.5	23.6	2,5124E+10	6098300000	6596100000
0	9.3	9.3	9.3	21615000	0	0
22.7	22.7	22.7	22.7	807720000	156410000	223300000
0	22.9	22.9	18.1	106760000	0	0
32.5	27.3	28	28	5689000000	677660000	981680000
31.7	45.4	45.4	41.7	4181900000	806890000	578300000
22.3	0	0	0	238690000	64399000	69062000
9.9	2.8	2.8	2.8	1400600000	399240000	471530000
8.9	8.9	3.4	3.4	84664000	13625000	17567000
10.2	8.8	7	7.2	547170000	114750000	152390000
10	6.8	6.8	5.4	261070000	49589000	40304000
25.3	15.4	15.3	17.2	1607900000	298600000	325010000
10.2	0.8	0.8	0.8	1076100000	289250000	361050000
8.5	14.2	14.2	14.2	92001000	8687200	7167700
36.3	20.3	20.3	14	196320000	62406000	54176000
28.1	16.6	14.5	16.6	338150000	62018000	89506000
10.6	4.2	4.8	6.8	158970000	27365000	28281000
57.6	12.6	15.3	15.3	3,8376E+10	1,0065E+10	1,1125E+10
67.8	68.3	66.3	68.3	7258000000	1189300000	1280900000
0	9.6	4	4	1310500000	0	0
6.8	9	5.7	7.5	590650000	67366000	88967000
2.9	3.9	2.9	2.9	98844000	10179000	21030000
34.1	23.5	16.5	22.4	1978400000	422170000	517220000
5.9	1.2	1.2	1.2	305540000	88819000	98983000
9.4	4.3	4.2	5	525050000	132010000	145150000
9.9	0	0	0	721630000	194310000	252490000
2.1	2.1	3.5	3.5	60644000	7807100	9128800
6.2	4	4	4	141640000	33448000	40774000
11.3	0	0	0	343520000	106330000	114260000
30.5	23.6	23.6	23.6	406220000	45687000	55180000
28.9	0	0	0	997900000	315910000	328630000
16.3	21.7	25.1	24.7	956910000	103200000	123200000
11	13.4	13.4	15.1	1670000000	195640000	240700000
43.8	19.8	17.1	19.8	387000000	124420000	125080000
3.5	0	0	0	216120000	29816000	87489000
23.3	11.8	13.4	13.4	495930000	106100000	114090000

20.9	25.9	18.4	20.9	246500000	33872000	40981000
0	4.5	3.6	4.5	98574000	0	0
19	0	0	0	283240000	87701000	102330000
21.6	2.1	4.2	2.1	536240000	144670000	173980000
50.8	30.8	30.8	30.8	49734000	16780000	15686000
11.1	16.8	15.5	13.4	822590000	73438000	100160000
20.5	0	0	0	242190000	63603000	91534000
4.4	7.8	4.9	7.8	113940000	8779500	8333100
9.7	18.2	13.6	18.2	262980000	52680000	59039000
2.1	0	0	0	53731000	15099000	18659000
43.6	35.5	37.7	37.7	8289300000	1953300000	1984500000
1.6	15.2	6	3.3	41571000	0	0
14.9	9.5	8.2	10.9	1400400000	288680000	328000000
0.5	0	0	0	14402000	4406700	5124300
5.9	9.8	9.8	9.8	597610000	46735000	83016000
4.2	0	0	0	32153000	9216000	10450000
27	3.4	3.4	4.1	989190000	262360000	317100000
34.7	40.6	40	41.6	3859800000	443970000	536240000
4.9	3.6	4	4	219600000	19975000	51211000
2	0	0.9	0.9	23403000	3765500	6317200
67.1	0	0	0	3,7375E+11	1,1212E+11	1,3359E+11
35.6	27.8	22.3	30.2	1380000000	217160000	244530000
5.2	0	0	0	70387000	21141000	18991000
13.5	3.8	3.2	5	1108400000	273440000	361900000
67.4	40.5	43.1	45.7	73222000	3461500	30255000
7.7	7.7	4.1	7.7	70278000	12726000	18447000
0.7	0	0	0	10468000	2569200	4400200
1.6	1.6	1.6	1.6	58514000	9141900	11979000
21.2	0	0	0	241260000	70449000	84555000
4.2	0.2	0	0	375930000	106700000	105540000
55.3	52	52	47.5	7170300000	1330700000	1334900000
2.7	3.8	3.8	3.8	39161000	7910300	9715500
49.7	54.9	54.9	54.9	3991500000	438380000	531960000
35.3	15.1	33.2	33.2	1058500000	243520000	314650000
16.8	12.7	16.8	16.8	294490000	43061000	53558000
9.2	3.9	3.9	3.9	274890000	65263000	87279000
6.9	0	0	0	123600000	37147000	39022000
29.5	34.8	34.8	34.8	329580000	34512000	48000000
36.8	0	0	0	4240500000	1319000000	1301700000
5.7	7.9	12	12	369390000	42626000	50699000
0	7.4	0	0	1,341E+10	0	0
24.5	12.8	12.8	12.8	1249000000	435510000	380370000
2.1	8	8	8	49308000	2360400	0
0	7	0	0	168490000	0	0
2.2	0	0	0	14588000	3575000	4839800

4.7	5.2	5.2	7.6	57287000	8273900	9843600
3.4	0	0	0	19069000	6603900	6640000
36.5	5.6	5.6	5.6	287300000	72966000	93277000
16.6	0	0	0	68402000	19729000	21177000
15.5	8.3	8.2	10.5	252160000	64166000	66473000
17.4	10.4	7.6	6.5	430410000	112500000	106210000
31.3	28.2	28.5	31.3	177440000	25891000	28773000
33.3	16.1	17.3	17.3	26172000	9396200	10287000
55.8	58.4	59.5	68.9	1,8575E+10	1021700000	789400000
32.5	19.9	15.6	24.2	1781600000	388320000	477500000
12.2	0	0	0	343080000	100860000	109810000
0	11.8	0	11.8	4207200	0	0
9.8	0	0	0	31125000	10783000	12314000
9.7	0	0	0	187480000	56319000	62853000
14.7	19.5	19.5	25	192840000	30481000	30878000
5.4	0	0	0	64387000	19335000	20130000
2.2	0	0	0	137890000	42942000	47248000
0	9.5	16.2	16.2	166950000	0	0
12.5	0	0	0	517540000	151160000	178680000
24.6	1.3	2.6	4.1	878470000	253290000	241630000
31.6	16.2	16.2	19.4	828310000	145680000	125440000
37.7	8.5	8.5	8.5	1331700000	331050000	434120000
5.3	0	0	0	77380000	23249000	27373000
0	4.2	4.2	4.2	21883000	0	0
5.1	0	0	0	46666000	12462000	18368000
3.6	0	0	0	26390000	7378600	8995300
30.7	9.2	15.2	13.6	555390000	106910000	73036000
3.6	3.6	3.6	3.6	98217000	9873700	6382000
0	0	0	7.9	3503000	0	0
0	2.2	2.2	2.2	25030000	0	0
19.9	19.9	19.9	19.9	1804500000	348040000	426180000
5.5	0.6	1.4	1.4	170230000	50152000	54538000
5.3	0	0	0	123650000	36470000	42383000
28.2	9.2	9.9	9.1	5237500000	1309000000	1587100000
7.1	11.9	13.6	13.6	465290000	26433000	49509000
3.9	0	0	0	191880000	56169000	55374000
2.2	0	0	0	18974000	6138700	5286100
10.9	0	0	0.9	396170000	117250000	122950000
11.3	0	0	0	434100000	134160000	139570000
7	9.2	9.2	9.2	240390000	14105000	17665000
15.7	0	0	0	244590000	47706000	93511000
2.4	0	0	0	112740000	28979000	35733000
4.8	0	0	0	71640000	19929000	26071000
49.1	42.8	40.8	42.8	6412500000	1202600000	1484200000
5.5	0.7	0.7	0.7	338090000	91355000	107850000

43.5	1.3	1.3	1.3	###########	738080000	770100000
7.1	0	0	0	112630000	34829000	40952000
12.4	2.2	4.9	4.9	617510000	187090000	33528000
0	4.7	4.7	4.7	15784000	0	0
13.8	0	0	0	164880000	48297000	54032000
19.7	0	0	0	358120000	135160000	102270000
7.6	0	0	0	177670000	53634000	46388000
7.7	4.3	4.3	4.3	667930000	135680000	130780000
8.5	0	0	0	144250000	41200000	50589000
3.4	0	0	0	98302000	29166000	34090000
19	0	0	0	157260000	38485000	59680000
1	0	0	0	17338000	5683500	5634400
18.2	3.2	3.2	3.2	512080000	118640000	231170000
15.3	15.3	15.3	15.3	195270000	36676000	36687000
6.6	0	0	0	47620000	0	21507000
0	3.7	3.7	3.7	800400000	0	0
48.1	52.6	44	52.6	160730000	36373000	32466000
3	0	0	0	41032000	15843000	17605000
0	16.2	11.4	16.2	71706000	0	0
6	2.8	2.8	2.8	72478000	15074000	14994000
7.1	0	0	0	380310000	120430000	141650000
4.5	0.4	0.4	0.4	281160000	77467000	98623000
2.4	0.6	0	0	67953000	9401100	5412400
18.1	0	0	0	923790000	281610000	304640000
4.8	1.4	1.4	1.4	160350000	32925000	39431000
0.8	1.5	1.5	2.1	69030000	5513200	8707300
0.2	0	0	0	43769000	18877000	22933000
4.3	0	0	0	21291000	5885500	6070800
0	4	1.2	0	42943000	0	0
4.7	0	0	0	16011000	4208000	6038300
5.7	0	0	0	25877000	6847500	9004400
18	8.8	8.8	8.8	223000000	56567000	29833000
14.8	3.1	3.1	3.1	678740000	181170000	211320000
15.4	20.2	24.7	22.3	678370000	52700000	61402000
40.1	25.3	25.3	25.3	627070000	20248000	66618000
33.6	39.7	39.7	39.7	489460000	86983000	55812000
5.4	0	0	0	123140000	35307000	46051000
0	5.2	5.2	5.2	29239000	0	0
24.2	8.1	8.1	8.1	837420000	212210000	244720000
66.1	45.4	45.4	48.1	7993400	2635300	3298800
1.9	0	0	0	58451000	17262000	20560000
36.6	33.3	30.9	36.7	1,3956E+10	2426700000	2355300000
24.4	0	0	0	36661000	8973800	13543000
13	5.3	3.7	5.3	184370000	32556000	45133000
2.4	0	1.7	1.7	32136000	7294700	9036700

16.9	0	0	0	406820000	113230000	137870000
4.5	0	0	0	292230000	78398000	103860000
0	50.9	0	0	21905000	0	0
13.1	3.2	0	3.2	232700000	64150000	68026000
8.4	8.5	6.3	8.5	193710000	36967000	44294000
13.5	7	10.6	10.6	285370000	76544000	101050000
11.7	0	0	0	20193000	6044200	7161500
6.5	0	0	0	97405000	30346000	33073000
39.4	14.4	14.4	14.4	2343900000	675250000	819400000
5.1	2.6	2.6	2.6	33773000	9449700	12375000
3.4	2.8	2.8	2.8	79412000	23587000	32036000
6.4	6.4	6.4	6.4	106440000	10226000	25034000
2.3	10.4	10.4	10.4	68820000	3019100	5598700
2.1	0	0	0	20001000	5273100	8232600
5	1.2	1.2	1.2	97599000	25481000	27909000
4.1	7.6	7.6	7.6	32272000	5395300	5819200
15	13	13	15.4	453260000	55259000	61675000
5.7	0	0	0	87183000	21602000	34956000
16.2	13.8	12.3	13.8	679220000	45470000	54555000
27.4	0	0	0	2,0738E+10	6306500000	6866500000
22.8	31.1	24.9	31.6	629060000	117010000	92341000
1.8	0	0	0	29499000	7339600	12104000
0	0.8	0.8	0.8	5763800	0	0
17.1	0	0	0	1125800000	338080000	368620000
11.5	0	0	0	416310000	119370000	141810000
7.4	10.6	8.7	10.6	148280000	16853000	23606000
33.1	36.8	36.8	34	948640000	120220000	159990000
0.3	0.3	0	0.3	742510000	212120000	189880000
2.3	0	0	0	25125000	8647500	6793000
4.3	1.5	1.5	2.6	68553000	15080000	18817000
19.1	0	0	0	159750000	47891000	50503000
18.2	0	0	0	143310000	39668000	48680000
16.5	16.2	17.4	18.9	1974200000	295740000	349750000
13.2	6.4	6.4	8.4	193380000	42231000	53927000
42.8	6.2	7.4	6.8	1,0921E+10	3166500000	3674900000
1.9	4	4	4	40626000	4222000	3976800
17.8	17.8	17.8	17.8	406900000	22561000	75960000
15.8	9.1	10.9	11.4	1329300000	271090000	298990000
28.3	4.9	4.9	4.9	793530000	230910000	222380000
3.4	4.4	5.6	4.1	235460000	31413000	34437000
50.8	52.5	52.5	53.9	1,4378E+10	1658500000	1982900000
2.5	0	0	0	57640000	16258000	17578000
7.4	7.4	7.4	7.4	56159000	10826000	12591000
5.2	0	0	0	16337000	4928200	4614000
2.1	2.1	2.1	2.1	28513000	5673900	5895300

8.7	0	0	0	35990000	11196000	10789000
6.1	0	0	0	66777000	19796000	21639000
10.8	0	0	0	58321000	17896000	21114000
12.8	16	15.8	18.8	787700000	92860000	92042000
0	33	21.4	33	102350000	0	0
18	11.6	14	14	213210000	54575000	43372000
28.8	36.1	34.5	33.9	1350800000	156630000	177330000
8	10	10	10	362370000	31974000	37630000
6.9	0	0	0	218810000	61280000	81740000
37.4	0	0	0	399560000	114600000	137090000
1.5	0	0	0	41147000	10627000	17002000
3.8	3.8	3.8	3.8	43758000	7316100	8244100
8.7	5.5	3.6	5.5	207540000	54741000	41051000
11	0	0	0	58047000	19162000	14731000
6.4	1.7	1.7	3.9	84944000	17740000	24270000
7	0	0	0	398130000	127180000	127030000
9.5	0	0	0	42960000	12021000	14249000
34.4	23.5	22.7	24.8	5275900000	1185400000	1464100000
1.2	4.7	4.7	4.7	13302000	0	0
8.8	0	0	0	26989000	8877400	7855100
14.9	0.8	0.8	0.8	975320000	300560000	363030000
8.9	0	0	0	131620000	37957000	46467000
12.9	0	2	2	171890000	45267000	55894000
7.4	1	1	1	246540000	56503000	53004000
6.4	0	0	0	497980000	133010000	168690000
64	9.2	9.2	12.5	4,6004E+10	1,5578E+10	1,6604E+10
7.3	0	0	0	124310000	35023000	42254000
10	6.1	6.1	6.1	16788000	4519500	5216800
14.8	43.2	43.2	43.2	581660000	53984000	72642000
7.7	11.4	14.8	14.8	112740000	17318000	21175000
26.9	19.9	21.2	19.5	4411700000	871750000	1124800000
16.6	8.5	14.2	12.3	580210000	110580000	107660000
51.7	50.7	53.1	62.6	2021500000	218090000	248150000
24	34.1	34.1	34.1	1166700000	157970000	110300000
17.7	3	4.1	5	1327800000	327110000	409630000
11	11	0	11	58446000	7932400	10579000
36.1	39.5	39.5	39.5	1192800000	143440000	187550000
43.7	10.8	10.8	10.8	447060000	137400000	145770000
19	8.9	8.9	8.9	1100200000	361780000	367630000
18	0	0	0	1308400000	56813000	507520000
10	0	0	0	102520000	31952000	36815000
21.2	21.7	21.7	21.7	455230000	79658000	86411000
55.8	15.1	15.1	15.1	104780000	21127000	28362000
17.9	4.4	11.1	11.1	151590000	30712000	42450000
21.6	25.2	25.2	25.2	29454000	0	0

14.5			0		0		0	485190000	135540000	166570000
	15	27.2		27.2		27.2		155420000	12183000	15472000
6.9			0		0		0	75979000	23248000	24721000
21.7			0		0		0	114120000	33824000	40916000
7.5		2.3		2.3		2.3		44786000	6109900	14083000
12.4			0		0		0	1263300000	357210000	453380000
12.5			0		0		0	353220000	98665000	118080000
38.4		11.1		11.1		11.1		145880000	44102000	38029000
6.4		8.1			11	9.4		215890000	20347000	39140000
12.9		2.4		1.6		2.4		314470000	69471000	78156000
2.8		3.6		3.6		3.6		60306000	2461000	0
22.5		5.5		5.5		5.5		284590000	91653000	92269000
0.9			0		0		0	8736900	2114900	3277800
72.1		72.1		72.1		72.1		634500000	96646000	111470000
20.9		5.7		5.7		9.8		578370000	153620000	197830000
4.1			0		0		0	71655000	24558000	25829000
2.6		1.9			0	1.9		44093000	9723700	15932000
39.3			0		0	0.9		4907800000	1457200000	1605000000
10.8		11.7		11.7		5.4		72136000	13607000	17956000
5.6		5.6		3.3		5.6		156450000	16538000	29814000
10.8		23.1		21.9		21.9		2451600000	134410000	128130000
21.4		11.4		21.4			17	351560000	54728000	69129000
4.6			0		0		0	7678300	2514000	2210700
4.1			0		0		0	22742000	6974200	7729500
23.5			0		0		0	473540000	143640000	155350000
43.8		2.3		2.3		2.3		5890200000	1626300000	1715800000
24.1		27.5		23.8		27.5		479910000	59618000	57165000
3.2		5.7		8.1		5.7		42777000	3625100	4284600
60.4		22.6		17.7		22.6		4685300000	1325700000	1574800000
2.3			0		0		0	37058000	10061000	12250000
	14		0		0		0	184610000	48173000	60671000
3.9			0		0		0	14274000	6001000	8273200
10.7		15.5			11	15.5		100130000	17480000	20560000
	20	11.8		11.8		16.7		238720000	32886000	47795000
1.1			0		0		0	16870000	4888100	5652200
11.3		11.3			0	11.3		167870000	23764000	34027000
2.4			0		0		0	16837000	3863800	5297400
47.4		46.4		49.7		49.7		8156700000	1101200000	1168900000
7.6			0		0		0	549700000	210990000	98217000
1.1			0		0		0	11735000	3439200	3871700
10.4			0		0		0	53304000	14754000	17846000
7.2			0		0		0	935470000	281440000	279950000
12.3		22.3		22.6		26.7		460940000	23629000	43260000
13.7		6.4		6.4		6.4		142130000	9359900	35903000
3.6			7		7		10	96877000	9259000	8534000

7.2	0	0	0	84955000	20754000	29474000
7.4	4.8	4.8	4.8	360430000	46531000	67286000
3.6	3.6	0	3.6	17933000	4616500	0
0	11.9	11.9	9.1	99035000	0	0
10.9	0	0	0	71645000	18448000	26446000
3.6	0	0	0	23456000	7050500	8861700
12.7	0	0	0	128680000	37365000	39983000
12.6	0	0	0	188520000	47377000	64128000
22.4	15.5	17.5	18.7	2973000000	514000000	649680000
9.4	0	0	0	23511000	7971700	11282000
3.1	1.6	1.6	1.6	85396000	19818000	25123000
15.8	25.3	25.3	25.3	133150000	6903300	6193500
25.8	0	1.6	1.6	727220000	208760000	245180000
6.5	5.3	6.5	4.4	192020000	35981000	40740000
7.6	0	0	0	56191000	16469000	17680000
4.7	0	0	0	40815000	12671000	18809000
12	12.3	12.3	12.3	453690000	87350000	86418000
3.1	0	0	0	60183000	16594000	20882000
13.8	15.6	15.6	15.6	345620000	61371000	62856000
0	7	0	0	4630600	0	0
2	0	0	0	34414000	9441300	12396000
0	2.9	2.9	2.9	21500000	0	0
11.7	2.7	2.7	2.7	504550000	132400000	161670000
13.8	0	0.7	0.7	956580000	273390000	303600000
5.7	0	0	0	214290000	60506000	70819000
7.9	6.8	6.8	4.6	212440000	56219000	46949000
8.1	0	0	0	240960000	68236000	79297000
23.8	0	0	0	1058900000	330160000	356860000
45.6	34.5	27.2	34.5	1856800000	385700000	430910000
15	12.7	10.3	14.2	4032000000	519850000	624920000
48.8	22.5	28.3	30.7	2423100000	664830000	682160000
2.7	0	0	0	0	0	0
5.3	0	0	0	216460000	73517000	63118000
6.5	0	0	0	78820000	25396000	24191000
30.6	28.7	29	39.7	1932600000	301790000	282630000
39.7	29.3	26.1	33.4	832550000	164500000	179560000
22.3	15.3	17.3	18.7	3833900000	522680000	798750000
41.8	28.9	28.9	28.9	3008200000	770130000	907940000
38.7	0	0	0	4256600000	1302900000	1321000000
38.3	31.9	34.7	37.5	4508200000	739690000	865340000
7.9	0	0	0	16895000	4980300	5529200
24.3	28.2	33.2	36.1	888670000	48566000	59252000
20	11.2	11.2	11.2	65518000	15076000	15238000
6.7	2.2	5	5	60669000	9839100	13604000
21.9	20	20	20	359160000	76205000	98034000

10.3	17.5	17.5	17.5	200040000	22820000	33102000
9.4	13.3	14.3	15.6	440440000	50281000	60180000
16.1	0	0	0	777390000	231280000	246440000
4.2	1.5	1.5	1.5	714460000	90832000	117470000
30.8	9.5	9.5	9.5	1455400000	437870000	447990000
19.7	11.5	12.5	12.1	1782600000	396130000	510250000
9.4	6.2	6.2	2.3	244440000	63306000	72621000
5.8	3.2	3.2	3.2	165380000	23657000	47126000
6.2	6.2	0	0	24460000	0	0
0	0	0	0	1223000000	94642000	228480000
0	0	0	0	33688000	0	0
0	0	0	0	132190000	32310000	68045000
0	0	0	0	93692000	0	0
0	0	0	0	83562000	11502000	10580000
0	0	0	0	158920000	1550200	3188000
0	0	0	0	711030000	0	0
0	0	0	0	7807200	0	0
0	0	0	0	1453800000	195840000	230090000
0	0	0	0	133280000	0	0
0	0	0	0	266660000	46017000	103250000
0	0	0	0	11116000	11116000	0
0	0	0	0	1,0221E+10	0	0
0	0	0	0	52944000	37661000	0
0	0	0	0	4,5155E+10	6548600000	7495600000
0	0	0	0	3755900000	714060000	883490000
0	0	0	0	566190000	91372000	120240000
67.1	34.2	34.2	34.2	65988000	10186000	9725600
10.9	12.5	12.5	14	896620000	115110000	119660000
34.3	26.2	30.2	30.2	491090000	91549000	90765000

Intensity CBX	Intensity WT	Intensity WT	Intensity WT	iBAQ	iBAQ CBX2_1	iBAQ CBX2_2
50502000	10076000	12655000	24213000	30320000	6332800	7746600
34235000	13820000	8340200	24130000	18104000	3786000	4252400
41748000	3887300	1017000	2227300	8207900	852800	1245200
53554000	223460000	172920000	264150000	25800000	1406600	1358600
67830000	54593000	26432000	50689000	12438000	1982600	2140700
11453000	3054400	3892300	7947300	2103900	381760	467580
4766700	0	0	0	1294700	338040	523320
751950000	178260000	91505000	253760000	372540000	62752000	97208000
59616000	60698000	40509000	69291000	18819000	2198100	3085000
10121000	0	0	0	3002900	1083300	795050
15257000	19130000	24245000	36007000	5110100	559420	607440
10543000	14307000	11658000	16835000	4958000	668920	732870
132630000	48397000	25790000	55883000	24394000	3655000	3225600
3709800	2921000	1386300	2387300	387540	66224	67546
37473000	0	0	0	7371700	2374400	2499100
3070100	0	0	0	697730	461580	0
139950000	51316000	44452000	105050000	26508000	6513200	3767300
0	0	0	0	3382300	3382300	0
378320000	30506000	31643000	48450000	18892000	5083000	6048600
74468000	16440000	8262000	10701000	5300700	1108800	1512100
1001200000	377760000	572560000	650540000	690500000	149250000	169520000
14712000	5990700	3877200	13486000	12561000	2006100	2941400
445670000	442270000	353310000	559090000	47532000	6749300	7442700
25518000	0	0	0	3664100	1086600	1159900
228320000	0	0	0	21881000	6566500	7440900
13988000	4772200	3275500	3817700	5889100	1196100	1461300
73407000	10680000	5869400	9820800	7652800	1853700	2235600
6940900	0	0	0	3057300	851620	1214100
49866000	3939900	7761100	12778000	6284600	1330900	2700800
98650000	0	0	0	11846000	3382100	4174600
358310000	3389400	0	0	67736000	23141000	18760000
0	0	0	0	522060	238930	283130
64483000	0	0	0	3506900	1610300	0
14292000	9662400	9703300	11596000	3735100	597860	623200
31006000	17279000	13748000	19698000	2845900	317960	627240
12705000	0	0	0	3168100	1103100	1271000
76706000	28349000	26438000	51340000	17452000	3890300	3404600
26554000	0	0	0	3118600	725000	1239100
11287000	0	0	0	7846400	2436100	2588400
90941000	23858000	13098000	35619000	10319000	2788800	2574700
10867000	0	0	0	849000	244630	302520
181090000	0	0	0	7582700	2263400	2533400
6328500	9063900	0	14466000	1830600	242000	290420
17584000	87014000	62548000	79268000	18943000	1696900	2751600

196360000	9593800	9983100	19186000	13108000	3479200	4285000
17491000	15159000	13298000	24371000	2750900	311520	371210
8522800	15997000	9979100	21372000	2473200	258140	288530
19858000	0	0	0	532970	150540	203540
71197000	0	0	0	9614800	3006100	3513200
17591000	0	0	0	2740000	793040	969650
5727400	0	0	0	846810	230950	257900
7063200	19726000	9224300	14361000	1522000	144010	149350
59659000	43698000	13402000	30753000	8554200	1334500	2133000
63863000	0	0	0	5164900	1484600	1684600
137200000	39416000	35211000	30086000	55914000	13847000	15188000
30401000	2839600	0	3448300	3397100	1429300	556730
137790000	40691000	27496000	47103000	25797000	6195800	6280800
5682200	0	0	0	2922300	730580	771190
0	984130000	0	4611100	36620000	0	0
12227000	0	0	2111300	532330	137030	152280
26157000	91041000	91805000	117160000	13102000	745200	277130
51468000	33282000	13488000	22431000	29863000	6458000	8321100
8985900	0	0	0	4922600	1890200	1235200
963560000	233170000	151530000	251220000	426070000	83629000	113950000
2252600000	0	0	0	458550000	147890000	137380000
23584000	17028000	6440300	18271000	3062300	530590	1111600
152140000	137510000	0	140800000	33366000	5558700	7309800
8963800	0	0	0	2077000	876820	303780
54326000	0	0	0	18277000	5202900	6283800
2254100000	2093900000	1319300000	2516400000	494830000	67833000	71188000
28172000	0	0	0	2978900	855380	1080100
81069000	40322000	2552100000	50645000	47373000	981670	989790
229440000	41943000	36454000	58161000	68492000	16484000	11342000
8060600	0	0	0	2882700	724140	815130
7331400	0	0	0	1249100	366480	533490
20617000	0	0	0	2081900	775770	324410
105740000	0	0	0	5237000	1707800	1974100
284100000	65977000	59497000	82721000	140260000	28068000	41867000
32731000	3413200	1822400	3360100	3630800	1028900	1224300
16991000	0	0	0	12432000	3642700	4541600
68545000	30473000	13683000	21460000	18513000	4059200	4133800
39452000	51098000	30028000	55073000	13653000	1746000	2148200
14965000	0	0	0	1009000	308290	335750
450740000	159920000	98888000	131160000	137190000	28558000	32202000
16526000	0	0	0	1850500	552840	546450
57846000	3625500	3063000	5257700	5000500	1377400	1629100
94394000	10912000	9274700	0	8806600	1931900	2467800
11142000	0	0	0	1213100	297150	556540
149830000	5965900	0	7340000	94057000	21724000	31548000

0	11151000	8851400	15773000	4471900	0	0
80767000	34347000	20672000	59747000	26334000	3790200	7502900
36436000	15043000	3030600	28851000	17450000	3111100	3919000
21095000	7846000	21228000	42272000	26114000	3133500	4492000
74269000	0	0	0	1203100	287400	465620
10374000	9608800	7419800	11037000	3102700	451390	515790
367080000	414760000	241340000	380300000	38529000	5014200	5995400
7733200	0	0	0	586720	235210	0
27935000	51278000	33463000	30826000	6772400	718570	928780
74837000	0	0	0	19136000	5357200	6976000
5504400	0	0	0	2182700	913260	810770
304300000	21189000	10464000	17095000	29116000	8958800	9124500
11201000	20261000	12920000	18562000	45177000	10760000	13435000
16842000	52728000	46605000	59034000	20924000	1744600	1658800
23204000	35821000	28346000	40068000	5816700	634450	787760
538010000	0	0	0	18018000	5799700	5231200
11805000	0	0	0	884330	237660	277760
278990000	378290000	143840000	491340000	44092000	3057800	4106900
203190000	80724000	66251000	97722000	49349000	10976000	10381000
0	10878000	1966000	2126500	1497000	0	0
45321000	0	0	0	5775700	2082500	1175400
106430000	14675000	3175300	6989000	25324000	6147500	7243000
374690000	68280000	66949000	133810000	120870000	26775000	29722000
9310300	10397000	5376700	6603500	4675600	1306500	488360
53219000	0	0	0	4580700	1168400	1638400
15293000	0	0	0	1680000	540430	593400
30439000	12811000	17013000	21440000	5717200	1025200	1287600
291940000	70351000	49249000	93559000	37998000	10668000	10494000
265780000	71782000	59636000	89951000	20502000	4269800	5406800
592000000	348890000	294660000	461160000	133900000	22114000	26946000
33516000	0	0	0	4871500	1284700	1822800
6284700	6759100	5825700	5296900	2306200	594520	613230
23881000	0	0	0	1507100	426280	257320
5253400	0	0	0	1672000	524640	490670
6100700	0	0	0	582680	164990	252800
30403000	0	0	0	7872100	2634300	2704200
7317600	0	0	0	4796600	2357400	0
5045000	4778200	2854300	4633600	2695900	353210	419280
1,3393E+10	1967400000	1689900000	2552800000	359180000	90944000	100690000
0	6142500	4615100	7901500	1166200	0	0
74364000	0	0	0	7724800	2409500	2751100
21105000	10952000	8271600	13171000	44197000	7956900	9490300
7383900	0	0	0	4772400	1541300	1385100
866760000	428020000	282820000	387130000	143400000	29553000	28429000
0	7081700	5390300	0	1221400	308430	289410

33786000	19036000	5805700	20955000	50284000	18433000	5323600
258880000	33084000	29701000	33127000	14917000	3251200	4425600
674310000	394080000	304720000	449640000	567450000	93519000	109380000
16104000	17916000	29428000	63016000	21718000	1371300	4538500
15705000	0	0	0	1240700	350870	413900
10956000	7928400	6901800	8857700	3693100	542010	676490
12038000	0	0	0	1418700	533700	403470
323890000	0	0	0	43325000	13144000	15458000
51016000	0	0	0	46423000	12962000	16456000
13434000	26407000	12808000	21860000	13168000	1096300	1427200
104790000	0	0	0	36202000	10117000	12986000
83136000	0	0	0	4599800	876830	1041200
18926000	5363400	3754700	4287000	1616300	351860	390630
38312000	0	0	0	8291200	2606700	3290000
9820500	27078000	20351000	26522000	4458500	322380	328270
7483800	0	0	0	2562100	809460	683530
329970000	473370000	218480000	337740000	48697000	4537600	7414100
15363000	3644400	0	3524300	2184100	600900	644380
257210000	166190000	100220000	194340000	15237000	2576000	3214500
850310000	37148000	47283000	55669000	53637000	13968000	18138000
36472000	4870400	7302100	8011900	4534600	1007600	1260700
7541300	4731700	0	5513600	1433600	327350	297750
58310000	13057000	17030000	22713000	4686900	819980	1089100
2832200000	1004600000	686720000	1347600000	692120000	149710000	150990000
4749800	0	0	0	1183900	244300	464650
34812000	0	0	0	2130500	738260	635430
223580000	0	0	0	4401300	1117100	1577500
11058000	0	0	6123100	8794900	2351600	2148000
407720000	105550000	39440000	107150000	110020000	23909000	31127000
7939000	3361000	1714800	3391100	7493000	1564600	1826900
14036000	0	0	0	310890	83208	109730
13092000	0	0	0	2234000	631920	783880
136160000	19426000	16705000	22147000	78742000	17383000	22472000
62815000	0	0	0	4480800	1261300	1521700
0	12564000	7506300	8861400	346220	67278	73753
18086000	6894500	0	5532900	6700700	1313300	1573200
7672000	0	0	0	852450	0	0
112860000	20585000	5703900	20785000	11554000	2941600	3614600
95480000	0	0	0	5274200	1552500	1773200
38295000	7634400	7815800	11932000	2973000	595730	813480
16691000	5065800	7202200	11578000	6227500	668740	1505100
17377000	7414700	5608200	8944500	3995100	0	968640
325520000	55435000	52988000	110840000	346460000	78191000	86668000
0	84978000	25096000	20797000	65435000	0	0
386810000	320530000	225160000	357840000	57972000	8304900	10567000

445150000	512990000	454400000	590880000	384830000	43007000	55616000
46456000	129400000	99775000	134820000	7713700	661030	833810
208180000	152230000	89816000	160980000	41340000	6924900	7840800
16140000	0	0	0	4463100	1372600	1476400
0	47324000	0	0	1051600	0	0
67859000	0	0	0	7500300	2272400	2618000
210230000	39388000	14243000	28897000	23823000	5915300	6647900
4998100000	2603000000	2241000000	3507000000	3727400000	720230000	782350000
50358000	4583200	3079900	4192600	30972000	8501200	10028000
29737000	0	0	0	1163000	360500	371490
83899000	9189400	5247000	8696600	9396100	2465500	2814100
12789000	0	0	0	2334300	698440	836520
98944000	0	0	0	18452000	6010700	5845300
3281000	0	0	0	2212000	795240	596460
234780000	1908100	2065800	3627500	6540200	1884400	2077300
4217200	0	0	0	1171400	343210	359610
173090000	2597300	5115500	7384300	16341000	4703800	5364700
34073000	0	0	0	16502000	5479100	5343900
15478000	0	0	0	1496800	436860	486690
484150000	55625000	45116000	56280000	28468000	6941200	9869400
96295000	3791900	0	0	10930000	3023500	3735800
1084400000	1347800000	595690000	739460000	221840000	13392000	20074000
13405000	0	0	0	637250	195050	218790
1972500000	2108900000	1232500000	2608200000	420490000	56530000	70552000
27303000	0	0	0	3427900	1154800	1086100
187460000	126150000	81329000	142880000	23476000	3573500	4083700
0	7421700	5178200	9145700	1279200	0	0
1441900000	530040000	350300000	705140000	1114000000	341270000	167220000
103480000	201690000	181760000	291170000	47284000	4663400	3716000
226650000	17281000	27829000	41649000	28672000	6887000	9248200
875450000	1271900000	497910000	544230000	221300000	13294000	20388000
76247000	61053000	56677000	99595000	28062000	3901300	4589500
56686000	24699000	20090000	28752000	58592000	14719000	17828000
264830000	122320000	100620000	150690000	21706000	4617100	5042000
0	3419600	2506000	6324900	1361200	0	0
10423000	6767400	4873500	8059500	12208000	2161800	2515900
13272000	11026000	7947400	8925600	5124700	976860	980880
6045300000	647770000	547120000	754960000	1788500000	525180000	536450000
15101000	18506000	17978000	20511000	1874600	200860	287280
73107000	56797000	37073000	54090000	14532000	1886700	2597100
677910000	1742600000	105870000	123140000	215160000	43770000	24192000
0	4091400	2921100	2892600	521320	0	0
44260000	40280000	12166000	33720000	10457000	1551300	2041000
17309000	6822200	4157200	4969300	2264300	489540	586970
536530000	155240000	62172000	112070000	132110000	27330000	38161000

275070000	144350000	78375000	168330000	71510000	14003000	15874000
0	5667400	5390600	0	5724800	1833200	2509300
8214400	867110	2429700	2867000	2515100	362140	715100
72968000	0	0	6207900	15468000	4716300	4660900
121970000	0	0	0	2963700	839450	984360
58707000	8923900	3545200	0	8287800	2556900	2341600
36026000	51214000	42144000	53572000	15469000	1855100	2178800
6314900	0	4347700	6120300	1716600	341730	325920
20646000	0	0	0	2876900	805520	984790
0	20827000	15798000	29079000	1398000	0	0
114350000	7199900	0	6926200	10672000	3530600	3127000
9659000	0	0	0	374110	94281	116120
0	26949000	11765000	21611000	12065000	0	0
29583000	0	0	0	3722900	1098900	1215200
10075000	0	0	0	3378200	306920	2698100
99307000	0	0	0	10451000	2561800	3751500
33519000	20819000	14133000	16315000	16143000	2894100	3828200
70231000	52357000	18021000	38091000	15251000	2577900	3267300
8728700	0	0	0	1780100	533970	622610
1906500000	5062000	889530	6738600	85014000	22490000	26984000
78534000	0	0	0	6660700	1989100	2217400
118250000	10068000	8297000	10615000	9398100	2312000	2996400
14711000	18868000	8491800	17266000	9889500	0	0
10514000	0	0	0	4214300	1455000	1445100
22320000	0	0	0	6972200	1711200	2470900
7003500	0	0	0	3518500	1026100	1325100
6673400	8018700	10577000	14335000	2625500	263350	277760
12709000	0	0	0	3403500	1244400	1003800
59471000	0	0	0	1093000	296560	338920
6012200	0	0	0	379220	100530	128380
7028100	0	0	0	1238900	459250	366250
14805000	0	0	0	4684100	1324800	2013300
0	43026000	39045000	55056000	5078800	0	0
482070000	279810000	99483000	213440000	127400000	23993000	26633000
22059000	18607000	0	16535000	6773900	1027500	1346300
19820000	34721000	9115400	34613000	21272000	1904500	2989300
11962000	17049000	7930000	14293000	5541200	424360	459150
27676000	0	0	0	3419600	1245000	856640
13920000	0	0	0	1577100	512810	484320
10345000	0	0	0	1494400	413520	391200
196140000	17436000	13346000	20909000	33078000	8646000	10664000
0	47102000	12932000	87757000	29558000	0	0
231180000	0	0	0	10133000	2842700	3620500
7359600	0	0	0	911760	309320	308060
163010000	196580000	172680000	185040000	89613000	10601000	13800000

22689000	0	0	0	1709000	497520	563250

K4;R9RIA8;R9RI97;R9RFL1;R7RVB7;R7RV73;R4RWV9;R4RUH0;R4NLT9;R4NJ42;R4L6P2;R4L0K0;R4KXX0;Q9TQP8;Q9MW39;
;F8R183;F8R181;F8R177;F8R176;F8R174;F8R173;F8R172;F8R171;F8R170;F8R165;F8R163;F8R162;F8R156;F8R155;F8R154;
9UR01;R9RVK4;R9RIA8;R9RI97;R9RFL1;R7RVB7;R7RV73;R4RWV9;R4RUH0;R4NLT9;R4NJ42;R4L6P2;R4L0K0;R4KXX0;Q9TQ
;F8R183;F8R181;F8R177;F8R176;F8R174;F8R173;F8R172;F8R171;F8R170;F8R165;F8R163;F8R162;F8R156;F8R155;F8R154;
273;273;273;273;273;273;273;273;273;273;273;273;273;273;273;273;273;273;273;273;273;273;273;273;273;273;273;273

76537000	77738000	27786000	39003000	20542000	3477000	4061500
145060000	0	0	0	6127100	1803400	2158700
0	0	0	0	524180	524180	0
11876000	0	0	0	334300	68258	61277
9120300	0	0	0	7296500	1995600	2260800
5899200	0	0	0	60538	19709	19141
24951000	12040000	8881300	31820000	3937700	519270	643700
44063000	0	0	0	9491400	2062800	3422900
0	11993000	0	0	2398500	0	0
95502000	0	23717000	32226000	16645000	4995700	0
345090000	226960000	186560000	269170000	39929000	6037000	8197300
10454000	0	0	0	9356200	3059700	2811900
9446400	2806200	1378400	3269800	4958000	1317900	1762200
9612500	0	0	0	464990	142510	167440
78777000	3545000	2500600	4156100	31339000	8228700	10399000
5520400	0	0	0	1048400	317990	362400
101930000	0	0	0	31736000	10034000	11510000
98083000	0	0	0	33318000	9428600	7542400
55509000	10228000	12439000	8766900	16090000	3725800	5118500
8579400000	5811000000	4485800000	6398300000	217560000	33181000	43180000
14486000	131160000	58549000	92252000	9835800	220540	351370
3978800	0	0	0	1665400	472450	529770
62159000	40266000	47734000	77026000	8822400	1100500	1411200
42338000	0	0	0	4752400	1380500	1607800
22925000	0	0	0	1645800	498870	543660
16713000	0	0	0	238690	61368	90280
27818000	0	0	0	1918000	512160	673800
6454000	0	0	0	259120	52143	99411
3240400	0	0	0	845140	283080	238020
133010000	161980000	61703000	218820000	210140000	38791000	27468000
42581000	0	0	0	1138100	339510	355030
0	110770000	79672000	131890000	14015000	0	0
9324800	17486000	11236000	17195000	1305400	114790	148300
5559500	0	0	0	909580	236030	302920
12483000	0	0	0	3959500	1295100	1277400
7433100	0	0	0	699140	392180	148810
11363000	4768600	3395300	4668400	2108500	362080	472920
8301900	0	0	4991500	3426500	881240	883590

40600000	32226000	22338000	37277000	28441000	4245300	5275300
8273900	0	0	0	805180	74658	445220
3317000	0	0	0	890070	285760	302760
0	2197200	1816900	3787200	600100	0	0
215580000	0	0	0	31734000	8667300	11720000
22369000	18842000	16112000	18693000	19092000	2437800	3984600
0	6010800	0	0	667870	0	0
5240200	0	0	0	582890	166920	206360
0	0	0	0	10368000	4944100	5424100
112090000	25553000	16970000	25514000	31619000	7634600	5972300
55881000	64163000	22558000	33313000	18174000	1978200	2663900
279120000	77407000	45822000	104510000	21506000	4415800	5570600
0	3816400	2911000	3503300	682040	0	0
34255000	32407000	32199000	51315000	4364500	525960	573880
37525000	11897000	7615400	13049000	14961000	3518200	3655000
15796000	0	0	0	4045000	1162900	1302400
5019800	0	0	0	1458300	362420	593940
4882000	7953500	4409300	6775800	1087500	122010	190670
57177000	23525000	27831000	45834000	25688000	3120100	5415400
0	796610000	2301900	2302900	29675000	0	0
35794000	6824300	4177800	4730700	3745500	886690	1196700
211180000	127330000	116870000	139630000	71746000	10816000	15160000
10217000	5369600	4540200	4681000	2012300	383310	447620
24862000	10665000	0	0	9866100	2692100	3944200
76688000	0	0	0	5785500	1616500	2202700
9808800	0	0	0	1601900	555030	292320
10405000	0	0	0	4822500	1249500	1838900
822660000	890280000	520060000	557910000	116750000	13074000	19106000
59411000	0	0	0	3354600	822880	1008300
33986000	0	0	0	1657600	469230	559030
20743000	13941000	5103500	17178000	14073000	1813600	2765600
8713200	0	0	0	1212000	345360	382570
27433000	0	0	0	785720	243240	253720
10405000	38351000	34632000	46612000	7634900	332530	460270
4627300	2888200	1337100	2439700	1234300	222620	347420
10161000	0	0	0	1460100	491790	460210
146080000	45279000	35506000	55806000	31617000	7878600	8034300
1,409E+10	0	0	0	1669000000	487190000	618170000
0	4086600	2263200	4462900	1081300	0	0
12894000	26151000	19619000	27719000	2645700	261150	224970
24421000	0	0	0	2828300	866370	944420
2782700	0	0	0	89244	27982	28524
9900500	0	0	0	3184100	996090	1087900
26124000	8778600	8230100	13330000	7822200	1674900	1804000
4484600	0	0	0	397380	108860	133880

844670000	0	0	0	87237000	22914000	31836000
17193000	54176000	22341000	27207000	6719700	166360	795450
12337000	0	0	0	1191200	353760	343910
381700000	380570000	338600000	550170000	282940000	35961000	40602000
32099000	0	0	0	17219000	3905400	5288500
0	12533000	10836000	6441600	3726400	0	0
25848000	0	0	0	1382100	397090	506370
10320000	0	0	0	1163400	390630	359940
142610000	15471000	15838000	22410000	16145000	4525200	4608300
9445000	8252600	5915400	8745500	2114700	338020	428380
164010000	122600000	92678000	131530000	96149000	14618000	17678000
132800000	190150000	123770000	207460000	16778000	1986700	2447800
25721000	66067000	51335000	82891000	4973500	378510	409490
629110000	121680000	98255000	141530000	22881000	5363200	6387900
28036000	22417000	13460000	33312000	18078000	2918000	3006700
39691000	0	0	0	2857500	806170	1083200
65126000	115680000	70959000	135330000	26389000	2451300	2432300
7268400	0	0	0	503570	150820	187560
1687800000	1451900000	1130500000	1669800000	220930000	34449000	34170000
90933000	0	0	0	11750000	3727800	3475800
85539000	52507000	53914000	65788000	19507000	2695100	4538300
22844000	0	0	0	815960	133790	206250
20944000	0	0	0	2311800	712960	646880
57067000	20456000	16801000	21745000	29558000	5714000	9335700
1580900000	113160000	79399000	103110000	143950000	41459000	43852000
4813700	0	0	0	964610	310580	283750
0	1285700000	0	0	116880000	0	0
126660000	11660000	6588000	7728200	4394900	1308000	1078500
7505700	0	0	0	2572300	834380	799750
378780000	2784500	2475500	6084800	95802000	26867000	33469000
7167600	2565100	2287300	2737000	1164100	239760	282730
7590200	3399700	1997900	3935800	4709300	883010	1005700
59795000	75402000	41152000	94981000	17437000	3622800	2960800
3602100	0	0	0	674710	193050	269770
43727000	40396000	27450000	33429000	31926000	4926900	6284500
23501000	16138000	13807000	16761000	1692600	327870	375940
36311000	7986300	14067000	20652000	3676600	805200	944200
0	17223000	15438000	27842000	1890700	0	0
32068000	0	0	0	1819900	415380	658750
4387200	6921000	4608100	6537300	1068900	121530	83811
107090000	50439000	33931000	68983000	20980000	3764100	4814200
34581000	0	0	0	5506800	1651800	1693700
9035400	0	0	0	1140300	316010	413540
204970000	162700000	142720000	224350000	48522000	7156600	7968300
503660000	176920000	78286000	174820000	276660000	50141000	70901000

198570000	0	0	0	13821000	4786100	3668100
72323000	0	0	0	11436000	3502400	4127200
253650000	49891000	50361000	37911000	98728000	21989000	27763000
0	0	3537600	6366000	194190	0	0
367960000	36100000	32255000	56772000	96628000	23672000	28130000
4946900	4010600	2270500	6067500	6578400	1342200	912390
132410000	122790000	98331000	117830000	15680000	1804500	2091800
44791000	37079000	64921000	51921000	30572000	2731600	5760800
19026000	0	0	0	7863100	4058000	0
249650000	132600000	72902000	146760000	151950000	29006000	36961000
44005000	0	0	0	951800	264090	314790
18414000	0	0	0	1690700	340750	549310
28396000	0	0	0	1863100	481630	671620
22245000	0	0	0	2479100	703710	919800
56805000	3886600	2708900	5820200	10930000	3045100	3269900
27116000	0	0	0	1048900	319400	324800
6631700	2120900	1965800	3109700	4609400	0	0
27376000	19510000	16010000	22835000	7762600	857380	1547100
22271000	0	0	0	845760	248920	303800
0	138120000	154410000	147030000	4352000	0	0
0	154330000	0	0	8573700	0	0
488650000	139460000	130590000	186720000	23646000	5883500	5156600
18413000	0	0	0	2586300	789920	713270
309090000	0	0	0	11789000	3072400	3564700
24000000	19704000	24857000	40493000	1314600	198360	115690
15321000	38922000	37288000	49932000	2070800	139120	163370
4765500	0	0	0	1301900	418740	353690
0	25076000	390960	723340	2182500	0	0
274040000	68862000	64124000	87927000	15479000	3432300	4190600
8514100	0	0	0	498300	148440	151850
0	8786200	7410200	7901100	560410	0	0
56453000	0	0	0	4772700	1399300	1713000
14035000	0	0	0	3924600	1713200	935430
0	4612400	10810000	26548000	1134300	0	0
50147000	1051100	0	2746700	3303600	921220	1066700
43360000	43733000	0	0	18956000	4189100	5090500
15635000	0	0	0	4016300	1600600	1112800
29530000	4527900	4764500	5193500	2313500	494610	596260
46140000	11961000	10206000	13037000	4691600	1075800	1356200
55331000	48601000	39095000	52697000	19543000	2812600	2750000
3543200	0	0	0	540170	151820	179930
27288000	0	0	0	1947400	325510	741680
122870000	511860000	21764000	6197000	24402000	1641300	671060
91903000	0	0	0	16422000	7303700	2048400
31688000	55604000	58460000	67436000	8561200	806350	1092800

83194000	7488100	7958800	15946000	7318100	2083800	2137400
19514000	25520000	17370000	29858000	4125900	383690	560740
21869000	0	0	0	1759900	677390	549160
48172000	21751000	12035000	30092000	4780000	1012400	1161800
13768000	0	0	0	1838700	459320	780730
344890000	202420000	119040000	246770000	76322000	13717000	14545000
37956000	0	0	0	4013100	1238100	1369300
29928000	0	0	0	2665100	742040	957620
37397000	71665000	13959000	139030000	20425000	1155100	1799800
121470000	0	31840000	40156000	61660000	12995000	16421000
6253500	0	0	0	614430	184210	189700
64975000	0	0	0	7236900	1965300	2564400
803910000	471280000	370090000	561680000	96678000	17583000	19447000
16435000	0	0	0	2578100	1070800	724660
23794000	0	0	0	2996800	592500	1004700
0	5517100	0	2433100	496880	0	0
311300000	584240000	272090000	348460000	198860000	23656000	23590000
13580000	8468500	7713800	8477900	7233400	1209100	1775400
32826000	0	0	0	2685900	606460	711640
529660000	150150000	107890000	153760000	199620000	40005000	55005000
130250000	54880000	43917000	73508000	42712000	7749600	9749800
38011000	0	0	0	8583600	2948400	3101100
43914000	18573000	16653000	30736000	2727400	541790	615930
55508000	38811000	19317000	25745000	27436000	4261000	5751900
37712000	0	0	0	11571000	3835400	3021500
301590000	26593000	37188000	58656000	50256000	10872000	14441000
0	3670000	1978100	9995100	3910800	0	0
161460000	34439000	27230000	45705000	86481000	16424000	25252000
129250000	198340000	193480000	262440000	26563000	2727800	3216300
110550000	192330000	99331000	92304000	30032000	2942500	3541500
699700000	64863000	37950000	58497000	48898000	12809000	13431000
13414000	12261000	5588400	11156000	2904000	441220	534590
233550000	159180000	132200000	221780000	76186000	12519000	13887000
8905600	0	0	0	477290	135800	155960
13827000	0	0	0	1086800	297100	383060
1760400	2701500	1574900	1494900	1040200	203310	0
7479300	3132100	1760200	3846600	2033100	327130	458380
64281000	2034000	2205800	2801800	6135200	1828200	1929600
125120000	29338000	25848000	38724000	8362800	1929700	2221000
47629000	0	0	3261700	3257700	941800	1104300
21516000	0	0	0	2759400	839900	653820
98362000	0	2678200	4608000	13843000	4425400	4135400
22189000	0	3198200	4083400	2848300	736470	830460
169980000	189050000	167330000	276620000	52068000	6923600	4995100
7958100	0	0	0	4871400	1249800	1632100

14994000	12860000	8688100	20055000	3267800	513770	490140
3286400	1936400	1356900	3559400	1409400	210190	277510
47345000	0	0	0	6537500	1823800	2346500
53031000	13378000	10384000	16349000	13071000	3075900	3342000
38841000	38282000	16739000	22685000	9358300	1335200	1889000
0	908230000	2449900	0	29377000	0	0
17489000	0	0	0	1743900	465980	605250
94590000	148630000	136030000	206310000	25036000	2240100	2603900
0	0	0	0	0	0	0
46451000	0	0	0	3542700	1095500	1081000
6504400	22792000	19249000	30049000	6363000	387020	362060
103680000	116550000	62975000	186090000	19028000	2421000	3198700
184830000	401570000	121250000	231900000	37184000	4154900	5396100
863210000	216560000	93670000	181380000	89918000	23251000	22964000
0	6039900	5766200	8220700	514510	59686	103470
166070000	16542000	7417100	8288600	9404900	2585800	2599500
13231000	1609100	1509400	2349100	1076600	235900	321230
35121000	0	0	0	1537800	385550	525060
11519000	0	0	0	7980200	2161500	2939000
18150000	0	0	0	3797800	1519800	1143600
61352000	0	0	0	4732000	1287200	1691900
162840000	85871000	51937000	75297000	12798000	3124400	2302300
13374000	0	0	0	1746100	1003100	0
11807000	24894000	11405000	17348000	10458000	1778100	2134000
14684000	2343000	4192700	17634000	33231000	7123100	6680600
7598800000	955160000	779240000	1308200000	617710000	166740000	163360000
2898300	2472200	1529900	2919000	526260	73696	101870
76088000	0	0	0	6347900	1755000	2479400
218720000	22553000	6358700	20897000	9953800	2800800	3021800
6322200	7268800	2791700	4779200	988360	170200	156850
19056000	0	0	0	2697000	740440	953620
118010000	9596000	9745100	11318000	20709000	5448500	6515900
10207000	0	0	0	15684000	657800	13325000
47047000	9253600	1411000	20966000	14694000	2729400	4096600
158360000	170820000	49192000	124590000	93994000	13801000	17321000
3671900	0	0	0	2205000	535220	751830
9805000	0	0	0	1022200	299330	359710
139900000	141200000	108210000	186240000	39646000	4894500	4459800
12382000	5080600	3123800	5288700	2695900	497920	675930
5747600	12018000	9333600	14339000	6548600	650290	718690
0	4595900	3257900	3952600	1967700	0	0
690770000	75856000	66881000	102550000	116720000	25255000	32967000
228590000	54651000	53713000	45122000	99543000	14449000	30511000
0	5164100	2420500	5776300	1670100	0	0
740350000	348710000	216650000	333190000	555300000	125800000	156350000

72354000	46840000	0	22103000	86049000	18621000	20328000
182440000	53181000	51434000	133680000	391290000	61458000	224650000
653880000	559900000	339430000	604340000	205200000	27618000	33743000
20937000	4947900	7398600	11187000	3764600	725090	1018100
1876600000	1306000000	1451500000	1831300000	1360100000	201320000	235130000
8676700	0	0	0	652750	210680	219590
1369100000	75746000	45060000	75372000	164020000	56731000	64989000
104930000	0	0	0	8310400	2343100	2469700
14461000	137330000	92474000	169910000	12586000	308000	444640
2939800	0	0	0	462370	102160	164230
529470000	368250000	221270000	461410000	202800000	31344000	39756000
244860000	356970000	277820000	531280000	37799000	4274200	5859200
9975800	0	0	0	2341300	729040	780910
19164000	9078400	2167600	5113200	2042000	390210	467640
28028000	7095600	4053300	6382500	6669600	1398500	1766500
5437200	15249000	18777000	10187000	2287000	229370	148040
14566000	7028700	7645800	10922000	8126100	1299000	1806800
160780000	15899000	11783000	18731000	14298000	4061900	4480800
0	47896000	0	0	1596500	0	0
5105800	0	0	0	349670	97397	114270
175980000	945230	1180900	4341500	23095000	7085200	8077000
3387500	0	0	0	1500300	407280	609040
3760900	0	0	0	287430	0	123920
12164000	0	0	0	909730	213140	384690
109440000	13291000	12397000	18353000	10906000	2889400	3502700
3657000000	0	0	0	783180000	203730000	364330000
139850000	1599700000	1379300000	20098000	50653000	1424500	1668300
26737000	24890000	14814000	27024000	5510300	806950	1108500
212950000	75813000	73480000	89921000	46431000	9812900	10021000
121480000	0	0	0	9730300	2592800	3341100
42422000	0	0	0	3204400	1055800	1032300
5470900	3589800	2163900	5794900	2873800	535840	446900
0	0	0	0	94020	0	94020
94278000	0	0	0	3808200	1055700	1231900
172660000	20576000	19753000	24975000	12026000	3869400	2207600
0	20377000	12483000	13605000	7066300	1258200	0
337510000	216930000	149320000	248630000	53119000	9701200	10576000
6723300	7664800	31620000	39843000	12049000	606550	711200
43916000	5250300	13513000	14604000	5879800	864960	1654600
14053000	0	0	0	1792100	538550	550920
420390000	295880000	155970000	531590000	168810000	23882000	27939000
73359000	11987000	0	7419900	23636000	5925900	7402700
1376200000	126540000	82905000	112980000	726020000	209790000	233120000
19923000	0	0	0	2766200	1058800	841210
643030000	340020000	275620000	448900000	60434000	10020000	13293000

71756000	25005000	6172000	31280000	3668900	801240	922550
260270000	0	0	0	6809300	2222100	2343600
139660000	0	0	0	2854400	796760	975040
1919000000	0	0	0	165000000	48971000	56061000
21638000	0	0	0	1275400	365620	340340
7013500	0	1342200	1418300	3644100	934530	1080600
308170000	2723300	8571400	10039000	60431000	19788000	17107000
77425000	5242800	3518600	4570900	10558000	3243000	3189700
205810000	5815900	4676400	7306100	118720000	39094000	34905000
140560000	122580000	81895000	100040000	15183000	2261200	2805900
20363000	31816000	33534000	49110000	6723300	651860	678460
642380000	200670000	164510000	292820000	34886000	6508100	8675600
518060000	0	0	0	41467000	10777000	14991000
7032400	0	0	0	1380900	409310	469300
43393000	10901000	16967000	25089000	5939400	1076700	1421600
2393700	0	0	1550400	334330	84184	70865
274300000	3857500	1759200	5767900	49624000	18089000	13679000
83869000	92826000	67040000	118430000	19378000	2749400	2699100
11527000	0	0	0	919120	270930	298890
187770000	0	0	0	20989000	6321100	7156800
11604000	0	0	0	1103200	266270	390590
0	0	0	0	0	0	0
179270000	158500000	142670000	194300000	16234000	2429300	2368600
3720200000	77744000	47790000	95095000	485740000	150470000	156140000
29411000	5002200	0	2628100	8225100	2515300	2623100
43185000	42398000	35460000	95778000	18501000	2232900	2716400
13903000	0	0	0	1638900	503180	503770
20931000	0	0	0	3735300	1494200	845760
8361600	0	0	0	1271900	382860	424470
6787700	8407300	4240000	10444000	2717300	355210	370190
140190000	54723000	48795000	61262000	14066000	2667800	2926600
38424000	0	0	0	1458800	459350	408340
4080100	0	0	0	248350	53978	97225
26792000	0	0	0	4305700	1345500	1471700
17854000	0	0	0	2161900	722580	801630
71125000	0	0	0	4510500	1825600	1262400
32553000	19588000	10824000	26820000	6485300	1105200	1299000
4430800	0	0	0	853230	272280	240120
20364000	12891000	8275600	17230000	6235500	1166100	1396900
7480400	0	2673700	3646600	5023600	1052200	1211300
0	38856000	0	0	1942800	0	0
26030000	14314000	12592000	18947000	4907500	824950	957250
17496000	0	0	0	1174700	213980	487890
327210000	7027600	3331000	7879000	13215000	3935900	4343700
22961000	0	0	0	1331500	468890	273900

215880000	60239000	38491000	50198000	19226000	3600300	5202100
0	17436000	11466000	18074000	2609800	0	0
320100000	30250000	25914000	34572000	66671000	17142000	20183000
7862700	6280500	5320600	7264400	2037000	235690	316420
0	5364000	5820500	5296500	1831200	0	0
18901000	105050000	4943300	15468000	5003200	521470	780040
17667000	0	0	0	7565500	2340400	2701300
14650000	7632900	8837500	8943700	4983300	861900	1039500
12185000	0	0	0	8454800	2575900	2832500
6406000	0	3008500	10035000	2344100	723270	0
211060000	0	0	0	15133000	4207100	5371700
0	2838100	4397600	9109900	860290	0	0
29004000	2034900	0	8261900	11130000	2927700	3289300
0	8090200	0	0	622320	0	0
52348000	0	0	0	8822600	2347900	3395400
43154000	52133000	39654000	72817000	6704300	779360	978360
5288700	0	0	0	927550	257310	339690
12865000	13128000	12829000	17889000	9337000	987660	1260400
0	0	850690	2665100	85751	0	0
8631600	6282300	15464000	18912000	3821600	477140	606130
18020000	0	0	0	1133700	306220	398460
5241700	4305800	3377000	8873800	1161500	132300	190820
12924000	2143000	9245700	11544000	2077100	388960	407500
7037200	0	0	0	4545200	1568300	1569500
53170000	56445000	59162000	73807000	8392600	959650	1212800
8833400	0	0	0	3507700	1378800	867030
77601000	16217000	9890200	25232000	32078000	6325600	7331900
0	3296600	2281800	6058600	2909300	0	0
233100000	57903000	35079000	61980000	109380000	24363000	29575000
18269000	0	0	0	4528700	1245000	1622900
0	16961000	5635900	21708000	2606200	0	0
0	66662000	42288000	65276000	4467400	0	0
27730000	0	0	0	6789300	2022400	2456000
23200000	0	0	0	10812000	2947600	3997800
130950000	11672000	11374000	17903000	34090000	8140100	10323000
48479000	147120000	145560000	246370000	16577000	899270	989660
142610000	0	0	0	35117000	9504600	11351000
30597000	1580300	2005900	0	6012500	1920600	1955400
24580000	0	0	0	2512800	788160	814320
3047900	0	0	0	901710	261790	301260
21056000	0	0	0	11705000	2847200	4646900
5596300	0	0	0	1315300	518230	502530
73057000	0	10032000	10695000	8966900	2561800	2653700
4888500	0	0	0	1716000	433130	584510
5948400	0	0	0	658290	189420	240080

20690000	0	0	0	3357700	1050200	1218600
8290200	6375600	3972700	10835000	713770	0	86684
123520000	0	0	0	6695900	1835900	1919100
0	4622700	1501700	3042700	1833400	0	0
4984900	0	0	0	1012100	264280	332450
22333000	34460000	17500000	31457000	17193000	1871200	2103300
16257000	19087000	15875000	14733000	4102200	490610	613770
20866000	19951000	18825000	21082000	5566000	742130	979830
808560000	11484000	5668400	10313000	51166000	14093000	17630000
40486000	0	0	0	4133000	824160	1284500
10904000	37683000	25414000	49717000	3486500	219590	173940
49446000	6701400	4209600	0	7216800	1457000	2406700
27070000	25440000	11259000	17189000	18908000	3201000	2213700
8924500	0	0	0	3657400	1216900	1325000
6798800	0	0	0	940790	260910	0
34442000	3617300	11026000	16821000	6020400	807870	1335700
16133000	0	0	0	39315000	15393000	7789900
354360000	201330000	68364000	215630000	75355000	12932000	13031000
57995000	57975000	28729000	34957000	26134000	4795900	5005600
3292600	0	0	0	838410	297020	267000
39198000	0	0	0	2087900	687760	566090
6601800	4336300	4113200	2854700	4601800	741480	1302300
6948300	0	0	0	388370	103280	173020
412000000	0	0	0	41631000	12154000	14218000
313950000	68604000	38543000	69498000	19948000	4889500	5973500
34450000	3148200	2950500	3920200	2553500	798000	788740
0	36115000	35417000	41792000	5151100	0	0
252420000	13176000	8830400	19001000	19835000	4785900	7326800
12971000	38173000	28896000	47295000	5096800	547250	570290
74144000	96838000	76681000	103850000	18157000	1726200	1783900
245670000	132140000	78287000	153110000	26491000	5345500	5524800
0	5174700	3981600	28167000	4487300	0	754950
24349000	17803000	15645000	25848000	13457000	1945000	2217800
8263000	0	0	0	431860	109780	160060
15521000	0	0	0	765310	319700	163400
0	48859000	20784000	44589000	11423000	0	0
12660000	0	0	0	866460	164230	360070
9185900	12462000	5566300	5593800	4763500	636140	846520
44319000	10536000	6295600	12216000	7147200	1780700	1872800
522230000	68190000	45359000	87610000	73519000	20479000	20159000
8913100	2320200	1896100	3741500	1574100	365170	475390
9419000	0	0	0	904490	242660	384800
11050000	0	0	0	6066600	1820000	2036500
0	757880	893930	1248600	966820	0	0
6066700	2824100	2453700	4030400	1268500	271520	264810

23476000	4039300	3829300	5505200	16088000	3633400	5084100
17122000	0	0	0	1553500	477420	485670
0	0	0	4072600	339390	0	0
106790000	36755000	31185000	59744000	18417000	3435300	5211900
823020000	85674000	51608000	73985000	69632000	20261000	21417000
0	0	0	0	1776900	1776900	0
47784000	0	0	0	4866600	1076400	1275300
7790400	0	0	0	994930	320570	264350
112090000	67884000	58839000	63259000	46864000	7541200	9115400
46816000	0	0	0	1714000	457080	606720
77722000	0	0	0	3978900	1327000	1212600
52136000	18355000	17702000	23988000	5336000	1037600	1266500
76878000	7194400	7960100	10097000	3355700	911570	1082400
28883000	0	0	0	1155400	351670	366150
0	7846300	3054400	7705200	1240400	0	0
19270000	0	0	0	1044100	314680	351530
6229600	0	0	0	322150	96163	106180
93715000	0	0	0	7775200	2170400	2481000
8590500	0	0	0	656890	313270	0
120830000	0	0	0	3985800	1248200	1208100
613590000	0	0	0	13572000	3874400	4408300
17979000	0	0	8345500	7291400	2260000	1740900
81190000	47634000	17894000	26659000	5856300	500910	792790
13011000	5740100	6991300	10517000	3451700	425220	760200
167570000	0	0	0	3135100	882420	1128000
43627000	20324000	9007400	41367000	12548000	2359100	2567300
0	10853000	6961900	9156300	1226000	0	0
10239000	0	0	0	3245000	1538600	0
9874900	0	0	0	4663100	1342500	1674800
34385000	6597900	4688700	9585900	9824400	2345600	2455400
0	18006000	7041800	12431000	7495900	0	0
85737000	12639000	5338100	13007000	58244000	12054000	17010000
10172000	0	0	0	3261200	951920	1179000
8991700	4570800	1665200	5392700	3451800	815350	761820
21019000	0	0	0	13846000	3322700	5268200
23363000	6185400	0	4606400	15599000	4677200	4090500
540820000	9632200	20581000	20272000	37644000	10944000	12278000
6115300	4117700	3004400	3417700	5547100	935830	1280200
11692000	19506000	8748400	7999300	10041000	1438000	612510
10100000	0	0	0	7838200	2314400	2157100
510720000	26024000	21685000	36208000	207600000	54346000	68303000
58336000	60960000	17062000	57970000	17786000	2697900	2942500
0	4255600	3155300	4788500	2439900	0	0
128920000	35010000	8033200	25415000	25377000	6459400	6581800
52889000	0	0	3648300	3307600	906880	1085900

0	403450000	0	0	14943000	0	0
0	64657000	0	0	2229500	0	0
55093000	46721000	34819000	52043000	3278800	482490	652230
6771500	5432200	0	0	962760	371170	210230
0	1077800000	0	0	39919000	0	0
0	117180000	0	0	4040600	0	0
4145400000	3680400000	3234400000	4528200000	2115300000	353190000	344980000
1203500000	4066400000	393400000	414400000	267480000	34783000	23123000
0	0	3439300	0	132280	0	0
2178100000	289810000	3830200000	455460000	847260000	53092000	43776000
162710000	56487000	1193600000	45451000	139440000	3343400	3528800
397280000	55086000	106090000	50543000	134010000	20772000	11736000
165240000	37938000	372020000	16958000	88242000	2628500	1019100
671710000	56375000	449280000	115760000	127070000	4677100	4837000
1,6E+11	1,2918E+11	8,5604E+10	1,2208E+11	1,9122E+10	3361700000	3338400000
7128200	0	0	0	2664200	882110	763830
988490000	3244200000	217720000	296800000	182450000	15094000	9111800
324700000	33669000	46110000	51789000	36704000	10495000	10475000
1075400000	3518600000	2560500000	4030300000	260680000	16353000	20636000
341330000	4312400000	24136000	64658000	167910000	2308000	2066900
3,6872E+10	1,2985E+10	7910200000	7655400000	3221300000	483470000	221560000
612720000	1010100000	388690000	319840000	107130000	16575000	15351000
0	15467000	11365000	18071000	2494600	0	0
1004100	2673600	2072100	1502200	718230	194890	70088
8520600000	6784900000	3456000000	2199500000	1855700000	419990000	629540000
33165000	0	0	0	1074200	223760	0
0	3495500000	2439400	856930	106020000	0	0
230580000	347200000	25850000	41827000	34666000	6651700	5757500
0	8092500000	9419400	1962200	312850000	0	1162800
84631000	60569000	68703000	57109000	15584000	2201400	4037300
8881500	0	2246100	3796900	2932400	565490	708660
17011000	9501300	8124000	14649000	762710	121330	143560
26140000	64001000	3248100	3918000	4322900	225850	741570
99852000	56763000	0	1004800	13356000	4045800	3875500
6126400	0	0	0	1671000	643430	414930
0	145640000	0	0	5201600	0	0
273080000	29868000	14238000	15926000	5317100	474670	341020
0	0	0	0	883580	0	883580
50157000	49500000	16981000	25299000	7951200	1682500	1690000
0	2523200000	2744700	0	90214000	0	0
0	5490300	0	0	322960	0	0
36704000	38847000	8291500	4805900	4454800	801010	967440
632270	1,6789E+10	21576000	5153200	543150000	45612	642080
72757000	5298600	9102400	6514700	5315700	1044300	1041300
0	0	0	0	0	0	0

266700000	8650800	5979500	3974500	9661900	290070	168360
421940000	203660000	115600000	112130000	17800000	2298600	5462100
0	4763300000	3839700	0	681020000	0	0
1163900	919920000	988780	0	28815000	0	0
281490000	0	0	0	158770000	84597000	38986000
0	489360000	0	0	81560000	0	0
0	372920000	0	2758700	12523000	0	0
368580000	174620000	134510000	165870000	42399000	6803000	9233700
109790000	0	0	0	12784000	4426600	4815900
29661000	0	0	0	2207600	565430	769850
6536200	0	0	0	739750	225970	295900
25803000	0	0	2334400	934330	257290	269240
7210200	2842000	2344900	4050000	3185800	621340	737040
80701000	59863000	5665600	24671000	31183000	5299100	6894500
44006000	65076000	45264000	74786000	11772000	1197600	1409500
24001000	0	0	0	7197700	1936800	2594000
15785000	3510500	2714400	5079700	874560	251740	212370
0	19685000	7196600	22449000	2466600	0	0
39078000	25558000	16258000	30501000	16547000	2689100	3731300
9512600	17870000	5870500	10503000	3668900	543400	0
57928000	0	0	0	6141900	1765700	2148200
43232000	26976000	16431000	25724000	6403900	1270600	1828500
49272000	40635000	6709200	13429000	5011600	1145100	1115300
97618000	0	0	0	1326700	350150	381280
143390000	175800000	190070000	225150000	19136000	2068800	2378400
24529000	38722000	66805000	109650000	16025000	1090700	833560
6749200	0	0	0	3477500	1234400	893270
142110000	22412000	14719000	26013000	43677000	11559000	11592000
0	10343000	0	0	1477500	0	0
9079700	17349000	38220000	9234800	10789000	563870	989500
123800000	0	0	0	10662000	4716100	562800
26984000	0	0	0	1560400	328820	604000
77518000	71935000	50739000	74082000	10202000	1460200	1708600
14130000	28840000	21718000	26649000	1536400	141820	176780
152030000	21316000	16611000	42267000	22252000	4965500	5675000
46800000	31836000	14406000	39444000	3210300	461560	678620
18438000	13676000	8304100	15653000	4609800	737550	757150
49918000	873920	12707000	11026000	3407300	915560	939170
6100800	8454000	4487500	7048600	3176500	382670	421920
15007000	0	0	0	1848100	280680	413020
37149000	9542900	4158200	11276000	6748600	1714700	1927600
29315000	6158400	0	6024600	43917000	10802000	12367000
265710000	21490000	13859000	31868000	129380000	33287000	40609000
13431000	0	0	0	4794000	1606000	1269300
31429000	9456400	2310700	8776800	46109000	8105800	12016000

155010000	0	0	0	25170000	8297800	8713100
17157000	0	0	0	3067500	857040	1257300
0	28231000	27534000	29963000	12247000	0	0
78063000	46789000	31954000	68841000	10457000	1744200	2265300
59633000	3575200	3844200	6352800	775970	223500	217290
0	28994000	11492000	9149500	49636000	0	0
26006000	0	0	0	8788100	2388500	3148900
732250000	12299000	7331800	20666000	71867000	21652000	23575000
45752000	0	0	0	1463200	439070	479430
11651000	9369800	8721000	10269000	1183100	190370	159140
4088000	0	0	0	1407600	377900	518730
252050000	0	0	0	30575000	10762000	7811200
18676000	15290000	9172400	10440000	9506700	1646800	1906800
15614000	0	0	0	341180	149750	41287
6577600	4556400	4754600	5713400	683270	168840	187130
0	0	0	3432800	381430	0	0
808090000	217840000	224150000	379740000	60233000	12813000	16077000
283150000	121350000	136210000	215000000	78251000	16617000	14403000
12572000	0	0	0	4268600	1411400	1285600
377180000	10613000	5875300	22885000	56620000	16423000	18273000
113230000	44757000	28673000	60953000	9893300	1809400	2044600
6291500	0	0	0	2270400	543610	828030
48545000	55826000	47087000	75893000	4126700	523470	571900
77002000	8403400	6540200	9692200	6886800	1852700	2210800
14308000	0	0	0	3107900	971300	944210
5811000	0	0	0	5118400	1496400	1685000
707890000	110440000	102960000	129290000	50413000	11032000	14948000
114880000	0	0	0	57882000	19435000	22036000
11162000	6478500	6225000	6535000	2403900	458790	497500
15394000	0	0	0	2316500	720300	634080
7722600000	3274200000	1817800000	4155700000	1608300000	326270000	339240000
0	10821000	2985600	4589200	1082100	0	0
36294000	0	0	0	3660100	781720	1228600
126500000	5126400	5067000	5131500	11937000	3436700	4202200
39751000	0	0	0	4637600	1505300	1403900
87768000	107240000	84949000	126710000	62737000	7671300	9880200
3681400	0	0	0	604930	341970	0
69966000	34156000	0	54534000	17721000	3337100	4468100
274490000	130540000	126030000	166080000	80539000	13388000	17356000
0	0	0	0	0	0	0
6017400	0	0	0	246140	102660	64305
517660000	94058000	84181000	110640000	203000000	46103000	56079000
44470000	0	13570000	13487000	22865000	3402800	7541200
33193000	119650000	100450000	219860000	22345000	1116900	1513600
1628800000	1489200000	1283900000	1734900000	237770000	32745000	39165000

9584800	8675400	0	0	89741	22054	24620
15970000	0	0	0	5336200	1902200	1152600
6515900	4195000	1320800	1583800	2231700	478500	515430
8890300	0	0	0	279810	0	123840
73292000	0	0	0	11367000	3476900	4032500
3,2399E+10	1,5169E+10	7567400000	5773700000	3209500000	531260000	713400000
18430000	0	0	0	1187000	375750	392350
219540000	8646500	15292000	21846000	11712000	3417100	2880000
42484000	45597000	30240000	43927000	10007000	1376700	1575800
69251000	40884000	32505000	53249000	46276000	7707800	10584000
35844000	0	0	0	1820200	637270	493670
223600000	122590000	123120000	167820000	64802000	9472900	12854000
0	7467500	5552600	11145000	1726100	0	0
26168000	3983100	2060800	3347300	1747700	408830	530730
95644000	88432000	46526000	75887000	58432000	8964000	11157000
301970000	0	0	0	8406700	2538000	2755500
464290000	203140000	189090000	219520000	254150000	44159000	56268000
17337000	0	0	0	3616300	1252800	1029800
87475000	0	0	0	6393900	639920	608430
10622000	4863800	3141700	6758300	4288000	756930	710390
3424300	0	831550	1561300	702180	156970	129690
0	74351000	59532000	85143000	24336000	0	0
0	3250500	2606100	4029000	1977100	0	0
248090000	138890000	123330000	129520000	75388000	10022000	19663000
125820000	23480000	19251000	23188000	6723400	1523300	1950300
0	16533000	13526000	12362000	8484300	0	0
9899100	2869700	2705600	4405200	5585100	1275600	1469500
93952000	11446000	6724000	11842000	31877000	7300700	9081300
18557000	0	0	0	3152200	834730	1080300
18601000	12438000	3047200	4415800	2921100	618860	628220
0	15396000	0	0	1539600	0	0
292810000	19547000	13332000	19022000	33512000	10123000	8401700
4898200000	2198500000	1215100000	2331000000	578460000	115190000	130680000
81083000	17359000	13849000	21804000	50321000	7857800	15644000
510390000	558560000	460380000	757960000	237320000	27409000	33966000
29898000	7595200	4140700	8067900	2551800	414630	631040
12472000	0	0	0	1383100	411530	472730
20959000	3122600	2905300	3275700	2634900	530580	788530
95966000	85964000	43376000	107320000	36041000	5116100	7165800
363720000	91374000	97178000	119390000	63249000	13731000	17533000
53973000	0	0	0	4592400	1435000	1698700
16499000	0	0	0	4847600	1373800	1640600
68798000	0	0	0	16997000	5221700	5521100
82126000	84743000	46075000	64833000	41464000	5901800	7784300
56371000	28681000	22940000	38500000	21859000	3771100	4770700

11192000	0	0	0	2626900	585090	1024400
0	7174900	5093900	6813800	1363000	0	0
74011000	3440700	3787700	5217900	17384000	4664200	5515500
49617000	0	0	0	16675000	4538300	5934200
5599400	0	0	0	1908500	742220	544130
23970000	0	0	0	2579600	1158400	594690
29050000	13745000	12859000	14247000	17039000	3287900	3765300
1305200000	71701000	47115000	80216000	65254000	18478000	20386000
20201000	0	0	0	4111200	1343100	1214100
15242000	22129000	0	4976900	6619000	1105300	1278900
83669000	116000000	67371000	57152000	65602000	9032400	10257000
0	0	0	0	122010	53749	68262
252230000	14903000	10069000	15889000	78365000	25516000	23540000
83374000	0	0	0	11417000	3633900	2878800
1383000000	0	4543500	10672000	116780000	34114000	38975000
4263100	0	0	0	1233200	324730	375580
103620000	4547300	22954000	29049000	4530900	1200500	1423600
12986000	0	0	0	160340	42641	55569
346060000	12307000	13211000	17181000	23625000	6380100	7763100
592980000	209640000	162620000	254200000	96921000	19517000	24385000
127710000	1925500	2172100	3695600	6342300	2161600	1624100
97262000	0	0	0	11599000	3379400	4166800
20096000	0	3286200	4689700	3055400	533730	767190
182850000	15364000	7912300	13551000	10386000	3097400	2895400
3648900	0	0	0	492310	122020	218260
155910000	212470000	161040000	238170000	40679000	4252300	5722500
13265000	0	0	0	1517300	466420	498170
100910000	11156000	5602300	22545000	7218100	1382700	2145500
413180000	405130000	157690000	249670000	48659000	5065400	7544300
0	10824000	8815600	10264000	1993600	0	0
28295000	14877000	7355200	11433000	3398500	476610	856540
429450000	377180000	237450000	419820000	142060000	21731000	22732000
672290000	1286300	0	3352000	65541000	17173000	23297000
26259000	36422000	30410000	46726000	1633600	202210	183030
7131000	0	0	0	2788100	411100	0
372560000	130040000	70644000	168190000	48176000	8788600	8494100
219030000	37224000	32933000	52161000	21158000	4538200	5608400
29431000	0	0	0	1439600	484230	464890
106060000	0	0	0	4978500	1589200	1591600
22584000	1735200	7853400	15359000	3298800	397130	638280
21384000	34954000	29149000	51243000	16029000	2006600	1592700
19687000	5368300	760120	6114100	4199000	1131800	1293300
166940000	78905000	86403000	123920000	20498000	3041200	3633500
35517000	2939100	2567800	6527600	14594000	4199000	4451500
124950000	169940000	107040000	240250000	30478000	3381700	4160900

54716000	106930000	27682000	85673000	40809000	4159200	6093800
190350000	62697000	52136000	75032000	9787000	1638000	2016600
160770000	63694000	37578000	44310000	50683000	10589000	1799500
133280000	29371000	25490000	39905000	3839300	906160	1000600
173870000	4738200	3147800	3700600	3741200	1032900	1364500
55924000	79908000	61023000	96079000	43321000	4805500	5967200
0	5113000	2819400	9895900	1980900	0	0
7168200	0	0	0	223310	66492	78047
22700000	0	0	0	1488100	234550	604960
102670000	9249800	5489000	7433300	5909600	1568000	2112200
143620000	0	0	0	2659400	798740	764340
0	179070000	0	331660	14950000	0	0
297910000	54869000	53125000	87403000	20374000	4652200	5654700
953100	16537000	13982000	16153000	6727400	1144900	1613800
139350000	52761000	51718000	82082000	9208600	1739000	2037900
124420000	64304000	59147000	81175000	13425000	2192500	2795000
0	104730000	0	99941000	4873200	0	0
13082000	0	0	0	1947100	524920	733630
0	8125300	7167400	8308100	674310	0	0
24375000	0	0	0	4217400	1183900	1510100
11067000	0	0	0	1105100	373780	405840
34705000	0	0	0	3099300	929590	1050200
272340000	56671000	37040000	74012000	46134000	10780000	13351000
105140000	20781000	19516000	29686000	4721400	970720	1284200
9151400	0	0	0	1694800	365250	625540
5625600000	3676200000	2383200000	2885600000	595190000	110050000	129760000
61037000	72041000	50627000	73323000	46014000	5680100	8205700
39360000	0	0	0	4926700	1824300	1134400
91549000	88408000	25096000	42303000	19588000	2828600	2209000
197770000	16830000	13240000	18853000	39503000	13049000	8833200
214770000	5522300	2992500	0	15888000	5290800	4562500
83801000	0	0	0	4673300	1277600	1533500
0	7183900	4607400	4890500	2085200	0	0
988810000	464610000	500480000	624810000	243160000	51780000	55662000
65601000	34974000	12495000	14960000	11597000	3281400	2496100
4262900000	3433000000	1718100000	2322500000	1500500000	232260000	290200000
2557700000	103840000	64175000	101170000	389680000	121050000	119850000
47967000	0	0	0	1458100	490280	382850
20316000	0	0	0	10675000	1850300	4761100
82816000	12926000	12964000	12220000	28547000	7779000	8675100
812890000	45090000	19286000	41974000	72319000	23009000	22273000
513030000	450260000	326050000	468570000	295010000	44492000	55190000
654390000	1237700000	1058800000	1621700000	568180000	46399000	64526000
45851000	0	0	7910900	26404000	7722900	7928800
0	5086700	4880500	4493200	535570	0	0

5160200	0	0	0	1896700	572850	586720
6775200000	128860000	68317000	94838000	650600000	203030000	211990000
1,0447E+10	371790000	237170000	385070000	883010000	260150000	276180000
1225600000	222730000	145750000	263330000	47477000	11301000	13245000
8571000	0	0	0	4668700	1756300	1841000
160680000	29940000	21242000	68841000	29656000	7996000	7624700
4367700	7180000	0	3691000	2821500	671740	456530
48223000	18241000	9599700	12529000	5346100	1142000	1251100
140090000	81902000	15398000	31009000	10994000	2525900	2503900
2113300000	2495900000	1449500000	2168800000	570980000	71563000	88047000
729800000	1166700000	461290000	490130000	26514000	4014200	5027400
828540000	426490000	649690000	588000000	397880000	47283000	101330000
1154500000	1146800000	2255600000	1512700000	935080000	79607000	181080000
56723000	0	0	0	3331800	1053300	1045400
31699000	6678300	4712100	6954800	7715400	1744700	2121100
17937000	11251000	8503100	9863800	6041900	1088200	1295500
6662300	0	0	0	8829900	2796500	3812600
286880000	53425000	38224000	54046000	28819000	6752500	7647100
104470000	0	0	0	8839400	2945700	2523600
74731000	32755000	25537000	44952000	45766000	9219900	11121000
1191500000	11945000	1914100	3957100	45391000	13238000	16028000
59822000	0	0	0	8926300	2807600	2970200
1766000000	180990000	148220000	196570000	158630000	44263000	48885000
263400000	98510000	51284000	62366000	6850600	1472400	1829200
6146400	2595100	0	2959800	1283400	338990	294380
693220000	141010000	79037000	153930000	132710000	29039000	36976000
30634000	0	0	0	4564000	1593600	2069400
123140000	157310000	46337000	84566000	21054000	2276200	2956300
31342000	2784900	2292300	2621700	4120100	1203300	1142100
0	40685000	8134800	2981300	4316800	0	0
192950000	31951000	25553000	48377000	32248000	7637300	8882500
655800000	143970000	77850000	114980000	196480000	49096000	48126000
161580000	45469000	28084000	54051000	79238000	19131000	18796000
185660000	70049000	31713000	35621000	20811000	5423100	4967300
669500000	446690000	327630000	484390000	197070000	30763000	37761000
0	8798900	3148000	5755200	2950400	0	0
1118100000	1098800000	780710000	1478800000	332140000	56180000	40361000
922780000	286420000	243250000	385490000	400690000	75572000	95377000
36156000	0	0	0	3109600	993930	985830
1541500000	15689000	10150000	18115000	220170000	48251000	113200000
0	4511500	603120	0	2557300	0	0
516730000	954060000	829210000	1131800000	273260000	27615000	31165000
348090000	80895000	68458000	125780000	126990000	28796000	28942000
0	7110700	9023400	5842800	3139600	0	0
12207000	0	0	0	2414900	681730	861220

###########	0	0	0	139180000	45340000	44888000
22611000	98311000	22422000	26747000	12841000	1046200	1164100
0	25834000	62655000	75682000	23453000	0	0
150830000	0	0	0	8364000	2426200	2728600
7594200000	0	0	0	1879800000	617790000	629110000
180170000	29080000	16766000	39401000	25723000	6360300	7298100
186390000	28712000	21388000	40371000	10268000	2457800	2953200
385550000	787180000	625130000	772810000	150090000	12639000	15035000
1,3907E+10	8428500000	5107700000	5051500000	1153800000	205230000	136230000
54553000	0	0	0	11358000	4258200	2902900
1007300000	217010000	184520000	246870000	196750000	39441000	53831000
285660000	127140000	136240000	169400000	165880000	29987000	33257000
505750000	342040000	220110000	286390000	72531000	6142200	12217000
164080000	153950000	62373000	144740000	31102000	4287400	6616800
520080000	128410000	70916000	119010000	78084000	15693000	18264000
26807000	4528400	2686100	4523000	1065900	243070	287540
385220000	138980000	109190000	187140000	168820000	31823000	45823000
15406000	0	0	0	1534300	443400	520280
5555300	0	0	0	1609000	476510	515250
2023400000	1112400000	856790000	1286800000	235810000	44522000	44633000
122340000	5778700	3784700	5701700	5702400	1585600	1744300
104840000	45958000	20582000	23200000	94588000	26858000	19085000
173340000	0	0	0	6758500	2093400	2115900
370680000	78054000	69965000	113150000	27561000	7221800	6298300
287740000	256480000	172080000	331550000	97347000	14929000	16928000
188610000	46439000	38767000	51379000	98880000	18906000	25775000
121090000	35085000	15280000	36416000	53685000	13263000	14438000
32286000	0	0	0	4980800	1826300	1255400
27266000	0	0	0	9413600	2931700	3073700
10720000	0	0	0	1139600	342510	427450
20320000	6454900	0	5661500	470180	129940	80749
104850000	0	0	0	9404700	2966500	2943100
37168000	0	0	0	350160	133520	27973
161920000	83477000	69547000	90429000	81116000	10877000	12329000
36982000	31296000	31000000	34362000	9082400	1372700	1635100
10714000	4733000	6188700	6610400	1760100	255870	327280
114670000	10610000	2313700	21716000	4230000	1051900	1288100
179220000	93806000	74642000	134550000	24283000	4389600	5280800
199580000	249150000	230280000	306460000	7425600	962800	1220900
847940000	13015000	19867000	25451000	193080000	52759000	64799000
4163300	0	0	0	2208200	820390	0
94704000	0	0	0	36324000	11046000	11749000
327460000	0	0	0	4952200	1455300	1745800
35894000	24296000	13673000	29461000	8579100	1109800	1391500
570130000	775760000	520980000	814400000	179440000	15761000	22555000

###########	301430000	319320000	553810000	255140000	71746000	72517000
11615000	15518000	9727500	18336000	8502300	1106300	1263000
313240000	0	0	0	13099000	3872900	4813900
171650000	0	0	0	29909000	8226700	10239000
7607800	0	0	0	272780	66439	97663
57890000	92676000	46069000	33743000	65184000	9067500	10041000
3934400	0	0	0	252850	80278	66237
8251800	8344200	4542300	7916100	8877500	1369200	1697300
87297000	0	0	0	4958100	1648400	1369800
46507000	20179000	32198000	43529000	12466000	1713000	2376200
729680000	797040000	669560000	1025200000	64160000	8216200	9255400
255240000	151350000	92867000	233140000	62060000	11363000	7602800
186040000	40441000	24540000	64162000	65899000	14713000	16166000
8683900	0	0	0	476220	140410	165530
18157000	34705000	17884000	21235000	9274600	1034500	1164800
0	33752000	8588500	3874400	11554000	0	0
12841000	0	0	0	5821500	1888600	1792800
0	21429000	6039000	0	2075800	348630	353750
105980000	55842000	42815000	52793000	89820000	18559000	19774000
285100000	43295000	29031000	28463000	149060000	34801000	49948000
297000000	24248000	36394000	39399000	56347000	13508000	18024000
47509000	3988300	2984800	4729200	7582100	1886800	2405800
381660000	102720000	76195000	120820000	137520000	27907000	33902000
229750000	35593000	28959000	26242000	164400000	35239000	49029000
295530000	14143000	44926000	49871000	101110000	25090000	31080000
18537000	6433200	5296900	7562900	36032000	7257900	9858400
469120000	134590000	100860000	125680000	154130000	29239000	41861000
0	33206000	5360900	2446200	10253000	0	0
60438000	39115000	20459000	34304000	61139000	9357100	13203000
145960000	79167000	38997000	43264000	75321000	13629000	17779000
89525000	124360000	60545000	58154000	77171000	9546600	12194000
849970000	140680000	180540000	237180000	281940000	63835000	77267000
507140000	377510000	316190000	389040000	184300000	26273000	35729000
219970000	46531000	50473000	59587000	117210000	22640000	31809000
69081000	0	0	0	32650000	5929700	12904000
0	0	0	0	6245800	3174800	3071000
37388000	0	6740900	10381000	100290000	18472000	27306000
382550000	123300000	92795000	147400000	134140000	25302000	34233000
383870000	104810000	79636000	112680000	129590000	27067000	34428000
522020000	106950000	77287000	98579000	158510000	35448000	42577000
49292000	20887000	20020000	23481000	32284000	4509400	8828000
158450000	75644000	70556000	99642000	35258000	7071700	6907100
22508000	0	0	0	2924300	996710	989730
215530000	93449000	63490000	106760000	177760000	43961000	37950000
15797000	28418000	3811200	5719300	16095000	2884700	2461500

#######################		760680000	977080000	387080000	75290000	73371000
114320000	3441600	1988100	4851900	23045000	5417800	8043000
6119400000	1876400000	1329900000	1761400000	981750000	228070000	225720000
4854000000	123840000	112460000	186620000	656460000	202290000	202890000
8350100000	297710000	216790000	389260000	1131400000	347290000	321420000
16500000	0	0	0	4724500	1385400	1505700
81692000	18396000	9014600	22963000	18146000	5042900	4299200
45926000	4135700	3377100	5325600	10831000	3033400	3276900
11019000	0	0	0	1276600	378950	438580
7736400000	1452300000	1258100000	1982400000	114200000	27720000	29982000
0	11729000	4721800	5163600	1662700	0	0
91795000	173690000	84905000	77624000	161540000	31281000	44659000
0	57104000	18054000	31600000	8896500	0	0
1629000000	932260000	620390000	848000000	153760000	18315000	26532000
921870000	620210000	361470000	893210000	380180000	73353000	52573000
105220000	0	0	0	12562000	3389400	3634800
478640000	21406000	12543000	17249000	77812000	22180000	26196000
16971000	18771000	7402000	10327000	6512600	1048100	1351300
165260000	47371000	30573000	36820000	9948600	2086400	2770800
57111000	64897000	31583000	17591000	6367700	1209500	983020
427940000	176560000	135260000	244500000	18696000	3472200	3779200
393170000	10421000	8920600	13330000	6725900	1807800	2256600
11644000	20454000	18256000	25792000	11500000	1085900	895960
79740000	0	0	0	9348700	2971700	2579800
109680000	27056000	16170000	33722000	13526000	2480700	3580300
34600000	19366000	16283000	33079000	8367100	1440300	1488500
1,7054E+10	44366000	35585000	52938000	2952000000	774200000	855750000
1419100000	1149700000	892430000	1326600000	483870000	79286000	85392000
0	1309400000	653370	467640	655240000	0	0
123250000	153420000	52801000	104850000	9682800	1104400	1458500
26984000	20113000	12032000	8506200	2196500	226210	467330
585870000	166400000	114620000	172170000	141320000	30155000	36944000
91800000	11394000	6231000	8317800	812620	236220	263250
152080000	29868000	22989000	42956000	4773100	1200100	1319500
274830000	0	0	0	5117900	1378100	1790700
11185000	8130200	8468000	15925000	1639000	211000	246720
43577000	7946900	6237600	9656200	1556500	367560	448060
122930000	0	0	0	38169000	11814000	12696000
116650000	92922000	45115000	50673000	31248000	3514400	4244600
353370000	0	0	0	31184000	9872100	10270000
115090000	209290000	172090000	234040000	20802000	2243600	2678300
301390000	337260000	268650000	326340000	21973000	2574200	3167100
137500000	0	0	0	19350000	6221100	6254100
98815000	0	0	0	1566100	216060	633980
140210000	43008000	40640000	51881000	19837000	4244100	4563400

44429000	38365000	26959000	61891000	16433000	2258200	2732100
0	35430000	27829000	35316000	1564700	0	0
93209000	0	0	0	20232000	6264400	7309400
200820000	5020500	7341300	4402400	16250000	4383900	5272100
17268000	0	0	0	2486700	838990	784300
137590000	198990000	141580000	170830000	22232000	1984800	2707100
87050000	0	0	0	17299000	4543100	6538100
12937000	30599000	18868000	34426000	3675600	283210	268810
40999000	28852000	30871000	50535000	20229000	4052300	4541500
19974000	0	0	0	1119400	314550	388720
2477200000	583980000	508010000	782280000	345390000	81388000	82688000
0	41571000	0	0	1539700	0	0
417580000	115310000	97941000	152860000	14290000	2945700	3347000
4871300	0	0	0	128590	39345	45753
82878000	129680000	104860000	150440000	6949000	543430	965300
12487000	0	0	0	1461500	418910	474980
341450000	18901000	20500000	28876000	13739000	3643900	4404200
611880000	784100000	591700000	891940000	116960000	13454000	16250000
59446000	29490000	25172000	34305000	2311600	210260	539060
7376000	0	2951900	2992800	458890	73834	123870
1,2804E+11	0	0	0	1,3348E+10	4004200000	4771100000
294580000	206880000	159400000	257410000	37296000	5869100	6609000
30255000	0	0	0	1117300	335570	301440
349570000	41358000	26562000	55527000	11918000	2940200	3891400
33508000	0	5997200	0	620520	29335	256400
18604000	7209600	3862500	9429600	3904300	706980	1024800
3498400	0	0	0	106810	26216	44900
11646000	7252600	8600200	9894400	1581500	247080	323740
86260000	0	0	0	14192000	4144100	4973800
99873000	63804000	0	0	1296300	367940	363950
1447100000	1125500000	824340000	1107900000	421780000	78275000	78525000
8525000	4975800	2878200	5155800	1864800	376680	462640
586460000	838880000	670690000	925140000	249470000	27399000	33248000
340720000	22961000	51229000	85456000	66158000	15220000	19665000
65292000	39202000	38963000	54413000	11780000	1722500	2142300
97561000	8323000	4657600	11802000	7635700	1812900	2424400
47435000	0	0	0	1526000	458610	481750
57579000	74642000	38326000	76523000	20599000	2157000	3000000
1619800000	0	0	0	65238000	20292000	20025000
60759000	78694000	60130000	76479000	10261000	1184000	1408300
0	1,341E+10	0	0	3352400000	0	0
433080000	0	0	0	62448000	21775000	19019000
4276300	13871000	12051000	16749000	1494200	71527	0
0	168490000	0	0	18722000	0	0
6173600	0	0	0	455890	111720	151240

10921000	8288800	7187500	12772000	3580400	517120	615220
5824600	0	0	0	1059400	366880	368890
103210000	5833400	5246500	6763900	13681000	3474600	4441800
27496000	0	0	0	4275100	1233100	1323500
78350000	10356000	12111000	20710000	8695300	2212600	2292200
135510000	29744000	17582000	28857000	8783900	2296000	2167600
25947000	31555000	29996000	35278000	2499200	364660	405260
6488100	0	0	0	688730	247270	270720
1146900000	4858300000	3097000000	7662000000	1857500000	102170000	78940000
530180000	110860000	86017000	188730000	61435000	13390000	16466000
132410000	0	0	0	7978600	2345700	2553600
0	1954300	0	2253000	467470	0	0
8028500	0	0	0	432300	149760	171030
68309000	0	0	0	5356600	1609100	1795800
34402000	28481000	27630000	40968000	19284000	3048100	3087800
24922000	0	0	0	2384700	716110	745540
47704000	0	0	0	1723700	536770	590600
0	47758000	62075000	57121000	9820800	0	0
187700000	0	0	0	10148000	2963900	3503500
342130000	6129000	16209000	19078000	33787000	9742000	9293300
179740000	112920000	104280000	160250000	41416000	7283800	6271900
492630000	21238000	22051000	30592000	49321000	12261000	16078000
26758000	0	0	0	2091300	628350	739800
0	6918000	5955700	9009200	1458900	0	0
15836000	0	0	0	992890	265140	390820
10017000	0	0	0	628340	175680	214170
248850000	39194000	49514000	37883000	22215000	4276400	2921500
40522000	17807000	15369000	8263500	8928900	897610	580180
0	0	0	3503000	318460	0	0
0	9278900	5781300	9969300	521450	0	0
342540000	276740000	164800000	246200000	257780000	49719000	60883000
47861000	3599700	5772100	8311600	2431900	716460	779120
44800000	0	0	0	1075200	317130	368540
1819700000	161640000	147910000	212100000	48050000	12009000	14561000
53671000	107660000	96385000	131630000	27370000	1554900	2912300
80336000	0	0	0	1845000	540080	532450
7549500	0	0	0	592940	191830	165190
146440000	0	0	9532900	3601600	1065900	1117800
160370000	0	0	0	6114100	1889600	1965800
19565000	63022000	52039000	73996000	10452000	613270	768040
103370000	0	0	0	6114800	1192700	2337800
48033000	0	0	0	1034400	265860	327820
25640000	0	0	0	1628200	452930	592520
1766700000	689490000	514010000	755440000	156400000	29333000	36201000
122660000	5051100	4754500	6419900	5046100	1363500	1609700

944350000	3837200	2454700	4107600	52403000	15704000	16385000
36848000	0	0	0	4896900	1514300	1780500
274020000	22123000	34193000	66561000	26848000	8134100	1457700
0	4923000	4899600	5961800	657680	0	0
62546000	0	0	0	4710700	1379900	1543800
120690000	0	0	0	10533000	3975300	3008000
77651000	0	0	0	2912700	879250	760470
176710000	89505000	59549000	75706000	5259300	1068300	1029800
52464000	0	0	0	2943900	840820	1032400
35046000	0	0	0	3932100	1166600	1363600
59092000	0	0	0	7148100	1749300	2712700
6020100	0	0	0	168330	55179	54703
151760000	4131100	2839700	3544400	21337000	4943300	9632000
41991000	38580000	19685000	21653000	6102300	1146100	1146500
26113000	0	0	0	5291100	0	2389600
0	295630000	252370000	252400000	57172000	0	0
35428000	18704000	14894000	22860000	7653600	1732100	1546000
7584900	0	0	0	3419400	1320200	1467100
0	26733000	13363000	31610000	2868300	0	0
17846000	8741800	6462200	9361100	5177000	1076700	1071000
118230000	0	0	0	4753800	1505400	1770600
98337000	2236100	1621300	2875000	2008300	553340	704450
13632000	39508000	0	0	1235500	170930	98407
337540000	0	0	0	13585000	4141300	4480100
39650000	17513000	13345000	17485000	1843100	378440	453230
7779900	10412000	9420900	27197000	908290	72542	114570
1959600	0	0	0	221060	95337	115820
9334800	0	0	0	1637800	452730	466990
0	41638000	1305400	0	1226900	0	0
5764600	0	0	0	485180	127520	182980
10025000	0	0	0	1617300	427970	562770
72602000	24504000	14696000	24800000	18583000	4713900	2486100
232880000	19739000	12974000	20657000	8177600	2182800	2546000
47753000	129990000	257660000	128870000	56531000	4391700	5116800
272050000	139720000	63332000	65096000	48236000	1557500	5124500
102700000	77127000	45198000	121640000	44496000	7907500	5073800
41779000	0	0	0	2018600	578800	754930
0	8808000	8774200	11657000	1538900	0	0
269960000	38213000	31586000	40720000	24630000	6241600	7197700
2059300	0	0	0	399670	131760	164940
20628000	0	0	0	730640	215780	257000
2939700000	2228400000	1653400000	2352300000	422910000	73537000	71372000
14145000	0	0	0	5237300	1282000	1934700
67918000	13522000	5613700	19628000	5422700	957510	1327400
9948400	0	2596100	3260200	845680	191970	237810

155720000	0	0	0	45203000	12581000	15319000
109970000	0	0	0	1719000	461170	610920
0	21905000	0	0	4380900	0	0
85812000	8042800	0	6673300	4562800	1257800	1333800
42995000	25424000	13780000	30246000	6918100	1320200	1581900
72476000	4731400	10927000	19645000	16786000	4502600	5943900
6987400	0	0	0	2243700	671580	795730
33985000	0	0	0	3246800	1011500	1102400
803900000	13158000	12579000	19603000	334840000	96464000	117060000
11948000	0	0	0	993330	277930	363980
5066700	6126800	6828000	5767700	3176500	943470	1281400
14670000	22015000	13828000	20669000	7603000	730410	1788200
4756500	22699000	12230000	20517000	2867500	125800	233280
6494900	0	0	0	454560	119840	187100
30144000	5242700	4561600	4260700	1991800	520010	569570
5366400	4944600	3168700	7577800	1290900	215810	232770
75583000	93270000	75797000	91679000	25181000	3070000	3426400
30625000	0	0	0	2235500	553900	896300
63393000	241710000	24385000	249710000	16980000	1136700	1363900
7564600000	0	0	0	768060000	233570000	254310000
147750000	93514000	53517000	124930000	48389000	9000900	7103200
10056000	0	0	0	1053600	262130	432290
0	2021800	429410	3312500	44337	0	0
419120000	0	0	0	9153000	2748600	2996900
155130000	0	0	0	3227200	925370	1099300
26737000	21552000	22716000	36820000	3223600	366360	513180
186480000	135680000	131950000	214320000	47432000	6010900	7999700
216630000	54026000	0	69859000	2970100	848480	759510
9684500	0	0	0	326300	112300	88220
24714000	2849700	2615700	4477000	2142300	471260	588020
61360000	0	0	0	8875200	2660600	2805700
54960000	0	0	0	10236000	2833400	3477100
353830000	340130000	242650000	392050000	31336000	4694300	5551600
55258000	12339000	9000500	20626000	6906500	1508300	1926000
3874200000	67633000	62391000	75844000	101120000	29319000	34027000
5676600	8585200	7625400	10540000	2031300	211100	198840
69137000	71241000	106650000	61352000	36991000	2051000	6905500
361770000	112890000	97426000	187150000	13564000	2766300	3050900
254500000	29238000	22030000	34474000	39677000	11545000	11119000
44053000	40085000	41417000	44051000	3225400	430320	471740
2201600000	2880900000	2223500000	3430100000	410790000	47387000	56656000
23804000	0	0	0	543770	153380	165830
11764000	7402100	6081500	7495600	6239900	1202900	1398900
6794400	0	0	0	1166900	352010	329570
5749500	3875600	3010200	4308100	983200	195650	203290

14005000	0	0	0	2570700	799720	770620
25341000	0	0	0	1589900	471340	515210
19311000	0	0	0	7290200	2237100	2639200
134480000	145400000	139030000	183880000	14862000	1752100	1736700
0	50405000	17321000	34624000	17058000	0	0
48578000	19587000	22035000	25060000	10153000	2598800	2065300
187330000	292910000	223920000	312700000	64325000	7458500	8444500
41402000	80763000	66346000	104250000	25883000	2283900	2687900
75788000	0	0	0	5913700	1656200	2209200
147880000	0	0	0	19978000	5729900	6854400
13517000	0	0	0	956900	247140	395400
7932300	7694300	5529000	7042000	3646500	609680	687010
65076000	16253000	8791000	21630000	3402300	897400	672970
24154000	0	0	0	3414500	1127200	866530
25834000	6492000	3745300	6863000	2235400	466850	638680
143920000	0	0	0	3317700	1059900	1058500
16691000	0	0	0	2045700	572420	678500
1570100000	313440000	271450000	471490000	229390000	51539000	63657000
0	4186900	3492600	5622200	316710	0	0
10257000	0	0	0	1927800	634100	561080
299440000	5197500	2462500	4633700	9562000	2946700	3559100
47192000	0	0	0	5062100	1459900	1787200
62562000	0	3209200	4958900	10743000	2829200	3493400
106250000	6267100	7921600	16592000	4325300	991280	929890
196280000	0	0	0	2829400	755740	958460
1,3766E+10	15107000	15250000	24710000	3066900000	1038500000	1106900000
47030000	0	0	0	3884600	1094500	1320400
7051900	0	0	0	883590	237870	274570
74492000	135190000	111950000	133400000	96943000	8997300	12107000
22208000	8435700	20076000	23527000	7516000	1154600	1411700
1038500000	451970000	462880000	461770000	63937000	12634000	16302000
99071000	20595000	116330000	125970000	17582000	3351000	3262600
297090000	373890000	393560000	490680000	134760000	14539000	16543000
200450000	232520000	168650000	296780000	72917000	9873200	6893800
430170000	45766000	46368000	68741000	11750000	2894700	3625000
13880000	14745000	0	11311000	7305800	991540	1322300
214660000	221610000	188320000	237270000	79523000	9562500	12504000
163890000	0	0	0	22353000	6870000	7288600
319440000	18479000	13441000	19473000	55012000	18089000	18381000
744020000	0	0	0	32709000	1420300	12688000
33756000	0	0	0	2384300	743070	856160
105560000	70754000	50315000	62530000	25290000	4425400	4800600
34562000	7190700	6467900	7070400	20956000	4225300	5672500
39222000	4645600	16742000	17821000	16844000	3412500	4716700
0	8879800	7032900	13542000	1732600	0	0

183090000	0	0	0	4332100	1210100	1487300
15677000	32507000	30646000	48932000	7770900	609150	773600
28011000	0	0	0	4748700	1453000	1545000
39380000	0	0	0	19020000	5637400	6819300
15135000	3281800	3365900	2810700	2239300	305490	704130
452760000	0	0	0	11082000	3133400	3977000
136480000	0	0	0	4905900	1370300	1639900
38071000	9693300	5447000	10534000	36469000	11026000	9507200
53391000	33155000	34851000	35009000	10281000	968920	1863800
136810000	10917000	5252100	13861000	5933400	1310800	1474600
4176300	14165000	18290000	21214000	2741200	111860	0
93496000	2516300	1515900	3137000	14229000	4582700	4613400
3344200	0	0	0	96009	23241	36020
134530000	108620000	75698000	107530000	126900000	19329000	22295000
205090000	5869200	4191800	11772000	27541000	7315200	9420400
21268000	0	0	0	1990400	682170	717480
14170000	2084700	0	2181900	1160300	255890	419270
1840800000	0	0	4811400	74361000	22079000	24318000
19671000	8216400	8768400	3917000	6011300	1133900	1496300
38005000	29670000	19069000	23359000	8691900	918770	1656300
176880000	575910000	537090000	899170000	51075000	2800200	2669400
75980000	31183000	56282000	64263000	35156000	5472800	6912900
2953600	0	0	0	590640	193390	170050
8037900	0	0	0	874680	268240	297290
174550000	0	0	0	18941000	5745500	6214000
1823300000	237020000	217590000	270160000	93495000	25815000	27234000
83052000	111660000	54256000	114160000	22853000	2839000	2722100
5129000	9233400	8694500	11811000	1859900	157610	186290
1625700000	54346000	46517000	58243000	223110000	63131000	74990000
14747000	0	0	0	398470	108180	131720
75770000	0	0	0	7100500	1852800	2333500
0	0	0	0	130960	55055	75901
24534000	14668000	5722200	17163000	8344000	1456700	1713400
50078000	28097000	20770000	59095000	23872000	3288600	4779500
6329300	0	0	0	411450	119220	137860
36203000	35859000	0	38018000	11991000	1697500	2430500
7676000	0	0	0	623600	143100	196200
1777900000	1669700000	1037200000	1401800000	203920000	27530000	29224000
240500000	0	0	0	3983400	1528900	711720
4424500	0	0	0	255120	74766	84167
20703000	0	0	0	4845800	1341300	1622400
374080000	0	0	0	3317300	998020	992740
43024000	92201000	114810000	144020000	25608000	1312700	2403300
57742000	15789000	10849000	12485000	15792000	1040000	3989300
11261000	17074000	12291000	38458000	3125100	298680	275290

34727000	0	0	0	2929500	715660	1016400
135820000	37268000	41218000	32298000	8581600	1107900	1602100
4813800	5047400	0	3455100	896640	230820	0
0	37113000	28932000	32990000	2912800	0	0
26751000	0	0	0	3770800	970950	1391900
7544100	0	0	0	1172800	352530	443080
51336000	0	0	0	5594900	1624500	1738400
77016000	0	0	0	3625400	911100	1233200
747640000	414460000	240120000	407080000	84942000	14686000	18562000
4257500	0	0	0	1567400	531440	752110
25914000	5016500	4093300	5430600	1498200	347690	440750
7481600	42595000	49521000	20458000	14795000	767030	688170
263220000	0	4668000	5389000	22725000	6523800	7661800
50149000	25290000	15508000	24350000	5053100	946870	1072100
22043000	0	0	0	1702800	499050	535750
9334100	0	0	0	2550900	791950	1175600
96612000	50695000	59222000	73393000	56711000	10919000	10802000
22707000	0	0	0	1504600	414860	522060
82535000	47268000	25086000	66504000	11149000	1979700	2027600
0	4630600	0	0	55126	0	0
12577000	0	0	0	537720	147520	193690
0	7162400	5531400	8806000	693540	0	0
144750000	23519000	16611000	25602000	6552600	1719400	2099600
376740000	0	348960	2500100	10629000	3037700	3373400
82965000	0	0	0	3571500	1008400	1180300
71583000	14075000	9881100	13737000	3862600	1022200	853620
93428000	0	0	0	6024000	1705900	1982400
371870000	0	0	0	44121000	13757000	14869000
554430000	217440000	76722000	191630000	97728000	20300000	22679000
718570000	782950000	544080000	841640000	33600000	4332100	5207700
767330000	87791000	68988000	152040000	89746000	24623000	25265000
0	0	0	0	0	0	0
79823000	0	0	0	2023000	687070	589880
29233000	0	0	0	2021000	651190	620290
433020000	293000000	169180000	453000000	69022000	10778000	10094000
179630000	89873000	65782000	153210000	36198000	7152100	7806900
865010000	503170000	390060000	754180000	91282000	12445000	19018000
945500000	130080000	100480000	154040000	125340000	32089000	37831000
1632700000	0	0	0	128990000	39482000	40030000
1049100000	648380000	486930000	718740000	250460000	41094000	48074000
6385300	0	0	0	1535900	452750	502650
69847000	248480000	196740000	265780000	59245000	3237700	3950200
15102000	7427400	4097500	8577200	8189700	1884500	1904700
13146000	6125200	7831400	10124000	2527900	409960	566830
114030000	25741000	18656000	26496000	119720000	25402000	32678000

47396000	33836000	25009000	37880000	25005000	2852500	4137800
48219000	109750000	64445000	107560000	9574800	1093100	1308300
299660000	0	0	0	7067100	2102600	2240300
119180000	179500000	103170000	104310000	13231000	1682100	2175300
491770000	25139000	19999000	32593000	36384000	10947000	11200000
518940000	97195000	100080000	160050000	33012000	7335700	9449100
43645000	17410000	40568000	6890700	7885200	2042100	2342600
65854000	9390000	8613700	10741000	3758700	537660	1071100
15330000	9129900	0	0	2038300	0	0
222650000	262040000	166590000	248570000	NaN	NaN	NaN
0	0	0	33688000	NaN	NaN	NaN
31831000	0	0	0	NaN	NaN	NaN
0	24723000	41881000	27089000	NaN	NaN	NaN
6963900	21444000	15669000	17402000	NaN	NaN	NaN
55843000	70615000	27724000	0	NaN	NaN	NaN
0	185670000	361620000	163740000	NaN	NaN	NaN
7807200	0	0	0	NaN	NaN	NaN
277300000	324180000	426390000	0	NaN	NaN	NaN
0	0	133280000	0	NaN	NaN	NaN
117390000	0	0	0	NaN	NaN	NaN
0	0	0	0	NaN	NaN	NaN
0	5262600000	4958600000	0	NaN	NaN	NaN
15283000	0	0	0	NaN	NaN	NaN
7963800000	6855500000	8603000000	7688600000	NaN	NaN	NaN
879040000	474880000	199650000	604770000	NaN	NaN	NaN
133340000	79372000	68138000	73723000	NaN	NaN	NaN
46077000	0	0	0	13198000	2037200	1945100
191740000	168270000	120770000	181080000	14010000	1798600	1869800
134060000	72896000	42309000	59515000	40924000	7629100	7563700

iBAQ CBX2_3	iBAQ WT1	iBAQ WT2	iBAQ WT3	LFQ intensity	LFQ intensity	LFQ intensity
8417000	1679300	2109200	4035500	28530000	30923000	28971000
4279400	1727500	1042500	3016200	30723000	28971000	25943000
5218400	485910	127120	278410	6793000	8657200	31313000
1727500	7208400	5578000	8520800	94763000	80020000	83112000
2826200	2274700	1101300	2112100	44056000	39040000	43064000
545370	145450	185350	378440	8152500	8328800	8686800
433330	0	0	0	0	0	3566200
125330000	29711000	15251000	42293000	368670000	529030000	453360000
3506800	3570500	2382900	4075900	47840000	56990000	56509000
1124600	0	0	0	12733000	0	4588800
635730	797070	1010200	1500300	23255000	19797000	17653000
702880	953780	777200	1122300	0	0	0
8842300	3226500	1719400	3725500	50981000	44807000	74191000
90484	71243	33812	58227	0	0	0
2498200	0	0	0	39099000	31411000	25909000
236160	0	0	0	0	0	2296900
6664400	2443600	2116800	5002500	86815000	76971000	77384000
0	0	0	0	16911000	0	0
6005000	484230	502280	769040	308960000	325100000	271390000
1816300	400980	201510	260990	33303000	38228000	39314000
143030000	53966000	81794000	92935000	1006300000	930860000	667440000
2942300	1198100	775440	2697200	0	0	0
8253100	8190200	6542900	10353000	368120000	343720000	339100000
1417600	0	0	0	19564000	18236000	19099000
7873200	0	0	0	182080000	196270000	171520000
1748600	596520	409440	477210	0	0	0
2621700	381420	209620	350740	33589000	40676000	43481000
991550	0	0	0	0	0	5192800
1511100	119390	235180	387220	51221000	56492000	51419000
4289100	0	0	0	79735000	83519000	72267000
25594000	242100	0	0	291840000	275970000	253790000
0	0	0	0	0	8661900	0
1896600	0	0	0	0	0	48243000
793980	536800	539070	644230	0	0	0
721060	401840	319730	458100	17521000	19859000	20379000
794060	0	0	0	0	0	9505300
4261400	1574900	1468800	2852200	65820000	51593000	54321000
1154500	0	0	0	17066000	25760000	18625000
2821900	0	0	0	0	0	8444800
2755800	722970	396910	1079400	67924000	60031000	60438000
301850	0	0	0	0	0	8130000
2786000	0	0	0	146660000	143990000	135880000
275150	394080	0	628950	0	0	0
1034300	5118400	3679300	4662800	48681000	81385000	28024000

4462700	218040	226890	436040	127500000	118870000	130510000
514450	445850	391110	716780	0	0	0
293890	551620	344110	736960	12919000	12693000	10898000
178900	0	0	0	17706000	19243000	14366000
3095500	0	0	0	66169000	67397000	59469000
977290	0	0	0	14470000	15181000	13041000
357960	0	0	0	0	0	4285000
172270	481120	224980	350260	0	0	0
2057200	1506800	462150	1060500	45075000	42689000	46504000
1995700	0	0	0	50075000	44574000	47758000
15244000	4379500	3912400	3342900	119960000	113700000	113090000
1169300	109210	0	132630	36852000	0	23053000
7252000	2141700	1447200	2479100	94999000	87397000	85524000
1420500	0	0	0	0	0	4251200
0	36449000	0	170780	0	0	0
207240	0	0	35784	0	0	0
968760	3371900	3400200	4339200	70310000	0	73329000
6433600	4160300	1685900	2803900	36417000	43026000	36495000
1797200	0	0	0	0	0	6722800
137650000	33310000	21647000	35889000	538780000	626830000	632430000
173270000	0	0	0	1947900000	1553500000	1656700000
512700	370170	140010	397200	29991000	34548000	22209000
7244600	6548000	0	6704600	0	0	0
896380	0	0	0	8766100	0	6708500
6790700	0	0	0	41788000	43348000	40706000
98004000	91041000	57359000	109410000	1610600000	1571300000	1601300000
1043400	0	0	0	23186000	25538000	20938000
1351100	672040	42534000	844090	746330000	720430000	743700000
25493000	4660400	4050400	6462300	117800000	138380000	134550000
1343400	0	0	0	0	0	6030600
349110	0	0	0	0	0	5485000
981760	0	0	0	0	0	15425000
1555100	0	0	0	112220000	105590000	94775000
40586000	9425400	8499600	11817000	187170000	207340000	199720000
1091000	113770	60746	112000	28974000	28935000	29551000
4247700	0	0	0	0	0	12712000
5272700	2344100	1052500	1650800	47457000	40967000	45420000
2191800	2838800	1668200	3059600	42346000	37470000	38494000
365000	0	0	0	12922000	12251000	10696000
40976000	14539000	8989800	11924000	255060000	305830000	349570000
751160	0	0	0	12686000	10751000	11598000
1652800	103580	87514	150220	49159000	48629000	43536000
3630500	419680	356720	0	57049000	53335000	66551000
359430	0	0	0	8943700	15191000	8493800
37459000	1491500	0	1835000	87598000	109680000	112020000

0	1393900	1106400	1971700	0	0	0
6212900	2642100	1590100	4595900	48322000	59714000	53972000
4554500	1880400	378820	3606400	24165000	25393000	27885000
4219100	1569200	4245500	8454400	0	0	0
450120	0	0	0	54683000	59216000	56241000
576320	533820	412210	613180	0	0	0
7197700	8132600	4732100	7456800	271270000	278550000	295750000
351510	0	0	0	0	0	5785600
997690	1831400	1195100	1100900	20916000	26150000	24286000
6803400	0	0	0	56894000	67829000	57270000
458700	0	0	0	0	8504300	0
9509300	662160	327010	534230	224300000	199770000	200570000
3733700	6753600	4306500	6187400	0	24696000	0
1684200	5272800	4660500	5903400	25221000	17977000	16064000
800140	1235200	977440	1381700	31689000	34078000	29913000
6987100	0	0	0	428310000	386340000	386520000
368920	0	0	0	7884700	7727700	8593900
7971100	10808000	4109800	14038000	109950000	131130000	181970000
12699000	5045200	4140700	6107600	150360000	141370000	127580000
0	1087800	196600	212650	0	0	0
2517800	0	0	0	34373000	25378000	30133000
9675800	1334100	288670	635360	62293000	64579000	74503000
37469000	6828000	6694900	13381000	240270000	256490000	264150000
846390	945200	488790	600320	14372000	0	0
1774000	0	0	0	34658000	43610000	39561000
546190	0	0	0	15070000	13786000	12241000
1268300	533810	708880	893350	25672000	27048000	21432000
9731300	2345000	1641600	3118600	232180000	235890000	182900000
5906300	1595200	1325200	1998900	160530000	155370000	153360000
29600000	17444000	14733000	23058000	423020000	443410000	430340000
1764000	0	0	0	25224000	29368000	25164000
285670	307230	264800	240770	13076000	11796000	0
823470	0	0	0	12815000	0	17413000
656670	0	0	0	0	0	3930300
164880	0	0	0	0	8176000	0
2533600	0	0	0	32287000	26596000	23839000
2439200	0	0	0	0	0	5474700
560560	530910	317140	514840	0	0	0
114470000	16816000	14443000	21819000	9249300000	9703700000	9179100000
0	383910	288450	493840	0	0	0
2564300	0	0	0	70668000	70274000	54305000
10552000	5475900	4135800	6585400	0	0	0
1846000	0	0	0	0	0	5524300
37685000	18610000	12296000	16832000	643600000	623030000	627300000
0	354090	269520	0	0	5059400	0

11262000	6345200	1935200	6984900	0	0	0
5283300	675190	606140	676070	164220000	154210000	137040000
134860000	78817000	60945000	89928000	470370000	486270000	520210000
2013000	2239500	3678500	7877100	0	35256000	0
475920	0	0	0	11643000	11708000	11916000
782600	566320	492980	632690	0	0	0
481540	0	0	0	13343000	0	0
14722000	0	0	0	285950000	287580000	243170000
17005000	0	0	0	0	0	38168000
1919200	3772400	1829800	3122900	0	0	0
13099000	0	0	0	80600000	91836000	77709000
2681800	0	0	0	39034000	41026000	37534000
511510	144960	101480	115870	13126000	12797000	13889000
2394500	0	0	0	39776000	44599000	32008000
446380	1230800	925040	1205600	0	0	0
1069100	0	0	0	0	0	5599000
8918200	12794000	5904800	9128100	233400000	290240000	291400000
640140	151850	0	146850	13800000	13199000	12435000
3384300	2186700	1318600	2557200	189840000	187750000	174300000
18485000	807560	1027900	1210200	534230000	723990000	630690000
1458900	194820	292080	320480	25717000	28934000	25375000
342780	215080	0	250620	0	0	0
1457800	326430	425760	567820	29567000	35332000	28888000
188810000	66976000	45782000	89842000	2022100000	1909900000	1830000000
474980	0	0	0	0	0	3553600
756780	0	0	0	28213000	29521000	27820000
1706700	0	0	0	157500000	177980000	158760000
2764500	0	0	1530800	0	0	8273100
33976000	8795600	3286700	8929400	222200000	258080000	265450000
1984700	840260	428700	847780	0	0	0
117950	0	0	0	9774900	11293000	10748000
818230	0	0	0	10106000	10985000	9776700
27232000	3885100	3341000	4429300	87464000	99524000	100010000
1697700	0	0	0	45638000	49609000	47632000
0	89107	53236	62847	0	0	0
2260800	861810	0	691610	10462000	10462000	14115000
852450	0	0	0	0	0	5739900
3526700	643270	178250	649550	80908000	77775000	69925000
1948600	0	0	0	76437000	75767000	71246000
911790	181770	186090	284090	22373000	25716000	25717000
1669100	506580	720220	1157800	0	13219000	12634000
1336700	570360	431400	688040	0	0	13001000
108510000	18478000	17663000	36948000	206870000	199510000	210780000
0	42489000	12548000	10398000	0	0	0
11721000	9713000	6823000	10844000	301990000	315360000	287130000

63592000	73285000	64914000	84411000	287990000	361620000	345860000
703890	1960600	1511700	2042700	59352000	76216000	60958000
9051500	6618800	3905100	6999300	167890000	163320000	161450000
1614000	0	0	0	13741000	12890000	12076000
0	1051600	0	0	0	0	0
2610000	0	0	0	56386000	56337000	54132000
8085800	1514900	547810	1111400	121730000	132540000	132610000
833010000	433840000	373510000	584500000	4382900000	3880700000	3654000000
10072000	916640	615970	838520	42536000	40793000	37886000
430970	0	0	0	24046000	22442000	23039000
3226900	353440	201810	334490	60849000	41482000	42268000
799300	0	0	0	0	11454000	9813400
6596300	0	0	0	89645000	77031000	74149000
820260	0	0	0	0	0	2454700
2497700	20299	21977	38590	172470000	169260000	181740000
468580	0	0	0	0	0	3155100
5769600	86577	170520	246140	152920000	97107000	134810000
5678800	0	0	0	30998000	27501000	27894000
573280	0	0	0	11861000	11256000	11745000
8802700	1011400	820280	1023300	301800000	361250000	345410000
4012300	158000	0	0	74705000	78812000	69459000
54222000	67391000	29785000	36973000	263210000	324340000	696520000
223410	0	0	0	11436000	11208000	10563000
73054000	78106000	45647000	96600000	1601800000	1766100000	1713600000
1187100	0	0	0	27818000	20622000	20380000
5513600	3710300	2392000	4202500	118290000	114260000	135990000
0	436570	304600	537980	0	0	0
288380000	106010000	70060000	141030000	1331800000	1030200000	817450000
5173800	10084000	9087900	14559000	118730000	102910000	125080000
9066200	691240	1113200	1666000	163700000	142900000	123570000
51497000	74816000	29289000	32014000	263390000	361560000	803670000
5083100	4070200	3778500	6639700	66890000	67300000	68301000
11337000	4939700	4018100	5750400	60578000	63587000	59750000
4996800	2307900	1898500	2843200	211220000	203210000	205110000
0	379960	278440	702760	0	0	0
2605700	1691800	1218400	2014900	0	0	0
1020900	848120	611340	686580	12311000	11910000	9553900
549570000	58888000	49738000	68633000	4924200000	5475700000	4765800000
290400	355880	345730	394440	13523000	18282000	16606000
3323000	2581700	1685100	2458700	46138000	53163000	51687000
37662000	96811000	5881400	6841300	1070800000	829680000	871270000
0	215340	153740	152240	0	0	0
2329500	2120000	640300	1774700	30013000	34727000	32609000
618180	243650	148470	177480	0	0	0
41271000	11941000	4782500	8621000	319760000	365260000	374900000

17192000	9022100	4898500	10521000	226060000	231220000	205240000
0	708420	673820	0	15352000	16861000	0
821440	86711	242970	286700	0	0	6145700
5612900	0	0	477530	61069000	52318000	55477000
1139900	0	0	0	93240000	93234000	86663000
2795600	424950	168820	0	50918000	44076000	45604000
2251600	3200900	2634000	3348200	0	0	0
394680	0	271730	382520	0	0	0
1086600	0	0	0	15273000	16058000	15775000
0	443130	336120	618700	0	0	0
3573300	225000	0	216440	95852000	95161000	94977000
163710	0	0	0	0	0	7226400
0	5389700	2352900	4322100	0	0	0
1408700	0	0	0	23127000	21526000	22863000
373150	0	0	0	0	63676000	0
4137800	0	0	0	71080000	78136000	65265000
3724300	2313200	1570400	1812800	22777000	23314000	20237000
3696400	2755600	948490	2004800	46572000	49834000	49446000
623480	0	0	0	7155600	7716200	6753200
35306000	93740	16473	124790	2004800000	922940000	940380000
2454200	0	0	0	63947000	60917000	59565000
3284800	279660	230470	294870	90833000	91137000	84018000
2451800	3144700	1415300	2877700	0	0	0
1314200	0	0	0	0	0	7866000
2790000	0	0	0	0	0	16699000
1167200	0	0	0	0	0	5239700
351230	422040	556680	754450	0	0	0
1155300	0	0	0	0	0	9508000
457470	0	0	0	39429000	39327000	42803000
150310	0	0	0	0	0	4498100
413420	0	0	0	0	0	5258100
1345900	0	0	0	14675000	19262000	11071000
0	1593600	1446100	2039100	0	0	0
34434000	19986000	7105900	15246000	306590000	285190000	325420000
1696900	1431300	0	1271900	16277000	13544000	16209000
3303300	5786800	1519200	5768800	0	0	0
1087500	1549900	720910	1299400	0	0	8949400
1317900	0	0	0	26360000	0	20491000
580020	0	0	0	12111000	10286000	10486000
689640	0	0	0	0	0	7739300
10897000	968660	741450	1161600	113940000	117140000	186000000
0	9420500	2586400	17551000	0	0	0
3669500	0	0	0	180480000	203710000	167240000
294380	0	0	0	0	0	5506100
14819000	17871000	15698000	16822000	171160000	200210000	182460000

648260	0	0	0	16699000	18207000	16714000

;R4KXX0;Q9TQP8;Q9MW39;Q9GIP0;Q9BCN8;Q95IZ1;Q95IG6;Q8SNC6;Q8MHP8;Q8MHP7;Q8MGZ8;Q8HWT0;Q75WB6;Q7
162;F8R156;F8R155;F8R154;F8R153;F8R151;F8R149;F8R146;F8R145;F8R144;F8R143;F8R142;F8R141;F8R140;F8R139;F8R
4L6P2;R4L0K0;R4KXX0;Q9TQP8;Q9MW39;Q9GIP0;Q9BCN8;Q95IZ1;Q95IG6;Q8SNC6;Q8MHP8;Q8MHP7;Q8MGZ8;Q8HWT0
162;F8R156;F8R155;F8R154;F8R153;F8R151;F8R149;F8R146;F8R145;F8R144;F8R143;F8R142;F8R141;F8R140;F8R139;F8R
73;273;273;273;273;273;273;273;273;273;273;273;273;273;273;273;273;273;273;273;273;273;273;273;273;273;273;

4502100	4572800	1634500	2294300	55854000	59931000	52691000
2165100	0	0	0	119420000	125100000	111250000
0	0	0	0	3145100	0	0
204760	0	0	0	0	0	8885300
3040100	0	0	0	0	0	6823400
21688	0	0	0	0	0	4413500
891110	429990	317190	1136400	14464000	17204000	16850000
4005700	0	0	0	32078000	29026000	27462000
0	2398500	0	0	0	0	0
7346300	0	1824400	2478900	0	0	0
8627400	5674000	4664000	6729200	248230000	271080000	251540000
3484700	0	0	0	0	0	7821300
1049600	311800	153150	363310	0	13863000	0
155040	0	0	0	8920100	9059100	7122300
11254000	506430	357230	593730	56891000	62303000	60972000
368030	0	0	0	0	0	4130100
10193000	0	0	0	97224000	92718000	87258000
16347000	0	0	0	57497000	45143000	66868000
4625700	852350	1036600	730580	43702000	52375000	43851000
47929000	32464000	25060000	35744000	5791300000	6560600000	6173200000
452680	4098700	1829700	2882900	11685000	14503000	17370000
663140	0	0	0	0	0	2976800
1726600	1118500	1326000	2139600	46938000	53342000	53139000
1764100	0	0	0	33400000	34688000	30448000
603280	0	0	0	18627000	18178000	17360000
87046	0	0	0	0	0	12504000
732050	0	0	0	19625000	22599000	20430000
107570	0	0	0	0	0	4828600
324040	0	0	0	0	0	2424300
33251000	40495000	15426000	54706000	152690000	107070000	104090000
443550	0	0	0	32752000	30626000	30863000
0	4816300	3464000	5734200	0	0	0
175940	329930	212000	324440	0	0	0
370630	0	0	0	0	0	4159300
1386900	0	0	0	0	0	9338900
158150	0	0	0	18432000	0	0
598080	250980	178700	245710	0	0	0
1037700	0	0	623930	0	0	0

5800000	4603600	3191200	5325200	30885000	31862000	32599000
285310	0	0	0	0	11286000	0
301550	0	0	0	0	0	2481600
0	169010	139760	291330	0	0	0
11347000	0	0	0	159030000	201650000	159920000
3728200	3140300	2685400	3115400	0	0	0
0	667870	0	0	0	0	0
209610	0	0	0	0	0	3920400
0	0	0	0	0	18965000	0
11209000	2555300	1697000	2551400	66107000	52009000	71420000
4298500	4935600	1735200	2562600	22113000	24442000	35663000
6343700	1759300	1041400	2375300	192270000	191760000	182070000
0	254420	194060	233550	0	0	0
744680	704490	699980	1115500	34194000	33292000	35174000
4169400	1321900	846150	1449900	31675000	28844000	27973000
1579600	0	0	0	0	0	11818000
501980	0	0	0	0	0	3755600
157480	256560	142240	218570	0	0	0
6353000	2613900	3092300	5092700	28418000	43517000	42588000
0	29504000	85255	85291	0	0	0
1154600	220140	134770	152600	28909000	30053000	27732000
16244000	9794800	8990200	10741000	146820000	166740000	146430000
486540	255690	216200	222910	0	0	0
2260200	969570	0	0	27254000	32231000	26653000
1966400	0	0	0	66146000	67691000	61670000
754520	0	0	0	7389100	0	7164800
1734200	0	0	0	0	0	7784500
24929000	26978000	15760000	16906000	415160000	530190000	586570000
1523400	0	0	0	36867000	32709000	41337000
629370	0	0	0	25202000	26246000	25704000
3457100	2323500	850590	2863100	0	0	0
484070	0	0	0	0	0	6518800
288770	0	0	0	23536000	21797000	19367000
547640	2018400	1822700	2453300	0	0	0
272190	169900	78654	143510	0	0	0
508060	0	0	0	0	0	7602100
8115300	2515500	1972600	3100300	123270000	115090000	97633000
563600000	0	0	0	1,2498E+10	1,1517E+10	1,0879E+10
0	408660	226320	446290	0	0	0
322350	653780	490480	692980	0	0	0
1017500	0	0	0	20982000	20086000	17808000
32738	0	0	0	0	0	2081900
1100100	0	0	0	0	0	7407100
2009500	675270	633080	1025400	20814000	20203000	19018000
154640	0	0	0	0	0	3355100

32487000	0	0	0	619860000	671280000	660080000
818730	2579800	1063800	1295600	0	26725000	0
493490	0	0	0	8744300	7988900	8856400
47712000	47572000	42325000	68771000	278600000	281730000	286780000
8024700	0	0	0	0	0	24015000
0	1566600	1354600	805200	0	0	0
478670	0	0	0	22068000	22863000	19751000
412820	0	0	0	0	0	7721300
5093200	552540	565650	800340	104190000	112560000	89739000
393540	343860	246480	364400	0	0	0
20502000	15325000	11585000	16441000	108320000	114410000	109930000
2505600	3587700	2335300	3914400	149760000	141550000	139300000
476310	1223500	950650	1535000	35512000	33591000	31990000
7068600	1367200	1104000	1590200	420070000	419880000	396620000
3504500	2802100	1682500	4164000	25484000	23926000	20888000
968070	0	0	0	32547000	36442000	32579000
3618100	6426500	3942200	7518400	45250000	54398000	49357000
165190	0	0	0	0	0	5437900
43277000	37227000	28988000	42817000	1335100000	1257100000	1274100000
4546600	0	0	0	74802000	64545000	64004000
4073300	2500300	2567400	3132800	52520000	69357000	64542000
475920	0	0	0	0	0	17091000
952010	0	0	0	14523000	15297000	13974000
7133400	2557000	2100100	2718200	30542000	37976000	38164000
49403000	3536300	2481200	3222200	1246300000	1131100000	1120000000
370280	0	0	0	0	0	3601400
0	116880000	0	0	0	0	0
1666600	153420	86684	101690	85684000	56207000	76765000
938210	0	0	0	0	0	5615400
34435000	253130	225040	553170	333410000	282680000	284640000
311630	111520	99449	119000	0	0	5362400
1265000	566610	332990	655970	0	0	0
2391800	3016100	1646100	3799200	61276000	64317000	66456000
211890	0	0	0	0	0	2694900
6246700	5770800	3921500	4775500	32033000	33715000	29447000
331000	227300	194470	236070	22193000	19568000	22431000
885630	194790	343110	503710	27470000	33077000	26564000
0	538220	482440	870060	0	0	0
745760	0	0	0	24058000	20835000	21719000
168740	266190	177230	251440	0	0	3282300
5099600	2401900	1615800	3284900	74604000	71674000	70986000
2161300	0	0	0	27138000	23206000	25644000
410700	0	0	0	0	0	6759900
9316800	7395500	6487400	10198000	154650000	175090000	155630000
83944000	29486000	13048000	29137000	311850000	362840000	378970000

5366700	0	0	0	155150000	149790000	139340000
3806500	0	0	0	66004000	64837000	58358000
31706000	6236400	6295200	4738800	148910000	166120000	186700000
0	0	69365	124820	0	0	0
33451000	3281800	2932200	5161100	228980000	220010000	220190000
1236700	1002600	567610	1516900	0	0	0
3310300	3069800	2458300	2945800	99748000	112620000	109420000
4976800	4119900	7213500	5769000	24811000	45692000	33790000
3805100	0	0	0	0	0	14234000
35664000	18944000	10415000	20966000	221590000	247050000	208200000
372920	0	0	0	30818000	32507000	33228000
800620	0	0	0	0	0	8667700
709890	0	0	0	19559000	23236000	21198000
855600	0	0	0	19050000	19794000	17000000
3787000	259110	180590	388010	46750000	42705000	41593000
404720	0	0	0	0	0	20287000
2210600	706950	655280	1036600	0	0	0
1711000	1219400	1000600	1427200	0	0	0
293040	0	0	0	18490000	20114000	17158000
0	1367500	1528800	1455700	0	0	0
0	8573700	0	0	0	0	0
6515400	1859500	1741200	2489600	341490000	326930000	308190000
1083100	0	0	0	11539000	9847800	13740000
5151500	0	0	0	197920000	223210000	179950000
220180	180770	228040	371500	27133000	25675000	25768000
191520	486520	466100	624150	27812000	28983000	29848000
529500	0	0	0	3813500	0	3520500
0	2089600	32580	60279	0	0	0
4349800	1093000	1017800	1395700	203490000	204580000	194250000
198000	0	0	0	0	0	6369900
0	204330	172330	183750	0	0	0
1660400	0	0	0	45395000	51749000	43577000
1275900	0	0	0	0	0	10501000
0	124660	292160	717510	0	0	0
1223100	25636	0	66992	36919000	37836000	38759000
4817700	4859200	0	0	37603000	39794000	32790000
1302900	0	0	0	16974000	12690000	12913000
820290	125770	132350	144260	17645000	19122000	21894000
1281700	332260	283500	362150	31732000	39981000	29297000
3952200	3471500	2792500	3764100	33537000	41257000	40966000
208430	0	0	0	0	0	2650900
880250	0	0	0	15408000	16702000	18493000
4095500	17062000	725460	206570	23276000	11726000	83637000
7069400	0	0	0	65322000	60998000	60663000
990240	1737600	1826900	2107400	0	0	0

2248500	202380	215100	430970	66472000	52828000	55381000
672900	880020	598980	1029600	12897000	16892000	17228000
533400	0	0	0	22381000	22621000	18814000
1120300	505830	279880	699800	0	0	0
598610	0	0	0	0	15696000	0
18152000	10654000	6265500	12988000	228130000	246050000	249000000
1405800	0	0	0	33017000	32828000	28296000
965420	0	0	0	25204000	19776000	22834000
2493200	4777700	930590	9268600	25101000	28337000	31307000
20245000	0	5306700	6692600	0	0	0
240520	0	0	0	0	0	4678600
2707300	0	0	0	47446000	51744000	50383000
21727000	12737000	10002000	15180000	603730000	597260000	571090000
782600	0	0	0	16884000	16478000	14722000
1399600	0	0	0	11575000	12627000	18601000
0	344820	0	152070	0	0	0
31130000	58424000	27209000	34846000	229640000	222980000	247500000
1508900	940950	857080	941990	0	0	0
1367800	0	0	0	0	18440000	21048000
58851000	16683000	11987000	17085000	333930000	383270000	373090000
10854000	4573300	3659800	6125700	108960000	89014000	106090000
2534100	0	0	0	42968000	32938000	28981000
627350	265320	237890	439090	27190000	36071000	26196000
6938500	4851400	2414700	3218100	32083000	37441000	34308000
4714000	0	0	0	0	0	28214000
17741000	1564300	2187600	3450400	147450000	167290000	184150000
0	917510	494510	2498800	0	0	0
26909000	5739800	4538400	7617400	93950000	117950000	114700000
3401200	5219400	5091500	6906400	159710000	148990000	141040000
5264200	9158700	4730000	4395400	69875000	73010000	95932000
18413000	1706900	998680	1539400	500140000	393350000	445540000
609720	557310	254020	507100	10332000	10276000	9874400
15570000	10612000	8813400	14786000	163500000	190430000	178400000
185530	0	0	0	0	0	6662800
406680	0	0	0	10093000	11401000	10337000
195600	300170	174990	166100	0	0	0
575330	240930	135400	295890	0	0	0
2142700	67799	73527	93392	50557000	55017000	47963000
2406200	564180	497080	744690	77429000	67273000	71810000
1134000	0	0	77661	42389000	36341000	37000000
1265700	0	0	0	14817000	0	15558000
4918100	0	133910	230400	81632000	73732000	79028000
964750	0	139050	177540	17285000	16427000	16523000
8498900	9452500	8366700	13831000	97144000	105950000	93940000
1989500	0	0	0	0	0	5953900

599780	514410	347520	802220	12913000	10939000	12852000
298770	176040	123350	323580	0	0	0
2367200	0	0	0	34503000	43429000	34985000
3787900	955590	741710	1167800	34872000	34762000	35747000
2044300	2014800	881010	1193900	22583000	28151000	26546000
0	29298000	79029	0	0	0	0
672640	0	0	0	12577000	12822000	13556000
3261700	5125100	4690800	7114100	97036000	97627000	96775000
0	0	0	0	0	0	0
1366200	0	0	0	39140000	31867000	33118000
464600	1628000	1374900	2146400	0	0	0
2962400	3330000	1799300	5316900	87247000	103480000	82321000
5436100	11811000	3566100	6820500	168600000	192410000	176450000
27845000	6985800	3021600	5851100	548560000	564000000	543490000
0	105960	101160	144220	0	0	0
3533400	351960	157810	176350	106740000	90040000	121730000
367540	44696	41927	65254	0	0	9899200
627150	0	0	0	0	0	26276000
2879700	0	0	0	0	0	8618000
1134400	0	0	0	23326000	16660000	13904000
1752900	0	0	0	45149000	51147000	46418000
3193000	1683700	1018400	1476400	116320000	108770000	107850000
743020	0	0	0	18055000	0	0
1180700	2489400	1140500	1734800	17858000	18575000	0
7341900	1171500	2096400	8817000	12466000	10777000	12086000
205370000	25815000	21060000	35358000	5477900000	5380600000	5043600000
103510	88292	54639	104250	0	0	0
2113600	0	0	0	63114000	75250000	59759000
3364900	346980	97826	321490	198670000	133770000	171550000
197570	227150	87239	149350	0	0	0
1003000	0	0	0	13922000	16080000	14160000
6941600	564470	573240	665760	94097000	96571000	87067000
1701200	0	0	0	0	0	7636600
4704700	925360	141100	2096600	32380000	37198000	36038000
19795000	21353000	6149000	15574000	103140000	110150000	106880000
917970	0	0	0	0	0	2747100
363150	0	0	0	8068500	8507400	7330900
7363000	7431300	5695400	9802100	118590000	103680000	132000000
728330	298860	183750	311100	8351500	9890600	9529900
718440	1502200	1166700	1792300	0	0	0
0	765980	542990	658770	0	0	0
43173000	4741000	4180100	6409100	341740000	334380000	384130000
32656000	7807200	7673200	6446100	104130000	156800000	138390000
0	645510	302560	722030	0	0	0
123390000	58118000	36108000	55531000	734340000	740940000	523810000

24118000	15613000	0	7367700	55344000	55722000	54592000
45609000	13295000	12858000	33419000	478850000	320930000	368010000
43592000	37326000	22629000	40289000	416040000	433840000	472640000
951690	224900	336300	508520	16695000	18425000	16073000
268090000	186570000	207350000	261610000	1147800000	1184400000	1222000000
222480	0	0	0	0	0	6491500
37002000	2047200	1217800	2037100	0	0	0
3497600	0	0	0	67241000	72364000	73952000
413170	3923600	2642100	4854600	28664000	30357000	24379000
195990	0	0	0	0	0	2199400
44123000	30688000	18439000	38451000	356160000	392720000	361320000
4801100	6999300	5447500	10417000	286170000	266270000	246360000
831310	0	0	0	9043300	7884800	7475000
638820	302610	72255	170440	11790000	12400000	14117000
2156000	545820	311790	490960	18116000	20046000	21061000
209120	586490	722210	391820	0	0	0
1820700	878580	955730	1365300	0	0	0
4466200	441650	327320	520320	126560000	104380000	93887000
0	1596500	0	0	0	0	0
138000	0	0	0	0	0	3820000
7651200	41097	51344	188760	166740000	152130000	138130000
483930	0	0	0	0	0	2534400
163520	0	0	0	0	0	2813700
311900	0	0	0	0	0	9100500
3218700	390900	364630	539800	95989000	97445000	78493000
215120000	0	0	0	0	0	2736000000
2118900	24239000	20898000	304520	888280000	797390000	904900000
1028400	957290	569780	1039400	20000000	24344000	20771000
12526000	4459600	4322400	5289500	151160000	155720000	140270000
3796400	0	0	0	86167000	94213000	77600000
1116400	0	0	0	41101000	34989000	30054000
607880	398870	240440	643870	0	0	4093100
0	0	0	0	0	2136700	0
1520600	0	0	0	68005000	70351000	64394000
4316600	514400	493820	624370	111400000	88666000	95020000
0	2547100	1560400	1700600	0	0	0
11638000	7480400	5149000	8573500	235630000	241730000	220810000
840410	958110	3952400	4980400	0	0	0
1909400	228270	587540	634950	23821000	25585000	24765000
702650	0	0	0	10468000	9898000	10550000
35032000	24657000	12997000	44299000	279230000	264930000	296100000
8151000	1331900	0	824430	54701000	56888000	54863000
229370000	21090000	13818000	18830000	1147400000	1038400000	977160000
866230	0	0	0	24178000	17107000	14885000
13979000	7391700	5991800	9758700	428340000	498270000	438720000

1039900	362390	89449	453330	46017000	48112000	47386000
2243700	0	0	0	248540000	227450000	214120000
1082600	0	0	0	100450000	116950000	97232000
59968000	0	0	0	1511500000	1592000000	1425700000
569420	0	0	0	13090000	13736000	14561000
1168900	0	223700	236380	0	0	0
22012000	194520	612240	717040	260610000	177700000	249080000
3519300	238310	159940	207770	71560000	62242000	56808000
41161000	1163200	935280	1461200	195160000	157150000	149680000
3194700	2786000	1861200	2273700	95425000	105440000	103190000
814530	1272600	1341400	1964400	26963000	25665000	25684000
9733100	3040500	2492500	4436600	348780000	435610000	381990000
15699000	0	0	0	399480000	394720000	380590000
502320	0	0	0	0	0	5261300
1549800	389320	605960	896050	24774000	26026000	27818000
108810	0	0	70472	0	0	1790900
17144000	241090	109950	360490	293460000	189760000	202730000
3225700	3570200	2578500	4555200	73733000	58498000	65460000
349300	0	0	0	8941800	8620300	0
7510800	0	0	0	157900000	155740000	141260000
446330	0	0	0	0	0	8681900
0	0	0	0	0	0	0
3038500	2686500	2418200	3293300	156470000	158990000	139470000
169100000	3533800	2172300	4322500	3147300000	2958300000	2735500000
2450900	416850	0	219010	27736000	28780000	23184000
2699100	2649900	2216200	5986100	39610000	38604000	32255000
631930	0	0	0	11186000	9634000	10339000
1395400	0	0	0	0	0	15660000
464530	0	0	0	0	0	6255800
452520	560480	282670	696250	0	0	0
3894200	1520100	1355400	1701700	92845000	82081000	97007000
591140	0	0	0	28118000	25897000	24321000
97145	0	0	0	0	0	3052500
1488400	0	0	0	23077000	23877000	20465000
637660	0	0	0	17802000	17678000	17729000
1422500	0	0	0	72203000	63162000	64298000
1479700	890360	492010	1219100	24424000	24892000	24360000
340830	0	0	0	0	0	3314900
1272700	805670	517220	1076900	0	0	0
1496100	0	534730	729310	0	0	0
0	1942800	0	0	0	0	0
1131700	622350	547470	823760	18285000	18966000	19776000
472870	0	0	0	11581000	13185000	12019000
4674400	100390	47586	112560	280890000	250860000	249890000
588740	0	0	0	15339000	15056000	14407000

6168000	1721100	1099700	1434200	101950000	126870000	142170000
0	968690	636980	1004100	0	0	0
22865000	2160700	1851000	2469400	198400000	225110000	241020000
436820	348920	295590	403580	0	0	0
0	596000	646730	588490	0	0	0
484640	2693700	126750	396620	23758000	28482000	18789000
2523800	0	0	0	15495000	16867000	13766000
1126900	587150	679810	687980	10293000	11461000	10945000
3046300	0	0	0	10382000	9666800	9275100
533830	0	250700	836280	0	0	0
5554300	0	0	0	164510000	177490000	154190000
0	149370	231450	479470	0	0	0
3625500	254360	0	1032700	22002000	21586000	21038000
0	622320	0	0	0	0	0
3079300	0	0	0	44356000	46990000	38187000
1027500	1241300	944150	1733700	30545000	33053000	32869000
330540	0	0	0	0	0	3956800
1608100	1641000	1603700	2236200	0	0	0
0	0	20749	65002	0	0	0
479530	349020	859120	1050700	0	0	0
429040	0	0	0	13323000	13906000	13742000
201600	165610	129890	341300	0	0	0
461590	76536	330200	412300	11757000	11153000	10840000
1407400	0	0	0	0	0	5264900
1363300	1447300	1517000	1892500	44868000	48616000	47546000
1261900	0	0	0	0	0	6608700
11086000	2316700	1412900	3604600	43648000	42241000	44253000
0	824150	570460	1514700	0	0	0
33301000	8271900	5011300	8854300	138630000	151750000	139510000
1660800	0	0	0	14865000	15254000	12848000
0	997730	331520	1277000	0	0	0
0	1709300	1084300	1673800	0	0	0
2310900	0	0	0	23240000	26631000	20906000
3866700	0	0	0	19984000	19327000	16698000
11904000	1061100	1034000	1627600	89533000	100690000	96542000
1212000	3678100	3639000	6159300	65445000	63267000	67681000
14261000	0	0	0	100180000	105020000	95763000
1912300	98770	125370	0	34094000	26716000	20160000
910350	0	0	0	19825000	19604000	19459000
338660	0	0	0	0	0	2280300
4211200	0	0	0	14728000	19985000	15585000
294540	0	0	0	9853000	8339200	0
2922300	0	401300	427790	60936000	62789000	52968000
698360	0	0	0	0	0	3657300
228790	0	0	0	0	0	4450300

1088900	0	0	0	20042000	20062000	15567000
176390	135650	84526	230520	0	0	0
2940900	0	0	0	91678000	75233000	73061000
0	924530	300330	608540	0	0	0
415410	0	0	0	0	0	3729500
2791700	4307500	2187400	3932100	16554000	19864000	18176000
738960	867570	721590	669690	0	0	0
993630	950060	896430	1003900	19635000	23193000	20009000
18804000	267060	131820	239840	1049100000	179800000	547120000
2024300	0	0	0	19038000	29072000	21119000
272610	942070	635350	1242900	21663000	23233000	19289000
2747000	372300	233870	0	32647000	35738000	32700000
4511700	4240100	1876600	2864900	0	0	0
1115600	0	0	0	0	0	6676900
679880	0	0	0	0	0	5086500
2026000	212780	648610	989480	0	0	0
16133000	0	0	0	0	0	12070000
20845000	11843000	4021400	12684000	191110000	180230000	200170000
5272300	5270400	2611800	3177900	43296000	40626000	47596000
274380	0	0	0	0	0	2463400
834010	0	0	0	28465000	29457000	26985000
943120	619470	587600	407810	0	0	0
112070	0	0	0	0	9376700	0
15259000	0	0	0	336670000	323580000	311680000
5813900	1270400	713750	1287000	217540000	238810000	237870000
748920	68439	64141	85222	32546000	30930000	30720000
0	1641600	1609900	1899600	0	0	0
6642700	346740	232380	500020	176580000	113900000	150750000
405340	1192900	902990	1478000	20265000	26008000	22892000
3089400	4034900	3195000	4327200	55708000	55553000	77191000
6299100	3388100	2007400	3925900	183030000	172850000	159590000
0	517470	398160	2816700	0	0	0
2705500	1978100	1738400	2872000	0	0	0
162020	0	0	0	0	0	6182000
282210	0	0	0	15559000	0	13637000
0	4885900	2078400	4458900	0	0	0
342160	0	0	0	0	0	9471600
918590	1246200	556630	559380	0	0	0
2110400	501710	299790	581690	31901000	29092000	29117000
23738000	3099600	2061800	3982300	391500000	329090000	337540000
387520	100880	82437	162670	8160200	9145300	7319000
277030	0	0	0	0	11427000	7055300
2210100	0	0	0	0	0	8267300
0	252630	297980	416210	0	0	0
288890	134480	116840	191930	0	0	0

4695300	807850	765860	1101000	18350000	22659000	16942000
590400	0	0	0	14312000	11766000	12889000
0	0	0	339390	0	0	0
4449500	1531400	1299400	2489400	63752000	68551000	59181000
22244000	2315500	1394800	1999600	701500000	608260000	617780000
0	0	0	0	10662000	0	0
2514900	0	0	0	23923000	25776000	27682000
410020	0	0	0	0	0	5828400
11209000	6788400	5883900	6325900	74775000	87031000	82121000
650230	0	0	0	34837000	35771000	35511000
1439300	0	0	0	66431000	60631000	59979000
1409100	496070	478440	648330	30280000	29329000	30127000
1025000	95925	106130	134620	60226000	52839000	53367000
437620	0	0	0	23884000	20229000	21830000
0	523090	203630	513680	0	0	0
377850	0	0	0	16101000	15309000	14727000
119800	0	0	0	0	0	4660700
3123800	0	0	0	68470000	64503000	67312000
343620	0	0	0	0	0	6427000
1529400	0	0	0	100430000	83931000	88075000
5289600	0	0	0	470930000	452870000	431670000
2247400	0	0	1043200	0	0	13451000
2136600	1253500	470900	701560	22963000	31909000	46081000
813200	358760	436960	657340	0	0	0
1124600	0	0	0	132940000	142850000	127970000
2908400	1354900	600490	2757800	33715000	33721000	31136000
0	493310	316450	416190	0	0	0
1706400	0	0	0	0	0	7660000
1645800	0	0	0	0	0	7387900
3125900	599810	426250	871450	25184000	26182000	23769000
0	3601300	1408400	2486200	0	0	0
21434000	3159900	1334500	3251800	48048000	59492000	64293000
1130200	0	0	0	8478600	9166600	7807700
817420	415530	151380	490250	0	0	0
5254800	0	0	0	13173000	18550000	15713000
4672600	1237100	0	921280	23454000	18373000	16915000
13191000	234930	501970	494430	424260000	458660000	398250000
1223100	823540	600890	683540	0	0	0
1948600	3251100	1458100	1333200	9303700	0	9220700
3366600	0	0	0	0	0	7556300
72960000	3717700	3097800	5172600	320980000	355500000	333410000
3646000	3810000	1066400	3623100	47655000	46134000	49208000
0	851110	631060	957700	0	0	0
8057600	2188100	502080	1588500	83084000	70295000	86582000
1230000	0	0	84844	39728000	39195000	40456000

0	14943000	0	0	0	0	0
0	2229500	0	0	0	0	0
626060	530920	395670	591400	40148000	50097000	39939000
211610	169760	0	0	11877000	0	0
0	39919000	0	0	0	0	0
0	4040600	0	0	0	0	0
376850000	334580000	294030000	411660000	3919700000	3662400000	3051600000
41499000	140220000	13566000	14290000	1243700000	870400000	930660000
0	0	132280	0	0	0	0
242010000	32202000	425580000	50606000	426620000	337620000	1522700000
14792000	5135200	108510000	4131900	0	0	0
66214000	9181100	17682000	8423900	91998000	53472000	204960000
23606000	5419700	53146000	2422600	59922000	0	127820000
61065000	5125000	40843000	10523000	85694000	74882000	422870000
3999900000	3229500000	2140100000	3051900000	1,1012E+11	1,2427E+11	1,1616E+11
1018300	0	0	0	0	0	5333000
32950000	108140000	7257300	9893300	383610000	256310000	626400000
11196000	1161000	1590000	1785800	272360000	198060000	160460000
21507000	70372000	51210000	80606000	951150000	1004200000	917210000
11770000	148700000	832290	2229600	126030000	106930000	276360000
1418100000	499430000	304240000	294440000	1,2201E+10	5119400000	2,6143E+10
19765000	32585000	12538000	10317000	618340000	347460000	571250000
0	859260	631370	1003900	0	0	0
62758	167100	129510	93887	0	0	0
327720000	260960000	132920000	84594000	9189100000	1,552E+10	6620800000
850390	0	0	0	0	0	24813000
0	105920000	73923	25968	0	0	0
7951000	11973000	891370	1442300	192990000	160160000	171250000
0	311250000	362280	75470	0	8615800	0
2918300	2088600	2369100	1969300	71510000	80779000	71102000
986830	0	249570	421880	0	0	0
171830	95973	82061	147970	0	0	0
901390	2206900	112000	135100	0	0	0
3443200	1957400	0	34648	112370000	90071000	85958000
612640	0	0	0	0	0	4583500
0	5201600	0	0	0	0	0
3690200	403620	192400	215220	54679000	33047000	102070000
0	0	0	0	0	21625000	0
1618000	1596800	547770	816100	0	0	0
0	90116000	98026	0	0	0	0
0	322960	0	0	0	0	0
1112200	1177200	251260	145630	26672000	27502000	27626000
20396	541580000	695990	166230	0	0	0
2508900	182710	313870	224640	24793000	23025000	43799000
0	0	0	0	0	0	0

8603300	279060	192890	128210	10587000	7548100	194950000
4964000	2396000	1360000	1319200	178530000	392100000	284330000
0	680470000	548530	0	0	0	0
36370	28747000	30899	0	0	0	0
35186000	0	0	0	664190000	282000000	213800000
0	81560000	0	0	0	0	0
0	12431000	0	91956	0	0	0
11518000	5456800	4203400	5183600	222650000	247580000	217130000
3541700	0	0	0	110200000	119360000	120300000
872370	0	0	0	19633000	22526000	22135000
217870	0	0	0	0	0	4890100
373960	0	0	33832	16841000	16059000	20397000
801130	315780	260540	450000	0	0	0
8966800	6651500	629510	2741200	43206000	49763000	48073000
1760200	2603000	1810500	2991400	33414000	34152000	35776000
2666800	0	0	0	17891000	20060000	17844000
239170	53190	41128	76965	14896000	8850700	10894000
0	984270	359830	1122500	0	0	0
3552500	2323500	1478000	2772800	31840000	34121000	31705000
679470	1276400	419320	750220	0	0	0
2228000	0	0	0	45306000	49751000	43010000
1271500	793400	483260	756600	39285000	48165000	34934000
1231800	1015900	167730	335730	34679000	32901000	33010000
595230	0	0	0	65132000	61181000	58800000
2867800	3516100	3801400	4502900	138740000	133620000	132130000
1442900	2277700	3929700	6450200	35442000	15064000	27809000
1349800	0	0	0	0	0	5049400
14211000	2241200	1471900	2601300	110080000	107860000	105300000
0	1477500	0	0	0	0	0
1135000	2168700	4777500	1154300	0	0	0
5382700	0	0	0	59991000	78742000	73677000
627530	0	0	0	16662000	21305000	19062000
1987600	1844500	1301000	1899500	67502000	73068000	66417000
188400	384540	289570	355310	0	0	0
7601700	1065800	830530	2113400	96201000	97852000	95361000
731260	497440	225100	616310	23534000	32540000	28919000
1024300	759800	461340	869610	0	0	13794000
1040000	18207	264730	229710	45054000	37711000	37932000
554620	768550	407950	640780	0	0	0
1154400	0	0	0	0	0	11227000
1857500	477150	207910	563820	26285000	20739000	19924000
14657000	3079200	0	3012300	21409000	21259000	22487000
44284000	3581700	2309800	5311300	140440000	136810000	143550000
1918800	0	0	0	11541000	8268000	9248000
15714000	4728200	1155400	4388400	0	0	0

8158600	0	0	0	155590000	135870000	126880000
953150	0	0	0	0	0	12836000
0	4033000	3933500	4280400	0	0	0
2230400	1336800	912970	1966900	60305000	67650000	53801000
272300	16325	17554	29008	38411000	41346000	38001000
0	28994000	11492000	9149500	0	0	0
3250700	0	0	0	18067000	21786000	20731000
25250000	424090	252820	712620	606810000	607390000	566950000
544670	0	0	0	37467000	34619000	34227000
242720	195210	181690	213940	9437300	6517400	8576300
511000	0	0	0	0	0	3058400
12003000	0	0	0	170120000	142430000	140130000
2075100	1698800	1019200	1160000	0	0	0
150140	0	0	0	15628000	0	11628000
99661	69036	72039	86567	0	10795000	0
0	0	0	381430	0	0	0
15540000	4189200	4310500	7302700	563810000	599570000	525230000
17697000	7584100	8513100	13437000	200940000	181860000	190380000
1571500	0	0	0	0	0	9406100
19851000	558570	309230	1204500	291790000	311400000	295100000
2761700	1091600	699340	1486700	63821000	62327000	70392000
898780	0	0	0	0	0	4707000
647260	744350	627820	1011900	52444000	49024000	47760000
2139000	233430	181670	269230	65153000	71349000	57372000
1192400	0	0	0	0	0	10705000
1937000	0	0	0	0	0	4347500
16463000	2568300	2394400	3006800	391100000	426580000	416510000
16411000	0	0	0	117510000	127470000	111840000
531500	308500	296430	311190	0	0	0
962120	0	0	0	10677000	9916500	11316000
429040000	181900000	100990000	230870000	5602100000	5366200000	5353100000
0	636560	175630	269950	0	0	0
1649700	0	0	0	20286000	22239000	22608000
3833300	155350	153550	155500	105030000	115670000	107300000
1728300	0	0	0	33764000	28724000	30100000
9752000	11916000	9438800	14079000	81661000	92029000	78581000
262960	0	0	0	0	0	2754300
4372900	2134700	0	3408400	55384000	59795000	53049000
19606000	9324500	9002200	11863000	154200000	188100000	176440000
0	0	0	0	0	0	0
79176	0	0	0	0	0	4501900
64708000	11757000	10523000	13830000	334490000	357510000	336290000
7411700	0	2261700	2247800	0	39602000	33219000
1383100	4985400	4185200	9160700	53976000	65082000	52238000
44023000	40248000	34701000	46888000	1288800000	1347000000	1280400000

22606	20461	0	0	0	0	0
2281400	0	0	0	0	0	11948000
592360	381360	120070	143980	0	0	0
155970	0	0	0	0	0	6651300
3857500	0	0	0	63966000	60691000	63210000
1045100000	489330000	244110000	186250000	1,469E+10	1,9484E+10	2,6299E+10
418860	0	0	0	15449000	15986000	13976000
4480500	176460	312080	445840	113990000	121170000	159910000
1847100	1982500	1314800	1909900	34541000	33409000	34841000
9893000	5840600	4643600	7607000	49818000	58780000	50525000
689310	0	0	0	31051000	27972000	23370000
14907000	8172500	8207900	11188000	135940000	155940000	160700000
0	533390	396610	796100	0	0	0
594720	90525	46837	76075	18009000	0	19557000
11956000	11054000	5815700	9485800	64230000	75238000	70741000
3113100	0	0	0	243710000	244270000	217770000
66327000	29020000	27013000	31360000	279780000	317790000	290700000
1333600	0	0	0	0	0	12971000
5145600	0	0	0	27215000	25598000	32551000
1180300	540420	349080	750920	0	0	0
244590	0	59396	111520	0	0	0
0	8261200	6614600	9460300	0	0	0
0	650100	521230	805790	0	0	0
17720000	9920800	8809600	9251200	164100000	216520000	182040000
2132500	397960	326290	393020	64705000	74313000	81867000
0	3306700	2705300	2472400	0	0	0
1414200	409950	386510	629320	0	0	0
11744000	1430700	840500	1480200	45124000	44343000	71945000
1237200	0	0	0	0	0	13884000
808760	540800	132490	191990	0	0	13917000
0	1539600	0	0	0	0	0
12731000	849880	579670	827060	177030000	179990000	186930000
153070000	68702000	37972000	72843000	3579100000	3593900000	3589500000
16217000	3471800	2769800	4360800	47478000	56317000	47767000
39261000	42966000	35414000	58305000	351170000	377550000	386310000
906000	230160	125480	244480	15925000	19708000	17234000
498880	0	0	0	0	0	9331100
911260	135760	126320	142420	0	0	15681000
6854700	6140300	3098300	7665400	65287000	73668000	62867000
17320000	4351200	4627500	5685500	202840000	227880000	201580000
1458700	0	0	0	53551000	53538000	41324000
1833200	0	0	0	12577000	12719000	12319000
6254400	0	0	0	54569000	54760000	52666000
8212600	8474300	4607500	6483300	52160000	66043000	63784000
5124700	2607300	2085500	3500000	35780000	40827000	37562000

1017400	0	0	0	0	0	8373000
0	512490	363850	486700	0	0	0
6167600	286720	315640	434820	56724000	57148000	55322000
6202100	0	0	0	36703000	41530000	35776000
622150	0	0	0	0	0	4189200
826550	0	0	0	27483000	19684000	19433000
4149900	1963600	1837000	2035300	23411000	23081000	22107000
22899000	1257900	826580	1407300	925420000	939300000	944070000
1554000	0	0	0	15790000	14497000	16083000
1524200	2212900	0	497690	0	0	0
11953000	16571000	9624400	8164500	0	0	0
0	0	0	0	0	5847400	0
25223000	1490300	1006900	1588900	176470000	220380000	196240000
4904400	0	0	0	59669000	52924000	54339000
43218000	0	141980	333500	1051500000	1133100000	1013200000
532890	0	0	0	0	0	3189400
1233500	54135	273270	345830	93571000	109230000	80085000
62132	0	0	0	8971300	10406000	9401900
8440600	300180	322210	419050	259430000	268660000	242940000
25782000	9115000	7070500	11052000	401580000	459220000	411760000
2409600	36331	40982	69729	104500000	87668000	93180000
4052600	0	0	0	82104000	82091000	77088000
1256000	0	205390	293110	8442200	10827000	0
3657000	307280	158250	271020	167370000	127660000	123170000
152040	0	0	0	0	0	2729900
6236600	8498600	6441700	9526900	123810000	143760000	139630000
552710	0	0	0	10816000	10525000	10228000
2655600	293570	147430	593280	44729000	54882000	52307000
12152000	11916000	4638000	7343200	160190000	202930000	298030000
0	721630	587700	684240	0	0	0
943150	495910	245170	381100	19024000	19636000	19267000
28630000	25146000	15830000	27988000	324490000	340600000	326010000
24900000	47642	0	124150	516210000	526440000	473820000
234450	325190	271520	417200	29982000	24321000	25814000
2377000	0	0	0	0	0	5335100
15523000	5418200	2943500	7007900	167120000	192600000	216570000
7065500	1200800	1062300	1682600	99054000	100090000	114250000
490520	0	0	0	24425000	27224000	23805000
1797700	0	0	0	91408000	82749000	81037000
1075400	82631	373970	731390	11172000	9981600	15799000
1944000	3177700	2649900	4658400	0	0	0
1093700	298240	42229	339670	17424000	15608000	11540000
5058900	2391100	2618300	3755300	97613000	103170000	113690000
4439600	367390	320970	815950	35871000	28114000	27306000
4462400	6069400	3822700	8580400	106780000	108180000	98096000

6079600	11881000	3075800	9519200	51560000	58864000	51723000
3070100	1011200	840900	1210200	88700000	106940000	107690000
20096000	7961800	4697300	5538700	68742000	56031000	78711000
1129500	248900	216010	338180	79980000	78016000	71835000
1259900	34335	22810	26816	148510000	145920000	140870000
6213800	8878700	6780300	10675000	56148000	62603000	57009000
0	568110	313270	1099500	0	0	0
78771	0	0	0	0	0	5362900
648570	0	0	0	0	18511000	16980000
1833400	165170	98018	132740	71372000	99908000	51318000
1096300	0	0	0	97696000	98446000	94765000
0	14923000	0	27639	0	0	0
6079700	1119800	1084200	1783700	195850000	213210000	194500000
79425	1378100	1165200	1346100	0	0	0
2322500	879350	861960	1368000	99177000	104490000	100240000
3190300	1648800	1516600	2081400	79392000	97032000	83547000
0	2493700	0	2379500	0	0	0
688520	0	0	0	0	0	9787200
0	232150	204780	237370	0	0	0
1523400	0	0	0	20355000	20331000	17611000
325510	0	0	0	0	0	8280200
1119500	0	0	0	25822000	30345000	27071000
13617000	2833500	1852000	3700600	184030000	201030000	177180000
1480900	292690	274870	418110	60241000	64969000	70076000
703960	0	0	0	0	0	6846700
137210000	89664000	58128000	70380000	4364200000	4384200000	3975000000
7629700	9005200	6328400	9165400	38717000	53219000	34583000
1968000	0	0	0	0	0	29447000
5385200	5200500	1476200	2488400	44575000	30597000	67549000
14126000	1202100	945720	1346600	115590000	130660000	140260000
5804700	149250	80878	0	192330000	155300000	156370000
1862200	0	0	0	59409000	62775000	58322000
0	897990	575920	611320	0	0	0
52043000	24453000	26341000	32885000	883440000	798340000	723110000
2981900	1589700	567970	679980	62164000	42917000	41895000
355240000	286080000	143170000	193540000	2491900000	2915900000	3053300000
134610000	5465300	3377600	5324900	2070600000	1817600000	1823700000
584960	0	0	0	36223000	34090000	33217000
4063300	0	0	0	0	0	15200000
8281600	1292600	1296400	1222000	75575000	76956000	63047000
23909000	1326200	567240	1234500	887260000	779810000	422460000
57004000	50029000	36228000	52064000	473980000	501850000	448700000
65439000	123770000	105880000	162170000	582300000	676120000	569580000
9170200	0	0	1582200	40084000	33398000	34089000
0	188400	180760	166410	0	0	0

737170	0	0	0	0	0	3860600
225840000	4295200	2277200	3161300	6033700000	5516600000	5018600000
316560000	11266000	7186900	11669000	8446600000	7693800000	7679100000
15131000	2749700	1799300	3251000	738460000	769540000	744090000
1071400	0	0	0	14120000	12805000	0
8034200	1497000	1062100	3442000	126910000	114300000	107270000
485300	797780	0	410120	0	0	0
1607400	608030	319990	417620	23266000	24078000	26687000
3113100	1820000	342170	689090	100910000	86944000	95778000
105660000	124790000	72474000	108440000	1397500000	1502700000	1519400000
4477300	7157700	2830000	3006900	689370000	762730000	642840000
82854000	42649000	64969000	58800000	445240000	817820000	582130000
128280000	127420000	250620000	168080000	672230000	1425000000	926580000
1233100	0	0	0	46636000	42869000	43420000
2438400	513720	362470	534990	23244000	24265000	22990000
1379800	865490	654080	758750	14366000	14422000	13500000
2220800	0	0	0	0	0	4984400
9562700	1780800	1274100	1801500	180220000	179480000	181520000
3370100	0	0	0	86090000	70502000	81267000
10676000	4679300	3648100	6421700	61835000	66035000	60623000
15887000	159270	25521	52761	968630000	1006900000	931980000
3148500	0	0	0	53564000	45979000	47885000
50456000	5171200	4234800	5616300	1310600000	1468600000	1324000000
1965600	735150	382720	465420	148520000	158380000	151530000
341470	144170	0	164430	0	0	0
43326000	8813300	4939800	9620400	394830000	415900000	426580000
900990	0	0	0	47540000	47592000	43469000
4736300	6050300	1782200	3252500	81435000	78861000	119350000
1424600	126580	104190	119170	27247000	21635000	23004000
0	3390400	677900	248440	0	0	0
10155000	1681600	1344900	2546200	123720000	124740000	122310000
65580000	14397000	7785000	11498000	444140000	426660000	429480000
23082000	6495600	4012000	7721600	119590000	97206000	111540000
5989000	2259700	1023000	1149100	151740000	119630000	122440000
44633000	29779000	21842000	32293000	436270000	465100000	457070000
0	1466500	524670	959210	0	0	0
58849000	57832000	41090000	77831000	1031800000	830990000	908200000
115350000	35803000	30406000	48186000	546930000	624550000	602260000
1129900	0	0	0	32218000	26494000	27719000
57091000	581080	375920	670940	1480800000	2005300000	1641600000
0	2255700	301560	0	0	0	0
32296000	59629000	51826000	70735000	561020000	573140000	497930000
38677000	8988400	7606400	13976000	223010000	217830000	221940000
0	1015800	1289100	834690	0	0	0
871950	0	0	0	9375900	10731000	9109100

48950000	0	0	0	921110000	820450000	795400000
1413200	6144500	1401400	1671700	0	0	0
0	3690600	8950700	10812000	0	0	0
3209200	0	0	0	110320000	115020000	113630000
632850000	0	0	0	7141600000	6659200000	5776700000
8189300	1321800	762100	1790900	121180000	116700000	123870000
3270000	503720	375230	708260	112720000	121580000	109140000
18359000	37485000	29768000	36801000	440120000	432420000	458170000
347660000	210710000	127690000	126290000	8598400000	3911000000	1,0624E+10
4196400	0	0	0	44008000	38467000	46682000
62958000	13563000	11532000	15429000	639580000	685330000	688240000
40808000	18163000	19463000	24199000	191740000	184400000	200930000
20230000	13682000	8804500	11455000	163630000	255230000	355900000
6310900	5921100	2398900	5566900	104540000	121380000	108870000
27373000	6758700	3732400	6263800	223620000	298090000	301120000
372320	62894	37307	62819	11354000	11887000	13182000
42802000	15442000	12132000	20794000	292340000	332540000	316040000
570600	0	0	0	11835000	12316000	11626000
617260	0	0	0	0	0	4156300
56206000	30900000	23800000	35745000	1418300000	1415400000	1396400000
2109400	99632	65254	98306	95751000	87340000	88831000
26211000	11489000	5145500	5800000	90334000	0	95535000
2549200	0	0	0	136650000	127420000	133730000
8237400	1734500	1554800	2514500	255290000	221490000	212050000
17984000	16030000	10755000	20722000	248300000	236690000	224620000
31434000	7739800	6461200	8563200	107120000	118660000	122170000
15136000	4385600	1910000	4552000	91892000	101800000	90583000
1899100	0	0	0	26387000	27719000	19750000
3408200	0	0	0	22725000	21827000	20793000
369650	0	0	0	0	0	8020000
162560	51639	0	45292	12518000	12284000	15467000
3495100	0	0	0	87485000	79254000	77878000
188670	0	0	0	29593000	0	24518000
23131000	11925000	9935400	12918000	80368000	81998000	116750000
1681000	1422600	1409100	1561900	0	0	0
446430	197210	257860	275430	0	0	0
1451500	134310	29287	274890	77584000	84620000	83025000
5430900	2842600	2261900	4077200	123270000	136970000	127280000
1061600	1325300	1224900	1630100	208200000	230950000	197810000
70662000	1084600	1655600	2120900	634680000	684530000	625380000
1387800	0	0	0	0	0	3114800
13529000	0	0	0	75858000	78161000	66043000
1751100	0	0	0	272360000	274070000	256050000
2111400	1429200	804300	1733000	22923000	23023000	21772000
30007000	40829000	27420000	42863000	353780000	421600000	463770000

85886000	6413400	6794100	11783000	###########	2635800000	2809400000
1290600	1724300	1080800	2037300	0	0	0
4411800	0	0	0	273360000	283680000	251040000
11443000	0	0	0	124780000	135190000	126110000
108680	0	0	0	0	0	5691800
11578000	18535000	9213800	6748500	53436000	53157000	53358000
106340	0	0	0	0	0	2943600
1650400	1668800	908460	1583200	0	0	0
1939900	0	0	0	70677000	59615000	63075000
2735700	1187000	1894000	2560500	36668000	38298000	36619000
10575000	11551000	9703800	14858000	555610000	566330000	554540000
15014000	8902900	5462800	13714000	187920000	174770000	180530000
20672000	4493400	2726700	7129200	101050000	124120000	103660000
170270	0	0	0	0	0	6496900
1396700	2669600	1375700	1633400	0	0	0
0	8437900	2147100	968600	0	0	0
2140100	0	0	0	0	0	9606800
0	1071500	301950	0	0	0	0
21197000	11168000	8562900	10559000	80500000	81902000	70119000
47517000	7215800	4838400	4743800	162650000	244210000	184720000
18563000	1515500	2274600	2462500	226000000	264860000	223570000
2639400	221570	165820	262730	33360000	37018000	36979000
42406000	11413000	8466100	13425000	236280000	261330000	285040000
57437000	8898200	7239700	6560500	125860000	127830000	159430000
32837000	1571500	4991700	5541200	176860000	200930000	182050000
9268700	3216600	2648500	3781500	0	0	0
46912000	13459000	10086000	12568000	264900000	325110000	309640000
0	8301400	1340200	611540	0	0	0
15110000	9778700	5114600	8576100	40454000	37531000	48520000
20851000	11310000	5571000	6180600	82154000	82090000	87245000
14921000	20726000	10091000	9692400	71004000	74453000	90207000
84997000	14068000	18054000	23718000	557120000	602050000	563230000
39011000	29039000	24322000	29926000	413320000	480190000	411620000
36662000	7755200	8412100	9931200	123030000	151880000	153780000
13816000	0	0	0	40710000	53275000	43741000
0	0	0	0	0	8052900	0
37388000	0	6740900	10381000	0	0	0
38255000	12330000	9279500	14740000	264760000	303830000	305600000
38387000	10481000	7963600	11268000	265380000	280240000	283260000
52202000	10695000	7728700	9857900	293390000	332630000	334440000
8215300	3481100	3336700	3913500	0	0	0
8339700	3981300	3713500	5244300	112370000	112450000	107240000
937820	0	0	0	22523000	20550000	18449000
43105000	18690000	12698000	21352000	223240000	169920000	166350000
3159400	5683500	762250	1143900	0	0	0

86823000	64708000	38034000	48854000	###########	1301800000	1325200000
8793600	264740	152930	373220	70006000	90312000	87034000
291400000	89352000	63331000	83876000	4657100000	3916000000	4101700000
231140000	5897000	5355400	8886800	3962200000	3391100000	3385300000
417500000	14886000	10839000	19463000	6045700000	5228300000	5512200000
1833400	0	0	0	12196000	12491000	11971000
5446200	1226400	600970	1530900	59661000	46840000	53774000
3532800	318130	259780	409660	41467000	34752000	34811000
459110	0	0	0	8978400	8964200	8596300
35165000	6601300	5718500	9011100	5503800000	5398900000	5146400000
0	902260	363220	397200	0	0	0
18359000	34738000	16981000	15525000	156890000	193800000	69415000
0	4758700	1504500	2633300	0	0	0
44028000	25196000	16767000	22919000	655250000	835160000	1140000000
83806000	56383000	32861000	81200000	757940000	722160000	717000000
5538100	0	0	0	67749000	68528000	67213000
26591000	1189200	696840	958280	429540000	428490000	276200000
1305500	1444000	569380	794350	13722000	15467000	12779000
3004800	861300	555870	669450	74140000	81206000	80620000
1393000	1582900	770310	429040	55816000	45303000	43989000
4976100	2053000	1572800	2843000	257400000	243140000	268670000
2457300	65130	55754	83312	239510000	331690000	312790000
1455500	2556800	2282000	3223900	0	0	0
3797200	0	0	0	57135000	55835000	56449000
4387100	1082200	646810	1348900	60301000	59618000	60234000
1821100	1019300	857000	1741000	28656000	23053000	26043000
1311800000	3412800	2737300	4072100	1,1196E+10	1,1023E+10	9935800000
94604000	76648000	59496000	88443000	1175400000	1122900000	1078000000
0	654680000	326690	233820	0	0	0
2020400	2515100	865590	1718900	66630000	79609000	96544000
599650	446950	267380	189030	0	15508000	21975000
41848000	11886000	8187100	12298000	384070000	409430000	396350000
244150	30302	16572	22122	83419000	78536000	79350000
1382500	271530	208990	390510	98936000	98610000	93255000
1949100	0	0	0	198570000	224970000	197080000
302290	219740	228860	430420	0	0	0
478870	87328	68546	106110	22892000	24444000	20446000
13659000	0	0	0	98059000	101740000	92349000
8972700	7147900	3470400	3897900	43923000	45559000	83018000
11043000	0	0	0	302350000	290290000	274890000
2502000	4549800	3741100	5087700	127220000	136950000	122400000
3965700	4437600	3534800	4294000	193770000	227150000	231900000
6875100	0	0	0	123900000	109390000	103330000
716050	0	0	0	60333000	60760000	59125000
5608600	1720300	1625600	2075200	73212000	82840000	78365000

2961900	2557700	1797300	4126100	34328000	36057000	34461000
0	562370	441720	560570	0	0	0
6657800	0	0	0	86126000	87080000	73089000
6085600	152140	222460	133410	143070000	148060000	153120000
863400	0	0	0	16666000	13762000	12982000
3718600	5378200	3826500	4617100	104720000	118130000	114900000
6217800	0	0	0	71731000	72182000	63558000
417320	987060	608630	1110500	0	0	0
3153700	2219400	2374700	3887300	50505000	52671000	59847000
416120	0	0	0	15046000	16376000	14929000
103220000	24333000	21167000	32595000	1816600000	1737000000	1699200000
0	1539700	0	0	0	0	0
4261000	1176600	999400	1559800	259970000	273690000	270100000
43493	0	0	0	0	0	3644500
963690	1507900	1219300	1749300	77904000	90547000	78983000
567590	0	0	0	9129800	9213800	9348400
4742400	262520	284730	401060	249390000	261720000	265620000
18542000	23761000	17930000	27029000	383940000	441620000	424240000
625740	310420	264970	361110	26081000	34311000	34912000
144630	0	57879	58683	0	0	0
4572800000	0	0	0	1,0646E+11	1,1059E+11	9,5951E+10
7961600	5591200	4308200	6956900	230460000	216400000	231310000
480240	0	0	0	19792000	21779000	18806000
3758900	444710	285610	597060	246930000	280120000	290470000
283970	0	50824	0	0	26015000	25500000
1033500	400530	214590	523870	13017000	15450000	14173000
35698	0	0	0	0	0	2617300
314770	196020	232440	267420	0	0	0
5074100	0	0	0	72161000	71481000	65251000
344390	220010	0	0	105060000	89403000	79214000
85121000	66203000	48491000	65168000	1286100000	1250600000	1158900000
405950	236940	137060	245510	0	0	6378000
36654000	52430000	41918000	57821000	486900000	479570000	483550000
21295000	1435100	3201800	5341000	216890000	242590000	213690000
2611700	1568100	1558500	2176500	42235000	44348000	53550000
2710000	231190	129380	327830	62655000	54516000	50622000
585610	0	0	0	35535000	36895000	34313000
3598700	4665100	2395400	4782700	33786000	41026000	41553000
24920000	0	0	0	1220500000	1282000000	1166200000
1687700	2185900	1670300	2124400	63747000	60292000	66794000
0	3352400000	0	0	0	0	0
21654000	0	0	0	422470000	330470000	339050000
129580	420350	365190	507550	0	0	0
0	18722000	0	0	0	0	0
192930	0	0	0	0	0	4618800

682570	518050	449220	798230	0	0	0
323590	0	0	0	0	0	4357700
4914800	277780	249830	322090	74173000	80295000	77248000
1718500	0	0	0	19883000	19909000	19019000
2701700	357110	417610	714130	56995000	50112000	54261000
2765600	607030	358810	588910	82233000	68802000	79248000
365450	444430	422490	496870	0	0	0
170740	0	0	0	0	0	4854100
114690000	485830000	309700000	766200000	857340000	726500000	779710000
18282000	3822800	2966100	6507800	358050000	379490000	359970000
3079300	0	0	0	103730000	103160000	89018000
0	217140	0	250330	0	0	0
111510	0	0	0	0	0	6006500
1951700	0	0	0	53934000	54663000	53767000
3440200	2848100	2763000	4096800	32766000	29088000	29720000
923050	0	0	0	19014000	17643000	18919000
596310	0	0	0	43791000	39099000	37040000
0	2809300	3651500	3360000	0	0	0
3680400	0	0	0	149030000	162840000	135900000
13159000	235730	623430	733760	226570000	172770000	274520000
8987200	5645800	5214200	8012600	108770000	108850000	106530000
18246000	786580	816690	1133100	346310000	280900000	405020000
723200	0	0	0	24353000	23277000	19564000
0	461200	397050	600610	0	0	0
336940	0	0	0	13539000	15048000	11779000
238490	0	0	0	7502800	7727300	7505100
9954000	1567800	1980600	1515300	72328000	69135000	123190000
3683800	1618800	1397200	751230	10580000	0	33999000
0	0	0	318460	0	0	0
0	193310	120440	207690	0	0	0
48934000	39534000	23542000	35171000	379100000	378050000	261060000
683720	51425	82459	118740	43940000	48005000	41687000
389570	0	0	0	35944000	36623000	34467000
16694000	1482900	1357000	1945900	1234000000	1212500000	1171800000
3157100	6333000	5669700	7742900	51482000	60929000	51081000
772460	0	0	0	51757000	57811000	51378000
235920	0	0	0	0	0	5648200
1331300	0	0	86662	115380000	113770000	105130000
2258700	0	0	0	125020000	126060000	119560000
850650	2740100	2262600	3217200	49127000	52340000	50764000
2584400	0	0	0	68919000	71571000	66293000
440670	0	0	0	30284000	33379000	32485000
582730	0	0	0	19877000	23192000	18831000
43090000	16817000	12537000	18425000	1182300000	1309800000	1214300000
1830700	75390	70963	95820	89751000	94002000	89668000

20093000	81642	52227	87397	713520000	740830000	663380000
1602100	0	0	0	33368000	37603000	27221000
11914000	961860	1486700	2894000	140160000	111890000	145990000
0	205130	204150	248410	0	0	0
1787000	0	0	0	49737000	46920000	45662000
3549600	0	0	0	111550000	97659000	105630000
1273000	0	0	0	48262000	54048000	49966000
1391400	704760	468890	596110	118810000	117330000	116830000
1070700	0	0	0	39709000	45105000	39857000
1401900	0	0	0	0	0	26220000
2686000	0	0	0	41129000	49552000	44179000
58448	0	0	0	0	0	4504000
6323200	172130	118320	147690	139200000	162040000	133000000
1312200	1205600	615150	676670	35319000	30233000	29661000
2901400	0	0	0	0	0	19537000
0	21117000	18026000	18029000	0	0	0
1687000	890690	709250	1088600	34645000	27020000	29591000
632080	0	0	0	15653000	15578000	0
0	1069300	534510	1264400	0	0	0
1274700	624410	461580	668650	0	0	13352000
1477800	0	0	0	110570000	113370000	108750000
702410	15972	11581	20535	78161000	86363000	72720000
247850	718330	0	0	9250900	0	10349000
4963800	0	0	0	280220000	267460000	251150000
455740	201300	153390	200980	32450000	34350000	30255000
102370	136990	123960	357860	0	0	0
9896.8	0	0	0	0	0	1466100
718060	0	0	0	0	0	6983900
0	1189600	37297	0	0	0	0
174680	0	0	0	0	0	4312800
626580	0	0	0	7076100	7598700	7543700
6050200	2042000	1224700	2066600	48355000	38097000	47633000
2805700	237820	156310	248880	152700000	195420000	180880000
3979400	10833000	21471000	10739000	99488000	109610000	106560000
20927000	10748000	4871700	5007400	27611000	61463000	158130000
9336200	7011600	4108900	11058000	71607000	67739000	67005000
684900	0	0	0	35947000	40802000	30067000
0	463580	461800	613540	0	0	0
7940100	1123900	929010	1197600	183320000	193310000	162680000
102970	0	0	0	0	0	1540700
257850	0	0	0	16716000	17598000	16352000
89083000	67528000	50104000	71281000	2190200000	2027500000	2031800000
2020700	0	0	0	9247100	12109000	10038000
1997600	397700	165110	577280	28997000	28078000	35695000
261800	0	68318	85794	0	0	7443000

17302000	0	0	0	108040000	132770000	109440000
646900	0	0	0	81700000	91525000	78230000
0	4380900	0	0	0	0	0
1682600	157700	0	130850	66413000	58971000	62427000
1535500	908000	492150	1080200	31723000	36225000	28310000
4263300	278320	642790	1155600	66924000	78713000	73453000
776370	0	0	0	6171400	6132600	0
1132800	0	0	0	30283000	28762000	25636000
114840000	1879800	1797100	2800400	628330000	756330000	608260000
351420	0	0	0	0	0	8939200
202670	245070	273120	230710	23492000	28096000	0
1047900	1572500	987700	1476300	0	21940000	0
198190	945790	509560	854890	0	0	0
147610	0	0	0	0	0	4859200
615190	106990	93095	86953	25612000	23248000	23568000
214650	197780	126750	303110	0	0	0
4199100	5181700	4210900	5093300	56692000	61676000	56126000
785260	0	0	0	30620000	21537000	22912000
1584800	6042800	609620	6242600	126030000	128100000	123950000
280170000	0	0	0	5899800000	6240700000	5682800000
11365000	7193400	4116700	9610300	76741000	72073000	79969000
359130	0	0	0	0	0	7523200
0	15552	3303.2	25481	0	0	0
3407500	0	0	0	324350000	334880000	314630000
1202500	0	0	0	118470000	128300000	112620000
581250	468530	493820	800440	19875000	23895000	23634000
9323800	6784000	6597500	10716000	137000000	155750000	157350000
866530	216100	0	279440	209080000	168360000	162720000
125770	0	0	0	8758000	0	7135000
772300	89054	81741	139900	16203000	18521000	15293000
3408900	0	0	0	51457000	40711000	45773000
3925700	0	0	0	41116000	41997000	40223000
5616400	5398900	3851500	6223000	308400000	326170000	280920000
1973500	440690	321450	736660	37761000	44382000	38625000
35872000	626230	577690	702260	3190000000	3146500000	2906100000
283830	429260	381270	527020	0	0	0
6285200	6476400	9695600	5577400	22660000	64938000	53554000
3691500	1152000	994140	1909700	239920000	225710000	227950000
12725000	1461900	1101500	1723700	217540000	177380000	147760000
603460	549110	567360	603440	48235000	48594000	46154000
62904000	82312000	63528000	98004000	1590300000	1737300000	1585400000
224560	0	0	0	19084000	0	14983000
1307100	822460	675730	832850	0	0	0
485310	0	0	0	0	0	5083200
198260	133640	103800	148550	0	0	0

1000400	0	0	0	11151000	9455600	10498000
603370	0	0	0	17480000	22323000	17867000
2413900	0	0	0	17013000	17150000	16636000
2537400	2743300	2623300	3469500	123510000	130120000	122990000
0	8400800	2886800	5770700	0	0	0
2313200	932690	1049300	1193300	40860000	42065000	39489000
8920500	13948000	10663000	14890000	164090000	166380000	147500000
2957300	5768800	4739000	7446700	0	0	0
2048300	0	0	0	63569000	67393000	58468000
7393900	0	0	0	112410000	114900000	117760000
314350	0	0	0	0	0	10113000
661030	641190	460750	586830	0	0	0
1066800	266450	144110	354580	41349000	36373000	36129000
1420800	0	0	0	17973000	15145000	16991000
679830	170840	98560	180610	17941000	21044000	19297000
1199300	0	0	0	116490000	117390000	112010000
794800	0	0	0	12019000	12393000	12551000
68263000	13628000	11802000	20499000	1131100000	1086300000	1024500000
0	99689	83156	133860	0	0	0
732630	0	0	0	8389800	7408300	7619000
2935700	50955	24143	45428	282030000	296290000	263590000
1815100	0	0	0	34311000	42915000	36654000
3910100	0	200580	309930	45256000	47909000	47764000
1864100	109950	138980	291100	61464000	59875000	60987000
1115200	0	0	0	136480000	151270000	139560000
917750000	1007100	1016700	1647400	1,2346E+10	1,1414E+10	1,0452E+10
1469700	0	0	0	35659000	35636000	35847000
371150	0	0	0	0	0	5275900
12415000	22532000	18658000	22234000	58954000	74563000	75537000
1480500	562380	1338400	1568500	18374000	20764000	19414000
15050000	6550200	6708400	6692300	847590000	877860000	781520000
3002100	624080	3525200	3817300	136910000	95992000	108060000
19806000	24926000	26237000	32712000	298750000	241040000	236710000
12528000	14533000	10541000	18549000	143840000	112900000	120850000
3806800	405000	410340	608330	269160000	322620000	288710000
1735000	1843200	0	1413800	0	0	0
14311000	14774000	12555000	15818000	164030000	185720000	187110000
8194400	0	0	0	134000000	129260000	124160000
15972000	923930	672040	973640	303070000	204500000	314710000
18601000	0	0	0	333740000	367170000	356160000
785030	0	0	0	29675000	33018000	26693000
5864400	3930800	2795300	3473900	74211000	77335000	76645000
6912400	1438100	1293600	1414100	21099000	25501000	25176000
4358000	516170	1860200	1980100	29304000	37971000	29887000
0	522340	413700	796560	0	0	0

1634700	0	0	0	135610000	144500000	138000000
783850	1625400	1532300	2446600	20827000	21589000	20736000
1750700	0	0	0	0	0	20957000
6563300	0	0	0	33859000	35799000	29392000
756750	164090	168300	140530	0	0	11323000
3971600	0	0	0	360070000	384610000	347550000
1895600	0	0	0	99842000	103420000	100720000
9517700	2423300	1361700	2633400	49773000	26324000	29729000
2542400	1578800	1659600	1667100	0	41324000	43470000
2581400	205980	99096	261520	51266000	54665000	58028000
189830	643860	831370	964260	0	0	0
4674800	125820	75797	156850	86245000	82140000	73868000
36749	0	0	0	0	0	2502000
26906000	21723000	15140000	21506000	99089000	95121000	99665000
9766200	279480	199610	560580	154250000	165850000	137790000
590780	0	0	0	23675000	23342000	16030000
372910	54861	0	57418	9586300	13118000	11547000
27891000	0	0	72900	1416800000	1461800000	1358600000
1639300	684700	730700	326410	0	0	14717000
2111400	1648300	1059400	1297700	20510000	26564000	28261000
3685000	11998000	11189000	18733000	170310000	168820000	161850000
7598000	3118300	5628200	6426300	49842000	52550000	45031000
227200	0	0	0	0	0	2209800
309150	0	0	0	0	0	6013600
6982000	0	0	0	141990000	139820000	128200000
28941000	3762300	3453900	4288300	1582400000	1510000000	1331000000
3954800	5317100	2583600	5436000	60984000	56721000	63317000
223000	401450	378020	513510	0	0	0
77414000	2587900	2215100	2773500	1340300000	1209500000	1187100000
158570	0	0	0	0	0	11033000
2914200	0	0	0	56031000	52627000	49234000
0	0	0	0	0	7231500	0
2044500	1222400	476850	1430200	18985000	21095000	19497000
5007800	2809700	2077000	5909500	31390000	38504000	33100000
154370	0	0	0	0	0	4735300
2585900	2561300	0	2715600	29196000	33820000	31676000
284300	0	0	0	0	0	5742800
44448000	41741000	25929000	35046000	980540000	1172500000	1308100000
1742700	0	0	0	161570000	156120000	159080000
96185	0	0	0	0	0	3310200
1882100	0	0	0	0	0	15489000
1326500	0	0	0	281060000	256350000	268610000
2390200	5122300	6378200	8001200	56601000	69371000	39577000
6415800	1754300	1205400	1387200	0	33561000	41022000
363270	550770	396490	1240600	0	0	0

1197500	0	0	0	20203000	25812000	26483000
3233900	887340	981370	769000	43668000	52479000	55545000
240690	252370	0	172760	0	0	0
0	1091600	850950	970280	0	0	0
1407900	0	0	0	20256000	21497000	19825000
377200	0	0	0	0	0	5644100
2232000	0	0	0	37061000	35948000	37711000
1481100	0	0	0	48384000	57060000	55606000
21361000	11842000	6860600	11631000	468870000	536440000	529230000
283840	0	0	0	8104400	9728400	0
454630	88008	71812	95274	19971000	22073000	19122000
831290	4732700	5502300	2273100	0	0	0
8225700	0	145870	168410	207050000	210420000	202530000
1319700	665530	408110	640790	27062000	23371000	26591000
667970	0	0	0	15944000	15918000	16552000
583380	0	0	0	12507000	16605000	0
12076000	6336800	7402700	9174200	72371000	66387000	64846000
567670	0	0	0	16610000	18554000	16672000
2662400	1524800	809210	2145300	58071000	52296000	52956000
0	55126	0	0	0	0	0
196520	0	0	0	9300900	11066000	9318600
0	231040	178430	284060	0	0	0
1879900	305440	215730	332490	97231000	122790000	103090000
4186000	0	3877.3	27779	282010000	269940000	268150000
1382800	0	0	0	57720000	60007000	60728000
1301500	255910	179660	249770	41143000	36781000	44019000
2335700	0	0	0	72243000	66809000	68395000
15495000	0	0	0	335270000	311400000	273640000
29181000	11444000	4038000	10086000	375550000	349920000	381130000
5988000	6524600	4534000	7013700	483620000	538570000	531280000
28420000	3251500	2555100	5631000	573080000	551380000	524080000
0	0	0	0	0	0	0
746010	0	0	0	68089000	66026000	54292000
749560	0	0	0	24446000	23092000	20874000
15465000	10464000	6042200	16179000	252180000	278690000	281640000
7810100	3907500	2860100	6661200	160490000	140610000	123760000
20595000	11980000	9287200	17957000	551630000	595760000	588610000
39396000	5419800	4186900	6418100	698270000	735570000	677120000
49477000	0	0	0	1239600000	1293400000	1146100000
58284000	36021000	27052000	39930000	695820000	695300000	707810000
580480	0	0	0	0	0	4777200
4656500	16565000	13116000	17719000	74547000	84353000	79773000
1887700	928430	512180	1072200	0	0	11298000
547750	255220	326310	421830	9839700	11873000	9853000
38008000	8580400	6218600	8832000	70032000	80085000	77990000

5924500	4229600	3126100	4735000	29941000	38712000	48188000
1048200	2385900	1401000	2338400	71006000	67553000	68447000
2724200	0	0	0	225870000	228520000	216500000
2207000	3324100	1910600	1931700	89969000	106340000	86363000
12294000	628490	499980	814830	389150000	357340000	337620000
9610000	1799900	1853300	2963900	328840000	377930000	278560000
1407900	561610	1308600	222280	53570000	58274000	52270000
1496700	213410	195770	244110	25753000	29124000	27668000
1277500	760820	0	0	0	0	0
NaN	NaN	NaN	NaN	0	0	0
NaN	NaN	NaN	NaN	0	0	0
NaN	NaN	NaN	NaN	0	0	23814000
NaN	NaN	NaN	NaN	0	0	0
NaN	NaN	NaN	NaN	0	0	0
NaN	NaN	NaN	NaN	0	0	41779000
NaN	NaN	NaN	NaN	0	0	0
NaN	NaN	NaN	NaN	0	0	5841000
NaN	NaN	NaN	NaN	0	0	0
NaN	NaN	NaN	NaN	0	0	0
NaN	NaN	NaN	NaN	0	0	43913000
NaN	NaN	NaN	NaN	11116000	0	0
NaN	NaN	NaN	NaN	0	0	0
NaN	NaN	NaN	NaN	0	0	11434000
NaN	NaN	NaN	NaN	0	0	0
NaN	NaN	NaN	NaN	0	0	0
NaN	NaN	NaN	NaN	0	0	0
9215400	0	0	0	0	9029000	33945000
2995900	2629200	1887000	2829400	138920000	149570000	147840000
11171000	6074700	3525700	4959600	61502000	65682000	68835000

LFQ intensity	LFQ intensity	LFQ intensity	MS/MS Coun	MS/MS Coun	MS/MS Coun	MS/MS Coun
30598000	27450000	38513000	3	2	4	1
0	0	0	1	1	3	1
0	0	0	0	2	2	1
261770000	246450000	303050000	3	2	3	8
70610000	73549000	60164000	3	4	5	4
0	0	0	1	1	2	0
0	0	0	1	1	0	0
265060000	307860000	259220000	5	6	7	6
76937000	69553000	64711000	2	3	5	5
0	0	0	2	0	1	0
21237000	36493000	36954000	1	2	2	1
18654000	22400000	0	0	1	1	1
56312000	89586000	65068000	3	2	3	4
0	0	3070800	0	0	1	1
0	0	0	1	2	1	0
0	0	0	1	0	0	0
101740000	143040000	112130000	7	3	6	4
0	0	0	1	0	0	0
50131000	69662000	71368000	19	17	16	3
26409000	34415000	34793000	3	2	6	2
771820000	911800000	740610000	5	7	8	5
0	0	17347000	0	1	0	0
549190000	665460000	758300000	17	17	17	18
0	0	0	2	1	1	0
0	0	0	11	7	8	0
0	0	4910600	1	0	1	1
27290000	25807000	28543000	4	4	6	1
0	0	0	0	0	0	0
0	0	0	3	5	2	0
0	0	0	7	5	6	0
0	0	0	11	10	11	0
0	0	0	1	0	0	0
0	0	0	1	0	1	0
0	0	14916000	1	1	1	1
24470000	28790000	23711000	1	2	3	1
0	0	0	1	0	0	0
42190000	48362000	72478000	5	4	4	2
0	0	0	0	2	1	0
0	0	0	1	0	0	0
44814000	58670000	44577000	4	4	3	2
0	0	0	1	1	1	0
0	0	0	13	8	12	0
0	0	18607000	1	0	0	0
71420000	110650000	78361000	0	2	1	2

39090000	47532000	57638000	6	6	6	1
19550000	25762000	0	1	1	1	1
13593000	15260000	23348000	0	0	2	0
0	0	0	1	0	2	0
0	0	0	6	6	3	0
0	0	0	1	2	1	0
0	0	0	0	0	0	0
26794000	17042000	18109000	0	0	1	3
57390000	30043000	38085000	4	4	3	4
0	0	0	5	1	2	0
0	0	0	4	4	6	1
0	0	0	2	1	1	1
72058000	69561000	82077000	6	7	10	4
0	0	0	0	0	0	0
1286600000	0	0	0	0	0	13
0	0	2715700	0	0	0	0
91987000	100130000	149660000	2	0	3	0
42723000	38847000	43638000	3	4	3	2
0	0	0	0	0	1	0
347810000	382670000	393460000	8	10	11	5
0	0	0	21	21	22	0
0	0	0	2	4	2	3
0	0	181100000	1	1	2	2
0	0	0	2	1	2	0
0	0	0	3	4	4	0
2712900000	2368500000	3170500000	24	24	28	31
0	0	0	1	2	2	0
839710000	1103600000	1028300000	5	5	4	2
54464000	77360000	86207000	7	6	7	2
0	0	0	1	1	0	0
0	0	0	1	0	0	0
0	0	0	1	1	1	0
0	0	0	9	9	8	0
103720000	147010000	126950000	5	5	6	1
0	0	0	2	3	2	1
0	0	0	0	0	1	0
43415000	31434000	36006000	4	4	4	2
59205000	47744000	64694000	1	3	2	2
0	0	0	1	1	2	0
180620000	209580000	218650000	6	6	9	3
0	0	0	2	3	1	0
0	0	0	3	5	5	0
0	0	0	2	1	6	1
0	0	0	1	2	1	0
0	0	0	1	2	2	0

15344000	14904000	21589000	0	0	0	0
64507000	62290000	67537000	0	3	3	0
0	0	39221000	1	1	2	0
0	38890000	37244000	1	0	1	0
0	0	0	5	5	5	0
0	0	14197000	1	1	1	1
543170000	451870000	451090000	13	10	12	13
0	0	0	1	0	0	0
56744000	55286000	51213000	2	3	2	3
0	0	0	2	3	4	0
0	0	0	1	0	0	0
44592000	33838000	41687000	10	8	8	1
0	0	0	1	1	1	1
58816000	82080000	78605000	3	0	1	4
33617000	40651000	38492000	3	3	3	4
0	0	0	23	17	23	0
0	0	0	0	2	0	0
514980000	358520000	547180000	5	6	11	12
124650000	159520000	127570000	5	4	6	3
14221000	0	0	0	0	0	2
0	0	0	3	2	3	0
34702000	0	0	4	5	4	1
135400000	136160000	162150000	4	6	8	2
0	0	0	0	1	1	1
0	0	0	2	3	3	0
0	0	0	2	1	1	0
0	32068000	28365000	1	2	2	1
139870000	141580000	186340000	9	8	8	3
144960000	151120000	162200000	12	12	10	5
466060000	609300000	598350000	13	14	15	8
0	0	0	2	2	1	0
0	0	0	0	3	1	0
0	0	0	1	0	1	0
0	0	0	1	1	0	0
0	0	0	1	1	1	0
0	0	0	2	0	0	0
0	0	0	1	0	0	0
0	0	5960100	1	0	1	1
3493700000	4365100000	3952000000	142	141	164	57
0	0	10164000	0	0	0	0
0	0	0	3	3	3	0
0	0	16941000	1	1	0	1
0	0	0	1	0	0	0
561600000	527620000	516300000	21	19	21	14
0	0	0	1	0	0	1

0	0	26954000	1	0	1	1
71646000	90229000	68002000	12	9	11	3
531320000	522160000	597560000	6	6	6	4
35586000	47373000	74419000	1	1	1	1
0	0	0	2	0	2	0
0	0	11393000	1	0	1	1
0	0	0	2	1	1	0
0	0	0	10	10	9	0
0	0	0	1	1	1	0
33935000	24438000	28824000	1	0	1	2
0	0	0	6	5	5	0
0	0	0	2	3	2	0
0	0	0	1	2	2	1
0	0	0	3	2	2	0
35726000	36681000	36126000	1	1	1	2
0	0	0	1	1	1	0
505760000	399330000	406550000	10	10	12	17
0	0	0	1	1	2	1
221400000	242250000	245580000	14	22	16	12
72685000	130380000	121840000	18	24	24	2
0	0	0	1	3	1	1
0	0	7092000	0	1	0	1
32435000	39883000	27335000	3	3	4	1
1475500000	1457800000	1828800000	18	17	19	10
0	0	0	0	0	1	0
0	0	0	2	3	3	0
0	0	0	13	11	14	0
0	0	0	0	1	0	0
151070000	184290000	188770000	4	6	7	3
0	0	4361900	1	1	1	0
0	0	0	2	0	1	0
0	0	0	1	2	2	0
0	0	0	3	3	3	1
0	0	0	3	4	3	0
16098000	13761000	12356000	0	1	0	2
0	0	0	0	0	2	0
0	0	0	1	0	1	0
59167000	0	45541000	8	5	2	1
0	0	0	7	4	7	0
0	20118000	19943000	3	2	3	0
0	13900000	14590000	1	2	2	1
0	0	0	0	1	1	1
119110000	156330000	129420000	7	5	7	3
104170000	47081000	34704000	0	0	0	3
408230000	467100000	399160000	7	10	7	7

645320000	883390000	752040000	8	7	7	13
151420000	156810000	155590000	5	3	5	10
194190000	170150000	193940000	6	6	6	5
0	0	0	1	2	2	0
61869000	0	0	0	0	0	3
0	0	0	4	4	3	0
85060000	44396000	61803000	9	9	11	2
3367400000	4324500000	4622300000	17	19	17	16
0	0	0	3	2	4	1
0	0	0	5	3	4	0
0	33201000	34269000	5	4	4	0
0	0	0	0	1	1	0
0	0	0	5	5	4	0
0	0	0	1	0	1	0
0	0	0	9	8	11	0
0	0	0	1	0	1	0
0	24564000	21190000	8	5	8	0
0	0	0	2	1	1	0
0	0	0	1	1	1	0
133180000	161960000	146450000	17	18	20	3
0	0	0	7	6	8	1
1786400000	1134600000	871610000	9	10	21	25
0	0	0	2	1	0	0
2590000000	2162400000	3307800000	28	29	30	31
0	0	0	2	0	2	0
188170000	146260000	184360000	7	8	8	5
9600400	9760800	12033000	0	0	0	1
751040000	794910000	1053700000	5	4	5	4
258640000	266500000	350440000	6	3	5	6
51129000	85191000	105590000	7	8	6	0
1479500000	886380000	658970000	10	12	16	19
71343000	91828000	126660000	4	4	3	3
36785000	41327000	39683000	3	3	1	1
180290000	215550000	207700000	16	17	12	8
0	0	8135600	0	0	0	0
0	0	10367000	1	1	1	0
0	0	0	1	1	2	0
932680000	1155200000	1021800000	16	20	22	11
18740000	26660000	26033000	2	1	1	2
75292000	67576000	67192000	4	4	5	5
448970000	615970000	478560000	7	6	8	2
0	0	3720700	0	0	0	1
43686000	34180000	40625000	3	1	5	3
0	0	6391900	1	1	1	0
183840000	184820000	214200000	8	11	10	3

167990000	169970000	206870000	6	5	7	4
0	0	0	1	1	0	0
0	0	0	0	1	0	0
0	0	0	5	3	5	0
0	0	0	6	8	6	0
0	0	0	5	3	6	1
62735000	91451000	62474000	1	1	1	2
0	0	7872400	1	1	0	0
0	0	0	1	1	3	0
29070000	29026000	36823000	0	0	0	2
0	0	0	6	5	3	0
0	0	0	0	1	0	0
32101000	26903000	26579000	0	0	0	1
0	0	0	2	4	1	0
0	0	0	1	2	1	0
0	0	0	5	6	6	0
33615000	32835000	23760000	1	2	2	2
67956000	41248000	52725000	5	5	5	3
0	0	0	0	2	1	0
46254000	0	15407000	38	35	42	0
0	0	0	2	3	4	0
0	0	0	4	9	5	0
0	0	22209000	0	0	1	1
0	0	0	1	1	1	0
0	0	0	0	1	1	0
0	0	0	1	0	0	0
11969000	21340000	15891000	1	0	1	1
0	0	0	1	1	1	0
0	0	0	5	6	4	0
0	0	0	1	0	1	0
0	0	0	0	0	1	0
0	0	0	2	2	2	0
55171000	62841000	83912000	0	0	0	3
307170000	229770000	236590000	13	9	13	10
0	0	20398000	0	2	1	1
0	0	44522000	0	0	1	1
0	0	0	0	0	2	1
0	0	0	2	0	2	0
0	0	0	2	2	3	0
0	0	0	1	0	1	0
42011000	42327000	44009000	8	8	10	1
57453000	46579000	95219000	0	0	0	1
0	0	0	12	9	9	0
0	0	0	1	0	1	0
197600000	261630000	184290000	2	2	3	3

0	0	0	1	2	2	0

MGZ8;Q8HWT0;Q75WB6;Q75NY8;Q75NJ3;Q70I55;Q6L6T0;Q6L6R5;Q6L6Q3;Q6L6P9;Q6L676;Q6L675;Q6L625;Q6I6G7;Q6I6

;F8R141;F8R140;F8R139;F8R138;F8R137;F8R136;F8R135;F8R134;F8R133;F8R132;F8R131;F8R130;F8R129;F8R128;F8R126;

;Q8MHP7;Q8MGZ8;Q8HWT0;Q75WB6;Q75NY8;Q75NJ3;Q70I55;Q6L6T0;Q6L6R5;Q6L6Q3;Q6L6P9;Q6L676;Q6L675;Q6L625

;F8R141;F8R140;F8R139;F8R138;F8R137;F8R136;F8R135;F8R134;F8R133;F8R132;F8R131;F8R130;F8R129;F8R128;F8R126;

3;273;273;273;273;273;273;273;273;273;273;273;273;273;273;273;273;273;273;273;273;273;273;273;273;273;273;2

86418000	60381000	66520000	5	2	2	3
0	0	0	6	9	8	0
0	0	0	0	0	0	0
0	0	0	0	0	2	0
0	0	0	0	1	1	0
0	0	0	1	0	1	0
14865000	15379000	43896000	1	2	1	1
0	0	0	2	3	4	0
15679000	0	0	0	0	0	1
0	0	41451000	0	0	2	0
314210000	335180000	365490000	12	16	14	7
0	0	0	0	0	1	0
0	0	0	0	1	1	1
0	0	0	1	1	2	0
0	0	0	2	2	3	0
0	0	0	1	0	1	0
0	0	0	3	4	4	0
0	0	0	1	2	1	0
0	0	0	2	1	1	1
7845400000	8798800000	8336000000	152	162	174	146
222570000	82109000	79863000	1	1	2	9
0	0	0	1	1	1	0
75181000	68502000	76659000	3	2	3	2
0	0	0	3	3	3	0
0	0	0	1	2	2	0
0	0	0	1	1	1	0
0	0	0	0	2	0	0
0	0	0	0	1	1	0
0	0	0	0	1	1	0
164720000	166670000	267010000	2	1	1	1
0	0	0	4	4	2	0
152270000	138610000	176330000	0	0	0	6
22886000	22260000	21373000	1	0	0	1
0	0	0	1	0	0	0
0	0	0	1	1	1	0
0	0	0	1	0	1	0
0	0	6004900	1	1	1	1
0	0	6420400	1	1	1	0

41146000	40489000	48294000	3	2	4	3
0	0	0	0	0	1	0
0	0	0	1	0	1	0
0	0	4871400	0	0	0	0
0	0	0	5	3	5	0
0	0	24044000	0	1	1	0
7858300	0	0	0	0	0	1
0	0	0	1	0	0	0
0	0	0	1	1	0	0
41555000	39312000	40764000	4	4	4	2
79953000	52424000	53180000	2	1	3	3
113890000	120500000	140360000	7	9	15	4
0	0	4506300	0	0	0	0
42053000	49290000	48997000	3	3	3	4
0	0	0	2	2	2	1
0	0	0	0	0	1	0
0	0	0	1	1	0	0
0	0	8715600	1	1	0	0
37031000	46855000	58119000	1	3	4	1
1041500000	0	0	0	0	0	6
0	0	0	2	2	4	0
167860000	230330000	181020000	5	7	5	5
0	0	6021100	1	1	1	0
0	0	0	1	3	2	1
0	0	0	5	5	4	0
0	0	0	1	1	1	0
0	0	0	1	1	1	0
1017500000	1157700000	766150000	10	12	15	16
0	0	0	2	3	5	0
0	0	0	2	2	3	0
0	0	22096000	0	0	1	0
0	0	0	0	0	1	0
0	0	0	2	1	3	0
51394000	66642000	58452000	1	1	1	2
0	0	3138200	0	0	1	0
0	0	0	1	0	1	0
67186000	95985000	77398000	7	7	7	3
0	0	0	56	57	58	0
0	0	5740600	0	0	0	0
32234000	34873000	40349000	2	0	0	1
0	0	0	3	2	2	0
0	0	0	0	1	1	0
0	0	0	1	1	0	0
0	17267000	17442000	1	2	1	1
0	0	0	0	1	0	0

0	0	0	3	2	3	0
45781000	35820000	54929000	0	2	1	3
0	0	0	2	0	1	0
531460000	640930000	691470000	7	7	10	8
0	0	0	1	1	1	0
0	0	8285700	0	0	0	1
0	0	0	2	0	1	0
0	0	0	0	1	0	0
0	48067000	50820000	7	9	7	1
0	0	11249000	1	1	1	1
161590000	197470000	178690000	6	6	7	4
205770000	196690000	237720000	10	8	9	10
76007000	73153000	100170000	4	2	3	5
225620000	261380000	249580000	24	21	24	5
31131000	31259000	30617000	2	1	2	1
0	0	0	4	3	2	0
133800000	145570000	164090000	1	3	1	4
0	0	0	1	0	1	0
1853700000	2044100000	2220400000	31	28	30	21
0	0	0	4	3	7	0
77675000	111760000	84674000	2	3	4	3
0	0	0	0	1	2	0
0	0	0	1	0	3	0
38347000	51402000	44180000	4	1	3	2
223820000	225590000	222060000	28	22	27	8
0	0	0	0	1	1	0
1680900000	0	0	0	0	0	4
35559000	22589000	26829000	9	4	8	2
0	0	0	1	1	1	0
0	0	0	10	8	8	0
0	0	0	0	1	0	0
0	0	5062500	1	0	1	0
97512000	101080000	109010000	3	5	3	4
0	0	0	1	0	1	0
57700000	53545000	47651000	1	3	3	2
0	0	0	1	2	0	1
18180000	25947000	26754000	0	2	3	0
31656000	26887000	29383000	0	0	0	2
0	0	0	3	0	1	0
0	0	0	1	0	0	1
84245000	71653000	94100000	3	4	3	4
0	0	0	0	0	3	0
0	0	0	1	0	0	0
211250000	269200000	271930000	7	6	8	6
217840000	183180000	201070000	4	5	5	3

0	0	0	9	6	5	0
0	0	0	5	3	3	0
0	0	106860000	3	3	4	0
0	0	8188400	0	0	0	0
95374000	101720000	121520000	11	9	9	2
0	0	7804500	1	0	1	0
129540000	147190000	146470000	5	6	9	10
48928000	122590000	67328000	2	2	2	2
0	0	0	1	0	0	0
150500000	131280000	157500000	6	5	6	3
0	0	0	3	3	2	0
0	0	0	1	1	1	0
0	0	0	1	3	2	0
0	0	0	0	1	2	0
0	0	0	3	3	4	0
0	0	0	1	1	0	0
0	0	3999900	0	1	1	0
0	0	29372000	1	1	1	1
0	0	0	2	2	2	0
0	355130000	130020000	0	0	0	0
192150000	0	0	0	0	0	3
245560000	297530000	297480000	18	14	19	7
0	0	0	2	2	2	0
0	0	0	12	10	11	0
28104000	35314000	34107000	2	3	1	0
44832000	42226000	46912000	1	1	2	2
0	0	0	0	0	2	0
32783000	0	0	0	0	0	2
101550000	136600000	134770000	14	15	17	4
0	0	0	1	0	1	0
0	0	10163000	0	0	0	1
0	0	0	3	4	3	0
0	0	0	1	0	1	0
0	21272000	33601000	0	0	0	0
0	0	0	3	5	2	0
0	0	0	3	3	3	2
0	0	0	1	1	2	0
0	0	0	1	2	2	1
0	28152000	23098000	3	3	3	1
60840000	77184000	66911000	1	2	4	4
0	0	0	0	0	1	0
0	0	0	1	2	0	0
726660000	20090000	12257000	1	1	2	3
0	0	0	5	2	4	0
0	0	86741000	1	1	1	1

22972000	24481000	31892000	7	5	7	0
30785000	32076000	35135000	2	2	2	2
0	0	0	3	2	2	0
0	0	38706000	1	1	1	1
0	0	0	0	1	1	0
264190000	277400000	305730000	9	10	10	6
0	0	0	0	2	2	0
0	0	0	2	3	3	0
56639000	54476000	172330000	0	0	1	0
0	0	51652000	1	1	1	0
0	0	0	1	1	1	0
0	0	0	5	2	4	0
630730000	765780000	758370000	20	18	23	17
0	0	0	2	1	3	0
0	0	0	0	1	3	0
0	0	3129600	0	0	0	1
699610000	436550000	385000000	8	8	8	13
0	0	10905000	1	1	1	1
0	0	0	0	1	1	0
227200000	245450000	227000000	7	7	10	5
90928000	72354000	75855000	5	6	5	3
0	0	0	2	2	0	0
35533000	44257000	34962000	4	2	1	0
53729000	42455000	36711000	2	1	3	0
0	0	0	1	1	1	0
84856000	107400000	115410000	7	6	8	4
0	0	12856000	0	0	0	0
55831000	64140000	59784000	2	3	4	2
246230000	258720000	320300000	8	10	8	13
208710000	160330000	107160000	5	4	3	10
111160000	111900000	120600000	18	17	18	5
17026000	10197000	13411000	1	1	0	2
221900000	248470000	288710000	7	7	7	6
0	0	0	0	1	1	0
0	0	0	2	1	2	0
0	0	1922900	0	0	1	1
0	0	4947800	1	1	1	1
0	0	0	4	3	6	0
60897000	70078000	80641000	7	6	4	3
0	0	0	4	3	5	0
0	0	0	0	0	2	0
0	0	0	4	3	4	0
0	0	0	1	1	2	0
279660000	347340000	323240000	5	6	5	9
0	0	0	1	1	1	0

16980000	14306000	26048000	1	0	2	2
0	0	4578400	0	1	0	0
0	0	0	4	3	3	0
23090000	25985000	27606000	2	1	2	1
44790000	38822000	36228000	1	2	3	3
1167900000	0	0	0	0	0	6
0	0	0	2	1	2	0
184880000	207380000	211170000	6	1	4	6
0	0	0	0	0	1	0
0	0	0	1	2	3	0
29714000	37469000	38169000	1	0	0	2
154980000	138010000	206600000	6	8	7	5
323470000	281840000	285940000	8	8	8	7
347800000	356990000	319060000	21	22	24	11
8590200	10178000	10757000	1	0	0	0
55690000	0	0	5	6	8	2
0	0	0	1	1	1	0
0	0	0	1	0	0	0
0	0	0	1	1	1	0
0	0	0	2	2	2	0
0	0	0	4	3	3	0
131440000	112610000	115510000	9	7	7	4
0	0	0	2	0	0	0
0	0	0	1	1	0	0
0	9578500	22724000	1	0	1	1
1656000000	2009100000	1932500000	69	62	71	21
0	0	3754700	1	1	1	0
0	0	0	5	6	3	0
23359000	16347000	42236000	12	8	10	0
0	0	6147300	1	1	1	0
0	0	0	2	1	2	0
0	0	0	5	5	7	1
0	0	0	0	1	0	0
0	0	19652000	3	3	2	0
198460000	163030000	146240000	3	4	2	3
0	0	0	0	0	1	0
0	0	0	1	1	2	0
159210000	177560000	194980000	3	2	3	6
0	0	0	2	1	1	0
16260000	17338000	18450000	1	1	0	1
0	0	5084200	0	0	0	0
200330000	238330000	233220000	11	9	14	3
114410000	90962000	86616000	1	5	5	2
0	0	7429900	0	0	0	1
449820000	483260000	480320000	9	10	7	6

58881000	0	0	2	2	3	1
0	0	0	2	1	1	1
720970000	618920000	757310000	11	8	15	13
0	0	0	2	4	3	1
2045800000	3079700000	2417800000	15	18	16	15
0	0	0	1	1	1	0
0	0	96950000	2	2	2	1
0	0	0	5	5	7	0
190610000	182350000	154210000	1	1	2	8
0	0	0	0	0	1	0
403200000	502420000	532980000	8	8	8	7
437050000	542280000	564580000	13	14	12	15
0	0	0	2	1	0	0
0	0	0	2	1	2	1
0	0	0	1	2	2	1
0	33322000	15781000	1	0	0	0
0	0	14049000	1	1	1	1
44586000	50710000	54869000	10	9	7	2
62617000	0	0	0	0	0	2
0	0	0	0	0	1	0
0	0	0	8	4	5	0
0	0	0	0	0	0	0
0	0	0	0	0	0	0
0	0	0	1	0	0	0
0	0	35894000	9	8	7	1
0	0	0	0	0	1	0
737170000	761610000	967120000	3	2	6	1
33110000	0	35251000	2	2	2	1
113980000	131580000	137990000	6	5	6	4
0	0	0	5	5	3	0
0	0	0	4	4	3	0
0	0	0	0	0	1	0
0	0	0	0	0	0	0
0	0	0	1	5	6	0
52311000	58910000	51726000	6	5	5	1
0	0	17500000	1	0	0	0
304130000	345240000	328320000	13	16	15	10
0	60572000	51297000	1	1	1	1
0	32874000	23663000	2	3	2	1
0	0	0	1	1	2	0
422630000	401630000	597250000	9	9	11	7
0	0	0	4	4	4	1
472630000	203870000	141220000	26	25	25	7
0	0	0	2	2	2	0
485780000	600280000	575450000	11	18	19	7

42944000	0	53077000	2	4	4	0
0	0	0	14	10	13	0
0	0	0	9	11	10	0
0	0	0	27	24	27	0
0	0	0	3	2	4	0
0	0	1824300	1	1	1	0
0	34773000	24109000	4	3	6	0
0	0	0	6	5	4	1
0	0	0	6	7	6	0
146440000	167030000	140980000	4	5	5	7
30992000	43953000	62155000	1	1	1	2
313570000	462170000	421580000	16	15	18	11
0	0	0	13	16	17	0
0	0	0	0	0	1	0
30777000	32491000	34571000	0	3	4	1
0	0	0	1	0	0	0
0	0	0	4	3	3	0
128890000	136760000	134450000	2	2	1	2
0	0	0	1	1	0	0
0	0	0	8	8	8	0
0	0	0	0	0	1	0
0	0	0	0	0	1	0
192680000	226000000	256650000	8	6	9	9
188700000	156540000	225290000	7	7	6	2
0	0	0	3	3	3	1
76526000	79742000	85899000	2	3	2	2
0	0	0	1	2	2	0
0	0	0	0	0	1	0
0	0	0	1	0	1	0
8946400	0	15478000	0	0	0	0
68714000	99604000	96657000	8	4	5	3
0	0	0	3	3	4	0
0	0	0	0	0	1	0
0	0	0	1	1	3	0
0	0	0	2	2	2	0
0	0	0	8	3	3	0
25507000	20693000	34631000	2	1	1	2
0	0	0	1	1	1	0
0	0	22163000	1	1	1	1
0	0	4690500	1	1	1	0
50799000	0	0	0	0	0	2
19219000	23463000	25208000	2	3	3	3
0	0	0	1	1	2	0
10791000	0	11711000	18	14	15	0
0	0	0	3	2	3	0

101280000	103470000	88050000	4	10	14	7
0	0	23249000	0	0	0	0
57998000	72484000	65152000	6	8	9	2
0	0	9344100	1	1	1	0
0	0	6812700	0	0	0	1
111010000	0	28799000	1	1	1	4
0	0	0	1	2	1	0
11368000	14757000	13579000	0	1	1	0
0	0	0	2	1	1	0
0	0	12908000	2	0	0	0
0	0	0	10	10	9	0
0	0	11718000	0	0	0	1
0	0	14124000	3	3	3	0
10577000	0	0	0	0	0	1
0	0	0	2	1	3	0
74241000	87847000	80223000	5	3	4	5
0	0	0	1	0	1	0
0	0	23011000	1	1	1	1
0	0	3428100	0	0	0	0
0	29608000	24366000	1	0	1	0
0	0	0	2	0	2	0
0	0	11414000	1	0	0	0
0	15926000	13432000	1	1	1	0
0	0	0	1	1	1	0
74527000	97799000	87344000	3	2	5	5
0	0	0	0	1	1	0
24368000	20095000	41453000	3	3	4	2
0	0	7793100	0	0	0	0
95352000	116640000	106290000	7	7	7	5
0	0	0	1	1	2	1
22139000	0	27958000	0	0	0	1
82932000	86919000	82337000	0	0	0	2
0	0	0	1	3	1	0
0	0	0	1	1	2	0
0	0	0	6	4	7	1
198980000	231090000	268680000	3	4	3	5
0	0	0	2	2	4	0
0	0	0	2	2	0	0
0	0	0	2	1	3	0
0	0	0	0	0	0	0
0	0	0	2	2	1	0
0	0	0	1	1	1	0
0	0	0	7	7	7	0
0	0	0	0	1	1	0
0	0	0	0	0	1	0

0	0	0	2	2	2	0
8824600	0	13447000	0	1	1	1
0	0	0	5	6	5	0
0	0	3913800	0	0	0	1
0	0	0	0	0	1	0
38834000	36411000	35610000	2	1	2	2
32349000	29251000	12739000	1	0	1	2
22055000	31128000	22454000	2	2	2	2
28380000	38096000	37558000	20	17	14	1
0	0	0	3	3	3	0
32004000	35379000	53393000	0	0	3	2
0	0	0	3	3	2	0
33617000	21818000	21521000	1	1	2	2
0	0	0	1	1	0	0
0	0	0	0	0	1	0
0	19160000	23616000	0	1	1	0
0	0	0	0	1	0	0
285610000	222900000	270200000	7	7	8	6
83691000	59027000	43987000	4	5	2	5
0	0	0	1	0	0	0
0	0	0	3	2	2	0
0	0	3671900	0	1	1	0
0	0	0	0	2	0	0
0	0	0	13	13	13	0
120930000	96288000	122400000	14	18	15	4
0	0	0	4	2	5	0
45453000	62134000	61285000	0	0	0	1
67511000	49426000	93857000	8	6	7	1
40701000	40244000	59195000	1	2	0	2
104470000	136710000	111890000	4	4	4	3
183710000	179490000	220070000	14	11	11	5
0	0	36231000	0	1	0	0
0	0	33248000	1	1	1	1
0	0	0	1	1	1	0
0	0	0	3	1	1	0
64064000	0	57166000	0	0	0	1
0	0	0	0	0	1	0
0	0	7195200	1	1	1	1
18432000	14443000	23501000	3	3	1	1
143970000	142900000	172700000	16	15	14	8
0	0	0	1	2	1	0
0	0	0	0	1	1	0
0	0	0	1	0	1	0
0	0	1606100	0	0	0	0
0	0	5184300	0	1	0	0

0	0	0	2	2	2	0
0	0	0	1	1	2	0
0	0	5238500	0	0	0	0
102840000	80627000	81414000	4	5	3	2
143240000	154320000	139070000	21	16	20	4
0	0	0	1	0	0	0
0	0	0	2	2	3	0
0	0	0	0	0	1	0
95764000	98571000	83608000	3	2	2	3
0	0	0	2	4	4	0
0	0	0	2	4	5	0
39482000	37536000	40392000	2	4	3	1
0	31705000	26954000	7	5	7	0
0	0	0	2	2	2	0
0	5855800	0	0	0	0	0
0	0	0	2	0	1	0
0	0	0	1	1	1	0
0	0	0	6	3	4	0
0	0	0	1	1	0	0
0	0	0	8	6	7	0
0	0	0	22	30	29	0
0	0	0	1	1	1	0
65422000	32539000	38068000	1	2	4	3
0	0	13528000	1	1	1	1
0	0	0	11	9	13	0
0	35826000	36282000	0	2	1	1
0	0	11778000	0	0	0	1
0	0	0	1	1	1	0
0	0	0	1	1	1	0
0	0	0	2	2	2	1
0	0	15990000	0	0	0	0
0	0	0	2	3	3	1
0	0	0	1	1	2	0
0	0	6936600	1	1	0	0
0	0	0	2	2	1	0
0	0	0	2	2	2	1
19306000	38778000	31352000	15	19	18	1
0	0	4396200	0	1	1	1
26512000	14399000	10502000	0	1	2	2
0	0	0	1	1	1	0
81399000	94931000	116390000	4	5	5	2
68974000	36588000	66378000	3	3	3	3
0	0	6159400	0	0	0	1
55856000	39233000	50664000	6	6	7	2
0	0	0	4	3	4	0

527460000	0	0	0	0	0	7
84530000	0	0	0	0	0	2
68646000	66550000	63244000	3	2	1	2
0	0	0	1	1	0	0
############	0	0	0	0	0	5
153190000	0	0	0	0	0	1
4458500000	5539200000	5656800000	16	9	13	14
4630000000	751400000	581040000	23	14	18	34
0	6593700	0	0	0	0	0
443480000	7434900000	594130000	5	5	11	3
27786000	2357200000	35591000	1	0	0	0
73129000	252300000	63620000	2	0	5	1
31501000	613140000	26253000	2	2	4	2
112180000	848920000	139890000	3	2	8	1
1,6054E+11	1,7774E+11	1,6064E+11	203	219	218	201
0	0	0	0	1	0	0
4578900000	299830000	326650000	10	6	17	30
78654000	174380000	127940000	7	6	3	0
4486500000	4965600000	4798100000	25	29	34	49
5334300000	97211000	150640000	5	4	8	28
1,5426E+10	1,3208E+10	9883600000	62	44	84	59
1235900000	684440000	394050000	11	7	13	8
0	0	23245000	0	0	0	1
0	0	1932200	1	0	0	1
7922700000	5567900000	2639700000	46	62	44	43
0	0	0	1	0	0	0
4484500000	29729000	0	0	0	0	9
438810000	45907000	54353000	4	5	5	8
1,0473E+10	7122300	0	0	3	0	27
63236000	140240000	86987000	4	4	6	2
0	0	4883900	1	1	1	0
0	0	18843000	0	0	0	1
83672000	0	0	1	1	2	2
0	0	0	9	7	6	1
0	0	0	1	0	1	0
190410000	0	0	0	0	0	4
70958000	51725000	35835000	3	2	6	2
0	0	0	0	1	0	0
0	0	32542000	1	1	1	1
2942600000	0	0	0	0	0	13
7177800	0	0	0	0	0	1
0	0	0	2	1	1	1
2,1567E+10	12184000	3769800	0	2	0	38
0	26200000	19129000	0	0	4	1
0	0	0	0	0	0	0

0	0	0	0	0	8	1
283920000	256150000	153960000	7	13	10	9
6227300000	0	0	0	0	0	7
0	1895600	0	0	0	0	1
0	0	0	11	8	7	0
639770000	0	0	0	0	0	1
487550000	0	0	0	0	0	4
241380000	274350000	248150000	7	7	10	8
0	0	0	8	7	6	0
0	0	0	2	1	2	0
0	0	0	0	1	1	0
0	0	0	2	3	2	0
0	0	5209400	1	1	1	0
67061000	0	64200000	3	3	4	2
83571000	81423000	93377000	3	4	5	5
0	0	0	2	2	2	0
6684600	7215100	8463100	3	0	3	0
26420000	16021000	25967000	0	0	0	1
32646000	31234000	36486000	1	1	1	1
23363000	0	0	0	0	1	1
0	0	0	3	4	2	0
38149000	33830000	35379000	4	3	3	0
35534000	28681000	40118000	0	2	1	1
0	0	0	5	5	6	0
218790000	293650000	273740000	7	9	13	11
82368000	92790000	115000000	2	1	3	3
0	0	0	1	1	1	0
0	0	0	3	3	5	1
13522000	0	0	0	0	0	1
0	0	11879000	1	1	0	1
0	0	0	3	1	2	0
0	0	0	3	5	2	0
82582000	86208000	84022000	5	5	5	4
38721000	40611000	34286000	1	1	1	1
41499000	44896000	50536000	7	6	5	2
46753000	48305000	42442000	2	3	3	2
0	0	0	1	0	1	0
0	0	0	3	4	3	0
10124000	0	9995000	0	0	1	1
0	0	0	0	0	0	0
21628000	17956000	24206000	5	0	1	0
0	0	0	2	1	1	0
84499000	78591000	123250000	5	4	4	2
0	0	0	2	0	1	0
0	0	11289000	1	1	1	0

0	0	0	4	7	6	0
0	0	0	1	1	1	0
0	0	38541000	0	0	0	1
64162000	76523000	77290000	4	6	6	3
0	18255000	14684000	7	8	8	0
41734000	17266000	12707000	0	0	0	2
0	0	0	2	1	1	0
18561000	11835000	18514000	19	19	20	2
0	0	0	3	5	4	0
0	0	0	2	1	2	1
0	0	0	1	0	0	0
0	0	0	5	2	3	0
0	0	13428000	1	1	1	1
0	0	0	3	0	2	0
0	0	0	0	1	0	0
0	0	4415600	0	0	0	0
416390000	546180000	553390000	26	31	28	9
216850000	266730000	281390000	5	3	5	2
0	0	0	0	1	1	0
11113000	9197700	33678000	14	13	14	0
66346000	78684000	82486000	7	5	8	1
0	0	0	0	0	1	0
67644000	76063000	81011000	4	5	6	6
0	0	0	2	5	5	1
0	0	0	1	1	0	0
0	0	0	1	1	1	0
248440000	310420000	277790000	15	18	21	4
0	0	0	4	4	4	0
0	0	8405900	1	0	0	1
0	0	0	1	1	2	0
4101700000	4113700000	5191200000	30	30	40	19
0	0	5903000	0	0	0	1
0	0	0	1	1	2	0
0	0	0	7	8	5	0
0	0	0	3	1	2	0
124410000	161000000	140810000	3	3	3	4
0	0	0	1	0	1	0
0	0	0	0	1	2	1
237120000	275030000	200200000	8	8	7	5
0	0	0	0	1	0	0
0	0	0	1	1	0	0
161330000	209370000	173720000	4	7	6	1
0	0	0	1	1	2	0
168460000	182120000	193310000	2	3	2	4
1939500000	2308600000	2172400000	20	24	24	21

11342000	0	0	0	1	0	1
0	0	0	1	0	1	0
0	0	2037200	1	0	0	1
0	0	0	0	0	1	0
0	0	0	4	4	3	0
1,9923E+10	1,1522E+10	############	68	64	86	58
0	0	0	1	2	1	0
25376000	49058000	54254000	7	8	11	0
53273000	57818000	55336000	4	3	3	4
61806000	54378000	79481000	2	3	1	2
0	0	0	1	1	2	0
169700000	239570000	228230000	7	10	10	8
0	0	14336000	0	0	0	1
0	0	0	1	0	1	0
108370000	113730000	91400000	3	4	3	3
0	0	0	13	12	18	0
299700000	410000000	302160000	6	6	5	4
0	0	0	1	1	1	0
0	0	0	1	1	2	0
0	0	8693100	1	1	1	0
0	0	2008200	1	1	1	0
97197000	113780000	108530000	0	0	0	4
0	0	5182400	0	0	0	0
187830000	223100000	177570000	7	6	9	8
54600000	61000000	45530000	5	8	10	1
0	0	15901000	0	0	0	1
0	0	5666300	1	1	1	0
0	27134000	31775000	4	4	8	2
0	0	0	1	1	1	0
0	0	0	1	1	1	0
20128000	0	0	0	0	0	1
49662000	52116000	50652000	5	6	9	3
2580600000	2968000000	2894800000	39	44	46	29
27043000	33334000	33677000	3	3	2	2
711190000	852550000	1033200000	11	11	14	15
0	0	0	1	1	2	1
0	0	0	0	0	1	0
0	0	0	0	1	1	1
139120000	98033000	125720000	4	4	2	2
213450000	257180000	226580000	14	15	15	7
0	0	0	2	4	1	0
0	0	0	1	1	1	0
0	0	0	4	5	6	0
112380000	90167000	86480000	4	2	5	4
47559000	43005000	54878000	3	3	4	2

0	0	0	1	1	1	0
0	0	8764400	0	0	0	0
0	0	0	3	3	3	0
0	0	0	1	3	1	0
0	0	0	0	0	1	0
0	0	0	1	1	2	0
17283000	24263000	18591000	2	2	2	1
169430000	167740000	184270000	36	36	41	7
0	0	0	1	1	2	0
0	0	6401700	1	1	1	1
0	0	73513000	0	1	0	0
0	0	0	0	1	0	0
37813000	38509000	39446000	6	5	5	0
0	0	0	4	5	5	0
0	0	14888000	26	26	25	0
0	0	0	0	0	1	0
0	0	0	4	7	5	2
0	0	0	0	2	0	0
23429000	33624000	32635000	12	13	12	1
319860000	349900000	353270000	16	14	16	9
0	0	0	7	6	5	0
0	0	0	6	6	5	0
0	0	0	1	1	1	0
0	0	0	6	5	9	1
0	0	0	1	1	1	0
254290000	298550000	280840000	5	6	6	6
0	0	0	1	1	1	0
50090000	0	40879000	4	5	5	1
528590000	334140000	333570000	8	10	16	14
14985000	16403000	12867000	0	0	0	1
0	0	0	2	3	2	2
492170000	462590000	487800000	10	9	11	9
0	0	0	14	14	15	0
40778000	48363000	56974000	3	2	1	2
0	0	0	0	0	1	0
249920000	168390000	195040000	7	8	11	6
112650000	110420000	98957000	12	9	12	2
0	0	0	2	1	2	0
0	0	0	10	7	10	0
0	0	0	2	0	3	0
46309000	55237000	65948000	2	1	1	2
12424000	0	13337000	3	2	2	1
127710000	159600000	156490000	8	6	6	4
0	0	0	3	2	3	0
226430000	210610000	276320000	4	4	5	6

83802000	64126000	119300000	1	1	1	4
109280000	112840000	106240000	10	10	13	3
95399000	55087000	75916000	3	0	2	3
67337000	68686000	82580000	9	8	9	2
0	0	0	14	10	13	0
93126000	100940000	107250000	1	2	3	4
0	0	12729000	0	0	0	0
0	0	0	1	1	1	0
0	0	0	0	1	2	0
30156000	27101000	20334000	9	9	7	0
0	0	0	10	8	9	0
234110000	0	0	0	0	0	4
118340000	127420000	129720000	12	12	14	3
0	0	20777000	0	1	0	0
84061000	96723000	101980000	10	7	9	2
102320000	110380000	103070000	4	5	7	1
0	0	128550000	0	0	0	1
0	0	0	0	1	1	0
0	0	10687000	0	0	0	0
0	0	0	3	2	2	0
0	0	0	1	1	1	0
0	0	0	2	3	1	0
99409000	97882000	133520000	6	8	7	3
33698000	52148000	48914000	5	6	7	1
0	0	0	1	0	1	0
4598200000	4776500000	4261300000	55	51	60	46
120220000	75653000	111650000	1	3	1	2
0	0	0	1	0	1	0
123000000	42436000	59352000	2	1	6	6
28792000	49113000	45965000	5	3	6	1
0	0	0	12	7	12	0
0	0	0	6	7	7	0
0	0	6290600	0	0	0	1
655660000	1046800000	909600000	14	13	11	7
59712000	27630000	23875000	6	5	5	2
4236900000	3139000000	2880900000	18	20	22	18
171960000	190650000	213330000	16	16	15	5
0	0	0	5	3	2	0
0	0	0	1	0	1	0
0	0	0	4	4	3	1
66684000	18822000	24610000	24	24	23	2
520880000	554700000	534770000	9	8	10	9
1570100000	1786200000	2067200000	4	6	6	13
0	0	0	2	1	1	0
0	0	5779500	0	0	0	1

0	0	0	1	0	0	0
167580000	137480000	132080000	74	75	78	9
548170000	552810000	600300000	64	62	60	14
436560000	481180000	509600000	34	34	37	10
0	0	0	1	2	1	0
52252000	77593000	103520000	4	1	2	1
0	0	4747700	1	1	0	2
36316000	26706000	24455000	3	3	5	2
126710000	36643000	46461000	7	8	7	4
2929600000	2904400000	3018900000	18	21	26	27
1351000000	821320000	667320000	33	33	30	43
569920000	1291600000	740760000	9	11	9	6
1568400000	4278400000	1903300000	11	12	11	11
0	0	0	4	2	4	0
0	0	0	1	1	2	0
0	0	0	0	0	2	2
0	0	0	1	0	0	0
91063000	94638000	102160000	10	9	10	2
0	0	0	8	6	7	0
0	0	0	4	4	4	2
21002000	0	0	27	24	30	1
0	0	0	2	4	4	0
336760000	363540000	331730000	26	29	30	7
194480000	139780000	123270000	12	12	14	7
0	0	3807100	0	0	0	0
268980000	258530000	269430000	13	12	16	5
0	0	0	3	3	4	0
117970000	117910000	99430000	0	1	2	4
0	0	0	2	1	4	0
52441000	12038000	8141800	0	0	0	4
69810000	67958000	81437000	7	9	9	3
203270000	169560000	213570000	6	5	6	2
80278000	68638000	75364000	6	6	7	3
102350000	82030000	54235000	8	5	5	5
591110000	715480000	601920000	13	11	11	10
0	0	7402900	0	0	0	1
1395000000	1398400000	1806100000	15	11	20	16
451710000	495720000	532940000	14	14	18	10
0	0	0	4	3	4	0
0	0	0	1	1	3	3
0	1156300	0	0	0	0	1
1281700000	1395900000	1247300000	11	8	9	13
149230000	183590000	150460000	7	6	5	2
8245200	18377000	7489200	0	0	0	0
0	0	0	2	3	0	0

0	0	0	24	22	19	0
119320000	0	43610000	0	1	1	3
84488000	83456000	83299000	0	0	0	3
0	0	0	5	5	9	0
0	0	0	16	17	17	0
51720000	52543000	67621000	6	5	8	2
69432000	71516000	72750000	10	10	10	3
761010000	876970000	1005000000	6	9	7	8
1,1284E+10	9452000000	6583300000	57	39	59	54
0	0	0	4	2	3	0
342940000	350980000	385440000	16	13	16	6
227670000	234690000	232980000	5	4	5	5
543870000	398780000	319030000	4	7	14	7
199910000	163600000	193340000	6	6	5	8
211480000	190050000	223150000	10	8	13	5
12721000	10826000	12570000	3	1	2	1
189570000	208100000	228240000	7	7	5	2
0	0	0	2	2	2	0
0	0	0	1	1	1	0
1555500000	1792500000	1676900000	38	34	42	24
0	0	0	6	4	8	0
0	0	0	3	0	2	0
0	0	0	10	8	10	0
169900000	174170000	198870000	9	9	11	4
334380000	345360000	391890000	8	9	8	10
72771000	93604000	76526000	3	4	3	2
55780000	36594000	43011000	3	3	3	2
0	0	0	1	2	0	0
0	0	0	1	2	1	0
0	0	0	0	0	1	0
0	0	0	1	0	2	0
0	0	0	6	5	5	0
0	0	0	2	0	1	0
116830000	126050000	109500000	3	4	4	4
39497000	63863000	41187000	1	1	1	1
0	0	8502800	0	0	1	0
0	0	40540000	7	7	8	0
155110000	150470000	176970000	11	10	12	4
317540000	377960000	357050000	18	18	18	15
0	36217000	37206000	12	13	11	1
0	0	0	1	0	0	0
0	0	0	2	2	3	0
0	0	0	24	19	20	0
29743000	25857000	38951000	3	2	4	3
934650000	983550000	1003800000	9	12	13	17

668360000	896530000	965960000	44	53	45	8
21183000	14982000	26357000	1	1	1	2
0	0	0	15	15	16	0
0	0	0	5	6	7	0
0	0	0	0	1	1	0
88097000	60652000	76716000	2	2	2	3
0	0	0	0	0	1	0
0	0	10182000	0	1	1	1
0	0	0	8	4	7	0
37778000	49780000	44181000	2	2	1	2
1127600000	1215700000	1295600000	33	28	33	29
167710000	179990000	232290000	5	2	5	3
93977000	77939000	80483000	4	4	5	2
0	0	0	0	1	1	0
57764000	21893000	0	1	1	1	2
44125000	0	0	0	0	0	1
0	0	0	1	0	0	0
0	11578000	0	1	1	0	1
83863000	90112000	75007000	3	4	4	1
85858000	85848000	66552000	7	9	6	3
33183000	58112000	36834000	10	11	10	3
0	0	0	3	2	2	1
152920000	138050000	165570000	8	8	9	8
70007000	80122000	56817000	7	5	8	1
0	132230000	115540000	6	8	7	1
0	0	9728100	1	1	1	0
192750000	237800000	209750000	8	8	11	6
43411000	0	0	0	0	0	2
47165000	0	50398000	1	2	2	2
108510000	89005000	98300000	2	2	3	2
122680000	77909000	105410000	0	1	2	3
303280000	373290000	385770000	13	13	14	4
488630000	488820000	444510000	6	7	6	6
73980000	114050000	84762000	1	5	6	2
0	0	0	1	2	3	0
0	0	0	1	1	0	0
0	0	13353000	1	1	1	0
143620000	166550000	182800000	8	9	9	5
149510000	159740000	155290000	8	8	8	3
186390000	210200000	170240000	10	9	10	2
0	0	30203000	1	1	1	1
124690000	134110000	139090000	3	4	6	3
0	0	0	1	2	2	0
122120000	118130000	128380000	7	5	5	4
37152000	0	0	1	1	1	2

###################################			20	21	26	18
0	0	0	2	6	4	0
###########	2641000000	2748300000	26	27	30	17
258300000	261890000	323120000	44	46	42	10
547140000	636830000	751220000	58	48	57	13
0	0	0	2	0	1	0
34024000	26362000	43338000	7	6	5	2
0	0	0	1	3	4	0
0	0	0	1	2	2	0
2483000000	2849100000	2867700000	128	124	133	45
0	0	6641800	0	0	0	1
249720000	147390000	92749000	2	3	2	2
71708000	34019000	44187000	0	0	0	4
1256900000	1193100000	1092100000	21	18	27	19
840950000	576040000	1040700000	9	6	9	10
0	0	0	2	4	5	0
35819000	37738000	35933000	6	6	3	1
24251000	0	0	2	1	1	2
104760000	96024000	102750000	5	5	5	5
75678000	43050000	20420000	3	4	5	4
291420000	312360000	334490000	16	18	22	7
19932000	18059000	24451000	18	21	20	0
27059000	34844000	33014000	1	1	1	1
0	0	0	2	3	4	0
46596000	50686000	54623000	6	6	9	1
0	34046000	39940000	2	2	2	1
19305000	25011000	57015000	30	33	35	1
1419100000	1676600000	1693300000	11	12	11	13
1711800000	0	0	0	0	0	2
177070000	148100000	106060000	3	5	6	7
0	24052000	11044000	1	1	1	1
250150000	263730000	266350000	9	12	10	6
0	0	0	8	7	7	1
73299000	66611000	78504000	9	8	8	2
0	0	0	9	14	14	0
0	0	20485000	1	1	1	0
21112000	22662000	24903000	0	3	2	0
0	0	0	4	3	4	0
129830000	81721000	70289000	3	3	7	6
0	0	0	14	16	12	0
263610000	300350000	251050000	4	6	8	9
443560000	484460000	400780000	10	11	14	16
0	0	0	5	5	6	0
0	0	0	5	3	4	0
85686000	102390000	88033000	5	6	9	2

46985000	54608000	77947000	4	4	5	3
42290000	58522000	44285000	0	0	0	0
0	0	0	5	3	3	0
0	16825000	0	10	10	11	1
0	0	0	1	2	2	0
221490000	234420000	221590000	4	4	7	7
0	0	0	4	5	5	0
41185000	39067000	34684000	0	0	1	1
27804000	55418000	50620000	3	3	2	2
0	0	0	0	2	1	0
914340000	959260000	1133800000	29	22	27	9
54348000	0	0	0	0	0	1
183760000	224630000	210780000	13	14	16	8
0	0	0	0	0	1	0
146900000	170260000	170580000	2	4	5	10
0	0	0	2	1	1	0
32082000	42492000	44850000	19	22	19	2
1109300000	1178400000	1131000000	14	14	20	16
50021000	49118000	45706000	2	2	7	4
0	0	3849600	0	1	1	0
0	0	0	149	154	151	0
256460000	298690000	323490000	11	9	14	7
0	0	0	2	3	4	0
62202000	50762000	96067000	14	15	17	1
0	0	0	1	2	1	0
9163400	0	12519000	2	2	2	1
0	0	0	1	0	1	0
0	0	12727000	1	1	1	1
0	0	0	5	6	5	0
0	0	0	11	9	5	1
1372800000	1270700000	1583600000	18	16	16	12
0	0	0	1	0	0	0
1088100000	1193800000	1180200000	15	14	17	21
82594000	123320000	132530000	6	8	7	2
63298000	66604000	64628000	4	3	4	3
26133000	21472000	34136000	4	6	4	2
0	0	0	3	4	3	0
83420000	94161000	95091000	3	3	3	3
0	0	0	41	35	41	0
84257000	84477000	89363000	1	3	3	2
1,7084E+10	0	0	0	0	0	7
0	0	0	4	2	3	0
18475000	23393000	20914000	1	0	1	1
69371000	0	0	0	0	0	5
0	0	0	1	1	1	0

11353000	12451000	17240000	2	1	2	1
0	0	0	0	0	1	0
0	0	0	3	4	4	1
0	0	0	1	2	1	0
23962000	29671000	29280000	6	3	4	0
55079000	61401000	69673000	6	5	7	3
0	0	45377000	1	1	1	1
0	0	0	0	1	1	0
7213800000	4448100000	1,0319E+10	9	9	10	10
168740000	225340000	263360000	13	10	12	4
0	0	0	4	2	7	0
0	0	2897900	0	0	0	0
0	0	0	1	1	0	0
0	0	0	5	3	4	0
34872000	50565000	49102000	3	2	0	2
0	0	0	2	2	1	0
0	0	0	2	3	3	0
75745000	113650000	65520000	0	0	0	2
0	0	0	9	9	9	0
0	60978000	41243000	10	10	11	1
168970000	240620000	209740000	6	2	5	5
38978000	58431000	55506000	11	8	13	1
0	0	0	2	2	2	0
0	0	11588000	0	0	0	0
0	0	0	1	0	3	0
0	0	0	0	1	2	0
85504000	137940000	63722000	6	6	12	4
25931000	21929000	11126000	0	0	2	2
0	0	4505900	0	0	0	0
11129000	10021000	14888000	0	0	0	0
373330000	296290000	283430000	3	3	3	3
0	0	0	5	5	4	0
0	0	0	5	4	4	0
340380000	440120000	419130000	41	37	47	8
121430000	153710000	166080000	3	3	2	3
0	0	0	6	4	6	0
0	0	0	1	1	1	0
0	0	0	8	8	10	0
0	0	0	10	9	8	0
52065000	47555000	69671000	1	2	2	1
0	0	0	3	6	2	0
0	0	0	2	3	5	0
0	0	0	4	0	2	0
912930000	1058400000	994590000	39	38	43	27
0	0	0	5	7	8	0

0	0	0	24	18	20	0
0	0	0	2	3	3	0
0	76124000	98413000	3	2	5	1
0	0	7668500	0	0	0	1
0	0	0	4	4	4	0
0	0	0	6	7	8	0
0	0	0	2	4	4	0
124830000	126610000	105330000	11	9	10	5
0	0	0	4	5	5	0
0	0	0	1	1	1	0
0	0	0	3	2	2	0
0	0	0	1	0	1	0
0	0	0	5	5	7	1
47885000	46670000	26420000	4	3	4	3
0	0	0	0	0	1	0
0	485090000	323400000	0	0	0	0
0	0	0	1	2	2	0
0	0	0	2	2	1	0
28618000	32910000	39700000	0	0	0	1
0	0	0	1	0	0	0
0	0	0	12	11	11	0
0	0	0	8	6	10	0
0	0	0	0	0	2	1
0	0	0	13	12	14	0
0	0	0	2	2	5	1
21175000	24556000	20926000	1	0	0	0
0	0	0	1	1	0	0
0	0	0	0	0	1	0
45497000	0	0	0	0	0	4
0	0	0	0	0	1	0
0	0	0	1	1	2	0
32850000	29442000	32694000	2	2	3	1
31146000	22151000	34208000	10	13	11	2
195330000	245340000	215450000	3	3	4	4
199340000	125110000	98179000	1	5	9	6
104270000	122740000	123180000	4	3	3	5
0	0	0	3	4	3	0
11654000	16668000	15009000	0	0	0	1
80835000	96277000	74569000	11	8	9	2
0	0	0	1	1	1	0
0	0	0	1	1	2	0
2809200000	3389800000	3246200000	27	26	33	29
0	0	0	1	1	1	0
27555000	26342000	29840000	4	2	5	3
0	0	0	1	0	0	0

0	0	0	2	3	1	0
0	0	0	7	10	6	0
28637000	0	0	0	0	0	1
0	0	0	5	6	4	1
30435000	40255000	39464000	4	4	5	1
0	0	0	5	4	4	0
0	0	0	1	1	0	0
0	0	0	2	2	2	0
0	0	0	8	9	9	1
0	0	0	2	2	0	0
0	0	0	2	1	0	1
28432000	26675000	26714000	2	2	2	1
32870000	21085000	25558000	0	1	1	0
0	0	0	1	1	1	0
0	0	0	4	2	3	0
6082500	5884300	10320000	0	1	0	0
122370000	144550000	109480000	3	4	3	3
0	0	0	2	1	2	0
136930000	165970000	143550000	2	5	7	3
0	0	0	37	36	40	0
118100000	195620000	146970000	4	2	2	4
0	0	0	0	1	1	0
0	0	4260800	0	0	0	0
0	0	0	18	19	25	0
0	0	0	11	11	11	0
28906000	39758000	40509000	1	1	3	1
164890000	220600000	270030000	4	5	8	3
0	0	0	3	4	3	2
0	0	0	1	0	1	0
0	0	0	2	1	1	0
0	0	0	3	5	5	0
0	0	0	3	4	5	0
427220000	478210000	459410000	17	16	14	17
0	0	36474000	4	5	4	1
99479000	126030000	114510000	84	82	90	5
12000000	13839000	13562000	1	0	0	1
89415000	210630000	76008000	1	4	4	5
198770000	233130000	252710000	15	12	19	9
56688000	61226000	64441000	10	8	9	1
35935000	53808000	48941000	2	1	2	3
3837900000	4431500000	4077600000	32	36	38	42
0	0	0	1	0	3	0
0	0	9641500	1	1	1	0
0	0	0	1	1	1	0
0	0	5541400	0	1	1	1

0	0	0	2	2	1	0
0	0	0	1	2	1	0
0	0	0	1	3	1	0
184600000	215810000	190050000	7	6	7	6
56663000	45574000	41403000	0	0	0	3
34877000	41462000	30161000	5	2	4	0
370290000	424040000	393930000	9	9	12	12
81587000	151190000	0	0	0	1	1
0	0	0	6	5	3	0
0	0	0	8	4	7	0
0	0	0	1	1	1	0
0	0	9058000	1	1	1	1
26060000	31921000	33404000	4	4	5	1
0	0	0	2	1	1	0
0	0	0	2	2	2	1
0	0	0	7	8	12	0
0	0	0	2	2	2	0
512040000	654770000	716700000	23	25	24	7
5262900	6069700	8068700	0	0	0	0
0	0	0	0	1	2	0
0	0	0	14	14	16	0
0	0	0	2	1	2	0
0	0	0	2	3	5	0
0	0	0	1	4	7	0
0	0	0	10	7	8	0
2235900000	3182000000	77405000	48	45	50	2
0	0	0	3	4	3	0
0	0	0	0	1	1	0
165460000	204410000	157230000	2	4	3	3
20048000	29688000	23934000	1	1	2	0
625970000	836020000	674550000	32	30	28	12
81766000	123350000	144710000	9	4	3	1
413720000	650150000	691350000	8	9	11	9
315790000	329970000	390070000	3	3	5	7
89689000	141280000	132670000	17	17	18	2
0	0	14549000	0	0	1	1
271860000	314490000	296480000	8	8	7	9
0	0	0	6	6	6	0
50917000	58860000	65023000	8	5	9	0
0	0	0	6	7	6	0
0	0	0	5	3	3	0
86294000	99664000	77343000	6	3	3	3
0	0	0	2	1	3	0
0	0	0	2	3	3	0
0	0	17418000	0	0	0	0

0	0	0	14	12	12	0
39117000	40972000	53825000	2	2	3	5
0	0	0	1	1	1	0
0	0	0	2	2	2	0
0	0	0	0	1	1	1
0	0	0	19	20	22	0
0	0	0	7	4	7	0
0	0	0	2	0	1	0
49740000	47954000	46860000	1	1	1	2
50710000	0	57575000	3	4	7	1
0	0	27287000	0	0	0	0
0	0	0	5	6	5	0
0	0	0	1	1	2	0
135030000	153040000	138230000	5	5	6	3
10965000	11413000	30560000	9	11	10	1
0	0	0	2	2	1	0
0	0	0	2	0	1	0
0	0	0	45	46	50	0
0	0	0	1	1	1	1
33802000	0	30729000	1	1	2	1
744460000	994540000	1078000000	7	5	6	22
69779000	98457000	87666000	2	2	3	1
0	0	0	0	1	1	0
0	0	0	1	1	1	0
0	0	0	9	10	7	0
309890000	404470000	409900000	43	38	38	1
129200000	129920000	128420000	5	4	6	4
13127000	15411000	15394000	1	1	1	1
120350000	159500000	136880000	27	24	26	2
0	0	0	1	0	1	0
0	0	0	4	3	5	0
0	0	0	1	0	0	0
17699000	0	17784000	1	2	1	1
50026000	56811000	54862000	2	2	4	1
0	0	0	0	1	0	0
40056000	0	41627000	1	2	2	2
0	0	0	1	0	1	0
2192200000	2003600000	1730400000	28	34	36	35
0	0	0	13	7	12	0
0	0	0	1	1	1	0
0	0	0	1	1	1	0
0	0	0	17	19	23	0
122250000	169810000	161910000	2	1	2	4
0	0	0	0	1	2	1
26668000	26984000	41701000	0	0	1	2

0	0	0	1	1	3	0
78379000	83054000	63125000	3	4	4	2
0	0	4444300	1	0	1	0
41726000	56756000	47939000	0	0	0	2
0	0	0	1	1	2	0
0	0	0	1	1	1	0
0	0	0	4	3	4	0
0	0	0	3	4	6	0
516720000	558230000	557550000	21	15	19	8
0	0	0	0	2	1	0
0	0	0	2	2	2	0
45440000	96474000	35026000	1	1	1	1
0	0	0	12	10	13	0
38450000	34458000	45063000	3	4	3	3
0	0	0	3	4	1	0
0	0	0	2	1	1	0
88018000	109540000	108220000	4	4	4	1
0	0	0	1	2	2	0
60286000	70681000	79204000	3	5	4	5
6053800	0	0	0	0	0	1
0	0	0	1	2	1	0
0	0	11327000	0	0	0	1
54472000	35352000	64599000	7	10	9	2
0	0	0	15	12	18	0
0	0	0	3	6	3	0
26151000	25030000	32702000	5	5	5	3
0	0	0	4	5	6	0
0	0	0	10	10	12	0
282860000	205770000	257620000	15	13	15	9
975740000	1199200000	1014900000	19	25	31	24
196080000	199660000	233490000	22	18	16	5
0	0	0	0	1	1	0
0	0	0	6	5	5	0
0	0	0	1	2	3	0
386520000	381890000	548130000	12	9	12	9
126650000	147860000	197150000	9	7	11	5
714630000	847730000	908490000	18	18	23	14
214810000	239870000	256360000	21	22	19	5
0	0	0	28	26	27	0
902610000	1029000000	956110000	15	13	15	12
0	0	0	1	1	1	0
308550000	287010000	362290000	2	3	2	7
0	0	0	1	1	1	1
0	0	0	2	2	1	0
40156000	40870000	41571000	3	3	4	2

32551000	39617000	39112000	1	1	2	1
111730000	106370000	116750000	6	5	3	9
0	0	0	17	13	22	0
0	0	0	1	1	1	0
66545000	74680000	85178000	16	16	17	4
222740000	244650000	300300000	12	16	18	4
37808000	58049000	0	4	5	4	2
21422000	28707000	24050000	3	6	5	1
11936000	0	0	0	0	1	1
0	0	159860000	0	1	1	1
0	0	43332000	0	0	0	0
0	0	0	0	1	0	0
0	0	34844000	0	0	0	0
0	0	11192000	1	1	0	1
0	0	0	0	0	1	1
0	0	210620000	0	0	0	0
0	0	0	0	0	1	0
0	408720000	0	1	1	1	1
0	255520000	0	0	0	0	0
0	0	0	1	1	1	0
0	0	0	1	0	0	0
0	4753200000	0	0	0	0	1
0	0	0	1	0	0	0
0	0	4944900000	1	1	1	0
0	0	388950000	1	1	1	1
0	0	47414000	1	1	1	1
0	0	0	1	1	1	0
190150000	204660000	216450000	6	7	9	7
114020000	108180000	105920000	5	4	4	4

MS/MS Coun	MS/MS Coun	MS/MS Coun	Only identifie	Reverse	Potential con	id
1	2	13				0
1	1	8				1
0	0	5				2
5	10	31				3
1	4	21				4
0	1	5				5
0	0	2				6
4	5	33				7
5	5	25				8
0	0	3				9
2	2	10				10
1	0	4				11
2	2	16				12
0	0	2				13
0	0	4				14
0	0	1				15
3	7	30				16
0	0	1				17
3	6	64				18
1	2	16				19
4	8	37				20
0	1	2				21
17	21	107				22
0	0	4				23
0	0	26				24
1	1	5				25
0	0	15				26
0	0	0				27
0	2	12				28
0	0	18				29
0	0	32				30
0	0	1				31
0	0	2				32
0	0	4				33
1	3	11				34
0	0	1				35
2	5	22				36
0	0	3				37
0	0	1				38
0	3	16				39
0	0	3				40
0	0	33				41
0	2	3				42
3	5	13				43

2	3	24			44
1	0	5			45
0	2	4			46
0	0	3			47
0	0	15			48
0	0	4			49
0	0	0	+		50
0	1	5			51
2	3	20			52
0	0	8			53
0	0	15			54
0	1	6			55
4	4	35			56
0	0	0			57
0	0	13		+	58
0	1	1			59
2	5	12			60
2	2	16			61
0	0	1			62
3	5	42			63
0	0	64			64
0	2	13			65
0	2	8			66
0	0	5			67
0	0	11			68
26	32	165			69
0	0	5			70
3	5	24			71
4	3	29			72
0	0	2			73
0	0	1			74
0	0	3			75
0	0	26			76
3	4	24			77
0	1	9			78
0	0	1			79
1	3	18			80
0	4	12			81
0	0	4			82
3	5	32			83
0	0	6			84
0	1	14			85
0	0	10			86
0	0	4			87
0	1	6			88

0	2	2		89
1	5	12		90
0	2	6		91
1	2	5		92
0	0	15		93
1	1	6		94
10	18	76		95
0	0	1		96
3	3	16		97
0	0	9		98
0	0	1		99
0	2	29		100
1	1	6		101
3	3	14		102
2	4	19		103
0	0	63		104
0	0	2		105
8	20	62		106
3	4	25		107
0	0	2		108
0	0	8		109
0	2	16		110
3	4	27		111
0	0	3		112
0	0	8		113
0	0	4		114
1	2	9		115
3	6	37		116
8	8	55		117
9	16	75		118
0	0	5		119
1	1	6		120
0	0	2		121
0	0	2		122
0	0	3		123
0	0	2		124
0	0	1		125
1	1	5		126
62	79	645		127
1	1	2		128
0	0	9		129
1	1	5		130
0	0	1	+	131
10	15	100		132
0	0	2		133

0	1	4		134
1	2	38		135
4	6	32		136
2	3	9		137
0	0	4		138
0	0	3		139
0	0	4		140
0	0	29		141
0	0	3	+	142
1	2	7		143
0	0	16		144
0	0	7		145
0	0	6		146
0	0	7		147
1	3	9		148
0	0	3		149
9	13	71		150
0	0	5		151
8	16	88		152
2	5	75		153
0	0	6		154
0	1	3		155
1	3	15		156
7	17	88		157
0	0	1		158
0	0	8		159
0	0	38		160
0	1	2		161
1	3	24		162
0	1	4		163
0	0	3		164
0	0	5		165
1	1	12		166
0	0	10		167
0	0	3		168
0	1	3		169
0	0	2		170
0	2	18		171
0	0	18		172
0	2	10		173
1	1	8		174
0	0	3		175
3	4	29		176
0	0	3		177
6	12	49		178

8	12	55	179
7	11	41	180
5	8	36	181
0	0	5	182
0	0	3	183
0	0	11	184
2	4	37	185
14	20	103	186
1	1	12	187
0	0	12	188
0	0	13	189
0	0	2	190
0	0	14	191
0	0	2	192
1	1	30	193
0	0	2	194
0	2	23	195
0	0	4	196
0	0	3	197
4	6	68	198
0	0	22	199
13	16	94	200
0	0	3	201
23	35	176	202
0	0	4	203
5	8	41	204
0	2	3	205
2	5	25	206
5	10	35	207
2	5	28	208
15	14	86	209
3	4	21	210
1	0	9	211
7	8	68	212
0	1	1	213
1	1	5	214
0	1	5	215
12	12	93	216
1	3	10	217
3	6	27	218
1	4	28	219
1	1	3	220
1	5	18	221
0	1	4	222
6	6	44	223

4	7	33	224
1	0	3	225
0	1	2	226
0	1	14	227
0	0	20	228
0	0	15	229
3	1	9	230
1	1	4	231
0	0	5	232
2	3	7	233
0	1	15	234
0	0	1	235
1	2	4	236
0	0	7	237
0	0	4	238
0	0	17	239
1	2	10	240
2	4	24	241
0	0	3	242
0	2	117	243
0	0	9	244
1	1	20	245
0	1	3	246
0	0	3	247
0	0	2	248
0	0	1	249
1	1	5	250
0	0	3	251
0	0	15	252
0	0	2	253
0	0	1	254
0	0	6	255
2	5	10	256
2	9	56	257
0	1	5	258
0	1	3	259
1	0	4	260
0	0	4	261
0	0	7	262
0	0	2	263
1	2	30	264
0	4	5	265
0	0	30	266
0	0	2	267
4	3	17	268

0	0	5			269

Q6L675;Q6L625;Q6I6G7;Q6I688;Q6F3I9;Q6F3H0;Q6F3E3;Q6F3E2;Q6AW25;Q68Y63;Q60I39;Q5W9R8;Q5R1M1;Q5R1M0;Q
R130;F8R129;F8R128;F8R126;F8R125;F8R124;F8R123;F8R122;F8R121;F8R120;F8R119;F8R118;F8R117;F8R116;F8R115;F8R
L6P9;Q6L676;Q6L675;Q6L625;Q6I6G7;Q6I688;Q6F3I9;Q6F3H0;Q6F3E3;Q6F3E2;Q6AW25;Q68Y63;Q60I39;Q5W9R8;Q5R1M
R130;F8R129;F8R128;F8R126;F8R125;F8R124;F8R123;F8R122;F8R121;F8R120;F8R119;F8R118;F8R117;F8R116;F8R115;F8R
3;273;273;273;273;273;273;273;273;273;273;273;273;273;273;273;273;273;273;273;273;273;273;273;273;273;273;273;273;27

3	3	18			271
0	0	23			272
0	0	0			273
0	0	2			274
0	0	2			275
0	0	2			276
1	2	8			277
0	1	10			278
0	0	1			279
0	1	3	+		280
5	14	68			281
0	0	1			282
0	0	3			283
0	0	4			284
0	1	8			285
0	0	2			286
0	0	11			287
0	0	4			288
1	1	7			289
126	171	931			290
2	5	20			291
0	0	3			292
4	5	19			293
0	0	9			294
0	0	5			295
0	0	3			296
0	0	2			297
0	0	2			298
0	0	2			299
2	2	9		+	300
0	0	10			301
4	11	21			302
0	3	5			303
0	0	1			304
0	0	3			305
0	0	2			306
1	1	6			307
0	1	4			308

2	4	18		309
0	0	1		310
0	0	2		311
0	1	1		312
0	0	13		313
1	1	4		314
0	0	1	+	315
0	0	1		316
0	0	2		317
2	2	18		318
2	2	13		319
2	6	43		320
1	0	1		321
2	5	20		322
1	1	9		323
0	0	1		324
0	0	2		325
0	1	3		326
3	2	14		327
0	0	6	+	328
1	1	10		329
4	5	31		330
0	1	4		331
0	1	8		332
0	0	14		333
0	0	3		334
0	0	3		335
13	14	80		336
0	0	10		337
0	0	7		338
1	1	3		339
0	0	1		340
0	0	6		341
0	2	7		342
0	1	2		343
0	0	2		344
4	6	34		345
0	0	171		346
0	1	1		347
1	2	6		348
0	0	7		349
0	0	2		350
0	0	2		351
1	2	8		352
0	0	1		353

0	0	8		354
1	3	10		355
0	0	3		356
5	9	46		357
0	0	3		358
1	0	2		359
0	0	3		360
0	0	1		361
1	3	28		362
1	1	6		363
4	6	33		364
11	17	65		365
4	8	26		366
7	11	92		367
1	3	10		368
0	0	9		369
2	5	16		370
0	0	2		371
20	35	165		372
0	0	14		373
0	6	18		374
0	0	3		375
0	0	4		376
0	3	13		377
7	7	99		378
0	0	2		379
0	0	4	+	380
1	1	25		381
0	0	3		382
0	1	27		383
0	1	2		384
0	1	3		385
2	5	22		386
0	0	2		387
1	2	12		388
0	1	5		389
1	2	8		390
2	1	5		391
0	0	4		392
0	0	2		393
2	3	19		394
0	0	3		395
0	0	1		396
6	12	45		397
3	2	22		398

0	0	20		399
0	0	11		400
0	2	12		401
0	1	1		402
1	3	35		403
0	1	3		404
4	8	42		405
2	2	12		406
0	0	1		407
5	7	32		408
0	0	8		409
0	0	3		410
0	0	6		411
0	0	3		412
0	1	11		413
0	0	2		414
1	1	4		415
1	1	6		416
0	0	6		417
1	1	2		418
0	0	3		419
7	12	77		420
0	0	6		421
0	0	33		422
1	5	12		423
4	5	15		424
0	0	2		425
0	0	2		426
7	11	68		427
0	0	2		428
0	0	1		429
0	0	10		430
0	0	2		431
1	3	4		432
0	1	11		433
0	0	11		434
0	0	4		435
0	0	6		436
1	1	12		437
2	5	18		438
0	0	1		439
0	0	3		440
0	0	7	+	441
0	0	11		442
1	1	6		443

1	3	23	444
2	3	13	445
0	0	7	446
1	1	6	447
0	0	2	448
7	9	51	449
0	0	4	450
0	0	8	451
1	6	8	452
1	1	5	453
0	0	3	454
0	0	11	455
15	20	113	456
0	0	6	457
0	0	4	458
0	0	1	459
7	8	52	460
1	1	6	461
0	0	2	462
4	5	38	463
2	4	25	464
0	0	4	465
0	5	12	466
1	2	9	467
0	0	3	468
4	5	34	469
0	2	2	470
1	3	15	471
10	11	60	472
5	5	32	473
3	4	65	474
0	1	5	475
5	7	39	476
0	0	2	477
0	0	5	478
0	0	2	479
1	1	6	480
0	1	14	481
1	3	24	482
0	1	13	483
0	0	2	484
0	1	12	485
1	0	5	486
10	13	48	487
0	0	3	488

1	2	8		+	489
0	1	2			490
0	0	10			491
0	1	7			492
2	2	13			493
0	0	6		+	494
0	0	5			495
8	9	34			496
0	0	1			497
0	0	6			498
1	1	5			499
4	12	42			500
8	11	50			501
8	11	97			502
0	2	3			503
1	1	23			504
0	1	4			505
0	0	1	+		506
0	0	3			507
0	0	6			508
0	0	10			509
2	8	37			510
0	0	2			511
0	1	3			512
0	2	5			513
25	40	288			514
1	1	5			515
0	0	14			516
0	4	34			517
1	1	5			518
0	0	5			519
0	1	19			520
0	0	1			521
0	3	11			522
2	4	18			523
0	0	1			524
0	0	4			525
3	8	25			526
0	1	5			527
1	2	6			528
0	1	1			529
5	7	49			530
1	1	15			531
0	0	1			532
6	7	45			533

0	1	9		534
1	0	6		535
6	13	66		536
0	2	12		537
13	12	89		538
0	0	3		539
1	0	8		540
0	0	17		541
4	8	24		542
0	0	1		543
5	10	46		544
14	23	91		545
0	0	3		546
1	1	8		547
1	0	7		548
1	1	3		549
1	1	6		550
2	3	33		551
0	0	2		552
0	0	1		553
0	1	18		554
0	0	0		555
0	0	0		556
0	0	1		557
1	2	28		558
0	0	1	+	559
0	3	15		560
1	2	10		561
2	6	29		562
0	0	13		563
0	0	11		564
0	1	2		565
0	0	0	+	566
0	0	12		567
1	2	20		568
0	1	2		569
6	14	74		570
2	3	9		571
2	2	12		572
0	0	4		573
5	11	52		574
0	1	14		575
2	5	90		576
0	0	6		577
13	16	84		578

0	2	12	579
0	0	37	580
0	0	30	581
0	0	78	582
0	0	9	583
0	1	4	584
0	2	15	585
0	0	16	586
1	1	21	587
5	4	30	588
2	6	13	589
7	15	82	590
0	0	46	591
0	0	1	592
2	3	13	593
0	0	1	594
0	1	11	595
2	1	10	596
0	0	2	597
0	0	24	598
0	0	1	599
0	0	1	600
9	15	56	601
2	2	26	602
0	0	10	603
2	6	17	604
0	0	5	605
0	0	1	606
0	0	2	607
1	2	3	608
2	5	27	609
0	0	10	610
0	0	1	611
0	0	5	612
0	0	6	613
0	0	14	614
2	2	10	615
0	0	3	616
1	1	6	617
0	1	4	618
0	0	2	619
3	2	16	620
0	0	4	621
0	2	49	622
0	0	8	623

3	4	42	624
0	1	1	625
2	2	29	626
1	1	5	627
1	0	2	628
0	2	9	629
0	0	4	630
0	2	4	631
0	0	4	632
0	1	3	633
0	0	29	634
1	1	3	635
0	2	11	636
0	0	1	637
0	0	6	638
3	8	28	639
0	0	2	640
1	1	6	641
0	1	1	642
0	2	4	643
0	0	4	644
1	2	4	645
1	3	7	646
0	0	3	647
4	6	25	648
0	0	2	649
1	3	16	650
0	1	1	651
2	3	31	652
0	0	5	653
0	3	4	654
3	7	12	655
0	0	5	656
0	0	4	657
0	2	20	658
5	14	34	659
0	0	8	660
0	0	4	661
0	0	6	662
0	0	0	663
0	0	5	664
0	0	3	665
1	1	23	666
0	0	2	667
0	0	1	668

0	0	6	669
0	1	4	670
0	0	16	671
0	0	1	672
0	0	1	673
1	3	11	674
2	1	7	675
2	2	12	676
0	2	54	677
0	0	9	678
2	4	11	679
0	0	8	680
0	1	7	681
0	0	2	682
0	0	1	683
0	2	4	684
0	0	1	685
2	7	37	686
2	3	21	687
0	0	1	688
0	0	7	689
1	1	4	690
0	0	2	691
0	0	39	692
2	7	60	693
0	1	12	694
1	2	4	695
2	3	27	696
2	6	13	697
7	6	28	698
4	11	56	699
1	1	3	700
1	1	6	701
0	0	3	702
0	0	5	703
0	2	3	704
0	0	1	705
0	0	4	706
1	2	11	707
5	6	64	708
0	1	5	709
0	0	2	710
0	0	2	711
0	1	1	712
0	1	2	713

0	1	7		714
0	0	4		715
0	1	1		716
3	2	19		717
5	7	73		718
0	0	1		719
0	0	7		720
0	0	1		721
4	4	18		722
0	0	10		723
0	0	11		724
1	3	14		725
1	1	21		726
0	0	6		727
1	1	2		728
0	0	3		729
0	0	3		730
0	0	13		731
0	0	2		732
0	0	21		733
0	0	81		734
0	0	3		735
2	1	13		736
0	0	4		737
0	0	33		738
2	3	9		739
0	0	1		740
0	0	3		741
0	0	3		742
0	1	8		743
1	1	2		744
0	1	10		745
0	0	4		746
0	1	3		747
0	0	5		748
0	0	7		749
3	3	59		750
1	1	5		751
1	1	7	+	752
0	0	3		753
1	2	19		754
2	5	19		755
0	0	1		756
0	3	24		757
0	1	12		758

0	0	7		+	759
0	0	2		+	760
1	3	12		+	761
0	0	2		+	762
0	0	5		+	763
0	0	1		+	764
12	14	78		+	765
8	8	105		+	766
1	0	1	+	+	767
12	6	42		+	768
8	1	10		+	769
6	2	16		+	770
7	1	18		+	771
9	4	27		+	772
193	219	1253		+	773
0	0	1		+	774
5	7	75		+	775
2	1	19		+	776
51	58	246		+	777
0	5	50		+	778
44	55	348		+	779
5	7	51		+	780
0	1	2		+	781
1	1	4		+	782
22	24	241		+	783
0	0	1		+	784
0	0	9		+	785
1	1	24		+	786
0	0	30		+	787
1	1	18		+	788
0	1	4		+	789
0	1	2		+	790
0	1	7		+	791
0	0	23		+	792
0	0	2		+	793
0	0	4		+	794
2	1	16		+	795
0	0	1		+	796
1	1	6	+	+	797
0	0	13		+	798
0	0	1		+	799
1	0	6		+	800
0	0	40		+	801
1	0	6		+	802
0	1	1	+	+	803

0	0	9	+	804
5	6	50	+	805
0	0	7	+	806
0	0	1	+	807
0	0	26	+	808
0	0	1	+	809
0	0	4	+	810
4	8	44		811
0	0	21		812
0	0	5		813
0	0	2		814
0	1	8		815
0	1	4		816
1	2	15		817
4	5	26		818
0	0	6		819
0	1	7		820
0	3	4		821
2	1	7		822
1	1	4		823
0	0	9		824
1	2	13		825
0	2	6		826
0	0	16		827
10	18	68		828
4	5	18		829
0	0	3		830
1	1	14		831
0	0	1		832
1	0	4		833
0	0	6		834
0	0	10		835
4	9	32		836
1	2	7		837
1	5	26		838
2	1	13		839
0	1	3		840
0	0	10		841
0	1	3		842
0	0	0		843
0	1	7		844
0	1	5		845
1	2	18		846
0	0	3		847
0	1	4		848

0	0	17		849
0	0	3		850
1	0	2		851
2	9	30		852
1	2	26		853
1	1	4		854
0	0	4		855
0	1	61		856
0	0	12		857
1	2	9		858
0	0	1		859
0	0	10		860
1	1	6		861
0	0	5		862
0	1	2		863
0	1	1		864
10	18	122		865
2	3	20		866
0	0	2		867
0	2	43		868
2	7	30		869
0	0	1		870
3	8	32		871
0	0	13		872
0	0	2		873
0	0	3		874
6	10	74		875
0	0	12		876
0	1	3		877
0	0	4		878
22	30	171		879
0	0	1	+	880
0	0	4		881
0	1	21		882
0	0	6		883
3	5	21		884
0	0	2		885
0	0	4		886
6	6	40		887
0	0	1	+	888
0	0	2		889
2	3	23		890
1	1	6		891
9	10	30		892
16	25	130		893

0	0	2			894
0	0	2			895
0	0	2			896
0	0	1			897
0	0	11			898
44	49	369		+	899
0	0	4			900
2	3	31			901
1	4	19			902
1	2	11			903
0	0	4			904
5	7	47			905
0	1	2			906
0	1	3			907
2	5	20			908
0	0	43			909
2	5	28			910
0	0	3			911
0	0	4			912
0	1	4			913
0	1	4			914
2	4	10			915
1	1	2			916
5	7	42			917
1	2	27			918
1	1	3			919
0	1	4			920
1	2	21			921
0	0	3			922
0	1	4			923
0	0	1	+		924
1	2	26			925
23	36	217			926
0	0	10			927
12	21	84			928
0	2	7			929
0	0	1			930
0	0	3			931
3	6	21			932
7	9	67			933
0	0	7			934
0	0	3			935
0	0	15			936
3	4	22			937
2	3	17			938

0	0	3	939
0	1	1	940
1	1	11	941
0	0	5	942
0	0	1	943
0	0	4	944
2	2	11	945
2	7	129	946
0	0	4	947
0	1	5	948
2	1	4	949
0	0	1	950
0	2	18	951
0	0	14	952
0	2	79	953
0	0	1	954
1	1	20	955
0	0	2	956
2	2	42	957
9	14	78	958
0	1	19	959
0	0	17	960
0	1	4	961
1	1	23	962
0	0	3	963
6	9	38	964
0	0	3	965
0	3	18	966
7	12	67	967
0	2	3	968
0	0	9	969
7	11	57	970
0	1	44	971
2	4	14	972
0	0	1	973
3	7	42	974
3	7	45	975
0	0	5	976
0	0	27	977
1	1	7	978
2	3	11	979
0	2	10	980
4	7	35	981
0	1	9	982
3	9	31	983

2	4	13	984
3	9	48	985
1	2	11	986
3	3	34	987
0	0	37	988
4	4	18	989
0	2	2	990
0	0	3	991
0	0	3	992
0	2	27	993
0	0	27	994
0	0	4	995
4	8	53	996
1	0	2	997
3	10	41	998
3	8	28	999
0	0	1	1000
0	0	2	1001
0	1	1	1002
0	0	7	1003
0	0	3	1004
0	0	6	1005
4	3	31	1006
2	4	25	1007
0	0	2	1008
38	43	293	1009
1	3	11	1010
0	0	2	1011
1	2	18	1012
2	1	18	1013
1	0	32	1014
0	0	20	1015
0	0	1	1016
9	10	64	1017
0	1	19	1018
13	18	109	1019
5	6	63	1020
0	0	10	1021
0	0	2	1022
1	2	15	1023
0	3	76	1024
9	10	55	1025
10	18	57	1026
0	1	5	1027
1	2	4	1028

0	0	1	1029
3	8	247	1030
12	20	232	1031
10	15	140	1032
0	0	4	1033
0	3	11	1034
0	1	5	1035
1	1	15	1036
2	3	31	1037
17	27	136	1038
14	22	175	1039
10	9	54	1040
14	13	72	1041
0	0	10	1042
1	1	6	1043
1	0	5	1044
0	0	1	1045
3	4	38	1046
0	0	21	1047
0	1	15	1048
0	1	83	1049
0	0	10	1050
5	9	106	1051
3	5	53	1052
0	1	1	1053
4	9	59	1054
0	0	10	1055
1	1	9	1056
1	1	9	1057
0	0	4	1058
1	5	34	1059
1	5	25	1060
2	3	27	1061
2	1	26	1062
8	12	65	1063
0	1	2	1064
13	23	98	1065
12	13	81	1066
0	0	11	1067
1	1	10	1068
0	0	1	1069
14	18	73	1070
4	4	28	1071
2	0	2	1072
0	0	5	1073

0	0	65	1074
0	1	6	1075
5	4	12	1076
0	0	19	1077
0	0	50	1078
1	3	25	1079
4	5	42	1080
11	12	53	1081
37	47	293	1082
0	0	9	1083
4	8	63	1084
4	4	27	1085
7	8	47	1086
3	9	37	1087
4	7	47	1088
1	1	9	1089
5	6	32	1090
0	0	6	1091
0	0	3	1092
24	32	194	1093
0	1	19	1094
0	1	6	1095
0	0	28	1096
4	7	44	1097
6	12	53	1098
1	2	15	1099
1	1	13	1100
0	0	3	1101
0	0	4	1102
0	0	1	1103
0	1	4	1104
0	0	16	1105
0	0	3	1106
3	3	21	1107
2	1	7	1108
1	0	2	1109
0	2	24	1110
5	10	52	1111
17	26	112	1112
0	2	39	1113
0	0	1	1114
0	0	7	1115
0	0	63	1116
1	3	16	1117
17	18	86	1118

6	21	177	1119
1	2	8	1120
0	0	46	1121
0	0	18	1122
0	0	2	1123
0	2	11	1124
0	0	1	1125
1	1	5	1126
0	0	19	1127
3	3	13	1128
29	44	196	1129
3	6	24	1130
0	5	20	1131
0	0	2	1132
1	1	7	1133
1	0	2	1134
0	0	1	1135
0	0	3	1136
2	3	17	1137
2	2	29	1138
1	2	37	1139
0	1	9	1140
4	5	42	1141
1	1	23	1142
1	3	26	1143
1	1	5	1144
4	5	42	1145
0	1	3	1146
1	2	10	1147
1	2	12	1148
0	2	8	1149
5	5	54	1150
6	7	38	1151
2	3	19	1152
0	0	6	1153
0	0	2	1154
0	1	4	1155
5	7	43	1156
2	5	34	1157
2	5	38	1158
1	1	6	1159
2	5	23	1160
0	0	5	1161
1	5	27	1162
1	0	6	1163

9	18	112	1164
0	1	13	1165
11	15	126	1166
10	17	169	1167
11	15	202	1168
0	0	3	1169
1	2	23	1170
0	2	10	1171
0	0	5	1172
46	77	553	1173
0	1	2	1174
1	2	12	1175
0	2	6	1176
16	16	117	1177
8	11	53	1178
0	0	11	1179
0	2	18	1180
1	1	8	1181
2	4	26	1182
1	0	17	1183
7	17	87	1184
1	2	62	1185
2	2	8	1186
0	0	9	1187
2	5	29	1188
1	2	10	1189
1	6	106	1190
8	17	72	1191
0	0	2	1192
2	4	27	1193
1	1	6	1194
5	6	48	1195
0	0	23	1196
2	6	35	1197
0	0	37	1198
1	1	5	1199
0	2	7	1200
0	0	11	1201
5	5	29	1202
0	0	42	1203
12	14	53	1204
15	15	81	1205
0	0	16	1206
0	0	12	1207
3	6	31	1208

1	6	23		1209
3	2	5		1210
0	0	11		1211
1	1	34		1212
0	0	5		1213
7	10	39		1214
0	0	14		1215
1	2	5		1216
2	2	14		1217
0	0	3		1218
15	20	122		1219
0	0	1	+	1220
5	11	67		1221
0	0	1		1222
5	8	34		1223
0	0	4		1224
0	4	66		1225
18	26	108		1226
0	5	20		1227
0	1	3		1228
0	0	454		1229
6	9	56		1230
0	0	9		1231
1	5	53		1232
1	0	5		1233
0	2	9		1234
0	0	2		1235
1	1	6		1236
0	0	16		1237
0	0	26		1238
10	15	87		1239
1	0	2		1240
18	21	106		1241
2	4	29		1242
2	4	20		1243
1	2	19		1244
0	0	10		1245
2	3	17		1246
0	0	117		1247
2	7	18		1248
0	0	7		1249
0	0	9		1250
2	3	8		1251
0	0	5		1252
0	0	3		1253

1	3	10	1254
0	0	1	1255
0	0	12	1256
0	0	4	1257
0	4	17	1258
2	2	25	1259
0	1	5	1260
0	0	2	1261
9	21	68	1262
3	9	51	1263
0	0	13	1264
0	2	2	1265
0	0	2	1266
0	0	12	1267
1	3	11	1268
0	0	5	1269
0	0	8	1270
4	2	8	1271
0	0	27	1272
1	2	35	1273
6	7	31	1274
2	1	36	1275
0	0	6	1276
1	1	2	1277
0	0	4	1278
0	0	3	1279
5	3	36	1280
1	0	5	1281
0	1	1	1282
0	2	2	1283
2	3	17	1284
0	1	15	1285
0	0	13	1286
10	14	157	1287
3	8	22	1288
0	0	16	1289
0	0	3	1290
0	1	27	1291
0	0	27	1292
0	2	8	1293
0	0	11	1294
0	0	10	1295
0	0	6	1296
23	25	195	1297
1	0	21	1298

0	1	63	1299
0	0	8	1300
2	2	15	1301
0	0	1	1302
0	0	12	1303
0	0	21	1304
0	0	10	1305
2	5	42	1306
0	0	14	1307
0	0	3	1308
0	0	7	1309
0	0	2	1310
0	1	19	1311
2	3	19	1312
0	0	1	1313
1	1	2	1314
0	1	6	1315
0	0	5	1316
1	4	6	1317
0	0	1	1318
0	0	34	1319
0	1	25	1320
0	0	3	1321
0	0	39	1322
1	2	13	1323
0	3	4	1324
0	0	2	1325
0	0	1	1326
0	0	4	1327
0	0	1	1328
0	0	4	1329
1	1	10	1330
1	3	40	1331
3	6	23	1332
3	4	28	1333
5	6	26	1334
0	0	10	1335
2	1	4	1336
3	2	35	1337
0	0	3	1338
0	0	4	1339
20	35	170	1340
0	0	3	1341
1	1	16	1342
0	1	2	1343

0	0	6		1344
0	0	23		1345
0	0	1	+	1346
0	0	16		1347
1	3	18		1348
0	2	15		1349
0	0	2		1350
0	0	6		1351
1	1	29		1352
0	0	4		1353
0	1	5		1354
2	2	11		1355
2	5	9		1356
0	0	3		1357
0	1	10		1358
0	2	3		1359
3	6	22		1360
0	0	5		1361
0	6	23		1362
0	0	113		1363
3	6	21		1364
0	0	2		1365
0	1	1		1366
0	0	62		1367
0	0	33		1368
3	5	14		1369
6	9	35		1370
0	2	14		1371
0	0	2		1372
0	2	6		1373
0	0	13		1374
0	0	12		1375
12	19	95		1376
0	1	15		1377
6	8	275		1378
1	1	4		1379
4	4	22		1380
8	12	75		1381
1	2	31		1382
5	4	17		1383
35	46	229		1384
0	0	4		1385
1	1	5		1386
0	0	3		1387
0	1	4		1388

0	0	5	1389
0	0	4	1390
0	0	5	1391
3	11	40	1392
1	2	6	1393
2	2	15	1394
8	13	63	1395
1	0	3	1396
0	0	14	1397
0	0	19	1398
0	0	3	1399
1	1	6	1400
1	3	18	1401
0	0	4	1402
0	1	8	1403
0	0	27	1404
0	0	6	1405
9	15	103	1406
0	2	2	1407
0	0	3	1408
1	0	45	1409
0	0	5	1410
1	0	11	1411
0	1	13	1412
0	0	25	1413
1	3	149	1414
0	0	10	1415
0	0	2	1416
3	5	20	1417
1	3	8	1418
19	21	142	1419
3	5	25	1420
10	11	58	1421
5	8	31	1422
2	5	61	1423
0	1	3	1424
8	9	49	1425
0	0	18	1426
1	1	24	1427
0	0	19	1428
0	0	11	1429
5	4	24	1430
1	0	7	1431
1	2	11	1432
0	1	1	1433

0	0	38	1434
4	5	21	1435
0	0	3	1436
0	0	6	1437
0	1	4	1438
0	0	61	1439
0	0	18	1440
0	1	4	1441
0	3	8	1442
0	1	16	1443
0	1	1	1444
0	1	17	1445
0	0	4	1446
6	5	30	1447
1	4	36	1448
0	0	5	1449
0	1	4	1450
0	1	142	1451
0	1	5	1452
0	1	6	1453
18	30	88	1454
3	2	13	1455
0	0	2	1456
0	0	3	1457
0	0	26	1458
1	1	122	1459
6	6	31	1460
2	1	7	1461
1	6	86	1462
0	0	2	1463
0	0	12	1464
0	0	1	1465
0	1	6	1466
3	4	16	1467
0	0	1	1468
0	2	9	1469
0	0	2	1470
34	36	203	1471
0	0	32	1472
0	0	3	1473
0	0	3	1474
0	0	59	1475
4	6	19	1476
1	0	5	1477
1	3	7	1478

0	0	5	1479
2	2	17	1480
0	1	3	1481
1	1	4	1482
0	0	4	1483
0	0	3	1484
0	0	11	1485
0	0	13	1486
10	16	89	1487
0	0	3	1488
0	1	7	1489
2	0	6	1490
0	0	35	1491
3	3	19	1492
0	0	8	1493
0	0	4	1494
2	4	19	1495
0	0	5	1496
2	4	23	1497
0	0	1	1498
0	0	4	1499
1	1	3	1500
0	5	33	1501
0	0	45	1502
0	0	12	1503
1	2	21	1504
0	0	15	1505
0	0	32	1506
3	8	63	1507
18	28	145	1508
5	12	78	1509
0	0	2	1510
0	0	16	1511
0	0	6	1512
3	18	63	1513
4	9	45	1514
13	21	107	1515
4	10	81	1516
0	0	81	1517
10	15	80	1518
0	0	3	1519
8	7	29	1520
0	1	5	1521
2	1	8	1522
2	2	16	1523

1	3	9				1524
3	10	36				1525
0	0	52				1526
2	1	6				1527
1	5	59				1528
6	8	64				1529
1	1	17				1530
2	2	19				1531
0	0	2				1532
1	1	5		+		1533
0	1	1		+	+	1534
0	0	1	+	+		1535
1	0	1	+	+		1536
1	1	5		+		1537
0	0	2		+		1538
1	0	1		+		1539
0	0	1		+		1540
1	0	5		+		1541
1	0	1		+		1542
0	0	3		+		1543
0	0	1		+		1544
1	0	2		+		1545
0	0	1		+		1546
1	1	5		+		1547
0	2	6		+		1548
1	1	6		+		1549
0	0	3				1550
9	8	46				1551
1	3	21				1552

Peptide IDs	Peptide is ra	Mod. peptide	Evidence IDs	MS/MS IDs	Best MS/MS	Oxidation (M
993;2655;32	True;True;Tr	1012;2706;3	4547;4548;4	3465;3466;3	3467;9407;11489;14711;3	
189;3577;62	True;True;Tr	194;3636;63	873;874;875;	653;12853;2	653;12853;22457;23262	
666;3047	True;True	681;3100	3054;3055;3	2270;2271;2	2272;10867	
1234;2334;3	True;True;Tr	1259;2379;3	5735;5736;5	4365;4366;4	4371;8331;12883;18482;2	
51;369;3898;	True;True;Tr	52;376;3960;	214;215;216;	165;166;167;	165;1231;14078;18756;36	
2881;3013;8	True;True;Tr	2933;3066;8	13363;13364	10233;10724	10233;10724;31997	
2895	True	2947	13423;13424	10280;10281	10280	
717;2967;42	True;True;Tr	734;3019;42	3301;3302;3	2467;2468;2	2469;10528;15242;31556;	
1984;3099;3	True;True;Tr	2029;3152;3	9428;9429;9	7241;7242;7	7241;11069;12734;13142;	
4914;7432	True;True	4989;7566	22999;23000	17863;17864	17863;26887	
5151;6553;8	True;True;Tr	5228;6678;8	24035;24036	18642;18643	18643;23779;31107;36978	
4010;6465;8	False;True;Tr	4073;6586;8	18693;18694	14451;14452	14452;23441;29820	
940;1454;20	True;True;Tr	959;1483;21	4326;4327;4	3301;3302;3	3304;5219;7493;13392;30	
3407;6878	True;True	3465;7008	15868;15869	12212;25010	12212;25010	
1041;3241;3	True;True;Tr	1062;3295;3	4805;4806;4	3661;3662;1	3662;11586;13278	
9975	True	10142	46540;46541	36193	36193	
501;679;187	True;True;Tr	512;696;191	2301;2302;2	1699;2327;2	1699;2327;6831;9702;102	
5954	True	6040	27819	21620	21620	
389;1214;12	True;True;Tr	396;1238;13	1733;1734;1	1293;1294;1	1295;4286;4547;4688;942	
12;1584;435	True;True;Tr	12;1614;442	59;60;61;746	53;5737;157	53;5737;15758;32537;325	
657;2100;24	True;True;Tr	670;671;672;	2994;2995;2	2229;2230;2	2235;7598;8	0;1
370;3702;62	False;False;T	377;3761;64	1648;1649;1	1233;1234;1	1235;13280;22754;36896	
363;680;120	True;True;Tr	370;697;123	1603;1604;1	1196;1197;1	1196;2330;4268;8525;102	
1000;5603	True;True	1020;5684	4602;4603;4	3503;3504;2	3503;20417	
1196;3706;4	True;True;Tr	1220;3765;4	5538;5539;5	4224;4225;4	4227;13290;14662;15597;	
5503	True	5583	25786;25787	20081;20082	20081	
402;742;200	True;True;Tr	410;759;205	1792;1793;1	1334;1335;1	1335;2562;7295;9542;127	
7668	True	7809	35618;35619	27661	27661	
244;6275;67	True;True;Tr	249;6390;69	1100;29224;	815;22689;2	815;22690;24745;25348;2	
730;840;346	True;True;Tr	747;859;352	3376;3377;3	2528;2529;2	2528;2987;12485;20571;2	
1515;4268;5	True;True;Tr	1544;4335;5	7112;7113;7	5467;5468;5	5470;15404;18539;25918;	
5282;9768	True;False	5361;9934	24703;24704	19168;35527	19168;35527	
5286;9768	True;False	5365;9934	24720;24721	19180;19181	19180;35527	
589;8327	True;True	600;8478	2702;2703;2	1995;1996;3	1995;30165	
1505;3059;9	True;True;Tr	1534;3112;9	7070;7071;7	5440;5441;5	5443;10920;32619	
8700	True	8852	40515;40516	31508	31508	
697;5169;52	True;True;Tr	714;5246;53	3218;3219;3	2396;2397;2	2401;18734;19039;20937;	
1108;9017	True;True	1131;9170	5111;5112;5	3896;3897;3	3897;32594	
8786	True	8939	40974;40975	31846	31846	
7594;7946;8	True;True;Tr	7733;8093;9	35318;35319	27430;28711	27430;28712;32126;33252	
7331	True	7465	34110;34111	26473;26474	26473	
539;600;667;	True;True;Tr	550;612;682;	2474;2475;2	1821;1822;1	1822;2020;2274;6079;984	
4924;5475	True;True	4999;5555	23048;23049	17906;17907	17907;19965	
1431;2034;4	True;False;Tr	1460;2079;4	6702;6703;6	5160;5161;5	5162;7373;16772;22091;3	

2741;3713;5	True;True;Tr	2793;3772;5	12718;12719	9692;9693;9	9693;13352;20850;23014;	
2875;9237	True;True	2927;9394	13320;13321	10193;10194	10193;33458	
1591;6907;9	True;True;Tr	1621;7037;9	7494;7495;7	5758;25102;	5758;25103;33739	
1604;3843	True;True	1634;3904	7557;7558;7	5811;5812;1	5812;13867	
1794;2577;3	False;True;Tr	1833;2627;3	8503;8504;8	6551;6552;6	6553;9155;13710;16300;1	
3350;5817	True;True	3406;5901	15591;15592	11972;11973	11972;21112	
1052;1723;6	True;False;Fa	1073;1760;6	4847;4848;4	3686;6323;2	3686;6323;2	2
2758;6375;1	True;True;Tr	2810;6491;1	12817;12818	9775;9776;2	9775;23099;36373	
1496;4183;4	True;True;Tr	1525;4249;4	7029;7030;1	5405;15103;	5405;15103;17285;23322;	
2049;4023;6	True;True;Tr	2094;4086;7	9690;9691;9	7423;14513;	7423;14517;25347;30538	
1900;3431;4	True;True;Tr	1944;3489;4	8988;8989;8	6910;6911;6	6910;12326;15768;17600;	
569;6603	True;True	580;6729	2625;2626;3	1933;24009;	1933;24009	
193;1087;27	True;True;Tr	198;1110;27	887;888;889;	661;3811;38	661;3812;9658;16924;193	
3253	True	3307	15151;15152	11650;11651	11651	
835;1278;12	False;True;Tr	854;1305;13	3886;3887;3	2976;2977;4	2977;4534;4	3
2693;3534;7	True;True;Tr	2745;3593;7	12493;12494	9525;12696;	9525;12696;28270	
296;1341;41	True;True;Tr	302;1368;42	1308;1309;1	984;4770;47	984;4771;15023;15024;24	
1320;1321;3	True;True;Tr	1347;1348;3	6128;6129;6	4694;4695;4	4695;4698;13145;14006	
4179	True	4245	19485;19486	15090	15090	
1772;1773;6	True;True;Tr	1811;1812;6	8412;8413;8	6477;6478;6	6477;6485;23221;23232;3	
1258;1439;2	True;True;Tr	1284;1468;2	5836;5837;5	4453;4454;4	4455;5185;10412;12025;1	
339;597;319	True;False;Fa	346;608;609;	1490;1491;1	1116;1117;1	1117;2013;1	4;5
584	True	595	2682;2683;2	1973;1974;1	1976	
9375	True	9534	43622;43623	33921;33922	33923	
207;1153;42	True;True;Tr	212;1177;43	937;938;939;	699;700;701;	700;4090;15456;37715	
1803;3089;3	True;True;Tr	1842;3142;3	8545;8546;8	6582;6583;6	6585;11019;	6;7;8
2317;6979	True;True	2362;7110	10822;10823	8269;8270;2	8270;25366	
604;2645;41	True;True;Tr	617;2696;42	2761;2762;2	2032;9350;9	2032;9351;15002;16475;1	
2329;3244;4	True;True;Tr	2374;3298;4	10878;10879	8314;8315;8	8318;11601;17411;25063;	
4603	True	4674	21416;21417	16606;16607	16607	
7805;10265	True;True	7949;10437	36246;36247	28133;37163	28133;37164	
4298;7732	True;True	4365;7875	19988;19989	15542;27889	15542;27890	
1445;1857;6	True;True;Tr	1474;1901;6	6756;6757;6	5197;5198;6	5197;6796;21891;23256;2	
81;99;2172;3	True;True;Tr	83;101;2217;	351;352;353;	263;264;265;	264;329;7823;12150;1337	
44;7563;771	True;True;Tr	45;7700;786	185;35158;3	144;27311;2	144;27312;27851;34206;3	
8500	True	8651	39576;39577	30762	30762	
156;478;253	True;True;Tr	158;487;258	686;687;688;	517;518;163	518;1638;9020;10111;101	
911;3087;58	True;True;Tr	930;3140;59	4202;4203;4	3205;11014;	3205;11015;21357;35798;	
3593;6024;9	True;False;Tr	3652;6110;9	16744;16745	12911;12912	12911;21875;33574	
2632;2687;2	True;True;Tr	2683;2739;3	12195;12196	9311;9497;9	9311;9499;10470;24456;2	
1888;9318	True;True	1932;9477	8939;8940;8	6877;6878;6	6877;33741	
1275;2368;3	True;True;Tr	1302;2413;3	5929;5930;5	4530;4531;8	4531;8442;11749;13577;2	
2234;4853;5	True;True;Tr	2279;4928;5	10488;10489	8022;17602;	8022;17602;19955;24252;	
8317;9869	True;True	8468;10035	38729;38730	30115;35853	30115;35853	
749;4996	True;True	766;5072	3448;3449;3	2587;2588;1	2587;18158	

3326;3902	True;True	3382;3964	15462;15463	11877;14086	11877;14086	
1671;5337;8	True;True;Tr	1707;5416;8	7911;7912;7	6112;19362;	6112;19364;29842;30180	
1369;9593;9	True;False;Fa	1396;9757;9	6373;6374;6	4900;4901;4	4902;34804;35201;37796	
3219;7301	True;True	3273;7434	14970;14971	11502;26372	11502;26374	
2169;2474;5	True;True;Tr	2214;2523;5	10214;10215	7819;8813;1	7819;8813;19353;26234;2	
5451	True	5531	25559;25560	19902;19903	19905	
243;797;221	True;True;Tr	248;816;225	1094;1095;1	810;811;812;	812;2816;7966;10977;110	
1998	True	2043	9477;9478	7274	7274	
8322;9139;9	True;True;Tr	8473;9293;9	38747;38748	30130;30131	30131;33037;33041;36742	
1518;3067;4	True;True;Tr	1547;3120;4	7130;7131;7	5482;10938;	5482;10938;16538;34295;	
7969;8230	True;True	8116;8381	37062;37063	28778;29778	28778;29778	
109;1145;11	True;True;Tr	111;1169;11	472;473;474;	357;358;406	358;4068;4069;12795;145	
7687;9790	True;True	7830;9956	35711;35712	27731;35604	27731;35604	
297;4277;68	True;True;Tr	303;4344;69	1311;1312;1	985;986;154	985;15436;24910;28827;2	
1854;6303;6	True;True;Tr	1898;6418;6	8805;8806;8	6780;6781;6	6780;22795;23768;28975	
432;816;955;	True;True;Tr	440;835;974;	1964;1965;1	1452;2903;2	1452;2904;3346;3409;364	
2485;9922	True;True	2534;10089	11590;11591	8851;36018	8851;36018	
546;635;948;	True;True;Tr	557;648;967;	2511;2512;2	1851;2161;3	1851;2161;3327;6846;948	
2652;2844;3	True;True;Tr	2703;2896;3	12290;12291	9380;9381;9	9381;10102;11449;17079;	
119;3341	True;True	121;3397	521;522;523;	395;11942	395;11942	
2870;2962;3	True;True;Tr	2922;3014;4	13299;13300	10178;10179	10179;10515;14327;1552	
2221;4038;4	True;True;Tr	2266;4101;4	10449;10450	7996;7997;1	7997;14570;17371;18595;	
760;4515;59	True;True;Tr	777;4585;60	3510;3511;3	2633;2634;2	2634;16313;21508;25033;	
1508;5138	True;True	1537;5215	7084;7085;2	5452;18604;	5452;18605	
811;2378;54	True;True;Tr	830;2423;54	3780;3781;3	2882;8472;8	2882;8474;19754;30470	
9470;9492	True;True	9632;9654	44123;44124	34325;34326	34327;34407	
2;10641	True;True	2;10819	14;15;16;17;	11;12;13;14;	12;38539	
255;906;259	True;True;Tr	260;925;265	1146;1147;1	858;859;860;	860;3189;9224;10305;109	
525;1517;15	True;True;Tr	536;1546;15	2420;2421;2	1780;1781;1	1780;5481;5485;7235;112	
949;950;108	True;True;Tr	968;969;111	4361;4362;4	3328;3329;3	3331;3333;3	9
1837;3270;1	True;True;Tr	1880;3324;1	8734;8735;8	6730;6731;6	6731;11702;36768	
121;2024;95	True;True;Tr	123;2069;97	530;531;958	398;7347;73	398;7347;34718	
1892;5161	True;True	1936;5238	8964;8965;8	6894;18687	6894;18687	
2437	True	2483	11346;11347	8653;8654	8654	
4888;7653	True;True	4963;7793	22880;35542	17773;27606	17773;27607	
8032;9786	True;True	8179;9952	37391;37392	29036;35588	29036;35588	
5734	True	5816	26799;26800	20840	20840	
6061	True	6147	28255;28256	21965;21966	21967	
78;214;261;4	True;True;Tr	79;80;219;26	333;334;335;	250;251;252;	255;726;887;	10;11;12;13
6068;7628	True;True	6154;7767	28294;35437	22004;27522	22004;27522	
1735;3115;3	True;True;Tr	1772;3169;3	8241;8242;8	6360;6361;6	6362;11145;11873;18279	
2255	True	2300	10566;10567	8075;8076;8	8075	
4307	True	4374	20024;20025	15569	15569	
208;1095;13	True;True;Tr	213;1118;13	941;942;943;	702;703;704;	703;3856;4880;5909;1169	
2989;5041	True;True	3041;5118	13852;13853	10625;18317	10625;18317	

63	True	64	261;262;263;	198;199;200;	199
503;832;901;	True;True;Tr	514;851;920;	2313;2314;3	1706;1707;2	1707;2959;3173;3387;351
181;6241;62	True;True;Tr	186;6356;63	828;829;830;	620;22580;2	620;22580;22583;28628;2
5350;5604;6	False;True;Tr	5429;5685;6	25009;25010	19404;19405	19404;20421;24932;3679
4024;7080	True;True	4087;7212	18765;18766	14518;14519	14519;25699
9740	True	9906	45434;45435	35350;35351	35350
4146;5749	True;True	4212;5831	19352;26853	14997;20887	14997;20889
2103;2520;4	True;True;Tr	2148;2569;4	9928;9929;9	7607;7608;7	7608;8987;15111;21950;2
7639	True	7778	35482;35483	27561;27562	27561
8956;10499	True;True	9109;10675	41704;41705	32410;32411	32410;38025
809;8005;92	True;True;Tr	828;8152;93	3768;3769;3	2871;2872;2	2875;28943;33403;36746
718;4457;98	True;True;Tr	735;4526;99	3320;3321;3	2485;16076;	2485;16078;35717
372;2959;44	True;True;Tr	379;3011;45	1657;1658;1	1242;1243;1	1242;10505;16055;19445
3716;5926;8	True;True;Tr	3775;6011;8	17305;17306	13360;13361	13360;21500;31920
5679;5709;9	True;True;Tr	5761;5791;9	26563;26564	20671;20782	20671;20786;34177
9723	True	9889	45345;45346	35275;35276	35277
1071;1213;1	True;True;Tr	1092;1237;1	4923;4924;4	3737;3738;3	3737;4283;6887;7028;979
6710;10093	True;True	6839;10261	31421;31422	24447;24448	24449;36588
136;399;400;	True;True;Tr	138;407;408;	604;605;606;	459;460;461;	459;1324;1327;1498;4212
601;739;106	True;True;Tr	613;756;108	2745;2746;2	2021;2022;2	2023;2551;3731;5079;561
3521;5541;7	True;True;Tr	3580;5622;7	16396;16397	12654;20220	12654;20221;25444
1759	True	1796	8346;8347;8	6434;6435;6	6434
1768;5553;7	True;True;Tr	1807;5634;7	8395;8396;8	6464;6465;6	6464;20264;26510;28139;
942;1610;19	False;True;Tr	961;1641;16	4335;4336;4	3308;3309;3	3311;5841;7 14;15
7774;8916	True;False	7918;9069	36108;36109	28027;32289	28027;32289
509;5188;88	True;True;Tr	520;5265;90	2352;24239;	1735;18801;	1735;18802;32190;33617
175;272;865;	False;True;Fa	180;277;884;	805;806;807;	602;603;604;	603;928;3064;4308;7180;
2053;9451	True;True	2098;9613	9711;44040;	7442;34268	7442;34268
878;1880;24	True;True;Tr	897;1924;25	4073;4074;8	3114;3115;6	3115;6858;8868;9587;139
5030	True	5106	23544;23545	18283;18284	18285
212;5936;81	True;True;Tr	217;6022;82	959;960;961;	718;21521;2	718;21521;29385
723;1183	True;True	740;1207	3353;3354;3	2512;2513;2	2512;4188
7056;9934	True;True	7188;10101	32925;32926	25621;25622	25621;36055
4280;6585;7	True;True;Tr	4347;6711;7	19917;19918	15444;15445	15444;23927;26365;2647
1853;5333;9	True;True;Tr	1897;5412;1	8802;8803;8	6779;19352;	6779;19352;35846
1987;9661	True;True	2032;9827	9439;9440;9	7251;7252;3	7251;35068
1497	True	1526	7031;7032	5406;5407	5406
222;237;127	True;True;Tr	227;242;130	1004;1066;1	745;787;453	745;787;4533;9136;10671
908;1706;67	True;True;Tr	927;1743;68	4190;4191;4	3196;3197;6	3197;6251;24595;33118;3
1721;3883;5	True;True;Tr	1758;3945;5	8178;8179;8	6314;6315;6	6314;14010;20768
8884;9244	True;True	9037;9401	41367;41368	32127;32128	32128;33482
4696;9390	True;True	4768;9549	21869;21870	16951;16952	16951;33982
3927;4641;1	True;True;Tr	3989;4712;1	18314;18315	14156;14157	14156;16736;38181;3818
152;8795;95	True;True;Tr	154;8948;96	672;673;674;	505;31868;3	505;31868;34437
1833;2636;4	True;True;Tr	1876;2687;4	8716;8717;8	6719;6720;6	6720;9317;14999;15846;1

1343;8633;8	True;True;Tr	1370;8784;8	6233;6234;6	4781;4782;4	4782;31272;31274;33585;	
190;545;104	True;True;Tr	195;556;106	876;877;878;	654;655;656;	656;1849;3674;8487;9436	
975;1775;32	True;True;Tr	994;1814;32	4469;4470;4	3401;3402;3	3403;6490;11480;12239;1	
7416;8925	True;True	7550;9078	34533;34534	26830;26831	26831;32307	
1043;5026;5	True;True;Tr	1064;5102;5	4811;23530;	3664;18272;	3664;18272;20230	
2804;3826;5	True;True;Tr	2856;3886;5	13004;13005	9938;13814;	9938;13814;20711;33773;	
488;531;266	True;True;Tr	497;542;271	2235;2236;2	1660;1661;1	1660;1799;9439;9991;108	
494;4814;48	True;True;Tr	504;4888;48	2273;2274;2	1686;1687;1	1686;17447;	16
3251;5303	True;True	3305;5382	15139;15140	11639;11640	11644;19247	
513;1030;14	True;True;Tr	524;1050;15	2361;2362;2	1739;1740;1	1739;3618;5360;7259;208	
424;792;123	True;True;Tr	432;811;126	1922;1923;1	1421;1422;2	1421;2797;4372;4377;649	
9298;10037	True;True	9457;10204	43279;43280	33670;36372	33670;36372	
929;1199;53	True;True;Tr	948;1223;54	4279;4280;4	3264;3265;3	3264;4237;19356;24838;2	
4105	True	4168	19121;19122	14810;14811	14811	
80;905;2101;	True;True;Tr	82;924;2146;	348;349;350;	262;3186;31	262;3187;7601;8506;8852	
575	True	586	2641;2642;2	1942;1943	1943	
1022;1309;3	True;True;Tr	1042;1336;3	4711;4712;4	3587;3588;3	3587;4653;11151;14416;1	
2907;4944	True;True	2959;5019	13460;13461	10301;10302	10301;17991	
6488;9391	True;True	6609;9550	30322;30323	23530;33983	23530;33984	
249;626;843;	True;True;Tr	254;639;862;	1119;1120;1	829;830;831;	831;2104;2995;4617;5575	
1104;2544;2	True;True;Tr	1127;2593;2	5094;5095;5	3882;9049;9	3882;9050;9091;10297;11	
1806;1966;1	True;True;Tr	1845;2011;2	8560;8561;8	6595;6596;6	6598;7165;7	17;18
6735;7921	True;True	6864;8068	31529;31530	24539;24540	24539;28606	
4;350;351;10	True;True;Tr	4;357;358;10	26;27;28;29;	23;24;25;26;	25;1151;1165;3660;4277;4	
8444;10621	True;True	8595;10799	39330;39331	30565;30566	30565;38466	
1583;2270;3	True;True;Tr	1613;2315;3	7458;7459;7	5732;5733;5	5734;8121;11260;11577;1	
3503;7747	True;True	3562;7890	16315;16316	12596;12597	12597;27930	
370;3702;37	True;True;Tr	377;3761;37	1648;1649;1	1233;1234;1	1235;13280;13285;22748;	
91;471;1493;	True;True;Tr	93;480;1522;	388;389;215	295;1597;15	295;1598;5397;5710;6999	
885;3289;44	True;True;Tr	904;3343;45	4094;4095;4	3129;3130;1	3129;11766;16061;17601;	
1807;1933;3	True;True;Tr	1846;1977;3	8568;8569;9	6599;7048;7	6599;7052;1	19
67;1582;401	True;True;Tr	68;1612;407	283;284;285;	214;215;216;	216;5731;14452;21476;29	
1516;5293;6	True;True;Tr	1545;5372;6	7118;7119;7	5471;5472;5	5473;19201;23306	
605;1490;20	True;True;Tr	618;1519;20	2764;2765;2	2033;2034;2	2035;5376;7320;7376;737	
3697	True	3756	17210;17211	13270	13270	
3921	True	3983	18290;18291	14141;14142	14141	
15;3183	True;True	15;3237	70;71;72;73;	59;60;61;62;	61;11372	
1113;1170;3	True;True;Tr	1136;1137;1	5142;5143;5	3928;3929;3	3935;4133;1	20
729;1653;49	True;True;Tr	746;1689;50	3373;3374;3	2526;2527;6	2526;6060;18057;35959	
2269;2912;3	True;True;Fa	2314;2964;3	10621;10622	8115;8116;8	8116;10326;12059;12897;	
696;2650;31	True;True;Tr	713;2701;32	3215;3216;3	2395;9376;1	2395;9376;11386;12445;1	
4423	True	4492	20561;20562	15974;15975	15974	
443;4529;70	True;True;Tr	451;4599;71	2009;2010;2	1487;1488;1	1488;16368;25614;28725;	
10090	True	10258	47073;47074	36574;36575	36574	
463;880;370	True;True;Tr	472;899;376	2117;2118;2	1571;1572;1	1573;3118;13299;13511;1	

163;420;359	True;True;Tr	165;428;365	726;727;728;	550;551;552;	550;1400;12924;14308;24	
5024;6923;9	True;True;Tr	5100;7053;9	23525;23526	18269;25156	18269;25156;34646	
314;1128;97	True;True;Fa	320;1152;99	1378;1379;1	1031;4007;3	1031;4007;35527	
280;331;116	True;True;Tr	285;338;118	1255;1256;1	950;1093;10	950;1097;4102;12564;334	
142;468;356	True;True;Tr	144;477;362	631;632;633;	481;482;158	482;1584;12815;13146;13	
354;4451;50	True;True;Tr	361;4520;51	1564;1565;1	1171;1172;1	1172;16063;18288;25025;	
1081;2972;4	True;True;Tr	1102;3024;4	4973;4974;4	3787;3788;1	3787;10547;17547;34302	
7673	True	7814	35636;35637	27672;27673	27673	
3595;5518;9	True;True;Tr	3654;5598;9	16750;16751	12915;12916	12916;20140;35527	
2338;3997;4	True;True;Tr	2383;4060;4	10920;10921	8341;14401;	8341;14402;17787;27310	
615;2228;32	True;True;Tr	628;2273;32	2812;2813;2	2076;2077;2	2078;8013;11558;24925;2	
4571;7872	True;True	4642;8018	21273;21274	16509;28372	16509;28372	
4173;6408	True;True	4239;6529	19461;19462	15072;23241	15072;23241	
348;4522;74	True;True;Tr	355;4592;76	1528;1529;1	1141;16346;	1141;16347;27054;31554	
4058;4093	True;True	4121;4156	18917;19076	14654;14778	14654;14780	
311;312;405	True;True;Tr	317;318;411	1369;1370;1	1024;1025;1	1025;1027;14639;14655;1	
289;301;405	True;True;Tr	294;307;411	1285;1286;1	970;996;997;	970;997;14642;30690	
1419;1787;3	True;True;Tr	1448;1826;3	6643;6644;6	5114;5115;5	5115;6523;11539;12494;1	
307;9693	True;True	313;9859	1355;1356;1	1013;1014;3	1014;35164	
5;140;638;11	True;True;Tr	5;142;651;11	32;33;34;35;	29;30;31;477	29;478;2170;4030;4281;43	
54;1514;155	True;True;Tr	55;1543;158	226;227;228;	170;171;546	171;5466;5633;19339;207	
138;378;130	True;True;Tr	140;385;133	620;621;168	473;474;125	473;1257;4637;8787;1907	
4786	True	4860	22316;22317	17323;17324	17324	
1457	True	1486	6810;6811;6	5233;5234;5	5233	
9729	True	9895	45376;45377	35302;35303	35302	
143	True	145	634;635;636	483	483	
382;8517	True;True	389;8668	1697;1698;1	1266;1267;1	1266;30828	
6127	True	6219	28531;28532	22183;22184	22184	
529;794;347	True;True;Tr	540;813;353	2435;2436;2	1792;1793;2	1793;2805;12501;13400;2	
5065;7042	True;True	5142;7174	23691;32870	18396;25587	18396;25587	
172;6566	False;True	177;6691	793;794;795;	594;23819	594;23819	
326;8789	True;True	333;8942	1435;1436;1	1073;1074;1	1074;31853	
318;4544;51	True;True;Tr	324;4614;52	1394;1395;1	1041;1042;1	1041;16421;18588;30542;	
2646;2647;3	True;True;Tr	2697;2698;3	12253;12254	9354;9355;9	9356;9361;1	21
1612;3050;6	True;False;Tr	1644;3103;6	7607;7608;7	5852;5853;5	5852;10880;22224	
287;7570	True;True	292;7708	1280;1281;3	966;967;273	966;27346	
1315;10241	True;True	1342;10413	6109;6110;6	4672;37095;	4672;37096	
108;1381	True;True	110;1408	470;471;643	355;356;494	356;4947	
612;3181;98	True;True;Tr	625;3235;10	2800;2801;2	2069;2070;1	2070;11368;35750	
2564	True	2614	11937;11938	9112;9113	9113	
2025;2427;4	True;True;Tr	2070;2473;4	9587;9588;9	7350;8632;1	7350;8632;16739;19311;2	
2705;4143;6	True;True;Tr	2757;4209;6	12547;12548	9576;14986;	9576;14986;22496;27108	
1329;2576;3	True;True;Tr	1356;2626;3	6166;6167;6	4725;4726;4	4726;9152;12970;18227;1	
8238	True	8389	38372;38373	29811;29812	29811	
1946;4328;5	True;True;Tr	1990;4396;5	9226;9227;9	7093;7094;7	7095;15650;19249;25706	

8937;8985;9	True;True;Tr	9090;9138;9	41615;41616	32338;32339	32338;32508;34315;35549	

Q5W9R8;Q5R1M1;Q5R1M0;Q5NTA3;Q5EPE9;Q546Q4;Q546F7;Q540M3;Q4ZG94;Q4ZG93;Q4W6C5;Q4W6C3;Q4W6C0;Q4W
;F8R117;F8R116;F8R115;F8R114;F8R113;F8R112;F8R111;F8R110;F8R109;F8R108;F8R106;F8R
Y63;Q60I39;Q5W9R8;Q5R1M1;Q5R1M0;Q5NTA3;Q5EPE9;Q546Q4;Q546F7;Q540M3;Q4ZG94;Q4ZG93;Q4W6C5;Q4W6C3;
;F8R117;F8R116;F8R115;F8R114;F8R113;F8R112;F8R111;F8R110;F8R109;F8R108;F8R106;F8R
273;273;273;273;273;273;273;273;273;273;273;273;273;273;273;273;273;273;273;273;273;273;273;273;273;273;273;273;273;273;273

1375;1712;1	True;True;Tr	1402;1749;1	6405;6406;6	4922;4923;6	4923;6270;6820;8454;224	
594;1413;16	True;True;Tr	605;1442;16	2724;2725;2	2008;5091;5	2008;5091;5956;7656;793	
3530	True	3589	16431	12685	12685	
8460;9832	True;True	8611;9998	39392;45933	30615;35737	30615;35737	
9549	True	9711	44523;44524	34628;34629	34629	
3041;3042;5	False;True;Fa	3094;3095;5	14142;14143	10849;10850	10850;10852;19844;36344	
65;7826	True;True	66;7972	269;270;271;	204;205;282	205;28218	
4156;6941;8	True;True;Tr	4222;7072;8	19389;19390	15026;15027	15026;25265;31897;37824	
6795	True	6925	31827	24775	24775	
7004	True	7135	32693;32694	25448;25449	25448	22
516;517;823;	True;True;Tr	527;528;842;	2370;2371;2	1746;1747;1	1749;1753;2919;6392;809	
9218	True	9375	42932;42933	33396	33396	
9;5255	True;True	9;5333	50;51;52;245	45;46;19046;	45;19047	
2618;8481	True;True	2669;8632	12144;12145	9276;30692;	9276;30693	
453;1330;51	True;True;Tr	461;1357;51	2049;2050;2	1511;1512;1	1511;4728;18560;20832	
7239	True	7372	33695;33696	26160;26161	26160	
4041;6018;1	True;True;Tr	4104;6104;1	18834;18835	14575;14576	14576;21854;36952;37597	
7142;9333	True;True	7275;9492	33283;33284	25867;33783	25867;33784	
5284	True	5363	24708;24709	19170;19171	19171	
113;185;186;	True;True;Tr	115;190;191;	489;490;491;	370;371;372;	376;636;643;	23
1548;2212;2	True;True;Tr	1577;2257;2	7284;7285;7	5593;5594;5	5595;7967;10359;15462;2	
7779	True	7923	36132;36133	28047;28048	28047	
2003;2949;3	False;True;Tr	2048;3001;3	9496;9497;9	7287;7288;7	7291;10477;12707;23064;	
812;1207;21	True;True;Tr	831;1231;22	3783;3784;3	2883;4269;4	2883;4271;7911;15260	
1412;2084;5	True;True;Tr	1441;2129;5	6615;6616;6	5089;5090;7	5089;7543;18316	
2560	True	2609	11921;11922	9100;9101;9	9101	
31;3507	True;True	32;3566	135;136;137;	112;113;126	112;12611	
8455	True	8606	39368;39369	30592;30593	30592	
3566	True	3625	16598;16599	12808;12809	12809	
5187	True	5264	24227;24228	18792;18793	18799	
4249;6655;7	True;True;Tr	4316;6782;7	19786;19787	15333;24236	15333;24237;26315;31385	
853;1632;23	True;True;Tr	872;1668;23	3961;3962;3	3029;3030;5	3029;5951;8214;10282;11	
8419;8467;1	True;True;Tr	8570;8618;1	39217;39218	30457;30458	30458;30635;38391	
2094	True	2139	9883;9884;9	7572	7572	
8349	True	8500	38894;38895	30237;30238	30239	
2047;2436;6	True;True;Fa	2092;2482;6	9688;11343;	7421;8652;2	7421;8652;22817	
10413	True	10588	48581;48582	37667;37668	37668	
7317	True	7450	34038;34039	26423;26424	26424	

1555;4408;9!	True;True;Tr	1584;4477;9:	7320;7321;7:	5619;5620;5(	5620;15915;34750
2251;2739;4:	True;True;Tr	2296;2791;4!	10544;10545	8059;9687;9(	8059;9688;17170
8662	True	8814	40331;40332	31367;31368	31368
4435	True	4504	20617;20618	16019	16019
1747;3812;5:	True;True;Tr	1784;3872;5:	8290;8291;8:	6397;13765;:	6397;13765;18524;19008;
1193	True	1217	5523;5524;5!	4215;4216;4:	4216
6825	True	6955	31943	24861	24861
4055;8910;8!	False;False;T	4118;9063;9(	18901;18902	14643;14644	14645;32278;32283
7816	True	7961	36299;36300	28174;28175	28175
2087;2551;3:	True;True;Tr	2132;2600;3·	9857;9858;9!	7551;7552;7!	7551;9074;12049;19891
1581;2509;3(	True;True;Tr	1611;2558;3(	7449;7450;7·	5725;8916;8!	5725;8917;10864;10996
118;2341;28!	True;True;Tr	120;2386;29(	516;517;518;	390;391;392;	391;8352;10142;11377;11
4155	True	4221	19386;19387	15025	15025
116;630;196!	True;True;Tr	118;643;201:	507;508;509;	382;383;211:	382;2119;7177;10385;220
5572;7494	True;True	5653;7631	26099;26100	20313;20314	20318;27110
410	True	418	1835;1836;1!	1358	1358
9764	True	9930	45620;45621	35514;35515	35515
3430	True	3488	15983;15984	12323;12324	12325
656;4770;71!	True;True;Tr	669;4844;72!	2991;2992;2!	2227;2228;1:	2228;17275;25904;38212
835;1634;24!	False;False;F	854;1670;24!	3886;3887;3!	2976;2977;5!	2977;5955;8697;17741;18
1976;2585;3(	True;True;Tr	2021;2635;3:	9387;9388;9:	7211;9174;9:	7211;9175;13244;15588
1988;2311;3:	True;True;Tr	2033;2356;3:	9444;9445;9·	7253;7254;7:	7255;8245;11846;26721;3(
5950	True	6036	27801;27802	21606;21607	21608
1050;8752;9:	True;True;Tr	1071;8905;9!	4840;4841;4!	3681;3682;3(	3683;31731;35551
1192;2375;3(	True;True;Tr	1216;2420;3(	5521;5522;1:	4214;8464;8·	4214;8465;10750;13579;1
336;337	True;True	343;344	1482;1483;1·	1110;1111;1:	1110;1112
7838	True	7984	36387;36388	28247;28248	28249
82;686;951;1	True;True;Tr	84;703;970;1	357;358;359;	269;270;235:	270;2354;3334;6021;1032
1715;1749;1!	True;True;Tr	1752;1786;1!	8135;8136;8:	6277;6278;6:	6280;6402;6896;26214
701;1251;14:	False;False;F	718;1277;14!	3239;3240;3:	2413;2414;4·	2413;4443;5141;8503;129
2894	True	2946	13417;13418	10277;10278	10277
567	True	578	2619;2620;2(	1930	1930
5411;7090;7!	True;True;Tr	5491;7222;8(	25391;25392	19772;19773	19773;25716;28673;34961
1018;1922;3:	True;False;Fa	1038;1966;3:	4697;4698;4(	3581;7016;7(	3581;7016;11430;16709;2:
1067;1452	True;True	1088;1481	4909;4910;4!	3730;5213	3730;5213
9814	True	9980	45851;45852	35688;35689	35689
1301;1388;1(	True;True;Tr	1328;1416;1(	6048;6049;6(	4626;4627;4(	4626;4969;6028;6969;704
766;767;891;	True;True;Tr	783;784;785;	3538;3539;3!	2652;2653;2(	2655;2670;3: 24;25;26;27;:
10276	True	10448	47941;47942	37196	37196
4461;6499;7(	True;True;Tr	4530;6620;7:	20720;20721	16088;23558	16088;23558;27472;37110
1423;2605;2!	True;True;Tr	1452;2656;2!	6664;6665;6(	5132;5133;5:	5133;9240;9963;27540;30
5146	True	5223	24011;24012	18623;18624	18623
6949	True	7080	32471;32472	25289;25290	25290
3804;4931	True;True	3863;5006	17762;17763	13729;13730	13729;17926
7731	True	7874	35930;35931	27888	27888

72;2886;568	True;True;Tr	73;2938;577	309;310;311;	233;10246;1	233;10247;20709;25799
1228;5911;6	True;True;Tr	1252;5996;6	5696;5697;5	4337;4338;2	4338;21447;23493;31808
5210;5985	True;True	5287;6071	24355;24356	18895;18896	18896;21720
316;3167;65	True;True;Fa	322;3221;67	1384;1385;1	1033;1034;1	1034;11305;23890;23911;
10231	True	10403	47745;47746	37053;37054	37055
8501	True	8652	39579;39580	30763;30764	30763
1157;3463;8	True;True;Tr	1181;3521;8	5365;5366;5	4097;12451;	4097;12451;30248
8402	True	8553	39137;39138	30413	30413
251;442;690;	True;True;Tr	256;450;707;	1124;1125;1	833;834;835;	833;1486;2371;6363;9469
9351	True	9510	43512;43513	33849;33850	33851
2111;2985;4	True;True;Tr	2156;3037;4	9966;9967;9	7636;7637;7	7637;10605;15461;17664;
655;2600;30	True;True;Tr	668;2651;31	2985;2986;2	2222;2223;2	2223;9226;10929;12740;1
358;1024;21	True;True;Tr	365;1044;21	1578;1579;1	1179;3593;7	1179;3593;7732;8020;899
708;1118;22	True;True;Tr	725;1142;23	3272;3273;3	2443;2444;3	2443;3949;8159;9056;905
3868;5807;8	True;True;Tr	3930;5891;8	18050;18051	13961;13962	13961;21076;31700
611;5060;72	True;True;Tr	624;5137;73	2798;2799;2	2067;2068;1	2067;18379;26165;36068
1186;3259;8	True;True;Tr	1210;3313;8	5494;5495;5	4194;4195;4	4195;11676;31591;32156;
6952	True	7083	32479;32480	25293;25294	25293
1418;1526;1	True;True;Tr	1447;1555;1	6641;6642;7	5113;5506;5	5113;5506;5738;5762;612
1534;3361;3	True;True;Tr	1563;3417;3	7215;7216;7	5541;5542;5	5543;12018;13519;16090;
218;1312;15	True;True;Tr	223;1339;15	985;986;987;	733;4665;46	733;4665;5514;7707;1152
2143;3683	True;True	2188;3742	10105;17153	7736;13225;	7736;13225
236;678;485	True;True;Tr	241;695;493	1063;1064;1	786;2326;17	786;2326;17610
268;293;467	True;True;Tr	273;299;474	1210;1211;1	915;916;917;	919;978;16830;32285
17;325;777;8	True;True;Tr	17;332;796;8	79;80;81;143	64;65;66;106	64;1070;2728;2964;3236;
3887	True	3949	18132;18133	14022;14023	14023
6824;10104;	True;True;Tr	6954;10272;	31942;47151	24860;36636	24860;36636;36637
543;624;223	True;True;Tr	554;637;227	2495;2496;2	1839;1840;1	1840;2098;8018;15037;18
5646	True	5727	26428;26429	20552;20553	20552
2127;2800;3	True;True;Tr	2172;2852;3	10048;10049	7697;9924;9	7697;9926;10902;15078;1
1366;4161	True;True	1393;4227	6358;6359;6	4889;15035;	4889;15035
1477	True	1506	6921;6922;6	5326;5327;5	5326
1056;1483;3	True;True;Tr	1077;1512;3	4862;6949;6	3697;5346;5	3697;5347;12769;16143;1
5173	True	5250	24180;24181	18749;18750	18749
4177;5091	True;True	4243;5168	19473;19474	15081;15082	15081;18480
5386;6516;8	True;True;Tr	5465;6637;8	25233;25234	19587;23637	19587;23638;29144
640;6496;10	True;True;Tr	653;6617;10	2918;2919;2	2176;2177;2	2177;23549;37048
1792;5372;6	True;True;Tr	1831;5451;6	8492;8493;2	6543;19519;	6543;19519;24373;25307
819;5673;77	True;True;Tr	838;5754;79	3813;3814;3	2911;2912;2	2911;20648;28010;33476
115;10488	True;True	117;10664	504;505;506;	381;38000	381;38000
133;818;340	True;True;Tr	135;837;346	592;593;594;	454;455;456;	456;2910;12204;20291;23
2887;3163;5	True;True;Tr	2939;3217;5	13384;13385	10248;11296	10248;11296;20570;26714
141;10393	True;True	143;10568	628;629;630;	479;480;376	480;37600
45;964;997;1	True;True;Tr	46;983;1017;	186;187;188;	145;146;336	145;3372;3489;5022;7578
636;1248;59	True;True;Tr	649;1274;60	2901;2902;2	2162;2163;2	2162;4424;21644;34598

1681;2242;3	True;True;Tr	1718;2287;3	7969;7970;7	6146;6147;6	6148;8040;12105;13508;1	
558;2339;29	True;True;Tr	569;2384;30	2588;2589;2	1908;8342;8	1908;8344;10632;22634;3	
1512;2970;5	True;True;Tr	1541;3022;5	7101;7102;7	5462;5463;5	5464;10542;19275;20446;	
6277	True	6392	29229;29230	22693	22693	
1492;1990;1	True;True;Tr	1521;2035;2	7006;7007;7	5390;5391;5	5391;7262;7263;10480;13	
6279	True	6394	29237;29238	22699;22700	22701	
1280;2488;2	True;True;Tr	1307;2537;2	5943;5944;5	4541;4542;4	4541;8857;9289;13295;13	
504;873;349	False;False;F	515;892;355	2315;2316;2	1708;1709;1	1716;3092;12566;16276;1	
7296	True	7429	33952;33953	26357	26357	
1268;4087;4	True;True;Tr	1295;4150;4	5896;5897;5	4504;4505;4	4504;14763;17578;21634;	
1339;8306;8	True;True;Tr	1366;8457;8	6213;6214;6	4764;30072;	4764;30072;30357;32003	
4055;4056	False;True	4118;4119	18901;18902	14643;14644	14645;14646	
4549;7341;9	True;True;Tr	4620;7475;9	21166;21167	16434;16435	16434;26508;34824	
5889;10111	True;True	5973;10279	27481;27482	21367;36652	21367;36652	
6729;7073;9	True;True;Tr	6858;7205;1	31505;31506	24518;24519	24522;25686;35764	
5046	True	5123	23620;23621	18340;18341	18340	
9860	True	10026	46053;46054	35826;35827	35826	
2648	True	2699	12270;12271	9366;9367;9	9368	
8514;9479;9	True;True;Tr	8665;9641;9	39642;39643	30821;30822	30822;34349;35092	
4255;6652	True;True	4322;6779	19813;19814	15351;24227	15351;24227	
200;919;176	False;False;F	205;938;179	914;4238;42	677;3231;32	677;3232;64	32
359;747;851;	True;True;Tr	366;764;870;	1581;1582;1	1180;1181;1	1180;2581;3025;3734;474	
6743;8630	True;True	6872;8781	31571;31572	24572;24573	24572;31250	
1377;3541;4	True;True;Tr	1404;3600;4	6420;6421;6	4935;4936;1	4935;12721;14950;16971;	
689;5463;55	True;True;Tr	706;5543;56	3178;3179;2	2369;19937;	2369;19937;20168;20821;	
32;149;2006;	True;True;Tr	33;151;2051;	138;658;659;	114;499;500;	114;501;7297;8633;21889	
877	True	896	4068;4069;4	3112;3113	3113	
3094;6001	True;True	3147;6087	14396;28031	11038;21782	11038;21782	
436;870;168	True;True;Tr	444;889;172	1984;1985;1	1467;1468;1	1468;3075;6173;6338;881	
10185	True	10356	47556;47557	36919;36920	36919	
10646	True	10824	49741;49742	38558	38558	
599;5740;58	True;True;Tr	611;5822;59	2739;2740;2	2016;2017;2	2017;20854;21181;22894	
4626	True	4697	21527;21528	16687;16688	16687	
464;673;268	True;True;Tr	473;690;273	2123;2124;3	1576;1577;2	1576;2307;9481	
987;2218;22	True;True;Tr	1006;2263;2	4524;4525;4	3441;7986;7	3441;7987;8127;15230;16	
4933;6918;1	True;True;Tr	5008;7048;1	23092;23093	17937;17938	17939;25139	33
2404;7288	True;True	2449;7421	11193;11194	8534;8535;8	8534;26334	
448;3021;55	True;True;Tr	456;3074;56	2030;2031;2	1500;1501;1	1500;10742;20280	
552;3634;39	True;True;Tr	563;3693;40	2550;2551;2	1879;1880;1	1881;13048;14403;23539	
3319;3540;7	True;True;Tr	3374;3599;7	15428;15429	11854;11855	11856;12715;27818;3308	
10434	True	10609	48679;48680	37740	37740	
4053;7811	True;True	4116;7956	18895;18896	14641;28151	14641;28152	
180;199;445;	False;False;T	185;204;453;	825;826;827;	618;619;675;	618;676;1496;2951;6433;	
1254;3236;3	True;True;Tr	1280;3290;3	5827;5828;1	4446;4447;1	4446;11566;11682;21258;	
7327	True	7460	34084;34085	26454;26455	26457	

1127;3828;5	True;True;Tr	1151;3888;5	5238;5239;5	4003;4004;4	4003;13822;18606;20757;	
178;179;133	False;False;T	183;184;136	816;817;818;	611;612;613;	615;617;4744;5285;12320	
7084;8352;1	True;True;Tr	7216;8503;1	33042;38902	25703;30245	25703;30247;36381	
745;966;120	False;False;F	762;985;122	3433;3434;3	2571;2572;2	2571;3375;4	13
566;5205;97	True;True;Tr	577;5282;99	2618;24317;	1929;18863;	1929;18863;35571	
981;2382;49	True;True;Tr	1000;2427;5	4495;4496;4	3422;3423;3	3422;8484;18016;21705;2	
4055;4057	False;True	4118;4120	18901;18902	14643;14644	14645;14652	
4028;5877;9	True;True;Tr	4091;5961;9	18777;18778	14525;14526	14527;21335;33439	
2114;2615;3	True;True;Tr	2159;2666;3	9990;9991;1	7655;9268;1	7655;9268;13066;16160;2	
636;1248;34	False;False;T	649;1274;35	2901;2902;2	2162;2163;2	2162;4424;12499;21644	
1606	True	1636	7566;7567;7	5818;5819;5	5818	
645;2670;33	True;True;Tr	658;2721;34	2942;2943;2	2193;2194;9	2193;9442;12020;21759;3	
771;931;957;	True;True;Tr	790;950;976;	3582;3583;3	2694;2695;2	2697;3273;3355;4339;601	
5731;7307;9	True;True;Tr	5813;7440;9	26790;26791	20834;26390	20834;26391;33455	
2209;7711;9	True;True;Tr	2254;7854;9	10401;10402	7964;27812;	7964;27812;35106	
43	True	44	183;184	143	143	
2614;3805;4	True;True;Tr	2665;3864;3	12129;12130	9265;9266;9	9267;13734;	34;35
9307	True	9466	43315;43316	33699;33700	33699	
7589;8965;9	True;True;Tr	7728;9118;9	35295;35296	27415;32442	27415;32443;34808	
486;1821;30	True;True;Tr	495;1862;31	2224;2225;2	1655;6654;1	1655;6654;10971;13951;1	
66;722;1265;	True;True;Tr	67;739;1292;	275;276;277;	206;207;208;	207;2505;4494;19561;321	
5769;7320;8	True;True;Tr	5852;7453;8	26942;26943	20944;26432	20944;26432;30366	
376;1126;14	True;True;Tr	383;1150;14	1674;1675;1	1254;1255;4	1254;4002;5191;13248;25	
5685;9674;9	True;True;Tr	5767;9840;1	26595;26596	20697;20698	20698;35114;35744	
5656	True	5737	26460;26461	20573;20574	20573	
137;4834;51	True;True;Tr	139;4909;52	608;609;610;	462;463;464;	463;17536;18578;19341;2	
4539;6177	True;True	4609;6278	21124;21125	16408;22366	16408;22366	
695;3150;46	True;True;Tr	712;3204;46	3209;3210;3	2390;2391;2	2393;11261;16626;34466	
428;1263;13	True;True;Tr	436;1290;13	1948;1949;1	1441;1442;1	1443;4484;4649;9082;942	
92;345;1298;	True;True;Tr	94;352;1325;	390;391;392;	296;297;298;	303;1133;46	36;37;38
25;134;1267;	True;True;Tr	26;136;1294;	111;112;113;	92;93;457;45	92;457;4502;7046;10362;1	
6136;8058	True;True	6229;8205	28568;28569	22212;22213	22213;29129	
528;1310;53	True;True;Tr	539;1337;54	2429;2430;2	1786;1787;1	1789;4658;19404;23124;2	
7662	True	7803	35592;35593	27648;27649	27648	
3198;6276	True;True	3252;6391	14862;14863	11415;11416	11417;22692	
1310;2039;5	False;True;Fa	1337;2084;5	6086;6087;6	4655;4656;4	4658;7388;19404;24933	
322	True	328	1415;1416;1	1053;1054;1	1058	
2062;2659;3	True;True;Tr	2107;2710;3	9733;12343;	7457;9421;9	7457;9422;13865;25688;2	
2663;2714;3	True;True;Tr	2714;2766;3	12354;12355	9427;9611;9	9427;9612;13058;16069;2	
2472;4690;6	True;True;Tr	2521;4762;6	11528;11529	8806;8807;8	8808;16937;24376;27459;	
765;2619;69	True;True;Tr	782;2670;71	3535;3536;3	2651;9277;2	2651;9277;25391	
3200;5468;6	True;True;Tr	3254;5548;6	14866;14867	11419;11420	11419;19946;23384;23791	
3213;5735;9	False;True;Tr	3267;5817;1	14932;14933	11470;11471	11480;20843;35931	
2733;3080;3	True;True;Tr	2785;3133;3	12694;12695	9677;10993;	9677;10993;11150;13366;	
2611	True	2662	12122;12123	9258;9259;9	9260	

5492;8214	True;True	5572;8365	25730;25731	20036;20037	20038;29724	
5927	True	6012	27668;27669	21501;21502	21502	
320;422;890	True;True;Tr	326;430;905	1403;1404;1	1046;1047;1	1049;1417;32228	
4805;6430;6	True;True;Tr	4879;6551;6	22403;22404	17402;17403	17402;23298;23301	
2575;3164;3	True;True;Tr	2625;3218;3	11978;11979	9147;9148;9	9151;11297;12534;27434	
200;304;919;	False;True;Fa	205;310;938;	914;1342;42	677;1005;32	677;1005;32	32
2863;3596;5	True;True;Tr	2915;3655;5	13270;13271	10156;10157	10157;12917;19972;2910	
79;1054;359	True;True;Tr	81;1075;365	342;343;344;	259;260;261;	261;3691;12921;18203;19	
1098	True	1121	5071	3862	3862	
1609;2535;1	True;True;Tr	1640;2584;1	7581;7582;7	5831;9029;9	5831;9029;38474	
1521;5856;5	True;True;Tr	1550;5940;6	7148;7149;7	5493;5494;5	5494;21261;21656	
2728;2802;2	True;True;Tr	2780;2854;2	12662;12663	9652;9653;9	9655;9931;10013;13447;1	
1562;1579;1	True;True;Tr	1591;1609;1	7355;7356;7	5646;5647;5	5646;5721;6062;9604;109	
452;785;187	True;True;Tr	460;804;192	2045;2046;2	1509;1510;2	1510;2778;6850;7193;800	
2056;9508	True;True	2101;9670	9716;9717;9	7446;7447;3	7446;34459	
1667;2543;2	True;True;Tr	1703;2592;2	7899;7900;7	6103;6104;6	6103;9048;9263;14885;27	
2197;6648	True;True	2242;6775	10354;10355	7929;7930;7	7931;24218	
9228	True	9385	42978;42979	33435	33435	39
9083	True	9237	42289;42290	32856;32857	32857	
5718;9043	True;True	5800;9196	26749;26750	20812;20813	20814;32698	
76;2851;711	True;True;Tr	77;2903;724	326;327;328;	245;10119;1	245;10119;25793;26212;3	
2031;2765;3	True;True;Tr	2076;2817;3	9608;9609;9	7361;7362;7	7361;9789;11111;11784;1	
3498;9823	True;True	3557;9989	16297;45884	12579;35706	12579;35706	
1204;3980;9	True;True;Tr	1228;4043;1	5578;5579;5	4254;14333;3	4254;14333;35871	
3342;3343	True;True	3398;3399	15545;15546	11943;11944	11943;11947	
647;1288;12	True;True;Tr	660;1315;13	2951;2952;2	2198;2199;2	2198;4571;4	40
6739	True	6868	31553;31554	24561;24562	24562	
1189;2337;2	True;True;Tr	1213;2382;2	5508;5509;5	4203;4204;8	4204;8338;9171;9956;124	
232;485;556;	True;True;Tr	237;494;567;	1045;1046;1	772;1653;16	772;1653;1899;5165;5637	
1697;2822	False;True	1734;2874	8060;8061;8	6225;6226;6	6225;9981	
206;2641;29	True;True;Tr	211;2692;29	934;935;936;	697;698;934	697;9342;10382;22202	
112;340;298	True;True;Tr	114;347;303	480;481;482;	362;363;364;	363;1119;10585;11165;25	
7049	True	7181	32903;32904	25608	25608	
2151;3850;5	True;True;Tr	2196;3911;5	10136;10137	7759;7760;1	7760;13885;19700;35994	
131;5419;72	True;True;Tr	133;5499;73	579;580;581;	444;445;446;	449;19792;26046	
8485	True	8636	39496;39497	30698	30698	
1155;10081	True;True	1179;10249	5359;5360;5	4094;4095;3	4095;36539	
1019;1922;3	True;True;Tr	1039;1966;3	4700;4701;4	3582;3583;3	3584;7016;11430;17237;2	
1731;6618	True;True	1768;6744	8230;8231;8	6352;6353;6	6354;24061	
5008;7790	True;True	5084;7934	23443;23444	18205;28090	18205;28092	
6742	True	6871	31568;31569	24571	24571	
1365;1905;2	True;True;Tr	1392;1949;2	6355;6356;6	4887;4888;6	4888;6976;8416;9248;114	
2272;2529;2	True;True;Tr	2317;2578;2	10637;10638	8129;8130;9	8129;9011;9013;14622;22	
6411	True	6532	29939;29940	23247	23247	
1376;1639;4	True;True;Tr	1403;1675;4	6408;6409;6	4924;4925;4	4927;6001;1	41

2010;8881;9	True;True;Tr	2055;9034;9	9523;9524;9	7308;7309;7	7308;32115;	42
4623;5085	True;True	4694;5162	21509;21510	16675;16676	16676;18453	
3025;5377;5	True;True;Tr	3078;5456;5	14026;14027	10752;10753	10755;19540	43;44
3237;4538;5	True;True;Tr	3291;4608;6	15050;15051	11567;11568	11567;16407;21546;22892	
276;1685;65	True;True;Tr	281;1722;67	1240;1241;1	937;938;939;	938;6169;23890;23895;24	
9070	True	9224	42228;42229	32812;32813	32812	
833;921;411	False;False;F	852;940;417	3869;3870;3	2960;2961;2	2964;3236;14853;15890;2	
231;2109;23	True;True;Tr	236;2154;24	1043;1044;9	771;7634;84	771;7634;8411;20837;269	
1836;6108;7	True;True;Tr	1879;6197;7	8731;8732;8	6729;22130;	6729;22130;25697;27220;	
8035	True	8182	37402;37403	29042;29043	29043	
1709;2992;4	True;True;Tr	1746;3044;4	8109;8110;8	6262;6263;6	6263;10638;	45
394;451;130	True;True;Tr	402;459;133	1753;1754;1	1307;1308;1	1310;1508;4645;4908;620	
5733;9943	True;True	5815;10110	26796;26797	20838;20839	20839;36088	
557;3158;60	True;True;Tr	568;3212;61	2582;2583;2	1902;1903;1	1904;11280;21942;24924	
1819;8975	True;True	1860;9128	8642;8643;8	6651;6652;3	6652;32473	
479;8261;92	True;True;Tr	488;8412;93	2200;2201;2	1639;29874;	1639;29874;33324	
6844	True	6974	32014;32015	24918;24919	24920	
397;1156;24	True;True;Tr	405;1180;24	1769;1770;1	1319;4096;8	1319;4096;8644;9978;116	
180;199;445;	False;False;F	185;204;453;	825;826;827;	618;619;675;	618;676;1496;2951;6433;	
8330	True	8481	38802;38803	30174	30174	
1021;2171;2	True;True;Tr	1041;2216;2	4708;4709;4	3586;7821;9	3586;7821;9343;11161;17	
5101	True	5178	23844;23845	18507;18508	18508	
7309	True	7442	34006;34007	26398	26398	
5887;8254	True;True	5971;8405	27473;27474	21362;29860	21362;29861	
361;1176;15	True;True;Tr	368;1200;15	1596;1597;1	1190;1191;1	1192;4162;5554;9698;111	
7387	True	7521	34354;34355	26649	26649	46
1450;2225;2	True;True;Tr	1479;2270;2	6776;6777;6	5208;5209;8	5208;8007;8898;10245;16	
330;6286	True;True	337;6401	1453;1454;1	1087;1088;1	1088;22729	
46;256;1114;	True;True;Tr	47;261;1138;	192;193;194;	147;148;149;	151;864;3938;22011;2707	
1919;2315;5	True;True;Tr	1963;2360;6	9109;9110;9	7012;8261;8	7012;8262;21700;23821;2	
573;3870;51	True;True;Tr	584;3932;52	2635;2636;2	1939;13967;	1939;13969;18571;26525;	
2325;6222	True;True	2370;6336	10860;10861	8302;22538	8302;22538	
6175	True	6276	28771	22361	22361	47
2055;3767;3	True;True;Tr	2100;3826;4	9713;9714;9	7444;7445;1	7444;13586;14328;30785;	
111;4892;66	True;True;Tr	113;4967;67	478;479;228	360;361;177	360;17783;24277;30402;3	
5441	True	5521	25514;25515	19871;19872	19871	
13;360;405;5	True;True;Tr	13;367;413;5	62;63;64;65;	54;55;56;57;	54;1189;1349;1775;3163;3	
3107;5177;5	True;True;Tr	3160;5254;5	14476;14477	11108;11109	11108;18758;18764	
1198;4400;4	True;True;Tr	1222;4469;4	5551;5552;2	4234;15885;	4234;15885;17767;18048	
511;6744	True;True	522;6873	2355;2356;2	1737;24575;	1737;24577	
97;683;684;6	True;True;Tr	99;700;701;7	417;418;419;	315;316;317;	316;2338;2344;2385;1079	
1856;9670	True;True	1900;9836	8817;8818;8	6790;6791;6	6792;35093	
1117;1907;2	True;True;Tr	1141;1951;2	5162;5163;5	3945;3946;3	3947;6983;8599;8611;155	
4384;5762	True;True	4453;5845	20367;20368	15817;15818	15819;20928	
408;682;111	True;True;Tr	416;699;113	1825;1826;1	1352;1353;1	1353;2337;3903;4078;430	

613;3671;50	True;True;Tr	626;3730;51	2803;2804;2	2071;2072;1	2072;13185;18489;18521;	
333;1406;32	True;True;Tr	340;1435;32	1473;1474;1	1106;1107;5	1107;5071;11450;11825;1	
22;290;619;8	True;True;Tr	23;295;632;8	98;99;100;12	82;971;972;2	82;971;2085;2999;3282;41	
194;900;157	True;True;Tr	199;919;160	890;891;892;	662;663;317	662;3171;57	48
1869;3996;7	True;True;Tr	1913;4059;7	8873;8874;1	6829;6830;1	6830;14400;27352;31331	
5470	True	5550	25631;25632	19951;19952	19951	
1826;2717;2	True;True;Tr	1867;2769;2	8670;8671;1	6672;9619;9	6672;9619;9908;9909;199	
2000;2830;3	True;True;Tr	2045;2882;3	9482;9483;9	7276;7277;7	7276;10018;11195;12371;	
2124;3835;5	True;True;Tr	2169;3896;5	10030;10031	7684;7685;7	7685;13846;18181;22346;	
70;1378;166	True;True;Tr	71;1405;170	296;297;298;	222;223;224;	222;4937;6102;12635;171	
617;1042;57	True;True;Tr	630;1063;57	2818;2819;2	2080;3663;2	2080;3663;20775;28327;3	
98;702;1231;	True;True;Tr	100;719;125	428;429;430;	323;324;325;	324;2419;4357;7326;9163	
912;1834;24	True;True;Tr	931;1877;24	4205;4206;4	3206;3207;3	3208;6724;8634;14332;17	
1723;6502;6	False;False;T	1760;6623;6	8190;30378;	6323;23566;	6323;23568;	2
1951;3196;5	True;True;Tr	1996;3250;5	9258;9259;9	7118;7119;1	7119;11411;18467;19447;	
8259;9903	True;True	8410;10069	38451;46234	29872;35965	29872;35965	
308;2454;27	True;True;Tr	314;2500;27	1358;1359;1	1015;8704;8	1015;8706;9665;14750	
365;426;427;	False;False;F	372;434;435;	1618;1619;1	1209;1210;1	1214;1430;1433;1998;315	
2165;8580	True;True	2210;8731	10195;10196	7805;31068	7805;31068	
305;306;313;	True;True;Tr	311;312;319;	1343;1344;1	1006;1007;1	1008;1012;1028;3056;920	
4757	True	4831	22171;22172	17176	17176	
2217	True	2262	10434	7985	7985	
581;3815;42	True;True;Tr	592;3875;43	2663;2664;2	1960;1961;1	1962;13772;15353;15909;	
323;995;101	False;False;T	329;330;101	1421;1422;1	1059;1060;1	1061;3475;3	49;50;51;52;
1185;6775;1	True;True;Tr	1209;6905;1	5491;5492;5	4191;4192;4	4193;24714;38424	
1908;4214;4	True;True;Tr	1952;4280;4	9065;9066;9	6984;6985;6	6984;15198;16018;20262;	
444;5460;65	True;True;Tr	452;5540;67	2015;2016;2	1493;19929;	1493;19929;23939	
3514	True	3573	16369;16370	12634	12634	
8714	True	8866	40582;40583	31560;31561	31561	
571;2550;60	True;True;Tr	582;2599;61	2630;2631;2	1937;9072;9	1937;9073;22086	
327;585;296	True;True;Tr	334;596;301	1438;1439;1	1076;1077;1	1076;1982;10508;12623;2	
169;3523;51	True;True;Tr	174;3582;51	775;776;777;	587;588;589;	589;12656;18564;30808;3	
4372	True	4441	20319;20320	15786	15786	
295;1159;53	True;True;Tr	301;1183;53	1305;1306;1	981;982;983;	982;4101;19279	
497;1111;40	True;True;Tr	507;1134;41	2287;2288;2	1692;1693;3	1693;3910;14691	
126;643;182	True;True;Tr	128;656;186	557;558;293	423;2186;21	423;2186;6671;9055;9947	
8611;9598	True;True	8762;9762	40097;40098	31167;31168	31168;34817	
5510	True	5590	25827;25828	20115;20116	20116	
6592	True	6718	30811;30812	23950;23951	23950	
5505	True	5585	25798;25799	20090;20091	20091	
904;1136	True;True	923;1160	4178;5282	3185;4035	3185;4035	
5247;9399;1	True;True;Tr	5325;9558;1	24520;24521	19016;19017	19020;34013;37489	
1233;6859;7	True;True;Tr	1258;6989;7	5733;5734;3	4364;24965;	4364;24967;28277	
1646;1742;2	True;True;Tr	1682;1779;2	7804;7805;7	6035;6036;6	6036;6383;9680;10097;10	
2603;3229;3	True;True;Tr	2654;3283;4	12088;12089	9234;9235;9	9234;11545;14305;25505	

763;1601;207	True;True;Tru	780;1631;213	3524;3525;35	2643;2644;26	2644;5804;7503;8481;111
1934;1935;30	False;False;Fa	1978;1979;30	9176;9177;91	7059;7060;70	7059;7067;10781;10792;10
383;1103;140	True;True;Tru	390;1126;149	1703;1704;17	1269;1270;12	1271;3876;5274;11708;11
6501	True	6622	30372;30373	23561;23562	23561
2747	True	2799	12749;12750	9719;9720	9720
597;3199;449	True;True;Fa	608;609;3253	2734;2735;14	2013;2014;11	2013;11418;14;5
1306;1479	True;True	1333;1508	6068;6069;60	4639;5339;53	4639;5340
416;7212;772	True;True;Tru	424;7345;780	1862;1863;18	1376;1377;13	1377;26090;27858
3856;5720	True;True	3918;5802	18000;18001	13920;13921	13920;20816
4563;7030;85	True;False;Tr	4634;7162;87	21243;21244	16491;16492	16491;25556;31005
685;2779;297	True;True;Tru	702;2831;303	3153;3154;31	2348;2349;23	2350;9840;10584;14633;1
8643;9135	True;True	8795;9289	40247;40248	31299;31300	31299;33019
3543;4945;10	True;True;Tru	3602;5020;10	16484;16485	12725;12726	12727;17993;36433
1237	True	1262	5751	4378	4378
315;1016;192	True;True;Tru	321;1036;190	1381;1382;13	1032;3572;35	1032;3573;7015;11430;17
1867;2598;27	True;True;Tru	1911;2649;27	8865;8866;88	6825;9218;92	6825;9223;9540;17731;220
8875	True	9028	41338;41339	32104;32105	32105
8374	True	8525	39012;39013	30327;30328	30332
8074	True	8221	37592;37593	29182	29182
1029;6794	True;True	1049;6924	4745;4746;47	3613;3614;36	3614;24774
7013;9301	True;True	7145;9460	32734;32735	25476;25477	25476;33686
1541;3885	True;True	1570;3947	7245;7246;72	5563;5564;55	5565;14020
1868;2174;30	True;True;Tru	1912;2219;31	8868;8869;88	6826;6827;68	6826;7832;13247
4909	True	4984	22973;22974	17839;17840	17840
5593;5886;65	True;True;Tru	5674;5970;66	26185;26186	20378;21361	20378;21361;23635;30594
10170	True	10338	47470;47471	36847;36848	36848
1424;3535;80	True;True;Tru	1453;3594;81	6667;6668;66	5135;5136;51	5136;12697;31186;32407
5608	True	5689	26264;26265	20434	20434
421;1197;390	True;True;Tru	429;1221;402	1904;1905;19	1408;1409;14	1409;4230;14303;17490;2
8707;10511	True;True	8859;10687	40552;40553	31542;38060	31542;38063
3813;5236;58	True;True;Tru	3873;5314;59	17814;17815	13768;18992	13768;18992;21400
2249;3613;44	True;True;Tru	2294;3672;45	10538;10539	8054;8055;12	8054;12966;16098;21108;
1780;9049;92	True;True;Tru	1819;9202;94	8443;8444;84	6502;6503;32	6502;32714;33521
1077;8320	True;True	1098;8471	4945;4946;49	3754;3755;30	3755;30126
267;1166;290	True;True;Tru	272;1190;295	1204;1205;12	909;910;911;	913;4118;10300;14645;280
1433;2323;29	True;True;Tru	1462;2368;29	6711;6712;67	5164;8292;82	5164;8297;10381;14725;20
714;8147;960	True;True;Tru	731;8297;973	3294;37970;3	2462;29513;2	2462;29515;34844;35045
4088;4322;40	True;True;Tru	4151;4389;47	19058;19059	14765;15616	14765;15616;16949;28125
857;2236;859	True;True;Tru	876;2281;870	3980;3981;39	3041;8024;80	3041;8024;30962
4988	True	5063	23357;23358	18138;18139	18138
3487;6360;74	True;True;Tru	3545;6475;75	16251;16252	12546;12547	12546;23051;26717
430;821	True;True	438;840	1959;1960;19	1449;1450;29	1450;2914
39;252;1739;	True;True;Tru	40;257;1776;	168;169;170;	134;135;839;	134;841;6375;6750;8914;1
8809	True	8962	41050;41051	31901;31902	31902
9263	True	9420	43127;43128	33556	33556

4379;8725	True;True	4448;8877	20346;20347	15806;15807	15806;31601	
3622;5781	True;True	3681;5864	16866;16867	12986;21002	12986;21003	
1184;1713;2	True;True;Tr	1208;1750;2	5488;5489;5	4190;6272;6	4190;6272;8010;14962;22	
60	True	61	253;254;255	193	193	
9196	True	9352	42818;42819	33290	33290	
469;9167;98	True;True;Tr	478;9322;10	2140;2141;2	1585;1586;1	1586;33150;35928	
373;1065;10	False;False;F	380;1086;10	1660;1661;1	1244;1245;1	1247;3720;3729;4532;813	
4638;10199;	True;True;Tr	4709;10371;	21582;21583	16722;16723	16722;36959;38477	
328;403;854	True;True;Tr	335;411;873	1444;1445;1	1080;1081;1	1081;1338;3032;6406;667	
278;303;310	True;True;Tr	283;309;316	1249;1250;1	946;947;948	947;1004;1021;1040	
2191;6346;8	True;True;Tr	2236;6461;8	10332;10333	7918;7919;2	7919;22967;31130;32789;	
28;709;2982	True;True;Tr	29;726;3034	126;127;128	103;104;105	105;2446;10589;28130;33	
8224;9550;9	True;True;Tr	8375;9712;9	38310;38311	29760;34630	29760;34633;34635	
2093	True	2138	9880;9881;9	7570;7571	7571	
2764	True	2816	12835;12836	9788	9788	
861;5226;73	True;True;Tr	880;5304;74	4000;4001;4	3055;18966;	3055;18966;26460	
6607	True	6733	30895;30896	24024	24024	
4208;4217;4	True;True;Tr	4274;4283;4	19600;19601	15185;15186	15187;15206;15345;16894	
1303;3045;3	True;True;Tr	1330;3098;3	6058;6059;6	4633;4634;4	4635;10861;12792;12933;	
8768	True	8921	40892;40893	31787	31787	
1506;2079;8	True;True;Tr	1535;2124;8	7075;7076;7	5444;5445;7	5444;7519;29891;35610	
10068	True	10236	46937;46938	36476;36477	36479	
5686;9665	True;True	5768;9831	26601;26602	20700;35075	20700;35075	
462;1463;30	True;True;Tr	471;1492;30	2114;2115;2	1568;1569;1	1570;5261;10856;11242;1	
698;701;125	True;True;Tr	715;718;127	3224;3225;3	2402;2403;2	2404;2413;4443;4452;514	
1373;1796;5	True;True;Tr	1400;1835;5	6393;6394;6	4913;4914;4	4914;6560;20194;26570;3	
241;4718;49	True;True;Tr	246;4790;50	1088;1089;1	807;17036;1	807;17036;17914;27887	
642;652;395	True;True;Tr	655;665;402	2929;2930;2	2183;2184;2	2183;2212;14262;16715;1	
2132;2722;3	True;True;Tr	2177;2774;3	10059;10060	7704;7705;7	7705;9628;1	54
158;1253;25	True;True;Tr	160;1279;25	695;696;697	523;524;525	523;4445;9037;20377;217	
644;1818;25	True;True;Tr	657;1859;26	2937;2938;2	2189;2190;2	2190;6649;9079;13604;15	
5696;8361	True;True	5778;8512	26647;38940	20735;30277	20735;30278	
5843	True	5927	27304;27305	21227;21228	21228	
2050;2377;3	True;False;Fa	2095;2422;3	9693;9694;9	7424;7425;7	7424;8469;11405;16938;2	
2494;2737;4	True;True;Tr	2543;2789;4	11622;11623	8871;9682;9	8871;9682;16642	
3787;4543	True;True	3846;4613	17681;17682	13663;16419	13663;16420	
523;1533	True;True	534;1562	2405;2406;7	1772;5539;5	1772;5540	
9374	True	9533	43616;43617	33917;33918	33920	
4357;6130;6	True;False;Fa	4426;6222;6	20257;20258	15746;15747	15749;22190	55
551;817;102	True;True;Tr	562;836;104	2538;2539;2	1873;1874;1	1874;2906;3607;4040;625	
1501;10210	True;True	1530;10382	7054;7055;7	5428;5429;5	5429;36983	
8907;10101	True;True	9060;10269	41515;41516	32270;36628	32270;36628	
9092	True	9246	42333;42334	32878;32879	32878	
5617	True	5698	26303;26304	20459	20459	
1361	True	1388	6336;6337;6	4877;4878	4877	

1428;7939	True;True	1457;8086	6682;6683;6(	5145;5146;5:	5145;28674
868;3861;59!	True;True;Tr	887;3923;60	4022;4023;4(	3072;3073;1:	3072;13947;21628
2413	True	2458	11240	8577	8577
95;1017;192(	True;True;Tr	97;1037;196	408;409;410;	310;311;357!	310;3580;7013;11430;16
669;772;135:	True;True;Tr	684;791;138(	3071;3072;3(	2286;2700;2:	2286;2701;4836;8362;865
5182	True	5259	24213	18776	18776
1841;6198;7:	True;True;Tr	1884;6304;7:	8753;8754;8:	6740;6741;2:	6741;22442;26000;35818
346;1007;16:	False;False;F	353;1027;16	1518;1519;1!	1134;1135;1:	1137;3537;5849;6330;633
562;1855;38:	True;True;Tr	573;1899;38	2600;2601;2(	1914;1915;1!	1915;6786;13811;15262;2
1528;1703;4	True;True;Tr	1557;1740;4	7183;7184;7:	5517;5518;6:	5517;6243;15919;30964;3
347;2597;40!	True;True;Tr	354;2648;41!	1525;1526;1!	1139;1140;9:	1140;9216;14772;19875;2
1964;2258;7:	True;True;Tr	2009;2303;7!	9312;9313;9:	7154;7155;7:	7155;8093;26647;27664;3
1317;1887;4:	True;True;Tr	1344;1931;4	6118;6119;6:	4685;4686;4(	4685;6875;15759;28608;2
4993;7295;7!	True;True;Tr	5069;7428;7:	23378;23379	18151;18152	18151;26356;27381;30182
4997;9835	True;True	5073;10001	23390;23391	18159;35745	18159;35745
559;4349;71:	True;True;Tr	570;4418;72	2591;2592;2!	1909;15719;:	1909;15719;25800;37712
3777	True	3836	17622;17623	13615;13616	13617
3060;3643;3!	True;True;Tr	3113;3702;3!	14237;14238	10921;13069	10921;13070;14176;16137
5265;9768	True;False	5344;9934	24616;24617	19090;19091	19091;35527
533;2008;24:	True;True;Tr	544;2053;24:	2452;2453;2	1805;1806;7:	1805;7305;8648;8678;124
175;413;576;	True;True;Tr	180;421;587;	805;806;807;	602;603;604;	603;1364;1945;3064;3953
9036;9321	True;True	9189;9480	42047;42048	32667;32668	32668;33753
216;375;990;	True;True;Tr	221;382;100!	977;978;979;	729;730;125:	729;1252;3444;4010;1210
4125	True	4189	19234;19235	14908;14909	14910
191;1871;24:	True;True;Tr	196;1915;24!	879;880;887	657;6832;68:	657;6832;8565;13396;150
1083;3734;9:	True;True;Tr	1104;3793;9	4982;4983;4!	3791;3792;3:	3791;13416;33506;33507
2578	True	2628	11990;11991	9156	9156
89;7077	True;True	91;7209	379;380;330:	286;287;256!	286;25694
8241	True	8392	38380;38381	29817;29818	29819
677;7740;10:	True;True;Tr	694;7883;10	3117;3118;3:	2321;2322;2:	2322;27917;37073
9759	True	9925	45593;45594	35492;35493	35492
1013;9356	True;True	1033;9515	4673;4674;4(	3563;3564;3!	3566;33873
4610;9365	True;True	4681;9524	21453;21454	16630;16631	16632;33900
2568	True	2618	11956;11957	9128;9129;9:	9128
4756;6804	True;True	4830;6934	22168;22169	17174;17175	17174;24806
7219	True	7352	33606;33607	26105;26106	26107
943;2412;27(	True;True;Tr	962;2457;28:	4346;4347;4:	3318;8570;8!	3318;8571;9786;9787;106
4081	True	4144	19026;19027	14740;14741	14741
171;8873	True;True	176;9026	787;788;789;	592;593;320!	593;32100
3354	True	3410	15613;15614	11989;11990	11991
472;1151;61:	True;True;Tr	481;1175;62:	2163;2164;2:	1600;1601;1(	1600;4087;22356;25507;3
5218;7877;8:	True;True;Tr	5295;8023;8:	24394;24395	18929;18930	18931;28395;29624;34529
2951	True	3003	13681;13682	10482	10482
496;1319;16:	True;True;Tr	506;1346;17:	2284;2285;2:	1691;4690;4(	1691;4691;6140;7837;125
620;3584;36(	True;True;Tr	633;3643;37:	2830;2831;2	2086;2087;2(	2088;12874;13156;25138;

835;2451;41	False;False;F	854;2497;42	3886;3887;3	2976;2977;8	2977;8697;15051;17741;1	
4880;5083;7	False;True;Tr	4955;5160;7	22836;22837	17740;17741	17741;18451;25832	
2304;2305;5	True;True;Tr	2349;2350;5	10766;10767	8221;8222;8	8223;8229;19000	
195;831;181	True;False;Fa	200;850;185	893;894;895;	664;665;295	664;2951;66	56
2274;2451;4	True;False;Fa	2319;2497;4	10642;11400	8132;8696;8	8132;8697;1	57
199;828;175	False;False;F	204;847;179	908;909;910;	675;676;293	676;2933;6433;9117;9127	
743;4126;41	True;True;Tr	760;4190;41	3424;3425;3	2563;2564;2	2563;14923;	58
166;741;883;	True;True;Tr	168;169;758;	738;739;740;	561;562;563;	565;2559;31	59;60;61;62;
253;541;414	True;False;Fa	258;552;420	1133;2486;2	842;1830;18	842;1833;14	62
1937;2022;2	True;True;Tr	1981;2067;2	9194;9195;9	7071;7342;8	7071;7342;8	65
598;662;442	True;True;Tr	610;677;449	2736;2737;2	2015;2255;1	2015;2255;15979;22687;2	
1038;1630;2	True;True;Tr	1059;1665;1	4792;4793;4	3650;3651;5	3650;5937;9	66
6831;6947;7	True;True;Tr	6961;7078;7	31959;32460	24874;25280	24874;25280	67
5819;8993;9	True;True;Tr	5903;9146;9	27175;27176	21117;21118	21117;32535;33095;3309	
220;221;269;	True;True;Tr	225;226;274;	991;992;993;	735;736;737;	738;743;920;	68;69;70
3667	True	3726	17083;17084	13176	13176	
180;199;831;	True;True;Fa	185;204;850;	825;826;827;	618;619;675;	618;676;2951;3338;6433;	
828;881;176	False;True;Tr	847;900;179	3841;3842;3	2933;2934;2	2933;3119;6438;9117;912	
748;979;114	True;True;Tr	765;998;116	3442;3443;3	2582;2583;2	2585;3419;4	71;72;73
740;883;884;	True;False;Fa	757;902;903;	3411;3412;3	2553;2554;2	2554;3125;3	62;74;75;76;
167;168;254;	True;True;Tr	170;171;172;	748;749;750;	567;568;569;	567;581;849;	62;78;79;80;
830;1758;25	True;False;Fa	849;1795;26	3855;3856;3	2945;2946;2	2945;6433;9	82;83
982	True	1001	4501;4502;4	3426;3427	3427	
3582	True	3641	16682;16683	12865;12866	12865	
1488;2082;2	True;True;Tr	1517;2127;2	6976;6977;6	5367;5368;5	5367;7529;1	84;85;86;87;
831;1766;18	False;False;F	850;1804;18	3861;3862;3	2951;2952;2	2951;6457;6	91;92;93
200;498;920;	False;True;Tr	205;508;939;	914;2290;22	677;1694;16	677;1695;32	32;94
538;882;963;	True;True;Tr	549;901;982;	2471;2472;2	1819;1820;3	1819;3123;3	61
835;1634;24	True;True;Tr	854;1670;24	3886;3887;3	2976;2977;5	2977;5955;8697;15051;17	
258;553;143	True;True;Tr	263;564;146	1161;1162;1	871;1884;18	871;1890;5170;8460;1081	
10350	True	10523	48273;48274	37442;37443	37444	
281	True	286	1258;1259;1	951;952;953;	954	
195;1242;25	False;True;Fa	200;1267;26	893;894;895;	664;665;439	664;4392;9117;9127;9637	
196;2498;24	True;True;Tr	201;2547;25	896;897;898;	666;667;888	666;8882;8886;9117;9127	
2631	True	2682	12192;12193	9309;9310	9310	
165;835;127	True;False;Fa	167;854;130	737;3886;38	560;2976;29	560;2977;4534;4569;1504	
2284;2824;3	True;True;Tr	2329;2876;3	10697;10698	8174;8175;8	8175;9988;13509;26243;2	
196;1814;25	False;False;F	201;1854;18	896;897;898;	666;667;663	666;6639;91	92
828;1758;25	True;False;Fa	847;1795;26	3841;3842;3	2933;2934;2	2933;6433;9	82;83
200;498;827;	False;False;T	205;508;846;	914;2290;22	677;1694;16	677;1695;29	32;94;95
2451;4982;7	False;False;F	2497;5057;7	11400;23336	8696;8697;1	8697;18120;25931;29022	
7578;8047;8	True;True;Tr	7716;8194;8	35237;35238	27371;27372	27374;29085;30167	
200;919;111	True;True;Tr	205;938;114	914;4238;42	677;3231;32	677;3232;39	32;96
195;958;272	False;True;Fa	200;977;277	893;894;895;	664;665;335	664;3357;9632;19859;294	
262;1342;10	True;False;Fa	267;1369;10	1183;6227;6	890;4772;47	890;4779;36	62

2565;2567;2	False;False;F	2615;2617;2	11940;11941	9114;9115;9	9117;9127;9643;25710;26	
3012;3382;3	True;True;Tr	3065;3438;3	13976;13977	10721;10722	10721;12107;12113;1307	
1313;1314;7	True;True;Tr	1340;1341;7	6103;6104;6	4667;4668;4	4668;4670;27121	
498;827;252	False;False;F	508;846;257	2290;2291;2	1694;1695;2	1695;2931;8	32;94
257;2620;28	True;True;Tr	262;2671;28	1158;1159;1	868;869;870;	868;9278;10088;10688;11	
3262	True	3316	15193	11679	11679	
835;2451;41	False;False;F	854;2497;42	3886;3887;3	2976;2977;8	2977;8697;1	57
184;1411;38	True;True;Tr	189;1440;39	843;844;845;	629;630;631;	630;5085;13880;14355;16	
1878;1898;3	True;True;Tr	1922;1942;3	8900;8901;8	6853;6854;6	6854;6906;13780;20367;2	
5726;5870;7	True;True;Tr	5808;5954;8	26776;26777	20827;21305	20827;21306;28529	
6264	True	6379	29185;29186	22665;22666	22666	
2194;3785;4	True;True;Tr	2239;3844;4	10345;10346	7924;13654;	7924;13654;15595;20292;	
1994	True	2039	9462;9463;9	7266;7267;7	7266	
1478;3549;3	True;True;Tr	1507;3608;3	6927;6928;6	5329;5330;5	5331;12745;13367	
530;1133;16	True;True;Tr	541;1157;16	2438;2439;2	1794;1795;1	1797;4016;5885;9882;330	
6921;7318	True;True	7051;7451	32327;32328	25150;25151	25151;26429	
2484;9085;9	True;True;Tr	2533;9239;1	11584;11585	8848;8849;8	8848;32861;36066	
406;7223;77	True;True;Tr	414;7356;78	1819;1820;1	1350;26120;	1350;26120;27931;38566	
1729;8893	True;True	1766;9046	8221;8222;8	6348;6349;3	6349;32179	
4449;9546	True;True	4518;9708	20678;44509	16058;34617	16058;34620	
418;1080;53	True;True;Tr	426;1101;54	1874;1875;1	1384;1385;3	1385;3786;19564;20520;2	
2122;3455;5	True;True;Tr	2167;3513;5	10019;10020	7676;7677;7	7677;12416;18985;30782	
1936;4574;8	True;True;Tr	1980;4645;8	9188;9189;9	7069;7070;1	7070;16514;29424;36006	
1093;3654;4	True;True;Tr	1116;3713;4	5047;5048;5	3841;3842;3	3841;13128;14781;15075;	
495;564;100	True;True;Tr	505;575;102	2279;2280;2	1689;1690;1	1689;1924;3521;6901;722	
71;1194;167	True;True;Tr	72;1218;170	302;303;304;	228;229;230;	232;4220;6111;11817;194	
587	True	598	2693;2694;2	1986;1987;1	1986	
2294;5753;6	True;True;Tr	2339;5835;6	10728;10729	8194;8195;2	8195;20895;22759;22766;	
6033	True	6119	28157	21895	21895	97
986;2193	True;True	1005;2238	4521;4522;4	3440;7921;7	3440;7921	
2845;6181;7	True;True;Tr	2897;6284;8	13200;13201	10103;22386	10103;22386;28620	
526;3771;50	True;False;Tr	537;3830;51	2426;17596;	1783;13599;	1783;13600;18297;25462;	
2397;2669;2	True;True;Tr	2442;2720;2	11166;11167	8517;9441;9	8517;9441;9673;9831;220	
211;381;116	False;False;F	216;388;118	953;954;955;	712;713;714;	716;1263;41	98
2874;3126;3	True;True;Tr	2926;3180;3	13314;13315	10187;10188	10188;11168;11529;19241	
6774;9350;9	True;True;Tr	6904;9509;9	31737;31738	24710;24711	24712;33848;35346	
7063;8679	True;True	7195;8831	32957;32958	25643;25644	25644;31432	
595;913;983;	True;True;Tr	606;932;100	2727;2728;4	2009;3209;3	2009;3209;3430;20236;20	
3260;5016;7	True;True;Tr	3314;5092;7	15188;15189	11677;18228	11677;18228;26421;2734	
6042	True	6128	28192;28193	21921	21921	
1756;3391;8	True;True;Tr	1793;3447;9	8332;8333;8	6427;6428;6	6429;12152;32069;34883	
2080	True	2125	9818;9819;9	7520;7521;7	7522	
4244;6052;6	True;True;Tr	4311;6138;6	19758;19759	15309;15310	15313;21943;24064;2406	
1999;7804	True;True	2044;7948	9479;9480;9	7275;28131;	7275;28131	
2882	True	2934	13366;13367	10234;10235	10234	

481;565;383	True;True;Tr	490;576;389	2206;2207;2	1643;1644;1	1643;1928;13854;22723;2	
480;565;383	True;False;Fa	489;576;389	2203;2204;2	1640;1641;1	1642;1928;13854;22723;3	
6905;9540	False;True	7035;9702	32256;32257	25095;25096	25095;34592	
1215;2302;4	True;True;Tr	1239;2347;4	5638;5639;5	4294;4295;4	4294;8217;15563;18705;1	
439;2861;49	True;True;Tr	447;2913;50	1999;2000;2	1480;1481;1	1481;10153;18134;18529;	
10141	True	10309	47338;47339	36763;36764	36765	
7796;9106	True;True	7940;9260	36219;36220	28118;32921	28118;32922	
173;1918;28	True;True;Tr	178;1962;28	796;797;798;	595;596;597;	596;7011;10108;11826;12	
21;5722;649	True;True;Tr	22;5804;661	95;96;97;267	81;20818;23	81;20818;23545;24327;29	
514;2176;70	True;True;Tr	525;2221;71	2364;2365;2	1742;7835;7	1742;7836;25569;33135	
1149	True	1173	5338;5339;5	4082	4082	
323;996;101	False;True;Fa	329;330;101	1421;1422;1	1059;1060;1	1061;3482;3	49;52;53;99
876	True	895	4062;4063;4	3106;3107;3	3111	
84;5423;987	True;True;Tr	86;5503;100	363;364;254	274;275;198	275;19816;35880	
2999;4885	True;True	3051;4960	13916;13917	10673;10674	10673;17761	
4348	True	4417	20225	15718	15718	
1519;2128;2	True;True;Tr	1548;2173;2	7133;7134;7	5483;7698;1	5483;7698;10168;10169;1	
475;2416;24	True;False;Fa	484;2461;24	2183;2184;2	1619;1620;1	1621;8590;8	100
9709	True	9875	45281;45282	35223;35224	35224	
1529;1750;3	True;True;Tr	1558;1787;3	7186;7187;8	5519;6403;6	5519;6405;12978;21449;2	
11;984;4002;	True;True;Tr	11;1003;406	56;57;58;450	50;51;52;343	51;3431;14411;17812;221	
5528	True	5608	25907;25908	20176	20176	
2096;2493;2	True;True;Tr	2141;2542;2	9893;9894;9	7579;7580;7	7580;8870;9334;13957;20	
1224;5712;8	True;True;Tr	1248;5794;8	5681;5682;5	4324;4325;2	4325;20792;30414;32114;	
10116	True	10284	47211;47212	36673;36674	36674	
6343	True	6458	29575;29576	22961;22962	22963	
1240;1569;2	True;True;Tr	1265;1599;2	5758;5759;5	4386;5680;5	4386;5681;7789;8257;116	
945;6400;71	True;True;Tr	964;6521;72	4352;4353;4	3321;3322;2	3321;23200;25868;30395	
614;3679;60	False;False;F	627;3738;61	2806;2807;2	2073;2074;2	2074;13220;22084;24366;	
4228;10004;	True;True;Tr	4294;10171;	19694;19695	15249;36278	15249;36280;38557	
942;1599;20	True;True;Tr	961;1629;20	4335;4336;4	3308;3309;3	3311;5785;7	101;102;103;
1723;1820;6	False;True;Fa	1760;1861;6	8190;8645;8	6323;6653;2	6323;6653;2	2
764;3798;96	True;True;Tr	781;3857;98	3530;3531;3	2648;2649;2	2648;13712;34965	
225;960;136	True;True;Tr	230;979;139	1017;1018;1	753;754;755;	755;3360;4882;11722;159	
2883;6492;7	True;True;Tr	2935;6613;7	13372;13373	10239;10240	10240;23542;26644;3451	
2366;2833;4	True;True;Tr	2411;2885;4	11049;11050	8439;10059;	8439;10061;16370;20472;	
1872	True	1916	8881;8882	6834;6835	6835	
3373;4104;4	True;True;Tr	3429;4167;4	15690;19116	12072;14807	12072;14809;17370	
321;946;119	True;True;Tr	327;965;121	1409;1410;1	1050;1051;1	1051;3323;4209;10393;10	
781;830;175	True;False;Fa	800;849;179	3641;3855;3	2769;2945;2	2769;2945;6	82;83
3475	True	3533	16198;16199	12502;12503	12502	
961;3542;48	True;True;Tr	980;3601;49	4411;4412;4	3361;3362;1	3362;12724;	106
4854;10355	True;True	4929;10528	22671;22672	17604;17605	17605;37462	
1129;2059;4	True;True;Tr	1153;2104;4	5246;5247;5	4008;4009;7	4009;7453;16537;16714;1	
719;727;147	False;True;Tr	736;744;149	3323;3324;3	2486;2487;2	2489;2524;5289;5952;657	

7108	True	7241	33163;33164	25786;25787	25786	
3846	True	3907	17950;17951	13878;13879	13878	
4012;5750	True;True	4075;5832	18702;18703	14456;20890	14456;20890	
2410;4373;5	False;False;T	2455;4442;5	11226;11227	8565;8566;1	8565;15788;21013;30404	
387;1272;22	True;True;Tr	394;1299;23	1727;1728;1	1291;4525;8	1291;4525;8181;10746;11	
197;198;829;	True;True;Tr	202;203;848;	899;900;901;	668;669;670;	668;674;294	56;107;108;1
515;3007	True;True	526;3059	2367;2368;2	1743;1744;1	1745;10695	
209;1220;18	True;True;Tr	214;1244;19	947;948;949;	708;4311;68	708;4311;6808;7834;8313	
2943;3571;4	True;True;Tr	2995;3630;4	13635;13636	10448;10449	10450;12830;16651;17092	
3349;7178;7	True;False;Tr	3405;7311;7	15582;15583	11968;11969	11970;25979;26725;3550	
94;362;398;7	False;False;F	96;369;406;8	405;406;407;	309;1193;11	309;1193;1320;2785;3228	
1001;2423;3	True;True;Tr	1021;2469;3	4605;4606;4	3505;3506;3	3507;8620;10962;11715;1	
2401	True	2446	11181;11182	8526;8527	8527	
182;888;215	True;True;Tr	187;907;220	831;832;833;	621;622;623;	623;3134;7781;10138	
1175;2790;7	False;False;T	1199;2842;7	5441;5442;5	4150;4151;4	4154;9891;25979;35503;3	
707;1331;17	True;True;Tr	724;1358;18	3269;3270;3	2440;2441;2	2440;4730;6541;7351;970	
2125;5081;6	True;True;Tr	2170;5158;6	10036;10037	7689;7690;7	7689;18445;22316;35211;	
10516	True	10692	49127;49128	38083;38084	38083	
105;1064;19	True;True;Tr	107;1085;19	464;4894;48	351;3717;37	351;3717;7120	
3106	True	3159	14470;14471	11104;11105	11106	
5498	True	5578	25760;25761	20059;20060	20061	
433;592;351	True;True;Tr	441;603;357	1967;1968;1	1453;1454;1	1454;2003;12644;24839	
5401	True	5480	25340;25341	19731;19732	19731	
1141;1794;3	True;False;Tr	1165;1833;3	5292;5293;5	4043;4044;4	4043;6553;12943;21158;2	
139;482;250	True;True;Tr	141;491;255	622;623;624;	475;476;164	475;1645;8912;13946;151	
5413	True	5493	25397;25398	19777;19778	19777	
3734;8621	False;True	3793;8772	17388;17389	13414;13415	13416;31207	
1707;3231;3	True;True;Tr	1744;3285;3	8097;8098;8	6252;11549;	6252;11551;11555;11798;	
6689	True	6817	31313;31314	24361;24362	24363	
1427;9733	True;True	1456;9899	6679;6680;6	5142;5143;5	5142;35322	
9109	True	9263	42409	32929	32929	111
7;19;1480;21	True;True;Tr	7;19;1509;21	41;42;43;44;	36;37;38;39;	37;72;5342;7674;18683;21	
719;1471;17	True;True;Tr	736;1500;18	3323;3324;3	2486;2487;2	2489;5296;6562;6859;776	
3724;4200;1	True;True;Tr	3783;4266;1	17333;17334	13382;13383	13382;15164;37190	
1465;1472;1	True;True;Tr	1494;1501;1	6854;6855;6	5268;5269;5	5271;5300;5363;6798;680	
5185;5397;6	True;True;Tr	5262;5476;6	24220;25326	18782;19723	18782;19723;24217;3715	
1222;1832	True;True	1246;1875	5669;5670;5	4318;6718	4318;6718	
1771;4025;7	True;True;Tr	1810;4088;7	8409;8410;8	6475;6476;1	6476;14520;27161	
1410;2442;4	True;True;Tr	1439;2488;4	6604;6605;6	5082;5083;8	5082;8671;15152;19298;1	
1286;1794;3	True;True;Tr	1313;1833;3	5969;5970;5	4563;4564;4	4567;6553;10984;13795;2	
356;973;201	True;True;Tr	363;992;205	1572;1573;1	1177;3393;3	1177;3394;7316;15699;16	
1058;2452	True;True	1079;2498	4869;4870;4	3701;8698;8	3701;8698	
887;1487;23	True;True;Tr	906;1516;23	4098;4099;4	3132;3133;5	3132;5365;8389;8392;876	
2682;2706;5	True;True;Tr	2734;2758;5	12437;12438	9483;9577;9	9483;9584;18745;23238;3	
637;728;179	True;True;Tr	650;745;183	2906;2907;2	2165;2166;2	2166;2525;6546;36986	

10138	True	10306	47320;47321	36747;36748	36749	
3969	True	4032	18501;18502	14312	14312	
5493;5560	True;True	5573;5641	25736;25737	20040;20041	20042;20287	
1061;1879;8	True;True;Tr	1082;1923;8	4882;4883;4	3711;3712;6	3712;6856;31086	
1699	True	1736	8068;8069;8	6229;6230;6	6231	
172;1669	True;True	177;1705	793;794;795;	594;6107;61	594;6107	
8592;9191	True;True	8743;9347	40013;40014	31099;31100	31100;33269	
30;814;1143;	True;True;Tr	31;833;1167;	132;133;134;	109;110;111;	111;2886;4053;4062;6185	
6414;7734	True;True	6535;7877	29950;29951	23254;27893	23254;27895	
1034;3317	True;True	1054;3372	4768;4769;4	3628;3629;3	3629;11850	
5190	True	5267	24245;24246	18805;18806	18806	
10076	True	10244	46988;46989	36518	36518	
2760;3175;3	True;True;Tr	2812;3229;3	12823;12824	9780;9781;1	9781;11340;11880;11884;	
2684;3043;3	True;True;Tr	2736;3096;4	12442;12443	9485;9486;9	9485;10855;14322;15902;	
493;1232;18	True;True;Tr	503;1257;18	2270;2271;2	1683;1684;1	1683;4362;6	112
2902	True	2954	13447;13448	10295	10295	
2170;3071;3	True;True;Tr	2215;3124;3	10217;14286	7820;10956;	7820;10957;12481;15182;	
4557;5708	True;True	4628;5790	21214;21215	16469;20781	16469;20781	
593;1873;19	True;True;Tr	604;1917;19	2719;2720;2	2005;2006;2	2006;6836;7076;12071;15	
923;1499;24	True;True;Tr	942;1528;25	4257;4258;4	3247;5411;5	3247;5416;8814;13876;21	
1012;2450;4	True;True;Tr	1032;2496;4	4670;4671;4	3560;3561;3	3562;8693;16115;20252;2	
621;1950;28	True;True;Tr	634;1995;28	2837;2838;2	2092;2093;2	2093;7117;10019;30441;3	
1164;9945;1	True;True;Tr	1188;10112;	5389;5390;5	4113;36092;	4113;36092;37083	
106;282;437;	True;True;Tr	108;287;445;	465;466;126	352;956;147	352;956;1472;1578;1897;3	
2689	True	2741	12469;12470	9507;9508;9	9508	
793;3586;45	True;True;Tr	812;3645;45	3680;3681;3	2798;2799;2	2803;12878;16320;18680;	
393;4211	True;True	401;4277	1750;1751;1	1306;15192;	1306;15192	
148;1605;18	True;True;Tr	150;1635;18	655;656;657;	497;498;581	497;5813;6656;10394;115	
380;1622;17	True;True;Tr	387;1657;18	1688;1689;1	1261;5916;5	1261;5917;6573;7265;751	
892;8698	True;True	911;8850	4115;4116;4	3141;31503;	3141;31503	
3374;3801;5	True;True;Tr	3430;3860;5	15691;15692	12073;12074	12074;13720;21009;35402	
2828;5351;5	True;True;Tr	2880;5430;5	13091;13092	10002;10003	10007;19409;20503;2229	
676;844;242	True;True;Tr	693;863;247	3114;3115;3	2319;2320;2	2320;2996;8631;9440;112	
786;2500;44	True;True;Tr	805;2549;45	3659;3660;3	2783;8887;8	2783;8889;16105;29570;3	
4788	True	4862	22323;22324	17331	17331	
380;435;156	False;True;Tr	387;443;159	1688;1689;1	1261;1463;1	1261;1464;5678;6193;726	
145;367;554;	True;True;Tr	147;374;565;	640;641;642;	486;487;488;	490;1220;1893;2545;8894	
886;1575;51	True;True;Tr	905;1605;52	4097;7426;7	3131;5706;5	3131;5706;18651;26731;3	
411;2718;33	True;True;Tr	419;2770;34	1838;1839;1	1359;1360;1	1360;9620;12132;18291;2	
3618;6778;7	True;True;Tr	3677;6908;7	16850;16851	12975;24724	12975;24724;25960;3809	
5804;6862;7	True;True;Tr	5888;6992;8	27106;27107	21064;21065	21071;24971;28724	
103;2001;35	True;True;Tr	105;2046;35	455;456;457;	345;7279;72	345;7279;12694;28655	
544;2878;49	True;True;Tr	555;2930;49	2500;2501;2	1843;1844;1	1846;10223;17885;21145;	
796;3717;79	True;True;Tr	815;3776;81	3692;3693;3	2810;2811;2	2812;13363;28775	
1414;1489;2	True;True;Tr	1443;1518;2	6621;6622;6	5093;5094;5	5100;5375;10071;10944;1	

3035;7008;1	True;True;Tr	3088;7140;1	14103;14104	10816;10817	10817;25466;37820;38147	
1746;2995;3	True;True;Tr	1783;3047;3	8287;8288;8	6395;6396;1	6396;10656;12355;14021;	
59;6385;999	True;True;Tr	60;6501;101	248;249;250;	188;189;190;	189;23130;36261	
73;700;1403;	True;True;Tr	74;717;1432;	312;313;314;	234;2408;24	234;2411;5059;5924;6380	
170;542;224	True;True;Tr	175;553;229	784;785;786;	591;1836;18	591;1837;8049;8489;9538	
791;1590;16	True;True;Tr	810;1620;16	3676;7488;7	2796;5755;5	2796;5757;5808;14960;28	
2934;7435	True;True	2986;7569	13584;13585	10402;26895	10402;26895	
6012	True	6098	28082;28083	21830;21831	21830	
3484;6643	True;True	3542;6770	16242;16243	12540;24195	12540;24196	
110;259;136	True;True;Tr	112;264;139	475;476;477;	359;872;873;	359;872;4884;8396;8520;	
1106;4448;5	True;True;Tr	1129;4517;5	5106;5107;2	3892;16057;	3892;16057;19774;21051;	
3161;6165;6	True;True;Tr	3215;6266;6	14702;14703	11292;22331	11292;22331	113;114;115
235;1400;15	True;True;Tr	240;1428;16	1054;1055;1	779;780;781;	779;5038;5776;8528;1421	
14;298	True;True	14;304	67;68;69;131	58;987	58;987	
520;1710;24	True;True;Tr	531;1747;25	2386;2387;2	1758;1759;6	1758;6267;8777;11713;12	
144;2057;24	True;True;Tr	146;2102;24	637;638;639;	484;485;744	485;7448;8612;10327;117	
7282	True	7415	33890;33891	26316	26316	116;117
8912	True	9065	41529;41530	32281;32282	32282	
7608	True	7747	35361;35362	27464;27465	27465	
1906;3155;7	True;True;Tr	1950;3209;7	9057;9058;9	6977;6978;1	6977;11271;28170	
10049	True	10216	46849;46850	36411;36412	36413	
2260;4353;5	True;True;Tr	2305;4422;5	10594;10595	8097;8098;1	8098;15737;20386;27505	
2046;2291;6	True;True;Tr	2091;2336;7	9685;9686;9	7420;8184;8	7420;8184;25362;27797;2	
795;2559;37	True;True;Tr	814;2608;38	3689;3690;3	2807;2808;2	2808;9097;13591;15517;1	
7963;8623	True;True	8110;8774	37037;37038	28759;31219	28759;31219	
123;124;386;	True;True;Tr	125;126;393;	534;535;536;	401;402;403;	404;409;128	118;119
1120;1221;7	True;True;Tr	1144;1245;7	5177;5178;5	3954;4312;4	3954;4313;27610;38053	
631;1035;21	False;False;F	644;1055;10	2877;2878;2	2121;2122;2	2129;3633;7	120;121;122;
2936;4121;5	True;True;Tr	2988;4185;5	13592;13593	10408;10409	10408;14891;19376;2147	
784;3605;49	True;True;Tr	803;3664;50	3650;3651;3	2775;2776;1	2775;12949;18128;18585;	
628;972;175	True;True;Tr	641;991;179	2864;2865;2	2111;2112;2	2112;3391;6410;6519;106	
1008;3517;5	True;True;Tr	1028;3576;5	4646;4647;4	3538;3539;1	3539;12642;20444;21204;	
5456	True	5536	25588;25589	19923	19923	
125;1417;23	True;True;Tr	127;1446;23	552;553;554;	418;419;420;	419;5112;8299;10498;116	
429;2850;42	True;True;Tr	437;2902;42	1953;1954;1	1445;1446;1	1446;10118;15265;15294;	
159;160;140	True;True;Tr	161;162;142	701;702;703;	529;530;531;	531;536;503	128;129;130
457;634;246	True;True;Fa	465;466;647;	2066;2067;2	1524;1525;1	1530;2146;8	121;122;123;
2363;4240;4	True;True;Tr	2408;4307;4	11041;11042	8435;15295;	8435;15295;16073;23984;	
7924	True	8071	36854;36855	28614;28615	28614	
1831;2539;3	True;True;Tr	1874;2588;3	8710;8711;8	6715;6716;6	6717;9039;12640;16536;2	
1459;1640;1	True;True;Tr	1488;1676;1	6819;6820;6	5242;5243;5	5242;6011;7074;8109;904	
130;1444;17	True;True;Tr	132;1473;17	570;571;572;	435;436;437;	439;5196;6284;9756;2044	
1677;3130;3	True;True;Tr	1714;3184;3	7947;7948;7	6133;6134;6	6136;11190;11193;11728;	
370;956;370	False;True;Fa	377;975;376	1648;1649;1	1233;1234;1	1235;3348;13280;13281	
4930;5010	True;True	5005;5086	23077;23078	17923;17924	17925;18208	

622	True	635	2840;2841;28	2095	2095	
492;625;135	True;True;Tr	501;502;638;	2255;2256;2	1670;1671;1	1676;2100;4	134
834;1250;13	True;True;Tr	853;1276;13	3878;3879;3	2968;2969;2	2969;4435;4	135;136;137;
344;1074;15	True;True;Tr	351;1095;15	1514;1515;1	1131;1132;3	1132;3746;5544;5588;604	
5621;5806	True;True	5702;5890	26324;26325	20473;20474	20473;21075	
1416;2324;2	True;False;Tr	1445;2369;2	6632;6633;6	5104;8298;8	5104;8299;10347;10498;1	
2908;9414	True;True	2960;9574	13463;43811	10304;34075	10304;34077	
1165;2457;5	True;True;Tr	1189;2503;5	5392;5393;5	4114;4115;4	4115;8724;18664;32658;3	
837;1089;12	True;True;Tr	856;1112;12	3890;3891;3	2979;2980;2	2981;3825;4389;7345;871	
474;889;283	True;True;Tr	483;908;288	2171;2172;2	1607;1608;1	1612;3136;1	139
233;250;277;	True;True;Tr	238;255;282;	1048;1049;1	773;774;775;	773;832;945;	140;141;142
505;506;954;	True;True;Tr	516;517;973;	2326;2327;2	1719;1720;1	1720;1721;3341;8422;125	
504;873;349	True;True;Tr	515;892;355	2315;2316;2	1708;1709;1	1716;3092;12566;16276;1	
484;1997;65	True;True;Tr	493;2042;66	2218;2219;2	1650;1651;1	1650;7273;23701;29446;3	
3358;5487	True;True	3414;5567	15628;15629	12002;20009	12002;20010	
8337;9475;9	True;True;Tr	8488;9637;1	38829;38830	30189;30190	30189;34342;36034	
733	True	750	3385;3386;3	2535;2536	2536	
1302;2283;2	True;True;Tr	1329;2328;2	6052;6053;6	4629;4630;4	4631;8170;10181;11409;1	
2330;2569;4	True;True;Tr	2375;2619;4	10884;10885	8320;8321;9	8320;9133;14623;19928;2	
440;441;140	True;True;Tr	448;449;143	2002;2003;2	1483;1484;5	1483;1484;5064;5069;141	
94;275;362;3	True;True;Tr	96;280;369;4	405;406;407;	309;936;119	309;936;1193;1320;2785;3	
2346;3715;1	True;True;Tr	2391;3774;1	10955;10956	8371;8372;8	8373;13359;37194	
228;357;650;	True;True;Tr	233;364;663;	1031;1032;1	762;763;117	763;1178;2203;2208;3257	
274;385;395;	True;True;Tr	279;392;403;	1233;1234;1	931;932;933;	934;1284;1313;1425;2372	
1187;5691	True;True	1211;5773	5500;5501;2	4198;20713	4198;20713	
674;675;147	True;True;Tr	691;692;150	3102;3103;3	2308;2309;2	2309;2314;5309;5714;114	
3261;5110;7	True;True;Tr	3315;5187;7	15191;15192	11678;18535	11678;18535;27338;3138	
510;1299;25	True;True;Tr	521;1326;26	2353;2354;6	1736;4622;9	1736;4622;9	143;144
1028;1852;7	True;True;Tr	1048;1896;7	4739;4740;4	3608;3609;3	3608;6778;26336;35625	
1744;3837;9	True;True;Tr	1781;3898;9	8278;8279;1	6389;13848;	6389;13848;35631;35823	
234;1399;21	True;True;Tr	239;1427;21	1051;1052;1	776;777;778;	777;5032;7750;8056;9373	
875;1025;36	True;True;Tr	894;1045;36	4055;4056;4	3101;3102;3	3103;3595;12955;16453;1	
1777;4865;5	True;True;Tr	1816;4940;6	8434;8435;8	6495;6496;1	6495;17670;21681;24954;	
712;1002;21	True;True;Tr	729;1022;22	3285;3286;3	2455;3511;3	2455;3511;7934;12875;15	
670;2933;39	True;True;Tr	685;686;298	3074;3075;3	2287;2288;2	2289;10400;	145
532	True	543	2449;2450;2	1803;1804	1803	
1809;2034;2	True;True;Tr	1848;2079;2	8576;8577;8	6605;6606;6	6610;7373;7	146
499;2067;20	True;True;Tr	509;510;211	2295;2296;2	1696;1697;7	1696;7474;7	147
746;1152;33	True;True;Tr	763;1176;33	3436;3437;3	2576;2577;2	2578;4089;11845;16001;1	
447;1447;32	True;True;Tr	455;1476;33	2027;2028;2	1499;5202;5	1499;5204;11792;12858;1	
2233	True	2278	10486;10487	8021	8021	
286;384;547;	True;True;Tr	291;391;558;	1275;1276;1	963;964;965;	965;1278;1853;4330;5597	
1311;2090;3	True;True;Tr	1338;2135;3	6091;6092;6	4659;4660;4	4660;7564;13470;13633;1	
3492;3493	True;True	3551;3552	16276;16277	12568;12569	12568;12569	
1589;8243;9	True;True;Tr	1619;8394;9	7485;7486;7	5754;29823;	5754;29824;33825	

1324;2262;2	True;True;Tr	1351;2307;2	6149;6150;6	4710;4711;4	4710;8101;9610;10489;12
1723;6502;7	True;True;Tr	1760;6623;7	8190;30378;	6323;23566;	6323;23568;	2;148
1046;2971;7	True;True;Tr	1067;3023;7	4821;4822;4	3668;3669;3	3669;10543;26987;27542
1617;2955;3	True;True;Tr	1651;3007;3	7655;7656;7	5891;5892;1	5891;10494;13759;18488;
1259;1437;1	True;True;Tr	1285;1286;1	5842;5843;5	4459;4460;4	4464;5173;5	149;150
583;715;255	True;True;Tr	594;732;260	2678;2679;2	1970;1971;1	1970;2463;9083;9085;147
36;477;1730;	True;True;Tr	37;486;1767;	151;152;153;	123;124;125;	124;1635;6350;6815;8053
2306;4042;4	True;True;Tr	2351;4105;4	10776;10777	8230;8231;8	8231;14578;	151;152
831;1766;18	True;True;Tr	850;1804;18	3861;3862;3	2951;2952;2	2951;6457;6	91;92;93
2198;3691;4	True;True;Tr	2243;3750;4	10360;10361	7933;13242;	7933;13242;16109;22675;
3;127;128;72	True;True;Tr	3;129;130;73	20;21;22;23;	17;18;19;20;	20;426;431;2496;2503;690
1;2489;2786;	True;True;Tr	1;2538;2838;	6;7;8;9;10;11	5;6;7;8;9;10;	8;8861;9876;15822;16698
449;1673;17	True;True;Tr	457;1710;17	2033;2034;2	1502;1503;1	1502;6117;6326;6576;660
299;932;933;	True;True;Tr	305;951;952;	1316;1317;1	988;989;990;	993;3275;3277;3685;6641
374;417;139	True;True;Tr	381;425;142	1666;1667;1	1250;1379;1	1250;1381;5017;8418;130
705;2558;43	True;True;Tr	722;2607;44	3260;3261;3	2433;2434;2	2436;9093;15795;25321
260;1147;46	True;True;Tr	265;1171;47	1167;1168;1	874;875;876;	875;4071;16721;17757;17
2118;8430	True;True	2163;8581	10006;10007	7665;7666;7	7666;30515
1850	True	1894	8793;8794;8	6772;6773;6	6773
540;668;107	True;True;Tr	551;683;109	2477;2478;2	1824;1825;1	1826;2281;3749;5499;624
1762;1866;2	True;True;Tr	1799;1910;2	8355;8356;8	6440;6441;6	6442;6824;8992;16045;18
1587;5908	True;True	1617;5993	7479;7480;7	5749;5750;5	5751;21441
838;845;847;	True;True;Tr	857;864;866;	3896;3897;3	2983;2984;2	2984;2998;3001;8177;172
602;1711;19	True;True;Tr	614;1748;19	2751;2752;2	2027;6269;6	2027;6269;6991;8691;105
1722;3258;4	True;True;Tr	1759;3312;4	8184;8185;8	6317;6318;6	6317;11671;14417;14673;
3504;3650;1	True;True;Tr	3563;3709;1	16318;16319	12598;12599	12598;13117;37224
201;3250;38	True;True;Tr	206;3304;39	915;916;917;	678;679;680;	680;11638;13911
1132;1195;2	True;True;Tr	1156;1219;2	5255;5256;5	4014;4222;4	4014;4222;7957
5761;8713	True;True	5844;8865	26906;26907	20923;20924	20925;31559
7568;9580	False;True	7706;9744	35193;35194	27338;27339	27338;34743
1752;5271;5	True;True;Tr	1789;5350;5	8309;8310;2	6409;19109;	6409;19109;20929;27854
202;1540;15	True;True;Tr	207;1569;15	921;922;923;	684;685;686;	684;5562;5653;7449;1978
977;3252;73	True;True;Tr	996;3306;75	4478;4479;4	3410;11649;	3410;11649;26648
2690;6767;6	True;True;Tr	2742;6897;6	12472;12473	9510;9511;9	9516;24682;24856;36821
4617;4730;6	False;False;T	4688;4803;6	21479;21480	16651;17092	16651;17092;23859;27322
4812	True	4886	22435;22436	17428;17429	17428
759;1530;16	True;True;Tr	776;1559;17	3507;3508;3	2630;2631;2	2632;5524;6137;12023;15
491;1675;16	True;True;Tr	500;1712;17	2249;2250;2	1667;1668;1	1667;6126;6132;7671;897
716;1200;12	True;True;Tr	733;1224;12	3298;3299;3	2466;4238;4	2466;4238;4246;4716;640
710;1684;35	True;True;Tr	727;1721;36	3278;3279;3	2448;2449;2	2448;6166;12810;16449;1
2210	True	2255	10404;10405	7965	7965
2629;2630;7	True;True;Tr	2680;2681;7	12186;12187	9306;9307;9	9306;9308;27391;36440;3
609;1694;20	True;True;Tr	622;1731;20	2786;2787;2	2056;2057;2	2058;6210;7360;7925;829
1169;3074;3	True;True;Fa	1193;3127;3	5416;14301;	4129;10967;	4129;10969;12824;14064;
1934;1935;3	True;True;Tr	1978;1979;3	9176;9177;9	7059;7060;7	7059;7067;10685;10781;1

61;62;135;15	True;True;Tr	62;63;137;15	256;257;258;	194;195;196;	195;197;458;	153;154;155;
7694;8660	True;True	7837;8812	35747;35748	27759;27760	27760;31363	
1073;1084;2	True;True;Tr	1094;1105;2	4932;4933;4	3744;3745;3	3745;3794;8164;9312;931	
1004;1092;1	True;True;Tr	1024;1115;1	4623;4624;4	3518;3519;3	3520;3840;3875;5437;609	
10209	True	10381	47640;47641	36981;36982	36982	
1390;2327;3	True;True;Tr	1418;2372;3	6469;6470;6	4973;8307;8	4973;8311;11448;16633	
9627	True	9792	44901;44902	34926	34926	
7650	True	7790	35528;35529	27595;27596	27596	
431;866;247	True;True;Tr	439;885;252	1962;1963;4	1451;3066;3	1451;3066;8803;17804;18	
1430;1770;4	True;True;Tr	1459;1809;4	6691;6692;6	5152;5153;5	5154;6474;15695	
458;671;116	True;True;Tr	467;687;118	2081;2082;2	1541;1542;1	1544;2298;4112;5247;525	
941;1599;20	True;False;Tr	960;1629;20	4331;4332;4	3306;3307;5	3306;5785;7	160
863;3639;56	True;True;Tr	882;3698;56	4006;4007;4	3058;3059;3	3059;13062;20415;24272;	
7118	True	7251	33213;33214	25826;25827	25827	
3227;3733;7	True;False;Tr	3281;3792;7	15005;15006	11533;11534	11536;13412;27055	
4264;10462	True;True	4331;10638	19854;48829	15383;37854	15383;37854	
9342	True	9501	43466;43467	33818	33818	
346;1007;11	False;False;F	353;1027;11	1518;1519;1	1134;1135;1	1137;3537;4	161;162
1734;5300;9	True;True;Tr	1771;5379;9	8238;8239;8	6358;6359;1	6359;19237;33284;34615	
687;1387;27	True;True;Tr	704;1415;28	3162;3163;3	2357;2358;2	2359;4967;9783;12982;16	
807;938;146	True;True;Tr	826;957;149	3753;3754;3	2856;2857;2	2856;3297;5265;10001;16	
420;3598;39	False;False;F	428;3657;40	1895;1896;1	1399;1400;1	1400;12924;14308;19936;	
155;2168;40	True;True;Tr	157;2213;41	683;684;685;	514;515;516;	514;7817;14732;18408;19	
176;1209;21	True;True;Tr	181;1233;22	808;809;810;	605;606;607;	607;4274;7915;14148;141	
342;4269;44	True;True;Tr	349;4336;45	1506;1507;1	1126;1127;1	1126;15408;16170;20982;	
3159	True	3213	14690;14691	11281;11282	11283	
1261;1788;1	True;True;Tr	1288;1827;1	5860;5861;5	4472;4473;4	4474;6530;6621;6817;232	
1256;3512	True;True	1282;3571	5831;5832;1	4449;4450;1	4450;12626	163
5827;6633	True;True	5911;6760	27212;27213	21146;21147	21148;24137	
1945;2994;6	True;True;Tr	1989;3046;6	9223;9224;9	7092;10650;	7092;10651;23802;29373	
2734;9521;9	True;True;Tr	2786;9683;1	12696;12697	9678;9679;3	9679;34518;35907	
396;3830;47	True;True;Tr	404;3890;48	1762;1763;1	1314;1315;1	1315;13825;17086;17551;	
1172;1174;1	True;True;Tr	1196;1198;1	5426;5427;5	4138;4139;4	4138;4146;4752;8273;334	
1882;3922;4	True;True;Tr	1926;3984;4	8912;8913;8	6860;6861;6	6860;14146;16725;17072;	
824;1537;53	True;True;Tr	843;1566;54	3830;3831;7	2922;5549;5	2922;5551;19390	
7314	True	7447	34032;34033	26419;26420	26419	
7167	True	7300	33389;33390	25945;25946	25945	
364;998;999;	True;True;Tr	371;1018;10	1609;1610;1	1200;1201;1	1204;3491;3496;6384;100	
213;761;387	True;True;Tr	218;778;394	962;963;964;	719;720;721;	719;2639;13999;15767;18	
788;871;872;	True;True;Tr	807;890;891;	3665;3666;3	2787;2788;3	2788;3078;3089;3707;539	
1178;2417;2	True;False;Fa	1202;2463;2	5461;5462;5	4166;4167;4	4169;8599;8611;15511;32	
646;3834;55	True;True;Tr	659;3895;55	2945;2946;2	2195;2196;2	2197;13844;20075;24902;	
3022;4253;6	True;True;Tr	3075;4320;6	14015;14016	10743;10744	10744;15344;22613	
1482;6253;1	True;True;Tr	1511;6368;1	6946;6947;6	5345;22616;	5345;22621;38274;38277;	
6146;9447	True;True	6241;9609	28620;44011	22246;34243	22246;34243	164

346;1007;11	True;True;Tr	353;1027;11	1518;1519;1	1134;1135;1	1137;3537;4	162;165
1923;1924;2	True;True;Tr	1967;1968;2	9124;9125;9	7020;7021;7	7020;7023;10366;22808;2	
1455;1456;2	True;True;Tr	1484;1485;2	6797;6798;6	5222;5223;5	5222;5226;7	166;167;168;
323;995;100	True;True;Tr	329;330;101	1421;1422;1	1059;1060;1	1061;3475;3	49;50;51;52;
631;1035;21	True;True;Tr	644;1055;10	2877;2878;2	2121;2122;2	2129;3633;7	120;121;122;
2213;9268	True;True	2258;9425	10412;10413	7968;7969;3	7968;33570	
1006;4764;4	True;True;Tr	1026;4838;4	4631;4632;4	3524;3525;3	3525;17246;17507;21486;	
2816;3272;3	True;True;Tr	2868;3326;3	13045;13046	9964;11704;	9964;11705;11707;22601;	
6779;8072	True;True	6909;8219	31755;31756	24725;24726	24725;29178	
285;368;467;	True;True;Tr	290;375;476;	1272;1273;1	962;1224;12	962;1226;15	171;172;173;
7669	True	7810	35621;35622	27662;27663	27662	
2275;5215	True;True	2320;5292	10643;10644	8133;8134;8	8135;18919	
944;2657;26	True;True;Tr	963;2708;27	4349;4350;4	3319;3320;9	3319;9412;9423;15970	
223;1484;16	True;True;Tr	228;1513;16	1005;1006;1	746;747;748;	748;5348;57	177
263;264;265;	True;True;Tr	268;269;270;	1184;1185;1	891;892;893;	895;898;902;5586;9944;11	
890;5092;58	True;True;Tr	909;5169;58	4109;4110;4	3138;3139;1	3139;18481;21082;21328;	
507;508;514	True;True;Tr	518;519;521	2334;2335;2	1723;1724;1	1727;1733;18607;20362;2	
5542;9778	True;True	5623;9944	25972;25973	20224;20225	20224;35560	
869;2557;49	True;True;Tr	888;2606;50	4025;4026;1	3074;9092;1	3074;9092;18068;20122;2	
603;2353;24	True;True;Tr	615;616;239	2754;2755;2	2028;2029;2	2030;8398;8	178;179
874;1075;15	True;True;Tr	893;1096;16	4051;4052;4	3099;3100;3	3099;3748;5722;7633;772	
774;1895;26	True;True;Tr	793;1939;27	3597;3598;3	2709;2710;2	2709;6898;9536;10116;12	
2685;5610	True;True	2737;5691	12445;12446	9488;9489;9	9492;20440	
2052;2622;4	False;False;F	2097;2673;4	9699;9700;9	7430;7431;7	7439;9286;1	166
86;3400;420	True;True;Tr	88;3456;426	367;368;369;	278;279;121	278;12190;15176;16629;1	
1173;2356;4	True;True;Tr	1197;2401;4	5432;5433;5	4142;4143;8	4142;8413;14879;32920;3	
1974;2586;3	True;True;Tr	2019;2636;3	9375;9376;9	7200;7201;7	7200;9182;1	180;181;182
151;928;325	True;True;Tr	153;947;331	666;667;668;	503;504;325	503;3263;11662;11669;13	
6829;10305	True;True	6959;10478	31956;48091	24872;37315	24872;37315	
38;438;1435;	True;True;Tr	39;446;1464;	163;164;165;	131;132;133;	132;1476;5167;17777;180	
1733;10040;	True;True;Tr	1770;10207;	8237;46803;	6357;36377;	6357;36378;36903	
161;3746;40	True;True;Tr	163;3805;40	716;717;718;	542;543;544;	545;13482;14441;15326;2	
164;224;138	True;True;Tr	166;229;141	735;736;101	558;559;749;	558;751;4972;14128;1578	
404;521;570;	True;True;Tr	412;532;581;	1804;1805;1	1343;1344;1	1345;1762;1936;5935;842	
579;1100;24	True;True;Tr	590;1123;25	2654;2655;2	1952;1953;1	1954;3869;8853;10358;13	
74;9630;102	True;True;Tr	75;9795;103	315;316;317;	235;236;237;	239;34943;37025	
2279;2542;2	True;True;Fa	2324;2591;2	10670;10671	8155;8156;8	8157;9047;9765;13152;19	
1273;1929;3	True;True;Tr	1300;1973;3	5923;5924;5	4526;4527;4	4527;7037;11334;13412	
1928;2688;2	True;True;Tr	1972;2740;3	9143;9144;9	7031;7032;7	7032;9503;10669;10910;1	
102;1384;15	True;True;Tr	104;1411;15	452;453;454;	342;343;344;	343;4956;5500;12958;159	
271;922;109	True;True;Tr	276;941;111	1224;1225;1	925;3242;32	925;3245;3858;8650;9939	
211;381;855;	True;True;Tr	216;388;874;	953;954;955;	712;713;714;	716;1263;30	98
457;631;246	False;False;T	465;466;644;	2066;2067;2	1524;1525;1	1530;2129;8	121;122;123;
245;466;726;	True;True;Tr	250;475;743;	1101;1102;1	816;1579;25	816;1579;2523;7560;2377	
629;2032;32	True;True;Tr	642;2077;33	2867;2868;2	2114;2115;2	2116;7365;11770;12240;1	

683;684;694;	False;False;T	700;701;711;	3140;3141;3	2338;2339;2	2338;2344;2389;10811;14	
40;1282;429	True;True;Tr	41;1309;436	171;172;173;	136;137;454	137;4549;15541;18038;18	
3451;4281;7	True;True;Tr	3509;4348;7	16079;16080	12400;12401	12401;15446;25585;2807	
3911;4099;4	True;True;Tr	3973;4162;4	18252;18253	14111;14112	14113;14793;15013;2060	
457;633;246	False;True;Fa	465;466;646;	2066;2067;2	1524;1525;1	1530;2136;8	122;123;124;
178;179;102	True;True;Tr	183;184;104	816;817;818;	611;612;613;	615;617;3585;3736;4746;	
1875;7076;7	True;True;Tr	1919;7208;7	8889;8890;8	6843;6844;6	6845;25693;27775;35563;	
578;2609;67	True;True;Tr	589;2660;68	2650;2651;2	1949;1950;1	1949;9251;24499;31593	
1249;1442;6	True;True;Tr	1275;1471;6	5803;5804;5	4427;4428;4	4428;5190;22094;38082	
4900;7801	True;True	4975;7945	22927;22928	17809;28126	17809;28126	
20;146;147;5	True;True;Tr	20;21;148;14	88;89;90;91;	74;75;76;77;	74;491;494;1	183;184
836;2451;41	True;False;Fa	855;2497;42	3889;11400;	2978;8696;8	2978;8697;15047;17741;2	
614;1395;20	True;True;Tr	627;1423;20	2806;2807;2	2073;2074;2	2074;5018;7341;9316;106	
8593	True	8744	40019;40020	31104	31104	
56;412;1023;	True;True;Tr	57;420;1043;	231;232;233;	174;175;176;	175;1362;3590;6075;8183	
6899;8936	True;True	7029;9089	32220;32221	25068;32335	25068;32337	
107;324;825;	True;True;Tr	109;331;844;	467;468;469;	353;354;106	353;1068;2924;5855;9733	
247;379;703;	True;True;Tr	252;386;720;	1110;1111;1	823;824;125	823;1258;2424;7963;9256	
300;459;133	True;True;Tr	306;468;135	1322;1323;1	994;995;155	994;1551;4732;12854;357	
217;5533	True;True	222;5613	980;981;982;	731;732;201	731;20193	
738;778;779;	True;True;Tr	755;797;798;	3405;3406;3	2548;2549;2	2550;2729;2	185;186;187;
596;688;106	True;True;Tr	607;705;108	2729;2730;2	2010;2011;2	2010;2368;3715;7928;102	
7561;8046;9	True;True;Tr	7698;8193;9	35152;35153	27307;27308	27307;29084;34816;3482	
456;537;198	True;True;Tr	464;548;203	2063;2064;2	1521;1522;1	1522;1816;7249;13221;14	
78;214;261;4	False;False;F	79;80;219;26	333;334;335;	250;251;252;	255;726;887;	10;11;12;13
1099;9123	True;True	1122;9277	5072;5073;5	3863;3864;3	3863;32984	
4252	True	4319	19798;19799	15342;15343	15343	
7845	True	7991	36417;36418	28271;28272	28271	
309;1297;37	True;True;Tr	315;1324;38	1361;1362;1	1016;1017;1	1017;4620;13556;18593;3	
68;483;608;7	True;True;Tr	69;492;621;7	290;291;292;	220;1647;16	220;1647;2055;2594;4588	
476;1294;24	True;True;Tr	485;1321;24	2189;2190;2	1626;1627;1	1628;4602;8	100;193
1072;3495	True;True	1093;3554	4929;4930;4	3742;3743;1	3742;12575	
461;1367;15	True;True;Tr	470;1394;15	2111;2112;2	1566;1567;4	1566;4895;5577;9033;938	
3738;4008;4	True;True;Fa	3797;4071;4	17409;17410	13443;13444	13444;14431;15326;2054	
37;3531;404	True;True;Tr	38;3590;411	157;158;159;	129;130;126	129;12688;14627;34858;3	
1593;2774;6	True;True;Tr	1623;2826;6	7499;12876;	5761;9824;2	5761;9824;21873;22952;2	
820;1425;37	True;True;Tr	839;1454;38	3816;3817;3	2913;5139;5	2913;5140;13614;16101;2	
192;246;580;	False;True;Tr	197;251;591;	881;882;883;	658;659;660;	660;819;1959;17807;1910	
150;1977;27	True;True;Tr	152;2022;27	664;665;939	502;7212;72	502;7212;9690;11268;125	
85;1045;265	True;True;Tr	87;1066;270	365;366;481	276;277;366	277;3666;9417;10492;208	
9457;9458	True;True	9619;9620	44071;44072	34287;34288	34288;34293	
1035;1848;2	False;True;Fa	1055;1056;1	4772;4773;4	3632;3633;3	3633;6769;9	120;123;124;
3151;5023;6	True;True;Fa	3205;5099;6	14666;14667	11262;11263	11262;18268;24839;2872	
8638	True	8789;8790	40224;40225	31280;31281	31280	
9921	True	10088	46303;46304	36015;36016	36015	

1911;3375;5	True;True;Tr	1955;3431;5	9077;9078;9	6992;6993;6	6993;12081;18959	
988	True	1007	4527;4528;4	3442	3442	
229;899;296	True;True;Tr	234;918;301	1034;1035;1	764;3169;10	764;3169;10526;24684;26	
3215;3344;5	True;True;Tr	3269;3400;5	14947;14948	11484;11948	11484;11949;19351	
4452;4843;7	True;True;Tr	4521;4918;7	20688;20689	16066;17569	16066;17570;27448;29181	
6;69;352;605	True;True;Tr	6;70;359;618	37;38;39;40;	32;33;34;35;	35;221;1167;2035;7710;1	
458;671;116	False;False;F	467;687;118	2081;2082;2	1541;1542;1	1544;2298;4112;5247;525	
17;325;777;8	False;False;F	17;332;796;8	79;80;81;143	64;65;66;106	64;1070;2728;2964;3236;	
672;1162;37	True;True;Tr	688;689;118	3088;3089;3	2299;2300;2	2299;4109;1	194;195
2654;4082;5	True;True;Tr	2705;4145;5	12314;12315	9402;9403;9	9402;14746;21012;22137;	
574;867;145	True;True;Tr	585;886;148	2638;2639;2	1940;1941;3	1940;3069;5211;16773;18	
3308	True	3362	15379;15380	11820;11821	11821	
594;1413;16	False;False;F	605;1442;16	2724;2725;2	2008;5091;5	2008;5091;5956;7656;793	
879;6082;70	True;True;Tr	898;6168;71	4075;4076;4	3116;3117;2	3116;22037;25560;32461;	
2148;3105;4	True;True;Tr	2193;3158;4	10127;10128	7754;7755;1	7754;11103;15623;35760	
623;5013;61	True;True;Tr	636;5089;62	2843;2844;2	2096;2097;1	2096;18215;22181	
896;1468;22	True;True;Tr	915;1497;22	4139;4140;4	3159;3160;5	3160;5281;8051;32819	
937;5090;78	True;True;Tr	956;5167;79	4313;4314;2	3292;18474;	3292;18476;28186;30396;	
994;1779;25	True;True;Tr	1013;1818;2	4552;4553;4	3469;3470;6	3470;6501;9001;13399;15	
177;586;841;	True;True;Tr	182;597;860;	811;812;813;	608;609;610;	608;1985;2988;8219;1137	
3384;3424;3	True;True;Tr	3440;3482;3	15754;15755	12128;12129	12129;12307;13507;1520	
2545;3331;4	True;True;Tr	2594;3387;4	11854;11855	9052;9053;9	9052;11889;15237;18440;	
1720;2506;1	True;True;Tr	1757;2555;1	8175;8176;8	6311;6312;6	6313;8908;36965	
5564	True	5645	26066;26067	20294;20295	20295	
2974;4739;7	True;True;Tr	3026;4813;7	13778;13779	10554;10555	10555;17133;25843	
2331;2743	True;True	2376;2795	10887;10888	8322;9699;9	8322;9699	
934;1266;12	True;True;Tr	953;1293;12	4295;4296;4	3278;3279;4	3279;4499;4522;10080;12	
8825;8826	True;True	8978;8979	41115;41116	31946;31947	31946;31949	
6396	True	6517	29863	23186	23186	
8520;8807	True;True	8671;8960	39665;39666	30837;31898	30837;31898	
2071;8503;1	True;True;Tr	2116;8654;1	9782;9783;9	7494;7495;7	7495;30775;38409	
266;864;109	True;True;Tr	271;883;111	1201;1202;1	906;907;908;	908;3063;3846;22694;259	
423;2160;52	True;True;Tr	431;2205;53	1919;1920;1	1419;1420;7	1419;7791;19031;20106;3	
971;1333;16	True;True;Tr	990;1360;17	4454;4455;4	3388;3389;4	3389;4737;6223;6228;770	
1448;1449;2	True;True;Tr	1477;1478;2	6767;6768;6	5205;5206;5	5206;5207;7285;7335;253	
1621;2422;2	True;True;Tr	1656;2468;2	7681;7682;7	5913;5914;5	5914;8618;9622;13722;15	
4445	True	4514	20666;20667	16052;16053	16053	
409;1154;12	True;True;Tr	417;1178;13	1832;1833;1	1356;1357;4	1357;4093;4590;5465;756	
577;1177;14	True;True;Tr	588;1201;14	2647;2648;2	1947;1948;4	1948;4164;5080;6497;851	
1322;5545;7	True;True;Tr	1349;5626;7	6137;6138;6	4703;4704;4	4704;20231;26246	
2571;4317;4	True;True;Tr	2621;4384;4	11968;11969	9135;15596;	9135;15596;17320;24324;	
29;2591;474	True;True;Tr	30;2642;482	129;130;131;	106;107;108;	106;9199;17158;23683;38	
813;2477;10	True;True;Tr	832;2526;10	3786;3787;3	2884;8820;3	2884;8820;36340	
606;692;808;	True;True;Tr	619;709;827;	2769;2770;2	2037;2038;2	2042;2373;2859;2878;298	
239;1059;21	True;True;Tr	244;1080;22	1080;1081;1	800;801;370	800;3704;7796;9962;1153	

129;391;138	True;True;Tr	131;399;140	567;568;569;	432;433;434;	432;1301;4943;5674;5830	
1107;7618;7	True;True;Tr	1130;7757;7	5108;5109;5	3893;3894;3	3893;27492;27493	
153;3829;50	True;True;Tr	155;3889;51	675;676;677;	506;507;508;	507;13824;18270;29458;3	
10248	True	10420	47821;47822	37111	37111	
3194;3681;4	True;True;Tr	3248;3740;4	14850;14851	11407;13222	11407;13223;15374;2572	
48;1769;184	True;True;Tr	49;1808;188	207;8400;84	158;6468;64	158;6468;6751;9600;1257	
910;978;199	True;True;Tr	929;997;204	4199;4200;4	3204;3411;3	3204;3412;7270;28089;28	
527;1509;19	True;True;Tr	538;1538;20	2427;2428;7	1784;1785;5	1785;5455;7133;8367;130	
3869;4975;7	True;True;Tr	3931;5050;7	18056;18057	13964;13965	13964;18078;26281;3113	
5285	True	5364	24717;24718	19177;19178	19177	
3234;4771;5	True;True;Tr	3288;4845;5	15039;15040	11561;17277	11561;17277;21138;21900	
2838	True	2890	13168;13169	10081;10082	10081	
219;681;475	True;True;Tr	224;698;482	988;989;990;	734;2334;23	734;2335;17165;22253;26	
2672;2676	True;True	2723;2727	12381;12382	9444;9445;9	9444;9459	
7519	True	7656	34998;34999	27190	27190	
5648	True	5729	26434;26435	20557;20558	20557	
815;1293;23	False;False;F	834;1320;23	3795;3796;3	2891;2892;2	2894;4591;8345;17261;25	
4412;5477	True;True	4481;5557	20508;20509	15925;15926	15926;19967	
536;2512;96	True;True;Tr	547;2561;98	2466;2467;1	1815;8936;8	1815;8937;35159;36353	
6464;6692;7	True;True;Tr	6585;6820;7	30201;30202	23440;24372	23440;24372;27799	
210;1415;21	True;True;Tr	215;1444;22	950;951;952;	709;710;711;	709;5101;7827;7991;1375	
2278;2922;5	True;True;Tr	2323;2974;5	10664;10665	8151;8152;8	8151;10367;18466;19312;	
4919;7481;1	True;True;Tr	4994;7618;1	23020;34847	17882;27070	17882;27070;36962;3696	
582;2377;31	True;True;Tr	593;2422;32	2675;2676;2	1967;1968;1	1969;8469;11405;14897;1	
724;1432;32	True;True;Tr	741;1461;33	3356;3357;3	2515;5163;1	2515;5163;11746;13123;2	
1790;6285;6	True;True;Tr	1829;6400;6	8485;29267;	6539;22724;	6539;22724;24669	
7707	True	7850	35810;35811	27805;27806	27805	
5056	True	5133	23655;23656	18367	18367	
2105;9304;9	True;True;Tr	2150;9463;1	9932;43308;	7611;33693;	7611;33694;35793	
8913;8916	False;True	9066;9069	41532;41533	32283;32289	32283;32289	
1812;10384	True;True	1851;10559	8600;8601;8	6623;37570;	6623;37572	
0;7992;9265	True;True;Tr	0;8139;9422	0;1;2;3;4;5;3	0;1;2;3;4;288	1;28864;33563	
1420;1440;1	True;True;Tr	1449;1469;1	6652;6653;6	5122;5123;5	5123;5188;6521;7517;123	
2060;2061;2	True;True;Tr	2105;2106;2	9728;9729;9	7454;7455;7	7454;7456;8107;11591;20	
162;6637;68	True;True;Tr	164;6764;69	723;724;725;	548;549;241	549;24169;24852;25744;2	
42;936;1680;	True;True;Tr	43;955;1717;	177;178;179;	139;140;141;	142;3289;6145;12914;137	
2856;6507;7	True;True;Tr	2908;6628;7	13248;13249	10139;10140	10140;23583;27349;2759	
1391;6065	True;True	1419;6151	6472;6473;6	4974;4975;2	4974;21989	
33;294;2799;	True;True;Tr	34;300;2851;	139;140;141;	115;116;117;	116;980;9922;12570;1329	
631;1035;21	False;False;F	644;1055;10	2877;2878;2	2121;2122;2	2129;3633;7	120;121;122;
2372;5077;6	True;True;Tr	2417;5154;7	11073;11074	8455;8456;1	8456;18434;25297;35137	
365;426;427;	True;True;Tr	372;434;435;	1618;1619;1	1209;1210;1	1214;1430;1433;1998;315	
4765;7506	True;True	4839;7643	22208;22209	17247;17248	17248;27148	
87;227;1032;	True;True;Tr	89;232;1052;	372;1026;10	280;760;761;	280;760;3620;5713;12959	
4803;5546;8	True;True;Tr	4877;5627;8	22394;22395	17395;20232	17395;20232;30943	

3775;4972;9	True;True;Tr	3834;5047;9	17614;17615	13612;13613	13612;18070;33398	
18;1053;142	True;True;Tr	18;1074;145	82;83;84;485	67;68;3687;3	68;3688;5127;11967;1574	
2525;5216;7	False;False;T	2574;5293;7	11784;24386	8999;18921;	8999;18921;	32
1140;1636;5	True;True;Tr	1164;1672;5	5290;5291;7	4042;5957;5	4042;5957;18557;20507;2	
512;1014;38	True;True;Tr	523;1034;39	2358;2359;2	1738;3568;3	1738;3569;14004;15969;1	
215;519;229	True;True;Tr	220;530;233	974;975;976;	727;728;175	728;1757;8191;23080;324	
1044;6495	True;True	1065;6616	4812;4813;4	3665;23547	3665;23547	
5814;7060;8	True;True;Tr	5898;7192;9	27154;27155	21105;21106	21106;25632;32445	
1262;5238;7	True;True;Tr	1289;5316;7	5866;5867;5	4476;4477;4	4478;18996;26618;27059;	
4055;8911;8	False;True;Tr	4118;9064;9	18901;18902	14643;14644	14645;32280;32288	
2389;3588;5	True;True;Tr	2434;3647;6	11136;11137	8504;12884;	8504;12884;21731	
9548;10649	True;True	9710;10827	44517;44518	34622;34623	34627;38563	
3663;7189;7	True;True;Tr	3722;7322;7	17061;17062	13159;13160	13162;26002;26004;3587	
338	True	345	1487;1488;1	1113;1114;1	1114	
2028;3522;4	True;True;Tr	2073;3581;4	9596;9597;9	7353;7354;7	7354;12655;14945;30888	
335;1476;54	True;True;Tr	342;1505;55	1479;1480;1	1109;5325;1	1109;5325;19886	
500;1971;68	True;True;Tr	511;2016;69	2298;2299;2	1698;7189;7	1698;7190;24802;27037;2	
284;5559;81	True;True;Tr	289;5640;82	1270;1271;2	960;961;202	961;20282;29352;29830	
27;88;962;56	True;True;Tr	28;90;981;57	120;121;122;	100;101;102;	101;283;3363;20734;2107	
766;767;165	False;False;T	783;784;785;	3538;3539;3	2652;2653;2	2655;2670;6	24;25;196;19
388;757;758;	True;True;Tr	395;774;775;	1730;1731;1	1292;2623;2	1292;2625;2628;23007;35	
5287	True	5366	24722;24723	19182;19183	19183	
2048;7240	True;True	2093;7373	9689;33698;	7422;26162	7422;26162	
154;568;822;	True;True;Tr	156;579;841;	680;681;682;	511;512;513;	511;1932;2915;4625;6034	
96;174;865;1	True;True;Fa	98;179;884;1	414;415;416;	312;313;314;	314;601;3064;3619;4308;	
1305;2265;8	True;True;Tr	1332;2310;8	6065;6066;6	4638;8108;2	4638;8108;29516;32416;3	
1536;2746;2	True;True;Tr	1565;2798;2	7221;7222;7	5546;5547;5	5547;9709;10232;12901;1	
5327	True	5406	24910;24911	19324;19325	19332	
10134;10330	True;True	10302;10503	47308;47309	36741;37389	36741;37389	
2584;6073;7	True;True;Tr	2634;6159;7	12012;12013	9172;9173;2	9172;22014;27058	
41;1082;222	True;True;Tr	42;1103;227	174;175;176;	138;3789;37	138;3790;8014;26614;276	
1661;2944;3	True;True;Tr	1697;2996;3	7866;7867;7	6080;6081;1	6081;10454;12797;16562	
53;1352;137	True;True;Tr	54;1379;140	223;224;225;	169;4830;48	169;4833;4916;5617;6811	
4163;6870;9	True;True;Tr	4229;7000;9	19413;19414	15038;15039	15039;24980;32835;3330	
454;455;489;	True;True;Tr	462;463;498;	2055;2056;2	1515;1516;1	1515;1519;1663;3046;305	
9239;10564	True;True	9396;10742	43017;43018	33465;33466	33466;38294	
641;2939;73	True;True;Tr	654;2991;75	2923;2924;2	2178;2179;2	2180;10422;26685;38458	
1255;2392;2	True;True;Tr	1281;2437;2	5829;5830;1	4448;8507;8	4448;8507;9953;11512;11	
1446;2766;5	True;True;Tr	1475;2818;5	6759;6760;6	5199;5200;5	5199;9790;1	55
1784;2789;3	True;True;Tr	1823;2841;3	8458;8459;8	6517;9886;9	6517;9886;13625;15101;1	
373;460;106	True;True;Tr	380;469;108	1660;1661;1	1244;1245;1	1247;1556;3	198;199;200;
49;1637;438	True;True;Tr	50;1673;444	208;7751;77	159;5960;15	159;5960;15809;20647;26	
10520	True	10696	49144;49145	38096;38097	38096	
5956	True	6042	27823;27824	21622;21623	21624	
4270	True	4337	19877;19878	15410;15411	15413	

2481;10354	True;True	2530;10527	11568;11569	8837;8838;3	8837;37459	
7052;8131;8	True;True;Tr	7184;8280;8	32913;32914	25615;29452	25615;29452;32004;37704	
407;1502;54	True;True;Tr	415;1531;54	1822;1823;1	1351;5432;5	1351;5433;19742;36332	
377;1839;33	True;True;Tr	384;1882;33	1680;1681;8	1256;6736;1	1256;6736;11843;15423;1	
1015;5319;5	True;True;Tr	1035;5398;5	4682;4683;4	3571;19293;	3571;19293;19350	
1498;3623;5	True;True;Tr	1527;3682;5	7033;7034;7	5408;5409;5	5410;12988;20589;27604;	
35;58;101;58	True;True;Tr	36;59;103;59	148;149;150;	122;182;183;	122;185;338;1992;3648;25	
3165;7911;7	True;True;Tr	3219;8058;8	14716;14717	11303;28572	11303;28574;28605;37012	
734;1379;32	True;True;Tr	751;1406;33	3388;3389;3	2537;2538;2	2539;4939;11734;22650;2	
353;2308;37	True;True;Tr	360;2353;38	1561;1562;1	1168;1169;1	1170;8235;13683;21237;2	
5288	True	5367	24725;24726	19184;19185	19186	
8546	True	8697	39774;39775	30917;30918	30922	
288;1252;50	True;True;Tr	293;1278;51	1282;1283;1	968;969;444	968;4444;18448;22171;24	
2627;3981	True;True	2678;4044	12179;12180	9300;9301;9	9300;14334	
3135;4586;4	True;True;Tr	3189;4657;4	14591;14592	11205;16548	11205;16548;17888;33452	
8;649;799;18	True;True;Tr	8;662;818;18	47;48;49;295	42;43;44;220	43;2202;2828;6753;13569	
842;3357	True;True	861;3413	3908;3909;3	2991;2992;2	2992;11999	
64;100;434;8	True;True;Tr	65;102;442;8	267;268;437;	202;203;332;	202;332;1461;2847;2848;2	
823;4000;10	False;True;Tr	842;4063;10	3824;3825;3	2918;2919;2	2919;14405;37186	
6086;9188	True;True	6173;9344	28369;28370	22059;22060	22059;33265	
34;248;1179;	True;True;Tr	35;253;1203;	145;146;147;	120;121;825;	121;825;4172;11370;1432	
6196;8298;8	True;True;Tr	6302;8449;8	28884;28885	22438;30034	22438;30035;30590	
1422;3061;3	True;True;Tr	1451;3114;3	6659;6660;6	5128;5129;5	5128;10922;13807;19970;	
1542;5467;7	True;True;Tr	1571;5547;7	7251;7252;7	5566;5567;1	5566;19945;25497;29037;	
93;848;1962;	True;True;Tr	95;867;2007;	402;403;404;	306;307;308;	307;3004;7150;9261;1179	
1407;2574;3	True;True;Tr	1436;2624;3	6592;6593;6	5072;5073;5	5075;9144;1	180;181
3678;4946;5	True;True;Tr	3737;5021;6	17135;17136	13216;17996	13216;17996;21514;2173	
3240;5264;6	False;False;F	3294;5343;6	15069;15070	11580;11581	11583;19089;24730;2916	
419;1883;31	True;True;Tr	427;1927;31	1877;1878;1	1386;1387;1	1397;6867;11166;25590;2	
57;7658;106	True;True;Tr	58;7798;108	237;238;239;	177;178;179;	178;27620;38559	
332;390;502;	True;True;Tr	339;397;398;	1465;1466;1	1099;1100;1	1105;1297;1	205
55;856;1586;	True;True;Tr	56;875;1616;	229;230;397	172;173;303	172;3039;5743;14125;156	
1279;1475;2	True;True;Tr	1306;1504;2	5937;5938;5	4535;4536;4	4537;5321;9228;10276;21	
117;1188;13	True;True;Tr	119;1212;13	510;511;512;	384;385;386;	387;4202;4899;7340;1885	
415;563;706;	True;True;Tr	423;574;723;	1856;1857;1	1373;1374;1	1373;1920;2438;3829;425	
2843	True	2895	13192;13193	10098;10099	10100	
704;1847;18	True;True;Tr	721;1891;19	3254;3255;3	2427;2428;2	2432;6761;6840;7009;820	
83;632;2106;	True;True;Tr	85;645;2151;	360;361;362;	271;272;273;	271;2132;76	123;124;126;
3230;3690;6	True;True;Tr	3284;3749;6	15020;15021	11547;11548	11548;13240;21938;2412	
1705;1849;1	True;True;Tr	1742;1893;2	8092;8093;8	6250;6770;6	6250;6771;7151;19829;21	
627;4366;52	True;True;Tr	640;4435;53	2861;2862;2	2108;2109;2	2110;15774;18983;20114;	
924;3240;52	True;True;Tr	943;3294;53	4260;4261;4	3248;3249;3	3251;11583;19089;19193;	
6182;10318;	True;True;Tr	6285;10491;	28806;28807	22388;37352	22388;37352;37999;3851	
1260;3123;3	True;True;Tr	1287;3177;3	5857;5858;5	4471;11162;	4471;11163;12304;26781	
46;1114;607	False;False;F	47;1138;615	192;193;194;	147;148;149;	151;3938;22011;27078;28	

205;947;980;	True;True;Tr	210;966;999;	931;932;933;	694;695;696;	695;3325;3421;5650;7307	
1057;3438;3	True;True;Tr	1078;3496;3	4863;4864;4	3698;3699;3	3699;12344;13868;15032;	
5995;6125	True;True	6081;6217	28003;28004	21764;22178	21764;22180	
3148;4769	True;True	3202;4843	14651;14652	11253;11254	11254;17268	
1747;3811;5	False;True;Tr	1784;3871;5	8290;8291;8	6397;13763;	6397;13763;18522;27147;	
132;773;117	True;True;Tr	134;792;119	589;590;591;	451;452;453;	453;2707;4137;8672;8675	
826;2796;36	True;True;Tr	845;2848;36	3836;3837;3	2927;2928;2	2928;9912;12974;13757;1	
2361;4436;7	True;True;Tr	2406;4505;7	11032;11033	8430;16020;	8430;16020;25595	
47;3624;588	True;True;Tr	48;3683;597	201;202;203;	155;156;157;	156;12993;21365;27436;3	
349;2697;38	True;True;Tr	356;2749;39	1531;1532;1	1142;9533;1	1142;9533;14000;15149;2	
2695;10154	True;True	2747;10322	12502;12503	9529;9530;3	9530;36800	
16;561;1033;	True;True;Tr	16;572;1053;	76;77;78;259	63;1913;362	63;1913;3626;6040;20433	
1602;5543;5	True;True;Tr	1632;5624;5	7549;7550;2	5805;5806;2	5806;20229;20921	
1556;2621;6	True;True;Tr	1585;2672;6	7326;7327;7	5624;5625;5	5627;9283;21798;22199;3	
3457;3672;5	True;True;Tr	3515;3731;5	16107;16108	12424;12425	12424;13189	206
2189;8836;9	True;True;Tr	2234;8989;9	10323;10324	7913;31978;	7913;31980;33281	
1467;7096;1	True;True;Tr	1496;7228;1	6863;6864;6	5277;5278;2	5278;25733;38627	
371;699;902;	True;True;Tr	378;716;921;	1654;1655;1	1239;1240;1	1241;2406;3177;4491;471	
1181;5701;6	True;True;Tr	1205;5783;6	5476;5477;5	4181;20754;	4181;20754;22672	
1740;4709	True;True	1777;4781	8261;8262;8	6376;6377;6	6377;16999	
238;270;789;	True;True;Tr	243;275;808;	1068;1069;1	788;789;790;	793;923;2791;2795;3255;3	
26;75;7037	True;True;Tr	27;76;7169	114;115;116;	94;95;96;97;	96;243;25579	
6256	True	6371	29147;29148	22635;22636	22636	
9961	True	10128	46475;46476	36141;36142	36141	
2163;3142;4	True;True;Tr	2208;3196;4	10186;10187	7798;7799;7	7801;11234;17661;20155;	
10;183;334;6	True;True;Tr	10;188;341;6	53;54;55;837	47;48;49;625	47;626;1108;	207
120;4456;46	True;True;Tr	122;4525;46	524;525;526;	396;397;160	397;16075;16599;17100;2	
1239;4908;5	True;True;Tr	1264;4983;5	5755;5756;5	4385;17838;	4385;17838;21176	
52;1626;223	True;True;Tr	53;1661;227	220;221;222;	168;5925;59	168;5926;8017;8538;9797	
1700;5494;6	True;True;Tr	1737;5574;7	8071;8072;8	6232;6233;2	6233;20046;25306	
319;968;468	True;True;Tr	325;987;475	1400;1401;1	1045;3378;3	1045;3379;16925;18179;2	
174;1219;13	False;False;F	179;1243;13	802;803;804;	599;600;601;	601;4308;4706;9979;1017	
2146;4185;7	True;True;Tr	2191;4251;8	10115;10116	7744;7745;7	7746;15107;28846	
366;522;639;	True;True;Tr	373;533;652;	1624;1625;1	1215;1216;1	1218;1767;2175;6486	
10021	True	10188	46732;46733	36329	36329	
240;10652	True;True	245;10830	1083;1084;1	802;803;804;	805;38572	
9856;10634	True;True	10022;10812	46040;49678	35819;38513	35819;38513	
114;736;169	True;True;Tr	116;753;172	501;502;503;	380;2542;25	380;2543;62	208
204;852;378	True;True;Tr	209;871;384	929;930;395	692;693;302	693;3028;13631;15685;18	
7127	True	7260	33232;33233	25840;25841	25841	
898;2781	True;True	917;2833	4151;4152;4	3166;3167;3	3168;9844	
450;1559;17	True;True;Tr	458;1588;17	2041;2042;7	1506;5635;5	1506;5636;6294;7699;803	
3282;3626;3	True;True;Tr	3336;3685;3	15277;15278	11744;11745	11745;12999;13194;1323	
6355;9930	True;True	6470;10097	29638;29639	23018;23019	23019;36043	
2819;4222;9	True;True;Tr	2871;4288;9	13057;19659	9977;15226;	9977;15228;32580	

1642;8360;1	True;True;Tr	1678;8511;1	7785;7786;7	6019;30275;	6019;30276;37388	
23;24;329;64	True;True;Tr	24;25;336;65	101;102;103;	83;84;85;86;	84;89;1084;23310;31847;	
2628	True	2679	12182;12183	9303;9304;9	9305	
770;8422;84	True;True;Tr	789;8573;86	3579;3580;3	2693;30467;	2693;30467;30695;30896;	
4500;6135;6	True;True;Tr	4570;6228;6	20931;20932	16260;22209	16260;22210;24524	
8820	True	8973	41098;41099	31935;31936	31935	
2540;6601;9	True;True;Tr	2589;6727;9	11838;11839	9041;9042;2	9042;23997;33109;33656;	
744;4631;59	True;True;Tr	761;4702;60	3430;3431;3	2568;2569;2	2570;16708;21556;27617;	
1949;2379;2	False;True;Tr	1994;2424;2	9249;9250;9	7113;7114;7	7113;8476;8657;9004;976	
783;7486	True;True	802;7623	3648;3649;3	2773;2774;2	2774;27087	
226;9468	True;True	231;9630	1023;1024;1	757;758;759;	759;34317	
302;343	True;True	308;350	1333;1334;1	999;1000;10	999;1130	
518;2065;20	True;True;Tr	529;2110;21	2379;2380;2	1754;1755;1	1755;7463;7589;8062;221	
1047;2964;4	True;True;Tr	1068;3016;4	4826;4827;4	3672;3673;1	3673;10521;15320;28909;	
273;1525;28	True;True;Tr	278;1554;28	1230;1231;1	929;930;550	930;5504;9946;36436	
2777;2787	True;True	2829;2839	12891;12892	9834;9835;9	9835;9879	
1728;2116;3	True;True;Tr	1765;2161;3	8216;8217;8	6343;6344;6	6347;7659;14129;21115;2	
283;4061	True;True	288;4124	1267;1268;1	957;958;959;	959;14660	
1372;5109;5	True;True;Tr	1399;5186;5	6387;6388;6	4909;4910;4	4909;18534;19736;21553;	
2675	True	2726	12401	9458	9458	
2587;5624	True;True	2637;5705	12029;12030	9188;20487;	9188;20488	
9976	True	10143	46542;46543	36194;36195	36196	
392;735;148	True;True;Tr	400;752;151	1747;1748;1	1304;1305;2	1304;2540;5344;9949;174	
401;560;180	True;True;Tr	409;571;184	1789;1790;1	1331;1332;1	1331;1912;6594;7233;794	
242;4509;56	True;True;Tr	247;4579;57	1091;1092;1	808;809;162	808;16294;20634;20864;2	
1227;5764;6	True;True;Tr	1251;5847;6	5693;5694;5	4334;4335;4	4334;20933;22820;28663	
341;2723;31	True;True;Tr	348;2775;31	1503;1504;1	1124;1125;9	1124;9629;11149;18010;2	
1659;2938;3	True;True;Tr	1695;2990;3	7860;7861;7	6076;6077;6	6076;10415;11874;15549;	
985;2691;42	True;True;Tr	1004;2743;4	4515;4516;4	3436;3437;3	3437;9521;15244;16093;1	
50;607;1532;	True;True;Tr	51;620;1561;	209;210;211;	160;161;162;	160;2051;5537;7185;7391	
550;1026;18	True;True;Tr	561;1046;18	2532;2533;2	1868;1869;1	1870;3601;6580;8902;899	
4137	True	4202	19296;19297	14954;14955	14954	
3941;6074;6	True;True;Tr	4003;6160;6	18388;18389	14217;22015	14217;22016;23587;28531	
3660;6971;1	True;True;Tr	3719;7102;1	17053;17054	13155;25345	13155;25345;37310;38541	
470;490;164	True;True;Tr	479;499;168	2146;2147;2	1588;1589;1	1592;1666;6	209
648;732;175	True;True;Tr	661;749;179	2954;2955;2	2201;2534;6	2201;2534;6432;9686;127	
850;860;171	True;True;Tr	869;879;175	3941;3942;3	3017;3018;3	3020;3054;6	210
1861;1983;2	True;True;Tr	1905;2028;2	8840;8841;9	6809;6810;7	6810;7240;8105;9838;128	
893;1137;17	True;True;Tr	912;1161;18	4118;4119;4	3142;3143;4	3143;4037;6505;7702;821	
192;663;184	True;True;Tr	197;678;188	881;882;883;	658;659;660;	660;2259;6739;7695;9927	
2476	True	2525	11547;11548	8817;8818;8	8818	
487;1097;16	True;True;Tr	496;1120;16	2227;2228;2	1656;1657;1	1657;3861;6059;7971;123	
3477;10542	True;True	3535;10718	16207;16208	12511;12512	12512;38174	
7012;8639;9	True;True;Tr	7144;8791;9	32732;32733	25474;25475	25475;31287;35145	
1960;2466;4	True;True;Tr	2005;2514;4	9295;9296;9	7139;7140;7	7140;8780;17385	

2332;4968;6	True;True;Tr	2377;5043;6	10890;10891	8323;8324;8	8324;18060;23391	
104;3726;42	True;True;Tr	106;3785;42	458;459;460;	346;347;348;	348;13389;15177;18649;2	
122;355;113	True;True;Tr	124;362;115	532;533;156	399;400;117	400;1174;4011;5305;7075	
3899;6720;9	True;True;Tr	3961;6849;9	18207;18208	14081;24478	14081;24478;34457;34621	
658;1216;22	True;True;Tr	673;1240;22	3009;3010;3	2241;2242;4	2242;4297;7998;9945;104	
77;711;1055;	True;True;Tr	78;728;1076;	329;330;331;	246;247;248;	246;2452;3694;5700;5921	
2051;3651;4	True;True;Tr	2096;3710;4	9696;9697;9	7427;7428;7	7429;13121;16140;25374;	
4351;4368;7	True;True;Tr	4420;4437;7	20235;20236	15726;15727	15727;15778;28241;30077	
6076	True	6162	28321;28322	22019;22020	22020	
5325	True	5404	24893;24894	19313;19314	19313	
4952	True	5027	23192	18017	18017	
5072	True	5149	23724;23725	18420	18420	211
9908	True	10075	46261;46262	35985	35985	212
6262	True	6377	29171;29172	22657;22658	22659	
4567;5730	True;True	4638;5812	21259;21260	16499;20833	16499;20833	
10536	True	10712	49215;49216	38152	38152	
4193	True	4259	19551	15143	15143	
6552	True	6677	30613;30614	23774;23775	23774	
5586	True	5667	26165	20365	20365	
4916	True	4991	23008;23009	17870;17871	17871	
8864	True	9017	41294	32077	32077	
2441	True	2487	11363;11364	8667;8668	8668	
5984	True	6070	27949;27950	21719	21719	
9528	True	9690	44407;44408	34530;34531	34531	
2240	True	2285	10506;10507	8032;8033;8	8035	
10079	True	10247	47010;47011	36531;36532	36531	
952;1643;10	True;False;Fa	971;1679;10	4370;4371;4	3335;3336;3	3336;6021;37114	
90;230;1567;	True;True;Tr	92;235;1597;	381;382;383;	288;289;290;	288;765;5675;9617;12664	
2085;2252;2	True;True;Tr	2130;2297;2	9849;9850;9	7544;7545;7	7547;8060;9869;9872;166	

Oxidation (M) site positions
39;14711;31108

33;18482;21659;24776;25930;28761;30376;35736
3;18756;36912

32995;33001
734;13142;18846;22574;36237

1107;36978

3;13392;30791

1;9702;10288;13158;14351;17646;21673;22243;36472

7;4688;9425;9482;9601;10150;15440;15941;17150;23308;24477;24481;25788;26007;26615;26857;29832;30440;31190;3
32537;32539;36165;38323
60;63

3;8525;10283;12650;13027;13265;15567;15791;17821;19167;21017;21964;30649;34543;38200;38556

562;15597;16670;21908;23338;36469;38029

5;9542;12755;14100;36946;37798

45;25348;25938;38280
35;20571;27283;28279;29893;33017
539;25918;26600;32317;34004;35941;37167

039;20937;21494

2126;33252;36736

4;6079;9843;11819;13232;14958;26013;27358;28723;29081;30707;31248;32393;34149

72;22091;30221;31725;36940

350;23014;23862;30188;30252

10;16300;18811;26400;28528;35182;36140

223

27744;31969

768;17600;20512;20551;26874

16924;19358;26482;30312

303

3;15024;24162;26435;30685;30686;37135

21;23232;34173;37101;37230;38489
12;12025;16662;17096;21843;21848;22545;23101;26626;27687;32823;33431;37563;38076
43;180

27;42;321

02;16475;19169;20811;24135;25051;27756;33813
411;25063;27360

91;23256;27804;27944;32057;32637;33658
2150;13375;31210
51;34206;34709

10111;10113;28178
357;35798;37216

70;24456;25426;26982;29469;38014

49;13577;21097
29795;35378

53;26234;28709;29543;30905;38411

10977;11008;13445;13949;16549;16573;21630;24127;24424;24429;26880;27652;29463;32666;33770;35161;37032;3713

3041;36742
538;34295;35570

12795;14503;15530;21063;21868;32767;33963;35230;35252;36819

10;28827;29027

5;3409;3642;4326;5258;8039;9674;10184;10370;11878;12167;13274;13919;13953;14181;15272;18574;20955;28706;3107

7;6846;9484;10878;11347;11775;12730;12731;16543;18506;22792;28823;31769;32079;32902;34563;34922;36950
449;17079;21695;26218;33464

4327;15526;30118
26523;30008
508;25033;31900;32311;36211;37423

10305;10926;15007;15983;18583;25406;27096;28024;34795
5;7235;11276;13600;20484;21694;25462;25984;26319;32720;33267;35206;38347;38348
141

438;977;989;2034

5909;11699;12614;20370;21215;25101;26579;27443;29587;30944;31297;31705;31712;32528;32988;37381

3;3387;3516;3680;8563;16295;17241;26145;28548;33367;33437;33899
33;28628;28631;34238
4932;36796

11;21950;24568;25617;36718;37777

7;7028;9794;13057;13709;14398;17747;18655;18662;22615;24851;28716;35738;35872;36000;37687

1498;4212;5947;10290;12612;13054;14953;15835;16512;17916;19499;20454;20478;20590;21171;21403;21790;23246;25
1;5079;5616;6588;7332;10349;10734;11443;15430;15963;16258;19265;23523;24979;26606;26862;27766;30326;30573;30

30368;35633
271;345

308;7180;8148;8569;9531;9979;10174;16256;18365;22628;23432;23575;23578;23784;24528;25801;26172;29358;31703;

3;9587;13923;16261;25470;31109;33746

6365;26479;26481;29185

136;10671;12682;14106;15575;23100;28757;33975
95;33118;33235;33456;33538;33761

3181;38183

99;15846;18816;22160;22932;22934;23773;33962;34705;35511;38056

274;33585;33586;34804;35201;37790;37841
8487;9436;17394;26354;28120;28168;31136;31493;31894;33540;33583
30;12239;16702;16911;35929

711;33773;36970
9;9991;10870;10885;15993;16480;24957;29228;38370
65

0;7259;20826;30281
2;4377;6492;18700;32010;38095

56;24838;28324

8506;8852;14561;15562;21238;22608;25209;27670;29462;30279;31365;32153;36980;37220

51;14416;15269;17666;17826;19782;30541;32344

4617;5575;7501;7526;9597;11919;15340;18019;18441;20510;23761;24417;24721;25121;25158;25954;26922;30839;3090
1;10297;11355;12135;24331;27439;35874;37739
193;327

660;4277;4278;5240;6417;6426;11940;12159;14287;17012;18580;18878;19254;22078;22284;23210;23535;27243;30531;

50;11577;18967;22556;23629;26335;28795;33838

285;22748;36896
5710;6999;14185;15169;15558;17018;23529;28732
061;17601;17741;24875;24988;26276;26278;29204;34079;35732
137
2;21476;29382;37112

0;7376;7378;20491;22968;26595;27435;29012;29523;29565;32867;33050;33321;38588

38

059;12897;14085;17407;19689;24186;24787;25162;27901;27904;30672;37866
36;12445;12606;15602;22592;33903;38584

514;28725;35274;36997;37718

99;13511;13514;17164;25619;31049;35759;37666;38612;38616

4;14308;24629;31999

12564;33490
5;13146;13224;14767;20736;28576;33379;37148
25104;27566

58;24925;26314;26645;27992

39;14655;14658;23685;27440;29986

39;12494;15253;22720

30;4281;4394;4397;4743;7091;8625;9534;10623;10845;11011;12756;13125;13611;14227;16425;17001;18369;20516;224
19339;20717;20790
8787;19078;21337;21638;36216;36328;36369;36579

01;13400;27856;29840;36627

588;30542;35358
215

39;19311;23670;23673;29573;33883;36256;36259;37076;38403

70;18227;19037;22008;24223;24453;25473;25977;30244;32694;32963

4315;35549
V6C5;Q4W6C3;Q4W6C0;Q4W5U0;Q4VYE6;Q4LAU0;Q4A1H0;Q45XP

;Q4ZG93;Q4W6C5;Q4W6C3;Q4W6C0;Q4W5U0;Q4VYE6;Q4LAU0;Q4A1H0;Q45XP

273;273;273;273;273;273;273;273;273;273;273;273;273;273;273;273;273;273;273;273;273;273;273;273;273;273;273;273;273

0;8454;22479;22737;34146
5;7656;7937;8046;17730;22639;25266;26412;27500;29983;34742;36410

9844;36344

1897;37824

173
9;6392;8099;11002;14754;15538;16777;20408;22747;25854;26328;28235;29631;34607;34983;36152;36503;37592

5952;37597

1190
59;15462;21855;24419;26669;33313;35938;35939

707;23064;25871;30408;31941;31992

6315;31385;33236;33980
4;10282;11016;14239;25059;30373;36425

27503;31289

380;12067;14152;15771;17168;17843;18065;25828;26023;26323;35227;36309

10385;22017;24175

120;18452;21922;24124;25833;25835;25885;25936;26535;26536;32341;34052

46;26721;30619;32030;34438

50;13579;15300;18393

6021;10320;17030;20680;20917;24329;29803;34372;34415;34421;36530;37114

1;8503;12995;16301;18397;21006;23289;24765;31878;35911;37838;38165

3673;34961

30;16709;28423

3;6969;7041;9203;10936;17171;24280

337;377;488;490;496;549;552;568

7472;37110;37439

3;27540;30333

390;23911;27050;30351;37280;37284

6363;9469;14318;15434;27253;28656

461;17664;19303;27383;37026
29;12740;14292;15223;15723;16994;20304;21941;23048;23531;26387;26852;27295;29088;31175;32315;37017;37639
2;8020;8991;9545;24969;32685;35123;35592;38617
9;9056;9058;9480;9765;10308;11099;11304;13152;13195;14084;14571;16948;19893;19899;22920;23957;25180;27416;34

591;32156;36641

3;5762;6125;6275;7782;7788;9970;10541;11005;12313;12872;13801;16558;17375;18545;22365;25292;26228;26565;277
519;16090;18063;28826;31973
7707;11528;23194;30903;34018;34273

4529;4856;9345;13838;14853;15890;16540;19282;20185;21199;21821;21825;22568;22652;2

3;15037;18141;19932;20564;20891;29100;36040;36311;36782

02;15078;16751;19088;26907;37244;37510

59;16143;17288;17660;18958;24926;31059;35335;37295

4;20291;23267;26651;31021;36794
0570;26714

5022;7578;16297;16354;16835;17311;17528;27331;27733;27736;34121

05;13508;14453;14692;21912;30667;31093;31094;31830;31921
32;22634;35356
22107;22109

3;10480;13167;14183;24365;25332;26670;35296;38105

9;13295;13843;27933;28220;32986;35132;35635;35641;37619
56;16276;16281;16331;27965;27975;28435;28446

34886;37383

225
5;3734;4748;5894;8199;8513;10426;11998;13650;13984;15823;17286;20306;29229;29772;29853;30669;34434;34539;370

950;16971;17381;19924;21787;24950;27479;28020;32061;32959;33044;33412;33568;34209;37480
168;20821;21962;29167;29418;33723
633;21889;32933;33535;34842

3;6338;8811;11588;12101;12180;12939;13520;14541;15537;15942;16815;18235;20330;20334;21604;25132;26640;34771

7;15230;16940;18706
127

7818;33085;35716

951;6433;9117;9127;9632;11871;12287;12367;15755;16979;17282;17981;17983;19859;21018;21020;23350;23398;23408
582;21258;28721

506;20757;23127;25785;26091;35626;38501
285;12320;15054;17836;23024;27135;27919

438

16;21705;23431;30914;34724;37323;38135;38607

56;16160;21044;23122;28538

20;21759;33661
5;4339;6014;6225;7504;7514;9985;10091;11466;11485;12474;15837;16498;22309;22571;24844;25338;26127;27611;2845

61;100

71;13951;16585;18124;23094;30779;35679
19561;32106

1;13248;25649;27853

78;19341;20347;33692;33714;35102;37028

9;9082;9426;13379;15416;17766;20760;20861;24462;26698;28013;35862;36004
269;326;441
46;10362;12407;16579;18074;19083;19575;20068;20162;24819;27474;32107;32398;32399;34701

04;23124;24933;24947;32966

55;25688;27608;27718;29429;35936
58;16069;21493;22897;24640;27860;33282;33993
29520;32655

3384;23791;24652

150;13366;14424;15128;15908;34358;37039;37678

393

9972;29100

1;18203;19694;20529;24964;24975;28103;31675;32415;35143;36784

13;13447;16791;19155;22549;25773;27126;32872;32876;33722;37431;38013

2;9604;10963;14326;19741;21474;22872;32541;32832;34940

0;7193;8002;8004;11806;14056;14404;14816;19070;19215;20048;20055;20988;21120;21126;21641;22493;26203;30874;3

3;14885;27042;28741;28881;30968;33268;33905

110

93;26212;37436

11;11784;11879;13993;15043;31562;34382;36030;38610

38

1;9956;12403;28240

5165;5637;6106;7920;8523;8637;12370;16253;16423;16589;17999;19188;22812;25576;29066;29467;34700;36694

5;11165;25095

30;17237;29443;29445;29913;35494;37879

5;9248;11424;14568;20976;21250;21484;32431;36147;37475;37477

3;14622;22031;23643;27423

85

35

74;104
1546;22892
0;23895;24156;34949;36599;36604;37263;37268

53;15890;20181;20184;30825;33792;34931
20837;26947;30709;34890;37424
597;27220;27655;29640;31665;31741;32176;33711;36907;38483

21
5;4908;6206;6620;6886;7947;8558;10624;12467;12925;14797;19411;21465;31011;31020;31400;32895;33155;33894;3389

4;9978;11654;18975;19318;21234;25167;25563;29349;29361
951;6433;9117;9119;12287;12367;15755;16979;17981;17983;19859;21018;21020;23350;23398;23408;23457;23719;2562

3;11161;17008;18864;20058;32164;32422

4;9698;11142;12014;28646;29336
124
3;10245;16710;19374;28141

2011;27078;28654;32085
00;23821;26138;27523;37794
571;26525;29119

1
328;30785;31230;31256;38443
77;30402;34338;35746

775;3163;3195;11801;11849;14294;18026;18080;19035;21928;21933;22853;25646;25844;31583;36089;38415

2385;10799;10809;12752;15653;37816

9;8611;15511;32450;32853;32855

3;4078;4307;6180;6182;7030;7313;11813;12905;15673;18161;20896;21883;24592;24880;25123;25125;25620;25983;2695

489;18521;19876;29597;35845
50;11825;18008;19288;21260;21838;22941;25700;26305;28295;29924;30083;32424;33564;36922
99;3282;4128;4519;10588;15029;16591;20499;21052;31740;36013;36084;36420
351

3;9909;19918;25513;27159;32841
195;12371;36115
32257;34964
12635;17147;24265;24308;26546;27123;36537
75;28327;33123;36733
7326;9163;9332;10553;11290;12299;16161;18837;19112;22964;27795;28315;30510;31203;32688;34283;35753
4;14332;17644;21667;23868;23943;24487;29112;31376;32706;32788;33996;38119
183
29816;36671

3;1998;3155;3834;8532;9211;11013;12659;15257;16084;22776;22971;24233;26977;28936;29041;32742;32881;32886;347

3;3056;9200;14637;14775;28102

353;15909;16473;18639;19412;22670;25719;27274;28078;31932;35062;36173
267;278;342;363;378

33498;38592

08;12623;20196;22573;27453;28357;33172
54;30808;31386

9055;9947;29788;32577;35967

0;10097;10124;13442;14728;19271;21542;23215;23560;24465;25891;27255;28567;29539;29684;35660;35776

3;8481;11144;12622;15194;18945;21781;22758;26287;26528;31354;31933;32321;35326;37453
31;10792;10907;14251;14378;14386;16755;16960;18619;22177;26702;30495;37374;37801;37895;37900
4;11708;11768;19013;22914;23167;32524;34227

43;180

34;14633;16500;17529;17535;21224;23931;26936;29337;31865;33177

5;11430;17237;28836
0;17731;22028;23462;32371;33812;38295;38419

3635;30594;32608;34016;34169;35049;36235;36450

03;17490;22948;30466;34894

098;21108;21223;30090;30365;30869

0;14645;28025;32271;32278
31;14725;20515;27271;27390;30824;33420;34397;36024;36780

5949;28125;36118

750;8914;15448;19378;33076

);14962;22089;28835;31788;32601;32604

9;4532;8131;9136;14495;15548;15575

2;6406;6673;8287;8288;10148;11206;17154;24917;26578;26876;30088;30603;32521;33698;36520

130;32789;33956;35650;37185
9;28130;33754

5345;16894;23958;35532;35710;36007
792;12933;23486

56;11242;11966;14089;14353;18058;23059;23925;24928;26140;26289;29798;31498
3;4452;5141;7593;8494;10176;12995;13884;16301;18301;18397;20299;21006;23289;24765;26311;31003;31882;35911;37
94;26570;36693;38059

52;16715;17521;27723;29512;34070;37045
35
20377;21728;27273;30462;30579;35272;36828;37128
9;13604;15661;20829;20831;27237;29393;29680;31595;31862;32070;32583;33805;34339;35019;37862

05;16938;20866;21286;21618;21621;29568;30217;31553;32765;32997

37
7;4040;6257;6711;7465;11690;12528;18377;24896;25701;31418;32435;34850;36270;38499

11430;16308;33308
5;8362;8651;10497;18603;19964;22027;22682;24484;24897;25038;27287;27290;30283;30847;33786;34860;35651;35653

9;6330;6334;7610;13540;14701;14706;16087;31235;38334
11;15262;23327
19;30964;36295
72;19875;21110;29423
47;27664;31275;31692;33817
59;28608;29518;29617;31199;33668
7381;30182

4176;16137;22603;33328

3;8678;12405;21142;28818;31351;31369
3064;3953;4586;4706;7180;7462;8027;8029;9681;9979;10174;17599;17728;20643;22970;23185;23575;23780;24530;247

4010;12103;16928;28212

13396;15048;15702;15788;18778;21444;24320;25005;27375;30404;32542;37105;37107;38332

5;9787;10670;14789;14790;16467;16546;18295;21935;22924;23293;25057;26221;29863;29981;30882;32545;32908;3731

56;25507;35152
9624;34529;37134

0;7837;12583;15675;16043;22906;29109;35830
156;25138;35249

[illegible]51;17741;18452;20535;21922;24255;25931;26532;26533;26848;27694;32341;32567

465
329
9117;9127;9632;16979;17981;17983;19859;23457;30322;31319;37624;37661
94
119;237;257;272;368;376
148
135
79;22687;26967;32540
185
95
3095;33099;36069;36072;37258
111;469;571

338;6433;9117;9127;9632;12287;12367;15755;16979;17282;17981;17983;19859;21018;21020;23350;23398;23408;2345[illegible]
3;9117;9127;9632;15810;16988;18491;18554;19870;21885;23398;23408;25655;25845;29063;31861;32554;37624
193;430;550
121;239;259;274;296
150;271;291;306;321
274;303

245;269;276;286;296;326;411
263;300;473
399;410
226
741;18120;18452;20535;21922;25936;26535;26536;26848;27694;32341;34052
8460;10815;30540

9127;9637;16979;19859;23460
9117;9127;9632;12157;16988;19870;27545;28717;29046;31329;34698

4569;15047;17741;18120;18452;20532;21922;23163;25829;25831;25936;32574
09;26243;28391;31181
433
275;304
269;380;391

276;398
19859;29419;32791;34737;38064
83

3;25710;26422;28808;28883;29021;30892;32111;32112;34835;37624
2113;13073;13078;13080;25404;26197;26237;27926;37836

396;407
3;10688;11363;18567;27454;32349

364
0;14355;16934;19095;19096;20646;26189;29175;29220;32748;36150;37411
30;20367;21050;27490;29465;33087;33090

595;20292;29048

5;9882;33018

54;20520;28104

15184;23553
1;6901;7225;7230;7293;8616;11951;18274;22360;23651;26064;28844;31446;33674;35670;36429;36923;37172
11817;19413;29197;34595

759;22766;31958
21

297;25462;25984;32718;35205
3;9831;22006;23914;29672;34499
1425
1529;19241;35876;37246;38422

0;20236;20374;25352
5421;27344

4064;24068;32008

54;22723;26103;30059;30309
54;22723;30059

63;18705;19942;20215;21663;22393;24672;32103;37001
134;18529;20079;21691;25446;31500;38131

3;11826;12187;12290;14414;15022;18421;19579;20300;24014;24019;29353;34384;35634;37318;37434
5;24327;29113;33707;37142

302;313;398;413

58;10169;14102;15371;15860;16941;17232;17458;17932;22780;22901;25108;25171;27575;27577;27921;28451;29093;30
118

78;21449;21829;22383;25085;28158;28374;28781;31132;31295
17812;22172;25451;25958;26181;31545

4;13957;20249;22045;30173;30346;34128
33611;33776

56;13251;16403;18457;22610;22827;23508;23929;27040;27094;27928;29532;29534;31403;3

084;24366;27987;29720;36458

1;2;93;293;345
221

11722;15982;18053;18589;19537;20271;21443;26100
5644;34513
26014;31981

9;10393;10559;12544;14508;20121;21184;21187;30013;34309
89;118

8

37;16714;19885;23396;23983;27420;28123;30005;36164
9;5952;6570;7776;8448;9428;10215;10618;11611;12504;12629;16003;19232;20360;21527;21533;22365;23042;26331;283

1;10746;11294;36802
259;262;296;469;493

7834;8313;9066;21864;23072;23641;33842;38206
5651;17092;32532;37514
5725;35503;37447;37588
2785;3228;5388;5769;6356;6728;7359;8112;8684;8869;15189;16649;21089;21092;26948;28250;29605;29606;29607
52;11715;16357;17649;18418;24987;25052;27117

79;35503;37447;37588
1;7351;9708;9954;11750;15683;15821;19030;19149;22639;25004;27526;31151;31279;31743;31755;36273;37351
316;35211;35220

43;21158;22391;26452;28109;28525;35521
13946;15163;17908;21414;21524;26443;32582;36075

555;11798;11811;16645;21611

3
4;18683;21517;25498;29867;38351
2;6859;7763;10197;10618;11035;11279;11611;12629;12840;12841;13055;16003;19228;20214;20338;20344;22365;23027

3;6798;6807;11172;11180;16157;16456;21044;28533;32497;37156
4217;37152

52;19298;19299;22030;24773;24780;25702
34;13795;20240;21151;21939;28540;31576;32185;36639
5;15699;16986;18559;19863;20749;20752

9;8392;8764;14266;19925
45;23238;30037

4062;6185;7038;7793;8041;9160;10180;11023;11736;12423;13163;13235;16975;17481;20149;22394;23857;24803;2501

380;11884;27000;37197;38389
322;15902;33399
1

481;15182;18809;21769;22678;23339;30588;30697;35912

005;17368;19348;19393;19396;21246;23468;25705;30416;32644;35107;38167
897;25762;26540;27234;27272;28931;28993;31515;34918;35769;36086;36289
15;20252;22087;33633;38602
19;30441;30788;35194;35525

578;1897;3200;6238;8995;18209;19014;24191;25211;34344

320;18680;23936;24976;24977;25704;26136;38115

10394;11530;18553;20695;29442;37596
3;7265;7518;8179;10839;12736;17725;19455;23437;27378;30746;32162;32505;32702;36407;36833;36837

1009;35402
0503;22293;23698;25432;26498;33032;34584;34591;37251;38374
1;9440;11208;11403;14131;15544;16724;17743;23110;25327;27715;27779;28021;28210;29702;33912;34472
05;29570;33176

3;6193;7264;8305;10839;19454;20351;22013;23427;27378;32162;32512;34391;36829
2545;8894;8896;17477;18405;22916;27285;32066;33752
51;26731;37544
32;18291;26478;27478;28054;29370;30374
5960;38094

385;21145;24892;27751;34311

71;10944;12824;14095;16602;20576;26431

7820;38147
355;14021;16121;16126;17038;24177;25995;31839;32168;36399;37109;37333

5924;6380;7419;16166;21373;21954;22664;26463;32327;37491
8489;9538;9623;18149;18982;19001;23294;24913;26280;28046;31070;36103
3;14960;28689;35373

396;8520;9288;13289;15592;18566;21289;23867;27811;37386
774;21051;22719;24505;25067;29604;31567;33554;33775;35964;36002;37422
18;127;173
8528;14219;14564;15145;15705;17780;24973;26115;27573;33261;35156;36573

7;11713;12214;13771;17532;19111;19780;20759;21505;23166;24071;27486;27653;33888;34022;37350
10327;11790;14313;14338;23057;29510;30537;38342
92;99

52;27797;29855;30815;31550;34738
91;15517;16570;26559;29522;30286;38309

200;352

1;73;164;257;267;299;300;388
9376;21478;21761;21763;36330;37024
25796;36715
0;6519;10675;19697;23244;27807;33897;34346;35547;36804
444;21204;23218;23487;29814;34806;35648

10498;11680;11922;12516;16683;20101;22512;25468;31522;31662;37730
21775;32319
65;81;278
55;146;239;249;281;282;312;345;370
29035;32299

40;16536;25435;36680
4;8109;9043;9103;9632;15153;15158;15776;16988;17593;17595;19870;20706;22220;23632;25134;26483;27151;29576;30
9756;20449;28135;36560
193;11728;12800;15451;16612;16614;16814;16893;21371;22058;22642;27174;29004;29521;31960;31963;35707

332

61;93;122;549

4;5588;6045;7722;7942;8187;8285;8395;8880;8922;10145;10725;10949;11437;13784;14314;14783;14994;16533;17145;1

47;10498;11680;11864;11922;20101;31522;31872;37730

64;32658;37849

9;7345;8718;11111;30732;35163;36161;37711

550

131;149;783

1;8422;12566;16283;16334;23074;23077;27979

66;16276;16281;16331;27953;27958;27963;28435;28446

01;29446;33911;34827

31;11409;11691;13601;18220;19921;21963;36286;37985;38006

23;19928;29391;30191;37376;38009;38345

4;5069;14184;36121;36122

320;2785;3373;4717;4721;5388;6356;6728;7359;8112;8684;8869;9934;14168;15071;15189;15274;16649;18047;18882;18

2208;3257;4273;4550;4559;4765;8480;8938;14243;14422;15531;16517;16986;18039;18153;19863;20749;20752;20817;2

1425;2372;3269;6456;6895;7368;9774;10384;14134;17380;21888;29827;32132;34461;34522;34962;36217;36345;36502;

9;5714;11482;12950;15991;17733;23062;23662;23667;24583;28966;36704

7338;31389;37914

476;484

8056;9373;10155;10518;11139;11564;19956;31831

55;16453;18861;23270;37479

681;24954;31433

4;12875;15114;32017;33547;36035

446

1

52

45;16001;16774

92;12858;13855;15089;25685

4330;5597;7790;12033;16031;18214;18926;26942;28851;35363

70;13633;14530;24993

0;10489;12545;13861;14631;15109;15868;15874;16567;16760;24557;24706;24707;25536;32470;36157;37878
155;220

759;18488;23395;23645;30417;33248;36350
170;173
3;9085;14748;14788;20427;21406;33820
6815;8053;11120;14714;21522;23422;25774;29540;32386
68;343
257;294;467
109;22675;32269
6;2503;6903;9169;14171;14172;18034;19418;23288;24678;24834;24835;25992;26711;28383;29762;37469
822;16698;35948
6;6576;6602;10794;12833;16034;18608;27714;34846;34989;37595
3685;6641;7148;12604;15207;18841;19878;24333;24808;35788;37145
7;8418;13081;16259;17548;23763;28068;28755;29392;33928;33932;34345;34412;35555

1;17757;17919;38354

9;5499;6246;6892;8238;8709;9860;10631;11250;12059;14043;16287;19689;22070;23519;24186;24787;25162;27901;2790
2;16045;18836;34020;36464

1;8177;17280;21254;24315;27441;27918;28802;29223;30129;37993
1;8691;10599;12864;14874;16972;18399;20467;21612;22999;23792;24887;26441
417;14673;16592;16638;22406;31423;32911;32919;35299;36701

7449;19785;32367;34190

3859;27322;32532;37514

7;12023;15918;25443;30176;33855
2;7671;8974;9005;10388;17068;18773;19730;19966;20008;20669;26675
5;4716;6401;7318;7381;7384;7508;7992;8083;9201;9411;9437;9455;13064;13990;16048;16496;17590;19424;20031;2030
10;16449;16754;21265;22148;26283;26592;34265;38288

91;36440;38400
1;8433;11441;12651;13237;14329;15527;15797;16790;18698;20544;21182;22595;22904;232
324;14064;16609;24353;38393
35;10781;10792;10907;14251;14378;14386;16755;16960;18619;22215;26702;30495;37374;37801;37895;37900

367;369;457;558;571;592;596

4;9312;9314;9801;11116;12581;14724;14769;18020;18023;20015;30939;31000;32476;32661;32731;35007;35544;38381
5;5437;6095;14001;35857;38148

3;17804;18781;19402;22133;22668;24889;37305

2;5247;5254;5449;5932;6236;9913;9918;10644;10736;13459;14017;18978;21666;22247;23253;24469;24471;25142;2537
345
26376;33552

16;305

3;12982;16397;29615;38047;38560
5;10001;16081;18913;18914;32699;32796;38001
27739;31999
2;18408;19726;21513;22713;26549;37681
14148;14149;32557;33113;35690
170;20982;26590;29541;35685;35983;38467

1;6817;23258;23756;25988;26642;35950;35953
84

086;17551;17679;19702;22944;31606;33002;33003;34474;36304
2;8273;33486
725;17072;18031;18313;23345;36228;36229

5;6384;10085;17698
9;15767;18192;32026;35841;36553;37604
9;3707;5399;16317;27226;31843;35290;37052
1;15511;32450;32853;32855
075;24902;29645;36052;37723;38535

274;38277;38296
62

16;305
56;22808;24750;31072
102;276;404;410
302;313;377;398;413
1;73;164;257;267;299;300;330;363;388

507;21486;21960;25177;38520
25895;29656

384;1408;1998;3665;3687;4108

53
86;9944;11028;23922;26558;37356;37780
082;21328;25582;29623;31734
07;20362;27355

58;20122;24059;28062;31800;35255
133;425
2;7633;7725;7942;8187;8880;8922;8982;11437;12972;13303;13658;13787;14314;14858;14991;16533;18204;18282;1831
5;10116;12354;13177;14555;14873;16015;17367;18035;18701;23806;24197;24701;24886;25041;25580;26219;27816;298

102
76;16629;19283;20654;30949;32981;38054
79;32920;37188
30;47;54
2;11669;13033;14498;16997;17294;17298;20380;21859;25358;25359;25556;32325

17777;18054;18852;22965;25847;33134;33814

41;15326;20548;29689;33238;33879;38209
4128;15782;15784;34744;34837;34841;35010;35246;36233
6;5935;8429;9820;10992;19273;19451;22685;23544;23586;23650;26636;34649;37873;38030
3;10358;13386;14222;14710;21048;21271;25397;25949;28595;28707;29647;30234;30967;31889;35349;36139;36277;368

5;13152;19893;19899;25180;33129;34525;35244;36382

59;10910;11267;18613
12958;15985;16974;18342;20151;21266;21428;30741;34192;34193;35149
8650;9939;19198;20109;20542;21453;21914;24335;26350;27370;28326;29025;30196;30699;31510;34408
1425
73;164;257;299;300;388
7560;23771;32300;32844
70;12240;12848;17829;17833;23452;33582;34316;38523

9;10811;14061;15654;37822
1;18038;18565
5585;28073;29619;36945
5013;20601;25543;26846;32605;34666;35443;35578;37193
73;257;267;299;300;330;363;388
736;4746;5285;5425;12218;15054;17836;18432;23681;27135;27919
35565;37331

238;265
47;17741;25829;25831;25936;32341
1;9316;10689;11753;13220;14096;19819;20567;20673;21339;22084;24366;25330;25720;27987;28842;29417;29720;3040

6075;8183;13588;18411;24045;33071;33078;33094;37077

5855;9733;10718;13408;14216;14721;19263;21438;21987;22408;24316;24927;26489;26829;26894;28223;28967;29682;3
7963;9256;9496;12234;12439;12442;13134;16267;17142;19879;20941;23760;28748;30659;32495;33280;34396;34406;35
12854;35767;36941;38120

86;228;238;260;263;301;365;404
5;7928;10299;11861;11916;12979;13238;15063;19522;19753;21999;28461;28940;32297;33908;35789;38368
4816;34822
9;13221;14091;15420;22409;23340;23496;24301;24751;25852;25929;26132;26239;28029;31001;31443;33559;33640
438;977;989;2048

56;18593;32276
2594;4588;8337;10850;17881;18840;19783;19844;19938;20667;20716;29563;31301;36283;36344
137;166

7;9033;9389;15196;24517;24871;26666;27163;27785;29171;29658;29664;32975;34427;36059;36798
5326;20548;29741;33238;33245;33879;35451
27;34858;38495
73;22952;24769;30336;32340;38311
14;16101;27508;34082
7807;19108;24288;27485;28592;34637;34643;35413
11268;12524;13813;14135;14155;15571;16476;16900;17105;18470;20076;21062;22143;22537;22851;22911;23280;2435
10492;20809;24978;30829;31939;31940

73;257;267;299;300
4839;28728

5;24684;26624;28364

7448;29181;30973;31957;36020
35;7710;10249;17589;20491;22968;26595;29012;29523;32905;33050;33321;36541
2;5247;5254;5449;5932;6236;9913;9918;10644;10736;13459;14017;18978;21666;22247;23253;24475;25142;25373;2541
964;3236;4529;4856;9345;13838;14853;15890;16540;19282;20185;21821;21825;22652;24088;30825;33792;36506;3804
12;148
012;22137;23987;24246;26121;28453;29599;30865;31925;35041;37103
1;16773;18140;28173;36789

5;7656;7937;8046;17730;20556;25266;26412;34742;36410
560;32461;37732

186;30396;34746
1;13399;15681;21479;31402;31840;31913;32478
8219;11374;11375;15887;16318;17746;19289;21980;28596;34058
3507;15200;16666;25087;26890;31806;34280
237;18440;19563;22741;25573;26027;26095;31344;38271

2;10080;12291;24841;26661;26886;27168;30241;33495

22694;25922;27927;35357;37011
31;20106;35822;38292
3;6228;7703;7804;8766;9229;10456;10932;11630;11697;12199;14504;15973;16113;16173;19203;19268;19584;20376;21
5;7335;25388;31390;34270;36889
2;13722;15670;32094;32901;34516

0;5465;7569;8011;8541;9139;13356;15692;16399;16574;18345;28088;28355;30630;32934;33153;34554;38579
0;6497;8516;12969;16819;17779;18576;28837;31841;32864;33447

320;24324;27692;29047;30591;35691
3;23683;38005;38133

9;2878;2986;3885;6744;10566;13553;15092;15099;17493;17500;17953;17959;20718;22435;23973;24687;24689;28961;2
9962;11531;26998;34445;36696

5674;5830;6179;7352;7664;8459;10733;12450;15665;15667;15800;16494;17178;17234;19009;22123;24040;24042;2558

70;29458;37034

5374;25723;30272
9600;12576;21834;26330;31496;37872
0;28089;28353;33106
3;8367;13002;19996;21520;25928;26028;26150;26309;28329;31371;32392;38018
6281;31134;32091;32816

1138;21900;36281

5;22253;26933;32023;32095

286;25325;28184;28420;29544;29550;30140;30448;33294;35534;35977;37693

7991;13756;19134;19939;23521;24452;28612;29747;31202
466;19312;19368;20302;21053;23871;25055;29067;29160
5962;36964
05;14897;15977;16938;20866;21286;21618;21621;24719;29568;30217;31553;31687;32428;32765;32997
46;13123;26086;36439;38380

1;7517;12374;13857;14804;17173;21047;21256;21944;23054;23787;24046;27810;27891;34300;37988
7;11591;20166;31090;33444;35179
52;25744;26052;26564;29205
12914;13714;27571
7349;27593

2570;13292;25022;26017;30249;30879;37308;38343
1;73;164;257;267;299;300;330;388

3;1998;3155;3775;3783;8532;8798;9211;11013;11626;12659;14407;15257;16084;19146;22776;22971;24233;26977;2893

713;12959;15067;21475

1967;15745;16738;27162;29763;30936;32859;34448;36739
33
57;20507;23636;25572;32707
04;15969;19682;30020;32221;36722
23080;32484;32899

618;27059;35176

6004;35870

02;27037;27336;30442

0734;21074;21134;23796;26367
791;878;960;963
3;23007;35516;37169

4625;6034;6373;6536;7956;12156;14123;15028;15180;15191;15594;16056;16672;19281;21878;22578;22734;23133;2327
619;4308;4802;7315;8505;9777;10174;13789;23655;24025;25291;25997;26312;29358;30684;31703;33480;34073;35700;
16;32416;36397;36633
32;12901;12904;16833;22974;26968;30216;32751

26614;27656;35657;36767

5617;6811;7000;8168;8178;9329;9595;10430;12698;13972;13974;16507;17254;18375;20372;20857;20886;22817;25401;
2835;33303;33828;35558
3;3046;3052;3714;3898;4302;4381;5759;7603;8096;8233;8466;8615;8692;8866;9016;9976;11794;13201;14162;14458;157

3;11512;11910;12330;13754;14240;14264;23390;24215;24313;24388;25113;25440;25654;28097;28959;29920;30219;307
38
25;15101;18486;19503;31464;35160
145;155;206;207;267;300;373
9;20647;26104

2004;37704

43;15423;15687;15842;17526;24050;25715;26945;27527;30271;30578;36201

29751;36111
92;3648;25378;26415;28735;29375;30910;31453;31454;32654;37435
3605;37012
34;22650;29765;33906
33;21237;24534;24536;24813;25169

3;22171;24578;30435

7888;33452
753;13569;13960;17905;18686;21783;28285;31349;32457;33329;34521;34594

847;2848;2853;3221;3227;6575;14393;16653;17853;17861;20559;21103;24859;25990;26853;26883;26928;33375;33386;

1370;14320;15813;17482;18206;23770;24635;25016;25425;26437;28455;29019;29753;31360;34490;35076;38151;38383

307;19970;36271
497;29037;29351;29365;32928;38208
9261;11799;17834;18804;20922;21360;24193;26715;31472;31764;34706;35989;36966
50;57
1514;21733
4730;29168;34365
56;25590;26149;31798

959
14125;15649;18601;18602;23249;28617;31374;33763
3;10276;21421;21679;25812;30711;35241;35868;36296;37007
7340;18853;19975;30066
3;3829;4250;5923;8114;12022;14038;14339;18753;22132;23053;24360;24951;25647;27131;28726;30739;31347;34555;36

0;7009;8206;11201;12222;18963;19961;35012
257;267;299;300
1938;24122;28040;31097;36616;36842
1;19829;21772;24873;27897;32426;33132;36198
26628;33985
089;19193;24730;29168;34366
7999;38516

1;27078;28654;32085;34747

5650;7307;8436;10683;12712;13228;14729;18991;25609;27364;29987;30004;34720;35037;35509

18199;27536

522;27147;31289

8672;8675;12532;16466;16888;20118;20364;20901;21329;21334;22757;24219;26158;27721;29333;30390;30838;31678;3

74;13757;14234;14236;16591;18532;22879;31105;36421

55;27436;35304

00;15149;26177;26345;29177;32846;35847;38414

040;20433;21482;28138

98;22199;32889

204

7;4491;4713;5572;7089;7953;8580;9161;10334;11336;12056;12489;13178;13271;13370;13378;13692;13696;13726;13820

795;3255;3256;7568;8642;9833;10463;11724;11974;12194;17850;19128;20312;20453;20919;20994;21244;25030;28234;

561;20155;20192;21359;30705;33369

757

99;17100;21717;28289;30834;34762

8538;9797;12137;15122;16506;16880;16882;18136;18292;19722;22202;23691;27143;29917;34726;35071;37160;38486

25;18179;21503;22679

9979;10174;23784;25291;29358;31703;35912;36011

69

1;15685;18899;18934;20128;20497;21327;21416;21649;23240;24639;25155;32652

4;7699;8031;8047;9723;10162;10164;11198;11787;14335;15034;15961;18568;18625;18627;19222;19287;19849;22186;25

3194;13230;18859;36220;36227;36817

10;31847;33965

595;30896;33634

109;33656;35573
33478;34147
7;9004;9768;19532;19761;19768;22102;22305;24402;27080;28014;30099;30453;30890;32322;35125;37727

9;8062;22128;23097;24355;25340;25731;28066;32227;33635
320;28909;31716

29;21115;27463

736;21553;27244;29059;37814

4;9949;17417;19189;20822;20852;22676;31876;37518;37631;38362
4;7233;7944;10298;12578;13620;13805;13976;15889;19506;20522;20694;26093;30517;34008;35705;35898;38398
34;20864;26900;34083

49;18010;21011;25309;28569;29029
374;15549;17567;18363;26285;28397;30564;32006;34235;37609
44;16093;17653;25183;26402;29321;30422;32838;34068;36825;36969;37917
7185;7391;7396;8203;9900;10692;13914;15382;22097;22495;26176;27099;27394;27824;28885;29835;30665;30682;3130
0;8902;8998;10242;11684;12536;12590;14952;16690;19997;19999;25018;31785;33333;33716;36354;37241;37474

3587;28531;33705;35033;36973;37120
7310;38541
1
2;9686;12710;14342;18515;23191;30261;32298;35850;36995;37702
573
5;9838;12862;14229;14232;14888;16742;19208;20461;20789;21086;30184;33366;34259;35730;37581;37806
5;7702;8213;10464;16511;17141;23509;23514;24385;24747;25393;27086;27219;27951;28298;28301;29489;31566;31681
7695;9927;17807;17875;19107;22142;24288;27485;28594;34637;34643;35000;35004;35413

9;7971;12311;15443;20274;20278;22189;25998

77;18649;21310;23654;24677;27531;29460;38101;38451
5305;7075;8023;10524;13276;16779;19382;19518;19729;20332;20815;23647;24164;24770;24970;26514;28652;28763;28
4457;34621
3;9945;10404;10575;12397;14025;17091;18632;19826;20321;22550;26679;28343;30019;34865;37335
5700;5921;7127;7733;7735;11891;15324;19441;21459;22845;22875;24542;25117;26951;31220;34854;36109;37633
28998;34694
3241;30077;30081;37023

815
214

617;12664;13711;16365;19786;27488;28429;28463;30108;36917;37367
9;9872;16677;22358;31581;35704

26857;29832;30440;31190;33159;33766;33858;36438

66;33770;35161;37032;37138

272;18574;20955;28706;31075;31538;32692;34010;35883;36255;36744

2;34563;34922;36950

8;32988;37381

21171;21403;21790;23246;25324;29001;30023;34527;34648;36335;36336
26862;27766;30326;30573;30576;32740;32863;34793;38447

3;25801;26172;29358;31703;32291;35838;35912;37317

158;25954;26922;30839;30906;32739;33119;36846

4;23210;23535;27243;30531;32489;32598;34178;35165;37741

425;17001;18369;20516;22478;23299;23333;27101;27104;27233;27769;29023;32053;33148;34453;35142;35419;35647;3

73;273;273;273;273;273;273;273;273;273;273;273;273;273;273;273;273;273;273;273;273;273;273;273;273;273;273;273;2

3;36152;36503;37592

1175;32315;37017;37639

22920;23957;25180;27416;34525;35242;36390;37407;37473

365;25292;26228;26565;27773;28058;28638;29847;30439;31335;35306;36566;37183;38172;38528

4088;30825;33792;36512;38040;38045

9853;30669;34434;34539;37059;37060;37200

4;21604;25132;26640;34771;35659

18;21020;23350;23398;23408;23457;25043;25627;25907;25916;28246;29578;30322;30356;31320;31323;37428;37624;37

344;25338;26127;27611;28458;29655;31572;32292;33532

;21641;22493;26203;30874;35963;37803;38328

66;29467;34700;36694

400;32895;33155;33894;33896;34451;36516

398;23408;23457;23719;25627;25907;25916;28244;29578;30322;30356;31320;31323;36409;37428;37624;37661

1583;36089;38415

123;25125;25620;25983;26954;30735;30886;36630

32688;34283;35753

9041;32742;32881;32886;34714

684;35660;35776

7895;37900

26311;31003;31882;35911;37373;38165

019;37862

7;33786;34860;35651;35653;35832;36455

185;23575;23780;24530;24764;25739;26170;27046;28128;29358;31046;31221;31703;31756;32446;33395;33665;35912;3

81;30882;32545;32908;37313

20;23350;23398;23408;23457;23719;25043;25627;25907;25916;28244;29578;30322;30356;31320;31323;37428;37624;37
;31861;32554;37624

6429;36923;37172

318;37434

7577;27921;28451;29093;30424;31749;31823;32193;32351;32623;32794;35353;36773;37235

32576;32710;33475;36416

1533;22365;23042;26331;28312;30212;30612;30760;32383;32590;33831;35813;37329

250;29605;29606;29607

;31755;36273;37351

4;20338;20344;22365;23027;26331;26495;26709;27202;27211;30226;30760;31482;31491;32120;32462;32463;32866;33

49;22394;23857;24803;25012;25182;25689;25863;25892;27139;27388;29449;29949;33126;33383;33544;34277;34330;34

;36833;36837

02;33912;34472

022;37350

25134;26483;27151;29576;30696;32972;34299;34945;35046

31960;31963;35707

14783;14994;16533;17145;18204;21008;22456;24344;24727;25499;26369;26610;26634;28032;28774;28947;32585;3316

15274;16649;18047;18882;18993;21089;21092;26948;28250;28523;29605;29606;29607;31041

19863;20749;20752;20817;20996;21302;21711;21992;22466;24310;25605;29111;29152;29156;33756
2;34962;36217;36345;36502;36814;37490;37793

470;36157;37878

3;29762;37469

186;24787;25162;27901;27904;28302;28578;28587;30672;32267;34250;35596;37866

96;17590;19424;20031;20308;23517;24240;24643;25940;27630;27828;28231;28417;29685;32049;35654;37610;37922

281;23347;23518;23968;24408;27496;30632;30783;31129;31474;31887;35069;35074;36643

7801;37895;37900

;32731;35007;35544;38381

53;24469;24471;25142;25373;25410;25848;27831;28560;29996;31160;31379;32517;32563;32811;32930;34953;35916;38

91;16533;18204;18282;18319;19080;24493;25499;26904;27798;28767;29030;30070;31227;36702;37298;37467;37857;37
041;25580;26219;27816;29829;30628;31254;32593;32974;33973;34493;35173;37058

889;35349;36139;36277;36876;37304

;31510;34408

87;28842;29417;29720;30400;31040;31195;34979;38015

;26894;28223;28967;29682;31431;33876;35687;36050;38460
32495;33280;34396;34406;35080;35088;35187;35190;36127

08;35789;38368

001;31443;33559;33640

37;22851;22911;23280;24350;25268;26020;26024;26154;26231;27069;27431;27601;28553;29627;30895;31145;31146;3[illegible]

53;24475;25142;25373;25410;25848;27831;28560;29996;31160;31379;32517;32563;32811;32930;34953;35916;38116
38;30825;33792;36506;38040;38045

9203;19268;19584;20376;21766;21880;22208;22527;24411;24415;24647;24651;26151;26605;27150;27169;27276;28005;

3153;34554;38579

23973;24687;24689;28961;29768;30412;30712;30715;30767;30986;30990;31007;31915;31918;33914;35987

09;22123;24040;24042;25589;28018;29873;30532;32328;34351;35601;36851

2765;32997

76;22971;24233;26977;28936;29041;32742;32881;32886;34714;37601

378;22578;22734;23133;23273;25957;28637;29492;30622;31604;32054;32482;32763
4;31703;33480;34073;35700;35912

2;20857;20886;22817;25401;26921;36675

1794;13201;14162;14458;15730;16359;16752;16766;16812;17537;18259;18437;19367;19796;19798;19950;20496;20569;

097;28959;29920;30219;30790;31730;32058;32826;35933;37151;37855

3;26883;26928;33375;33386;35585;38437

0;34490;35076;38151;38383;38387

28726;30739;31347;34555;36010;36144;36991;37413

;29333;30390;30838;31678;31856;34165;36185;38470

78;13692;13696;13726;13820;14523;14867;16524;17101;17137;18093;20702;22548;22782;23799;24069;26847;27045;27

9;20994;21244;25030;28234;33944;36285;38511

;34726;35071;37160;38486

19222;19287;19849;22186;25399;26159;30570;31739;33571;33573;33664;33902;35172;36430

2;35125;37727

8;35705;35898;38398

385;29835;30665;30682;31305;31470;31527;33014;33015;33022;33178;33257;33677;36294
6;36354;37241;37474

30;37581;37806
8;28301;29489;31566;31681;31686;33687;33870;34758;36299;38134

24970;26514;28652;28763;28804;34399;35909

220;34854;36109;37633

;34453;35142;35419;35647;37736;38033

3;273;273;273;273;273;273;273;273;273;273;273;273;273;273;273;273;273;273;273;273;273;273;273;273;273;273;273;2

1320;31323;37428;37624;37661

37428;37624;37661

;32446;33395;33665;35912;37302

1320;31323;37428;37624;37661

2120;32462;32463;32866;33864;37392;37395

3383;33544;34277;34330;34331;36586

32;28774;28947;32585;33162;36900;37857;37994;38428;38433

32049;35654;37610;37922

2811;32930;34953;35916;38116

6702;37298;37467;37857;37997

29627;30895;31145;31146;31475;31912;33614;36955;36957;38462

2930;34953;35916;38116

5;27150;27169;27276;28005;29494;29641;29869;29942;30093;30567;31974;32081;32083;32217;32352;32670;32675;329

18;33914;35987

6;19798;19950;20496;20569;21652;21894;23070;24229;24405;24830;25860;26538;26926;27911;28381;28745;29017;292

3799;24069;26847;27045;27580;27910;27989;28227;31944;32013;34322;38506

3;273;273;273;273;273;273;273;273;273;273;273;273;273;273;273;273;273;273;273;273;273;273;273;273;273;

[illegible]217;32352;32670;32675;32926;33598;33954;36124;36654;37455

7911;28381;28745;29017;29263;29268;29269;29594;29889;30161;30636;30641;30849;30856;30858;30876;31035;31063;

273;273;273;273;

[illegible];30858;30876;31035;31063;31479;31690;33604;33819;34442;34832;34834;36348;36770;36812;37380;38521

812;37380;38521
