# Supplementary material for: The HDAC inhibitor SAHA regulates CBX2 stability via a SUMO-triggered ubiquitin-mediated pathway in leukemia
Source: Oncogene. 2018 Feb 22;37(19):2559–72. doi: 10.1038/s41388-018-0143-1 (PMC5945585; doi:10.1038/s41388-018-0143-1)
Supplement: Supplementary file 3 — Supplementary Table 2 GO 6h saha k562 u937 [file 41388_2018_143_MOESM3_ESM.pdf]

GO TERM BP3 COMMON ALTERED GENES UPON 6H SAHA TREATEMENT IN K562 AND U937	%	PValue	Fold Enrichment	Bonferroni	Benjamini	FDR
GO:0006996~organelle organization	30.25	4.01E-18	1.463790224	4.65E-15	4.65E-15	6.46E-15
GO:0034641~cellular nitrogen compound metabolic process	46.14	3.32E-16	1.288324372	3.86E-13	1.93E-13	5.33E-13
GO:0044260~cellular macromolecule metabolic process	56.25	2.48E-15	1.218126034	2.83E-12	9.44E-13	3.93E-12
GO:1901360~organic cyclic compound metabolic process	42.9	4.32E-13	1.267189712	5.01E-10	1.25E-10	6.96E-10
GO:0046483~heterocycle metabolic process	41.51	6.54E-13	1.273811823	7.58E-10	1.52E-10	1.05E-09
GO:0070727~cellular macromolecule localization	14.74	8.19E-13	1.659473569	9.49E-10	1.58E-10	1.32E-09
GO:0006139~nucleobase-containing compound metabolic process	40.74	8.48E-13	1.277105489	9.83E-10	1.40E-10	1.37E-09
GO:0006725~cellular aromatic compound metabolic process	41.67	1.16E-12	1.268687473	1.34E-09	1.68E-10	1.87E-09
GO:0043170~macromolecule metabolic process	58.8	1.16E-11	1.169648945	1.34E-08	1.49E-09	1.86E-08
GO:0044249~cellular biosynthetic process	42.13	1.18E-11	1.250703916	1.37E-08	1.37E-09	1.90E-08
GO:1901576~organic substance biosynthetic process	42.67	1.89E-11	1.244565063	2.19E-08	1.99E-09	3.04E-08
GO:0007049~cell cycle	14.66	3.73E-11	1.592533833	4.32E-08	3.60E-09	6.00E-08
GO:0051726~regulation of cell cycle	9.568	2.42E-10	1.780505039	2.80E-07	2.15E-08	3.89E-07
GO:0008104~protein localization	19.37	5.69E-10	1.436923501	6.59E-07	4.71E-08	9.16E-07
GO:0045184~establishment of protein localization	16.13	1.24E-08	1.449276707	1.43E-05	9.56E-07	1.99E-05
GO:0033554~cellular response to stress	14.51	1.31E-08	1.486005625	1.52E-05	9.52E-07	2.12E-05
GO:0031325~positive regulation of cellular metabolic process	20.91	1.00E-07	1.337750858	1.16E-04	6.85E-06	1.62E-04
GO:0009893~positive regulation of metabolic process	22.15	1.55E-07	1.317858273	1.80E-04	1.00E-05	2.50E-04
GO:0010604~positive regulation of macromolecule metabolic process	20.91	1.85E-07	1.329788055	2.14E-04	1.13E-05	2.97E-04
GO:0051649~establishment of localization in cell	15.43	4.06E-07	1.398632508	4.70E-04	2.35E-05	6.53E-04
GO:0060255~regulation of macromolecule metabolic process	38.12	5.62E-07	1.19055255	6.51E-04	3.10E-05	9.04E-04
GO:0007050~cell cycle arrest	3.086	6.13E-07	2.385412379	7.10E-04	3.23E-05	9.87E-04
GO:0043933~macromolecular complex subunit organization	18.21	6.64E-07	1.343914776	7.69E-04	3.35E-05	0.001069
GO:0022613~ribonucleoprotein complex biogenesis	5.015	8.48E-07	1.897769484	9.82E-04	4.09E-05	0.001365
GO:1902580~single-organism cellular localization	9.414	9.01E-07	1.551492126	0.0010433	4.18E-05	0.00145
GO:0010941~regulation of cell death	12.11	9.05E-07	1.458081341	0.0010482	4.03E-05	0.001457
GO:0010942~positive regulation of cell death	5.941	9.83E-07	1.777760997	0.0011382	4.22E-05	0.001582
GO:0008219~cell death	14.97	1.09E-06	1.388546353	0.0012609	4.51E-05	0.001752
GO:0080090~regulation of primary metabolic process	37.81	1.31E-06	1.184374937	0.0015173	5.24E-05	0.002109
GO:0046907~intracellular transport	12.58	1.51E-06	1.433080634	0.0017521	5.85E-05	0.002435
GO:0048523~negative regulation of cellular process	29.09	1.57E-06	1.228695406	0.0018159	5.86E-05	0.002524
GO:0019538~protein metabolic process	35.8	1.64E-06	1.191613367	0.0018993	5.94E-05	0.00264

GO:0031323~regulation of cellular metabolic process	37.89	2.76E-06	1.177450408	0.0031991	9.71E-05	0.00445
GO:0048522~positive regulation of cellular process	31.56	2.77E-06	1.20823098	0.0032002	9.43E-05	0.004452
GO:0045786~negative regulation of cell cycle	4.784	3.73E-06	1.848694594	0.0043188	1.24E-04	0.006011
GO:0051171~regulation of nitrogen compound metabolic process	29.48	4.16E-06	1.215590766	0.0048053	1.34E-04	0.00669
GO:1903047~mitotic cell cycle process	7.716	5.43E-06	1.574640194	0.0062768	1.70E-04	0.008745
GO:0022411~cellular component disassembly	4.938	7.80E-06	1.786683376	0.009001	2.38E-04	0.012557
GO:0051173~positive regulation of nitrogen compound metabolic process	13.35	9.62E-06	1.372084611	0.0110849	2.86E-04	0.01548
GO:0009889~regulation of biosynthetic process	28.7	1.89E-05	1.201502364	0.0216466	5.47E-04	0.030389
GO:0010605~negative regulation of macromolecule metabolic process	16.67	2.12E-05	1.301413719	0.0243121	6.00E-04	0.034177
GO:0010035~response to inorganic substance	4.475	5.23E-05	1.733111446	0.0587901	0.0014416	0.084114
GO:0016032~viral process	7.87	5.72E-05	1.479770568	0.0641673	0.0015411	0.092064
GO:0051128~regulation of cellular component organization	16.36	7.53E-05	1.27896303	0.0836149	0.0019825	0.121199
GO:0009892~negative regulation of metabolic process	17.52	8.89E-05	1.261397932	0.0978662	0.0022861	0.142939
GO:1903829~positive regulation of cellular protein localization	3.627	1.02E-04	1.814523397	0.111291	0.0025616	0.16373
GO:0009890~negative regulation of biosynthetic process	11.19	1.21E-04	1.351112481	0.1307459	0.0029768	0.194416
GO:0060341~regulation of cellular localization	6.867	1.25E-04	1.495530573	0.134966	0.003016	0.201161
GO:0051172~negative regulation of nitrogen compound metabolic process	11.19	1.30E-04	1.349318174	0.1397038	0.0030663	0.208773
GO:0031324~negative regulation of cellular metabolic process	16.36	1.36E-04	1.26696423	0.1462857	0.0031582	0.219417
GO:0009891~positive regulation of biosynthetic process	12.73	1.57E-04	1.313840412	0.1666876	0.003569	0.252931
GO:0031647~regulation of protein stability	2.546	1.78E-04	2.028385198	0.1864313	0.0039599	0.286144
GO:0032880~regulation of protein localization	7.639	1.92E-04	1.442219829	0.1992719	0.0041843	0.308173
GO:0044403~symbiosis, encompassing mutualism through parasitism	7.87	1.97E-04	1.432323014	0.2042513	0.004222	0.31681
GO:0044093~positive regulation of molecular function	12.96	4.91E-04	1.279566314	0.4342067	0.0103016	0.787865
GO:0070887~cellular response to chemical stimulus	17.82	5.23E-04	1.222546365	0.4546536	0.010769	0.83857
GO:1901700~response to oxygen-containing compound	10.57	7.61E-04	1.30965811	0.5860216	0.0153536	1.217411
GO:0009966~regulation of signal transduction	18.13	9.14E-04	1.207243389	0.6536551	0.0181153	1.461832
GO:0010033~response to organic substance	18.67	0.0010137	1.201793072	0.691313	0.0197254	1.619236
GO:1901575~organic substance catabolic process	13.04	0.0012107	1.253793709	0.7543965	0.0231289	1.931103
GO:0051129~negative regulation of cellular component organization	5.093	0.0012983	1.482281491	0.7781518	0.0243824	2.069558
GO:0080135~regulation of cellular response to stress	4.784	0.0014969	1.495501651	0.8238098	0.0276146	2.382464
GO:0007568~aging	2.701	0.0016337	1.751787216	0.8496851	0.0296318	2.597565
GO:0090559~regulation of membrane permeability	1.08	0.0020337	2.651353624	0.9055237	0.0361945	3.223747
GO:0050790~regulation of catalytic activity	15.66	0.0022821	1.207001459	0.9292042	0.0399191	3.610803

GO:0006793~phosphorus metabolic process	19.98	0.00231	1.175041474	0.931463	0.0397982	3.654201
GO:0008285~negative regulation of cell proliferation	5.17	0.002343	1.442331717	0.9340404	0.0397655	3.705479
GO:0044248~cellular catabolic process	11.19	0.0024512	1.257471021	0.9418347	0.0409676	3.873514
GO:0043414~macromolecule methylation	2.546	0.0025342	1.738615884	0.9471793	0.0417255	4.002106
GO:0051098~regulation of binding	2.778	0.0027719	1.681715728	0.9599276	0.044918	4.369667
GO:0071166~ribonucleoprotein complex localization	1.466	0.0032781	2.130173255	0.9777545	0.052189	5.148168
GO:0010646~regulation of cell communication	19.44	0.003371	1.169404976	0.9800314	0.0529046	5.290309
GO:0000075~cell cycle checkpoint	2.238	0.0036167	1.774736394	0.9849956	0.0559028	5.665517
GO:0009967~positive regulation of signal transduction	9.954	0.0036811	1.265997484	0.9860784	0.0561245	5.763597
GO:0045787~positive regulation of cell cycle	3.009	0.0037548	1.612264341	0.9872213	0.0564756	5.87565
GO:0080134~regulation of response to stress	9.028	0.0042587	1.276009988	0.9928905	0.0630104	6.639028
GO:0051301~cell division	4.552	0.0046738	1.43798881	0.9956151	0.0680856	7.263573
GO:0009725~response to hormone	6.327	0.0049547	1.345635145	0.9968386	0.0711468	7.683983
GO:0044802~single-organism membrane organization	6.404	0.0049887	1.343171722	0.9969615	0.070745	7.734794
GO:1902589~single-organism organelle organization	10.96	0.0055776	1.234510098	0.9984701	0.0778356	8.609858
GO:0010506~regulation of autophagy	2.469	0.0056012	1.667128354	0.9985115	0.0772253	8.644684
GO:0070482~response to oxygen levels	2.778	0.0057524	1.606734771	0.9987521	0.0783045	8.868134
GO:0044770~cell cycle phase transition	4.321	0.0061509	1.434736148	0.9992159	0.0825487	9.454304
GO:1904816~positive regulation of protein localization to chromosome, telomeric region	0.386	0.0080036	5.83929072	0.9999098	0.1049498	12.13344
GO:0006403~RNA localization	2.006	0.0085187	1.726880289	0.9999506	0.1101056	12.86492
GO:0046677~response to antibiotic	0.694	0.0087316	3.003063799	0.9999615	0.111473	13.1656
GO:0044711~single-organism biosynthetic process	9.182	0.0089195	1.244553306	0.9999691	0.1125088	13.43012
GO:0001101~response to acid chemical	2.469	0.0089245	1.613156573	0.9999693	0.1113639	13.43722
GO:0021915~neural tube development	1.62	0.010674	1.827951878	0.999996	0.1304233	15.86435
GO:1904951~positive regulation of establishment of protein localization	4.012	0.0115218	1.409561861	0.9999985	0.1386345	17.01745
GO:0060548~negative regulation of cell death	6.404	0.0115718	1.296752187	0.9999986	0.1377763	17.08497
GO:0009991~response to extracellular stimulus	3.472	0.012117	1.449754938	0.9999993	0.1423682	17.81815
GO:0023056~positive regulation of signaling	10.42	0.0123209	1.21044798	0.9999994	0.1431577	18.09075
GO:0044281~small molecule metabolic process	12.73	0.0123519	1.185217389	0.9999994	0.1420798	18.13206
GO:0001666~response to hypoxia	2.469	0.0131184	1.568033312	0.9999998	0.1487965	19.14893
GO:0007017~microtubule-based process	4.63	0.01339	1.36061143	0.9999998	0.1501948	19.50628
GO:0010647~positive regulation of cell communication	10.34	0.0140853	1.207662955	0.9999999	0.1559086	20.41463
GO:0044766~multi-organism transport	0.926	0.0153075	2.272588821	1	0.1667606	21.98788

GO:0023057~negative regulation of signaling	8.102	0.0159394	1.24072619	1	0.1714739	22.78985
GO:0071214~cellular response to abiotic stimulus	2.315	0.016223	1.568764671	1	0.172682	23.14721
GO:0006810~transport	28.24	0.0165512	1.099985625	1	0.1742958	23.55893
GO:1901564~organonitrogen compound metabolic process	14.2	0.0171676	1.161022414	1	0.1786162	24.32647
GO:1903749~positive regulation of establishment of protein localization to mitochondrion	1.235	0.0178072	1.933006583	1	0.1830521	25.11533
GO:0010648~negative regulation of cell communication	8.025	0.0188974	1.233068497	1	0.19153	26.44205
GO:0050792~regulation of viral process	2.238	0.0207989	1.551200894	1	0.2070551	28.70368
GO:0007507~heart development	3.858	0.0210355	1.371262009	1	0.2074145	28.98045
GO:0042127~regulation of cell proliferation	10.19	0.0216263	1.191937693	1	0.2108675	29.66719
GO:1902581~multi-organism cellular localization	0.849	0.0216838	2.26701875	1	0.2096342	29.73375
GO:0032204~regulation of telomere maintenance	0.849	0.0216838	2.26701875	1	0.2096342	29.73375
GO:0007098~centrosome cycle	0.926	0.02203	2.156045804	1	0.2109021	30.13297
GO:0009968~negative regulation of signal transduction	7.407	0.0230304	1.235420185	1	0.2176822	31.27446
GO:0006979~response to oxidative stress	3.086	0.0235068	1.422771343	1	0.219933	31.81194
GO:0061024~membrane organization	7.022	0.0239611	1.241773217	1	0.2219567	32.32077
GO:0042594~response to starvation	1.466	0.0246145	1.740337626	1	0.2255645	33.04642
GO:0055093~response to hyperoxia	0.463	0.0248737	3.503574432	1	0.2259206	33.33226
GO:0071496~cellular response to external stimulus	2.16	0.0277544	1.526849558	1	0.2469858	36.43279
GO:0044092~negative regulation of molecular function	7.407	0.0279029	1.223065984	1	0.2462932	36.58888
GO:0001890~placenta development	1.389	0.0292205	1.739705925	1	0.2545527	37.95829
GO:0009894~regulation of catabolic process	3.781	0.0293692	1.34911707	1	0.2538183	38.11111
GO:0009612~response to mechanical stimulus	1.775	0.0295338	1.603626108	1	0.2532143	38.27978
GO:0010256~endomembrane system organization	4.09	0.0296781	1.328725903	1	0.2524695	38.42734
GO:0051236~establishment of RNA localization	1.62	0.0303957	1.644135488	1	0.2559611	39.15626
GO:0051204~protein insertion into mitochondrial membrane	0.54	0.0312089	2.885296591	1	0.260077	39.97246
GO:0051130~positive regulation of cellular component organization	7.87	0.03204	1.208333363	1	0.2642377	40.79613
GO:0043903~regulation of symbiosis, encompassing mutualism through parasitism	2.315	0.0339337	1.475189235	1	0.2757922	42.6332
GO:0043433~negative regulation of sequence-specific DNA binding transcription factor activity	1.312	0.0347268	1.739000448	1	0.2794283	43.3866
GO:0090342~regulation of cell aging	0.54	0.0354736	2.802859546	1	0.2826771	44.08758
GO:0007584~response to nutrient	1.543	0.0359152	1.63909915	1	0.2837976	44.49822
GO:0030308~negative regulation of cell growth	1.543	0.0359152	1.63909915	1	0.2837976	44.49822
GO:0045926~negative regulation of growth	2.006	0.0362642	1.518215587	1	0.2842774	44.8207
GO:0009615~response to virus	2.469	0.0370136	1.441985618	1	0.2874175	45.50739

GO:0042180~cellular ketone metabolic process	1.929	0.0372513	1.529945167	1	0.2871282	45.72349
GO:1900182~positive regulation of protein localization to nucleus	1.235	0.0389767	1.751787216	1	0.2965367	47.26818
GO:0051186~cofactor metabolic process	2.932	0.0396129	1.383229386	1	0.2987489	47.82729
GO:0036119~response to platelet-derived growth factor	0.386	0.0421124	3.687973087	1	0.3126632	49.97063
GO:0044255~cellular lipid metabolic process	6.944	0.0430982	1.209287436	1	0.3168483	50.79295
GO:0090316~positive regulation of intracellular protein transport	2.083	0.0432505	1.478070464	1	0.315853	50.91888
GO:0010243~response to organonitrogen compound	5.401	0.0449439	1.243347074	1	0.3242205	52.2987
GO:0007165~signal transduction	33.95	0.0450422	1.067756017	1	0.3228744	52.37764
GO:0006656~phosphatidylcholine biosynthetic process	0.463	0.0455233	3.003063799	1	0.3238268	52.76243
GO:0035966~response to topologically incorrect protein	1.62	0.0471351	1.565426874	1	0.3314093	54.03021
GO:0032922~circadian regulation of gene expression	0.694	0.0480226	2.212783852	1	0.3346362	54.71454
GO:1990267~response to transition metal nanoparticle	1.235	0.0489599	1.698702755	1	0.3380923	55.42696
